# The Promise of Exome Sequencing for the Differential Diagnosis of Late-Onset End-Stage Renal Disease in Turkish Cypriots

**DOI:** 10.7759/cureus.86797

**Published:** 2025-06-26

**Authors:** Hasan H Kazan, Simge Ozadali, Düriye Deren Oygar, Guy H Neild, Cemal Gurkan

**Affiliations:** 1 Medical Biology, University of Health Sciences, Ankara, TUR; 2 Genetics, Dr. Fazıl Küçük Faculty of Medicine, Eastern Mediterranean University, Famagusta, CYP; 3 Nephrology, Burhan Nalbantoğlu State Hospital, Lefkoşa, CYP; 4 Nephrology, University College of London - Centre for Nephrology, London, GBR

**Keywords:** cyprus, end-stage renal disease, exome sequencing, isolated population, nephrogenetics

## Abstract

End-stage renal disease (ESRD) is a pathophysiological condition that requires radical solutions for treatment. Although ESRD is a worldwide health problem, the incidence of the disease is relatively high in the Mediterranean island of Cyprus. Moreover, the etiology of the disease largely remains poorly understood. Overlapping diseases such as diabetes mellitus may pose a challenge during the differential diagnosis of ESRD. Hence, molecular diagnosis could be powerful in terms of ESRD diagnosis. Nevertheless, the genetic isolation of Cyprus and the complexity of the disease necessitate more comprehensive, population-based approaches. In the current study, exome sequencing (ES) data obtained from 24 Turkish Cypriot patients with ESRD were re-analyzed. Variants were filtered according to the allele frequencies in population databases and the renal anomalies-related gene list. Variants were then prioritized according to the guidelines of the American College of Medical Genetics. The re-analysis underscored that different genes and their variants could explain the molecular mechanisms of the disease. However, numerous variants were found to be prevalent in all patients, limiting the ability to reach a population-based conclusion about the aetiology of ESRD in Cyprus. Although ES could be useful for the molecular diagnosis of ESRD, further population-based cohort studies and a whole genome database with data from healthy participants are still needed.

## Introduction

End-stage renal disease (ESRD) is a worldwide health concern that constitutes a severe problem, particularly for low- and middle-income countries. Moreover, a significant geographic variation in the incidence rates has also been noted [1,2]. For instance, the Middle East is one of the regions where ESRD cases are relatively high. As a part of this region, both Turkish and Greek Cypriots have a high prevalence with an elevated number of renal replacement therapy (RRT) [3]. Determining the primary renal diagnosis is crucial for comprehending the etiology of ESRD and chronic kidney disease, which affect an increasing percentage of individuals. Worldwide, ESRD attributable to type 2 diabetes and hypertension is on the increase, and that is especially significant for places like Cyprus [4,5]. However, lack of clear indications, complexity of the kidney diseases, and involvement of secondary phenotypes still limit the success rate of the clinical and differential diagnosis of kidney diseases in Cyrus [4,6].

As a small island in the eastern Mediterranean, Cyprus is home to Turkish and Greek populations, which are two major populations that seem to share a common, largely isolated genetic ancestry as dictated by geography [7-10]. Indeed, several diseases such as thalassemia and familial Mediterranean fever (FMF) are prevalent in the Cypriot population [11,12]. Because of the isolated nature of the island, the well-known diseases, including FMF, cystic fibrosis, Friedreich's ataxia, 21-hydroxylase deficiency, and breast cancer, could be attributed to founder mutations [13-18]. Thus, for deciphering the genetic variants causing the ESRD, which has even been reported in Cyprus as an endemic disease [6,19], a more comprehensive strategy is required. Incidence of ESRD positively correlates with the individuals’ age. Individuals older than 75 have been reported to have a four-fold higher tendency to become ESRD patients, while individuals aged between 45 and 64 are likely to have a two-fold higher risk of ESRD compared to average adults in the USA [1]. Increased adult age may result in age-dependent secondary phenotypes that could overlap with ESRD. Amongst these phenotypes, diabetes could directly be influential on renal health, causing diabetic nephropathy [20]. Moreover, for Cyprus, where FMF cases are with higher frequency [21], it could be challenging to differentially diagnose ESRD. Hence, molecular diagnosis would be a powerful approach to explore the primary phenotype in ESRD.

Exome sequencing (ES) has become a widely used diagnostic tool for the study of inherited diseases because it offers a comprehensive approach to genetic testing [22]. ES has been effectively used for detecting diagnostic mutations in ESRD patients [23]. Nevertheless, such studies documenting variants in a particular cohort, especially for isolated geographies including Cyprus, are still needed to explore the molecular background of the disease and the identification of putative founder variants. Hence, in the present study, the promise of ES in the differential diagnosis of 24 Turkish Cypriot patients with ESRD was investigated. Using the ES method, we aimed to (1) characterize ESRD-associated variants in Turkish Cypriots using ES and (2) assess overlap with diabetes/FMF to clarify etiology.

## Materials and methods

A total of 24 patients with ESRD participated in the study. Patient information, sex, age, laboratory findings, and clinical diagnosis were collected.

For ES analyses, 4 ml peripheral venous blood was collected from each patient in an EDTA-containing collection tube. Genomic DNAs were isolated using a commercial kit (DNeasy Blood & Tissue Kit; Hilden, Germany). ES was performed after the capture-based method using SureSelect Human All Exon Target Enrichment System (Agilent Technologies, Santa Clara, CA). Next, paired-end sequencing was performed using the Genome Analyzer IIx (Illumina Inc., San Diego, CA). Reads were aligned to the human genome assembly HG19 using the NovoAlign alignment tool (Novocraft Technologies, Petaling Jaya, Malaysia). Variant calling and annotations were obtained using the Samtools/mpileup utility [24] and Annovar tools [25], respectively. Finally, variants were evaluated using Franklin Genoox [26] (https://franklin.genoox.com/) according to the American College of Medical Genetics and Genomics (ACMG) criteria [27] and ClinVar databases [28] (https://www.ncbi.nlm.nih.gov/clinvar).

Observed variants were prioritized by two different approaches using VarAFT software [29]. First, rare variants that could explain the genetic basis of the phenotype were identified by filtering for those with minor allele frequencies (MAF) below 0.01% in population databases such as gnomAD v3.1 and the 1000 Genomes Project, using a disease-associated gene list from the Human Phenotype Ontology (HPO) (Appendix 1) [30]. Second, all heterozygous and homozygous common variants in genes linked to renal anomalies were analyzed across all patients to explore potential associations specific to Turkish Cypriot cases.

## Results

In this study, the participants were aged 42 years or older, with a mean age of 63 years (Table 1). The number of male patients was higher (71%). By clinical evaluations, symptoms and laboratory results of creatinine level, glomerular filtration rate, blood urea nitrogen level, and proteinuria, hematuria and cyst availability, the patients were clinically diagnosed as glomerulonephritis, chronic renal disease (CRD), ESRD, congenital anomalies of the kidneys and urinary tracts (CAKUT) or tubulointerstitial disease. In addition to those phenotypes, 10 patients (41.7%) had secondary diseases, among which diabetes mellitus (DM) was prevalent (8/24; 33.3% amongst all patients and 8/10; 80% in secondary diseases).

**Table 1:** Patients with demographic information and clinical diagnosis with laboratory findings. BUN: blood urea nitrogen; eGFR: estimated glomerular filtration rate; nr: not recorded; GN: glomerulonephritis; DM: diabetes mellitus; CRD: chronic renal disease; CAKUT: congenital anomalies of the kidneys and urinary tracts; ESRD: end-stage renal disease; TID: tubulointerstitial disease; FSGS: focal segmental glomerulosclerosis; MCDK: multi-cystic dysplastic kidney; CRF: chronic renal failure; ADTKD: autosomal dominant tubulointerstitial kidney disease

Patient	Sex	Age at Diagnosis/ Therapy	Creatinine (mg/dL)	BUN (mg/dL)	EGFR (%)	Hematuria	Proetinuria	Cyst	Clinical Diagnosis
1	Male	nr	nr	nr	nr	nr	nr	nr	Renal hypodysplasia
2	Female	62	nr	nr	nr	nr	nr	nr	GN
3	Male	42	5.74	39	11	-	-	-	GN / DM
4	Female	46	4.80	52.5	12	-	-	-	CRD
5	Male	75	2.00	nr	nr	-	-	Yes	CRD
6	Male	55	nr	nr	76	Yes	-	-	CRD
7	Male	52	9.70	33	5	-	-	-	GN / CAKUT
8	Male	65	1.29	34	61	Yes	-	-	GN / ESRD
9	Male	nr	nr	nr	nr	Yes	-	Yes	CRD / TID / ESRD
10	Male	69	6.75	49	7	-	-	-	CRD
11	Female	50	0.70	14	112	Yes	-	-	GN / FSGS
12	Male	63	5.75	101	9	-	Yes	Yes	ESRD / DM / MCDK
13	Male	59	1.33	24	nr	Yes	-	-	CRD / DM
14	Male	73	7.10	56.2	7	-	-	-	GN / DM / CAKUT
15	Male	77	1.17	21	42	-	-	-	CRD / DM
16	Male	87	nr	nr	nr	nr	nr	nr	GN
17	Male	61	0.88	21	91	Yes	Yes	Yes	CRF/ DM / CAKUT
18	Male	63	4.58	29	13	-	-	-	GN
19	Female	65	0.66	24	101	-	-	-	CRD / DM
20	Female	69	1.30	21	41	-	-	-	ESRD
21	Male	65	3.41	37	18	-	-	-	GN / ADTKD
22	Female	60	0.64	14	98	Yes	-	Yes (multi)	GN / DM / MCDK
23	Female	72	0.87	20	73	-	-	-	CRD
24	Male	56	7.90	39	7	-	-	-	FSGS / GN / DM

According to the ES analyses, there were variants with unknown significance (VUS), likely pathogenic (LP) or pathogenic properties on diverse genes that were/could be associated with the related phenotypes (Table 2). In isolated cases (according to the primary clinical evaluation and verbal declaration of the patients; Patients 1, 2, 4-11, 16, 20, 21 and 23), had at least one VUS on various genes except for Patient 5 and 17 who may have structural variants that could not be detected in the current study. For the patients with renal failure and DM (Patients 3, 12-15, 17, 19, 22 and 24), two patients had at least one variant that was particularly linked to DM, one patient had variants on different genes which could separately be reasons of renal failure and DM whereas the remaining had variants on genes solely related to renal failure. In Patient 18, who was diagnosed with glomerulonephritis and retinoblastoma, an LP variant in the HNF1A gene associated with DM was detected. Thus, the genetic background of the renal failures for Patients 3, 18, 1,9, and 22 was debatable in terms of whether it was the DM-associated secondary phenotype or the primary phenotype. In three patients (12.5%), in addition to the variants on other genes, pathogenic or LP variants in the MEFV gene were identified.

**Table 2:** WES results of the patients with ESRD. The results were presented by candidate VUS/LP/pathogenic variants, known gene-related disease/syndrome, heterozygosity, pathogenicity (predicted or previously reported) and ACMG criteria for each patient. WES: whole-exome sequencing; ESRD: End-stage renal disease; VUS: variant of uncertain significance; LP: likely pathogenic; P: pathogenic; ACMG: American College of Medical Genetics and Genomics OMIM: Online Mendelian Inheritance in Man; Het: heterozygous; Hom: homozygous; AD: autosomal dominant; AR: autosomal recessive; ND: not detected; XLR: X-linked recessive inheritance; Mu: multimodal or multiple modes of inheritance (?) Inheritance pattern not definitively established.

Patient	Variant	Disease, OMIM Codes, and Inheritance Pattern	Heterozygosity (Read/Allele Freq)	Pathogenicity	ACMG Criteria
1	PAX2:NM_000278.5:c.227G>A:p.Gly76Asp	Focal segmental glomerulosclerosis 7 (616002), AD; papillorenal syndrome (120330), AD	Het (144/0.38)	LP	PM1, PM2, PP2, PP3, PM5
2	LAMA5:NM_005560.6:c.2871C>A: p.Cys957Ter	Bent bone dysplasia syndrome 2 (620076) (?), AR; nephrotic syndrome, type 26 (620049), AR	Hom (19/1.0)	LP	PVS1, PM2
3	ABCC8: NM_000352.6:c.4209_4211del:p.Ile1404del	Diabetes mellitus, noninsulin-dependent (125853), AD; hyperinsulinemic hypoglycemia, familial, 1 (256450), AD/AR	Het (87/0.38)	VUS	PM1, PM2, PM4
	MEFV: NM_000243.3:c.2230G>T:p.Ala744Ser	Familial Mediterranean fever, AD (134610); familial Mediterranean fever, AR (249100); neutrophilic dermatosis, acute febrile (608068), AD	Het (55/0.49)	P	PM2, PP5, VS
4	CEP290:NM_025114.4:c.5998A>G:p.Ile2000Val	Bardet-Biedl syndrome 14 (615991) (?), AR; Joubert syndrome 5 (610188), AR; Leber congenital amaurosis 10 (611755) (?); Meckel syndrome 4 (611134), AR; Senior-Loken syndrome 6 (610189), AR	Het (164/0.42)	VUS	PM2, BP4
	CEP290:NM_025114.4:c.963T>A:p.Asp321Glu		Het (42/0.48)		PM2
5	ND	-	-	-	-
6	ARHGAP24:NM_001025616.3:c.1496G>A:p.Arg499Gln	Glomerulosclerosis, focal segmental, 1 (603278), AD (?)	Het (159/0.47)	VUS	PM2
	INF2:NM_022489.4:c.1670T>G:p.Val557Gly	Charcot-Marie-Tooth disease, dominant intermediate E (614455), AD; glomerulosclerosis, focal segmental, 5 (613237), AD (?)	Het (29/0.28)	VUS	PM2, BP4
7	ANLN:NM_018685.5:c.886_887insTTC:p.Ser295_Pro296insLeu	Focal segmental glomerulosclerosis 8 (616032), AD	Het (130/0.44)	VUS	PM2, PM4
	CHD1L:NM_004284.6:c.1929del:p.Arg643SerfsTer16	CAKUT, AD (?)	Het (43/0.58)	Pathogen	PVS1, PM2, PS4
8	DSTYK:NM_015375.3:c.764A>G:p.Asp255Gly	CAKUT 1 (610805), AD; spastic paraplegia 23 (270750), AR	Het (190/0.51)	VUS	PM2
9	CD46:NM_172351.3:c.1045G>C:p.Val349Leu	Hemolytic uremic syndrome, atypical, susceptibility to 2 (612922), AD/AR	Het (89/0.48)	VUS	PM2, BP4
	FN1:NM_212482.4:c.5125A>G:p.Ser1709Gly	Glomerulopathy with fibronectin deposits 2 (601894), AD; spondylometaphyseal dysplasia, corner fracture type (184255), AD	Het (114/0.54)	VUS	PM2, BP4
	INF2:NM_022489.4:c.1952C>T:p.Ser651Phe	Charcot-Marie-Tooth disease, dominant intermediate E (614455), AD; focal segmental glomerulosclerosis 5 (613237), AD	Het (117/0.58)	VUS	PM2
	PKD1:NM_001009944.3c.12218C>T:p.Thr4073Ile	Polycystic kidney disease 1 (173900), AD	Het (73/0.40)	VUS	PM2, BP4
	PKD1:NM_001009944.3c.6048C>G: p.Asp2016Glu		Het (136/0.63)		
10	ANLN:NM_018685.5:c.886_887insTTC:p.Ser295_Pro296insLeu	Focal segmental glomerulosclerosis 8 (616032), AD	Het (50/0.60)	VUS	PM2, PM4
	COL4A6:NM_001287758.2:c.1968_1970dup:p.Glu657dup	X-linked Alport syndrome, XLR	Hom (49/1.0)	VUS	PM2, PM4
11	ANLN:NM_018685.5:c.886_887insTTC:p.Ser295_Pro296insLeu	Focal segmental glomerulosclerosis 8 (616032), AD	Het (72/0.53)	VUS	PM2, PM4
	PKD1:NM_001009944.3:c.8914G>A:p.Asp2972Asn	Polycystic kidney disease 1 (173900), AD	Het (23/0.39)	VUS	PM2
12	ANLN:NM_018685.5:c.886_887insTTC:p.Ser295_Pro296insLeu	Focal segmental glomerulosclerosis 8 (616032), AD	Het (68/0.44)	VUS	PM2, PM4
	APOL1:NM_003661.4:c.518C>T:p.Thr173Ile	Focal segmental glomerulosclerosis 4, susceptibility to 612551, AD	Het (199/0.54)	VUS	PM2
	COL4A6:NM_001287758.2:c.1968_1970dup:p.Glu657dup	X-linked Alport syndrome, XLR	Hom (47/1.0)	VUS	PM2, PM4
	MYH9:NM_002473.6:c.3677G>A:p.Arg1226Gln	Macrothrombocytopenia and granulocyte inclusions with or without nephritis or sensorineural hearing loss (155100), AD	Het (249/0.46)	VUS	PM2, PP2
13	ANLN:NM_018685.5:c.886_887insTTC:p.Ser295_Pro296insLeu	Focal segmental glomerulosclerosis 8 (616032), AD	Het (82/0.41)	VUS	PM2, PM4
	COL4A6:NM_001287758.2:c.1968_1970dup:p.Glu657dup	X-linked Alport Syndrome, XLR	Hom (52/1.0)	VUS	PM2, PM4
	NPR1:NM_000906.4:c.1768G>A:p.Val590Met	Nephropathy-hypertension (161900), AD	Het (237/0.44)	VUS	PM2
14	COL4A3:NM_000091.5:c.3419-2del	Alport syndrome 3A, (104200), AD; Alport syndrome 3B (620536) AR; Benign familial hematuria 2 (620320) (?)	Het (18/0.22)	LP	PVS1, PM2
	COL4A6:NM_001287758.2:c.1968_1970dup:p.Glu657dup	X-linked Alport syndrome, XLR	Hom (50/1.0)	VUS	PM2, PM4
	FGA:NM_021871.4:c.451C>T:p.His151Tyr	Afibrinogenemia, congenital (202400), AR; Familial visceral amyloidosis (105200), AD; Congenital dysfibrinogenemia (616004) (?); hypodysfibrinogenemia, congenital (616004) (?)	Het (74/0.50)	VUS	PM2
	MEFV:NM_000243.3:c.2177T>C:p.Val726Ala	Familial Mediterranean fever (134610), AD; familial Mediterranean fever (249100), AR; Neutrophilic dermatosis, acute febrile (608068), AD	Het (127/0.53)	LP	PM2, PP5
	PKD1:NM_001009944.3:c.6535G>A:p.Val2179Ile	Polycystic kidney disease 1 (173900), AD	Het (123/0.50)	VUS	PM2
15	SLC36A2:NM_181776.3:c.164+1G>A	Hyperglycinuria 138500, AD; Iminoglycinuria (242600), AR/DR	Het (83/0.49)	VUS	PM2
	UMOD:NM_003361.4:c.-102-1G>A	Tubulointerstitial kidney disease 1 (162000), AD	Het (81/0.42)	VUS	PVS1, PM2
16	ANLN:NM_018685.5:c.886_887insTTC:p.Ser295_Pro296insLeu	Focal segmental glomerulosclerosis 8 (616032), AD	Hom (55/0.96)	VUS	PM2, PM4
	ANLN:NM_018685.5:c.971C>G:p.Pro324Arg		Het (89/0.45)	VUS	PM2, BP4
	COL4A6:NM_001287758.2:c.1968_1970dup:p.Glu657dup	X-linked Alport syndrome, XLR	Hom (34/1.0)	VUS	PM2, PM4
17	ND	-	-	-	-
18	ANLN:NM_018685.5:c.886_887insTTC:p.Ser295_Pro296insLeu	Focal segmental glomerulosclerosis 8 (616032), AD	Het (77/0.40)	VUS	PM2, PM4
	COL4A3:NM_000091.5:c.3419-2del	Alport syndrome 3A (104200), AD; Alport syndrome 3B, autosomal recessive (620536), AR; Benign familial hematuria 2 (620320) (?)	Het (28/0.29)	LP	PVS1, PM2
	HNF1A:NM_000545.8:c.481G>A:p.Ala161Thr	Diabetes mellitus, insulin-dependent (222100), AR; diabetes mellitus, noninsulin-dependent 2 (125853), AD; diabetes mellitus, insulin-dependent, 20 (612520) (?); hepatic adenoma, somatic (142330) (?); MODY, type III (600496), AD; renal cell carcinoma (144700) (?)	Het (214/0.53)	LP	PM1, PM2, PP2, PP3
19	APOE:NM_000041.4:c.688G>A:p.Glu230Lys	Lipoprotein glomerulopathy (611771) (?)	Het (108/0.56)	VUS	PM2
	SLC36A2:NM_181776.3:c.3G>A:p.Met1?	Hyperglycinuria (138500), AD; iminoglycinuria (242600), AR/DR	Het (33/0.45)	VUS	PM2
	UCP3:NM_003356.4:c.490G>A:p.Asp164Asn	Obesity, severe, and type II diabetes (601665), AD/AR/Mu	Het (313/0.45)	VUS	PM2
20	CFI:NM_000204.5:c.1405G>A:p.Val469Ile	Hemolytic uremic syndrome, atypical, susceptibility to 3 (612923), AD; Macular degeneration, age-related, 13, susceptibility to 615439, AD; Complement factor I deficiency (610984), AR	Het (78/0.53)	VUS	PM2
	PKD1:NM_001009944.3c.6048C>G: p.Asp2016Glu	Polycystic kidney disease 1 (173900), AD	Het (129/0.48)	VUS	PM2, BP4
	SLC3A1:NM_000341.4:c.247C>T:p.Arg83Cys	Cystinuria (220100), AD/AR	Het (120/0.53)	VUS	PM1_P, PM2, PP2, PP3
21	ANLN:NM_018685.5:c.886_887insTTC:p.Ser295_Pro296insLeu	Focal segmental glomerulosclerosis 8 (616032), AD	Het (74/1.0)	VUS	PM2, PM4
	COL4A6:NM_001287758.2:c.1968_1970dup:p.Glu657dup	X-linked Alport syndrome, XLR	Het (39/1.0)	VUS	PM2, PM4
	MEFV:NM_000243.3:c.758_759insCGCC:p.Pro254Alafs*72	Familial Mediterranean fever, AD (134610); familial Mediterranean fever, AR (249100); neutrophilic dermatosis, acute febrile (608068), AD	Het (124/0.37)	LP	PVS1, PM2
	SLC4A1:NM_000342.4:c.287G>A:p.Arg96His	Distal renal tubular acidosis 1 (179800), AD	Het (250/0.49)	VUS	PM2, BP4
	SOX17:NM_022454.4:c.976_977insGCACCA:p.His326delinsArgThrAsn	Vesicoureteral reflux 3 (613674), AD	Het (48/0.67)	VUS	PM2, PM4
	UMOD:NM_003361.4:c.1131G>T:p.Trp377Cys	Tubulointerstitial kidney disease 1 (162000), AD	Het (146/0.51)	VUS	PM2, PP2, PP3
22	ANLN:NM_018685.5:c.886_887insTTC:p.Ser295_Pro296insLeu	Focal segmental glomerulosclerosis 8 (616032), AD	Het (72/0.35)	VUS	PM2, PM4
	CFHR5:NM_030787.4:c.415A>C:p.Ile139Leu	Nephropathy due to CFHR5 deficiency (614809), AD	Het (49/0.53)	VUS	PM2, BP4
	PKD1:NM_001009944.3:c.12236C>T:p.Ser4079Phe	Polycystic kidney disease 1 (173900), AD	Het (66/0.59)	VUS	PM2
	WFS1:NM_006005.3:c.2391_2392insACG:p.Asp797_Val798insThr	Diabetes mellitus, noninsulin-dependent, association with 125853, AD	Het (284/0.50)	VUS	PM2, PM4
23	COL4A1:NM_001845.6:c.4601C>T:p.Ala1534Val	Angiopathy, hereditary, with nephropathy, aneurysms, and muscle cramps (611773), AD	Het (123/0.47)	VUS	PM2, PP2, PP3
	COL4A6:NM_001287758.2:c.1968_1970dup:p.Glu657dup	X-linked Alport syndrome, XLR	Hom (82/1.0)	VUS	PM2, PM4
24	ANLN:NM_018685.5:c.886_887insTTC:p.Ser295_Pro296insLeu	Focal segmental glomerulosclerosis 8 (616032), AD	Het (31/0.45)	VUS	PM2, PM4
	COL4A6:NM_001287758.2:c.1968_1970dup:p.Glu657dup	X-linked Alport Syndrome, XLR	Hom (37/1.0)	VUS	PM2, PM4
	EHHADH:NM_001966.4:c.883C>T:p.Arg295Trp	Fanconi renotubular syndrome 3 (615605), AD (?)	Het (152/0.41)	VUS	PM2
	THBD:NM_000361.3:c.617C>A:p.Ala206Glu	Hemolytic uremic syndrome, atypical, susceptibility to 6 (612926), AD; thrombophilia 12 due to thrombomodulin defect (614486), AD	Het (114/0.56)	VUS	PM2

Amongst the variants that were detected in the patients, the frequencies of ANLN:NM_018685.5:c.886_887insTTC:p.Ser295_Pro296insLeu with one homozygous and nine heterozygous patterns and COL4A6:NM_001287758.2:c.1968_1970dup:p.Glu657dup with one heterozygous and seven homozygous patterns was relatively high. When the common variants were screened without any filtering strategy (Appendices 4-5), the number of common variants was extremely high. Although previously reported variants were not commonly detected in our patients, the single-nucleotide polymorphisms (SNPs) in the collagen coding genes were particularly noteworthy.

## Discussion

The current study aims to retrospectively investigate the ES results of 24 patients with end-stage renal failure in a Turkish Cypriot sample pool. For molecular and differential diagnosis of ESRD, ES could be a powerful tool as it covers the whole protein-coding regions of all genes [22]. Moreover, ES may be used to identify novel genes associated with renal anomalies. In congenital renal anomalies, the applicability of the ES approach has already been reported [31-33]. Moreover, this method has also been proposed for adult patients with renal anomalies [23,34,35]. Yet, so far, the relevance of the ES approach in Cypriot patients with ESRD has not been exhaustively documented. Nevertheless, through the ES using a different Turkish Cypriot cohort, a seemingly non-pathogenic variant was noted (COL4A4:p.G545A) which appeared to increase the susceptibility, and perhaps acted as a hypomorphic variant contributing to Alport spectrum nephropathy [36].

In our cohort, the number of patients with overlapping diagnoses of ESRD and DM was remarkable (8/24; 33.3% amongst all patients and 8/10; 80% in secondary diseases; Table 1). The risk of having ESRD in DM patients has been well-documented [37-39]. The co-existence of DM and ESRD makes it challenging to segregate the genetic basis of each disease [40]. Thus, targeted sequencing of renal genes may not be sufficient to explore the genetic background of the disease [41].

The relatively advanced ages of the patients necessitated careful analyses of the variants and genes. In Patients 4, 15, and 17, the genes CEP290 [42] and UMOD [43] have been linked to the early-onset renal anomalies in the presence of additional phenotypes. However, incomplete penetrance could perhaps account for the late-onset manifestation of the disease, which requires further molecular studies.

High prevalence of the mutations in the MEFV gene associated with FMF in the Turkish Cypriot Population has already been reported [44]. In three patients in the current cohort, pathogenic or LP variants in the MEFV gene were also detected. Importantly, ESRD has been reported in FMF patients with mutations in the MEFV gene [45,46], emphasizing the overlapping phenotypes and importance of the MEFV gene in Turkish Cypriots.

Notably, variants of the ANLN and COL4A6 genes were frequently detected in the patients of the current study (Table 2). Even though ANLN has been linked to the nephrotic syndrome [47], the variant in the ANLN gene (c.886_887insTTC:p.Ser295_Pro296insLeu) was not reported previously. Critically, the COL4A6 gene has been pointed out to be a candidate gene for Alport Syndrome by co-deletion in addition to the COL4A5 gene [48]. Nevertheless, two variants (ANLN:NM_018685.5:c.886_887insTTC:p.Ser295_Pro296insLeu and COL4A6:NM_001287758.2:c.1968_1970dup:p.Glu657dup) were found to co-segregate in six patients (25%), which would show a novel dual gene responsible for renal anomalies. Further molecular studies should be conducted for a comprehensive conclusion.

Finally, an association analysis was carried out to elucidate heterozygous and homozygous common variants in all patients (Appendices 2-3). Because of the close community structure of Cyprus, as previously reported [10], the number of common variants was dramatically high; therefore, we manually searched the variants that were reported to be associated with renal diseases in Cyprus [49-51]. Although previously reported variants were not commonly detected in our patients, particularly the SNPs rs1516446, rs536174, rs598819, rs2881142, rs4623610, and rs1266736 in the collagen-coding genes could be regarded in further investigations. Importantly, in a genome-wide association study conducted in a European cohort, a SNP (rs55703767) in the COL4A3 gene was linked to diabetic kidney disease [52], underlying the value of SNPs in the collagen-coding genes as a risk factor in renal diseases.

As a retrospective approach, the data of the patients were re-analyzed in the present study. Unfortunately, some of the demographic parameters could not be obtained. The relationship between the genes/variants and the patient phenotypes may require additional laboratory tests and/or clinical investigations for a conclusion. Nonetheless, contact with the patients was limited, and such tests/investigations could not be performed. The findings were previously and clinically reported after confirmation of the critical variants by Sanger Sequencing. Nevertheless, the VUSs that were listed in Table 2 were not confirmed.

Variant filtering was primarily performed according to the allele frequencies. However, Cypriots are a part of an isolated population. Therefore, filtering by allele frequencies may not be the ideal strategy [53]. An attempt was made to find common variants without any variant filtration. However, the number of common variants was extremely high, and it was not possible to assess the disease-related variants without filtering by allele frequencies. A population-based genome profiling of healthy participants may overcome this problem by filtering the variants according to this local database.

## Conclusions

In Cyprus, renal diseases with end-stage renal failure are common; however, the etiology of the disease is unremarkably unknown. When the complexity of the disease is taken into consideration, genetic analyses could be informative in terms of understanding the molecular background of the disease. Genetic studies on Cypriots so far showed that ancestral or mutation profiles were different from those in the Turkish or Greek mainland. Hence, island-based population studies are necessary. In this perspective, as a part of an ongoing study, we conducted a cohort analysis of Turkish Cypriots with ESRD. Accordingly, at least one VUS for nearly all patients, except for one, was detected in diverse genes related to renal failure. The number of VUSs was critically high, requiring molecular and/or cohort confirmation. Nonetheless, since the genetic background of the island population may be isolated, such studies should be conducted in an island-specific fashion using a large and comprehensive sequencing approach as in the current study. Moreover, a genome project on the island by healthy participants could be useful for illuminating the genetic background of such phenotypes.

## Appendices

Appendix 1

Table 3 shows the disease-associated gene list from Human Phenotype Ontology [30].

**Table 3:** Disease-associated gene list from Human Phenotype Ontology

AAGAB
ABCA12
ABCC6
ABCC8
ABCG5
ABCG8
ACE
ACP5
ACSL4
ACTB
ACTG1
ACTG2
ACTN4
ACVRL1
ADA
ADA2
ADAMTS13
ADAMTS3
ADAMTSL1
ADAT3
ADCY10
ADGRG2
ADNP
AFF3
AFF4
AGGF1
AGPAT2
AGT
AGTR1
AGXT
AHI1
AIP
AIRE
AKR1D1
AKT1
AKT3
ALDH18A1
ALDH4A1
ALDOA
ALDOB
ALG1
ALG5
ALG8
ALG9
ALKBH8
ALMS1
ALOX12B
ALOXE3
ALPL
ALX4
AMER1
AMMECR1
AMN
ANKFY1
ANKLE2
ANKRD17
ANKS6
ANLN
ANOS1
ANTXR1
AP1S3
AP2S1
APC
APC2
APOA1
APOB
APOE
APOL1
APPL1
APRT
AQP2
ARHGAP24
ARHGDIA
ARID1A
ARID1B
ARID2
ARL3
ARL6
ARL6IP6
ARMC5
ARNT2
ARPC4
ARSK
ARVCF
ARX
ASPM
ASPRV1
ASXL1
ASXL2
ATN1
ATP1A1
ATP5F1A
ATP5F1D
ATP5F1E
ATP5MD
ATP6V0A4
ATP6V1B1
ATP6V1B2
ATP6V1E1
ATP7A
ATP7B
ATPAF1
ATPAF2
ATRX
AURKA
AVIL
AVPR2
AXIN2
B2M
B3GALT6
B3GLCT
B4GAT1
B9D1
B9D2
BANK1
BAP1
BAX
BAZ1B
BBIP1
BBS1
BBS10
BBS12
BBS2
BBS4
BBS5
BBS7
BBS9
BCL7B
BCOR
BCS1L
BICC1
BICRA
BLK
BLM
BMP4
BMPER
BMPR1A
BNC2
BRAF
BRCA1
BRCA2
BRD4
BRF1
BRIP1
BSCL2
BSND
BTNL2
BUB1
BUB1B
BUB3
BUD23
C1QA
C1QBP
C1S
C2CD3
C3
C4A
C4B
CA2
CACNA1D
CACNA1S
CAD
CAMK2A
CAMKMT
CAPN15
CASK
CASP10
CASR
CASZ1
CAV1
CC2D2A
CCBE1
CCDC141
CCDC22
CCDC28B
CCN2
CCND1
CCND2
CCNQ
CCR1
CCR6
CD109
CD151
CD2AP
CD46
CD81
CD96
CDC42
CDC42BPB
CDC73
CDK4
CDK5RAP2
CDK6
CDKL5
CDKN1A
CDKN1B
CDKN1C
CDKN2A
CDKN2B
CDKN2C
CDON
CEACAM3
CEACAM6
CEL
CENPE
CENPF
CENPJ
CEP120
CEP135
CEP152
CEP164
CEP19
CEP290
CEP41
CEP55
CEP57
CEP63
CEP83
C8orf37
CFB
CFH
CFHR1
CFHR3
CFHR5
CFI
CFTR
CHD4
CHD7
CHEK2
CHKA
CHN1
CHRM3
CHRNA3
CHST14
CHUK
CILK1
CISD2
CIT
CLCA4
CLCN3
CLCN5
CLCN7
CLCNKA
CLCNKB
CLDN10
CLDN16
CLDN19
CLDN2
CLEC7A
CLIP2
CLPB
CNNM2
COA8
COG1
COG6
COG7
COL14A1
COL18A1
COL3A1
COL4A1
COL4A3
COL4A4
COL4A5
COL4A6
COL7A1
COLEC10
COLEC11
COMT
COPA
COPB2
COQ2
COQ6
COQ7
COQ8B
CORIN
COX14
CPLANE1
CPLX1
CPOX
CPT1A
CPT2
CR2
CRB2
CREBBP
CRIPTO
CRTAP
CSPP1
CTBP1
CTH
CTLA4
CTNNB1
CTNS
CTU2
CUBN
CWC27
CYB561
CYBC1
CYP24A1
CYP4F22
DAAM2
DACT1
DCC
DCDC2
DCHS1
DCLRE1C
DCTN4
DDB1
DDX59
DDX6
DEAF1
DGKE
DHCR24
DHCR7
DHDDS
DHODH
DHX16
DHX37
DICER1
DIS3L2
DISP1
DKC1
DLC1
DLK1
DLL1
DLL4
DLST
DMP1
DMRT3
DMXL2
DNA2
DNAJB11
DNAJC21
DNAJC30
DNASE1
DNASE1L3
DNASE2
DNMT3A
DPF2
DPH1
DPH2
DPYSL5
DSE
DSTYK
DUSP6
DVL1
DYNC2H1
DYNC2I1
DYNC2I2
DYNC2LI1
DYRK1A
DZIP1L
EBP
EDNRA
EDNRB
EFEMP2
EHHADH
EHMT1
EIF2AK3
EIF4H
ELN
ELP1
EMP2
EN1
ENG
ENPP1
EP300
EPAS1
EPG5
ERAP1
ERBB3
ERCC4
ERCC6
ERCC8
ESCO2
ETFA
ETFB
ETFDH
ETS1
EVC
EVC2
EXT2
EXTL3
EYA1
F10
F2
F5
F8
FAH
FAM149B1
FAM20A
FAM20C
FAN1
FANCA
FANCB
FANCC
FANCD2
FANCE
FANCF
FANCG
FANCI
FANCL
FANCM
FAS
FASLG
FAT4
FBLN5
FBN1
FBXL4
FBXW11
FCGR2A
FCGR2B
FCGR3B
FEZF1
FGA
FGF10
FGF13
FGF17
FGF20
FGF23
FGF8
FGFR1
FGFR2
FGFR3
FH
FIBP
FIG4
FIP1L1
FKBP6
FKRP
FKTN
FLCN
FLI1
FLII
FLNA
FLNB
FLRT3
FLT1
FN1
FOCAD
FOXC2
FOXE1
FOXF1
FOXH1
FOXI1
FOXP1
FOXP3
FOXRED1
FRAS1
FREM1
FREM2
FUT8
FUZ
FXYD2
G6PC1
G6PC3
GABRD
GALNT3
GANAB
GAPVD1
GAS1
GATA1
GATA3
GATA4
GATM
GBA1
GBE1
GCDH
GCK
GCLC
GCM2
GDF2
GDF3
GDF6
GEMIN4
GFRA1
GLA
GLI1
GLI2
GLI3
GLIS2
GLIS3
GLMN
GNA11
GNAO1
GNAS
GNB1
GNB2
GNPTAB
GON7
GP1BA
GP1BB
GP9
GPC3
GPC4
GPKOW
GPR35
GRB10
GREB1L
GRHPR
GRIA3
GRIN1
GRIP1
GRM7
GSN
GSTM3
GTF2I
GTF2IRD1
GTF2IRD2
H19
H4C3
H4C5
H4C9
HAAO
HABP2
HBB
HDAC4
HDAC8
HEATR3
HELLPAR
HESX1
HFE
HGD
HIC1
HIRA
HLA-B
HLA-DPA1
HLA-DPB1
HLA-DRB1
HMBS
HMGA2
HMOX1
HNF1A
HNF1B
HNF4A
HNRNPH1
HNRNPK
HNRNPU
HOGA1
HOXA13
HOXD13
HPRT1
HPS1
HPSE2
HRAS
HS2ST1
HS6ST1
HSD11B2
HSD17B4
HSPA9
HSPG2
HYLS1
HYMAI
IARS1
IDH1
IFIH1
IFNG
IFNGR1
IFT122
IFT140
IFT172
IFT27
IFT43
IFT56
IFT74
IFT80
IGF2
IGHG1
IKZF1
IL10
IL12A
IL12A-AS1
IL17F
IL17RA
IL17RC
IL17RD
IL23R
IL2RG
IL36RN
IL6
IL7R
INF2
INPP5E
INS
INSL3
INSR
INTS1
INTU
INVS
IPO8
IQCB1
IQSEC2
IRAK1
IRF2BP2
IRF5
ITGA2
ITGA2B
ITGA3
ITGA6
ITGA8
ITGAM
ITGB3
ITGB4
ITPR3
JAG1
JAK1
JAM3
JAZF1
JMJD1C
KANK2
KANSL1
KARS1
KAT5
KAT6A
KAT6B
KCNA1
KCNAB2
KCNE5
KCNH1
KCNJ1
KCNJ10
KCNJ11
KCNJ2
KCNJ5
KCNN4
KCNQ1
KCNQ1OT1
KCTD1
KDM6A
KEAP1
KIAA0319L
KIAA0586
KIAA0753
KIF14
KIF1B
KIF7
KIRREL1
KITLG
KL
KLF11
KLLN
KLRC4
KMT2A
KMT2C
KMT2D
KNL1
KNSTRN
KRAS
KRT17
KYNU
LAGE3
LAMA3
LAMA5
LAMB2
LAMB3
LAMC2
LARGE1
LARS2
LCAT
LDHA
LDLR
LDLRAP1
LEMD3
LETM1
LHX1
LIG1
LIG4
LIMK1
LIPN
LMAN1
LMBRD1
LMNA
LMNB1
LMNB2
LMOD1
LMX1B
LONP1
LPIN1
LPIN2
LRIG2
LRP2
LRP4
LRP5
LTBP1
LTBP4
LUZP1
LYZ
LZTFL1
MAD2L2
MAFB
MAGED2
MAGI2
MAP2K1
MAP2K2
MAP3K1
MAP3K7
MAPK1
MAPKBP1
MARS1
MASP1
MAX
MBTPS2
MCC
MCFD2
MCM5
MCM7
MCPH1
MCTP2
MDH2
MDM2
MECP2
MED11
MED12
MED25
MEFV
MEG3
MEN1
MEOX1
MET
METTL27
METTL5
MFSD2A
MGME1
MIA3
MID1
MIF
MINPP1
MITF
MKKS
MKS1
MLH1
MLH3
MLXIPL
MMACHC
MME
MMP1
MMP23B
MMUT
MNX1
MOCOS
MOCS1
MOCS2
MPDU1
MPI
MRPL3
MRPS22
MRPS34
MSH2
MSH3
MSH6
MST1
MT-ATP6
MT-ATP8
MT-CO1
MT-CO2
MT-CO3
MT-CYB
MT-ND1
MT-ND2
MT-ND3
MT-ND4
MT-ND5
MT-ND6
MT-TE
MT-TF
MT-TH
MT-TK
MT-TL1
MT-TN
MT-TQ
MT-TS1
MT-TS2
MT-TT
MT-TV
MT-TW
MTRR
MTX2
MUC1
MVK
MYCN
MYD88
MYH11
MYH9
MYL9
MYLK
MYMK
MYMX
MYO1E
MYO5B
MYOCD
MYOD1
MYRF
NAA10
NABP1
NADK2
NADSYN1
NARS2
NBN
NCAPD3
NCAPG2
NCF1
NDNF
NDUFA1
NDUFA11
NDUFA6
NDUFAF1
NDUFAF2
NDUFAF3
NDUFAF4
NDUFAF5
NDUFAF6
NDUFAF8
NDUFB10
NDUFB11
NDUFB3
NDUFB9
NDUFS1
NDUFS2
NDUFS3
NDUFS4
NDUFS6
NDUFS7
NDUFS8
NDUFV1
NDUFV2
NEK1
NEK8
NELFA
NEU1
NEUROD1
NEUROD2
NEXMIF
NF1
NFIA
NFS1
NHERF1
NIPAL4
NIPBL
NLRP3
NOD2
NODAL
NOS1AP
NOTCH2
NOTCH3
NPHP1
NPHP3
NPHP4
NPHS1
NPHS2
NPM1
NR0B1
NR5A1
NRAS
NRIP1
NSD1
NSD2
NSDHL
NSUN2
NTRK1
NUBPL
NUMA1
NUP107
NUP133
NUP160
NUP205
NUP37
NUP85
NUP93
NXN
OBSCN
OCLN
OCRL
OFD1
OGG1
OPLAH
OSGEP
OTUD5
OXGR1
P4HA2
PACS1
PAFAH1B1
PAH
PALB2
PAX1
PAX2
PAX4
PAX6
PAX7
PBRM1
PBX1
PC
PCK1
PCK2
PCSK9
PDCD1
PDCD6IP
PDE6D
PDGFRB
PDGFRL
PDHA1
PDPN
PDSS2
PDX1
PEX1
PEX10
PEX11B
PEX12
PEX13
PEX14
PEX16
PEX19
PEX2
PEX26
PEX3
PEX5
PEX6
PEX7
PFKM
PGAM2
PGAP2
PGAP3
PGK1
PGM1
PGM3
PHC1
PHEX
PHF21A
PHGDH
PHKA2
PHKB
PHKG2
PHYH
PI4KA
PIDD1
PIEZO2
PIGA
PIGG
PIGL
PIGN
PIGO
PIGP
PIGQ
PIGT
PIGV
PIGW
PIGY
PIK3C2A
PIK3CA
PIK3CD
PIK3R2
PKD1
PKD2
PKDCC
PKHD1
PLA2G2A
PLAGL1
PLCD1
PLCE1
PLD1
PLEC
PLG
PLVAP
PLXNA1
PML
PMM2
PNKP
PNPLA2
PNPLA6
POGZ
POLE
POLR3A
POLRMT
POMT1
POMT2
POR
PORCN
POT1
POU3F4
POU6F2
PPFIBP1
PPM1B
PPOX
PPP1R15B
PPP2R1A
PPP2R3C
PPP3CA
PQBP1
PRCC
PRDM16
PRDX1
PREPL
PRF1
PRIM1
PRKACA
PRKACB
PRKAG2
PRKAR1A
PRKCD
PRKCSH
PRKCZ
PRMT7
PRODH
PROK2
PROKR2
PRPS1
PRTN3
PSMC1
PTCH1
PTEN
PTH
PTH1R
PTPN11
PTPN12
PTPN22
PTPRJ
PTPRO
PUF60
PUS3
PXK
PYCR2
PYGL
PYGM
RAB18
RAB23
RAB3GAP1
RAB3GAP2
RAD21
RAD51
RAD51C
RAD54B
RAF1
RAG1
RAG2
RAI1
RARA
RARB
RASA1
RASGRP1
RBBP8
RBM10
RBM8A
RECQL4
REN
RERE
REST
RET
RFC2
RFWD3
RFX7
RIPK4
RMND1
RMRP
RNF139
RNU4ATAC
RNU7-1
ROBO1
ROBO2
ROR2
RORA
RPGRIP1
RPGRIP1L
RPL11
RPL15
RPL18
RPL26
RPL27
RPL31
RPL35
RPL35A
RPL5
RPL8
RPL9
RPS10
RPS15A
RPS17
RPS19
RPS20
RPS24
RPS26
RPS27
RPS28
RPS29
RPS7
RRAGD
RREB1
RRM2B
RSPO2
RTL1
RTTN
RYR1
SAA1
SALL1
SALL4
SARS1
SARS2
SASS6
SAT1
SBDS
SC5D
SCAPER
SCARB2
SCARF2
SCLT1
SCN1B
SCN2A
SCNN1A
SCNN1B
SCNN1G
SDCCAG8
SDHA
SDHAF2
SDHB
SDHC
SDHD
SDR9C7
SEC23B
SEC24C
SEC61A1
SEC63
SEMA3A
SEMA3E
SEMA4A
SEMA4D
SERPINA1
SERPINF2
SERPINH1
SETBP1
SETD2
SF3B2
SF3B4
SGPL1
SH2B1
SHANK3
SHH
SHOC2
SHPK
SI
SIK1
SIX1
SIX2
SIX3
SIX5
SKI
SKIV2
KIAA0372
SLC11A1
SLC12A1
SLC12A3
SLC17A5
SLC1A1
SLC1A2
SLC22A12
SLC25A11
SLC25A20
SLC25A22
SLC26A1
SLC26A4
SLC26A9
SLC29A3
SLC2A2
SLC2A9
SLC30A9
SLC32A1
SLC34A1
SLC34A2
SLC34A3
SLC35A2
SLC36A2
SLC37A4
SLC3A1
SLC41A1
SLC4A1
SLC4A2
SLC4A4
SLC5A1
SLC5A2
SLC6A14
SLC6A17
SLC7A7
SLC7A9
SLC9A3
SLX4
SMAD4
SMARCA4
SMARCAL1
SMARCB1
SMARCC2
SMARCD1
SMARCE1
SMC1A
SMC3
SMO
SMOC1
SMS
SNAI2
SNAP29
SNRPB
SNRPN
SOCS1
SON
SOS1
SOX10
SOX11
SOX17
SOX18
SOX4
SOX9
SPART
SPECC1L
SPEN
SPINK5
SPINT2
SPOP
SPP1
SPRED1
SPRY2
SPRY4
SPTBN1
SRC
SRCAP
SREBF1
SRP54
SRY
SSR4
STAG1
STAT1
STAT2
STAT3
STAT4
STAT5B
STIL
STIM1
STK11
STOX1
STRA6
STRADA
STS
STX11
STX1A
STX3
STXBP1
STXBP2
SUCLG1
SUFU
SULT2B1
SURF1
TACR3
TAF13
TALDO1
TAOK1
TAPT1
TASP1
TBC1D20
TBC1D24
TBC1D7
TBC1D8B
TBCK
TBL1XR1
TBL2
TBX1
TBX15
TBX18
TBX22
TBX3
TBXT
TCF4
TCN2
TCTN1
TCTN2
TCTN3
TELO2
TFAP2A
TFE3
TGFB1
TGIF1
TGM1
THBD
THOC6
TIMMDC1
TKT
TLR2
TLR4
TLR7
TMCO1
TMEM107
TMEM126B
TMEM127
TMEM138
TMEM165
TMEM216
TMEM218
TMEM231
TMEM237
TMEM260
TMEM270
TMEM67
TMEM70
TNFAIP3
TNFSF4
TNIP1
TNXB
TOGARAM1
TOM1
TOPORS
TOR1A
TP53
TP53RK
TP63
TPRKB
TRAF3IP1
TRAF3IP2
TRAPPC10
MAP11
TREX1
TRIM28
TRIM32
TRIM37
TRIM8
TRIP11
TRIP13
TRMT5
TRNT1
TRPC6
TRPV6
TRRAP
TSC1
TSC2
TSR2
TTC21B
TTC8
TTR
TULP3
TXNDC15
TXNL4A
TYR
UBA2
UBAC2
UBE2A
UBE2L3
UBE2T
UBE4B
UBR1
UFD1
UMOD
UMPS
UNC13D
UQCC2
USF3
USP18
USP8
USP9X
VAC14
VAMP7
VANGL1
VDR
VHL
VIPAS39
VPS33A
VPS33B
VPS35L
VPS37D
VPS45
WAC
WARS1
WAS
WASHC5
WBP11
WDPCP
WDR11
WDR19
WDR35
WDR4
WDR62
WDR73
WFS1
WIPF1
WLS
WNT3
WNT4
WNT5A
WNT7B
WNT9B
WRN
WT1
WWOX
XDH
XPNPEP3
XRCC2
XRCC4
XYLT1
XYLT2
YAP1
YRDC
YWHAE
YY1
YY1AP1
ZAP70
ZBTB16
ZBTB18
ZEB2
ZFP57
ZFPM2
ZIC2
ZIC3
ZMIZ1
ZMPSTE24
ZMYM2
ZNF148
ZNF423
ZNF592
ZNF687
ZNF699
ZNFX1
ZPR1

Appendix 2

Table 4 shows the common variants within disease-related genes.

**Table 4:** Common variants within disease-related genes. SNV: single-nucleotide variant

Chr	Start	End	Ref	Alt	Func.refgene	Gene.refgene	ExonicFunc.refgene	AAChange.refgene	avsnp150	CLINSIG	
1	1275330	1275330	A	G	intronic	DVL1	.	.	rs11585063	.	ID=S2009_raw:GT=het:DP=6:FQ=0.33:SQ=-1;ID=S2503_raw:GT=het:DP=15:FQ=0.53:SQ=-1;ID=S2507_raw:GT=het:DP=15:FQ=0.4:SQ=-1;ID=S2668_raw:GT=het:DP=5:FQ=0.6:SQ=-1;ID=S2012_raw:GT=uncertain-het:DP=7:FQ=0.29:SQ=-1;ID=S2010_raw:GT=het:DP=10:FQ=0.7:SQ=-1;ID=S2662_raw:GT=het:DP=29:FQ=0.41:SQ=-1;ID=S2663_raw:GT=het:DP=12:FQ=0.42:SQ=-1;ID=S2011_raw:GT=het:DP=15:FQ=0.4:SQ=-1;ID=S2504_raw:GT=het:DP=19:FQ=0.58:SQ=-1;ID=S2008_raw:GT=het:DP=14:FQ=0.57:SQ=-1;ID=S2509_raw:GT=uncertain-het:DP=20:FQ=0.2:SQ=-1;ID=S2508_raw:GT=het:DP=17:FQ=0.35:SQ=-1;ID=S2666_raw:GT=het:DP=6:FQ=0.83:SQ=-1;ID=S2669_raw:GT=het:DP=6:FQ=0.5:SQ=-1;ID=S2166_raw:GT=uncertain-het:DP=15:FQ=0.27:SQ=-1;ID=S1775_raw:GT=het:DP=7:FQ=0.57:SQ=-1;ID=S2501_raw:GT=het:DP=15:FQ=0.53:SQ=-1;ID=S2500_raw:GT=uncertain-het:DP=22:FQ=0.27:SQ=-1;ID=S2502_raw:GT=uncertain-het:DP=18:FQ=0.22:SQ=-1;ID=S2505_raw:GT=uncertain-het:DP=17:FQ=0.29:SQ=-1;ID=S2176_raw:GT=het:DP=18:FQ=0.33:SQ=-1;ID=S2506_raw:GT=het:DP=21:FQ=0.38:SQ=-1;ID=S2667_raw:GT=het:DP=12:FQ=0.33:SQ=-1;
7	1528945	1528945	C	A	intronic	INTS1	.	.	rs78793025	.	ID=S2009_raw:GT=het:DP=39:FQ=0.49:SQ=-1;ID=S2503_raw:GT=uncertain-het:DP=44:FQ=0.2:SQ=-1;ID=S2507_raw:GT=het:DP=71:FQ=0.35:SQ=-1;ID=S2668_raw:GT=uncertain-het:DP=59:FQ=0.2:SQ=-1;ID=S2012_raw:GT=het:DP=39:FQ=0.41:SQ=-1;ID=S2010_raw:GT=het:DP=32:FQ=0.59:SQ=-1;ID=S2662_raw:GT=het:DP=90:FQ=0.31:SQ=-1;ID=S2663_raw:GT=uncertain-het:DP=43:FQ=0.28:SQ=-1;ID=S2011_raw:GT=het:DP=42:FQ=0.38:SQ=-1;ID=S2504_raw:GT=uncertain-het:DP=64:FQ=0.23:SQ=-1;ID=S2008_raw:GT=het:DP=53:FQ=0.36:SQ=-1;ID=S2509_raw:GT=het:DP=50:FQ=0.48:SQ=-1;ID=S2508_raw:GT=uncertain-het:DP=73:FQ=0.22:SQ=-1;ID=S2666_raw:GT=uncertain-het:DP=78:FQ=0.22:SQ=-1;ID=S2669_raw:GT=uncertain-het:DP=60:FQ=0.22:SQ=-1;ID=S2166_raw:GT=uncertain-het:DP=48:FQ=0.27:SQ=-1;ID=S1775_raw:GT=uncertain-het:DP=66:FQ=0.23:SQ=-1;ID=S2501_raw:GT=uncertain-het:DP=64:FQ=0.22:SQ=-1;ID=S2500_raw:GT=het:DP=47:FQ=0.3:SQ=-1;ID=S2502_raw:GT=uncertain-het:DP=64:FQ=0.25:SQ=-1;ID=S2505_raw:GT=het:DP=44:FQ=0.3:SQ=-1;ID=S2176_raw:GT=uncertain-het:DP=69:FQ=0.17:SQ=-1;ID=S2506_raw:GT=het:DP=41:FQ=0.41:SQ=-1;ID=S2667_raw:GT=uncertain-het:DP=59:FQ=0.27:SQ=-1;
7	1528946	1528946	A	G	intronic	INTS1	.	.	rs80163046	.	ID=S2009_raw:GT=het:DP=40:FQ=0.48:SQ=-1;ID=S2503_raw:GT=uncertain-het:DP=44:FQ=0.23:SQ=-1;ID=S2507_raw:GT=het:DP=71:FQ=0.35:SQ=-1;ID=S2668_raw:GT=uncertain-het:DP=60:FQ=0.2:SQ=-1;ID=S2012_raw:GT=het:DP=39:FQ=0.41:SQ=-1;ID=S2010_raw:GT=het:DP=32:FQ=0.63:SQ=-1;ID=S2662_raw:GT=het:DP=90:FQ=0.33:SQ=-1;ID=S2663_raw:GT=het:DP=45:FQ=0.31:SQ=-1;ID=S2011_raw:GT=het:DP=40:FQ=0.38:SQ=-1;ID=S2504_raw:GT=uncertain-het:DP=63:FQ=0.24:SQ=-1;ID=S2008_raw:GT=het:DP=52:FQ=0.42:SQ=-1;ID=S2509_raw:GT=het:DP=50:FQ=0.48:SQ=-1;ID=S2508_raw:GT=uncertain-het:DP=72:FQ=0.24:SQ=-1;ID=S2666_raw:GT=uncertain-het:DP=75:FQ=0.23:SQ=-1;ID=S2669_raw:GT=uncertain-het:DP=62:FQ=0.23:SQ=-1;ID=S2166_raw:GT=uncertain-het:DP=46:FQ=0.28:SQ=-1;ID=S1775_raw:GT=uncertain-het:DP=63:FQ=0.24:SQ=-1;ID=S2501_raw:GT=uncertain-het:DP=65:FQ=0.23:SQ=-1;ID=S2500_raw:GT=het:DP=47:FQ=0.3:SQ=-1;ID=S2502_raw:GT=uncertain-het:DP=62:FQ=0.26:SQ=-1;ID=S2505_raw:GT=het:DP=44:FQ=0.32:SQ=-1;ID=S2176_raw:GT=uncertain-het:DP=69:FQ=0.17:SQ=-1;ID=S2506_raw:GT=het:DP=39:FQ=0.44:SQ=-1;ID=S2667_raw:GT=het:DP=60:FQ=0.3:SQ=-1;
7	1528947	1528947	T	C	intronic	INTS1	.	.	rs79178284	.	ID=S2009_raw:GT=het:DP=38:FQ=0.5:SQ=-1;ID=S2503_raw:GT=uncertain-het:DP=40:FQ=0.25:SQ=-1;ID=S2507_raw:GT=het:DP=69:FQ=0.38:SQ=-1;ID=S2668_raw:GT=uncertain-het:DP=60:FQ=0.2:SQ=-1;ID=S2012_raw:GT=het:DP=38:FQ=0.42:SQ=-1;ID=S2010_raw:GT=het:DP=32:FQ=0.63:SQ=-1;ID=S2662_raw:GT=het:DP=88:FQ=0.34:SQ=-1;ID=S2663_raw:GT=het:DP=45:FQ=0.33:SQ=-1;ID=S2011_raw:GT=het:DP=37:FQ=0.38:SQ=-1;ID=S2504_raw:GT=uncertain-het:DP=64:FQ=0.23:SQ=-1;ID=S2008_raw:GT=het:DP=52:FQ=0.44:SQ=-1;ID=S2509_raw:GT=het:DP=49:FQ=0.49:SQ=-1;ID=S2508_raw:GT=uncertain-het:DP=71:FQ=0.24:SQ=-1;ID=S2666_raw:GT=uncertain-het:DP=77:FQ=0.23:SQ=-1;ID=S2669_raw:GT=uncertain-het:DP=60:FQ=0.22:SQ=-1;ID=S2166_raw:GT=uncertain-het:DP=45:FQ=0.29:SQ=-1;ID=S1775_raw:GT=uncertain-het:DP=64:FQ=0.25:SQ=-1;ID=S2501_raw:GT=uncertain-het:DP=63:FQ=0.24:SQ=-1;ID=S2500_raw:GT=het:DP=48:FQ=0.31:SQ=-1;ID=S2502_raw:GT=uncertain-het:DP=60:FQ=0.27:SQ=-1;ID=S2505_raw:GT=het:DP=43:FQ=0.3:SQ=-1;ID=S2176_raw:GT=uncertain-het:DP=68:FQ=0.18:SQ=-1;ID=S2506_raw:GT=het:DP=38:FQ=0.45:SQ=-1;ID=S2667_raw:GT=het:DP=59:FQ=0.31:SQ=-1;
7	1,52E+08	1,52E+08	T	A	exonic	KMT2C	nonsynonymous SNV	KMT2C:NM_170606:exon7:c.946A>T:p.T316S	rs10454320	.	ID=S2009_raw:GT=het:DP=35:FQ=0.69:SQ=-1;ID=S2503_raw:GT=het:DP=460:FQ=0.8:SQ=-1;ID=S2507_raw:GT=het:DP=422:FQ=0.77:SQ=-1;ID=S2668_raw:GT=het:DP=480:FQ=0.77:SQ=-1;ID=S2012_raw:GT=het:DP=26:FQ=0.77:SQ=-1;ID=S2010_raw:GT=het:DP=30:FQ=0.7:SQ=-1;ID=S2662_raw:GT=het:DP=35:FQ=0.49:SQ=-1;ID=S2663_raw:GT=het:DP=413:FQ=0.82:SQ=-1;ID=S2011_raw:GT=het:DP=49:FQ=0.71:SQ=-1;ID=S2504_raw:GT=het:DP=539:FQ=0.76:SQ=-1;ID=S2008_raw:GT=het:DP=43:FQ=0.67:SQ=-1;ID=S2509_raw:GT=het:DP=496:FQ=0.81:SQ=-1;ID=S2508_raw:GT=het:DP=535:FQ=0.79:SQ=-1;ID=S2666_raw:GT=het:DP=415:FQ=0.79:SQ=-1;ID=S2669_raw:GT=het:DP=304:FQ=0.76:SQ=-1;ID=S2166_raw:GT=het:DP=1040:FQ=0.79:SQ=-1;ID=S1775_raw:GT=het:DP=907:FQ=0.8:SQ=-1;ID=S2501_raw:GT=het:DP=528:FQ=0.79:SQ=-1;ID=S2500_raw:GT=het:DP=528:FQ=0.81:SQ=-1;ID=S2502_raw:GT=het:DP=505:FQ=0.78:SQ=-1;ID=S2505_raw:GT=het:DP=460:FQ=0.79:SQ=-1;ID=S2176_raw:GT=het:DP=1161:FQ=0.78:SQ=-1;ID=S2506_raw:GT=het:DP=405:FQ=0.8:SQ=-1;ID=S2667_raw:GT=het:DP=398:FQ=0.77:SQ=-1;
7	1,52E+08	1,52E+08	G	A	exonic	KMT2C	nonsynonymous SNV	KMT2C:NM_170606:exon7:c.871C>T:p.L291F	rs56850341	.	ID=S2009_raw:GT=het:DP=45:FQ=0.38:SQ=-1;ID=S2503_raw:GT=het:DP=216:FQ=0.64:SQ=-1;ID=S2507_raw:GT=het:DP=172:FQ=0.58:SQ=-1;ID=S2668_raw:GT=het:DP=175:FQ=0.5:SQ=-1;ID=S2012_raw:GT=het:DP=48:FQ=0.31:SQ=-1;ID=S2010_raw:GT=het:DP=47:FQ=0.34:SQ=-1;ID=S2662_raw:GT=uncertain-het:DP=19:FQ=0.26:SQ=-1;ID=S2663_raw:GT=het:DP=176:FQ=0.62:SQ=-1;ID=S2011_raw:GT=het:DP=75:FQ=0.56:SQ=-1;ID=S2504_raw:GT=het:DP=233:FQ=0.57:SQ=-1;ID=S2008_raw:GT=het:DP=55:FQ=0.45:SQ=-1;ID=S2509_raw:GT=het:DP=211:FQ=0.57:SQ=-1;ID=S2508_raw:GT=het:DP=211:FQ=0.54:SQ=-1;ID=S2666_raw:GT=het:DP=121:FQ=0.57:SQ=-1;ID=S2669_raw:GT=het:DP=142:FQ=0.57:SQ=-1;ID=S2166_raw:GT=het:DP=478:FQ=0.65:SQ=-1;ID=S1775_raw:GT=het:DP=338:FQ=0.61:SQ=-1;ID=S2501_raw:GT=het:DP=208:FQ=0.61:SQ=-1;ID=S2500_raw:GT=het:DP=226:FQ=0.6:SQ=-1;ID=S2502_raw:GT=het:DP=181:FQ=0.6:SQ=-1;ID=S2505_raw:GT=het:DP=202:FQ=0.62:SQ=-1;ID=S2176_raw:GT=het:DP=541:FQ=0.63:SQ=-1;ID=S2506_raw:GT=het:DP=198:FQ=0.67:SQ=-1;ID=S2667_raw:GT=het:DP=186:FQ=0.62:SQ=-1;
1	1,21E+08	1,21E+08	A	G	UTR5	NOTCH2	.	NM_024408:c.-245T>C;NM_001200001:c.-245T>C	rs2746700	.	ID=S2009_raw:GT=het:DP=15:FQ=0.47:SQ=-1;ID=S2503_raw:GT=het:DP=93:FQ=0.52:SQ=-1;ID=S2507_raw:GT=het:DP=110:FQ=0.55:SQ=-1;ID=S2668_raw:GT=uncertain-het:DP=96:FQ=0.25:SQ=-1;ID=S2012_raw:GT=het:DP=10:FQ=0.4:SQ=-1;ID=S2010_raw:GT=het:DP=20:FQ=0.55:SQ=-1;ID=S2662_raw:GT=uncertain-het:DP=238:FQ=0.29:SQ=-1;ID=S2663_raw:GT=het:DP=140:FQ=0.44:SQ=-1;ID=S2011_raw:GT=het:DP=29:FQ=0.55:SQ=-1;ID=S2504_raw:GT=het:DP=169:FQ=0.51:SQ=-1;ID=S2008_raw:GT=het:DP=24:FQ=0.67:SQ=-1;ID=S2509_raw:GT=het:DP=123:FQ=0.37:SQ=-1;ID=S2508_raw:GT=het:DP=145:FQ=0.52:SQ=-1;ID=S2666_raw:GT=het:DP=54:FQ=0.44:SQ=-1;ID=S2669_raw:GT=het:DP=156:FQ=0.54:SQ=-1;ID=S2166_raw:GT=het:DP=25:FQ=0.44:SQ=-1;ID=S1775_raw:GT=het:DP=12:FQ=0.42:SQ=-1;ID=S2501_raw:GT=het:DP=131:FQ=0.48:SQ=-1;ID=S2500_raw:GT=het:DP=134:FQ=0.44:SQ=-1;ID=S2502_raw:GT=het:DP=124:FQ=0.55:SQ=-1;ID=S2505_raw:GT=het:DP=133:FQ=0.48:SQ=-1;ID=S2176_raw:GT=het:DP=25:FQ=0.6:SQ=-1;ID=S2506_raw:GT=het:DP=141:FQ=0.57:SQ=-1;ID=S2667_raw:GT=het:DP=127:FQ=0.49:SQ=-1;
X	1,55E+08	1,55E+08	G	A	intergenic	VAMP7;IL9R	.	dist=42118;dist=11695	rs4013617	.	ID=S2009_raw:GT=het:DP=24:FQ=0.54:SQ=-1;ID=S2503_raw:GT=het:DP=35:FQ=0.4:SQ=-1;ID=S2507_raw:GT=het:DP=40:FQ=0.58:SQ=-1;ID=S2668_raw:GT=het:DP=46:FQ=0.46:SQ=-1;ID=S2012_raw:GT=het:DP=17:FQ=0.65:SQ=-1;ID=S2010_raw:GT=het:DP=26:FQ=0.35:SQ=-1;ID=S2662_raw:GT=het:DP=13:FQ=0.69:SQ=-1;ID=S2663_raw:GT=het:DP=40:FQ=0.5:SQ=-1;ID=S2011_raw:GT=het:DP=22:FQ=0.55:SQ=-1;ID=S2504_raw:GT=het:DP=43:FQ=0.4:SQ=-1;ID=S2008_raw:GT=uncertain-het:DP=15:FQ=0.27:SQ=-1;ID=S2509_raw:GT=het:DP=57:FQ=0.4:SQ=-1;ID=S2508_raw:GT=het:DP=58:FQ=0.53:SQ=-1;ID=S2666_raw:GT=het:DP=43:FQ=0.49:SQ=-1;ID=S2669_raw:GT=het:DP=35:FQ=0.46:SQ=-1;ID=S2166_raw:GT=het:DP=24:FQ=0.42:SQ=-1;ID=S1775_raw:GT=het:DP=24:FQ=0.63:SQ=-1;ID=S2501_raw:GT=het:DP=54:FQ=0.5:SQ=-1;ID=S2500_raw:GT=het:DP=59:FQ=0.56:SQ=-1;ID=S2502_raw:GT=het:DP=49:FQ=0.71:SQ=-1;ID=S2505_raw:GT=het:DP=54:FQ=0.37:SQ=-1;ID=S2176_raw:GT=het:DP=42:FQ=0.43:SQ=-1;ID=S2506_raw:GT=het:DP=35:FQ=0.6:SQ=-1;ID=S2667_raw:GT=het:DP=29:FQ=0.45:SQ=-1;
3	75986663	75986663	C	A	intergenic	ZNF717;ROBO2	.	dist=151929;dist=1102631	rs12171318	Benign	ID=S2009_raw:GT=het:DP=8:FQ=0.63:SQ=-1;ID=S2503_raw:GT=het:DP=98:FQ=0.71:SQ=-1;ID=S2507_raw:GT=het:DP=85:FQ=0.72:SQ=-1;ID=S2668_raw:GT=het:DP=127:FQ=0.58:SQ=-1;ID=S2012_raw:GT=het:DP=13:FQ=0.46:SQ=-1;ID=S2010_raw:GT=het:DP=13:FQ=0.54:SQ=-1;ID=S2662_raw:GT=het:DP=18:FQ=0.5:SQ=-1;ID=S2663_raw:GT=het:DP=86:FQ=0.76:SQ=-1;ID=S2011_raw:GT=het:DP=16:FQ=0.63:SQ=-1;ID=S2504_raw:GT=het:DP=86:FQ=0.72:SQ=-1;ID=S2008_raw:GT=het:DP=9:FQ=0.78:SQ=-1;ID=S2509_raw:GT=het:DP=96:FQ=0.69:SQ=-1;ID=S2508_raw:GT=het:DP=73:FQ=0.73:SQ=-1;ID=S2666_raw:GT=het:DP=147:FQ=0.73:SQ=-1;ID=S2669_raw:GT=het:DP=64:FQ=0.7:SQ=-1;ID=S2166_raw:GT=het:DP=51:FQ=0.59:SQ=-1;ID=S1775_raw:GT=het:DP=43:FQ=0.63:SQ=-1;ID=S2501_raw:GT=het:DP=103:FQ=0.68:SQ=-1;ID=S2500_raw:GT=het:DP=129:FQ=0.71:SQ=-1;ID=S2502_raw:GT=het:DP=146:FQ=0.73:SQ=-1;ID=S2505_raw:GT=het:DP=88:FQ=0.6:SQ=-1;ID=S2176_raw:GT=het:DP=72:FQ=0.58:SQ=-1;ID=S2506_raw:GT=het:DP=77:FQ=0.69:SQ=-1;ID=S2667_raw:GT=het:DP=78:FQ=0.73:SQ=-1;
3	75986686	75986686	A	G	intergenic	ZNF717;ROBO2	.	dist=151952;dist=1102608	rs62269817	.	ID=S2009_raw:GT=het:DP=17:FQ=0.53:SQ=-1;ID=S2503_raw:GT=het:DP=142:FQ=0.72:SQ=-1;ID=S2507_raw:GT=het:DP=127:FQ=0.73:SQ=-1;ID=S2668_raw:GT=het:DP=227:FQ=0.64:SQ=-1;ID=S2012_raw:GT=het:DP=16:FQ=0.5:SQ=-1;ID=S2010_raw:GT=het:DP=18:FQ=0.61:SQ=-1;ID=S2662_raw:GT=het:DP=22:FQ=0.59:SQ=-1;ID=S2663_raw:GT=het:DP=148:FQ=0.72:SQ=-1;ID=S2011_raw:GT=het:DP=18:FQ=0.67:SQ=-1;ID=S2504_raw:GT=het:DP=121:FQ=0.68:SQ=-1;ID=S2008_raw:GT=het:DP=16:FQ=0.75:SQ=-1;ID=S2509_raw:GT=het:DP=152:FQ=0.67:SQ=-1;ID=S2508_raw:GT=het:DP=117:FQ=0.66:SQ=-1;ID=S2666_raw:GT=het:DP=209:FQ=0.71:SQ=-1;ID=S2669_raw:GT=het:DP=100:FQ=0.66:SQ=-1;ID=S2166_raw:GT=het:DP=73:FQ=0.62:SQ=-1;ID=S1775_raw:GT=het:DP=63:FQ=0.62:SQ=-1;ID=S2501_raw:GT=het:DP=158:FQ=0.68:SQ=-1;ID=S2500_raw:GT=het:DP=187:FQ=0.7:SQ=-1;ID=S2502_raw:GT=het:DP=208:FQ=0.76:SQ=-1;ID=S2505_raw:GT=het:DP=123:FQ=0.58:SQ=-1;ID=S2176_raw:GT=het:DP=104:FQ=0.6:SQ=-1;ID=S2506_raw:GT=het:DP=115:FQ=0.67:SQ=-1;ID=S2667_raw:GT=het:DP=121:FQ=0.74:SQ=-1;
3	75986719	75986719	G	C	intergenic	ZNF717;ROBO2	.	dist=151985;dist=1102575	rs62269818	.	ID=S2009_raw:GT=het:DP=32:FQ=0.59:SQ=-1;ID=S2503_raw:GT=het:DP=198:FQ=0.69:SQ=-1;ID=S2507_raw:GT=het:DP=176:FQ=0.68:SQ=-1;ID=S2668_raw:GT=het:DP=336:FQ=0.56:SQ=-1;ID=S2012_raw:GT=het:DP=31:FQ=0.65:SQ=-1;ID=S2010_raw:GT=het:DP=37:FQ=0.57:SQ=-1;ID=S2662_raw:GT=het:DP=41:FQ=0.54:SQ=-1;ID=S2663_raw:GT=het:DP=214:FQ=0.7:SQ=-1;ID=S2011_raw:GT=het:DP=36:FQ=0.67:SQ=-1;ID=S2504_raw:GT=het:DP=199:FQ=0.65:SQ=-1;ID=S2008_raw:GT=het:DP=35:FQ=0.66:SQ=-1;ID=S2509_raw:GT=het:DP=205:FQ=0.61:SQ=-1;ID=S2508_raw:GT=het:DP=201:FQ=0.62:SQ=-1;ID=S2666_raw:GT=het:DP=268:FQ=0.68:SQ=-1;ID=S2669_raw:GT=het:DP=147:FQ=0.59:SQ=-1;ID=S2166_raw:GT=het:DP=128:FQ=0.6:SQ=-1;ID=S1775_raw:GT=het:DP=98:FQ=0.61:SQ=-1;ID=S2501_raw:GT=het:DP=227:FQ=0.68:SQ=-1;ID=S2500_raw:GT=het:DP=263:FQ=0.7:SQ=-1;ID=S2502_raw:GT=het:DP=271:FQ=0.75:SQ=-1;ID=S2505_raw:GT=het:DP=185:FQ=0.54:SQ=-1;ID=S2176_raw:GT=het:DP=172:FQ=0.62:SQ=-1;ID=S2506_raw:GT=het:DP=158:FQ=0.64:SQ=-1;ID=S2667_raw:GT=het:DP=160:FQ=0.74:SQ=-1;
3	75986794	75986794	G	A	intergenic	ZNF717;ROBO2	.	dist=152060;dist=1102500	rs80235520	.	ID=S2009_raw:GT=het:DP=40:FQ=0.45:SQ=-1;ID=S2503_raw:GT=het:DP=218:FQ=0.54:SQ=-1;ID=S2507_raw:GT=het:DP=202:FQ=0.48:SQ=-1;ID=S2668_raw:GT=het:DP=357:FQ=0.45:SQ=-1;ID=S2012_raw:GT=het:DP=39:FQ=0.54:SQ=-1;ID=S2010_raw:GT=het:DP=64:FQ=0.48:SQ=-1;ID=S2662_raw:GT=het:DP=47:FQ=0.47:SQ=-1;ID=S2663_raw:GT=het:DP=217:FQ=0.57:SQ=-1;ID=S2011_raw:GT=het:DP=78:FQ=0.54:SQ=-1;ID=S2504_raw:GT=het:DP=216:FQ=0.45:SQ=-1;ID=S2008_raw:GT=het:DP=55:FQ=0.47:SQ=-1;ID=S2509_raw:GT=het:DP=211:FQ=0.47:SQ=-1;ID=S2508_raw:GT=het:DP=227:FQ=0.42:SQ=-1;ID=S2666_raw:GT=het:DP=309:FQ=0.46:SQ=-1;ID=S2669_raw:GT=het:DP=168:FQ=0.39:SQ=-1;ID=S2166_raw:GT=het:DP=173:FQ=0.51:SQ=-1;ID=S1775_raw:GT=het:DP=186:FQ=0.41:SQ=-1;ID=S2501_raw:GT=het:DP=249:FQ=0.51:SQ=-1;ID=S2500_raw:GT=het:DP=265:FQ=0.57:SQ=-1;ID=S2502_raw:GT=het:DP=240:FQ=0.6:SQ=-1;ID=S2505_raw:GT=het:DP=217:FQ=0.38:SQ=-1;ID=S2176_raw:GT=het:DP=234:FQ=0.49:SQ=-1;ID=S2506_raw:GT=het:DP=181:FQ=0.45:SQ=-1;ID=S2667_raw:GT=het:DP=162:FQ=0.54:SQ=-1;
3	75986813	75986813	T	A	intergenic	ZNF717;ROBO2	.	dist=152079;dist=1102481	rs77101234	.	ID=S2009_raw:GT=het:DP=33:FQ=0.36:SQ=-1;ID=S2503_raw:GT=het:DP=181:FQ=0.49:SQ=-1;ID=S2507_raw:GT=het:DP=176:FQ=0.43:SQ=-1;ID=S2668_raw:GT=het:DP=287:FQ=0.38:SQ=-1;ID=S2012_raw:GT=het:DP=36:FQ=0.47:SQ=-1;ID=S2010_raw:GT=het:DP=51:FQ=0.41:SQ=-1;ID=S2662_raw:GT=het:DP=36:FQ=0.44:SQ=-1;ID=S2663_raw:GT=het:DP=174:FQ=0.53:SQ=-1;ID=S2011_raw:GT=het:DP=74:FQ=0.46:SQ=-1;ID=S2504_raw:GT=het:DP=186:FQ=0.39:SQ=-1;ID=S2008_raw:GT=het:DP=45:FQ=0.33:SQ=-1;ID=S2509_raw:GT=het:DP=173:FQ=0.38:SQ=-1;ID=S2508_raw:GT=het:DP=197:FQ=0.35:SQ=-1;ID=S2666_raw:GT=het:DP=253:FQ=0.38:SQ=-1;ID=S2669_raw:GT=het:DP=148:FQ=0.33:SQ=-1;ID=S2166_raw:GT=het:DP=162:FQ=0.46:SQ=-1;ID=S1775_raw:GT=het:DP=173:FQ=0.33:SQ=-1;ID=S2501_raw:GT=het:DP=206:FQ=0.43:SQ=-1;ID=S2500_raw:GT=het:DP=228:FQ=0.49:SQ=-1;ID=S2502_raw:GT=het:DP=196:FQ=0.48:SQ=-1;ID=S2505_raw:GT=het:DP=198:FQ=0.32:SQ=-1;ID=S2176_raw:GT=het:DP=205:FQ=0.43:SQ=-1;ID=S2506_raw:GT=het:DP=155:FQ=0.34:SQ=-1;ID=S2667_raw:GT=het:DP=132:FQ=0.48:SQ=-1;
3	75986833	75986833	C	T	intergenic	ZNF717;ROBO2	.	dist=152099;dist=1102461	rs73123284	.	ID=S2009_raw:GT=uncertain-het:DP=29:FQ=0.24:SQ=-1;ID=S2503_raw:GT=het:DP=143:FQ=0.37:SQ=-1;ID=S2507_raw:GT=het:DP=138:FQ=0.32:SQ=-1;ID=S2668_raw:GT=uncertain-het:DP=199:FQ=0.25:SQ=-1;ID=S2012_raw:GT=het:DP=26:FQ=0.35:SQ=-1;ID=S2010_raw:GT=het:DP=43:FQ=0.33:SQ=-1;ID=S2662_raw:GT=het:DP=33:FQ=0.39:SQ=-1;ID=S2663_raw:GT=het:DP=117:FQ=0.42:SQ=-1;ID=S2011_raw:GT=het:DP=57:FQ=0.33:SQ=-1;ID=S2504_raw:GT=het:DP=155:FQ=0.31:SQ=-1;ID=S2008_raw:GT=uncertain-het:DP=34:FQ=0.24:SQ=-1;ID=S2509_raw:GT=uncertain-het:DP=127:FQ=0.24:SQ=-1;ID=S2508_raw:GT=uncertain-het:DP=152:FQ=0.22:SQ=-1;ID=S2666_raw:GT=het:DP=195:FQ=0.31:SQ=-1;ID=S2669_raw:GT=uncertain-het:DP=110:FQ=0.25:SQ=-1;ID=S2166_raw:GT=het:DP=129:FQ=0.4:SQ=-1;ID=S1775_raw:GT=uncertain-het:DP=129:FQ=0.23:SQ=-1;ID=S2501_raw:GT=het:DP=157:FQ=0.31:SQ=-1;ID=S2500_raw:GT=het:DP=169:FQ=0.36:SQ=-1;ID=S2502_raw:GT=het:DP=155:FQ=0.36:SQ=-1;ID=S2505_raw:GT=uncertain-het:DP=166:FQ=0.25:SQ=-1;ID=S2176_raw:GT=het:DP=158:FQ=0.32:SQ=-1;ID=S2506_raw:GT=uncertain-het:DP=131:FQ=0.26:SQ=-1;ID=S2667_raw:GT=het:DP=105:FQ=0.39:SQ=-1;

Appendix 5

Table 5 shows the common variants across all the genes. 

**Table 5:** Common variants across all genes.

Chr	Start	End	Ref	Alt	Func.refgene	Gene.refgene	ExonicFunc.refgene	AAChange.refgene	avsnp150	CLINSIG	
2	2,16E+08	2,16E+08	T	C	intronic	ABCA12	.	.	rs7608150	.	ID=S2009_raw:GT=hom:DP=9:FQ=1:SQ=-1;ID=S2503_raw:GT=hom:DP=21:FQ=1:SQ=-1;ID=S2507_raw:GT=hom:DP=25:FQ=1:SQ=-1;ID=S2668_raw:GT=hom:DP=36:FQ=1:SQ=-1;ID=S2012_raw:GT=hom:DP=5:FQ=1:SQ=-1;ID=S2010_raw:GT=hom:DP=7:FQ=1:SQ=-1;ID=S2662_raw:GT=hom:DP=6:FQ=1:SQ=-1;ID=S2663_raw:GT=hom:DP=15:FQ=1:SQ=-1;ID=S2011_raw:GT=hom:DP=12:FQ=1:SQ=-1;ID=S2504_raw:GT=hom:DP=28:FQ=1:SQ=-1;ID=S2008_raw:GT=hom:DP=14:FQ=1:SQ=-1;ID=S2509_raw:GT=hom:DP=19:FQ=1:SQ=-1;ID=S2508_raw:GT=hom:DP=41:FQ=1:SQ=-1;ID=S2666_raw:GT=hom:DP=46:FQ=1:SQ=-1;ID=S2669_raw:GT=hom:DP=17:FQ=1:SQ=-1;ID=S2166_raw:GT=hom:DP=7:FQ=1:SQ=-1;ID=S1775_raw:GT=hom:DP=3:FQ=1:SQ=-1;ID=S2501_raw:GT=hom:DP=23:FQ=1:SQ=-1;ID=S2500_raw:GT=hom:DP=23:FQ=1:SQ=-1;ID=S2502_raw:GT=hom:DP=33:FQ=1:SQ=-1;ID=S2505_raw:GT=hom:DP=22:FQ=1:SQ=-1;ID=S2176_raw:GT=hom:DP=14:FQ=1:SQ=-1;ID=S2506_raw:GT=hom:DP=19:FQ=1:SQ=-1;ID=S2667_raw:GT=hom:DP=25:FQ=1:SQ=-1;
2	2,16E+08	2,16E+08	C	G	intronic	ABCA12	.	.	rs1326748	.	ID=S2009_raw:GT=hom:DP=28:FQ=1:SQ=-1;ID=S2503_raw:GT=hom:DP=17:FQ=1:SQ=-1;ID=S2507_raw:GT=hom:DP=16:FQ=1:SQ=-1;ID=S2668_raw:GT=hom:DP=11:FQ=1:SQ=-1;ID=S2012_raw:GT=hom:DP=22:FQ=1:SQ=-1;ID=S2010_raw:GT=hom:DP=23:FQ=1:SQ=-1;ID=S2662_raw:GT=hom:DP=4:FQ=1:SQ=-1;ID=S2663_raw:GT=hom:DP=12:FQ=1:SQ=-1;ID=S2011_raw:GT=hom:DP=42:FQ=1:SQ=-1;ID=S2504_raw:GT=hom:DP=18:FQ=1:SQ=-1;ID=S2008_raw:GT=hom:DP=24:FQ=1:SQ=-1;ID=S2509_raw:GT=hom:DP=14:FQ=1:SQ=-1;ID=S2508_raw:GT=hom:DP=19:FQ=1:SQ=-1;ID=S2666_raw:GT=hom:DP=22:FQ=1:SQ=-1;ID=S2669_raw:GT=hom:DP=11:FQ=1:SQ=-1;ID=S2166_raw:GT=hom:DP=54:FQ=1:SQ=-1;ID=S1775_raw:GT=hom:DP=33:FQ=1:SQ=-1;ID=S2501_raw:GT=hom:DP=27:FQ=1:SQ=-1;ID=S2500_raw:GT=hom:DP=17:FQ=1:SQ=-1;ID=S2502_raw:GT=hom:DP=21:FQ=1:SQ=-1;ID=S2505_raw:GT=hom:DP=19:FQ=1:SQ=-1;ID=S2176_raw:GT=hom:DP=36:FQ=1:SQ=-1;ID=S2506_raw:GT=hom:DP=19:FQ=1:SQ=-1;ID=S2667_raw:GT=hom:DP=23:FQ=1:SQ=-1;
2	2,16E+08	2,16E+08	G	A	intronic	ABCA12	.	.	rs7594353	.	ID=S2009_raw:GT=hom:DP=12:FQ=1:SQ=-1;ID=S2503_raw:GT=hom:DP=57:FQ=1:SQ=-1;ID=S2507_raw:GT=hom:DP=53:FQ=1:SQ=-1;ID=S2668_raw:GT=hom:DP=71:FQ=1:SQ=-1;ID=S2012_raw:GT=hom:DP=2:FQ=1:SQ=-1;ID=S2010_raw:GT=hom:DP=12:FQ=1:SQ=-1;ID=S2662_raw:GT=hom:DP=9:FQ=1:SQ=-1;ID=S2663_raw:GT=hom:DP=77:FQ=1:SQ=-1;ID=S2011_raw:GT=hom:DP=17:FQ=1:SQ=-1;ID=S2504_raw:GT=hom:DP=75:FQ=1:SQ=-1;ID=S2008_raw:GT=hom:DP=8:FQ=1:SQ=-1;ID=S2509_raw:GT=hom:DP=61:FQ=1:SQ=-1;ID=S2508_raw:GT=hom:DP=59:FQ=1:SQ=-1;ID=S2666_raw:GT=hom:DP=53:FQ=1:SQ=-1;ID=S2669_raw:GT=hom:DP=44:FQ=1:SQ=-1;ID=S2166_raw:GT=hom:DP=55:FQ=1:SQ=-1;ID=S1775_raw:GT=hom:DP=29:FQ=1:SQ=-1;ID=S2501_raw:GT=hom:DP=46:FQ=1:SQ=-1;ID=S2500_raw:GT=hom:DP=51:FQ=1:SQ=-1;ID=S2502_raw:GT=hom:DP=68:FQ=1:SQ=-1;ID=S2505_raw:GT=hom:DP=52:FQ=1:SQ=-1;ID=S2176_raw:GT=hom:DP=75:FQ=1:SQ=-1;ID=S2506_raw:GT=hom:DP=41:FQ=1:SQ=-1;ID=S2667_raw:GT=hom:DP=60:FQ=1:SQ=-1;
2	2,16E+08	2,16E+08	C	T	intronic	ABCA12	.	.	rs10192100	.	ID=S2009_raw:GT=hom:DP=14:FQ=1:SQ=-1;ID=S2503_raw:GT=hom:DP=59:FQ=1:SQ=-1;ID=S2507_raw:GT=hom:DP=54:FQ=1:SQ=-1;ID=S2668_raw:GT=hom:DP=68:FQ=1:SQ=-1;ID=S2012_raw:GT=hom:DP=1:FQ=1:SQ=-1;ID=S2010_raw:GT=hom:DP=15:FQ=1:SQ=-1;ID=S2662_raw:GT=hom:DP=11:FQ=1:SQ=-1;ID=S2663_raw:GT=hom:DP=73:FQ=1:SQ=-1;ID=S2011_raw:GT=hom:DP=19:FQ=1:SQ=-1;ID=S2504_raw:GT=hom:DP=79:FQ=1:SQ=-1;ID=S2008_raw:GT=hom:DP=8:FQ=1:SQ=-1;ID=S2509_raw:GT=hom:DP=65:FQ=1:SQ=-1;ID=S2508_raw:GT=hom:DP=55:FQ=1:SQ=-1;ID=S2666_raw:GT=hom:DP=61:FQ=1:SQ=-1;ID=S2669_raw:GT=hom:DP=43:FQ=1:SQ=-1;ID=S2166_raw:GT=hom:DP=60:FQ=1:SQ=-1;ID=S1775_raw:GT=hom:DP=34:FQ=1:SQ=-1;ID=S2501_raw:GT=hom:DP=46:FQ=1:SQ=-1;ID=S2500_raw:GT=hom:DP=57:FQ=1:SQ=-1;ID=S2502_raw:GT=hom:DP=70:FQ=1:SQ=-1;ID=S2505_raw:GT=hom:DP=57:FQ=1:SQ=-1;ID=S2176_raw:GT=hom:DP=78:FQ=1:SQ=-1;ID=S2506_raw:GT=hom:DP=40:FQ=1:SQ=-1;ID=S2667_raw:GT=hom:DP=63:FQ=1:SQ=-1;
2	2,16E+08	2,16E+08	G	A	intronic	ABCA12	.	.	rs6435868	.	ID=S2009_raw:GT=hom:DP=3:FQ=1:SQ=-1;ID=S2503_raw:GT=hom:DP=5:FQ=1:SQ=-1;ID=S2507_raw:GT=hom:DP=6:FQ=1:SQ=-1;ID=S2668_raw:GT=hom:DP=6:FQ=1:SQ=-1;ID=S2012_raw:GT=hom:DP=3:FQ=1:SQ=-1;ID=S2010_raw:GT=hom:DP=4:FQ=1:SQ=-1;ID=S2662_raw:GT=hom:DP=2:FQ=1:SQ=-1;ID=S2663_raw:GT=hom:DP=1:FQ=1:SQ=-1;ID=S2011_raw:GT=hom:DP=5:FQ=1:SQ=-1;ID=S2504_raw:GT=hom:DP=2:FQ=1:SQ=-1;ID=S2008_raw:GT=hom:DP=3:FQ=1:SQ=-1;ID=S2509_raw:GT=hom:DP=4:FQ=1:SQ=-1;ID=S2508_raw:GT=hom:DP=5:FQ=1:SQ=-1;ID=S2666_raw:GT=hom:DP=4:FQ=1:SQ=-1;ID=S2669_raw:GT=hom:DP=4:FQ=1:SQ=-1;ID=S2166_raw:GT=hom:DP=18:FQ=1:SQ=-1;ID=S1775_raw:GT=hom:DP=10:FQ=1:SQ=-1;ID=S2501_raw:GT=hom:DP=6:FQ=1:SQ=-1;ID=S2500_raw:GT=hom:DP=8:FQ=1:SQ=-1;ID=S2502_raw:GT=hom:DP=6:FQ=1:SQ=-1;ID=S2505_raw:GT=hom:DP=6:FQ=1:SQ=-1;ID=S2176_raw:GT=hom:DP=12:FQ=1:SQ=-1;ID=S2506_raw:GT=hom:DP=3:FQ=1:SQ=-1;ID=S2667_raw:GT=hom:DP=4:FQ=1:SQ=-1;
2	2,16E+08	2,16E+08	A	T	intronic	ABCA12	.	.	rs2948978	.	ID=S2009_raw:GT=hom:DP=15:FQ=1:SQ=-1;ID=S2503_raw:GT=hom:DP=6:FQ=1:SQ=-1;ID=S2507_raw:GT=hom:DP=1:FQ=1:SQ=-1;ID=S2668_raw:GT=hom:DP=11:FQ=1:SQ=-1;ID=S2012_raw:GT=hom:DP=21:FQ=1:SQ=-1;ID=S2010_raw:GT=hom:DP=21:FQ=1:SQ=-1;ID=S2662_raw:GT=hom:DP=2:FQ=1:SQ=-1;ID=S2663_raw:GT=hom:DP=2:FQ=1:SQ=-1;ID=S2011_raw:GT=hom:DP=28:FQ=1:SQ=-1;ID=S2504_raw:GT=hom:DP=3:FQ=1:SQ=-1;ID=S2008_raw:GT=hom:DP=19:FQ=1:SQ=-1;ID=S2509_raw:GT=hom:DP=9:FQ=1:SQ=-1;ID=S2508_raw:GT=hom:DP=10:FQ=1:SQ=-1;ID=S2666_raw:GT=hom:DP=3:FQ=1:SQ=-1;ID=S2669_raw:GT=hom:DP=5:FQ=1:SQ=-1;ID=S2166_raw:GT=hom:DP=2:FQ=1:SQ=-1;ID=S1775_raw:GT=hom:DP=10:FQ=1:SQ=-1;ID=S2501_raw:GT=hom:DP=5:FQ=1:SQ=-1;ID=S2500_raw:GT=hom:DP=8:FQ=1:SQ=-1;ID=S2502_raw:GT=hom:DP=3:FQ=1:SQ=-1;ID=S2505_raw:GT=hom:DP=7:FQ=1:SQ=-1;ID=S2176_raw:GT=hom:DP=4:FQ=1:SQ=-1;ID=S2506_raw:GT=hom:DP=4:FQ=1:SQ=-1;ID=S2667_raw:GT=hom:DP=10:FQ=1:SQ=-1;
16	16251435	16251435	G	A	intronic	ABCC6	.	.	rs3896244	.	ID=S2009_raw:GT=hom:DP=13:FQ=1:SQ=-1;ID=S2503_raw:GT=hom:DP=58:FQ=1:SQ=-1;ID=S2507_raw:GT=hom:DP=49:FQ=1:SQ=-1;ID=S2668_raw:GT=hom:DP=52:FQ=1:SQ=-1;ID=S2012_raw:GT=hom:DP=11:FQ=1:SQ=-1;ID=S2010_raw:GT=hom:DP=16:FQ=1:SQ=-1;ID=S2662_raw:GT=hom:DP=82:FQ=1:SQ=-1;ID=S2663_raw:GT=hom:DP=55:FQ=1:SQ=-1;ID=S2011_raw:GT=hom:DP=15:FQ=1:SQ=-1;ID=S2504_raw:GT=hom:DP=37:FQ=1:SQ=-1;ID=S2008_raw:GT=hom:DP=25:FQ=1:SQ=-1;ID=S2509_raw:GT=hom:DP=58:FQ=1:SQ=-1;ID=S2508_raw:GT=hom:DP=62:FQ=1:SQ=-1;ID=S2666_raw:GT=hom:DP=90:FQ=1:SQ=-1;ID=S2669_raw:GT=hom:DP=57:FQ=1:SQ=-1;ID=S2166_raw:GT=hom:DP=30:FQ=1:SQ=-1;ID=S1775_raw:GT=hom:DP=27:FQ=1:SQ=-1;ID=S2501_raw:GT=hom:DP=57:FQ=1:SQ=-1;ID=S2500_raw:GT=hom:DP=49:FQ=1:SQ=-1;ID=S2502_raw:GT=hom:DP=63:FQ=1:SQ=-1;ID=S2505_raw:GT=hom:DP=44:FQ=1:SQ=-1;ID=S2176_raw:GT=hom:DP=44:FQ=1:SQ=-1;ID=S2506_raw:GT=hom:DP=49:FQ=1:SQ=-1;ID=S2667_raw:GT=hom:DP=67:FQ=1:SQ=-1;
16	16263837	16263837	T	C	intronic	ABCC6	.	.	rs7201980	.	ID=S2009_raw:GT=hom:DP=2:FQ=1:SQ=-1;ID=S2503_raw:GT=hom:DP=16:FQ=1:SQ=-1;ID=S2507_raw:GT=hom:DP=24:FQ=1:SQ=-1;ID=S2668_raw:GT=hom:DP=38:FQ=1:SQ=-1;ID=S2012_raw:GT=hom:DP=3:FQ=1:SQ=-1;ID=S2010_raw:GT=hom:DP=4:FQ=1:SQ=-1;ID=S2662_raw:GT=hom:DP=57:FQ=1:SQ=-1;ID=S2663_raw:GT=hom:DP=28:FQ=1:SQ=-1;ID=S2011_raw:GT=hom:DP=4:FQ=1:SQ=-1;ID=S2504_raw:GT=hom:DP=18:FQ=1:SQ=-1;ID=S2008_raw:GT=hom:DP=3:FQ=1:SQ=-1;ID=S2509_raw:GT=hom:DP=22:FQ=1:SQ=-1;ID=S2508_raw:GT=hom:DP=29:FQ=1:SQ=-1;ID=S2666_raw:GT=hom:DP=37:FQ=1:SQ=-1;ID=S2669_raw:GT=hom:DP=49:FQ=1:SQ=-1;ID=S2166_raw:GT=hom:DP=10:FQ=1:SQ=-1;ID=S1775_raw:GT=hom:DP=4:FQ=1:SQ=-1;ID=S2501_raw:GT=hom:DP=27:FQ=1:SQ=-1;ID=S2500_raw:GT=hom:DP=24:FQ=1:SQ=-1;ID=S2502_raw:GT=hom:DP=39:FQ=1:SQ=-1;ID=S2505_raw:GT=hom:DP=29:FQ=1:SQ=-1;ID=S2176_raw:GT=hom:DP=8:FQ=1:SQ=-1;ID=S2506_raw:GT=hom:DP=30:FQ=1:SQ=-1;ID=S2667_raw:GT=hom:DP=19:FQ=1:SQ=-1;
16	16263864	16263864	A	G	intronic	ABCC6	.	.	rs6498608	.	ID=S2009_raw:GT=hom:DP=1:FQ=1:SQ=-1;ID=S2503_raw:GT=hom:DP=11:FQ=1:SQ=-1;ID=S2507_raw:GT=hom:DP=18:FQ=1:SQ=-1;ID=S2668_raw:GT=hom:DP=38:FQ=1:SQ=-1;ID=S2012_raw:GT=hom:DP=1:FQ=1:SQ=-1;ID=S2010_raw:GT=hom:DP=3:FQ=1:SQ=-1;ID=S2662_raw:GT=hom:DP=40:FQ=1:SQ=-1;ID=S2663_raw:GT=hom:DP=22:FQ=1:SQ=-1;ID=S2011_raw:GT=hom:DP=1:FQ=1:SQ=-1;ID=S2504_raw:GT=hom:DP=13:FQ=1:SQ=-1;ID=S2008_raw:GT=hom:DP=4:FQ=1:SQ=-1;ID=S2509_raw:GT=hom:DP=19:FQ=1:SQ=-1;ID=S2508_raw:GT=hom:DP=23:FQ=1:SQ=-1;ID=S2666_raw:GT=hom:DP=26:FQ=1:SQ=-1;ID=S2669_raw:GT=hom:DP=34:FQ=1:SQ=-1;ID=S2166_raw:GT=hom:DP=7:FQ=1:SQ=-1;ID=S1775_raw:GT=hom:DP=1:FQ=1:SQ=-1;ID=S2501_raw:GT=hom:DP=21:FQ=1:SQ=-1;ID=S2500_raw:GT=hom:DP=22:FQ=1:SQ=-1;ID=S2502_raw:GT=hom:DP=32:FQ=1:SQ=-1;ID=S2505_raw:GT=hom:DP=30:FQ=1:SQ=-1;ID=S2176_raw:GT=hom:DP=9:FQ=1:SQ=-1;ID=S2506_raw:GT=hom:DP=25:FQ=1:SQ=-1;ID=S2667_raw:GT=hom:DP=12:FQ=1:SQ=-1;
16	16267079	16267079	A	G	intronic	ABCC6	.	.	rs11866320	.	ID=S2009_raw:GT=hom:DP=2:FQ=1:SQ=-1;ID=S2503_raw:GT=hom:DP=21:FQ=1:SQ=-1;ID=S2507_raw:GT=hom:DP=16:FQ=1:SQ=-1;ID=S2668_raw:GT=hom:DP=24:FQ=1:SQ=-1;ID=S2012_raw:GT=hom:DP=6:FQ=1:SQ=-1;ID=S2010_raw:GT=hom:DP=11:FQ=1:SQ=-1;ID=S2662_raw:GT=hom:DP=18:FQ=1:SQ=-1;ID=S2663_raw:GT=hom:DP=11:FQ=1:SQ=-1;ID=S2011_raw:GT=hom:DP=7:FQ=1:SQ=-1;ID=S2504_raw:GT=hom:DP=15:FQ=1:SQ=-1;ID=S2008_raw:GT=hom:DP=4:FQ=1:SQ=-1;ID=S2509_raw:GT=hom:DP=16:FQ=1:SQ=-1;ID=S2508_raw:GT=hom:DP=15:FQ=1:SQ=-1;ID=S2666_raw:GT=hom:DP=26:FQ=1:SQ=-1;ID=S2669_raw:GT=hom:DP=8:FQ=1:SQ=-1;ID=S2166_raw:GT=hom:DP=27:FQ=1:SQ=-1;ID=S1775_raw:GT=hom:DP=13:FQ=1:SQ=-1;ID=S2501_raw:GT=hom:DP=20:FQ=1:SQ=-1;ID=S2500_raw:GT=hom:DP=16:FQ=1:SQ=-1;ID=S2502_raw:GT=hom:DP=23:FQ=1:SQ=-1;ID=S2505_raw:GT=hom:DP=24:FQ=1:SQ=-1;ID=S2176_raw:GT=hom:DP=25:FQ=1:SQ=-1;ID=S2506_raw:GT=hom:DP=25:FQ=1:SQ=-1;ID=S2667_raw:GT=hom:DP=20:FQ=1:SQ=-1;
16	16269640	16269640	G	-	intronic	ABCC6	.	.	.	.	ID=S2009_raw:GT=hom:DP=11:FQ=1:SQ=-1;ID=S2503_raw:GT=hom:DP=34:FQ=1:SQ=-1;ID=S2507_raw:GT=hom:DP=36:FQ=1:SQ=-1;ID=S2668_raw:GT=hom:DP=44:FQ=1:SQ=-1;ID=S2012_raw:GT=hom:DP=8:FQ=1:SQ=-1;ID=S2010_raw:GT=hom:DP=15:FQ=1:SQ=-1;ID=S2662_raw:GT=hom:DP=42:FQ=1:SQ=-1;ID=S2663_raw:GT=hom:DP=39:FQ=1:SQ=-1;ID=S2011_raw:GT=hom:DP=12:FQ=1:SQ=-1;ID=S2504_raw:GT=hom:DP=31:FQ=1:SQ=-1;ID=S2008_raw:GT=hom:DP=11:FQ=1:SQ=-1;ID=S2509_raw:GT=hom:DP=56:FQ=1:SQ=-1;ID=S2508_raw:GT=hom:DP=34:FQ=1:SQ=-1;ID=S2666_raw:GT=hom:DP=45:FQ=1:SQ=-1;ID=S2669_raw:GT=hom:DP=39:FQ=1:SQ=-1;ID=S2166_raw:GT=hom:DP=14:FQ=1:SQ=-1;ID=S1775_raw:GT=hom:DP=7:FQ=1:SQ=-1;ID=S2501_raw:GT=hom:DP=34:FQ=1:SQ=-1;ID=S2500_raw:GT=hom:DP=46:FQ=1:SQ=-1;ID=S2502_raw:GT=hom:DP=39:FQ=1:SQ=-1;ID=S2505_raw:GT=hom:DP=38:FQ=1:SQ=-1;ID=S2176_raw:GT=hom:DP=10:FQ=1:SQ=-1;ID=S2506_raw:GT=hom:DP=30:FQ=1:SQ=-1;ID=S2667_raw:GT=hom:DP=39:FQ=1:SQ=-1;
16	16271357	16271357	T	C	exonic	ABCC6	nonsynonymous SNV	ABCC6:NM_001171:exon19:c.2542A>G:p.M848V,ABCC6:NM_001351800:exon19:c.2200A>G:p.M734V	rs6416668	.	ID=S2009_raw:GT=hom:DP=83:FQ=1:SQ=-1;ID=S2503_raw:GT=hom:DP=128:FQ=1:SQ=-1;ID=S2507_raw:GT=hom:DP=166:FQ=1:SQ=-1;ID=S2668_raw:GT=hom:DP=161:FQ=1:SQ=-1;ID=S2012_raw:GT=hom:DP=71:FQ=1:SQ=-1;ID=S2010_raw:GT=hom:DP=121:FQ=1:SQ=-1;ID=S2662_raw:GT=hom:DP=155:FQ=1:SQ=-1;ID=S2663_raw:GT=hom:DP=140:FQ=1:SQ=-1;ID=S2011_raw:GT=hom:DP=139:FQ=1:SQ=-1;ID=S2504_raw:GT=hom:DP=177:FQ=1:SQ=-1;ID=S2008_raw:GT=hom:DP=106:FQ=1:SQ=-1;ID=S2509_raw:GT=hom:DP=172:FQ=1:SQ=-1;ID=S2508_raw:GT=hom:DP=160:FQ=1:SQ=-1;ID=S2666_raw:GT=hom:DP=140:FQ=1:SQ=-1;ID=S2669_raw:GT=hom:DP=123:FQ=1:SQ=-1;ID=S2166_raw:GT=hom:DP=159:FQ=1:SQ=-1;ID=S1775_raw:GT=hom:DP=166:FQ=0.99:SQ=-1;ID=S2501_raw:GT=hom:DP=125:FQ=1:SQ=-1;ID=S2500_raw:GT=hom:DP=110:FQ=1:SQ=-1;ID=S2502_raw:GT=hom:DP=149:FQ=1:SQ=-1;ID=S2505_raw:GT=hom:DP=160:FQ=1:SQ=-1;ID=S2176_raw:GT=hom:DP=189:FQ=1:SQ=-1;ID=S2506_raw:GT=hom:DP=133:FQ=1:SQ=-1;ID=S2667_raw:GT=hom:DP=132:FQ=1:SQ=-1;
16	16272670	16272670	T	C	exonic	ABCC6	synonymous SNV	ABCC6:NM_001171:exon18:c.2400A>G:p.G800G,ABCC6:NM_001351800:exon18:c.2058A>G:p.G686G	rs7500834	.	ID=S2009_raw:GT=hom:DP=69:FQ=1:SQ=-1;ID=S2503_raw:GT=hom:DP=138:FQ=1:SQ=-1;ID=S2507_raw:GT=hom:DP=123:FQ=1:SQ=-1;ID=S2668_raw:GT=hom:DP=161:FQ=1:SQ=-1;ID=S2012_raw:GT=hom:DP=67:FQ=1:SQ=-1;ID=S2010_raw:GT=hom:DP=105:FQ=1:SQ=-1;ID=S2662_raw:GT=hom:DP=138:FQ=1:SQ=-1;ID=S2663_raw:GT=hom:DP=132:FQ=1:SQ=-1;ID=S2011_raw:GT=hom:DP=75:FQ=1:SQ=-1;ID=S2504_raw:GT=hom:DP=152:FQ=1:SQ=-1;ID=S2008_raw:GT=hom:DP=70:FQ=1:SQ=-1;ID=S2509_raw:GT=hom:DP=107:FQ=1:SQ=-1;ID=S2508_raw:GT=hom:DP=134:FQ=1:SQ=-1;ID=S2666_raw:GT=hom:DP=168:FQ=1:SQ=-1;ID=S2669_raw:GT=hom:DP=142:FQ=1:SQ=-1;ID=S2166_raw:GT=hom:DP=83:FQ=1:SQ=-1;ID=S1775_raw:GT=hom:DP=55:FQ=1:SQ=-1;ID=S2501_raw:GT=hom:DP=102:FQ=1:SQ=-1;ID=S2500_raw:GT=hom:DP=139:FQ=1:SQ=-1;ID=S2502_raw:GT=hom:DP=180:FQ=1:SQ=-1;ID=S2505_raw:GT=hom:DP=142:FQ=1:SQ=-1;ID=S2176_raw:GT=hom:DP=93:FQ=1:SQ=-1;ID=S2506_raw:GT=hom:DP=164:FQ=1:SQ=-1;ID=S2667_raw:GT=hom:DP=130:FQ=1:SQ=-1;
17	61556527	61556527	A	G	intronic	ACE	.	.	rs4296	.	ID=S2009_raw:GT=hom:DP=63:FQ=1:SQ=-1;ID=S2503_raw:GT=hom:DP=24:FQ=1:SQ=-1;ID=S2507_raw:GT=hom:DP=15:FQ=1:SQ=-1;ID=S2668_raw:GT=hom:DP=29:FQ=1:SQ=-1;ID=S2012_raw:GT=hom:DP=64:FQ=1:SQ=-1;ID=S2010_raw:GT=hom:DP=66:FQ=1:SQ=-1;ID=S2662_raw:GT=hom:DP=21:FQ=0.95:SQ=-1;ID=S2663_raw:GT=hom:DP=15:FQ=1:SQ=-1;ID=S2011_raw:GT=hom:DP=86:FQ=1:SQ=-1;ID=S2504_raw:GT=hom:DP=32:FQ=1:SQ=-1;ID=S2008_raw:GT=hom:DP=67:FQ=1:SQ=-1;ID=S2509_raw:GT=hom:DP=27:FQ=1:SQ=-1;ID=S2508_raw:GT=hom:DP=37:FQ=1:SQ=-1;ID=S2666_raw:GT=hom:DP=17:FQ=1:SQ=-1;ID=S2669_raw:GT=hom:DP=22:FQ=1:SQ=-1;ID=S2166_raw:GT=hom:DP=60:FQ=1:SQ=-1;ID=S1775_raw:GT=hom:DP=45:FQ=1:SQ=-1;ID=S2501_raw:GT=hom:DP=33:FQ=1:SQ=-1;ID=S2500_raw:GT=hom:DP=34:FQ=1:SQ=-1;ID=S2502_raw:GT=hom:DP=25:FQ=1:SQ=-1;ID=S2505_raw:GT=hom:DP=23:FQ=1:SQ=-1;ID=S2176_raw:GT=hom:DP=32:FQ=1:SQ=-1;ID=S2506_raw:GT=hom:DP=33:FQ=1:SQ=-1;ID=S2667_raw:GT=hom:DP=28:FQ=1:SQ=-1;
7	5568493	5568493	A	G	intronic	ACTB	.	.	rs852422	.	ID=S2009_raw:GT=hom:DP=2:FQ=1:SQ=-1;ID=S2503_raw:GT=hom:DP=52:FQ=1:SQ=-1;ID=S2507_raw:GT=hom:DP=38:FQ=1:SQ=-1;ID=S2668_raw:GT=hom:DP=42:FQ=1:SQ=-1;ID=S2012_raw:GT=hom:DP=1:FQ=1:SQ=-1;ID=S2010_raw:GT=hom:DP=2:FQ=1:SQ=-1;ID=S2662_raw:GT=hom:DP=69:FQ=1:SQ=-1;ID=S2663_raw:GT=hom:DP=37:FQ=1:SQ=-1;ID=S2011_raw:GT=hom:DP=6:FQ=1:SQ=-1;ID=S2504_raw:GT=hom:DP=59:FQ=1:SQ=-1;ID=S2008_raw:GT=hom:DP=5:FQ=1:SQ=-1;ID=S2509_raw:GT=hom:DP=44:FQ=1:SQ=-1;ID=S2508_raw:GT=hom:DP=30:FQ=1:SQ=-1;ID=S2666_raw:GT=hom:DP=56:FQ=1:SQ=-1;ID=S2669_raw:GT=hom:DP=24:FQ=1:SQ=-1;ID=S2166_raw:GT=hom:DP=28:FQ=1:SQ=-1;ID=S1775_raw:GT=hom:DP=16:FQ=1:SQ=-1;ID=S2501_raw:GT=hom:DP=52:FQ=1:SQ=-1;ID=S2500_raw:GT=hom:DP=41:FQ=1:SQ=-1;ID=S2502_raw:GT=hom:DP=56:FQ=1:SQ=-1;ID=S2505_raw:GT=hom:DP=63:FQ=1:SQ=-1;ID=S2176_raw:GT=hom:DP=31:FQ=1:SQ=-1;ID=S2506_raw:GT=hom:DP=36:FQ=1:SQ=-1;ID=S2667_raw:GT=hom:DP=29:FQ=1:SQ=-1;
20	43254490	43254490	A	G	intronic	ADA	.	.	rs244074	.	ID=S2009_raw:GT=hom:DP=2:FQ=1:SQ=-1;ID=S2503_raw:GT=hom:DP=18:FQ=1:SQ=-1;ID=S2507_raw:GT=hom:DP=14:FQ=1:SQ=-1;ID=S2668_raw:GT=hom:DP=31:FQ=1:SQ=-1;ID=S2012_raw:GT=hom:DP=6:FQ=1:SQ=-1;ID=S2010_raw:GT=hom:DP=4:FQ=1:SQ=-1;ID=S2662_raw:GT=hom:DP=28:FQ=1:SQ=-1;ID=S2663_raw:GT=hom:DP=27:FQ=1:SQ=-1;ID=S2011_raw:GT=hom:DP=6:FQ=1:SQ=-1;ID=S2504_raw:GT=hom:DP=27:FQ=1:SQ=-1;ID=S2008_raw:GT=hom:DP=6:FQ=1:SQ=-1;ID=S2509_raw:GT=hom:DP=16:FQ=1:SQ=-1;ID=S2508_raw:GT=hom:DP=26:FQ=1:SQ=-1;ID=S2666_raw:GT=hom:DP=35:FQ=1:SQ=-1;ID=S2669_raw:GT=hom:DP=10:FQ=1:SQ=-1;ID=S2166_raw:GT=hom:DP=14:FQ=1:SQ=-1;ID=S1775_raw:GT=hom:DP=10:FQ=1:SQ=-1;ID=S2501_raw:GT=hom:DP=9:FQ=1:SQ=-1;ID=S2500_raw:GT=hom:DP=32:FQ=1:SQ=-1;ID=S2502_raw:GT=hom:DP=38:FQ=1:SQ=-1;ID=S2505_raw:GT=hom:DP=20:FQ=1:SQ=-1;ID=S2176_raw:GT=hom:DP=23:FQ=1:SQ=-1;ID=S2506_raw:GT=hom:DP=21:FQ=0.95:SQ=-1;ID=S2667_raw:GT=hom:DP=24:FQ=1:SQ=-1;
20	43264927	43264927	C	T	exonic;splicing	ADA	synonymous SNV	ADA:NM_000022:exon2:c.36G>A:p.V12V,ADA:NM_001322051:exon2:c.36G>A:p.V12V	rs394105	Benign	ID=S2009_raw:GT=hom:DP=29:FQ=1:SQ=-1;ID=S2503_raw:GT=hom:DP=92:FQ=1:SQ=-1;ID=S2507_raw:GT=hom:DP=81:FQ=1:SQ=-1;ID=S2668_raw:GT=hom:DP=157:FQ=1:SQ=-1;ID=S2012_raw:GT=hom:DP=30:FQ=1:SQ=-1;ID=S2010_raw:GT=hom:DP=30:FQ=1:SQ=-1;ID=S2662_raw:GT=hom:DP=60:FQ=1:SQ=-1;ID=S2663_raw:GT=hom:DP=102:FQ=1:SQ=-1;ID=S2011_raw:GT=hom:DP=41:FQ=1:SQ=-1;ID=S2504_raw:GT=hom:DP=124:FQ=1:SQ=-1;ID=S2008_raw:GT=hom:DP=38:FQ=1:SQ=-1;ID=S2509_raw:GT=hom:DP=74:FQ=1:SQ=-1;ID=S2508_raw:GT=hom:DP=94:FQ=1:SQ=-1;ID=S2666_raw:GT=hom:DP=143:FQ=1:SQ=-1;ID=S2669_raw:GT=hom:DP=92:FQ=1:SQ=-1;ID=S2166_raw:GT=hom:DP=54:FQ=1:SQ=-1;ID=S1775_raw:GT=hom:DP=45:FQ=1:SQ=-1;ID=S2501_raw:GT=hom:DP=119:FQ=1:SQ=-1;ID=S2500_raw:GT=hom:DP=92:FQ=1:SQ=-1;ID=S2502_raw:GT=hom:DP=106:FQ=1:SQ=-1;ID=S2505_raw:GT=hom:DP=83:FQ=1:SQ=-1;ID=S2176_raw:GT=hom:DP=62:FQ=1:SQ=-1;ID=S2506_raw:GT=hom:DP=94:FQ=1:SQ=-1;ID=S2667_raw:GT=hom:DP=119:FQ=1:SQ=-1;
4	73433315	73433315	G	C	intronic	ADAMTS3	.	.	rs6830556	.	ID=S2009_raw:GT=hom:DP=7:FQ=1:SQ=-1;ID=S2503_raw:GT=hom:DP=56:FQ=1:SQ=-1;ID=S2507_raw:GT=hom:DP=59:FQ=1:SQ=-1;ID=S2668_raw:GT=hom:DP=79:FQ=1:SQ=-1;ID=S2012_raw:GT=hom:DP=4:FQ=1:SQ=-1;ID=S2010_raw:GT=hom:DP=6:FQ=1:SQ=-1;ID=S2662_raw:GT=hom:DP=19:FQ=1:SQ=-1;ID=S2663_raw:GT=hom:DP=77:FQ=1:SQ=-1;ID=S2011_raw:GT=hom:DP=8:FQ=1:SQ=-1;ID=S2504_raw:GT=hom:DP=66:FQ=1:SQ=-1;ID=S2008_raw:GT=hom:DP=11:FQ=1:SQ=-1;ID=S2509_raw:GT=hom:DP=38:FQ=1:SQ=-1;ID=S2508_raw:GT=hom:DP=69:FQ=1:SQ=-1;ID=S2666_raw:GT=hom:DP=58:FQ=1:SQ=-1;ID=S2669_raw:GT=hom:DP=59:FQ=1:SQ=-1;ID=S2166_raw:GT=hom:DP=58:FQ=1:SQ=-1;ID=S1775_raw:GT=hom:DP=45:FQ=1:SQ=-1;ID=S2501_raw:GT=hom:DP=47:FQ=1:SQ=-1;ID=S2500_raw:GT=hom:DP=33:FQ=1:SQ=-1;ID=S2502_raw:GT=hom:DP=66:FQ=1:SQ=-1;ID=S2505_raw:GT=hom:DP=50:FQ=1:SQ=-1;ID=S2176_raw:GT=hom:DP=71:FQ=1:SQ=-1;ID=S2506_raw:GT=hom:DP=36:FQ=1:SQ=-1;ID=S2667_raw:GT=hom:DP=66:FQ=1:SQ=-1;
4	73434388	73434388	T	-	intronic	ADAMTS3	.	.	.	.	ID=S2009_raw:GT=hom:DP=31:FQ=1:SQ=-1;ID=S2503_raw:GT=hom:DP=65:FQ=1:SQ=-1;ID=S2507_raw:GT=hom:DP=63:FQ=1:SQ=-1;ID=S2668_raw:GT=hom:DP=60:FQ=1:SQ=-1;ID=S2012_raw:GT=hom:DP=41:FQ=1:SQ=-1;ID=S2010_raw:GT=hom:DP=53:FQ=1:SQ=-1;ID=S2662_raw:GT=hom:DP=59:FQ=0.97:SQ=-1;ID=S2663_raw:GT=hom:DP=62:FQ=1:SQ=-1;ID=S2011_raw:GT=hom:DP=35:FQ=1:SQ=-1;ID=S2504_raw:GT=hom:DP=62:FQ=1:SQ=-1;ID=S2008_raw:GT=hom:DP=41:FQ=1:SQ=-1;ID=S2509_raw:GT=hom:DP=64:FQ=1:SQ=-1;ID=S2508_raw:GT=hom:DP=84:FQ=1:SQ=-1;ID=S2666_raw:GT=hom:DP=55:FQ=1:SQ=-1;ID=S2669_raw:GT=hom:DP=54:FQ=1:SQ=-1;ID=S2166_raw:GT=hom:DP=62:FQ=1:SQ=-1;ID=S1775_raw:GT=hom:DP=55:FQ=1:SQ=-1;ID=S2501_raw:GT=hom:DP=64:FQ=1:SQ=-1;ID=S2500_raw:GT=hom:DP=60:FQ=1:SQ=-1;ID=S2502_raw:GT=hom:DP=72:FQ=1:SQ=-1;ID=S2505_raw:GT=hom:DP=66:FQ=1:SQ=-1;ID=S2176_raw:GT=hom:DP=113:FQ=1:SQ=-1;ID=S2506_raw:GT=hom:DP=68:FQ=1:SQ=-1;ID=S2667_raw:GT=hom:DP=39:FQ=1:SQ=-1;
9	1,36E+08	1,36E+08	A	G	intronic	ADAMTS13	.	.	rs3118668	.	ID=S2009_raw:GT=hom:DP=4:FQ=1:SQ=-1;ID=S2503_raw:GT=hom:DP=68:FQ=1:SQ=-1;ID=S2507_raw:GT=hom:DP=46:FQ=1:SQ=-1;ID=S2668_raw:GT=hom:DP=63:FQ=1:SQ=-1;ID=S2012_raw:GT=hom:DP=4:FQ=1:SQ=-1;ID=S2010_raw:GT=hom:DP=7:FQ=1:SQ=-1;ID=S2662_raw:GT=hom:DP=96:FQ=1:SQ=-1;ID=S2663_raw:GT=hom:DP=37:FQ=1:SQ=-1;ID=S2011_raw:GT=hom:DP=8:FQ=1:SQ=-1;ID=S2504_raw:GT=hom:DP=41:FQ=1:SQ=-1;ID=S2008_raw:GT=hom:DP=4:FQ=1:SQ=-1;ID=S2509_raw:GT=hom:DP=52:FQ=1:SQ=-1;ID=S2508_raw:GT=hom:DP=50:FQ=1:SQ=-1;ID=S2666_raw:GT=hom:DP=46:FQ=1:SQ=-1;ID=S2669_raw:GT=hom:DP=33:FQ=1:SQ=-1;ID=S2166_raw:GT=hom:DP=37:FQ=1:SQ=-1;ID=S1775_raw:GT=hom:DP=12:FQ=1:SQ=-1;ID=S2501_raw:GT=hom:DP=63:FQ=1:SQ=-1;ID=S2500_raw:GT=hom:DP=50:FQ=1:SQ=-1;ID=S2502_raw:GT=hom:DP=58:FQ=1:SQ=-1;ID=S2505_raw:GT=hom:DP=59:FQ=1:SQ=-1;ID=S2176_raw:GT=hom:DP=37:FQ=1:SQ=-1;ID=S2506_raw:GT=hom:DP=43:FQ=1:SQ=-1;ID=S2667_raw:GT=hom:DP=63:FQ=1:SQ=-1;
9	1,36E+08	1,36E+08	A	C	intronic	ADAMTS13	.	.	rs3124773	.	ID=S2009_raw:GT=hom:DP=233:FQ=1:SQ=-1;ID=S2503_raw:GT=hom:DP=98:FQ=1:SQ=-1;ID=S2507_raw:GT=hom:DP=114:FQ=1:SQ=-1;ID=S2668_raw:GT=hom:DP=137:FQ=1:SQ=-1;ID=S2012_raw:GT=hom:DP=272:FQ=1:SQ=-1;ID=S2010_raw:GT=hom:DP=269:FQ=1:SQ=-1;ID=S2662_raw:GT=hom:DP=208:FQ=1:SQ=-1;ID=S2663_raw:GT=hom:DP=119:FQ=1:SQ=-1;ID=S2011_raw:GT=hom:DP=359:FQ=1:SQ=-1;ID=S2504_raw:GT=hom:DP=128:FQ=0.98:SQ=-1;ID=S2008_raw:GT=hom:DP=317:FQ=1:SQ=-1;ID=S2509_raw:GT=hom:DP=85:FQ=1:SQ=-1;ID=S2508_raw:GT=hom:DP=116:FQ=1:SQ=-1;ID=S2666_raw:GT=hom:DP=81:FQ=1:SQ=-1;ID=S2669_raw:GT=hom:DP=129:FQ=1:SQ=-1;ID=S2166_raw:GT=hom:DP=88:FQ=1:SQ=-1;ID=S1775_raw:GT=hom:DP=47:FQ=1:SQ=-1;ID=S2501_raw:GT=hom:DP=89:FQ=1:SQ=-1;ID=S2500_raw:GT=hom:DP=99:FQ=1:SQ=-1;ID=S2502_raw:GT=hom:DP=129:FQ=1:SQ=-1;ID=S2505_raw:GT=hom:DP=93:FQ=1:SQ=-1;ID=S2176_raw:GT=hom:DP=102:FQ=1:SQ=-1;ID=S2506_raw:GT=hom:DP=107:FQ=1:SQ=-1;ID=S2667_raw:GT=hom:DP=140:FQ=1:SQ=-1;
1	1,68E+08	1,68E+08	G	C	intronic	ADCY10	.	.	rs4657721	.	ID=S2009_raw:GT=hom:DP=4:FQ=1:SQ=-1;ID=S2503_raw:GT=hom:DP=9:FQ=1:SQ=-1;ID=S2507_raw:GT=hom:DP=15:FQ=1:SQ=-1;ID=S2668_raw:GT=hom:DP=21:FQ=1:SQ=-1;ID=S2012_raw:GT=hom:DP=3:FQ=1:SQ=-1;ID=S2010_raw:GT=hom:DP=7:FQ=1:SQ=-1;ID=S2662_raw:GT=hom:DP=63:FQ=1:SQ=-1;ID=S2663_raw:GT=hom:DP=38:FQ=1:SQ=-1;ID=S2011_raw:GT=hom:DP=3:FQ=1:SQ=-1;ID=S2504_raw:GT=hom:DP=18:FQ=1:SQ=-1;ID=S2008_raw:GT=hom:DP=4:FQ=1:SQ=-1;ID=S2509_raw:GT=hom:DP=21:FQ=1:SQ=-1;ID=S2508_raw:GT=hom:DP=26:FQ=1:SQ=-1;ID=S2666_raw:GT=hom:DP=4:FQ=1:SQ=-1;ID=S2669_raw:GT=hom:DP=46:FQ=1:SQ=-1;ID=S2166_raw:GT=hom:DP=22:FQ=1:SQ=-1;ID=S1775_raw:GT=hom:DP=9:FQ=1:SQ=-1;ID=S2501_raw:GT=hom:DP=18:FQ=1:SQ=-1;ID=S2500_raw:GT=hom:DP=19:FQ=1:SQ=-1;ID=S2502_raw:GT=hom:DP=18:FQ=1:SQ=-1;ID=S2505_raw:GT=hom:DP=24:FQ=1:SQ=-1;ID=S2176_raw:GT=hom:DP=22:FQ=1:SQ=-1;ID=S2506_raw:GT=hom:DP=3:FQ=1:SQ=-1;ID=S2667_raw:GT=hom:DP=39:FQ=1:SQ=-1;
2	1E+08	1E+08	C	T	exonic	AFF3	nonsynonymous SNV	AFF3:NM_001025108:exon10:c.1148G>A:p.S383N,AFF3:NM_002285:exon10:c.1073G>A:p.S358N	rs4851223	.	ID=S2009_raw:GT=hom:DP=119:FQ=1:SQ=-1;ID=S2503_raw:GT=hom:DP=116:FQ=1:SQ=-1;ID=S2507_raw:GT=hom:DP=123:FQ=1:SQ=-1;ID=S2668_raw:GT=hom:DP=166:FQ=1:SQ=-1;ID=S2012_raw:GT=hom:DP=129:FQ=1:SQ=-1;ID=S2010_raw:GT=hom:DP=152:FQ=1:SQ=-1;ID=S2662_raw:GT=hom:DP=4:FQ=1:SQ=-1;ID=S2663_raw:GT=hom:DP=101:FQ=1:SQ=-1;ID=S2011_raw:GT=hom:DP=179:FQ=1:SQ=-1;ID=S2504_raw:GT=hom:DP=133:FQ=1:SQ=-1;ID=S2008_raw:GT=hom:DP=125:FQ=1:SQ=-1;ID=S2509_raw:GT=hom:DP=107:FQ=1:SQ=-1;ID=S2508_raw:GT=hom:DP=109:FQ=1:SQ=-1;ID=S2666_raw:GT=hom:DP=168:FQ=1:SQ=-1;ID=S2669_raw:GT=hom:DP=55:FQ=1:SQ=-1;ID=S2166_raw:GT=hom:DP=175:FQ=1:SQ=-1;ID=S1775_raw:GT=hom:DP=153:FQ=1:SQ=-1;ID=S2501_raw:GT=hom:DP=103:FQ=1:SQ=-1;ID=S2500_raw:GT=hom:DP=140:FQ=1:SQ=-1;ID=S2502_raw:GT=hom:DP=102:FQ=1:SQ=-1;ID=S2505_raw:GT=hom:DP=95:FQ=1:SQ=-1;ID=S2176_raw:GT=hom:DP=167:FQ=1:SQ=-1;ID=S2506_raw:GT=hom:DP=83:FQ=1:SQ=-1;ID=S2667_raw:GT=hom:DP=108:FQ=1:SQ=-1;
6	1,36E+08	1,36E+08	T	C	intronic	AHI1	.	.	rs2614270	.	ID=S2009_raw:GT=hom:DP=9:FQ=1:SQ=-1;ID=S2503_raw:GT=hom:DP=5:FQ=1:SQ=-1;ID=S2507_raw:GT=hom:DP=4:FQ=1:SQ=-1;ID=S2668_raw:GT=hom:DP=4:FQ=1:SQ=-1;ID=S2012_raw:GT=hom:DP=18:FQ=1:SQ=-1;ID=S2010_raw:GT=hom:DP=15:FQ=1:SQ=-1;ID=S2662_raw:GT=hom:DP=2:FQ=1:SQ=-1;ID=S2663_raw:GT=hom:DP=8:FQ=1:SQ=-1;ID=S2011_raw:GT=hom:DP=20:FQ=1:SQ=-1;ID=S2504_raw:GT=hom:DP=6:FQ=1:SQ=-1;ID=S2008_raw:GT=hom:DP=15:FQ=1:SQ=-1;ID=S2509_raw:GT=hom:DP=9:FQ=1:SQ=-1;ID=S2508_raw:GT=hom:DP=8:FQ=1:SQ=-1;ID=S2666_raw:GT=hom:DP=7:FQ=1:SQ=-1;ID=S2669_raw:GT=hom:DP=1:FQ=1:SQ=-1;ID=S2166_raw:GT=hom:DP=10:FQ=1:SQ=-1;ID=S1775_raw:GT=hom:DP=5:FQ=1:SQ=-1;ID=S2501_raw:GT=hom:DP=12:FQ=1:SQ=-1;ID=S2500_raw:GT=hom:DP=5:FQ=1:SQ=-1;ID=S2502_raw:GT=hom:DP=8:FQ=1:SQ=-1;ID=S2505_raw:GT=hom:DP=6:FQ=1:SQ=-1;ID=S2176_raw:GT=hom:DP=5:FQ=1:SQ=-1;ID=S2506_raw:GT=hom:DP=2:FQ=1:SQ=-1;ID=S2667_raw:GT=hom:DP=2:FQ=1:SQ=-1;
11	67258391	67258391	A	G	exonic	AIP	nonsynonymous SNV	AIP:NM_001302959:exon6:c.743A>G:p.Q248R,AIP:NM_003977:exon6:c.920A>G:p.Q307R	rs4930199	Benign	ID=S2009_raw:GT=hom:DP=34:FQ=1:SQ=-1;ID=S2503_raw:GT=hom:DP=113:FQ=1:SQ=-1;ID=S2507_raw:GT=hom:DP=91:FQ=1:SQ=-1;ID=S2668_raw:GT=hom:DP=139:FQ=1:SQ=-1;ID=S2012_raw:GT=hom:DP=33:FQ=1:SQ=-1;ID=S2010_raw:GT=hom:DP=43:FQ=1:SQ=-1;ID=S2662_raw:GT=hom:DP=264:FQ=1:SQ=-1;ID=S2663_raw:GT=hom:DP=122:FQ=1:SQ=-1;ID=S2011_raw:GT=hom:DP=56:FQ=1:SQ=-1;ID=S2504_raw:GT=hom:DP=104:FQ=1:SQ=-1;ID=S2008_raw:GT=hom:DP=48:FQ=1:SQ=-1;ID=S2509_raw:GT=hom:DP=133:FQ=1:SQ=-1;ID=S2508_raw:GT=hom:DP=80:FQ=1:SQ=-1;ID=S2666_raw:GT=hom:DP=115:FQ=1:SQ=-1;ID=S2669_raw:GT=hom:DP=116:FQ=1:SQ=-1;ID=S2166_raw:GT=hom:DP=117:FQ=1:SQ=-1;ID=S1775_raw:GT=hom:DP=116:FQ=1:SQ=-1;ID=S2501_raw:GT=hom:DP=144:FQ=1:SQ=-1;ID=S2500_raw:GT=hom:DP=97:FQ=1:SQ=-1;ID=S2502_raw:GT=hom:DP=107:FQ=1:SQ=-1;ID=S2505_raw:GT=hom:DP=96:FQ=1:SQ=-1;ID=S2176_raw:GT=hom:DP=121:FQ=1:SQ=-1;ID=S2506_raw:GT=hom:DP=96:FQ=1:SQ=-1;ID=S2667_raw:GT=hom:DP=158:FQ=1:SQ=-1;
1	19208146	19208146	-	GTTGCCTG	intronic	ALDH4A1	.	.	.	.	ID=S2009_raw:GT=hom:DP=12:FQ=1:SQ=-1;ID=S2503_raw:GT=hom:DP=37:FQ=1:SQ=-1;ID=S2507_raw:GT=hom:DP=26:FQ=1:SQ=-1;ID=S2668_raw:GT=hom:DP=37:FQ=1:SQ=-1;ID=S2012_raw:GT=hom:DP=12:FQ=1:SQ=-1;ID=S2010_raw:GT=hom:DP=12:FQ=1:SQ=-1;ID=S2662_raw:GT=hom:DP=41:FQ=1:SQ=-1;ID=S2663_raw:GT=hom:DP=21:FQ=1:SQ=-1;ID=S2011_raw:GT=hom:DP=15:FQ=1:SQ=-1;ID=S2504_raw:GT=hom:DP=31:FQ=1:SQ=-1;ID=S2008_raw:GT=hom:DP=19:FQ=1:SQ=-1;ID=S2509_raw:GT=hom:DP=23:FQ=1:SQ=-1;ID=S2508_raw:GT=hom:DP=30:FQ=1:SQ=-1;ID=S2666_raw:GT=hom:DP=22:FQ=1:SQ=-1;ID=S2669_raw:GT=hom:DP=20:FQ=1:SQ=-1;ID=S2166_raw:GT=hom:DP=61:FQ=1:SQ=-1;ID=S1775_raw:GT=hom:DP=33:FQ=1:SQ=-1;ID=S2501_raw:GT=hom:DP=30:FQ=1:SQ=-1;ID=S2500_raw:GT=hom:DP=32:FQ=1:SQ=-1;ID=S2502_raw:GT=hom:DP=37:FQ=1:SQ=-1;ID=S2505_raw:GT=hom:DP=33:FQ=1:SQ=-1;ID=S2176_raw:GT=hom:DP=53:FQ=1:SQ=-1;ID=S2506_raw:GT=hom:DP=30:FQ=1:SQ=-1;ID=S2667_raw:GT=hom:DP=27:FQ=1:SQ=-1;
1	19209543	19209543	A	G	intronic	ALDH4A1	.	.	rs6426611	.	ID=S2009_raw:GT=hom:DP=42:FQ=1:SQ=-1;ID=S2503_raw:GT=hom:DP=57:FQ=1:SQ=-1;ID=S2507_raw:GT=hom:DP=60:FQ=1:SQ=-1;ID=S2668_raw:GT=hom:DP=58:FQ=1:SQ=-1;ID=S2012_raw:GT=hom:DP=37:FQ=1:SQ=-1;ID=S2010_raw:GT=hom:DP=49:FQ=1:SQ=-1;ID=S2662_raw:GT=hom:DP=111:FQ=1:SQ=-1;ID=S2663_raw:GT=hom:DP=70:FQ=1:SQ=-1;ID=S2011_raw:GT=hom:DP=79:FQ=1:SQ=-1;ID=S2504_raw:GT=hom:DP=82:FQ=1:SQ=-1;ID=S2008_raw:GT=hom:DP=48:FQ=1:SQ=-1;ID=S2509_raw:GT=hom:DP=86:FQ=1:SQ=-1;ID=S2508_raw:GT=hom:DP=91:FQ=1:SQ=-1;ID=S2666_raw:GT=hom:DP=38:FQ=1:SQ=-1;ID=S2669_raw:GT=hom:DP=73:FQ=1:SQ=-1;ID=S2166_raw:GT=hom:DP=37:FQ=1:SQ=-1;ID=S1775_raw:GT=hom:DP=32:FQ=1:SQ=-1;ID=S2501_raw:GT=hom:DP=58:FQ=1:SQ=-1;ID=S2500_raw:GT=hom:DP=64:FQ=1:SQ=-1;ID=S2502_raw:GT=hom:DP=73:FQ=1:SQ=-1;ID=S2505_raw:GT=hom:DP=89:FQ=1:SQ=-1;ID=S2176_raw:GT=hom:DP=42:FQ=1:SQ=-1;ID=S2506_raw:GT=hom:DP=74:FQ=1:SQ=-1;ID=S2667_raw:GT=hom:DP=74:FQ=1:SQ=-1;
11	77832076	77832076	T	A	intronic	ALG8	.	.	rs518624	Benign	ID=S2009_raw:GT=hom:DP=55:FQ=1:SQ=-1;ID=S2503_raw:GT=hom:DP=37:FQ=1:SQ=-1;ID=S2507_raw:GT=hom:DP=45:FQ=1:SQ=-1;ID=S2668_raw:GT=hom:DP=58:FQ=1:SQ=-1;ID=S2012_raw:GT=hom:DP=82:FQ=1:SQ=-1;ID=S2010_raw:GT=hom:DP=88:FQ=1:SQ=-1;ID=S2662_raw:GT=hom:DP=5:FQ=1:SQ=-1;ID=S2663_raw:GT=hom:DP=40:FQ=1:SQ=-1;ID=S2011_raw:GT=hom:DP=117:FQ=1:SQ=-1;ID=S2504_raw:GT=hom:DP=57:FQ=1:SQ=-1;ID=S2008_raw:GT=hom:DP=58:FQ=1:SQ=-1;ID=S2509_raw:GT=hom:DP=28:FQ=1:SQ=-1;ID=S2508_raw:GT=hom:DP=33:FQ=1:SQ=-1;ID=S2666_raw:GT=hom:DP=31:FQ=1:SQ=-1;ID=S2669_raw:GT=hom:DP=8:FQ=1:SQ=-1;ID=S2166_raw:GT=hom:DP=34:FQ=1:SQ=-1;ID=S1775_raw:GT=hom:DP=34:FQ=1:SQ=-1;ID=S2501_raw:GT=hom:DP=32:FQ=1:SQ=-1;ID=S2500_raw:GT=hom:DP=42:FQ=1:SQ=-1;ID=S2502_raw:GT=hom:DP=44:FQ=1:SQ=-1;ID=S2505_raw:GT=hom:DP=36:FQ=1:SQ=-1;ID=S2176_raw:GT=hom:DP=56:FQ=1:SQ=-1;ID=S2506_raw:GT=hom:DP=48:FQ=1:SQ=-1;ID=S2667_raw:GT=hom:DP=46:FQ=1:SQ=-1;
11	1,12E+08	1,12E+08	G	-	splicing	ALG9	.	NM_001352418:exon1:c.60+1C>-;NM_001352418:exon2:c.61-1C>-;NM_001352417:exon1:c.60+1C>-;NM_001352417:exon2:c.61-1C>-;NM_024740:exon1:c.60+1C>-;NM_024740:exon2:c.61-1C>-;NM_001077690:exon1:c.60+1C>-;NM_001077690:exon2:c.61-1C>-	rs10708475	.	ID=S2009_raw:GT=hom:DP=12:FQ=1:SQ=-1;ID=S2503_raw:GT=hom:DP=75:FQ=1:SQ=-1;ID=S2507_raw:GT=hom:DP=82:FQ=1:SQ=-1;ID=S2668_raw:GT=hom:DP=48:FQ=1:SQ=-1;ID=S2012_raw:GT=hom:DP=28:FQ=1:SQ=-1;ID=S2010_raw:GT=hom:DP=18:FQ=1:SQ=-1;ID=S2662_raw:GT=hom:DP=86:FQ=1:SQ=-1;ID=S2663_raw:GT=hom:DP=63:FQ=1:SQ=-1;ID=S2011_raw:GT=hom:DP=21:FQ=1:SQ=-1;ID=S2504_raw:GT=hom:DP=72:FQ=1:SQ=-1;ID=S2008_raw:GT=hom:DP=20:FQ=1:SQ=-1;ID=S2509_raw:GT=hom:DP=97:FQ=1:SQ=-1;ID=S2508_raw:GT=hom:DP=96:FQ=1:SQ=-1;ID=S2666_raw:GT=hom:DP=19:FQ=1:SQ=-1;ID=S2669_raw:GT=hom:DP=115:FQ=1:SQ=-1;ID=S2166_raw:GT=hom:DP=75:FQ=1:SQ=-1;ID=S1775_raw:GT=hom:DP=43:FQ=1:SQ=-1;ID=S2501_raw:GT=hom:DP=87:FQ=1:SQ=-1;ID=S2500_raw:GT=hom:DP=71:FQ=1:SQ=-1;ID=S2502_raw:GT=hom:DP=62:FQ=1:SQ=-1;ID=S2505_raw:GT=hom:DP=82:FQ=1:SQ=-1;ID=S2176_raw:GT=hom:DP=78:FQ=1:SQ=-1;ID=S2506_raw:GT=hom:DP=71:FQ=1:SQ=-1;ID=S2667_raw:GT=hom:DP=89:FQ=1:SQ=-1;
17	7991330	7991330	T	G	upstream	ALOX12B;MIR4314	.	dist=44	rs9909784	.	ID=S2009_raw:GT=hom:DP=6:FQ=1:SQ=-1;ID=S2503_raw:GT=hom:DP=23:FQ=1:SQ=-1;ID=S2507_raw:GT=hom:DP=12:FQ=1:SQ=-1;ID=S2668_raw:GT=hom:DP=14:FQ=1:SQ=-1;ID=S2012_raw:GT=hom:DP=13:FQ=1:SQ=-1;ID=S2010_raw:GT=hom:DP=9:FQ=1:SQ=-1;ID=S2662_raw:GT=hom:DP=23:FQ=1:SQ=-1;ID=S2663_raw:GT=hom:DP=25:FQ=1:SQ=-1;ID=S2011_raw:GT=hom:DP=14:FQ=1:SQ=-1;ID=S2504_raw:GT=hom:DP=14:FQ=1:SQ=-1;ID=S2008_raw:GT=hom:DP=5:FQ=1:SQ=-1;ID=S2509_raw:GT=hom:DP=20:FQ=1:SQ=-1;ID=S2508_raw:GT=hom:DP=17:FQ=1:SQ=-1;ID=S2666_raw:GT=hom:DP=11:FQ=1:SQ=-1;ID=S2669_raw:GT=hom:DP=14:FQ=1:SQ=-1;ID=S2166_raw:GT=hom:DP=18:FQ=1:SQ=-1;ID=S1775_raw:GT=hom:DP=17:FQ=1:SQ=-1;ID=S2501_raw:GT=hom:DP=9:FQ=1:SQ=-1;ID=S2500_raw:GT=hom:DP=18:FQ=1:SQ=-1;ID=S2502_raw:GT=hom:DP=21:FQ=1:SQ=-1;ID=S2505_raw:GT=hom:DP=18:FQ=1:SQ=-1;ID=S2176_raw:GT=hom:DP=13:FQ=1:SQ=-1;ID=S2506_raw:GT=hom:DP=8:FQ=1:SQ=-1;ID=S2667_raw:GT=hom:DP=21:FQ=1:SQ=-1;
1	21890464	21890464	A	G	intronic	ALPL	.	.	rs1767428	.	ID=S2009_raw:GT=hom:DP=6:FQ=1:SQ=-1;ID=S2503_raw:GT=hom:DP=34:FQ=1:SQ=-1;ID=S2507_raw:GT=hom:DP=28:FQ=1:SQ=-1;ID=S2668_raw:GT=hom:DP=28:FQ=1:SQ=-1;ID=S2012_raw:GT=hom:DP=15:FQ=1:SQ=-1;ID=S2010_raw:GT=hom:DP=7:FQ=1:SQ=-1;ID=S2662_raw:GT=hom:DP=58:FQ=1:SQ=-1;ID=S2663_raw:GT=hom:DP=33:FQ=1:SQ=-1;ID=S2011_raw:GT=hom:DP=17:FQ=1:SQ=-1;ID=S2504_raw:GT=hom:DP=38:FQ=1:SQ=-1;ID=S2008_raw:GT=hom:DP=18:FQ=1:SQ=-1;ID=S2509_raw:GT=hom:DP=30:FQ=1:SQ=-1;ID=S2508_raw:GT=hom:DP=31:FQ=1:SQ=-1;ID=S2666_raw:GT=hom:DP=17:FQ=1:SQ=-1;ID=S2669_raw:GT=hom:DP=36:FQ=1:SQ=-1;ID=S2166_raw:GT=hom:DP=25:FQ=1:SQ=-1;ID=S1775_raw:GT=hom:DP=28:FQ=1:SQ=-1;ID=S2501_raw:GT=hom:DP=27:FQ=1:SQ=-1;ID=S2500_raw:GT=hom:DP=27:FQ=1:SQ=-1;ID=S2502_raw:GT=hom:DP=31:FQ=1:SQ=-1;ID=S2505_raw:GT=hom:DP=21:FQ=1:SQ=-1;ID=S2176_raw:GT=hom:DP=25:FQ=1:SQ=-1;ID=S2506_raw:GT=hom:DP=32:FQ=1:SQ=-1;ID=S2667_raw:GT=hom:DP=35:FQ=1:SQ=-1;
11	44297054	44297054	T	C	exonic	ALX4	synonymous SNV	ALX4:NM_021926:exon2:c.621A>G:p.S207S	rs10769028	Benign	ID=S2009_raw:GT=hom:DP=160:FQ=1:SQ=-1;ID=S2503_raw:GT=hom:DP=354:FQ=1:SQ=-1;ID=S2507_raw:GT=hom:DP=420:FQ=1:SQ=-1;ID=S2668_raw:GT=hom:DP=482:FQ=1:SQ=-1;ID=S2012_raw:GT=hom:DP=150:FQ=1:SQ=-1;ID=S2010_raw:GT=hom:DP=153:FQ=1:SQ=-1;ID=S2662_raw:GT=hom:DP=695:FQ=1:SQ=-1;ID=S2663_raw:GT=hom:DP=404:FQ=1:SQ=-1;ID=S2011_raw:GT=hom:DP=206:FQ=1:SQ=-1;ID=S2504_raw:GT=hom:DP=477:FQ=1:SQ=-1;ID=S2008_raw:GT=hom:DP=185:FQ=1:SQ=-1;ID=S2509_raw:GT=hom:DP=445:FQ=1:SQ=-1;ID=S2508_raw:GT=hom:DP=493:FQ=1:SQ=-1;ID=S2666_raw:GT=hom:DP=287:FQ=1:SQ=-1;ID=S2669_raw:GT=hom:DP=490:FQ=1:SQ=-1;ID=S2166_raw:GT=hom:DP=393:FQ=1:SQ=-1;ID=S1775_raw:GT=hom:DP=280:FQ=1:SQ=-1;ID=S2501_raw:GT=hom:DP=419:FQ=1:SQ=-1;ID=S2500_raw:GT=hom:DP=373:FQ=1:SQ=-1;ID=S2502_raw:GT=hom:DP=445:FQ=1:SQ=-1;ID=S2505_raw:GT=hom:DP=423:FQ=1:SQ=-1;ID=S2176_raw:GT=hom:DP=436:FQ=1:SQ=-1;ID=S2506_raw:GT=hom:DP=434:FQ=1:SQ=-1;ID=S2667_raw:GT=hom:DP=479:FQ=1:SQ=-1;
11	44297343	44297343	C	A	intronic	ALX4	.	.	rs11037930	.	ID=S2009_raw:GT=hom:DP=4:FQ=1:SQ=-1;ID=S2503_raw:GT=hom:DP=12:FQ=1:SQ=-1;ID=S2507_raw:GT=hom:DP=16:FQ=1:SQ=-1;ID=S2668_raw:GT=hom:DP=17:FQ=1:SQ=-1;ID=S2012_raw:GT=hom:DP=9:FQ=1:SQ=-1;ID=S2010_raw:GT=hom:DP=5:FQ=1:SQ=-1;ID=S2662_raw:GT=hom:DP=16:FQ=1:SQ=-1;ID=S2663_raw:GT=hom:DP=12:FQ=1:SQ=-1;ID=S2011_raw:GT=hom:DP=10:FQ=1:SQ=-1;ID=S2504_raw:GT=hom:DP=15:FQ=1:SQ=-1;ID=S2008_raw:GT=hom:DP=4:FQ=1:SQ=-1;ID=S2509_raw:GT=hom:DP=13:FQ=1:SQ=-1;ID=S2508_raw:GT=hom:DP=10:FQ=1:SQ=-1;ID=S2666_raw:GT=hom:DP=13:FQ=1:SQ=-1;ID=S2669_raw:GT=hom:DP=7:FQ=1:SQ=-1;ID=S2166_raw:GT=hom:DP=11:FQ=1:SQ=-1;ID=S1775_raw:GT=hom:DP=8:FQ=1:SQ=-1;ID=S2501_raw:GT=hom:DP=15:FQ=1:SQ=-1;ID=S2500_raw:GT=hom:DP=12:FQ=1:SQ=-1;ID=S2502_raw:GT=hom:DP=14:FQ=1:SQ=-1;ID=S2505_raw:GT=hom:DP=18:FQ=1:SQ=-1;ID=S2176_raw:GT=hom:DP=19:FQ=1:SQ=-1;ID=S2506_raw:GT=hom:DP=19:FQ=1:SQ=-1;ID=S2667_raw:GT=hom:DP=18:FQ=1:SQ=-1;
17	4074261	4074261	A	C	intronic	ANKFY1	.	.	rs897030	.	ID=S2009_raw:GT=hom:DP=6:FQ=1:SQ=-1;ID=S2503_raw:GT=hom:DP=26:FQ=1:SQ=-1;ID=S2507_raw:GT=hom:DP=23:FQ=1:SQ=-1;ID=S2668_raw:GT=hom:DP=17:FQ=1:SQ=-1;ID=S2012_raw:GT=hom:DP=6:FQ=1:SQ=-1;ID=S2010_raw:GT=hom:DP=4:FQ=1:SQ=-1;ID=S2662_raw:GT=hom:DP=28:FQ=1:SQ=-1;ID=S2663_raw:GT=hom:DP=14:FQ=1:SQ=-1;ID=S2011_raw:GT=hom:DP=6:FQ=1:SQ=-1;ID=S2504_raw:GT=hom:DP=18:FQ=1:SQ=-1;ID=S2008_raw:GT=hom:DP=3:FQ=1:SQ=-1;ID=S2509_raw:GT=hom:DP=19:FQ=1:SQ=-1;ID=S2508_raw:GT=hom:DP=15:FQ=1:SQ=-1;ID=S2666_raw:GT=hom:DP=10:FQ=1:SQ=-1;ID=S2669_raw:GT=hom:DP=19:FQ=1:SQ=-1;ID=S2166_raw:GT=hom:DP=30:FQ=1:SQ=-1;ID=S1775_raw:GT=hom:DP=19:FQ=1:SQ=-1;ID=S2501_raw:GT=hom:DP=17:FQ=1:SQ=-1;ID=S2500_raw:GT=hom:DP=22:FQ=1:SQ=-1;ID=S2502_raw:GT=hom:DP=27:FQ=1:SQ=-1;ID=S2505_raw:GT=hom:DP=21:FQ=1:SQ=-1;ID=S2176_raw:GT=hom:DP=40:FQ=1:SQ=-1;ID=S2506_raw:GT=hom:DP=26:FQ=1:SQ=-1;ID=S2667_raw:GT=hom:DP=15:FQ=1:SQ=-1;
17	4077485	4077485	G	A	intronic	ANKFY1	.	.	rs4790585	.	ID=S2009_raw:GT=hom:DP=15:FQ=1:SQ=-1;ID=S2503_raw:GT=hom:DP=17:FQ=1:SQ=-1;ID=S2507_raw:GT=hom:DP=21:FQ=1:SQ=-1;ID=S2668_raw:GT=hom:DP=28:FQ=1:SQ=-1;ID=S2012_raw:GT=hom:DP=18:FQ=1:SQ=-1;ID=S2010_raw:GT=hom:DP=23:FQ=1:SQ=-1;ID=S2662_raw:GT=hom:DP=32:FQ=1:SQ=-1;ID=S2663_raw:GT=hom:DP=27:FQ=1:SQ=-1;ID=S2011_raw:GT=hom:DP=29:FQ=1:SQ=-1;ID=S2504_raw:GT=hom:DP=22:FQ=1:SQ=-1;ID=S2008_raw:GT=hom:DP=22:FQ=1:SQ=-1;ID=S2509_raw:GT=hom:DP=32:FQ=1:SQ=-1;ID=S2508_raw:GT=hom:DP=16:FQ=1:SQ=-1;ID=S2666_raw:GT=hom:DP=18:FQ=1:SQ=-1;ID=S2669_raw:GT=hom:DP=21:FQ=1:SQ=-1;ID=S2166_raw:GT=hom:DP=12:FQ=1:SQ=-1;ID=S1775_raw:GT=hom:DP=3:FQ=1:SQ=-1;ID=S2501_raw:GT=hom:DP=31:FQ=1:SQ=-1;ID=S2500_raw:GT=hom:DP=22:FQ=1:SQ=-1;ID=S2502_raw:GT=hom:DP=18:FQ=1:SQ=-1;ID=S2505_raw:GT=hom:DP=13:FQ=1:SQ=-1;ID=S2176_raw:GT=hom:DP=15:FQ=1:SQ=-1;ID=S2506_raw:GT=hom:DP=24:FQ=1:SQ=-1;ID=S2667_raw:GT=hom:DP=22:FQ=1:SQ=-1;
4	73991006	73991006	T	C	exonic	ANKRD17	synonymous SNV	ANKRD17:NM_198889:exon16:c.2505A>G:p.A835A,ANKRD17:NM_001286771:exon17:c.2919A>G:p.A973A,ANKRD17:NM_015574:exon17:c.3255A>G:p.A1085A,ANKRD17:NM_032217:exon17:c.3258A>G:p.A1086A	rs6822576	.	ID=S2009_raw:GT=hom:DP=118:FQ=1:SQ=-1;ID=S2503_raw:GT=hom:DP=70:FQ=1:SQ=-1;ID=S2507_raw:GT=hom:DP=57:FQ=1:SQ=-1;ID=S2668_raw:GT=hom:DP=86:FQ=1:SQ=-1;ID=S2012_raw:GT=hom:DP=130:FQ=1:SQ=-1;ID=S2010_raw:GT=hom:DP=133:FQ=1:SQ=-1;ID=S2662_raw:GT=hom:DP=14:FQ=1:SQ=-1;ID=S2663_raw:GT=hom:DP=69:FQ=1:SQ=-1;ID=S2011_raw:GT=hom:DP=171:FQ=1:SQ=-1;ID=S2504_raw:GT=hom:DP=80:FQ=1:SQ=-1;ID=S2008_raw:GT=hom:DP=140:FQ=1:SQ=-1;ID=S2509_raw:GT=hom:DP=86:FQ=1:SQ=-1;ID=S2508_raw:GT=hom:DP=74:FQ=1:SQ=-1;ID=S2666_raw:GT=hom:DP=60:FQ=1:SQ=-1;ID=S2669_raw:GT=hom:DP=58:FQ=1:SQ=-1;ID=S2166_raw:GT=hom:DP=156:FQ=1:SQ=-1;ID=S1775_raw:GT=hom:DP=65:FQ=1:SQ=-1;ID=S2501_raw:GT=hom:DP=64:FQ=1:SQ=-1;ID=S2500_raw:GT=hom:DP=67:FQ=1:SQ=-1;ID=S2502_raw:GT=hom:DP=73:FQ=1:SQ=-1;ID=S2505_raw:GT=hom:DP=51:FQ=1:SQ=-1;ID=S2176_raw:GT=hom:DP=148:FQ=1:SQ=-1;ID=S2506_raw:GT=hom:DP=75:FQ=1:SQ=-1;ID=S2667_raw:GT=hom:DP=74:FQ=1:SQ=-1;
19	1467684	1467684	A	C	exonic	APC2	synonymous SNV	APC2:NM_001351273:exon14:c.4381A>C:p.R1461R,APC2:NM_005883:exon15:c.4384A>C:p.R1462R	rs265273	.	ID=S2009_raw:GT=hom:DP=48:FQ=1:SQ=-1;ID=S2503_raw:GT=hom:DP=41:FQ=1:SQ=-1;ID=S2507_raw:GT=hom:DP=38:FQ=1:SQ=-1;ID=S2668_raw:GT=hom:DP=44:FQ=1:SQ=-1;ID=S2012_raw:GT=hom:DP=32:FQ=1:SQ=-1;ID=S2010_raw:GT=hom:DP=32:FQ=1:SQ=-1;ID=S2662_raw:GT=hom:DP=135:FQ=1:SQ=-1;ID=S2663_raw:GT=hom:DP=72:FQ=1:SQ=-1;ID=S2011_raw:GT=hom:DP=36:FQ=1:SQ=-1;ID=S2504_raw:GT=hom:DP=36:FQ=1:SQ=-1;ID=S2008_raw:GT=hom:DP=41:FQ=1:SQ=-1;ID=S2509_raw:GT=hom:DP=52:FQ=1:SQ=-1;ID=S2508_raw:GT=hom:DP=49:FQ=1:SQ=-1;ID=S2666_raw:GT=hom:DP=12:FQ=1:SQ=-1;ID=S2669_raw:GT=hom:DP=68:FQ=1:SQ=-1;ID=S2166_raw:GT=hom:DP=55:FQ=1:SQ=-1;ID=S1775_raw:GT=hom:DP=25:FQ=1:SQ=-1;ID=S2501_raw:GT=hom:DP=49:FQ=1:SQ=-1;ID=S2500_raw:GT=hom:DP=42:FQ=1:SQ=-1;ID=S2502_raw:GT=hom:DP=43:FQ=1:SQ=-1;ID=S2505_raw:GT=hom:DP=51:FQ=1:SQ=-1;ID=S2176_raw:GT=hom:DP=37:FQ=1:SQ=-1;ID=S2506_raw:GT=hom:DP=79:FQ=1:SQ=-1;ID=S2667_raw:GT=hom:DP=85:FQ=1:SQ=-1;
2	21232803	21232803	T	C	exonic	APOB	nonsynonymous SNV	APOB:NM_000384:exon26:c.6937A>G:p.I2313V	rs584542	.	ID=S2009_raw:GT=hom:DP=113:FQ=1:SQ=-1;ID=S2503_raw:GT=hom:DP=104:FQ=1:SQ=-1;ID=S2507_raw:GT=hom:DP=86:FQ=1:SQ=-1;ID=S2668_raw:GT=hom:DP=109:FQ=1:SQ=-1;ID=S2012_raw:GT=hom:DP=154:FQ=1:SQ=-1;ID=S2010_raw:GT=hom:DP=168:FQ=1:SQ=-1;ID=S2662_raw:GT=hom:DP=2:FQ=1:SQ=-1;ID=S2663_raw:GT=hom:DP=83:FQ=1:SQ=-1;ID=S2011_raw:GT=hom:DP=193:FQ=1:SQ=-1;ID=S2504_raw:GT=hom:DP=99:FQ=1:SQ=-1;ID=S2008_raw:GT=hom:DP=144:FQ=1:SQ=-1;ID=S2509_raw:GT=hom:DP=83:FQ=1:SQ=-1;ID=S2508_raw:GT=hom:DP=94:FQ=1:SQ=-1;ID=S2666_raw:GT=hom:DP=113:FQ=1:SQ=-1;ID=S2669_raw:GT=hom:DP=40:FQ=1:SQ=-1;ID=S2166_raw:GT=hom:DP=135:FQ=1:SQ=-1;ID=S1775_raw:GT=hom:DP=87:FQ=1:SQ=-1;ID=S2501_raw:GT=hom:DP=81:FQ=1:SQ=-1;ID=S2500_raw:GT=hom:DP=113:FQ=1:SQ=-1;ID=S2502_raw:GT=hom:DP=124:FQ=1:SQ=-1;ID=S2505_raw:GT=hom:DP=56:FQ=1:SQ=-1;ID=S2176_raw:GT=hom:DP=153:FQ=1:SQ=-1;ID=S2506_raw:GT=hom:DP=91:FQ=1:SQ=-1;ID=S2667_raw:GT=hom:DP=77:FQ=1:SQ=-1;
2	21235475	21235475	T	C	exonic	APOB	nonsynonymous SNV	APOB:NM_000384:exon26:c.4265A>G:p.Y1422C	rs568413	.	ID=S2009_raw:GT=hom:DP=25:FQ=1:SQ=-1;ID=S2503_raw:GT=hom:DP=80:FQ=1:SQ=-1;ID=S2507_raw:GT=hom:DP=63:FQ=1:SQ=-1;ID=S2668_raw:GT=hom:DP=79:FQ=1:SQ=-1;ID=S2012_raw:GT=hom:DP=39:FQ=1:SQ=-1;ID=S2010_raw:GT=hom:DP=26:FQ=1:SQ=-1;ID=S2662_raw:GT=hom:DP=5:FQ=1:SQ=-1;ID=S2663_raw:GT=hom:DP=63:FQ=1:SQ=-1;ID=S2011_raw:GT=hom:DP=45:FQ=1:SQ=-1;ID=S2504_raw:GT=hom:DP=97:FQ=1:SQ=-1;ID=S2008_raw:GT=hom:DP=35:FQ=1:SQ=-1;ID=S2509_raw:GT=hom:DP=80:FQ=1:SQ=-1;ID=S2508_raw:GT=hom:DP=85:FQ=1:SQ=-1;ID=S2666_raw:GT=hom:DP=94:FQ=1:SQ=-1;ID=S2669_raw:GT=hom:DP=56:FQ=1:SQ=-1;ID=S2166_raw:GT=hom:DP=96:FQ=1:SQ=-1;ID=S1775_raw:GT=hom:DP=49:FQ=1:SQ=-1;ID=S2501_raw:GT=hom:DP=59:FQ=1:SQ=-1;ID=S2500_raw:GT=hom:DP=92:FQ=1:SQ=-1;ID=S2502_raw:GT=hom:DP=89:FQ=1:SQ=-1;ID=S2505_raw:GT=hom:DP=67:FQ=1:SQ=-1;ID=S2176_raw:GT=hom:DP=74:FQ=1:SQ=-1;ID=S2506_raw:GT=hom:DP=57:FQ=1:SQ=-1;ID=S2667_raw:GT=hom:DP=73:FQ=1:SQ=-1;
2	21236482	21236482	A	G	intronic	APOB	.	.	rs488329	.	ID=S2009_raw:GT=hom:DP=16:FQ=1:SQ=-1;ID=S2503_raw:GT=hom:DP=39:FQ=1:SQ=-1;ID=S2507_raw:GT=hom:DP=33:FQ=1:SQ=-1;ID=S2668_raw:GT=hom:DP=39:FQ=1:SQ=-1;ID=S2012_raw:GT=hom:DP=23:FQ=1:SQ=-1;ID=S2010_raw:GT=hom:DP=31:FQ=1:SQ=-1;ID=S2662_raw:GT=hom:DP=8:FQ=1:SQ=-1;ID=S2663_raw:GT=hom:DP=26:FQ=1:SQ=-1;ID=S2011_raw:GT=hom:DP=41:FQ=1:SQ=-1;ID=S2504_raw:GT=hom:DP=40:FQ=1:SQ=-1;ID=S2008_raw:GT=hom:DP=19:FQ=1:SQ=-1;ID=S2509_raw:GT=hom:DP=30:FQ=1:SQ=-1;ID=S2508_raw:GT=hom:DP=37:FQ=1:SQ=-1;ID=S2666_raw:GT=hom:DP=60:FQ=1:SQ=-1;ID=S2669_raw:GT=hom:DP=35:FQ=1:SQ=-1;ID=S2166_raw:GT=hom:DP=25:FQ=1:SQ=-1;ID=S1775_raw:GT=hom:DP=8:FQ=1:SQ=-1;ID=S2501_raw:GT=hom:DP=31:FQ=1:SQ=-1;ID=S2500_raw:GT=hom:DP=33:FQ=1:SQ=-1;ID=S2502_raw:GT=hom:DP=28:FQ=1:SQ=-1;ID=S2505_raw:GT=hom:DP=23:FQ=1:SQ=-1;ID=S2176_raw:GT=hom:DP=27:FQ=1:SQ=-1;ID=S2506_raw:GT=hom:DP=30:FQ=1:SQ=-1;ID=S2667_raw:GT=hom:DP=22:FQ=1:SQ=-1;
22	36655261	36655261	C	T	intronic	APOL1	.	.	rs136152	.	ID=S2009_raw:GT=hom:DP=21:FQ=1:SQ=-1;ID=S2503_raw:GT=hom:DP=30:FQ=1:SQ=-1;ID=S2507_raw:GT=hom:DP=26:FQ=1:SQ=-1;ID=S2668_raw:GT=hom:DP=24:FQ=1:SQ=-1;ID=S2012_raw:GT=hom:DP=23:FQ=1:SQ=-1;ID=S2010_raw:GT=hom:DP=24:FQ=0.96:SQ=-1;ID=S2662_raw:GT=hom:DP=31:FQ=1:SQ=-1;ID=S2663_raw:GT=hom:DP=21:FQ=1:SQ=-1;ID=S2011_raw:GT=hom:DP=23:FQ=1:SQ=-1;ID=S2504_raw:GT=hom:DP=23:FQ=1:SQ=-1;ID=S2008_raw:GT=hom:DP=19:FQ=1:SQ=-1;ID=S2509_raw:GT=hom:DP=23:FQ=1:SQ=-1;ID=S2508_raw:GT=hom:DP=21:FQ=1:SQ=-1;ID=S2666_raw:GT=hom:DP=24:FQ=1:SQ=-1;ID=S2669_raw:GT=hom:DP=27:FQ=1:SQ=-1;ID=S2166_raw:GT=hom:DP=23:FQ=1:SQ=-1;ID=S1775_raw:GT=hom:DP=10:FQ=1:SQ=-1;ID=S2501_raw:GT=hom:DP=22:FQ=1:SQ=-1;ID=S2500_raw:GT=hom:DP=30:FQ=1:SQ=-1;ID=S2502_raw:GT=hom:DP=24:FQ=1:SQ=-1;ID=S2505_raw:GT=hom:DP=28:FQ=1:SQ=-1;ID=S2176_raw:GT=hom:DP=19:FQ=1:SQ=-1;ID=S2506_raw:GT=hom:DP=16:FQ=1:SQ=-1;ID=S2667_raw:GT=hom:DP=12:FQ=1:SQ=-1;
4	86431994	86431997	CTCT	-	intronic	ARHGAP24	.	.	.	.	ID=S2009_raw:GT=hom:DP=16:FQ=1:SQ=-1;ID=S2503_raw:GT=hom:DP=11:FQ=1:SQ=-1;ID=S2507_raw:GT=hom:DP=4:FQ=1:SQ=-1;ID=S2668_raw:GT=hom:DP=9:FQ=1:SQ=-1;ID=S2012_raw:GT=hom:DP=15:FQ=1:SQ=-1;ID=S2010_raw:GT=hom:DP=18:FQ=1:SQ=-1;ID=S2662_raw:GT=hom:DP=2:FQ=1:SQ=-1;ID=S2663_raw:GT=hom:DP=7:FQ=1:SQ=-1;ID=S2011_raw:GT=hom:DP=26:FQ=1:SQ=-1;ID=S2504_raw:GT=hom:DP=6:FQ=1:SQ=-1;ID=S2008_raw:GT=hom:DP=19:FQ=1:SQ=-1;ID=S2509_raw:GT=hom:DP=2:FQ=1:SQ=-1;ID=S2508_raw:GT=hom:DP=10:FQ=1:SQ=-1;ID=S2666_raw:GT=hom:DP=4:FQ=1:SQ=-1;ID=S2669_raw:GT=hom:DP=6:FQ=1:SQ=-1;ID=S2166_raw:GT=hom:DP=8:FQ=1:SQ=-1;ID=S1775_raw:GT=hom:DP=2:FQ=1:SQ=-1;ID=S2501_raw:GT=hom:DP=9:FQ=1:SQ=-1;ID=S2500_raw:GT=hom:DP=12:FQ=1:SQ=-1;ID=S2502_raw:GT=hom:DP=7:FQ=1:SQ=-1;ID=S2505_raw:GT=hom:DP=4:FQ=1:SQ=-1;ID=S2176_raw:GT=hom:DP=12:FQ=1:SQ=-1;ID=S2506_raw:GT=hom:DP=4:FQ=1:SQ=-1;ID=S2667_raw:GT=hom:DP=5:FQ=1:SQ=-1;
10	1,04E+08	1,04E+08	AA	-	intronic	ARL3	.	.	rs59504153	.	ID=S2009_raw:GT=hom:DP=13:FQ=1:SQ=-1;ID=S2503_raw:GT=hom:DP=47:FQ=1:SQ=-1;ID=S2507_raw:GT=hom:DP=27:FQ=1:SQ=-1;ID=S2668_raw:GT=hom:DP=69:FQ=1:SQ=-1;ID=S2012_raw:GT=hom:DP=28:FQ=1:SQ=-1;ID=S2010_raw:GT=hom:DP=23:FQ=1:SQ=-1;ID=S2662_raw:GT=hom:DP=6:FQ=1:SQ=-1;ID=S2663_raw:GT=hom:DP=24:FQ=1:SQ=-1;ID=S2011_raw:GT=hom:DP=36:FQ=1:SQ=-1;ID=S2504_raw:GT=hom:DP=52:FQ=1:SQ=-1;ID=S2008_raw:GT=hom:DP=25:FQ=1:SQ=-1;ID=S2509_raw:GT=hom:DP=44:FQ=1:SQ=-1;ID=S2508_raw:GT=hom:DP=47:FQ=1:SQ=-1;ID=S2666_raw:GT=hom:DP=76:FQ=1:SQ=-1;ID=S2669_raw:GT=hom:DP=27:FQ=1:SQ=-1;ID=S2166_raw:GT=hom:DP=47:FQ=1:SQ=-1;ID=S1775_raw:GT=hom:DP=33:FQ=1:SQ=-1;ID=S2501_raw:GT=hom:DP=27:FQ=1:SQ=-1;ID=S2500_raw:GT=hom:DP=39:FQ=1:SQ=-1;ID=S2502_raw:GT=hom:DP=43:FQ=1:SQ=-1;ID=S2505_raw:GT=hom:DP=41:FQ=1:SQ=-1;ID=S2176_raw:GT=hom:DP=58:FQ=1:SQ=-1;ID=S2506_raw:GT=hom:DP=49:FQ=1:SQ=-1;ID=S2667_raw:GT=hom:DP=40:FQ=1:SQ=-1;
10	1,04E+08	1,04E+08	C	A	intronic	ARL3	.	.	rs2248684	.	ID=S2009_raw:GT=hom:DP=2:FQ=1:SQ=-1;ID=S2503_raw:GT=hom:DP=35:FQ=1:SQ=-1;ID=S2507_raw:GT=hom:DP=40:FQ=1:SQ=-1;ID=S2668_raw:GT=hom:DP=18:FQ=1:SQ=-1;ID=S2012_raw:GT=hom:DP=1:FQ=1:SQ=-1;ID=S2010_raw:GT=hom:DP=2:FQ=1:SQ=-1;ID=S2662_raw:GT=hom:DP=60:FQ=1:SQ=-1;ID=S2663_raw:GT=hom:DP=49:FQ=1:SQ=-1;ID=S2011_raw:GT=hom:DP=4:FQ=1:SQ=-1;ID=S2504_raw:GT=hom:DP=30:FQ=1:SQ=-1;ID=S2008_raw:GT=hom:DP=3:FQ=1:SQ=-1;ID=S2509_raw:GT=hom:DP=58:FQ=1:SQ=-1;ID=S2508_raw:GT=hom:DP=31:FQ=1:SQ=-1;ID=S2666_raw:GT=hom:DP=14:FQ=1:SQ=-1;ID=S2669_raw:GT=hom:DP=31:FQ=1:SQ=-1;ID=S2166_raw:GT=hom:DP=19:FQ=1:SQ=-1;ID=S1775_raw:GT=hom:DP=11:FQ=1:SQ=-1;ID=S2501_raw:GT=hom:DP=28:FQ=1:SQ=-1;ID=S2500_raw:GT=hom:DP=24:FQ=1:SQ=-1;ID=S2502_raw:GT=hom:DP=37:FQ=1:SQ=-1;ID=S2505_raw:GT=hom:DP=54:FQ=1:SQ=-1;ID=S2176_raw:GT=hom:DP=17:FQ=1:SQ=-1;ID=S2506_raw:GT=hom:DP=27:FQ=1:SQ=-1;ID=S2667_raw:GT=hom:DP=50:FQ=1:SQ=-1;
10	1,04E+08	1,04E+08	G	A	UTR5	ARL3	.	NM_004311:c.-75C>T	rs2248679	.	ID=S2009_raw:GT=hom:DP=45:FQ=1:SQ=-1;ID=S2503_raw:GT=hom:DP=34:FQ=1:SQ=-1;ID=S2507_raw:GT=hom:DP=42:FQ=1:SQ=-1;ID=S2668_raw:GT=hom:DP=39:FQ=1:SQ=-1;ID=S2012_raw:GT=hom:DP=34:FQ=1:SQ=-1;ID=S2010_raw:GT=hom:DP=68:FQ=1:SQ=-1;ID=S2662_raw:GT=hom:DP=66:FQ=1:SQ=-1;ID=S2663_raw:GT=hom:DP=28:FQ=1:SQ=-1;ID=S2011_raw:GT=hom:DP=77:FQ=1:SQ=-1;ID=S2504_raw:GT=hom:DP=46:FQ=1:SQ=-1;ID=S2008_raw:GT=hom:DP=64:FQ=1:SQ=-1;ID=S2509_raw:GT=hom:DP=41:FQ=1:SQ=-1;ID=S2508_raw:GT=hom:DP=66:FQ=1:SQ=-1;ID=S2666_raw:GT=hom:DP=17:FQ=1:SQ=-1;ID=S2669_raw:GT=hom:DP=49:FQ=1:SQ=-1;ID=S2166_raw:GT=hom:DP=34:FQ=1:SQ=-1;ID=S1775_raw:GT=hom:DP=33:FQ=1:SQ=-1;ID=S2501_raw:GT=hom:DP=33:FQ=1:SQ=-1;ID=S2500_raw:GT=hom:DP=26:FQ=1:SQ=-1;ID=S2502_raw:GT=hom:DP=43:FQ=1:SQ=-1;ID=S2505_raw:GT=hom:DP=67:FQ=1:SQ=-1;ID=S2176_raw:GT=hom:DP=30:FQ=1:SQ=-1;ID=S2506_raw:GT=hom:DP=42:FQ=1:SQ=-1;ID=S2667_raw:GT=hom:DP=46:FQ=1:SQ=-1;
1	1,97E+08	1,97E+08	A	G	exonic	ASPM	nonsynonymous SNV	ASPM:NM_018136:exon18:c.7480T>C:p.Y2494H	rs964201	Benign	ID=S2009_raw:GT=hom:DP=130:FQ=1:SQ=-1;ID=S2503_raw:GT=hom:DP=124:FQ=1:SQ=-1;ID=S2507_raw:GT=hom:DP=103:FQ=1:SQ=-1;ID=S2668_raw:GT=hom:DP=126:FQ=1:SQ=-1;ID=S2012_raw:GT=hom:DP=133:FQ=1:SQ=-1;ID=S2010_raw:GT=hom:DP=158:FQ=1:SQ=-1;ID=S2662_raw:GT=hom:DP=4:FQ=1:SQ=-1;ID=S2663_raw:GT=hom:DP=100:FQ=1:SQ=-1;ID=S2011_raw:GT=hom:DP=209:FQ=1:SQ=-1;ID=S2504_raw:GT=hom:DP=132:FQ=1:SQ=-1;ID=S2008_raw:GT=hom:DP=144:FQ=1:SQ=-1;ID=S2509_raw:GT=hom:DP=114:FQ=1:SQ=-1;ID=S2508_raw:GT=hom:DP=131:FQ=1:SQ=-1;ID=S2666_raw:GT=hom:DP=142:FQ=1:SQ=-1;ID=S2669_raw:GT=hom:DP=87:FQ=1:SQ=-1;ID=S2166_raw:GT=hom:DP=171:FQ=1:SQ=-1;ID=S1775_raw:GT=hom:DP=82:FQ=1:SQ=-1;ID=S2501_raw:GT=hom:DP=118:FQ=1:SQ=-1;ID=S2500_raw:GT=hom:DP=151:FQ=1:SQ=-1;ID=S2502_raw:GT=hom:DP=133:FQ=1:SQ=-1;ID=S2505_raw:GT=hom:DP=117:FQ=1:SQ=-1;ID=S2176_raw:GT=hom:DP=182:FQ=1:SQ=-1;ID=S2506_raw:GT=hom:DP=96:FQ=1:SQ=-1;ID=S2667_raw:GT=hom:DP=106:FQ=1:SQ=-1;
20	31022959	31022959	T	C	exonic	ASXL1	nonsynonymous SNV	ASXL1:NM_001363734:exon11:c.2261T>C:p.L754P,ASXL1:NM_015338:exon12:c.2444T>C:p.L815P	rs6058694	not_provided	ID=S2009_raw:GT=hom:DP=44:FQ=1:SQ=-1;ID=S2503_raw:GT=hom:DP=69:FQ=1:SQ=-1;ID=S2507_raw:GT=hom:DP=88:FQ=1:SQ=-1;ID=S2668_raw:GT=hom:DP=131:FQ=1:SQ=-1;ID=S2012_raw:GT=hom:DP=49:FQ=1:SQ=-1;ID=S2010_raw:GT=hom:DP=58:FQ=1:SQ=-1;ID=S2662_raw:GT=hom:DP=85:FQ=1:SQ=-1;ID=S2663_raw:GT=hom:DP=60:FQ=1:SQ=-1;ID=S2011_raw:GT=hom:DP=61:FQ=1:SQ=-1;ID=S2504_raw:GT=hom:DP=116:FQ=1:SQ=-1;ID=S2008_raw:GT=hom:DP=49:FQ=1:SQ=-1;ID=S2509_raw:GT=hom:DP=112:FQ=1:SQ=-1;ID=S2508_raw:GT=hom:DP=91:FQ=1:SQ=-1;ID=S2666_raw:GT=hom:DP=116:FQ=1:SQ=-1;ID=S2669_raw:GT=hom:DP=91:FQ=1:SQ=-1;ID=S2166_raw:GT=hom:DP=131:FQ=1:SQ=-1;ID=S1775_raw:GT=hom:DP=73:FQ=1:SQ=-1;ID=S2501_raw:GT=hom:DP=93:FQ=1:SQ=-1;ID=S2500_raw:GT=hom:DP=96:FQ=1:SQ=-1;ID=S2502_raw:GT=hom:DP=79:FQ=1:SQ=-1;ID=S2505_raw:GT=hom:DP=93:FQ=1:SQ=-1;ID=S2176_raw:GT=hom:DP=129:FQ=1:SQ=-1;ID=S2506_raw:GT=hom:DP=105:FQ=1:SQ=-1;ID=S2667_raw:GT=hom:DP=76:FQ=1:SQ=-1;
12	7044648	7044648	-	G	intronic	ATN1	.	.	.	.	ID=S2009_raw:GT=hom:DP=57:FQ=1:SQ=-1;ID=S2503_raw:GT=hom:DP=69:FQ=1:SQ=-1;ID=S2507_raw:GT=hom:DP=71:FQ=1:SQ=-1;ID=S2668_raw:GT=hom:DP=70:FQ=1:SQ=-1;ID=S2012_raw:GT=hom:DP=64:FQ=1:SQ=-1;ID=S2010_raw:GT=hom:DP=53:FQ=1:SQ=-1;ID=S2662_raw:GT=hom:DP=81:FQ=1:SQ=-1;ID=S2663_raw:GT=hom:DP=55:FQ=1:SQ=-1;ID=S2011_raw:GT=hom:DP=90:FQ=1:SQ=-1;ID=S2504_raw:GT=hom:DP=64:FQ=1:SQ=-1;ID=S2008_raw:GT=hom:DP=84:FQ=1:SQ=-1;ID=S2509_raw:GT=hom:DP=59:FQ=1:SQ=-1;ID=S2508_raw:GT=hom:DP=64:FQ=1:SQ=-1;ID=S2666_raw:GT=hom:DP=74:FQ=1:SQ=-1;ID=S2669_raw:GT=hom:DP=79:FQ=1:SQ=-1;ID=S2166_raw:GT=hom:DP=38:FQ=1:SQ=-1;ID=S1775_raw:GT=hom:DP=34:FQ=1:SQ=-1;ID=S2501_raw:GT=hom:DP=58:FQ=1:SQ=-1;ID=S2500_raw:GT=hom:DP=71:FQ=1:SQ=-1;ID=S2502_raw:GT=hom:DP=71:FQ=1:SQ=-1;ID=S2505_raw:GT=hom:DP=61:FQ=1:SQ=-1;ID=S2176_raw:GT=hom:DP=62:FQ=1:SQ=-1;ID=S2506_raw:GT=hom:DP=51:FQ=1:SQ=-1;ID=S2667_raw:GT=hom:DP=56:FQ=1:SQ=-1;
22	18084001	18084001	T	G	intronic	ATP6V1E1	.	.	rs5747254	.	ID=S2009_raw:GT=hom:DP=27:FQ=1:SQ=-1;ID=S2503_raw:GT=hom:DP=67:FQ=1:SQ=-1;ID=S2507_raw:GT=hom:DP=69:FQ=1:SQ=-1;ID=S2668_raw:GT=hom:DP=93:FQ=1:SQ=-1;ID=S2012_raw:GT=hom:DP=30:FQ=1:SQ=-1;ID=S2010_raw:GT=hom:DP=35:FQ=1:SQ=-1;ID=S2662_raw:GT=hom:DP=29:FQ=1:SQ=-1;ID=S2663_raw:GT=hom:DP=52:FQ=1:SQ=-1;ID=S2011_raw:GT=hom:DP=44:FQ=1:SQ=-1;ID=S2504_raw:GT=hom:DP=82:FQ=0.99:SQ=-1;ID=S2008_raw:GT=hom:DP=40:FQ=1:SQ=-1;ID=S2509_raw:GT=hom:DP=67:FQ=1:SQ=-1;ID=S2508_raw:GT=hom:DP=77:FQ=1:SQ=-1;ID=S2666_raw:GT=hom:DP=90:FQ=1:SQ=-1;ID=S2669_raw:GT=hom:DP=46:FQ=1:SQ=-1;ID=S2166_raw:GT=hom:DP=93:FQ=1:SQ=-1;ID=S1775_raw:GT=hom:DP=46:FQ=1:SQ=-1;ID=S2501_raw:GT=hom:DP=68:FQ=1:SQ=-1;ID=S2500_raw:GT=hom:DP=95:FQ=1:SQ=-1;ID=S2502_raw:GT=hom:DP=88:FQ=1:SQ=-1;ID=S2505_raw:GT=hom:DP=76:FQ=1:SQ=-1;ID=S2176_raw:GT=hom:DP=104:FQ=1:SQ=-1;ID=S2506_raw:GT=hom:DP=60:FQ=1:SQ=-1;ID=S2667_raw:GT=hom:DP=55:FQ=1:SQ=-1;
X	77296047	77296047	G	C	intronic	ATP7A	.	.	rs5913631	.	ID=S2009_raw:GT=hom:DP=12:FQ=1:SQ=-1;ID=S2503_raw:GT=hom:DP=24:FQ=1:SQ=-1;ID=S2507_raw:GT=hom:DP=34:FQ=1:SQ=-1;ID=S2668_raw:GT=hom:DP=90:FQ=1:SQ=-1;ID=S2012_raw:GT=hom:DP=16:FQ=1:SQ=-1;ID=S2010_raw:GT=hom:DP=26:FQ=1:SQ=-1;ID=S2662_raw:GT=hom:DP=3:FQ=1:SQ=-1;ID=S2663_raw:GT=hom:DP=69:FQ=1:SQ=-1;ID=S2011_raw:GT=hom:DP=29:FQ=1:SQ=-1;ID=S2504_raw:GT=hom:DP=30:FQ=1:SQ=-1;ID=S2008_raw:GT=hom:DP=32:FQ=1:SQ=-1;ID=S2509_raw:GT=hom:DP=30:FQ=1:SQ=-1;ID=S2508_raw:GT=hom:DP=34:FQ=1:SQ=-1;ID=S2666_raw:GT=hom:DP=31:FQ=0.97:SQ=-1;ID=S2669_raw:GT=hom:DP=25:FQ=1:SQ=-1;ID=S2166_raw:GT=hom:DP=65:FQ=1:SQ=-1;ID=S1775_raw:GT=hom:DP=30:FQ=1:SQ=-1;ID=S2501_raw:GT=hom:DP=43:FQ=1:SQ=-1;ID=S2500_raw:GT=hom:DP=35:FQ=1:SQ=-1;ID=S2502_raw:GT=hom:DP=77:FQ=1:SQ=-1;ID=S2505_raw:GT=hom:DP=39:FQ=1:SQ=-1;ID=S2176_raw:GT=hom:DP=76:FQ=1:SQ=-1;ID=S2506_raw:GT=hom:DP=34:FQ=1:SQ=-1;ID=S2667_raw:GT=hom:DP=60:FQ=1:SQ=-1;
12	58204283	58204283	T	C	exonic	AVIL	nonsynonymous SNV	AVIL:NM_006576:exon6:c.610A>G:p.K204E	rs2172521	.	ID=S2009_raw:GT=hom:DP=40:FQ=1:SQ=-1;ID=S2503_raw:GT=hom:DP=73:FQ=1:SQ=-1;ID=S2507_raw:GT=hom:DP=72:FQ=1:SQ=-1;ID=S2668_raw:GT=hom:DP=93:FQ=1:SQ=-1;ID=S2012_raw:GT=hom:DP=39:FQ=1:SQ=-1;ID=S2010_raw:GT=hom:DP=24:FQ=1:SQ=-1;ID=S2662_raw:GT=hom:DP=64:FQ=1:SQ=-1;ID=S2663_raw:GT=hom:DP=60:FQ=1:SQ=-1;ID=S2011_raw:GT=hom:DP=51:FQ=1:SQ=-1;ID=S2504_raw:GT=hom:DP=64:FQ=1:SQ=-1;ID=S2008_raw:GT=hom:DP=37:FQ=1:SQ=-1;ID=S2509_raw:GT=hom:DP=79:FQ=1:SQ=-1;ID=S2508_raw:GT=hom:DP=75:FQ=1:SQ=-1;ID=S2666_raw:GT=hom:DP=64:FQ=1:SQ=-1;ID=S2669_raw:GT=hom:DP=74:FQ=1:SQ=-1;ID=S2166_raw:GT=hom:DP=43:FQ=1:SQ=-1;ID=S1775_raw:GT=hom:DP=45:FQ=1:SQ=-1;ID=S2501_raw:GT=hom:DP=67:FQ=1:SQ=-1;ID=S2500_raw:GT=hom:DP=71:FQ=1:SQ=-1;ID=S2502_raw:GT=hom:DP=68:FQ=1:SQ=-1;ID=S2505_raw:GT=hom:DP=56:FQ=1:SQ=-1;ID=S2176_raw:GT=hom:DP=48:FQ=1:SQ=-1;ID=S2506_raw:GT=hom:DP=76:FQ=1:SQ=-1;ID=S2667_raw:GT=hom:DP=86:FQ=1:SQ=-1;
X	1,53E+08	1,53E+08	-	C	UTR5	AVPR2	.	NM_000054:c.-153_-152insC;NM_001146151:c.-153_-152insC	rs55712963	.	ID=S2009_raw:GT=hom:DP=3:FQ=1:SQ=-1;ID=S2503_raw:GT=hom:DP=11:FQ=1:SQ=-1;ID=S2507_raw:GT=hom:DP=9:FQ=1:SQ=-1;ID=S2668_raw:GT=hom:DP=19:FQ=1:SQ=-1;ID=S2012_raw:GT=hom:DP=4:FQ=1:SQ=-1;ID=S2010_raw:GT=hom:DP=14:FQ=1:SQ=-1;ID=S2662_raw:GT=hom:DP=22:FQ=1:SQ=-1;ID=S2663_raw:GT=hom:DP=12:FQ=1:SQ=-1;ID=S2011_raw:GT=hom:DP=10:FQ=1:SQ=-1;ID=S2504_raw:GT=hom:DP=10:FQ=1:SQ=-1;ID=S2008_raw:GT=hom:DP=13:FQ=1:SQ=-1;ID=S2509_raw:GT=hom:DP=8:FQ=1:SQ=-1;ID=S2508_raw:GT=hom:DP=8:FQ=1:SQ=-1;ID=S2666_raw:GT=hom:DP=9:FQ=1:SQ=-1;ID=S2669_raw:GT=hom:DP=6:FQ=1:SQ=-1;ID=S2166_raw:GT=hom:DP=11:FQ=1:SQ=-1;ID=S1775_raw:GT=hom:DP=7:FQ=1:SQ=-1;ID=S2501_raw:GT=hom:DP=7:FQ=1:SQ=-1;ID=S2500_raw:GT=hom:DP=18:FQ=1:SQ=-1;ID=S2502_raw:GT=hom:DP=26:FQ=1:SQ=-1;ID=S2505_raw:GT=hom:DP=12:FQ=1:SQ=-1;ID=S2176_raw:GT=hom:DP=12:FQ=1:SQ=-1;ID=S2506_raw:GT=hom:DP=11:FQ=1:SQ=-1;ID=S2667_raw:GT=hom:DP=12:FQ=1:SQ=-1;
X	1,53E+08	1,53E+08	T	G	splicing	AVPR2	.	NM_000054:exon3:c.26-6T>G;NM_001146151:exon3:c.26-6T>G	rs56689668	Benign/Likely_benign	ID=S2009_raw:GT=hom:DP=5:FQ=1:SQ=-1;ID=S2503_raw:GT=hom:DP=13:FQ=1:SQ=-1;ID=S2507_raw:GT=hom:DP=17:FQ=1:SQ=-1;ID=S2668_raw:GT=hom:DP=53:FQ=1:SQ=-1;ID=S2012_raw:GT=hom:DP=6:FQ=1:SQ=-1;ID=S2010_raw:GT=hom:DP=17:FQ=1:SQ=-1;ID=S2662_raw:GT=hom:DP=103:FQ=1:SQ=-1;ID=S2663_raw:GT=hom:DP=47:FQ=1:SQ=-1;ID=S2011_raw:GT=hom:DP=5:FQ=1:SQ=-1;ID=S2504_raw:GT=hom:DP=31:FQ=1:SQ=-1;ID=S2008_raw:GT=hom:DP=10:FQ=1:SQ=-1;ID=S2509_raw:GT=hom:DP=21:FQ=1:SQ=-1;ID=S2508_raw:GT=hom:DP=24:FQ=1:SQ=-1;ID=S2666_raw:GT=hom:DP=15:FQ=1:SQ=-1;ID=S2669_raw:GT=hom:DP=21:FQ=1:SQ=-1;ID=S2166_raw:GT=hom:DP=18:FQ=1:SQ=-1;ID=S1775_raw:GT=hom:DP=6:FQ=1:SQ=-1;ID=S2501_raw:GT=hom:DP=20:FQ=1:SQ=-1;ID=S2500_raw:GT=hom:DP=19:FQ=1:SQ=-1;ID=S2502_raw:GT=hom:DP=49:FQ=1:SQ=-1;ID=S2505_raw:GT=hom:DP=36:FQ=1:SQ=-1;ID=S2176_raw:GT=hom:DP=20:FQ=1:SQ=-1;ID=S2506_raw:GT=hom:DP=20:FQ=1:SQ=-1;ID=S2667_raw:GT=hom:DP=64:FQ=0.98:SQ=-1;
13	32278101	32278101	A	G	intergenic	B3GLCT;RXFP2	.	dist=371690;dist=35562	rs4627201	.	ID=S2009_raw:GT=hom:DP=5:FQ=1:SQ=-1;ID=S2503_raw:GT=hom:DP=3:FQ=1:SQ=-1;ID=S2507_raw:GT=hom:DP=7:FQ=1:SQ=-1;ID=S2668_raw:GT=hom:DP=7:FQ=1:SQ=-1;ID=S2012_raw:GT=hom:DP=3:FQ=1:SQ=-1;ID=S2010_raw:GT=hom:DP=3:FQ=1:SQ=-1;ID=S2662_raw:GT=hom:DP=4:FQ=1:SQ=-1;ID=S2663_raw:GT=hom:DP=8:FQ=1:SQ=-1;ID=S2011_raw:GT=hom:DP=5:FQ=1:SQ=-1;ID=S2504_raw:GT=hom:DP=8:FQ=1:SQ=-1;ID=S2008_raw:GT=hom:DP=3:FQ=1:SQ=-1;ID=S2509_raw:GT=hom:DP=14:FQ=1:SQ=-1;ID=S2508_raw:GT=hom:DP=7:FQ=1:SQ=-1;ID=S2666_raw:GT=hom:DP=4:FQ=1:SQ=-1;ID=S2669_raw:GT=hom:DP=6:FQ=1:SQ=-1;ID=S2166_raw:GT=hom:DP=17:FQ=1:SQ=-1;ID=S1775_raw:GT=hom:DP=12:FQ=1:SQ=-1;ID=S2501_raw:GT=hom:DP=8:FQ=1:SQ=-1;ID=S2500_raw:GT=hom:DP=7:FQ=1:SQ=-1;ID=S2502_raw:GT=hom:DP=6:FQ=1:SQ=-1;ID=S2505_raw:GT=hom:DP=5:FQ=1:SQ=-1;ID=S2176_raw:GT=hom:DP=10:FQ=1:SQ=-1;ID=S2506_raw:GT=hom:DP=3:FQ=1:SQ=-1;ID=S2667_raw:GT=hom:DP=5:FQ=1:SQ=-1;
4	1,03E+08	1,03E+08	T	C	exonic	BANK1	nonsynonymous SNV	BANK1:NM_001127507:exon10:c.1549T>C:p.C517R,BANK1:NM_001083907:exon11:c.1858T>C:p.C620R,BANK1:NM_017935:exon11:c.1948T>C:p.C650R	rs3113676	.	ID=S2009_raw:GT=hom:DP=26:FQ=1:SQ=-1;ID=S2503_raw:GT=hom:DP=50:FQ=1:SQ=-1;ID=S2507_raw:GT=hom:DP=44:FQ=1:SQ=-1;ID=S2668_raw:GT=hom:DP=63:FQ=1:SQ=-1;ID=S2012_raw:GT=hom:DP=38:FQ=1:SQ=-1;ID=S2010_raw:GT=hom:DP=50:FQ=1:SQ=-1;ID=S2662_raw:GT=hom:DP=2:FQ=1:SQ=-1;ID=S2663_raw:GT=hom:DP=41:FQ=1:SQ=-1;ID=S2011_raw:GT=hom:DP=69:FQ=1:SQ=-1;ID=S2504_raw:GT=hom:DP=39:FQ=1:SQ=-1;ID=S2008_raw:GT=hom:DP=44:FQ=1:SQ=-1;ID=S2509_raw:GT=hom:DP=37:FQ=1:SQ=-1;ID=S2508_raw:GT=hom:DP=62:FQ=1:SQ=-1;ID=S2666_raw:GT=hom:DP=87:FQ=1:SQ=-1;ID=S2669_raw:GT=hom:DP=9:FQ=1:SQ=-1;ID=S2166_raw:GT=hom:DP=34:FQ=1:SQ=-1;ID=S1775_raw:GT=hom:DP=25:FQ=1:SQ=-1;ID=S2501_raw:GT=hom:DP=52:FQ=1:SQ=-1;ID=S2500_raw:GT=hom:DP=51:FQ=1:SQ=-1;ID=S2502_raw:GT=hom:DP=56:FQ=1:SQ=-1;ID=S2505_raw:GT=hom:DP=29:FQ=1:SQ=-1;ID=S2176_raw:GT=hom:DP=59:FQ=1:SQ=-1;ID=S2506_raw:GT=hom:DP=35:FQ=1:SQ=-1;ID=S2667_raw:GT=hom:DP=62:FQ=1:SQ=-1;
11	66278059	66278059	C	G	upstream;downstream	BBS1;DPP3	.	dist=47;dist=929	rs1791688	.	ID=S2009_raw:GT=hom:DP=16:FQ=1:SQ=-1;ID=S2503_raw:GT=hom:DP=35:FQ=1:SQ=-1;ID=S2507_raw:GT=hom:DP=24:FQ=1:SQ=-1;ID=S2668_raw:GT=hom:DP=43:FQ=1:SQ=-1;ID=S2012_raw:GT=hom:DP=20:FQ=1:SQ=-1;ID=S2010_raw:GT=hom:DP=18:FQ=1:SQ=-1;ID=S2662_raw:GT=hom:DP=55:FQ=1:SQ=-1;ID=S2663_raw:GT=hom:DP=36:FQ=1:SQ=-1;ID=S2011_raw:GT=hom:DP=26:FQ=1:SQ=-1;ID=S2504_raw:GT=hom:DP=34:FQ=1:SQ=-1;ID=S2008_raw:GT=hom:DP=15:FQ=1:SQ=-1;ID=S2509_raw:GT=hom:DP=25:FQ=1:SQ=-1;ID=S2508_raw:GT=hom:DP=47:FQ=1:SQ=-1;ID=S2666_raw:GT=hom:DP=14:FQ=1:SQ=-1;ID=S2669_raw:GT=hom:DP=40:FQ=1:SQ=-1;ID=S2166_raw:GT=hom:DP=42:FQ=1:SQ=-1;ID=S1775_raw:GT=hom:DP=28:FQ=1:SQ=-1;ID=S2501_raw:GT=hom:DP=34:FQ=1:SQ=-1;ID=S2500_raw:GT=hom:DP=35:FQ=1:SQ=-1;ID=S2502_raw:GT=hom:DP=46:FQ=1:SQ=-1;ID=S2505_raw:GT=hom:DP=26:FQ=1:SQ=-1;ID=S2176_raw:GT=hom:DP=32:FQ=1:SQ=-1;ID=S2506_raw:GT=hom:DP=39:FQ=1:SQ=-1;ID=S2667_raw:GT=hom:DP=45:FQ=1:SQ=-1;
15	72987588	72987588	G	T	intronic	BBS4	.	.	rs4777527	Benign	ID=S2009_raw:GT=hom:DP=52:FQ=1:SQ=-1;ID=S2503_raw:GT=hom:DP=108:FQ=1:SQ=-1;ID=S2507_raw:GT=hom:DP=59:FQ=1:SQ=-1;ID=S2668_raw:GT=hom:DP=129:FQ=1:SQ=-1;ID=S2012_raw:GT=hom:DP=56:FQ=1:SQ=-1;ID=S2010_raw:GT=hom:DP=80:FQ=1:SQ=-1;ID=S2662_raw:GT=hom:DP=21:FQ=1:SQ=-1;ID=S2663_raw:GT=hom:DP=74:FQ=1:SQ=-1;ID=S2011_raw:GT=hom:DP=92:FQ=1:SQ=-1;ID=S2504_raw:GT=hom:DP=100:FQ=1:SQ=-1;ID=S2008_raw:GT=hom:DP=66:FQ=1:SQ=-1;ID=S2509_raw:GT=hom:DP=90:FQ=1:SQ=-1;ID=S2508_raw:GT=hom:DP=86:FQ=1:SQ=-1;ID=S2666_raw:GT=hom:DP=180:FQ=1:SQ=-1;ID=S2669_raw:GT=hom:DP=44:FQ=1:SQ=-1;ID=S2166_raw:GT=hom:DP=54:FQ=1:SQ=-1;ID=S1775_raw:GT=hom:DP=16:FQ=1:SQ=-1;ID=S2501_raw:GT=hom:DP=79:FQ=1:SQ=-1;ID=S2500_raw:GT=hom:DP=71:FQ=1:SQ=-1;ID=S2502_raw:GT=hom:DP=78:FQ=1:SQ=-1;ID=S2505_raw:GT=hom:DP=82:FQ=1:SQ=-1;ID=S2176_raw:GT=hom:DP=68:FQ=1:SQ=-1;ID=S2506_raw:GT=hom:DP=117:FQ=1:SQ=-1;ID=S2667_raw:GT=hom:DP=106:FQ=1:SQ=-1;
15	73023937	73023937	T	C	exonic	BBS4	synonymous SNV	BBS4:NM_001252678:exon11:c.390T>C:p.F130F,BBS4:NM_001320665:exon11:c.837T>C:p.F279F,BBS4:NM_033028:exon12:c.906T>C:p.F302F	rs12914333	Benign	ID=S2009_raw:GT=hom:DP=122:FQ=1:SQ=-1;ID=S2503_raw:GT=hom:DP=136:FQ=1:SQ=-1;ID=S2507_raw:GT=hom:DP=145:FQ=1:SQ=-1;ID=S2668_raw:GT=hom:DP=140:FQ=1:SQ=-1;ID=S2012_raw:GT=hom:DP=142:FQ=1:SQ=-1;ID=S2010_raw:GT=hom:DP=142:FQ=1:SQ=-1;ID=S2662_raw:GT=hom:DP=61:FQ=1:SQ=-1;ID=S2663_raw:GT=hom:DP=118:FQ=1:SQ=-1;ID=S2011_raw:GT=hom:DP=219:FQ=1:SQ=-1;ID=S2504_raw:GT=hom:DP=109:FQ=1:SQ=-1;ID=S2008_raw:GT=hom:DP=188:FQ=1:SQ=-1;ID=S2509_raw:GT=hom:DP=114:FQ=1:SQ=-1;ID=S2508_raw:GT=hom:DP=134:FQ=1:SQ=-1;ID=S2666_raw:GT=hom:DP=116:FQ=1:SQ=-1;ID=S2669_raw:GT=hom:DP=109:FQ=0.99:SQ=-1;ID=S2166_raw:GT=hom:DP=327:FQ=1:SQ=-1;ID=S1775_raw:GT=hom:DP=177:FQ=1:SQ=-1;ID=S2501_raw:GT=hom:DP=125:FQ=1:SQ=-1;ID=S2500_raw:GT=hom:DP=105:FQ=1:SQ=-1;ID=S2502_raw:GT=hom:DP=147:FQ=1:SQ=-1;ID=S2505_raw:GT=hom:DP=133:FQ=1:SQ=-1;ID=S2176_raw:GT=hom:DP=336:FQ=1:SQ=-1;ID=S2506_raw:GT=hom:DP=119:FQ=1:SQ=-1;ID=S2667_raw:GT=hom:DP=101:FQ=1:SQ=-1;
15	73024181	73024181	G	A	intronic	BBS4	.	.	rs9806635	.	ID=S2009_raw:GT=hom:DP=4:FQ=1:SQ=-1;ID=S2503_raw:GT=hom:DP=40:FQ=1:SQ=-1;ID=S2507_raw:GT=hom:DP=40:FQ=1:SQ=-1;ID=S2668_raw:GT=hom:DP=57:FQ=1:SQ=-1;ID=S2012_raw:GT=hom:DP=7:FQ=1:SQ=-1;ID=S2010_raw:GT=hom:DP=7:FQ=1:SQ=-1;ID=S2662_raw:GT=hom:DP=2:FQ=1:SQ=-1;ID=S2663_raw:GT=hom:DP=60:FQ=1:SQ=-1;ID=S2011_raw:GT=hom:DP=11:FQ=1:SQ=-1;ID=S2504_raw:GT=hom:DP=49:FQ=1:SQ=-1;ID=S2008_raw:GT=hom:DP=11:FQ=1:SQ=-1;ID=S2509_raw:GT=hom:DP=49:FQ=1:SQ=-1;ID=S2508_raw:GT=hom:DP=35:FQ=1:SQ=-1;ID=S2666_raw:GT=hom:DP=40:FQ=1:SQ=-1;ID=S2669_raw:GT=hom:DP=35:FQ=1:SQ=-1;ID=S2166_raw:GT=hom:DP=41:FQ=1:SQ=-1;ID=S1775_raw:GT=hom:DP=30:FQ=1:SQ=-1;ID=S2501_raw:GT=hom:DP=51:FQ=1:SQ=-1;ID=S2500_raw:GT=hom:DP=68:FQ=1:SQ=-1;ID=S2502_raw:GT=hom:DP=51:FQ=1:SQ=-1;ID=S2505_raw:GT=hom:DP=31:FQ=1:SQ=-1;ID=S2176_raw:GT=hom:DP=41:FQ=1:SQ=-1;ID=S2506_raw:GT=hom:DP=33:FQ=1:SQ=-1;ID=S2667_raw:GT=hom:DP=41:FQ=1:SQ=-1;
15	73028375	73028375	G	A	intronic	BBS4	.	.	rs12324395	.	ID=S2009_raw:GT=hom:DP=63:FQ=1:SQ=-1;ID=S2503_raw:GT=hom:DP=43:FQ=1:SQ=-1;ID=S2507_raw:GT=hom:DP=38:FQ=1:SQ=-1;ID=S2668_raw:GT=hom:DP=61:FQ=1:SQ=-1;ID=S2012_raw:GT=hom:DP=58:FQ=1:SQ=-1;ID=S2010_raw:GT=hom:DP=71:FQ=1:SQ=-1;ID=S2662_raw:GT=hom:DP=6:FQ=1:SQ=-1;ID=S2663_raw:GT=hom:DP=40:FQ=1:SQ=-1;ID=S2011_raw:GT=hom:DP=80:FQ=1:SQ=-1;ID=S2504_raw:GT=hom:DP=28:FQ=1:SQ=-1;ID=S2008_raw:GT=hom:DP=64:FQ=1:SQ=-1;ID=S2509_raw:GT=hom:DP=48:FQ=1:SQ=-1;ID=S2508_raw:GT=hom:DP=40:FQ=1:SQ=-1;ID=S2666_raw:GT=hom:DP=45:FQ=1:SQ=-1;ID=S2669_raw:GT=hom:DP=45:FQ=1:SQ=-1;ID=S2166_raw:GT=hom:DP=17:FQ=1:SQ=-1;ID=S1775_raw:GT=hom:DP=20:FQ=1:SQ=-1;ID=S2501_raw:GT=hom:DP=48:FQ=1:SQ=-1;ID=S2500_raw:GT=hom:DP=47:FQ=1:SQ=-1;ID=S2502_raw:GT=hom:DP=34:FQ=1:SQ=-1;ID=S2505_raw:GT=hom:DP=33:FQ=1:SQ=-1;ID=S2176_raw:GT=hom:DP=30:FQ=1:SQ=-1;ID=S2506_raw:GT=hom:DP=37:FQ=1:SQ=-1;ID=S2667_raw:GT=hom:DP=41:FQ=1:SQ=-1;
7	33569197	33569197	A	G	intronic	BBS9	.	.	rs1882040	.	ID=S2009_raw:GT=hom:DP=1:FQ=1:SQ=-1;ID=S2503_raw:GT=hom:DP=11:FQ=1:SQ=-1;ID=S2507_raw:GT=hom:DP=10:FQ=1:SQ=-1;ID=S2668_raw:GT=hom:DP=12:FQ=1:SQ=-1;ID=S2012_raw:GT=hom:DP=2:FQ=1:SQ=-1;ID=S2010_raw:GT=hom:DP=3:FQ=1:SQ=-1;ID=S2662_raw:GT=hom:DP=9:FQ=1:SQ=-1;ID=S2663_raw:GT=hom:DP=17:FQ=1:SQ=-1;ID=S2011_raw:GT=hom:DP=3:FQ=1:SQ=-1;ID=S2504_raw:GT=hom:DP=8:FQ=1:SQ=-1;ID=S2008_raw:GT=hom:DP=1:FQ=1:SQ=-1;ID=S2509_raw:GT=hom:DP=16:FQ=1:SQ=-1;ID=S2508_raw:GT=hom:DP=6:FQ=1:SQ=-1;ID=S2666_raw:GT=hom:DP=6:FQ=1:SQ=-1;ID=S2669_raw:GT=hom:DP=11:FQ=1:SQ=-1;ID=S2166_raw:GT=hom:DP=13:FQ=1:SQ=-1;ID=S1775_raw:GT=hom:DP=7:FQ=1:SQ=-1;ID=S2501_raw:GT=hom:DP=9:FQ=1:SQ=-1;ID=S2500_raw:GT=hom:DP=14:FQ=1:SQ=-1;ID=S2502_raw:GT=hom:DP=11:FQ=1:SQ=-1;ID=S2505_raw:GT=hom:DP=10:FQ=1:SQ=-1;ID=S2176_raw:GT=hom:DP=8:FQ=1:SQ=-1;ID=S2506_raw:GT=hom:DP=8:FQ=1:SQ=-1;ID=S2667_raw:GT=hom:DP=12:FQ=1:SQ=-1;
2	2,2E+08	2,2E+08	A	G	intronic	BCS1L	.	.	rs6722185	.	ID=S2009_raw:GT=hom:DP=101:FQ=1:SQ=-1;ID=S2503_raw:GT=hom:DP=57:FQ=1:SQ=-1;ID=S2507_raw:GT=hom:DP=47:FQ=1:SQ=-1;ID=S2668_raw:GT=hom:DP=94:FQ=1:SQ=-1;ID=S2012_raw:GT=hom:DP=115:FQ=1:SQ=-1;ID=S2010_raw:GT=hom:DP=110:FQ=1:SQ=-1;ID=S2662_raw:GT=hom:DP=78:FQ=1:SQ=-1;ID=S2663_raw:GT=hom:DP=53:FQ=1:SQ=-1;ID=S2011_raw:GT=hom:DP=155:FQ=1:SQ=-1;ID=S2504_raw:GT=hom:DP=68:FQ=1:SQ=-1;ID=S2008_raw:GT=hom:DP=113:FQ=1:SQ=-1;ID=S2509_raw:GT=hom:DP=58:FQ=1:SQ=-1;ID=S2508_raw:GT=hom:DP=56:FQ=1:SQ=-1;ID=S2666_raw:GT=hom:DP=92:FQ=1:SQ=-1;ID=S2669_raw:GT=hom:DP=50:FQ=1:SQ=-1;ID=S2166_raw:GT=hom:DP=23:FQ=1:SQ=-1;ID=S1775_raw:GT=hom:DP=7:FQ=1:SQ=-1;ID=S2501_raw:GT=hom:DP=56:FQ=1:SQ=-1;ID=S2500_raw:GT=hom:DP=72:FQ=1:SQ=-1;ID=S2502_raw:GT=hom:DP=73:FQ=1:SQ=-1;ID=S2505_raw:GT=hom:DP=71:FQ=1:SQ=-1;ID=S2176_raw:GT=hom:DP=13:FQ=1:SQ=-1;ID=S2506_raw:GT=hom:DP=46:FQ=1:SQ=-1;ID=S2667_raw:GT=hom:DP=59:FQ=1:SQ=-1;
10	60396306	60396306	G	C	intronic	BICC1	.	.	rs920258	.	ID=S2009_raw:GT=hom:DP=2:FQ=1:SQ=-1;ID=S2503_raw:GT=hom:DP=14:FQ=1:SQ=-1;ID=S2507_raw:GT=hom:DP=13:FQ=1:SQ=-1;ID=S2668_raw:GT=hom:DP=38:FQ=1:SQ=-1;ID=S2012_raw:GT=hom:DP=3:FQ=1:SQ=-1;ID=S2010_raw:GT=hom:DP=2:FQ=1:SQ=-1;ID=S2662_raw:GT=hom:DP=4:FQ=1:SQ=-1;ID=S2663_raw:GT=hom:DP=22:FQ=1:SQ=-1;ID=S2011_raw:GT=hom:DP=6:FQ=1:SQ=-1;ID=S2504_raw:GT=hom:DP=12:FQ=1:SQ=-1;ID=S2008_raw:GT=hom:DP=4:FQ=1:SQ=-1;ID=S2509_raw:GT=hom:DP=14:FQ=1:SQ=-1;ID=S2508_raw:GT=hom:DP=22:FQ=1:SQ=-1;ID=S2666_raw:GT=hom:DP=22:FQ=1:SQ=-1;ID=S2669_raw:GT=hom:DP=27:FQ=1:SQ=-1;ID=S2166_raw:GT=hom:DP=13:FQ=1:SQ=-1;ID=S1775_raw:GT=hom:DP=4:FQ=1:SQ=-1;ID=S2501_raw:GT=hom:DP=23:FQ=1:SQ=-1;ID=S2500_raw:GT=hom:DP=13:FQ=1:SQ=-1;ID=S2502_raw:GT=hom:DP=23:FQ=1:SQ=-1;ID=S2505_raw:GT=hom:DP=23:FQ=1:SQ=-1;ID=S2176_raw:GT=hom:DP=12:FQ=1:SQ=-1;ID=S2506_raw:GT=hom:DP=13:FQ=1:SQ=-1;ID=S2667_raw:GT=hom:DP=21:FQ=1:SQ=-1;
8	11416122	11416122	T	C	intronic	BLK	.	.	rs2467518	.	ID=S2009_raw:GT=hom:DP=62:FQ=1:SQ=-1;ID=S2503_raw:GT=hom:DP=62:FQ=1:SQ=-1;ID=S2507_raw:GT=hom:DP=40:FQ=1:SQ=-1;ID=S2668_raw:GT=hom:DP=40:FQ=1:SQ=-1;ID=S2012_raw:GT=hom:DP=62:FQ=1:SQ=-1;ID=S2010_raw:GT=hom:DP=64:FQ=1:SQ=-1;ID=S2662_raw:GT=hom:DP=65:FQ=1:SQ=-1;ID=S2663_raw:GT=hom:DP=44:FQ=1:SQ=-1;ID=S2011_raw:GT=hom:DP=111:FQ=1:SQ=-1;ID=S2504_raw:GT=hom:DP=37:FQ=1:SQ=-1;ID=S2008_raw:GT=hom:DP=82:FQ=1:SQ=-1;ID=S2509_raw:GT=hom:DP=52:FQ=1:SQ=-1;ID=S2508_raw:GT=hom:DP=45:FQ=1:SQ=-1;ID=S2666_raw:GT=hom:DP=25:FQ=1:SQ=-1;ID=S2669_raw:GT=hom:DP=27:FQ=1:SQ=-1;ID=S2166_raw:GT=hom:DP=33:FQ=1:SQ=-1;ID=S1775_raw:GT=hom:DP=15:FQ=1:SQ=-1;ID=S2501_raw:GT=hom:DP=48:FQ=1:SQ=-1;ID=S2500_raw:GT=hom:DP=34:FQ=1:SQ=-1;ID=S2502_raw:GT=hom:DP=39:FQ=1:SQ=-1;ID=S2505_raw:GT=hom:DP=48:FQ=1:SQ=-1;ID=S2176_raw:GT=hom:DP=26:FQ=1:SQ=-1;ID=S2506_raw:GT=hom:DP=68:FQ=1:SQ=-1;ID=S2667_raw:GT=hom:DP=50:FQ=1:SQ=-1;
13	32913055	32913055	A	G	exonic	BRCA2	synonymous SNV	BRCA2:NM_000059:exon11:c.4563A>G:p.L1521L	rs206075	Benign	ID=S2009_raw:GT=hom:DP=112:FQ=1:SQ=-1;ID=S2503_raw:GT=hom:DP=66:FQ=1:SQ=-1;ID=S2507_raw:GT=hom:DP=63:FQ=1:SQ=-1;ID=S2668_raw:GT=hom:DP=78:FQ=1:SQ=-1;ID=S2012_raw:GT=hom:DP=97:FQ=1:SQ=-1;ID=S2010_raw:GT=hom:DP=106:FQ=1:SQ=-1;ID=S2662_raw:GT=hom:DP=10:FQ=1:SQ=-1;ID=S2663_raw:GT=hom:DP=63:FQ=1:SQ=-1;ID=S2011_raw:GT=hom:DP=157:FQ=1:SQ=-1;ID=S2504_raw:GT=hom:DP=69:FQ=1:SQ=-1;ID=S2008_raw:GT=hom:DP=108:FQ=1:SQ=-1;ID=S2509_raw:GT=hom:DP=78:FQ=1:SQ=-1;ID=S2508_raw:GT=hom:DP=82:FQ=1:SQ=-1;ID=S2666_raw:GT=hom:DP=92:FQ=1:SQ=-1;ID=S2669_raw:GT=hom:DP=47:FQ=1:SQ=-1;ID=S2166_raw:GT=hom:DP=196:FQ=1:SQ=-1;ID=S1775_raw:GT=hom:DP=144:FQ=1:SQ=-1;ID=S2501_raw:GT=hom:DP=73:FQ=1:SQ=-1;ID=S2500_raw:GT=hom:DP=89:FQ=1:SQ=-1;ID=S2502_raw:GT=hom:DP=75:FQ=1:SQ=-1;ID=S2505_raw:GT=hom:DP=70:FQ=1:SQ=-1;ID=S2176_raw:GT=hom:DP=219:FQ=1:SQ=-1;ID=S2506_raw:GT=hom:DP=59:FQ=1:SQ=-1;ID=S2667_raw:GT=hom:DP=58:FQ=1:SQ=-1;
13	32915005	32915005	G	C	exonic	BRCA2	synonymous SNV	BRCA2:NM_000059:exon11:c.6513G>C:p.V2171V	rs206076	Benign	ID=S2009_raw:GT=hom:DP=94:FQ=1:SQ=-1;ID=S2503_raw:GT=hom:DP=102:FQ=1:SQ=-1;ID=S2507_raw:GT=hom:DP=61:FQ=1:SQ=-1;ID=S2668_raw:GT=hom:DP=118:FQ=1:SQ=-1;ID=S2012_raw:GT=hom:DP=131:FQ=1:SQ=-1;ID=S2010_raw:GT=hom:DP=111:FQ=1:SQ=-1;ID=S2662_raw:GT=hom:DP=1:FQ=1:SQ=-1;ID=S2663_raw:GT=hom:DP=91:FQ=1:SQ=-1;ID=S2011_raw:GT=hom:DP=184:FQ=1:SQ=-1;ID=S2504_raw:GT=hom:DP=71:FQ=1:SQ=-1;ID=S2008_raw:GT=hom:DP=113:FQ=1:SQ=-1;ID=S2509_raw:GT=hom:DP=65:FQ=1:SQ=-1;ID=S2508_raw:GT=hom:DP=86:FQ=1:SQ=-1;ID=S2666_raw:GT=hom:DP=67:FQ=1:SQ=-1;ID=S2669_raw:GT=hom:DP=42:FQ=1:SQ=-1;ID=S2166_raw:GT=hom:DP=68:FQ=1:SQ=-1;ID=S1775_raw:GT=hom:DP=54:FQ=1:SQ=-1;ID=S2501_raw:GT=hom:DP=68:FQ=1:SQ=-1;ID=S2500_raw:GT=hom:DP=85:FQ=1:SQ=-1;ID=S2502_raw:GT=hom:DP=88:FQ=1:SQ=-1;ID=S2505_raw:GT=hom:DP=59:FQ=1:SQ=-1;ID=S2176_raw:GT=hom:DP=87:FQ=1:SQ=-1;ID=S2506_raw:GT=hom:DP=43:FQ=1:SQ=-1;ID=S2667_raw:GT=hom:DP=70:FQ=1:SQ=-1;
13	32929387	32929387	T	C	exonic	BRCA2	nonsynonymous SNV	BRCA2:NM_000059:exon14:c.7397T>C:p.V2466A	rs169547	Benign	ID=S2009_raw:GT=hom:DP=20:FQ=1:SQ=-1;ID=S2503_raw:GT=hom:DP=93:FQ=1:SQ=-1;ID=S2507_raw:GT=hom:DP=92:FQ=1:SQ=-1;ID=S2668_raw:GT=hom:DP=129:FQ=1:SQ=-1;ID=S2012_raw:GT=hom:DP=21:FQ=1:SQ=-1;ID=S2010_raw:GT=hom:DP=27:FQ=1:SQ=-1;ID=S2662_raw:GT=hom:DP=7:FQ=1:SQ=-1;ID=S2663_raw:GT=hom:DP=75:FQ=1:SQ=-1;ID=S2011_raw:GT=hom:DP=38:FQ=1:SQ=-1;ID=S2504_raw:GT=hom:DP=87:FQ=1:SQ=-1;ID=S2008_raw:GT=hom:DP=24:FQ=1:SQ=-1;ID=S2509_raw:GT=hom:DP=74:FQ=1:SQ=-1;ID=S2508_raw:GT=hom:DP=90:FQ=1:SQ=-1;ID=S2666_raw:GT=hom:DP=140:FQ=1:SQ=-1;ID=S2669_raw:GT=hom:DP=64:FQ=1:SQ=-1;ID=S2166_raw:GT=hom:DP=81:FQ=1:SQ=-1;ID=S1775_raw:GT=hom:DP=57:FQ=1:SQ=-1;ID=S2501_raw:GT=hom:DP=87:FQ=1:SQ=-1;ID=S2500_raw:GT=hom:DP=110:FQ=1:SQ=-1;ID=S2502_raw:GT=hom:DP=76:FQ=1:SQ=-1;ID=S2505_raw:GT=hom:DP=84:FQ=1:SQ=-1;ID=S2176_raw:GT=hom:DP=104:FQ=1:SQ=-1;ID=S2506_raw:GT=hom:DP=78:FQ=1:SQ=-1;ID=S2667_raw:GT=hom:DP=71:FQ=1:SQ=-1;
17	59876788	59876789	GA	-	intronic	BRIP1	.	.	.	.	ID=S2009_raw:GT=hom:DP=1:FQ=1:SQ=-1;ID=S2503_raw:GT=hom:DP=3:FQ=1:SQ=-1;ID=S2507_raw:GT=hom:DP=2:FQ=1:SQ=-1;ID=S2668_raw:GT=hom:DP=4:FQ=1:SQ=-1;ID=S2012_raw:GT=hom:DP=3:FQ=1:SQ=-1;ID=S2010_raw:GT=hom:DP=2:FQ=1:SQ=-1;ID=S2662_raw:GT=hom:DP=1:FQ=1:SQ=-1;ID=S2663_raw:GT=hom:DP=1:FQ=1:SQ=-1;ID=S2011_raw:GT=hom:DP=2:FQ=1:SQ=-1;ID=S2504_raw:GT=hom:DP=1:FQ=1:SQ=-1;ID=S2008_raw:GT=hom:DP=1:FQ=1:SQ=-1;ID=S2509_raw:GT=hom:DP=1:FQ=1:SQ=-1;ID=S2508_raw:GT=hom:DP=2:FQ=1:SQ=-1;ID=S2666_raw:GT=hom:DP=1:FQ=1:SQ=-1;ID=S2669_raw:GT=hom:DP=1:FQ=1:SQ=-1;ID=S2166_raw:GT=hom:DP=17:FQ=1:SQ=-1;ID=S1775_raw:GT=hom:DP=8:FQ=1:SQ=-1;ID=S2501_raw:GT=hom:DP=1:FQ=1:SQ=-1;ID=S2500_raw:GT=hom:DP=3:FQ=1:SQ=-1;ID=S2502_raw:GT=hom:DP=2:FQ=1:SQ=-1;ID=S2505_raw:GT=hom:DP=3:FQ=1:SQ=-1;ID=S2176_raw:GT=hom:DP=18:FQ=1:SQ=-1;ID=S2506_raw:GT=hom:DP=5:FQ=1:SQ=-1;ID=S2667_raw:GT=hom:DP=2:FQ=1:SQ=-1;
2	2,02E+08	2,02E+08	-	C	upstream;downstream	C2CD6;TMEM237	.	dist=99;dist=903	.	.	ID=S2009_raw:GT=hom:DP=6:FQ=1:SQ=-1;ID=S2503_raw:GT=hom:DP=15:FQ=1:SQ=-1;ID=S2507_raw:GT=hom:DP=14:FQ=1:SQ=-1;ID=S2668_raw:GT=hom:DP=4:FQ=1:SQ=-1;ID=S2012_raw:GT=hom:DP=11:FQ=1:SQ=-1;ID=S2010_raw:GT=hom:DP=8:FQ=1:SQ=-1;ID=S2662_raw:GT=hom:DP=8:FQ=1:SQ=-1;ID=S2663_raw:GT=hom:DP=8:FQ=1:SQ=-1;ID=S2011_raw:GT=hom:DP=19:FQ=1:SQ=-1;ID=S2504_raw:GT=hom:DP=14:FQ=1:SQ=-1;ID=S2008_raw:GT=hom:DP=16:FQ=1:SQ=-1;ID=S2509_raw:GT=hom:DP=11:FQ=1:SQ=-1;ID=S2508_raw:GT=hom:DP=11:FQ=1:SQ=-1;ID=S2666_raw:GT=hom:DP=9:FQ=1:SQ=-1;ID=S2669_raw:GT=hom:DP=8:FQ=1:SQ=-1;ID=S2166_raw:GT=hom:DP=15:FQ=1:SQ=-1;ID=S1775_raw:GT=hom:DP=4:FQ=1:SQ=-1;ID=S2501_raw:GT=hom:DP=9:FQ=1:SQ=-1;ID=S2500_raw:GT=hom:DP=18:FQ=1:SQ=-1;ID=S2502_raw:GT=hom:DP=7:FQ=1:SQ=-1;ID=S2505_raw:GT=hom:DP=11:FQ=1:SQ=-1;ID=S2176_raw:GT=hom:DP=16:FQ=1:SQ=-1;ID=S2506_raw:GT=hom:DP=13:FQ=1:SQ=-1;ID=S2667_raw:GT=hom:DP=7:FQ=1:SQ=-1;
1	2,01E+08	2,01E+08	T	C	intronic	CACNA1S	.	.	rs6427875	.	ID=S2009_raw:GT=hom:DP=11:FQ=1:SQ=-1;ID=S2503_raw:GT=hom:DP=33:FQ=1:SQ=-1;ID=S2507_raw:GT=hom:DP=40:FQ=1:SQ=-1;ID=S2668_raw:GT=hom:DP=40:FQ=1:SQ=-1;ID=S2012_raw:GT=hom:DP=8:FQ=1:SQ=-1;ID=S2010_raw:GT=hom:DP=8:FQ=1:SQ=-1;ID=S2662_raw:GT=hom:DP=38:FQ=1:SQ=-1;ID=S2663_raw:GT=hom:DP=31:FQ=1:SQ=-1;ID=S2011_raw:GT=hom:DP=20:FQ=1:SQ=-1;ID=S2504_raw:GT=hom:DP=33:FQ=1:SQ=-1;ID=S2008_raw:GT=hom:DP=15:FQ=1:SQ=-1;ID=S2509_raw:GT=hom:DP=26:FQ=1:SQ=-1;ID=S2508_raw:GT=hom:DP=47:FQ=1:SQ=-1;ID=S2666_raw:GT=hom:DP=23:FQ=1:SQ=-1;ID=S2669_raw:GT=hom:DP=39:FQ=1:SQ=-1;ID=S2166_raw:GT=hom:DP=42:FQ=1:SQ=-1;ID=S1775_raw:GT=hom:DP=10:FQ=1:SQ=-1;ID=S2501_raw:GT=hom:DP=31:FQ=1:SQ=-1;ID=S2500_raw:GT=hom:DP=22:FQ=1:SQ=-1;ID=S2502_raw:GT=hom:DP=36:FQ=1:SQ=-1;ID=S2505_raw:GT=hom:DP=33:FQ=1:SQ=-1;ID=S2176_raw:GT=hom:DP=29:FQ=1:SQ=-1;ID=S2506_raw:GT=hom:DP=18:FQ=1:SQ=-1;ID=S2667_raw:GT=hom:DP=37:FQ=1:SQ=-1;
2	27440785	27440785	C	T	exonic	CAD	synonymous SNV	CAD:NM_001306079:exon2:c.123C>T:p.T41T,CAD:NM_004341:exon2:c.123C>T:p.T41T	rs1275536	.	ID=S2009_raw:GT=hom:DP=132:FQ=1:SQ=-1;ID=S2503_raw:GT=hom:DP=176:FQ=1:SQ=-1;ID=S2507_raw:GT=hom:DP=223:FQ=1:SQ=-1;ID=S2668_raw:GT=hom:DP=221:FQ=1:SQ=-1;ID=S2012_raw:GT=hom:DP=151:FQ=1:SQ=-1;ID=S2010_raw:GT=hom:DP=162:FQ=1:SQ=-1;ID=S2662_raw:GT=hom:DP=238:FQ=1:SQ=-1;ID=S2663_raw:GT=hom:DP=181:FQ=1:SQ=-1;ID=S2011_raw:GT=hom:DP=215:FQ=1:SQ=-1;ID=S2504_raw:GT=hom:DP=220:FQ=1:SQ=-1;ID=S2008_raw:GT=hom:DP=165:FQ=1:SQ=-1;ID=S2509_raw:GT=hom:DP=228:FQ=1:SQ=-1;ID=S2508_raw:GT=hom:DP=188:FQ=1:SQ=-1;ID=S2666_raw:GT=hom:DP=169:FQ=1:SQ=-1;ID=S2669_raw:GT=hom:DP=199:FQ=1:SQ=-1;ID=S2166_raw:GT=hom:DP=271:FQ=1:SQ=-1;ID=S1775_raw:GT=hom:DP=183:FQ=1:SQ=-1;ID=S2501_raw:GT=hom:DP=174:FQ=1:SQ=-1;ID=S2500_raw:GT=hom:DP=192:FQ=1:SQ=-1;ID=S2502_raw:GT=hom:DP=217:FQ=1:SQ=-1;ID=S2505_raw:GT=hom:DP=193:FQ=1:SQ=-1;ID=S2176_raw:GT=hom:DP=289:FQ=1:SQ=-1;ID=S2506_raw:GT=hom:DP=195:FQ=1:SQ=-1;ID=S2667_raw:GT=hom:DP=187:FQ=1:SQ=-1;
16	602046	602046	T	C	splicing	CAPN15	.	NM_005632:exon10:c.2346-5T>C	rs7196821	.	ID=S2009_raw:GT=hom:DP=42:FQ=1:SQ=-1;ID=S2503_raw:GT=hom:DP=50:FQ=1:SQ=-1;ID=S2507_raw:GT=hom:DP=54:FQ=1:SQ=-1;ID=S2668_raw:GT=hom:DP=60:FQ=1:SQ=-1;ID=S2012_raw:GT=hom:DP=50:FQ=1:SQ=-1;ID=S2010_raw:GT=hom:DP=44:FQ=1:SQ=-1;ID=S2662_raw:GT=hom:DP=103:FQ=1:SQ=-1;ID=S2663_raw:GT=hom:DP=41:FQ=1:SQ=-1;ID=S2011_raw:GT=hom:DP=63:FQ=1:SQ=-1;ID=S2504_raw:GT=hom:DP=55:FQ=1:SQ=-1;ID=S2008_raw:GT=hom:DP=71:FQ=1:SQ=-1;ID=S2509_raw:GT=hom:DP=52:FQ=1:SQ=-1;ID=S2508_raw:GT=hom:DP=69:FQ=1:SQ=-1;ID=S2666_raw:GT=hom:DP=47:FQ=1:SQ=-1;ID=S2669_raw:GT=hom:DP=47:FQ=1:SQ=-1;ID=S2166_raw:GT=hom:DP=19:FQ=1:SQ=-1;ID=S1775_raw:GT=hom:DP=17:FQ=1:SQ=-1;ID=S2501_raw:GT=hom:DP=47:FQ=1:SQ=-1;ID=S2500_raw:GT=hom:DP=57:FQ=1:SQ=-1;ID=S2502_raw:GT=hom:DP=73:FQ=1:SQ=-1;ID=S2505_raw:GT=hom:DP=48:FQ=1:SQ=-1;ID=S2176_raw:GT=hom:DP=15:FQ=1:SQ=-1;ID=S2506_raw:GT=hom:DP=84:FQ=1:SQ=-1;ID=S2667_raw:GT=hom:DP=76:FQ=1:SQ=-1;
3	1,22E+08	1,22E+08	G	A	intronic	CASR	.	.	rs9869985	Benign	ID=S2009_raw:GT=hom:DP=38:FQ=1:SQ=-1;ID=S2503_raw:GT=hom:DP=19:FQ=1:SQ=-1;ID=S2507_raw:GT=hom:DP=16:FQ=1:SQ=-1;ID=S2668_raw:GT=hom:DP=10:FQ=1:SQ=-1;ID=S2012_raw:GT=hom:DP=35:FQ=1:SQ=-1;ID=S2010_raw:GT=hom:DP=31:FQ=1:SQ=-1;ID=S2662_raw:GT=hom:DP=16:FQ=1:SQ=-1;ID=S2663_raw:GT=hom:DP=26:FQ=1:SQ=-1;ID=S2011_raw:GT=hom:DP=46:FQ=1:SQ=-1;ID=S2504_raw:GT=hom:DP=27:FQ=1:SQ=-1;ID=S2008_raw:GT=hom:DP=29:FQ=1:SQ=-1;ID=S2509_raw:GT=hom:DP=19:FQ=1:SQ=-1;ID=S2508_raw:GT=hom:DP=18:FQ=1:SQ=-1;ID=S2666_raw:GT=hom:DP=12:FQ=1:SQ=-1;ID=S2669_raw:GT=hom:DP=13:FQ=1:SQ=-1;ID=S2166_raw:GT=hom:DP=38:FQ=1:SQ=-1;ID=S1775_raw:GT=hom:DP=9:FQ=1:SQ=-1;ID=S2501_raw:GT=hom:DP=22:FQ=1:SQ=-1;ID=S2500_raw:GT=hom:DP=19:FQ=1:SQ=-1;ID=S2502_raw:GT=hom:DP=27:FQ=1:SQ=-1;ID=S2505_raw:GT=hom:DP=24:FQ=1:SQ=-1;ID=S2176_raw:GT=hom:DP=41:FQ=1:SQ=-1;ID=S2506_raw:GT=hom:DP=19:FQ=1:SQ=-1;ID=S2667_raw:GT=hom:DP=28:FQ=1:SQ=-1;
3	1,22E+08	1,22E+08	G	C	exonic	CASR	synonymous SNV	CASR:NM_000388:exon7:c.2244G>C:p.P748P,CASR:NM_001178065:exon7:c.2274G>C:p.P758P	rs2036400	Benign	ID=S2009_raw:GT=hom:DP=87:FQ=1:SQ=-1;ID=S2503_raw:GT=hom:DP=195:FQ=1:SQ=-1;ID=S2507_raw:GT=hom:DP=167:FQ=1:SQ=-1;ID=S2668_raw:GT=hom:DP=165:FQ=1:SQ=-1;ID=S2012_raw:GT=hom:DP=101:FQ=1:SQ=-1;ID=S2010_raw:GT=hom:DP=107:FQ=1:SQ=-1;ID=S2662_raw:GT=hom:DP=229:FQ=1:SQ=-1;ID=S2663_raw:GT=hom:DP=171:FQ=1:SQ=-1;ID=S2011_raw:GT=hom:DP=136:FQ=1:SQ=-1;ID=S2504_raw:GT=hom:DP=188:FQ=1:SQ=-1;ID=S2008_raw:GT=hom:DP=122:FQ=1:SQ=-1;ID=S2509_raw:GT=hom:DP=174:FQ=1:SQ=-1;ID=S2508_raw:GT=hom:DP=217:FQ=1:SQ=-1;ID=S2666_raw:GT=hom:DP=154:FQ=1:SQ=-1;ID=S2669_raw:GT=hom:DP=151:FQ=1:SQ=-1;ID=S2166_raw:GT=hom:DP=285:FQ=1:SQ=-1;ID=S1775_raw:GT=hom:DP=152:FQ=1:SQ=-1;ID=S2501_raw:GT=hom:DP=152:FQ=1:SQ=-1;ID=S2500_raw:GT=hom:DP=180:FQ=1:SQ=-1;ID=S2502_raw:GT=hom:DP=249:FQ=1:SQ=-1;ID=S2505_raw:GT=hom:DP=199:FQ=1:SQ=-1;ID=S2176_raw:GT=hom:DP=233:FQ=1:SQ=-1;ID=S2506_raw:GT=hom:DP=165:FQ=1:SQ=-1;ID=S2667_raw:GT=hom:DP=182:FQ=1:SQ=-1;
3	1,22E+08	1,22E+08	G	C	exonic	CASR	nonsynonymous SNV	CASR:NM_000388:exon7:c.3031G>C:p.E1011Q,CASR:NM_001178065:exon7:c.3061G>C:p.E1021Q	rs1801726	Benign	ID=S2009_raw:GT=hom:DP=42:FQ=1:SQ=-1;ID=S2503_raw:GT=hom:DP=180:FQ=1:SQ=-1;ID=S2507_raw:GT=hom:DP=212:FQ=1:SQ=-1;ID=S2668_raw:GT=hom:DP=219:FQ=1:SQ=-1;ID=S2012_raw:GT=hom:DP=38:FQ=1:SQ=-1;ID=S2010_raw:GT=hom:DP=32:FQ=1:SQ=-1;ID=S2662_raw:GT=hom:DP=272:FQ=1:SQ=-1;ID=S2663_raw:GT=hom:DP=196:FQ=1:SQ=-1;ID=S2011_raw:GT=hom:DP=66:FQ=1:SQ=-1;ID=S2504_raw:GT=hom:DP=174:FQ=1:SQ=-1;ID=S2008_raw:GT=hom:DP=45:FQ=1:SQ=-1;ID=S2509_raw:GT=hom:DP=220:FQ=1:SQ=-1;ID=S2508_raw:GT=hom:DP=243:FQ=1:SQ=-1;ID=S2666_raw:GT=hom:DP=146:FQ=1:SQ=-1;ID=S2669_raw:GT=hom:DP=104:FQ=1:SQ=-1;ID=S2166_raw:GT=hom:DP=284:FQ=1:SQ=-1;ID=S1775_raw:GT=hom:DP=165:FQ=1:SQ=-1;ID=S2501_raw:GT=hom:DP=180:FQ=1:SQ=-1;ID=S2500_raw:GT=hom:DP=180:FQ=1:SQ=-1;ID=S2502_raw:GT=hom:DP=223:FQ=1:SQ=-1;ID=S2505_raw:GT=hom:DP=202:FQ=1:SQ=-1;ID=S2176_raw:GT=hom:DP=257:FQ=1:SQ=-1;ID=S2506_raw:GT=hom:DP=145:FQ=1:SQ=-1;ID=S2667_raw:GT=hom:DP=180:FQ=1:SQ=-1;
3	1,22E+08	1,22E+08	A	T	UTR3	CASR	.	NM_000388:c.*60A>T;NM_001178065:c.*60A>T	rs4677948	Benign	ID=S2009_raw:GT=hom:DP=29:FQ=1:SQ=-1;ID=S2503_raw:GT=hom:DP=40:FQ=1:SQ=-1;ID=S2507_raw:GT=hom:DP=43:FQ=1:SQ=-1;ID=S2668_raw:GT=hom:DP=62:FQ=1:SQ=-1;ID=S2012_raw:GT=hom:DP=21:FQ=1:SQ=-1;ID=S2010_raw:GT=hom:DP=21:FQ=1:SQ=-1;ID=S2662_raw:GT=hom:DP=16:FQ=1:SQ=-1;ID=S2663_raw:GT=hom:DP=29:FQ=1:SQ=-1;ID=S2011_raw:GT=hom:DP=19:FQ=1:SQ=-1;ID=S2504_raw:GT=hom:DP=36:FQ=1:SQ=-1;ID=S2008_raw:GT=hom:DP=28:FQ=1:SQ=-1;ID=S2509_raw:GT=hom:DP=44:FQ=1:SQ=-1;ID=S2508_raw:GT=hom:DP=28:FQ=1:SQ=-1;ID=S2666_raw:GT=hom:DP=43:FQ=1:SQ=-1;ID=S2669_raw:GT=hom:DP=47:FQ=1:SQ=-1;ID=S2166_raw:GT=hom:DP=35:FQ=1:SQ=-1;ID=S1775_raw:GT=hom:DP=34:FQ=1:SQ=-1;ID=S2501_raw:GT=hom:DP=25:FQ=1:SQ=-1;ID=S2500_raw:GT=hom:DP=39:FQ=1:SQ=-1;ID=S2502_raw:GT=hom:DP=41:FQ=1:SQ=-1;ID=S2505_raw:GT=hom:DP=28:FQ=1:SQ=-1;ID=S2176_raw:GT=hom:DP=41:FQ=1:SQ=-1;ID=S2506_raw:GT=hom:DP=46:FQ=1:SQ=-1;ID=S2667_raw:GT=hom:DP=45:FQ=1:SQ=-1;
18	57134152	57134152	A	G	intronic	CCBE1	.	.	rs4940876	Benign	ID=S2009_raw:GT=hom:DP=43:FQ=1:SQ=-1;ID=S2503_raw:GT=hom:DP=97:FQ=1:SQ=-1;ID=S2507_raw:GT=hom:DP=72:FQ=1:SQ=-1;ID=S2668_raw:GT=hom:DP=104:FQ=1:SQ=-1;ID=S2012_raw:GT=hom:DP=59:FQ=1:SQ=-1;ID=S2010_raw:GT=hom:DP=56:FQ=1:SQ=-1;ID=S2662_raw:GT=hom:DP=22:FQ=1:SQ=-1;ID=S2663_raw:GT=hom:DP=82:FQ=1:SQ=-1;ID=S2011_raw:GT=hom:DP=63:FQ=1:SQ=-1;ID=S2504_raw:GT=hom:DP=79:FQ=1:SQ=-1;ID=S2008_raw:GT=hom:DP=52:FQ=1:SQ=-1;ID=S2509_raw:GT=hom:DP=90:FQ=1:SQ=-1;ID=S2508_raw:GT=hom:DP=79:FQ=1:SQ=-1;ID=S2666_raw:GT=hom:DP=89:FQ=1:SQ=-1;ID=S2669_raw:GT=hom:DP=72:FQ=1:SQ=-1;ID=S2166_raw:GT=hom:DP=34:FQ=1:SQ=-1;ID=S1775_raw:GT=hom:DP=18:FQ=1:SQ=-1;ID=S2501_raw:GT=hom:DP=72:FQ=1:SQ=-1;ID=S2500_raw:GT=hom:DP=93:FQ=1:SQ=-1;ID=S2502_raw:GT=hom:DP=68:FQ=1:SQ=-1;ID=S2505_raw:GT=hom:DP=87:FQ=1:SQ=-1;ID=S2176_raw:GT=hom:DP=50:FQ=1:SQ=-1;ID=S2506_raw:GT=hom:DP=66:FQ=1:SQ=-1;ID=S2667_raw:GT=hom:DP=93:FQ=1:SQ=-1;
6	1,32E+08	1,32E+08	G	C	exonic	CCN2	nonsynonymous SNV	CCN2:NM_001901:exon2:c.247C>G:p.H83D	rs7451102	.	ID=S2009_raw:GT=hom:DP=20:FQ=1:SQ=-1;ID=S2503_raw:GT=hom:DP=136:FQ=1:SQ=-1;ID=S2507_raw:GT=hom:DP=138:FQ=0.99:SQ=-1;ID=S2668_raw:GT=hom:DP=170:FQ=1:SQ=-1;ID=S2012_raw:GT=hom:DP=14:FQ=1:SQ=-1;ID=S2010_raw:GT=hom:DP=30:FQ=1:SQ=-1;ID=S2662_raw:GT=hom:DP=248:FQ=1:SQ=-1;ID=S2663_raw:GT=hom:DP=196:FQ=1:SQ=-1;ID=S2011_raw:GT=hom:DP=23:FQ=1:SQ=-1;ID=S2504_raw:GT=hom:DP=150:FQ=1:SQ=-1;ID=S2008_raw:GT=hom:DP=23:FQ=1:SQ=-1;ID=S2509_raw:GT=hom:DP=156:FQ=1:SQ=-1;ID=S2508_raw:GT=hom:DP=166:FQ=1:SQ=-1;ID=S2666_raw:GT=hom:DP=74:FQ=1:SQ=-1;ID=S2669_raw:GT=hom:DP=191:FQ=1:SQ=-1;ID=S2166_raw:GT=hom:DP=108:FQ=1:SQ=-1;ID=S1775_raw:GT=hom:DP=10:FQ=1:SQ=-1;ID=S2501_raw:GT=hom:DP=146:FQ=1:SQ=-1;ID=S2500_raw:GT=hom:DP=107:FQ=1:SQ=-1;ID=S2502_raw:GT=hom:DP=121:FQ=1:SQ=-1;ID=S2505_raw:GT=hom:DP=136:FQ=1:SQ=-1;ID=S2176_raw:GT=hom:DP=85:FQ=1:SQ=-1;ID=S2506_raw:GT=hom:DP=98:FQ=1:SQ=-1;ID=S2667_raw:GT=hom:DP=198:FQ=1:SQ=-1;
6	1,32E+08	1,32E+08	T	G	exonic	CCN2	synonymous SNV	CCN2:NM_001901:exon2:c.240A>C:p.L80L	rs12206231	.	ID=S2009_raw:GT=hom:DP=21:FQ=1:SQ=-1;ID=S2503_raw:GT=hom:DP=136:FQ=1:SQ=-1;ID=S2507_raw:GT=hom:DP=128:FQ=1:SQ=-1;ID=S2668_raw:GT=hom:DP=161:FQ=1:SQ=-1;ID=S2012_raw:GT=hom:DP=14:FQ=1:SQ=-1;ID=S2010_raw:GT=hom:DP=32:FQ=1:SQ=-1;ID=S2662_raw:GT=hom:DP=235:FQ=1:SQ=-1;ID=S2663_raw:GT=hom:DP=187:FQ=1:SQ=-1;ID=S2011_raw:GT=hom:DP=26:FQ=1:SQ=-1;ID=S2504_raw:GT=hom:DP=143:FQ=1:SQ=-1;ID=S2008_raw:GT=hom:DP=23:FQ=1:SQ=-1;ID=S2509_raw:GT=hom:DP=148:FQ=1:SQ=-1;ID=S2508_raw:GT=hom:DP=155:FQ=1:SQ=-1;ID=S2666_raw:GT=hom:DP=64:FQ=1:SQ=-1;ID=S2669_raw:GT=hom:DP=178:FQ=1:SQ=-1;ID=S2166_raw:GT=hom:DP=99:FQ=1:SQ=-1;ID=S1775_raw:GT=hom:DP=10:FQ=1:SQ=-1;ID=S2501_raw:GT=hom:DP=143:FQ=1:SQ=-1;ID=S2500_raw:GT=hom:DP=100:FQ=1:SQ=-1;ID=S2502_raw:GT=hom:DP=116:FQ=1:SQ=-1;ID=S2505_raw:GT=hom:DP=132:FQ=1:SQ=-1;ID=S2176_raw:GT=hom:DP=86:FQ=1:SQ=-1;ID=S2506_raw:GT=hom:DP=96:FQ=1:SQ=-1;ID=S2667_raw:GT=hom:DP=186:FQ=1:SQ=-1;
6	1,32E+08	1,32E+08	T	G	exonic	CCN2	synonymous SNV	CCN2:NM_001901:exon2:c.219A>C:p.P73P	rs6934749	.	ID=S2009_raw:GT=hom:DP=24:FQ=1:SQ=-1;ID=S2503_raw:GT=hom:DP=116:FQ=1:SQ=-1;ID=S2507_raw:GT=hom:DP=112:FQ=1:SQ=-1;ID=S2668_raw:GT=hom:DP=134:FQ=1:SQ=-1;ID=S2012_raw:GT=hom:DP=18:FQ=1:SQ=-1;ID=S2010_raw:GT=hom:DP=36:FQ=1:SQ=-1;ID=S2662_raw:GT=hom:DP=194:FQ=1:SQ=-1;ID=S2663_raw:GT=hom:DP=165:FQ=1:SQ=-1;ID=S2011_raw:GT=hom:DP=31:FQ=1:SQ=-1;ID=S2504_raw:GT=hom:DP=126:FQ=1:SQ=-1;ID=S2008_raw:GT=hom:DP=30:FQ=1:SQ=-1;ID=S2509_raw:GT=hom:DP=119:FQ=1:SQ=-1;ID=S2508_raw:GT=hom:DP=148:FQ=1:SQ=-1;ID=S2666_raw:GT=hom:DP=38:FQ=1:SQ=-1;ID=S2669_raw:GT=hom:DP=144:FQ=1:SQ=-1;ID=S2166_raw:GT=hom:DP=91:FQ=1:SQ=-1;ID=S1775_raw:GT=hom:DP=11:FQ=1:SQ=-1;ID=S2501_raw:GT=hom:DP=132:FQ=1:SQ=-1;ID=S2500_raw:GT=hom:DP=96:FQ=1:SQ=-1;ID=S2502_raw:GT=hom:DP=101:FQ=1:SQ=-1;ID=S2505_raw:GT=hom:DP=107:FQ=1:SQ=-1;ID=S2176_raw:GT=hom:DP=76:FQ=1:SQ=-1;ID=S2506_raw:GT=hom:DP=83:FQ=1:SQ=-1;ID=S2667_raw:GT=hom:DP=155:FQ=1:SQ=-1;
5	86667866	86667866	-	A	intronic	CCNH;RASA1	.	.	.	.	ID=S2009_raw:GT=hom:DP=12:FQ=1:SQ=-1;ID=S2503_raw:GT=hom:DP=19:FQ=1:SQ=-1;ID=S2507_raw:GT=hom:DP=19:FQ=1:SQ=-1;ID=S2668_raw:GT=hom:DP=25:FQ=1:SQ=-1;ID=S2012_raw:GT=hom:DP=13:FQ=0.92:SQ=-1;ID=S2010_raw:GT=hom:DP=12:FQ=1:SQ=-1;ID=S2662_raw:GT=hom:DP=2:FQ=1:SQ=-1;ID=S2663_raw:GT=hom:DP=16:FQ=1:SQ=-1;ID=S2011_raw:GT=hom:DP=22:FQ=0.95:SQ=-1;ID=S2504_raw:GT=hom:DP=15:FQ=0.93:SQ=-1;ID=S2008_raw:GT=hom:DP=13:FQ=1:SQ=-1;ID=S2509_raw:GT=hom:DP=18:FQ=1:SQ=-1;ID=S2508_raw:GT=hom:DP=15:FQ=1:SQ=-1;ID=S2666_raw:GT=hom:DP=17:FQ=1:SQ=-1;ID=S2669_raw:GT=hom:DP=12:FQ=1:SQ=-1;ID=S2166_raw:GT=hom:DP=56:FQ=0.96:SQ=-1;ID=S1775_raw:GT=hom:DP=49:FQ=0.98:SQ=-1;ID=S2501_raw:GT=hom:DP=22:FQ=1:SQ=-1;ID=S2500_raw:GT=hom:DP=18:FQ=1:SQ=-1;ID=S2502_raw:GT=hom:DP=19:FQ=1:SQ=-1;ID=S2505_raw:GT=hom:DP=29:FQ=1:SQ=-1;ID=S2176_raw:GT=hom:DP=45:FQ=0.98:SQ=-1;ID=S2506_raw:GT=hom:DP=13:FQ=1:SQ=-1;ID=S2667_raw:GT=hom:DP=20:FQ=1:SQ=-1;
6	47563692	47563692	C	T	exonic	CD2AP	synonymous SNV	CD2AP:NM_012120:exon12:c.1204C>T:p.L402L	rs2039503	Benign	ID=S2009_raw:GT=hom:DP=189:FQ=1:SQ=-1;ID=S2503_raw:GT=hom:DP=114:FQ=1:SQ=-1;ID=S2507_raw:GT=hom:DP=111:FQ=1:SQ=-1;ID=S2668_raw:GT=hom:DP=139:FQ=1:SQ=-1;ID=S2012_raw:GT=hom:DP=209:FQ=1:SQ=-1;ID=S2010_raw:GT=hom:DP=230:FQ=1:SQ=-1;ID=S2662_raw:GT=hom:DP=20:FQ=1:SQ=-1;ID=S2663_raw:GT=hom:DP=110:FQ=1:SQ=-1;ID=S2011_raw:GT=hom:DP=326:FQ=1:SQ=-1;ID=S2504_raw:GT=hom:DP=106:FQ=1:SQ=-1;ID=S2008_raw:GT=hom:DP=215:FQ=1:SQ=-1;ID=S2509_raw:GT=hom:DP=132:FQ=1:SQ=-1;ID=S2508_raw:GT=hom:DP=122:FQ=1:SQ=-1;ID=S2666_raw:GT=hom:DP=102:FQ=1:SQ=-1;ID=S2669_raw:GT=hom:DP=88:FQ=1:SQ=-1;ID=S2166_raw:GT=hom:DP=286:FQ=1:SQ=-1;ID=S1775_raw:GT=hom:DP=256:FQ=1:SQ=-1;ID=S2501_raw:GT=hom:DP=114:FQ=1:SQ=-1;ID=S2500_raw:GT=hom:DP=133:FQ=0.99:SQ=-1;ID=S2502_raw:GT=hom:DP=141:FQ=1:SQ=-1;ID=S2505_raw:GT=hom:DP=123:FQ=1:SQ=-1;ID=S2176_raw:GT=hom:DP=260:FQ=1:SQ=-1;ID=S2506_raw:GT=hom:DP=93:FQ=1:SQ=-1;ID=S2667_raw:GT=hom:DP=116:FQ=1:SQ=-1;
3	1,11E+08	1,11E+08	G	C	exonic	CD96	synonymous SNV	CD96:NM_005816:exon11:c.1362G>C:p.P454P,CD96:NM_198196:exon12:c.1410G>C:p.P470P	rs1533270	Benign	ID=S2009_raw:GT=hom:DP=19:FQ=1:SQ=-1;ID=S2503_raw:GT=hom:DP=108:FQ=1:SQ=-1;ID=S2507_raw:GT=hom:DP=69:FQ=1:SQ=-1;ID=S2668_raw:GT=hom:DP=107:FQ=1:SQ=-1;ID=S2012_raw:GT=hom:DP=29:FQ=1:SQ=-1;ID=S2010_raw:GT=hom:DP=24:FQ=1:SQ=-1;ID=S2662_raw:GT=hom:DP=2:FQ=1:SQ=-1;ID=S2663_raw:GT=hom:DP=109:FQ=1:SQ=-1;ID=S2011_raw:GT=hom:DP=37:FQ=1:SQ=-1;ID=S2504_raw:GT=hom:DP=88:FQ=1:SQ=-1;ID=S2008_raw:GT=hom:DP=25:FQ=1:SQ=-1;ID=S2509_raw:GT=hom:DP=84:FQ=1:SQ=-1;ID=S2508_raw:GT=hom:DP=95:FQ=1:SQ=-1;ID=S2666_raw:GT=hom:DP=101:FQ=1:SQ=-1;ID=S2669_raw:GT=hom:DP=24:FQ=1:SQ=-1;ID=S2166_raw:GT=hom:DP=112:FQ=1:SQ=-1;ID=S1775_raw:GT=hom:DP=84:FQ=1:SQ=-1;ID=S2501_raw:GT=hom:DP=82:FQ=1:SQ=-1;ID=S2500_raw:GT=hom:DP=78:FQ=1:SQ=-1;ID=S2502_raw:GT=hom:DP=85:FQ=1:SQ=-1;ID=S2505_raw:GT=hom:DP=74:FQ=1:SQ=-1;ID=S2176_raw:GT=hom:DP=108:FQ=1:SQ=-1;ID=S2506_raw:GT=hom:DP=79:FQ=1:SQ=-1;ID=S2667_raw:GT=hom:DP=72:FQ=1:SQ=-1;
14	1,03E+08	1,03E+08	T	C	intronic	CDC42BPB	.	.	rs8007924	.	ID=S2009_raw:GT=hom:DP=6:FQ=1:SQ=-1;ID=S2503_raw:GT=hom:DP=20:FQ=1:SQ=-1;ID=S2507_raw:GT=hom:DP=16:FQ=1:SQ=-1;ID=S2668_raw:GT=hom:DP=17:FQ=1:SQ=-1;ID=S2012_raw:GT=hom:DP=13:FQ=1:SQ=-1;ID=S2010_raw:GT=hom:DP=14:FQ=1:SQ=-1;ID=S2662_raw:GT=hom:DP=22:FQ=1:SQ=-1;ID=S2663_raw:GT=hom:DP=14:FQ=1:SQ=-1;ID=S2011_raw:GT=hom:DP=12:FQ=1:SQ=-1;ID=S2504_raw:GT=hom:DP=16:FQ=1:SQ=-1;ID=S2008_raw:GT=hom:DP=6:FQ=1:SQ=-1;ID=S2509_raw:GT=hom:DP=8:FQ=1:SQ=-1;ID=S2508_raw:GT=hom:DP=13:FQ=1:SQ=-1;ID=S2666_raw:GT=hom:DP=13:FQ=1:SQ=-1;ID=S2669_raw:GT=hom:DP=10:FQ=1:SQ=-1;ID=S2166_raw:GT=hom:DP=24:FQ=1:SQ=-1;ID=S1775_raw:GT=hom:DP=11:FQ=1:SQ=-1;ID=S2501_raw:GT=hom:DP=17:FQ=1:SQ=-1;ID=S2500_raw:GT=hom:DP=12:FQ=1:SQ=-1;ID=S2502_raw:GT=hom:DP=23:FQ=1:SQ=-1;ID=S2505_raw:GT=hom:DP=14:FQ=1:SQ=-1;ID=S2176_raw:GT=hom:DP=22:FQ=1:SQ=-1;ID=S2506_raw:GT=hom:DP=10:FQ=1:SQ=-1;ID=S2667_raw:GT=hom:DP=20:FQ=1:SQ=-1;
14	1,03E+08	1,03E+08	T	A	intronic	CDC42BPB	.	.	rs2093414	.	ID=S2009_raw:GT=hom:DP=18:FQ=1:SQ=-1;ID=S2503_raw:GT=hom:DP=14:FQ=1:SQ=-1;ID=S2507_raw:GT=hom:DP=7:FQ=1:SQ=-1;ID=S2668_raw:GT=hom:DP=9:FQ=1:SQ=-1;ID=S2012_raw:GT=hom:DP=15:FQ=1:SQ=-1;ID=S2010_raw:GT=hom:DP=18:FQ=1:SQ=-1;ID=S2662_raw:GT=hom:DP=3:FQ=1:SQ=-1;ID=S2663_raw:GT=hom:DP=6:FQ=1:SQ=-1;ID=S2011_raw:GT=hom:DP=29:FQ=1:SQ=-1;ID=S2504_raw:GT=hom:DP=11:FQ=1:SQ=-1;ID=S2008_raw:GT=hom:DP=13:FQ=1:SQ=-1;ID=S2509_raw:GT=hom:DP=8:FQ=1:SQ=-1;ID=S2508_raw:GT=hom:DP=9:FQ=1:SQ=-1;ID=S2666_raw:GT=hom:DP=1:FQ=1:SQ=-1;ID=S2669_raw:GT=hom:DP=7:FQ=1:SQ=-1;ID=S2166_raw:GT=hom:DP=33:FQ=1:SQ=-1;ID=S1775_raw:GT=hom:DP=32:FQ=1:SQ=-1;ID=S2501_raw:GT=hom:DP=9:FQ=1:SQ=-1;ID=S2500_raw:GT=hom:DP=5:FQ=1:SQ=-1;ID=S2502_raw:GT=hom:DP=6:FQ=1:SQ=-1;ID=S2505_raw:GT=hom:DP=5:FQ=1:SQ=-1;ID=S2176_raw:GT=hom:DP=39:FQ=1:SQ=-1;ID=S2506_raw:GT=hom:DP=14:FQ=1:SQ=-1;ID=S2667_raw:GT=hom:DP=10:FQ=1:SQ=-1;
14	1,03E+08	1,03E+08	G	T	intronic	CDC42BPB	.	.	rs1018550	.	ID=S2009_raw:GT=hom:DP=11:FQ=1:SQ=-1;ID=S2503_raw:GT=hom:DP=213:FQ=1:SQ=-1;ID=S2507_raw:GT=hom:DP=171:FQ=1:SQ=-1;ID=S2668_raw:GT=hom:DP=203:FQ=1:SQ=-1;ID=S2012_raw:GT=hom:DP=13:FQ=1:SQ=-1;ID=S2010_raw:GT=hom:DP=19:FQ=1:SQ=-1;ID=S2662_raw:GT=hom:DP=346:FQ=1:SQ=-1;ID=S2663_raw:GT=hom:DP=220:FQ=1:SQ=-1;ID=S2011_raw:GT=hom:DP=15:FQ=1:SQ=-1;ID=S2504_raw:GT=hom:DP=207:FQ=1:SQ=-1;ID=S2008_raw:GT=hom:DP=15:FQ=1:SQ=-1;ID=S2509_raw:GT=hom:DP=188:FQ=1:SQ=-1;ID=S2508_raw:GT=hom:DP=241:FQ=1:SQ=-1;ID=S2666_raw:GT=hom:DP=195:FQ=1:SQ=-1;ID=S2669_raw:GT=hom:DP=195:FQ=1:SQ=-1;ID=S2166_raw:GT=hom:DP=257:FQ=1:SQ=-1;ID=S1775_raw:GT=hom:DP=145:FQ=1:SQ=-1;ID=S2501_raw:GT=hom:DP=160:FQ=1:SQ=-1;ID=S2500_raw:GT=hom:DP=178:FQ=1:SQ=-1;ID=S2502_raw:GT=hom:DP=198:FQ=1:SQ=-1;ID=S2505_raw:GT=hom:DP=181:FQ=1:SQ=-1;ID=S2176_raw:GT=hom:DP=214:FQ=1:SQ=-1;ID=S2506_raw:GT=hom:DP=166:FQ=1:SQ=-1;ID=S2667_raw:GT=hom:DP=214:FQ=1:SQ=-1;
9	1,23E+08	1,23E+08	C	A	UTR5	CDK5RAP2	.	NM_001272039:c.-3G>T;NM_001011649:c.-3G>T;NM_018249:c.-3G>T	rs932975	Benign	ID=S2009_raw:GT=hom:DP=64:FQ=1:SQ=-1;ID=S2503_raw:GT=hom:DP=127:FQ=1:SQ=-1;ID=S2507_raw:GT=hom:DP=137:FQ=1:SQ=-1;ID=S2668_raw:GT=hom:DP=94:FQ=1:SQ=-1;ID=S2012_raw:GT=hom:DP=56:FQ=1:SQ=-1;ID=S2010_raw:GT=hom:DP=58:FQ=1:SQ=-1;ID=S2662_raw:GT=hom:DP=152:FQ=1:SQ=-1;ID=S2663_raw:GT=hom:DP=120:FQ=1:SQ=-1;ID=S2011_raw:GT=hom:DP=58:FQ=1:SQ=-1;ID=S2504_raw:GT=hom:DP=125:FQ=1:SQ=-1;ID=S2008_raw:GT=hom:DP=73:FQ=1:SQ=-1;ID=S2509_raw:GT=hom:DP=128:FQ=1:SQ=-1;ID=S2508_raw:GT=hom:DP=143:FQ=1:SQ=-1;ID=S2666_raw:GT=hom:DP=50:FQ=1:SQ=-1;ID=S2669_raw:GT=hom:DP=128:FQ=1:SQ=-1;ID=S2166_raw:GT=hom:DP=96:FQ=1:SQ=-1;ID=S1775_raw:GT=hom:DP=97:FQ=1:SQ=-1;ID=S2501_raw:GT=hom:DP=85:FQ=1:SQ=-1;ID=S2500_raw:GT=hom:DP=109:FQ=1:SQ=-1;ID=S2502_raw:GT=hom:DP=113:FQ=1:SQ=-1;ID=S2505_raw:GT=hom:DP=94:FQ=1:SQ=-1;ID=S2176_raw:GT=hom:DP=75:FQ=1:SQ=-1;ID=S2506_raw:GT=hom:DP=97:FQ=1:SQ=-1;ID=S2667_raw:GT=hom:DP=113:FQ=1:SQ=-1;
9	1,23E+08	1,23E+08	A	G	UTR5	CDK5RAP2	.	NM_001272039:c.-19T>C;NM_001011649:c.-19T>C;NM_018249:c.-19T>C	rs932974	Benign	ID=S2009_raw:GT=hom:DP=54:FQ=1:SQ=-1;ID=S2503_raw:GT=hom:DP=112:FQ=1:SQ=-1;ID=S2507_raw:GT=hom:DP=133:FQ=1:SQ=-1;ID=S2668_raw:GT=hom:DP=78:FQ=1:SQ=-1;ID=S2012_raw:GT=hom:DP=57:FQ=1:SQ=-1;ID=S2010_raw:GT=hom:DP=60:FQ=1:SQ=-1;ID=S2662_raw:GT=hom:DP=120:FQ=1:SQ=-1;ID=S2663_raw:GT=hom:DP=100:FQ=1:SQ=-1;ID=S2011_raw:GT=hom:DP=63:FQ=1:SQ=-1;ID=S2504_raw:GT=hom:DP=110:FQ=1:SQ=-1;ID=S2008_raw:GT=hom:DP=60:FQ=1:SQ=-1;ID=S2509_raw:GT=hom:DP=101:FQ=1:SQ=-1;ID=S2508_raw:GT=hom:DP=119:FQ=1:SQ=-1;ID=S2666_raw:GT=hom:DP=36:FQ=1:SQ=-1;ID=S2669_raw:GT=hom:DP=104:FQ=1:SQ=-1;ID=S2166_raw:GT=hom:DP=74:FQ=1:SQ=-1;ID=S1775_raw:GT=hom:DP=77:FQ=1:SQ=-1;ID=S2501_raw:GT=hom:DP=71:FQ=1:SQ=-1;ID=S2500_raw:GT=hom:DP=73:FQ=1:SQ=-1;ID=S2502_raw:GT=hom:DP=94:FQ=1:SQ=-1;ID=S2505_raw:GT=hom:DP=78:FQ=1:SQ=-1;ID=S2176_raw:GT=hom:DP=59:FQ=1:SQ=-1;ID=S2506_raw:GT=hom:DP=81:FQ=1:SQ=-1;ID=S2667_raw:GT=hom:DP=89:FQ=1:SQ=-1;
13	25457207	25457207	T	C	UTR3	CENPJ	.	NM_018451:c.*108A>G	rs9318911	.	ID=S2009_raw:GT=hom:DP=42:FQ=1:SQ=-1;ID=S2503_raw:GT=hom:DP=6:FQ=1:SQ=-1;ID=S2507_raw:GT=hom:DP=21:FQ=1:SQ=-1;ID=S2668_raw:GT=hom:DP=13:FQ=1:SQ=-1;ID=S2012_raw:GT=hom:DP=55:FQ=1:SQ=-1;ID=S2010_raw:GT=hom:DP=57:FQ=1:SQ=-1;ID=S2662_raw:GT=hom:DP=1:FQ=1:SQ=-1;ID=S2663_raw:GT=hom:DP=9:FQ=1:SQ=-1;ID=S2011_raw:GT=hom:DP=82:FQ=1:SQ=-1;ID=S2504_raw:GT=hom:DP=16:FQ=1:SQ=-1;ID=S2008_raw:GT=hom:DP=51:FQ=1:SQ=-1;ID=S2509_raw:GT=hom:DP=7:FQ=1:SQ=-1;ID=S2508_raw:GT=hom:DP=10:FQ=1:SQ=-1;ID=S2666_raw:GT=hom:DP=22:FQ=1:SQ=-1;ID=S2669_raw:GT=hom:DP=9:FQ=1:SQ=-1;ID=S2166_raw:GT=hom:DP=27:FQ=1:SQ=-1;ID=S1775_raw:GT=hom:DP=11:FQ=1:SQ=-1;ID=S2501_raw:GT=hom:DP=14:FQ=1:SQ=-1;ID=S2500_raw:GT=hom:DP=24:FQ=1:SQ=-1;ID=S2502_raw:GT=hom:DP=22:FQ=1:SQ=-1;ID=S2505_raw:GT=hom:DP=14:FQ=1:SQ=-1;ID=S2176_raw:GT=hom:DP=18:FQ=1:SQ=-1;ID=S2506_raw:GT=hom:DP=13:FQ=1:SQ=-1;ID=S2667_raw:GT=hom:DP=11:FQ=1:SQ=-1;
10	95262981	95262981	A	G	exonic	CEP55	nonsynonymous SNV	CEP55:NM_001127182:exon3:c.295A>G:p.T99A,CEP55:NM_018131:exon3:c.295A>G:p.T99A	rs7080916	.	ID=S2009_raw:GT=hom:DP=79:FQ=1:SQ=-1;ID=S2503_raw:GT=hom:DP=112:FQ=1:SQ=-1;ID=S2507_raw:GT=hom:DP=95:FQ=1:SQ=-1;ID=S2668_raw:GT=hom:DP=109:FQ=1:SQ=-1;ID=S2012_raw:GT=hom:DP=102:FQ=1:SQ=-1;ID=S2010_raw:GT=hom:DP=103:FQ=1:SQ=-1;ID=S2662_raw:GT=hom:DP=12:FQ=1:SQ=-1;ID=S2663_raw:GT=hom:DP=90:FQ=1:SQ=-1;ID=S2011_raw:GT=hom:DP=115:FQ=1:SQ=-1;ID=S2504_raw:GT=hom:DP=119:FQ=1:SQ=-1;ID=S2008_raw:GT=hom:DP=88:FQ=1:SQ=-1;ID=S2509_raw:GT=hom:DP=95:FQ=1:SQ=-1;ID=S2508_raw:GT=hom:DP=99:FQ=1:SQ=-1;ID=S2666_raw:GT=hom:DP=129:FQ=1:SQ=-1;ID=S2669_raw:GT=hom:DP=72:FQ=0.97:SQ=-1;ID=S2166_raw:GT=hom:DP=190:FQ=1:SQ=-1;ID=S1775_raw:GT=hom:DP=174:FQ=1:SQ=-1;ID=S2501_raw:GT=hom:DP=97:FQ=1:SQ=-1;ID=S2500_raw:GT=hom:DP=115:FQ=1:SQ=-1;ID=S2502_raw:GT=hom:DP=103:FQ=1:SQ=-1;ID=S2505_raw:GT=hom:DP=120:FQ=1:SQ=-1;ID=S2176_raw:GT=hom:DP=215:FQ=1:SQ=-1;ID=S2506_raw:GT=hom:DP=100:FQ=1:SQ=-1;ID=S2667_raw:GT=hom:DP=85:FQ=1:SQ=-1;
10	95279653	95279653	A	G	intronic	CEP55	.	.	rs7068719	.	ID=S2009_raw:GT=hom:DP=7:FQ=1:SQ=-1;ID=S2503_raw:GT=hom:DP=11:FQ=1:SQ=-1;ID=S2507_raw:GT=hom:DP=5:FQ=1:SQ=-1;ID=S2668_raw:GT=hom:DP=13:FQ=1:SQ=-1;ID=S2012_raw:GT=hom:DP=7:FQ=1:SQ=-1;ID=S2010_raw:GT=hom:DP=6:FQ=1:SQ=-1;ID=S2662_raw:GT=hom:DP=4:FQ=1:SQ=-1;ID=S2663_raw:GT=hom:DP=6:FQ=1:SQ=-1;ID=S2011_raw:GT=hom:DP=8:FQ=1:SQ=-1;ID=S2504_raw:GT=hom:DP=11:FQ=1:SQ=-1;ID=S2008_raw:GT=hom:DP=9:FQ=1:SQ=-1;ID=S2509_raw:GT=hom:DP=8:FQ=1:SQ=-1;ID=S2508_raw:GT=hom:DP=8:FQ=1:SQ=-1;ID=S2666_raw:GT=hom:DP=8:FQ=1:SQ=-1;ID=S2669_raw:GT=hom:DP=5:FQ=1:SQ=-1;ID=S2166_raw:GT=hom:DP=12:FQ=1:SQ=-1;ID=S1775_raw:GT=hom:DP=9:FQ=1:SQ=-1;ID=S2501_raw:GT=hom:DP=9:FQ=1:SQ=-1;ID=S2500_raw:GT=hom:DP=9:FQ=1:SQ=-1;ID=S2502_raw:GT=hom:DP=10:FQ=1:SQ=-1;ID=S2505_raw:GT=hom:DP=12:FQ=1:SQ=-1;ID=S2176_raw:GT=hom:DP=15:FQ=1:SQ=-1;ID=S2506_raw:GT=hom:DP=8:FQ=1:SQ=-1;ID=S2667_raw:GT=hom:DP=11:FQ=1:SQ=-1;
3	1,34E+08	1,34E+08	G	C	intronic	CEP63	.	.	rs12632372	.	ID=S2009_raw:GT=hom:DP=10:FQ=1:SQ=-1;ID=S2503_raw:GT=hom:DP=49:FQ=1:SQ=-1;ID=S2507_raw:GT=hom:DP=42:FQ=1:SQ=-1;ID=S2668_raw:GT=hom:DP=94:FQ=1:SQ=-1;ID=S2012_raw:GT=hom:DP=9:FQ=1:SQ=-1;ID=S2010_raw:GT=hom:DP=13:FQ=1:SQ=-1;ID=S2662_raw:GT=hom:DP=3:FQ=1:SQ=-1;ID=S2663_raw:GT=hom:DP=67:FQ=1:SQ=-1;ID=S2011_raw:GT=hom:DP=13:FQ=1:SQ=-1;ID=S2504_raw:GT=hom:DP=52:FQ=1:SQ=-1;ID=S2008_raw:GT=hom:DP=13:FQ=1:SQ=-1;ID=S2509_raw:GT=hom:DP=25:FQ=1:SQ=-1;ID=S2508_raw:GT=hom:DP=63:FQ=1:SQ=-1;ID=S2666_raw:GT=hom:DP=91:FQ=1:SQ=-1;ID=S2669_raw:GT=hom:DP=43:FQ=1:SQ=-1;ID=S2166_raw:GT=hom:DP=107:FQ=1:SQ=-1;ID=S1775_raw:GT=hom:DP=70:FQ=1:SQ=-1;ID=S2501_raw:GT=hom:DP=41:FQ=1:SQ=-1;ID=S2500_raw:GT=hom:DP=50:FQ=1:SQ=-1;ID=S2502_raw:GT=hom:DP=42:FQ=1:SQ=-1;ID=S2505_raw:GT=hom:DP=53:FQ=1:SQ=-1;ID=S2176_raw:GT=hom:DP=111:FQ=1:SQ=-1;ID=S2506_raw:GT=hom:DP=30:FQ=1:SQ=-1;ID=S2667_raw:GT=hom:DP=59:FQ=1:SQ=-1;
3	1,34E+08	1,34E+08	T	A	UTR3	CEP63	.	NM_001353118:c.*18T>A;NM_001353122:c.*18T>A;NM_001353110:c.*18T>A;NM_001353109:c.*18T>A;NM_001042383:c.*18T>A;NM_001353120:c.*18T>A;NM_001353108:c.*18T>A;NM_001042400:c.*18T>A;NM_001353119:c.*18T>A;NM_001353121:c.*18T>A;NM_001353126:c.*18T>A;NM_001353125:c.*18T>A;NM_025180:c.*18T>A;NM_001353112:c.*18T>A;NM_001353111:c.*18T>A	rs6777283	.	ID=S2009_raw:GT=hom:DP=68:FQ=1:SQ=-1;ID=S2503_raw:GT=hom:DP=45:FQ=1:SQ=-1;ID=S2507_raw:GT=hom:DP=32:FQ=1:SQ=-1;ID=S2668_raw:GT=hom:DP=27:FQ=1:SQ=-1;ID=S2012_raw:GT=hom:DP=71:FQ=1:SQ=-1;ID=S2010_raw:GT=hom:DP=74:FQ=1:SQ=-1;ID=S2662_raw:GT=hom:DP=4:FQ=1:SQ=-1;ID=S2663_raw:GT=hom:DP=24:FQ=1:SQ=-1;ID=S2011_raw:GT=hom:DP=118:FQ=1:SQ=-1;ID=S2504_raw:GT=hom:DP=21:FQ=1:SQ=-1;ID=S2008_raw:GT=hom:DP=71:FQ=1:SQ=-1;ID=S2509_raw:GT=hom:DP=46:FQ=1:SQ=-1;ID=S2508_raw:GT=hom:DP=39:FQ=1:SQ=-1;ID=S2666_raw:GT=hom:DP=19:FQ=1:SQ=-1;ID=S2669_raw:GT=hom:DP=15:FQ=1:SQ=-1;ID=S2166_raw:GT=hom:DP=91:FQ=1:SQ=-1;ID=S1775_raw:GT=hom:DP=78:FQ=1:SQ=-1;ID=S2501_raw:GT=hom:DP=23:FQ=1:SQ=-1;ID=S2500_raw:GT=hom:DP=32:FQ=1:SQ=-1;ID=S2502_raw:GT=hom:DP=34:FQ=1:SQ=-1;ID=S2505_raw:GT=hom:DP=43:FQ=1:SQ=-1;ID=S2176_raw:GT=hom:DP=68:FQ=1:SQ=-1;ID=S2506_raw:GT=hom:DP=26:FQ=1:SQ=-1;ID=S2667_raw:GT=hom:DP=20:FQ=1:SQ=-1;
5	1,23E+08	1,23E+08	G	A	intronic	CEP120	.	.	rs485847	.	ID=S2009_raw:GT=hom:DP=74:FQ=1:SQ=-1;ID=S2503_raw:GT=hom:DP=39:FQ=1:SQ=-1;ID=S2507_raw:GT=hom:DP=31:FQ=1:SQ=-1;ID=S2668_raw:GT=hom:DP=38:FQ=1:SQ=-1;ID=S2012_raw:GT=hom:DP=118:FQ=1:SQ=-1;ID=S2010_raw:GT=hom:DP=134:FQ=1:SQ=-1;ID=S2662_raw:GT=hom:DP=6:FQ=1:SQ=-1;ID=S2663_raw:GT=hom:DP=45:FQ=1:SQ=-1;ID=S2011_raw:GT=hom:DP=189:FQ=1:SQ=-1;ID=S2504_raw:GT=hom:DP=47:FQ=1:SQ=-1;ID=S2008_raw:GT=hom:DP=118:FQ=1:SQ=-1;ID=S2509_raw:GT=hom:DP=44:FQ=1:SQ=-1;ID=S2508_raw:GT=hom:DP=48:FQ=1:SQ=-1;ID=S2666_raw:GT=hom:DP=57:FQ=1:SQ=-1;ID=S2669_raw:GT=hom:DP=34:FQ=1:SQ=-1;ID=S2166_raw:GT=hom:DP=164:FQ=1:SQ=-1;ID=S1775_raw:GT=hom:DP=90:FQ=1:SQ=-1;ID=S2501_raw:GT=hom:DP=45:FQ=1:SQ=-1;ID=S2500_raw:GT=hom:DP=50:FQ=1:SQ=-1;ID=S2502_raw:GT=hom:DP=43:FQ=1:SQ=-1;ID=S2505_raw:GT=hom:DP=49:FQ=1:SQ=-1;ID=S2176_raw:GT=hom:DP=174:FQ=1:SQ=-1;ID=S2506_raw:GT=hom:DP=28:FQ=1:SQ=-1;ID=S2667_raw:GT=hom:DP=34:FQ=1:SQ=-1;
4	1,11E+08	1,11E+08	G	C	intronic	CFI	.	.	rs7441380	.	ID=S2009_raw:GT=hom:DP=32:FQ=1:SQ=-1;ID=S2503_raw:GT=hom:DP=77:FQ=1:SQ=-1;ID=S2507_raw:GT=hom:DP=74:FQ=1:SQ=-1;ID=S2668_raw:GT=hom:DP=73:FQ=1:SQ=-1;ID=S2012_raw:GT=hom:DP=33:FQ=1:SQ=-1;ID=S2010_raw:GT=hom:DP=45:FQ=1:SQ=-1;ID=S2662_raw:GT=hom:DP=3:FQ=1:SQ=-1;ID=S2663_raw:GT=hom:DP=61:FQ=1:SQ=-1;ID=S2011_raw:GT=hom:DP=66:FQ=1:SQ=-1;ID=S2504_raw:GT=hom:DP=71:FQ=1:SQ=-1;ID=S2008_raw:GT=hom:DP=35:FQ=1:SQ=-1;ID=S2509_raw:GT=hom:DP=76:FQ=1:SQ=-1;ID=S2508_raw:GT=hom:DP=77:FQ=1:SQ=-1;ID=S2666_raw:GT=hom:DP=79:FQ=1:SQ=-1;ID=S2669_raw:GT=hom:DP=33:FQ=1:SQ=-1;ID=S2166_raw:GT=hom:DP=30:FQ=1:SQ=-1;ID=S1775_raw:GT=hom:DP=20:FQ=1:SQ=-1;ID=S2501_raw:GT=hom:DP=64:FQ=0.98:SQ=-1;ID=S2500_raw:GT=hom:DP=88:FQ=1:SQ=-1;ID=S2502_raw:GT=hom:DP=69:FQ=1:SQ=-1;ID=S2505_raw:GT=hom:DP=74:FQ=1:SQ=-1;ID=S2176_raw:GT=hom:DP=41:FQ=1:SQ=-1;ID=S2506_raw:GT=hom:DP=55:FQ=1:SQ=-1;ID=S2667_raw:GT=hom:DP=61:FQ=1:SQ=-1;
15	78911261	78911261	T	C	splicing	CHRNA3	.	NM_001166694:exon2:c.83-4A>G;NM_000743:exon2:c.83-4A>G	rs2869547	.	ID=S2009_raw:GT=hom:DP=27:FQ=1:SQ=-1;ID=S2503_raw:GT=hom:DP=35:FQ=1:SQ=-1;ID=S2507_raw:GT=hom:DP=39:FQ=1:SQ=-1;ID=S2668_raw:GT=hom:DP=69:FQ=1:SQ=-1;ID=S2012_raw:GT=hom:DP=19:FQ=1:SQ=-1;ID=S2010_raw:GT=hom:DP=27:FQ=1:SQ=-1;ID=S2662_raw:GT=hom:DP=48:FQ=1:SQ=-1;ID=S2663_raw:GT=hom:DP=26:FQ=1:SQ=-1;ID=S2011_raw:GT=hom:DP=37:FQ=1:SQ=-1;ID=S2504_raw:GT=hom:DP=44:FQ=1:SQ=-1;ID=S2008_raw:GT=hom:DP=21:FQ=1:SQ=-1;ID=S2509_raw:GT=hom:DP=39:FQ=1:SQ=-1;ID=S2508_raw:GT=hom:DP=47:FQ=1:SQ=-1;ID=S2666_raw:GT=hom:DP=45:FQ=1:SQ=-1;ID=S2669_raw:GT=hom:DP=37:FQ=1:SQ=-1;ID=S2166_raw:GT=hom:DP=18:FQ=1:SQ=-1;ID=S1775_raw:GT=hom:DP=14:FQ=1:SQ=-1;ID=S2501_raw:GT=hom:DP=37:FQ=1:SQ=-1;ID=S2500_raw:GT=hom:DP=42:FQ=1:SQ=-1;ID=S2502_raw:GT=hom:DP=37:FQ=1:SQ=-1;ID=S2505_raw:GT=hom:DP=43:FQ=1:SQ=-1;ID=S2176_raw:GT=hom:DP=33:FQ=1:SQ=-1;ID=S2506_raw:GT=hom:DP=44:FQ=1:SQ=-1;ID=S2667_raw:GT=hom:DP=31:FQ=1:SQ=-1;
1	87018768	87018768	A	G	intronic	CLCA4	.	.	rs6676936	.	ID=S2009_raw:GT=hom:DP=23:FQ=1:SQ=-1;ID=S2503_raw:GT=hom:DP=91:FQ=1:SQ=-1;ID=S2507_raw:GT=hom:DP=111:FQ=1:SQ=-1;ID=S2668_raw:GT=hom:DP=130:FQ=1:SQ=-1;ID=S2012_raw:GT=hom:DP=15:FQ=1:SQ=-1;ID=S2010_raw:GT=hom:DP=14:FQ=1:SQ=-1;ID=S2662_raw:GT=hom:DP=193:FQ=1:SQ=-1;ID=S2663_raw:GT=hom:DP=118:FQ=1:SQ=-1;ID=S2011_raw:GT=hom:DP=26:FQ=1:SQ=-1;ID=S2504_raw:GT=hom:DP=119:FQ=1:SQ=-1;ID=S2008_raw:GT=hom:DP=26:FQ=1:SQ=-1;ID=S2509_raw:GT=hom:DP=113:FQ=1:SQ=-1;ID=S2508_raw:GT=hom:DP=134:FQ=1:SQ=-1;ID=S2666_raw:GT=hom:DP=93:FQ=1:SQ=-1;ID=S2669_raw:GT=hom:DP=119:FQ=1:SQ=-1;ID=S2166_raw:GT=hom:DP=145:FQ=1:SQ=-1;ID=S1775_raw:GT=hom:DP=68:FQ=1:SQ=-1;ID=S2501_raw:GT=hom:DP=107:FQ=1:SQ=-1;ID=S2500_raw:GT=hom:DP=108:FQ=1:SQ=-1;ID=S2502_raw:GT=hom:DP=129:FQ=1:SQ=-1;ID=S2505_raw:GT=hom:DP=114:FQ=1:SQ=-1;ID=S2176_raw:GT=hom:DP=151:FQ=1:SQ=-1;ID=S2506_raw:GT=hom:DP=100:FQ=1:SQ=-1;ID=S2667_raw:GT=hom:DP=93:FQ=1:SQ=-1;
4	1,71E+08	1,71E+08	G	A	exonic	CLCN3	nonsynonymous SNV	CLCN3:NM_001243374:exon1:c.50G>A:p.S17N	rs4692739	.	ID=S2009_raw:GT=hom:DP=24:FQ=1:SQ=-1;ID=S2503_raw:GT=hom:DP=97:FQ=1:SQ=-1;ID=S2507_raw:GT=hom:DP=72:FQ=1:SQ=-1;ID=S2668_raw:GT=hom:DP=95:FQ=1:SQ=-1;ID=S2012_raw:GT=hom:DP=41:FQ=1:SQ=-1;ID=S2010_raw:GT=hom:DP=37:FQ=1:SQ=-1;ID=S2662_raw:GT=hom:DP=15:FQ=1:SQ=-1;ID=S2663_raw:GT=hom:DP=102:FQ=1:SQ=-1;ID=S2011_raw:GT=hom:DP=67:FQ=1:SQ=-1;ID=S2504_raw:GT=hom:DP=99:FQ=1:SQ=-1;ID=S2008_raw:GT=hom:DP=37:FQ=1:SQ=-1;ID=S2509_raw:GT=hom:DP=91:FQ=1:SQ=-1;ID=S2508_raw:GT=hom:DP=69:FQ=1:SQ=-1;ID=S2666_raw:GT=hom:DP=112:FQ=1:SQ=-1;ID=S2669_raw:GT=hom:DP=89:FQ=1:SQ=-1;ID=S2166_raw:GT=hom:DP=126:FQ=1:SQ=-1;ID=S1775_raw:GT=hom:DP=104:FQ=1:SQ=-1;ID=S2501_raw:GT=hom:DP=72:FQ=1:SQ=-1;ID=S2500_raw:GT=hom:DP=87:FQ=1:SQ=-1;ID=S2502_raw:GT=hom:DP=80:FQ=1:SQ=-1;ID=S2505_raw:GT=hom:DP=66:FQ=1:SQ=-1;ID=S2176_raw:GT=hom:DP=136:FQ=1:SQ=-1;ID=S2506_raw:GT=hom:DP=71:FQ=1:SQ=-1;ID=S2667_raw:GT=hom:DP=76:FQ=0.99:SQ=-1;
1	16362621	16362621	G	A	intergenic	CLCNKA;CLCNKB	.	dist=2076;dist=7610	rs2009044	.	ID=S2009_raw:GT=hom:DP=52:FQ=1:SQ=-1;ID=S2503_raw:GT=hom:DP=189:FQ=1:SQ=-1;ID=S2507_raw:GT=hom:DP=310:FQ=1:SQ=-1;ID=S2668_raw:GT=hom:DP=368:FQ=1:SQ=-1;ID=S2012_raw:GT=hom:DP=61:FQ=1:SQ=-1;ID=S2010_raw:GT=hom:DP=56:FQ=1:SQ=-1;ID=S2662_raw:GT=hom:DP=484:FQ=1:SQ=-1;ID=S2663_raw:GT=hom:DP=136:FQ=1:SQ=-1;ID=S2011_raw:GT=hom:DP=98:FQ=1:SQ=-1;ID=S2504_raw:GT=hom:DP=267:FQ=1:SQ=-1;ID=S2008_raw:GT=hom:DP=61:FQ=1:SQ=-1;ID=S2509_raw:GT=hom:DP=286:FQ=1:SQ=-1;ID=S2508_raw:GT=hom:DP=287:FQ=1:SQ=-1;ID=S2666_raw:GT=hom:DP=363:FQ=1:SQ=-1;ID=S2669_raw:GT=hom:DP=315:FQ=1:SQ=-1;ID=S2166_raw:GT=hom:DP=241:FQ=1:SQ=-1;ID=S1775_raw:GT=hom:DP=242:FQ=1:SQ=-1;ID=S2501_raw:GT=hom:DP=242:FQ=1:SQ=-1;ID=S2500_raw:GT=hom:DP=254:FQ=1:SQ=-1;ID=S2502_raw:GT=hom:DP=326:FQ=1:SQ=-1;ID=S2505_raw:GT=hom:DP=273:FQ=1:SQ=-1;ID=S2176_raw:GT=hom:DP=243:FQ=1:SQ=-1;ID=S2506_raw:GT=hom:DP=245:FQ=1:SQ=-1;ID=S2667_raw:GT=hom:DP=292:FQ=1:SQ=-1;
11	72273172	72273172	A	G	intergenic	CLPB;LINC01537	.	dist=127444;dist=8528	rs904468	.	ID=S2009_raw:GT=hom:DP=9:FQ=1:SQ=-1;ID=S2503_raw:GT=hom:DP=18:FQ=1:SQ=-1;ID=S2507_raw:GT=hom:DP=11:FQ=1:SQ=-1;ID=S2668_raw:GT=hom:DP=21:FQ=1:SQ=-1;ID=S2012_raw:GT=hom:DP=8:FQ=1:SQ=-1;ID=S2010_raw:GT=hom:DP=3:FQ=1:SQ=-1;ID=S2662_raw:GT=hom:DP=13:FQ=1:SQ=-1;ID=S2663_raw:GT=hom:DP=3:FQ=1:SQ=-1;ID=S2011_raw:GT=hom:DP=14:FQ=1:SQ=-1;ID=S2504_raw:GT=hom:DP=20:FQ=1:SQ=-1;ID=S2008_raw:GT=hom:DP=11:FQ=1:SQ=-1;ID=S2509_raw:GT=hom:DP=9:FQ=1:SQ=-1;ID=S2508_raw:GT=hom:DP=20:FQ=1:SQ=-1;ID=S2666_raw:GT=hom:DP=9:FQ=1:SQ=-1;ID=S2669_raw:GT=hom:DP=8:FQ=1:SQ=-1;ID=S2166_raw:GT=hom:DP=13:FQ=1:SQ=-1;ID=S1775_raw:GT=hom:DP=11:FQ=1:SQ=-1;ID=S2501_raw:GT=hom:DP=7:FQ=1:SQ=-1;ID=S2500_raw:GT=hom:DP=19:FQ=1:SQ=-1;ID=S2502_raw:GT=hom:DP=18:FQ=1:SQ=-1;ID=S2505_raw:GT=hom:DP=14:FQ=1:SQ=-1;ID=S2176_raw:GT=hom:DP=28:FQ=1:SQ=-1;ID=S2506_raw:GT=hom:DP=17:FQ=1:SQ=-1;ID=S2667_raw:GT=hom:DP=9:FQ=1:SQ=-1;
16	50092428	50092428	G	C	intergenic	CNEP1R1;HEATR3	.	dist=21429;dist=7424	rs7191715	.	ID=S2009_raw:GT=hom:DP=7:FQ=1:SQ=-1;ID=S2503_raw:GT=hom:DP=54:FQ=1:SQ=-1;ID=S2507_raw:GT=hom:DP=43:FQ=1:SQ=-1;ID=S2668_raw:GT=hom:DP=32:FQ=1:SQ=-1;ID=S2012_raw:GT=hom:DP=5:FQ=1:SQ=-1;ID=S2010_raw:GT=hom:DP=6:FQ=1:SQ=-1;ID=S2662_raw:GT=hom:DP=28:FQ=1:SQ=-1;ID=S2663_raw:GT=hom:DP=27:FQ=1:SQ=-1;ID=S2011_raw:GT=hom:DP=3:FQ=1:SQ=-1;ID=S2504_raw:GT=hom:DP=61:FQ=1:SQ=-1;ID=S2008_raw:GT=hom:DP=7:FQ=1:SQ=-1;ID=S2509_raw:GT=hom:DP=36:FQ=1:SQ=-1;ID=S2508_raw:GT=hom:DP=33:FQ=1:SQ=-1;ID=S2666_raw:GT=hom:DP=28:FQ=1:SQ=-1;ID=S2669_raw:GT=hom:DP=29:FQ=1:SQ=-1;ID=S2166_raw:GT=hom:DP=15:FQ=1:SQ=-1;ID=S1775_raw:GT=hom:DP=11:FQ=1:SQ=-1;ID=S2501_raw:GT=hom:DP=47:FQ=1:SQ=-1;ID=S2500_raw:GT=hom:DP=25:FQ=1:SQ=-1;ID=S2502_raw:GT=hom:DP=41:FQ=1:SQ=-1;ID=S2505_raw:GT=hom:DP=45:FQ=1:SQ=-1;ID=S2176_raw:GT=hom:DP=23:FQ=1:SQ=-1;ID=S2506_raw:GT=hom:DP=41:FQ=1:SQ=-1;ID=S2667_raw:GT=hom:DP=25:FQ=1:SQ=-1;
16	50092580	50092590	TGAAGCTACAG	-	intergenic	CNEP1R1;HEATR3	.	dist=21581;dist=7262	.	.	ID=S2009_raw:GT=hom:DP=2:FQ=1:SQ=-1;ID=S2503_raw:GT=hom:DP=33:FQ=1:SQ=-1;ID=S2507_raw:GT=hom:DP=25:FQ=1:SQ=-1;ID=S2668_raw:GT=hom:DP=21:FQ=1:SQ=-1;ID=S2012_raw:GT=hom:DP=2:FQ=1:SQ=-1;ID=S2010_raw:GT=hom:DP=2:FQ=1:SQ=-1;ID=S2662_raw:GT=hom:DP=12:FQ=1:SQ=-1;ID=S2663_raw:GT=hom:DP=12:FQ=1:SQ=-1;ID=S2011_raw:GT=hom:DP=5:FQ=1:SQ=-1;ID=S2504_raw:GT=hom:DP=32:FQ=1:SQ=-1;ID=S2008_raw:GT=hom:DP=3:FQ=1:SQ=-1;ID=S2509_raw:GT=hom:DP=25:FQ=1:SQ=-1;ID=S2508_raw:GT=hom:DP=24:FQ=1:SQ=-1;ID=S2666_raw:GT=hom:DP=16:FQ=1:SQ=-1;ID=S2669_raw:GT=hom:DP=18:FQ=1:SQ=-1;ID=S2166_raw:GT=hom:DP=12:FQ=1:SQ=-1;ID=S1775_raw:GT=hom:DP=3:FQ=1:SQ=-1;ID=S2501_raw:GT=hom:DP=26:FQ=1:SQ=-1;ID=S2500_raw:GT=hom:DP=22:FQ=1:SQ=-1;ID=S2502_raw:GT=hom:DP=29:FQ=1:SQ=-1;ID=S2505_raw:GT=hom:DP=23:FQ=1:SQ=-1;ID=S2176_raw:GT=hom:DP=12:FQ=1:SQ=-1;ID=S2506_raw:GT=hom:DP=24:FQ=1:SQ=-1;ID=S2667_raw:GT=hom:DP=22:FQ=1:SQ=-1;
17	71197200	71197200	T	C	intronic	COG1	.	.	rs1026130	.	ID=S2009_raw:GT=hom:DP=19:FQ=1:SQ=-1;ID=S2503_raw:GT=hom:DP=28:FQ=1:SQ=-1;ID=S2507_raw:GT=hom:DP=20:FQ=1:SQ=-1;ID=S2668_raw:GT=hom:DP=15:FQ=1:SQ=-1;ID=S2012_raw:GT=hom:DP=12:FQ=1:SQ=-1;ID=S2010_raw:GT=hom:DP=10:FQ=1:SQ=-1;ID=S2662_raw:GT=hom:DP=2:FQ=1:SQ=-1;ID=S2663_raw:GT=hom:DP=14:FQ=1:SQ=-1;ID=S2011_raw:GT=hom:DP=19:FQ=1:SQ=-1;ID=S2504_raw:GT=hom:DP=21:FQ=1:SQ=-1;ID=S2008_raw:GT=hom:DP=19:FQ=1:SQ=-1;ID=S2509_raw:GT=hom:DP=11:FQ=1:SQ=-1;ID=S2508_raw:GT=hom:DP=22:FQ=1:SQ=-1;ID=S2666_raw:GT=hom:DP=11:FQ=1:SQ=-1;ID=S2669_raw:GT=hom:DP=9:FQ=1:SQ=-1;ID=S2166_raw:GT=hom:DP=21:FQ=1:SQ=-1;ID=S1775_raw:GT=hom:DP=20:FQ=1:SQ=-1;ID=S2501_raw:GT=hom:DP=20:FQ=1:SQ=-1;ID=S2500_raw:GT=hom:DP=14:FQ=1:SQ=-1;ID=S2502_raw:GT=hom:DP=18:FQ=1:SQ=-1;ID=S2505_raw:GT=hom:DP=16:FQ=1:SQ=-1;ID=S2176_raw:GT=hom:DP=27:FQ=1:SQ=-1;ID=S2506_raw:GT=hom:DP=17:FQ=1:SQ=-1;ID=S2667_raw:GT=hom:DP=17:FQ=1:SQ=-1;
17	71202718	71202718	T	C	intronic	COG1	.	.	rs7209284	.	ID=S2009_raw:GT=hom:DP=82:FQ=0.99:SQ=-1;ID=S2503_raw:GT=hom:DP=3:FQ=1:SQ=-1;ID=S2507_raw:GT=hom:DP=4:FQ=1:SQ=-1;ID=S2668_raw:GT=hom:DP=8:FQ=1:SQ=-1;ID=S2012_raw:GT=hom:DP=93:FQ=1:SQ=-1;ID=S2010_raw:GT=hom:DP=117:FQ=1:SQ=-1;ID=S2662_raw:GT=hom:DP=12:FQ=1:SQ=-1;ID=S2663_raw:GT=hom:DP=2:FQ=1:SQ=-1;ID=S2011_raw:GT=hom:DP=108:FQ=1:SQ=-1;ID=S2504_raw:GT=hom:DP=8:FQ=1:SQ=-1;ID=S2008_raw:GT=hom:DP=110:FQ=1:SQ=-1;ID=S2509_raw:GT=hom:DP=5:FQ=1:SQ=-1;ID=S2508_raw:GT=hom:DP=4:FQ=1:SQ=-1;ID=S2666_raw:GT=hom:DP=10:FQ=1:SQ=-1;ID=S2669_raw:GT=hom:DP=7:FQ=1:SQ=-1;ID=S2166_raw:GT=hom:DP=8:FQ=1:SQ=-1;ID=S1775_raw:GT=hom:DP=4:FQ=1:SQ=-1;ID=S2501_raw:GT=hom:DP=6:FQ=1:SQ=-1;ID=S2500_raw:GT=hom:DP=5:FQ=1:SQ=-1;ID=S2502_raw:GT=hom:DP=4:FQ=1:SQ=-1;ID=S2505_raw:GT=hom:DP=5:FQ=1:SQ=-1;ID=S2176_raw:GT=hom:DP=6:FQ=1:SQ=-1;ID=S2506_raw:GT=hom:DP=6:FQ=1:SQ=-1;ID=S2667_raw:GT=hom:DP=9:FQ=1:SQ=-1;
13	40377536	40377536	C	T	intergenic	COG6;LINC00332	.	dist=11734;dist=378410	rs7326741	.	ID=S2009_raw:GT=hom:DP=4:FQ=1:SQ=-1;ID=S2503_raw:GT=hom:DP=11:FQ=1:SQ=-1;ID=S2507_raw:GT=hom:DP=10:FQ=1:SQ=-1;ID=S2668_raw:GT=hom:DP=19:FQ=1:SQ=-1;ID=S2012_raw:GT=hom:DP=1:FQ=1:SQ=-1;ID=S2010_raw:GT=hom:DP=4:FQ=1:SQ=-1;ID=S2662_raw:GT=hom:DP=2:FQ=1:SQ=-1;ID=S2663_raw:GT=hom:DP=19:FQ=1:SQ=-1;ID=S2011_raw:GT=hom:DP=6:FQ=1:SQ=-1;ID=S2504_raw:GT=hom:DP=11:FQ=1:SQ=-1;ID=S2008_raw:GT=hom:DP=5:FQ=1:SQ=-1;ID=S2509_raw:GT=hom:DP=9:FQ=1:SQ=-1;ID=S2508_raw:GT=hom:DP=13:FQ=1:SQ=-1;ID=S2666_raw:GT=hom:DP=12:FQ=1:SQ=-1;ID=S2669_raw:GT=hom:DP=6:FQ=1:SQ=-1;ID=S2166_raw:GT=hom:DP=2:FQ=1:SQ=-1;ID=S1775_raw:GT=hom:DP=5:FQ=1:SQ=-1;ID=S2501_raw:GT=hom:DP=5:FQ=1:SQ=-1;ID=S2500_raw:GT=hom:DP=7:FQ=1:SQ=-1;ID=S2502_raw:GT=hom:DP=18:FQ=1:SQ=-1;ID=S2505_raw:GT=hom:DP=4:FQ=1:SQ=-1;ID=S2176_raw:GT=hom:DP=6:FQ=1:SQ=-1;ID=S2506_raw:GT=hom:DP=11:FQ=1:SQ=-1;ID=S2667_raw:GT=hom:DP=6:FQ=1:SQ=-1;
16	23400494	23400494	C	A	intronic	COG7	.	.	rs250586	.	ID=S2009_raw:GT=hom:DP=3:FQ=1:SQ=-1;ID=S2503_raw:GT=hom:DP=24:FQ=1:SQ=-1;ID=S2507_raw:GT=hom:DP=26:FQ=1:SQ=-1;ID=S2668_raw:GT=hom:DP=18:FQ=1:SQ=-1;ID=S2012_raw:GT=hom:DP=5:FQ=1:SQ=-1;ID=S2010_raw:GT=hom:DP=6:FQ=1:SQ=-1;ID=S2662_raw:GT=hom:DP=18:FQ=1:SQ=-1;ID=S2663_raw:GT=hom:DP=19:FQ=1:SQ=-1;ID=S2011_raw:GT=hom:DP=4:FQ=1:SQ=-1;ID=S2504_raw:GT=hom:DP=17:FQ=1:SQ=-1;ID=S2008_raw:GT=hom:DP=2:FQ=1:SQ=-1;ID=S2509_raw:GT=hom:DP=20:FQ=1:SQ=-1;ID=S2508_raw:GT=hom:DP=15:FQ=1:SQ=-1;ID=S2666_raw:GT=hom:DP=14:FQ=1:SQ=-1;ID=S2669_raw:GT=hom:DP=13:FQ=1:SQ=-1;ID=S2166_raw:GT=hom:DP=22:FQ=1:SQ=-1;ID=S1775_raw:GT=hom:DP=23:FQ=1:SQ=-1;ID=S2501_raw:GT=hom:DP=17:FQ=1:SQ=-1;ID=S2500_raw:GT=hom:DP=31:FQ=1:SQ=-1;ID=S2502_raw:GT=hom:DP=22:FQ=1:SQ=-1;ID=S2505_raw:GT=hom:DP=24:FQ=1:SQ=-1;ID=S2176_raw:GT=hom:DP=21:FQ=1:SQ=-1;ID=S2506_raw:GT=hom:DP=27:FQ=1:SQ=-1;ID=S2667_raw:GT=hom:DP=18:FQ=1:SQ=-1;
2	1,9E+08	1,9E+08	T	G	exonic	COL3A1	nonsynonymous SNV	COL3A1:NM_000090:exon50:c.4059T>G:p.H1353Q	rs1516446	Benign	ID=S2009_raw:GT=hom:DP=45:FQ=1:SQ=-1;ID=S2503_raw:GT=hom:DP=93:FQ=1:SQ=-1;ID=S2507_raw:GT=hom:DP=108:FQ=1:SQ=-1;ID=S2668_raw:GT=hom:DP=119:FQ=1:SQ=-1;ID=S2012_raw:GT=hom:DP=75:FQ=1:SQ=-1;ID=S2010_raw:GT=hom:DP=74:FQ=1:SQ=-1;ID=S2662_raw:GT=hom:DP=9:FQ=1:SQ=-1;ID=S2663_raw:GT=hom:DP=111:FQ=1:SQ=-1;ID=S2011_raw:GT=hom:DP=108:FQ=1:SQ=-1;ID=S2504_raw:GT=hom:DP=116:FQ=1:SQ=-1;ID=S2008_raw:GT=hom:DP=59:FQ=1:SQ=-1;ID=S2509_raw:GT=hom:DP=98:FQ=1:SQ=-1;ID=S2508_raw:GT=hom:DP=135:FQ=1:SQ=-1;ID=S2666_raw:GT=hom:DP=100:FQ=1:SQ=-1;ID=S2669_raw:GT=hom:DP=73:FQ=1:SQ=-1;ID=S2166_raw:GT=hom:DP=217:FQ=1:SQ=-1;ID=S1775_raw:GT=hom:DP=132:FQ=1:SQ=-1;ID=S2501_raw:GT=hom:DP=69:FQ=1:SQ=-1;ID=S2500_raw:GT=hom:DP=91:FQ=1:SQ=-1;ID=S2502_raw:GT=hom:DP=101:FQ=1:SQ=-1;ID=S2505_raw:GT=hom:DP=107:FQ=1:SQ=-1;ID=S2176_raw:GT=hom:DP=217:FQ=1:SQ=-1;ID=S2506_raw:GT=hom:DP=77:FQ=1:SQ=-1;ID=S2667_raw:GT=hom:DP=82:FQ=1:SQ=-1;
13	1,11E+08	1,11E+08	T	G	exonic	COL4A1	nonsynonymous SNV	COL4A1:NM_001845:exon25:c.1663A>C:p.T555P	rs536174	Benign	ID=S2009_raw:GT=hom:DP=7:FQ=1:SQ=-1;ID=S2503_raw:GT=hom:DP=97:FQ=1:SQ=-1;ID=S2507_raw:GT=hom:DP=86:FQ=1:SQ=-1;ID=S2668_raw:GT=hom:DP=137:FQ=1:SQ=-1;ID=S2012_raw:GT=hom:DP=6:FQ=1:SQ=-1;ID=S2010_raw:GT=hom:DP=7:FQ=1:SQ=-1;ID=S2662_raw:GT=hom:DP=113:FQ=1:SQ=-1;ID=S2663_raw:GT=hom:DP=128:FQ=1:SQ=-1;ID=S2011_raw:GT=hom:DP=8:FQ=1:SQ=-1;ID=S2504_raw:GT=hom:DP=137:FQ=1:SQ=-1;ID=S2008_raw:GT=hom:DP=9:FQ=1:SQ=-1;ID=S2509_raw:GT=hom:DP=87:FQ=1:SQ=-1;ID=S2508_raw:GT=hom:DP=111:FQ=1:SQ=-1;ID=S2666_raw:GT=hom:DP=86:FQ=1:SQ=-1;ID=S2669_raw:GT=hom:DP=115:FQ=1:SQ=-1;ID=S2166_raw:GT=hom:DP=85:FQ=1:SQ=-1;ID=S1775_raw:GT=hom:DP=45:FQ=1:SQ=-1;ID=S2501_raw:GT=hom:DP=98:FQ=1:SQ=-1;ID=S2500_raw:GT=hom:DP=115:FQ=1:SQ=-1;ID=S2502_raw:GT=hom:DP=71:FQ=1:SQ=-1;ID=S2505_raw:GT=hom:DP=105:FQ=1:SQ=-1;ID=S2176_raw:GT=hom:DP=89:FQ=1:SQ=-1;ID=S2506_raw:GT=hom:DP=88:FQ=1:SQ=-1;ID=S2667_raw:GT=hom:DP=91:FQ=1:SQ=-1;
13	1,11E+08	1,11E+08	A	G	intronic	COL4A1	.	.	rs598819	.	ID=S2009_raw:GT=hom:DP=50:FQ=1:SQ=-1;ID=S2503_raw:GT=hom:DP=69:FQ=1:SQ=-1;ID=S2507_raw:GT=hom:DP=96:FQ=1:SQ=-1;ID=S2668_raw:GT=hom:DP=94:FQ=1:SQ=-1;ID=S2012_raw:GT=hom:DP=65:FQ=1:SQ=-1;ID=S2010_raw:GT=hom:DP=67:FQ=1:SQ=-1;ID=S2662_raw:GT=hom:DP=29:FQ=1:SQ=-1;ID=S2663_raw:GT=hom:DP=72:FQ=1:SQ=-1;ID=S2011_raw:GT=hom:DP=79:FQ=1:SQ=-1;ID=S2504_raw:GT=hom:DP=84:FQ=1:SQ=-1;ID=S2008_raw:GT=hom:DP=65:FQ=1:SQ=-1;ID=S2509_raw:GT=hom:DP=55:FQ=1:SQ=-1;ID=S2508_raw:GT=hom:DP=71:FQ=1:SQ=-1;ID=S2666_raw:GT=hom:DP=73:FQ=1:SQ=-1;ID=S2669_raw:GT=hom:DP=66:FQ=1:SQ=-1;ID=S2166_raw:GT=hom:DP=127:FQ=1:SQ=-1;ID=S1775_raw:GT=hom:DP=68:FQ=1:SQ=-1;ID=S2501_raw:GT=hom:DP=66:FQ=1:SQ=-1;ID=S2500_raw:GT=hom:DP=61:FQ=1:SQ=-1;ID=S2502_raw:GT=hom:DP=104:FQ=1:SQ=-1;ID=S2505_raw:GT=hom:DP=64:FQ=1:SQ=-1;ID=S2176_raw:GT=hom:DP=137:FQ=1:SQ=-1;ID=S2506_raw:GT=hom:DP=71:FQ=1:SQ=-1;ID=S2667_raw:GT=hom:DP=73:FQ=1:SQ=-1;
X	1,07E+08	1,07E+08	T	C	intronic	COL4A6	.	.	rs2881142	.	ID=S2009_raw:GT=hom:DP=4:FQ=1:SQ=-1;ID=S2503_raw:GT=hom:DP=16:FQ=1:SQ=-1;ID=S2507_raw:GT=hom:DP=17:FQ=1:SQ=-1;ID=S2668_raw:GT=hom:DP=41:FQ=1:SQ=-1;ID=S2012_raw:GT=hom:DP=6:FQ=1:SQ=-1;ID=S2010_raw:GT=hom:DP=4:FQ=1:SQ=-1;ID=S2662_raw:GT=hom:DP=27:FQ=1:SQ=-1;ID=S2663_raw:GT=hom:DP=35:FQ=1:SQ=-1;ID=S2011_raw:GT=hom:DP=4:FQ=1:SQ=-1;ID=S2504_raw:GT=hom:DP=13:FQ=1:SQ=-1;ID=S2008_raw:GT=hom:DP=6:FQ=1:SQ=-1;ID=S2509_raw:GT=hom:DP=18:FQ=1:SQ=-1;ID=S2508_raw:GT=hom:DP=23:FQ=1:SQ=-1;ID=S2666_raw:GT=hom:DP=16:FQ=1:SQ=-1;ID=S2669_raw:GT=hom:DP=19:FQ=1:SQ=-1;ID=S2166_raw:GT=hom:DP=26:FQ=0.96:SQ=-1;ID=S1775_raw:GT=hom:DP=28:FQ=1:SQ=-1;ID=S2501_raw:GT=hom:DP=9:FQ=1:SQ=-1;ID=S2500_raw:GT=hom:DP=18:FQ=1:SQ=-1;ID=S2502_raw:GT=hom:DP=25:FQ=1:SQ=-1;ID=S2505_raw:GT=hom:DP=15:FQ=1:SQ=-1;ID=S2176_raw:GT=hom:DP=28:FQ=1:SQ=-1;ID=S2506_raw:GT=hom:DP=18:FQ=1:SQ=-1;ID=S2667_raw:GT=hom:DP=43:FQ=1:SQ=-1;
X	1,07E+08	1,07E+08	A	G	exonic	COL4A6	synonymous SNV	COL4A6:NM_001287759:exon29:c.2808T>C:p.R936R,COL4A6:NM_001287760:exon29:c.2808T>C:p.R936R,COL4A6:NM_001847:exon29:c.2811T>C:p.R937R,COL4A6:NM_033641:exon29:c.2808T>C:p.R936R,COL4A6:NM_001287758:exon30:c.2859T>C:p.R953R	rs4623610	Benign	ID=S2009_raw:GT=hom:DP=11:FQ=1:SQ=-1;ID=S2503_raw:GT=hom:DP=44:FQ=1:SQ=-1;ID=S2507_raw:GT=hom:DP=38:FQ=1:SQ=-1;ID=S2668_raw:GT=hom:DP=124:FQ=1:SQ=-1;ID=S2012_raw:GT=hom:DP=20:FQ=1:SQ=-1;ID=S2010_raw:GT=hom:DP=35:FQ=1:SQ=-1;ID=S2662_raw:GT=hom:DP=29:FQ=1:SQ=-1;ID=S2663_raw:GT=hom:DP=75:FQ=1:SQ=-1;ID=S2011_raw:GT=hom:DP=25:FQ=1:SQ=-1;ID=S2504_raw:GT=hom:DP=56:FQ=1:SQ=-1;ID=S2008_raw:GT=hom:DP=28:FQ=1:SQ=-1;ID=S2509_raw:GT=hom:DP=42:FQ=1:SQ=-1;ID=S2508_raw:GT=hom:DP=58:FQ=1:SQ=-1;ID=S2666_raw:GT=hom:DP=49:FQ=1:SQ=-1;ID=S2669_raw:GT=hom:DP=46:FQ=1:SQ=-1;ID=S2166_raw:GT=hom:DP=95:FQ=0.99:SQ=-1;ID=S1775_raw:GT=hom:DP=67:FQ=1:SQ=-1;ID=S2501_raw:GT=hom:DP=31:FQ=1:SQ=-1;ID=S2500_raw:GT=hom:DP=47:FQ=1:SQ=-1;ID=S2502_raw:GT=hom:DP=89:FQ=1:SQ=-1;ID=S2505_raw:GT=hom:DP=51:FQ=1:SQ=-1;ID=S2176_raw:GT=hom:DP=108:FQ=1:SQ=-1;ID=S2506_raw:GT=hom:DP=43:FQ=1:SQ=-1;ID=S2667_raw:GT=hom:DP=103:FQ=1:SQ=-1;
X	1,07E+08	1,07E+08	C	T	intronic	COL4A6	.	.	rs1266736	.	ID=S2009_raw:GT=hom:DP=4:FQ=1:SQ=-1;ID=S2503_raw:GT=hom:DP=27:FQ=1:SQ=-1;ID=S2507_raw:GT=hom:DP=17:FQ=1:SQ=-1;ID=S2668_raw:GT=hom:DP=17:FQ=1:SQ=-1;ID=S2012_raw:GT=hom:DP=5:FQ=1:SQ=-1;ID=S2010_raw:GT=hom:DP=8:FQ=1:SQ=-1;ID=S2662_raw:GT=hom:DP=2:FQ=1:SQ=-1;ID=S2663_raw:GT=hom:DP=37:FQ=1:SQ=-1;ID=S2011_raw:GT=hom:DP=6:FQ=1:SQ=-1;ID=S2504_raw:GT=hom:DP=8:FQ=1:SQ=-1;ID=S2008_raw:GT=hom:DP=16:FQ=1:SQ=-1;ID=S2509_raw:GT=hom:DP=21:FQ=1:SQ=-1;ID=S2508_raw:GT=hom:DP=14:FQ=1:SQ=-1;ID=S2666_raw:GT=hom:DP=13:FQ=1:SQ=-1;ID=S2669_raw:GT=hom:DP=12:FQ=1:SQ=-1;ID=S2166_raw:GT=hom:DP=19:FQ=1:SQ=-1;ID=S1775_raw:GT=hom:DP=9:FQ=1:SQ=-1;ID=S2501_raw:GT=hom:DP=21:FQ=1:SQ=-1;ID=S2500_raw:GT=hom:DP=12:FQ=1:SQ=-1;ID=S2502_raw:GT=hom:DP=36:FQ=1:SQ=-1;ID=S2505_raw:GT=hom:DP=16:FQ=1:SQ=-1;ID=S2176_raw:GT=hom:DP=10:FQ=1:SQ=-1;ID=S2506_raw:GT=hom:DP=4:FQ=1:SQ=-1;ID=S2667_raw:GT=hom:DP=24:FQ=1:SQ=-1;
3	48602524	48602524	-	GCG	intronic	COL7A1	.	.	.	.	ID=S2009_raw:GT=hom:DP=22:FQ=1:SQ=-1;ID=S2503_raw:GT=hom:DP=108:FQ=1:SQ=-1;ID=S2507_raw:GT=hom:DP=141:FQ=1:SQ=-1;ID=S2668_raw:GT=hom:DP=147:FQ=1:SQ=-1;ID=S2012_raw:GT=hom:DP=25:FQ=1:SQ=-1;ID=S2010_raw:GT=hom:DP=29:FQ=1:SQ=-1;ID=S2662_raw:GT=hom:DP=202:FQ=1:SQ=-1;ID=S2663_raw:GT=hom:DP=106:FQ=1:SQ=-1;ID=S2011_raw:GT=hom:DP=57:FQ=1:SQ=-1;ID=S2504_raw:GT=hom:DP=136:FQ=1:SQ=-1;ID=S2008_raw:GT=hom:DP=37:FQ=1:SQ=-1;ID=S2509_raw:GT=hom:DP=111:FQ=1:SQ=-1;ID=S2508_raw:GT=hom:DP=134:FQ=1:SQ=-1;ID=S2666_raw:GT=hom:DP=127:FQ=1:SQ=-1;ID=S2669_raw:GT=hom:DP=131:FQ=1:SQ=-1;ID=S2166_raw:GT=hom:DP=123:FQ=1:SQ=-1;ID=S1775_raw:GT=hom:DP=48:FQ=1:SQ=-1;ID=S2501_raw:GT=hom:DP=105:FQ=1:SQ=-1;ID=S2500_raw:GT=hom:DP=150:FQ=1:SQ=-1;ID=S2502_raw:GT=hom:DP=139:FQ=1:SQ=-1;ID=S2505_raw:GT=hom:DP=133:FQ=1:SQ=-1;ID=S2176_raw:GT=hom:DP=108:FQ=1:SQ=-1;ID=S2506_raw:GT=hom:DP=120:FQ=1:SQ=-1;ID=S2667_raw:GT=hom:DP=106:FQ=1:SQ=-1;
21	46924440	46924448	CCCAGGCCC	-	exonic;splicing	COL18A1	nonframeshift deletion	COL18A1:NM_030582:exon34:c.3369_3377del:p.1123_1126del,COL18A1:NM_130444:exon34:c.4074_4082del:p.1358_1361del,COL18A1:NM_130445:exon35:c.2829_2837del:p.943_946del	.	.	ID=S2009_raw:GT=hom:DP=10:FQ=1:SQ=-1;ID=S2503_raw:GT=hom:DP=22:FQ=1:SQ=-1;ID=S2507_raw:GT=hom:DP=54:FQ=1:SQ=-1;ID=S2668_raw:GT=hom:DP=34:FQ=1:SQ=-1;ID=S2012_raw:GT=hom:DP=14:FQ=1:SQ=-1;ID=S2010_raw:GT=hom:DP=19:FQ=1:SQ=-1;ID=S2662_raw:GT=hom:DP=78:FQ=1:SQ=-1;ID=S2663_raw:GT=hom:DP=31:FQ=1:SQ=-1;ID=S2011_raw:GT=hom:DP=25:FQ=1:SQ=-1;ID=S2504_raw:GT=hom:DP=30:FQ=1:SQ=-1;ID=S2008_raw:GT=hom:DP=16:FQ=1:SQ=-1;ID=S2509_raw:GT=hom:DP=30:FQ=1:SQ=-1;ID=S2508_raw:GT=hom:DP=32:FQ=1:SQ=-1;ID=S2666_raw:GT=hom:DP=3:FQ=1:SQ=-1;ID=S2669_raw:GT=hom:DP=55:FQ=1:SQ=-1;ID=S2166_raw:GT=hom:DP=25:FQ=1:SQ=-1;ID=S1775_raw:GT=hom:DP=29:FQ=1:SQ=-1;ID=S2501_raw:GT=hom:DP=34:FQ=1:SQ=-1;ID=S2500_raw:GT=hom:DP=34:FQ=1:SQ=-1;ID=S2502_raw:GT=hom:DP=31:FQ=1:SQ=-1;ID=S2505_raw:GT=hom:DP=35:FQ=1:SQ=-1;ID=S2176_raw:GT=hom:DP=24:FQ=1:SQ=-1;ID=S2506_raw:GT=hom:DP=30:FQ=1:SQ=-1;ID=S2667_raw:GT=hom:DP=46:FQ=1:SQ=-1;
1	1,6E+08	1,6E+08	A	G	intronic	COPA	.	.	rs2066320	.	ID=S2009_raw:GT=hom:DP=64:FQ=1:SQ=-1;ID=S2503_raw:GT=hom:DP=149:FQ=1:SQ=-1;ID=S2507_raw:GT=hom:DP=134:FQ=1:SQ=-1;ID=S2668_raw:GT=hom:DP=237:FQ=1:SQ=-1;ID=S2012_raw:GT=hom:DP=80:FQ=1:SQ=-1;ID=S2010_raw:GT=hom:DP=98:FQ=1:SQ=-1;ID=S2662_raw:GT=hom:DP=58:FQ=1:SQ=-1;ID=S2663_raw:GT=hom:DP=168:FQ=1:SQ=-1;ID=S2011_raw:GT=hom:DP=133:FQ=1:SQ=-1;ID=S2504_raw:GT=hom:DP=153:FQ=1:SQ=-1;ID=S2008_raw:GT=hom:DP=81:FQ=1:SQ=-1;ID=S2509_raw:GT=hom:DP=140:FQ=1:SQ=-1;ID=S2508_raw:GT=hom:DP=155:FQ=1:SQ=-1;ID=S2666_raw:GT=hom:DP=260:FQ=1:SQ=-1;ID=S2669_raw:GT=hom:DP=144:FQ=1:SQ=-1;ID=S2166_raw:GT=hom:DP=151:FQ=1:SQ=-1;ID=S1775_raw:GT=hom:DP=111:FQ=1:SQ=-1;ID=S2501_raw:GT=hom:DP=139:FQ=1:SQ=-1;ID=S2500_raw:GT=hom:DP=155:FQ=1:SQ=-1;ID=S2502_raw:GT=hom:DP=171:FQ=1:SQ=-1;ID=S2505_raw:GT=hom:DP=144:FQ=1:SQ=-1;ID=S2176_raw:GT=hom:DP=158:FQ=1:SQ=-1;ID=S2506_raw:GT=hom:DP=119:FQ=1:SQ=-1;ID=S2667_raw:GT=hom:DP=146:FQ=1:SQ=-1;
19	41216172	41216172	T	C	intronic	COQ8B	.	.	rs7258157	.	ID=S2009_raw:GT=hom:DP=35:FQ=1:SQ=-1;ID=S2503_raw:GT=hom:DP=16:FQ=1:SQ=-1;ID=S2507_raw:GT=hom:DP=10:FQ=1:SQ=-1;ID=S2668_raw:GT=hom:DP=2:FQ=1:SQ=-1;ID=S2012_raw:GT=hom:DP=35:FQ=1:SQ=-1;ID=S2010_raw:GT=hom:DP=41:FQ=1:SQ=-1;ID=S2662_raw:GT=hom:DP=10:FQ=1:SQ=-1;ID=S2663_raw:GT=hom:DP=8:FQ=1:SQ=-1;ID=S2011_raw:GT=hom:DP=59:FQ=1:SQ=-1;ID=S2504_raw:GT=hom:DP=11:FQ=1:SQ=-1;ID=S2008_raw:GT=hom:DP=44:FQ=1:SQ=-1;ID=S2509_raw:GT=hom:DP=12:FQ=1:SQ=-1;ID=S2508_raw:GT=hom:DP=5:FQ=1:SQ=-1;ID=S2666_raw:GT=hom:DP=5:FQ=1:SQ=-1;ID=S2669_raw:GT=hom:DP=2:FQ=1:SQ=-1;ID=S2166_raw:GT=hom:DP=16:FQ=1:SQ=-1;ID=S1775_raw:GT=hom:DP=9:FQ=1:SQ=-1;ID=S2501_raw:GT=hom:DP=15:FQ=1:SQ=-1;ID=S2500_raw:GT=hom:DP=8:FQ=1:SQ=-1;ID=S2502_raw:GT=hom:DP=5:FQ=1:SQ=-1;ID=S2505_raw:GT=hom:DP=5:FQ=1:SQ=-1;ID=S2176_raw:GT=hom:DP=12:FQ=1:SQ=-1;ID=S2506_raw:GT=hom:DP=7:FQ=1:SQ=-1;ID=S2667_raw:GT=hom:DP=8:FQ=1:SQ=-1;
5	37247700	37247700	G	A	intronic	CPLANE1	.	.	rs6876576	Benign	ID=S2009_raw:GT=hom:DP=75:FQ=1:SQ=-1;ID=S2503_raw:GT=hom:DP=80:FQ=1:SQ=-1;ID=S2507_raw:GT=hom:DP=80:FQ=1:SQ=-1;ID=S2668_raw:GT=hom:DP=110:FQ=1:SQ=-1;ID=S2012_raw:GT=hom:DP=99:FQ=1:SQ=-1;ID=S2010_raw:GT=hom:DP=93:FQ=1:SQ=-1;ID=S2662_raw:GT=hom:DP=4:FQ=1:SQ=-1;ID=S2663_raw:GT=hom:DP=72:FQ=1:SQ=-1;ID=S2011_raw:GT=hom:DP=127:FQ=1:SQ=-1;ID=S2504_raw:GT=hom:DP=79:FQ=1:SQ=-1;ID=S2008_raw:GT=hom:DP=82:FQ=0.99:SQ=-1;ID=S2509_raw:GT=hom:DP=82:FQ=1:SQ=-1;ID=S2508_raw:GT=hom:DP=93:FQ=1:SQ=-1;ID=S2666_raw:GT=hom:DP=125:FQ=1:SQ=-1;ID=S2669_raw:GT=hom:DP=42:FQ=1:SQ=-1;ID=S2166_raw:GT=hom:DP=90:FQ=1:SQ=-1;ID=S1775_raw:GT=hom:DP=44:FQ=1:SQ=-1;ID=S2501_raw:GT=hom:DP=71:FQ=1:SQ=-1;ID=S2500_raw:GT=hom:DP=77:FQ=1:SQ=-1;ID=S2502_raw:GT=hom:DP=85:FQ=1:SQ=-1;ID=S2505_raw:GT=hom:DP=72:FQ=1:SQ=-1;ID=S2176_raw:GT=hom:DP=80:FQ=1:SQ=-1;ID=S2506_raw:GT=hom:DP=62:FQ=1:SQ=-1;ID=S2667_raw:GT=hom:DP=85:FQ=1:SQ=-1;
9	1,26E+08	1,26E+08	C	A	exonic	CRB2	nonsynonymous SNV	CRB2:NM_173689:exon2:c.269C>A:p.T90N	rs2808415	.	ID=S2009_raw:GT=hom:DP=69:FQ=1:SQ=-1;ID=S2503_raw:GT=hom:DP=133:FQ=1:SQ=-1;ID=S2507_raw:GT=hom:DP=143:FQ=1:SQ=-1;ID=S2668_raw:GT=hom:DP=197:FQ=1:SQ=-1;ID=S2012_raw:GT=hom:DP=70:FQ=1:SQ=-1;ID=S2010_raw:GT=hom:DP=89:FQ=1:SQ=-1;ID=S2662_raw:GT=hom:DP=270:FQ=1:SQ=-1;ID=S2663_raw:GT=hom:DP=158:FQ=1:SQ=-1;ID=S2011_raw:GT=hom:DP=108:FQ=1:SQ=-1;ID=S2504_raw:GT=hom:DP=171:FQ=1:SQ=-1;ID=S2008_raw:GT=hom:DP=95:FQ=1:SQ=-1;ID=S2509_raw:GT=hom:DP=128:FQ=1:SQ=-1;ID=S2508_raw:GT=hom:DP=141:FQ=1:SQ=-1;ID=S2666_raw:GT=hom:DP=125:FQ=1:SQ=-1;ID=S2669_raw:GT=hom:DP=136:FQ=1:SQ=-1;ID=S2166_raw:GT=hom:DP=117:FQ=1:SQ=-1;ID=S1775_raw:GT=hom:DP=62:FQ=1:SQ=-1;ID=S2501_raw:GT=hom:DP=117:FQ=1:SQ=-1;ID=S2500_raw:GT=hom:DP=132:FQ=1:SQ=-1;ID=S2502_raw:GT=hom:DP=157:FQ=1:SQ=-1;ID=S2505_raw:GT=hom:DP=102:FQ=1:SQ=-1;ID=S2176_raw:GT=hom:DP=103:FQ=0.99:SQ=-1;ID=S2506_raw:GT=hom:DP=113:FQ=1:SQ=-1;ID=S2667_raw:GT=hom:DP=134:FQ=1:SQ=-1;
9	1,26E+08	1,26E+08	T	C	intronic	CRB2	.	.	rs2488603	.	ID=S2009_raw:GT=hom:DP=15:FQ=1:SQ=-1;ID=S2503_raw:GT=hom:DP=20:FQ=1:SQ=-1;ID=S2507_raw:GT=hom:DP=18:FQ=1:SQ=-1;ID=S2668_raw:GT=hom:DP=31:FQ=1:SQ=-1;ID=S2012_raw:GT=hom:DP=17:FQ=1:SQ=-1;ID=S2010_raw:GT=hom:DP=17:FQ=1:SQ=-1;ID=S2662_raw:GT=hom:DP=61:FQ=1:SQ=-1;ID=S2663_raw:GT=hom:DP=28:FQ=1:SQ=-1;ID=S2011_raw:GT=hom:DP=19:FQ=1:SQ=-1;ID=S2504_raw:GT=hom:DP=28:FQ=1:SQ=-1;ID=S2008_raw:GT=hom:DP=14:FQ=1:SQ=-1;ID=S2509_raw:GT=hom:DP=23:FQ=1:SQ=-1;ID=S2508_raw:GT=hom:DP=44:FQ=1:SQ=-1;ID=S2666_raw:GT=hom:DP=32:FQ=1:SQ=-1;ID=S2669_raw:GT=hom:DP=34:FQ=1:SQ=-1;ID=S2166_raw:GT=hom:DP=19:FQ=1:SQ=-1;ID=S1775_raw:GT=hom:DP=14:FQ=1:SQ=-1;ID=S2501_raw:GT=hom:DP=18:FQ=1:SQ=-1;ID=S2500_raw:GT=hom:DP=22:FQ=1:SQ=-1;ID=S2502_raw:GT=hom:DP=24:FQ=1:SQ=-1;ID=S2505_raw:GT=hom:DP=21:FQ=1:SQ=-1;ID=S2176_raw:GT=hom:DP=22:FQ=1:SQ=-1;ID=S2506_raw:GT=hom:DP=33:FQ=1:SQ=-1;ID=S2667_raw:GT=hom:DP=30:FQ=1:SQ=-1;
9	1,26E+08	1,26E+08	T	C	exonic	CRB2	nonsynonymous SNV	CRB2:NM_173689:exon8:c.2126T>C:p.V709A	rs2488602	.	ID=S2009_raw:GT=hom:DP=74:FQ=1:SQ=-1;ID=S2503_raw:GT=hom:DP=187:FQ=1:SQ=-1;ID=S2507_raw:GT=hom:DP=183:FQ=1:SQ=-1;ID=S2668_raw:GT=hom:DP=171:FQ=1:SQ=-1;ID=S2012_raw:GT=hom:DP=83:FQ=1:SQ=-1;ID=S2010_raw:GT=hom:DP=79:FQ=1:SQ=-1;ID=S2662_raw:GT=hom:DP=302:FQ=1:SQ=-1;ID=S2663_raw:GT=hom:DP=169:FQ=1:SQ=-1;ID=S2011_raw:GT=hom:DP=89:FQ=1:SQ=-1;ID=S2504_raw:GT=hom:DP=197:FQ=1:SQ=-1;ID=S2008_raw:GT=hom:DP=72:FQ=1:SQ=-1;ID=S2509_raw:GT=hom:DP=179:FQ=1:SQ=-1;ID=S2508_raw:GT=hom:DP=184:FQ=1:SQ=-1;ID=S2666_raw:GT=hom:DP=115:FQ=1:SQ=-1;ID=S2669_raw:GT=hom:DP=174:FQ=1:SQ=-1;ID=S2166_raw:GT=hom:DP=131:FQ=1:SQ=-1;ID=S1775_raw:GT=hom:DP=111:FQ=0.99:SQ=-1;ID=S2501_raw:GT=hom:DP=172:FQ=1:SQ=-1;ID=S2500_raw:GT=hom:DP=190:FQ=1:SQ=-1;ID=S2502_raw:GT=hom:DP=192:FQ=1:SQ=-1;ID=S2505_raw:GT=hom:DP=179:FQ=1:SQ=-1;ID=S2176_raw:GT=hom:DP=152:FQ=1:SQ=-1;ID=S2506_raw:GT=hom:DP=168:FQ=1:SQ=-1;ID=S2667_raw:GT=hom:DP=218:FQ=1:SQ=-1;
9	1,26E+08	1,26E+08	A	G	exonic	CRB2	nonsynonymous SNV	CRB2:NM_173689:exon10:c.2905A>G:p.T969A	rs2488601	.	ID=S2009_raw:GT=hom:DP=14:FQ=1:SQ=-1;ID=S2503_raw:GT=hom:DP=76:FQ=1:SQ=-1;ID=S2507_raw:GT=hom:DP=100:FQ=1:SQ=-1;ID=S2668_raw:GT=hom:DP=83:FQ=1:SQ=-1;ID=S2012_raw:GT=hom:DP=13:FQ=1:SQ=-1;ID=S2010_raw:GT=hom:DP=10:FQ=1:SQ=-1;ID=S2662_raw:GT=hom:DP=144:FQ=1:SQ=-1;ID=S2663_raw:GT=hom:DP=104:FQ=1:SQ=-1;ID=S2011_raw:GT=hom:DP=18:FQ=1:SQ=-1;ID=S2504_raw:GT=hom:DP=72:FQ=1:SQ=-1;ID=S2008_raw:GT=hom:DP=12:FQ=1:SQ=-1;ID=S2509_raw:GT=hom:DP=70:FQ=1:SQ=-1;ID=S2508_raw:GT=hom:DP=130:FQ=1:SQ=-1;ID=S2666_raw:GT=hom:DP=24:FQ=1:SQ=-1;ID=S2669_raw:GT=hom:DP=110:FQ=1:SQ=-1;ID=S2166_raw:GT=hom:DP=77:FQ=1:SQ=-1;ID=S1775_raw:GT=hom:DP=23:FQ=1:SQ=-1;ID=S2501_raw:GT=hom:DP=77:FQ=1:SQ=-1;ID=S2500_raw:GT=hom:DP=74:FQ=1:SQ=-1;ID=S2502_raw:GT=hom:DP=96:FQ=1:SQ=-1;ID=S2505_raw:GT=hom:DP=102:FQ=1:SQ=-1;ID=S2176_raw:GT=hom:DP=56:FQ=1:SQ=-1;ID=S2506_raw:GT=hom:DP=95:FQ=1:SQ=-1;ID=S2667_raw:GT=hom:DP=146:FQ=1:SQ=-1;
3	9975353	9975353	A	G	upstream;downstream	CRELD1;IL17RC	.	dist=171;dist=48	rs279551	.	ID=S2009_raw:GT=hom:DP=4:FQ=1:SQ=-1;ID=S2503_raw:GT=hom:DP=12:FQ=1:SQ=-1;ID=S2507_raw:GT=hom:DP=8:FQ=1:SQ=-1;ID=S2668_raw:GT=hom:DP=2:FQ=1:SQ=-1;ID=S2012_raw:GT=hom:DP=6:FQ=1:SQ=-1;ID=S2010_raw:GT=hom:DP=7:FQ=1:SQ=-1;ID=S2662_raw:GT=hom:DP=18:FQ=0.94:SQ=-1;ID=S2663_raw:GT=hom:DP=4:FQ=1:SQ=-1;ID=S2011_raw:GT=hom:DP=10:FQ=1:SQ=-1;ID=S2504_raw:GT=hom:DP=9:FQ=1:SQ=-1;ID=S2008_raw:GT=hom:DP=10:FQ=1:SQ=-1;ID=S2509_raw:GT=hom:DP=8:FQ=1:SQ=-1;ID=S2508_raw:GT=hom:DP=5:FQ=1:SQ=-1;ID=S2666_raw:GT=hom:DP=3:FQ=1:SQ=-1;ID=S2669_raw:GT=hom:DP=8:FQ=1:SQ=-1;ID=S2166_raw:GT=hom:DP=6:FQ=1:SQ=-1;ID=S1775_raw:GT=hom:DP=8:FQ=1:SQ=-1;ID=S2501_raw:GT=hom:DP=9:FQ=1:SQ=-1;ID=S2500_raw:GT=hom:DP=5:FQ=1:SQ=-1;ID=S2502_raw:GT=hom:DP=6:FQ=1:SQ=-1;ID=S2505_raw:GT=hom:DP=13:FQ=1:SQ=-1;ID=S2176_raw:GT=hom:DP=18:FQ=1:SQ=-1;ID=S2506_raw:GT=hom:DP=13:FQ=1:SQ=-1;ID=S2667_raw:GT=hom:DP=2:FQ=1:SQ=-1;
2	2,05E+08	2,05E+08	A	G	intronic	CTLA4	.	.	rs231776	.	ID=S2009_raw:GT=hom:DP=7:FQ=1:SQ=-1;ID=S2503_raw:GT=hom:DP=78:FQ=1:SQ=-1;ID=S2507_raw:GT=hom:DP=92:FQ=1:SQ=-1;ID=S2668_raw:GT=hom:DP=88:FQ=1:SQ=-1;ID=S2012_raw:GT=hom:DP=5:FQ=1:SQ=-1;ID=S2010_raw:GT=hom:DP=5:FQ=1:SQ=-1;ID=S2662_raw:GT=hom:DP=48:FQ=1:SQ=-1;ID=S2663_raw:GT=hom:DP=92:FQ=1:SQ=-1;ID=S2011_raw:GT=hom:DP=8:FQ=1:SQ=-1;ID=S2504_raw:GT=hom:DP=99:FQ=1:SQ=-1;ID=S2008_raw:GT=hom:DP=2:FQ=1:SQ=-1;ID=S2509_raw:GT=hom:DP=87:FQ=1:SQ=-1;ID=S2508_raw:GT=hom:DP=104:FQ=1:SQ=-1;ID=S2666_raw:GT=hom:DP=55:FQ=1:SQ=-1;ID=S2669_raw:GT=hom:DP=105:FQ=1:SQ=-1;ID=S2166_raw:GT=hom:DP=155:FQ=1:SQ=-1;ID=S1775_raw:GT=hom:DP=91:FQ=1:SQ=-1;ID=S2501_raw:GT=hom:DP=75:FQ=1:SQ=-1;ID=S2500_raw:GT=hom:DP=102:FQ=1:SQ=-1;ID=S2502_raw:GT=hom:DP=84:FQ=1:SQ=-1;ID=S2505_raw:GT=hom:DP=97:FQ=1:SQ=-1;ID=S2176_raw:GT=hom:DP=155:FQ=1:SQ=-1;ID=S2506_raw:GT=hom:DP=74:FQ=1:SQ=-1;ID=S2667_raw:GT=hom:DP=97:FQ=1:SQ=-1;
17	3553526	3553526	A	G	intronic	CTNS	.	.	rs161350	.	ID=S2009_raw:GT=hom:DP=71:FQ=1:SQ=-1;ID=S2503_raw:GT=hom:DP=69:FQ=1:SQ=-1;ID=S2507_raw:GT=hom:DP=60:FQ=1:SQ=-1;ID=S2668_raw:GT=hom:DP=107:FQ=1:SQ=-1;ID=S2012_raw:GT=hom:DP=92:FQ=1:SQ=-1;ID=S2010_raw:GT=hom:DP=69:FQ=1:SQ=-1;ID=S2662_raw:GT=hom:DP=30:FQ=1:SQ=-1;ID=S2663_raw:GT=hom:DP=76:FQ=1:SQ=-1;ID=S2011_raw:GT=hom:DP=85:FQ=1:SQ=-1;ID=S2504_raw:GT=hom:DP=67:FQ=1:SQ=-1;ID=S2008_raw:GT=hom:DP=73:FQ=1:SQ=-1;ID=S2509_raw:GT=hom:DP=60:FQ=1:SQ=-1;ID=S2508_raw:GT=hom:DP=53:FQ=1:SQ=-1;ID=S2666_raw:GT=hom:DP=112:FQ=1:SQ=-1;ID=S2669_raw:GT=hom:DP=56:FQ=1:SQ=-1;ID=S2166_raw:GT=hom:DP=119:FQ=1:SQ=-1;ID=S1775_raw:GT=hom:DP=50:FQ=1:SQ=-1;ID=S2501_raw:GT=hom:DP=63:FQ=1:SQ=-1;ID=S2500_raw:GT=hom:DP=57:FQ=1:SQ=-1;ID=S2502_raw:GT=hom:DP=70:FQ=1:SQ=-1;ID=S2505_raw:GT=hom:DP=83:FQ=1:SQ=-1;ID=S2176_raw:GT=hom:DP=82:FQ=1:SQ=-1;ID=S2506_raw:GT=hom:DP=74:FQ=1:SQ=-1;ID=S2667_raw:GT=hom:DP=59:FQ=1:SQ=-1;
17	3558417	3558417	C	G	intronic	CTNS	.	.	rs459613	Benign	ID=S2009_raw:GT=hom:DP=118:FQ=1:SQ=-1;ID=S2503_raw:GT=hom:DP=112:FQ=1:SQ=-1;ID=S2507_raw:GT=hom:DP=105:FQ=1:SQ=-1;ID=S2668_raw:GT=hom:DP=128:FQ=1:SQ=-1;ID=S2012_raw:GT=hom:DP=76:FQ=1:SQ=-1;ID=S2010_raw:GT=hom:DP=84:FQ=1:SQ=-1;ID=S2662_raw:GT=hom:DP=204:FQ=1:SQ=-1;ID=S2663_raw:GT=hom:DP=93:FQ=1:SQ=-1;ID=S2011_raw:GT=hom:DP=109:FQ=1:SQ=-1;ID=S2504_raw:GT=hom:DP=122:FQ=1:SQ=-1;ID=S2008_raw:GT=hom:DP=91:FQ=1:SQ=-1;ID=S2509_raw:GT=hom:DP=126:FQ=1:SQ=-1;ID=S2508_raw:GT=hom:DP=103:FQ=1:SQ=-1;ID=S2666_raw:GT=hom:DP=122:FQ=1:SQ=-1;ID=S2669_raw:GT=hom:DP=100:FQ=1:SQ=-1;ID=S2166_raw:GT=hom:DP=97:FQ=1:SQ=-1;ID=S1775_raw:GT=hom:DP=78:FQ=1:SQ=-1;ID=S2501_raw:GT=hom:DP=102:FQ=1:SQ=-1;ID=S2500_raw:GT=hom:DP=111:FQ=1:SQ=-1;ID=S2502_raw:GT=hom:DP=121:FQ=1:SQ=-1;ID=S2505_raw:GT=hom:DP=113:FQ=1:SQ=-1;ID=S2176_raw:GT=hom:DP=131:FQ=1:SQ=-1;ID=S2506_raw:GT=hom:DP=105:FQ=1:SQ=-1;ID=S2667_raw:GT=hom:DP=127:FQ=1:SQ=-1;
10	16943371	16943371	G	C	exonic	CUBN	nonsynonymous SNV	CUBN:NM_001081:exon52:c.8150C>G:p.S2717W	rs2796835	Benign	ID=S2009_raw:GT=hom:DP=60:FQ=1:SQ=-1;ID=S2503_raw:GT=hom:DP=134:FQ=1:SQ=-1;ID=S2507_raw:GT=hom:DP=97:FQ=1:SQ=-1;ID=S2668_raw:GT=hom:DP=136:FQ=1:SQ=-1;ID=S2012_raw:GT=hom:DP=60:FQ=1:SQ=-1;ID=S2010_raw:GT=hom:DP=79:FQ=1:SQ=-1;ID=S2662_raw:GT=hom:DP=30:FQ=1:SQ=-1;ID=S2663_raw:GT=hom:DP=123:FQ=1:SQ=-1;ID=S2011_raw:GT=hom:DP=112:FQ=1:SQ=-1;ID=S2504_raw:GT=hom:DP=137:FQ=0.99:SQ=-1;ID=S2008_raw:GT=hom:DP=68:FQ=1:SQ=-1;ID=S2509_raw:GT=hom:DP=132:FQ=1:SQ=-1;ID=S2508_raw:GT=hom:DP=157:FQ=1:SQ=-1;ID=S2666_raw:GT=hom:DP=165:FQ=1:SQ=-1;ID=S2669_raw:GT=hom:DP=123:FQ=1:SQ=-1;ID=S2166_raw:GT=hom:DP=182:FQ=1:SQ=-1;ID=S1775_raw:GT=hom:DP=104:FQ=1:SQ=-1;ID=S2501_raw:GT=hom:DP=125:FQ=1:SQ=-1;ID=S2500_raw:GT=hom:DP=121:FQ=1:SQ=-1;ID=S2502_raw:GT=hom:DP=129:FQ=1:SQ=-1;ID=S2505_raw:GT=hom:DP=109:FQ=1:SQ=-1;ID=S2176_raw:GT=hom:DP=204:FQ=1:SQ=-1;ID=S2506_raw:GT=hom:DP=88:FQ=1:SQ=-1;ID=S2667_raw:GT=hom:DP=128:FQ=1:SQ=-1;
10	16967401	16967401	C	T	exonic	CUBN	nonsynonymous SNV	CUBN:NM_001081:exon43:c.6485G>A:p.C2162Y	rs1276712	Benign	ID=S2009_raw:GT=hom:DP=67:FQ=1:SQ=-1;ID=S2503_raw:GT=hom:DP=92:FQ=1:SQ=-1;ID=S2507_raw:GT=hom:DP=79:FQ=1:SQ=-1;ID=S2668_raw:GT=hom:DP=100:FQ=1:SQ=-1;ID=S2012_raw:GT=hom:DP=77:FQ=1:SQ=-1;ID=S2010_raw:GT=hom:DP=96:FQ=1:SQ=-1;ID=S2662_raw:GT=hom:DP=6:FQ=1:SQ=-1;ID=S2663_raw:GT=hom:DP=70:FQ=1:SQ=-1;ID=S2011_raw:GT=hom:DP=124:FQ=1:SQ=-1;ID=S2504_raw:GT=hom:DP=99:FQ=1:SQ=-1;ID=S2008_raw:GT=hom:DP=91:FQ=1:SQ=-1;ID=S2509_raw:GT=hom:DP=76:FQ=1:SQ=-1;ID=S2508_raw:GT=hom:DP=99:FQ=1:SQ=-1;ID=S2666_raw:GT=hom:DP=126:FQ=1:SQ=-1;ID=S2669_raw:GT=hom:DP=86:FQ=1:SQ=-1;ID=S2166_raw:GT=hom:DP=124:FQ=1:SQ=-1;ID=S1775_raw:GT=hom:DP=114:FQ=1:SQ=-1;ID=S2501_raw:GT=hom:DP=86:FQ=1:SQ=-1;ID=S2500_raw:GT=hom:DP=95:FQ=1:SQ=-1;ID=S2502_raw:GT=hom:DP=88:FQ=1:SQ=-1;ID=S2505_raw:GT=hom:DP=105:FQ=1:SQ=-1;ID=S2176_raw:GT=hom:DP=149:FQ=1:SQ=-1;ID=S2506_raw:GT=hom:DP=68:FQ=1:SQ=-1;ID=S2667_raw:GT=hom:DP=59:FQ=1:SQ=-1;
10	17168615	17168615	T	C	intronic	CUBN	.	.	rs2295812	.	ID=S2009_raw:GT=hom:DP=15:FQ=1:SQ=-1;ID=S2503_raw:GT=hom:DP=10:FQ=1:SQ=-1;ID=S2507_raw:GT=hom:DP=11:FQ=1:SQ=-1;ID=S2668_raw:GT=hom:DP=5:FQ=1:SQ=-1;ID=S2012_raw:GT=hom:DP=22:FQ=1:SQ=-1;ID=S2010_raw:GT=hom:DP=24:FQ=1:SQ=-1;ID=S2662_raw:GT=hom:DP=1:FQ=1:SQ=-1;ID=S2663_raw:GT=hom:DP=10:FQ=1:SQ=-1;ID=S2011_raw:GT=hom:DP=42:FQ=1:SQ=-1;ID=S2504_raw:GT=hom:DP=5:FQ=1:SQ=-1;ID=S2008_raw:GT=hom:DP=39:FQ=1:SQ=-1;ID=S2509_raw:GT=hom:DP=3:FQ=1:SQ=-1;ID=S2508_raw:GT=hom:DP=9:FQ=1:SQ=-1;ID=S2666_raw:GT=hom:DP=4:FQ=1:SQ=-1;ID=S2669_raw:GT=hom:DP=7:FQ=1:SQ=-1;ID=S2166_raw:GT=hom:DP=21:FQ=1:SQ=-1;ID=S1775_raw:GT=hom:DP=8:FQ=1:SQ=-1;ID=S2501_raw:GT=hom:DP=9:FQ=1:SQ=-1;ID=S2500_raw:GT=hom:DP=9:FQ=1:SQ=-1;ID=S2502_raw:GT=hom:DP=11:FQ=1:SQ=-1;ID=S2505_raw:GT=hom:DP=14:FQ=1:SQ=-1;ID=S2176_raw:GT=hom:DP=16:FQ=1:SQ=-1;ID=S2506_raw:GT=hom:DP=10:FQ=1:SQ=-1;ID=S2667_raw:GT=hom:DP=13:FQ=1:SQ=-1;
10	17171265	17171265	G	C	intronic	CUBN	.	.	rs2281648	.	ID=S2009_raw:GT=hom:DP=81:FQ=1:SQ=-1;ID=S2503_raw:GT=hom:DP=81:FQ=1:SQ=-1;ID=S2507_raw:GT=hom:DP=71:FQ=1:SQ=-1;ID=S2668_raw:GT=hom:DP=79:FQ=1:SQ=-1;ID=S2012_raw:GT=hom:DP=137:FQ=1:SQ=-1;ID=S2010_raw:GT=hom:DP=149:FQ=1:SQ=-1;ID=S2662_raw:GT=hom:DP=13:FQ=1:SQ=-1;ID=S2663_raw:GT=hom:DP=62:FQ=1:SQ=-1;ID=S2011_raw:GT=hom:DP=194:FQ=1:SQ=-1;ID=S2504_raw:GT=hom:DP=50:FQ=1:SQ=-1;ID=S2008_raw:GT=hom:DP=121:FQ=1:SQ=-1;ID=S2509_raw:GT=hom:DP=51:FQ=1:SQ=-1;ID=S2508_raw:GT=hom:DP=97:FQ=1:SQ=-1;ID=S2666_raw:GT=hom:DP=87:FQ=1:SQ=-1;ID=S2669_raw:GT=hom:DP=38:FQ=0.97:SQ=-1;ID=S2166_raw:GT=hom:DP=177:FQ=1:SQ=-1;ID=S1775_raw:GT=hom:DP=132:FQ=1:SQ=-1;ID=S2501_raw:GT=hom:DP=68:FQ=1:SQ=-1;ID=S2500_raw:GT=hom:DP=78:FQ=1:SQ=-1;ID=S2502_raw:GT=hom:DP=51:FQ=1:SQ=-1;ID=S2505_raw:GT=hom:DP=69:FQ=1:SQ=-1;ID=S2176_raw:GT=hom:DP=169:FQ=1:SQ=-1;ID=S2506_raw:GT=hom:DP=63:FQ=1:SQ=-1;ID=S2667_raw:GT=hom:DP=72:FQ=1:SQ=-1;
18	49867224	49867224	T	C	exonic	DCC	nonsynonymous SNV	DCC:NM_005215:exon1:c.67T>C:p.F23L	rs9951523	.	ID=S2009_raw:GT=hom:DP=270:FQ=1:SQ=-1;ID=S2503_raw:GT=hom:DP=130:FQ=1:SQ=-1;ID=S2507_raw:GT=hom:DP=138:FQ=1:SQ=-1;ID=S2668_raw:GT=hom:DP=191:FQ=1:SQ=-1;ID=S2012_raw:GT=hom:DP=314:FQ=1:SQ=-1;ID=S2010_raw:GT=hom:DP=372:FQ=1:SQ=-1;ID=S2662_raw:GT=hom:DP=194:FQ=1:SQ=-1;ID=S2663_raw:GT=hom:DP=112:FQ=1:SQ=-1;ID=S2011_raw:GT=hom:DP=422:FQ=1:SQ=-1;ID=S2504_raw:GT=hom:DP=182:FQ=1:SQ=-1;ID=S2008_raw:GT=hom:DP=298:FQ=1:SQ=-1;ID=S2509_raw:GT=hom:DP=155:FQ=1:SQ=-1;ID=S2508_raw:GT=hom:DP=170:FQ=1:SQ=-1;ID=S2666_raw:GT=hom:DP=130:FQ=1:SQ=-1;ID=S2669_raw:GT=hom:DP=123:FQ=1:SQ=-1;ID=S2166_raw:GT=hom:DP=131:FQ=1:SQ=-1;ID=S1775_raw:GT=hom:DP=91:FQ=1:SQ=-1;ID=S2501_raw:GT=hom:DP=160:FQ=1:SQ=-1;ID=S2500_raw:GT=hom:DP=157:FQ=0.99:SQ=-1;ID=S2502_raw:GT=hom:DP=163:FQ=1:SQ=-1;ID=S2505_raw:GT=hom:DP=148:FQ=1:SQ=-1;ID=S2176_raw:GT=hom:DP=175:FQ=1:SQ=-1;ID=S2506_raw:GT=hom:DP=154:FQ=1:SQ=-1;ID=S2667_raw:GT=hom:DP=130:FQ=1:SQ=-1;
6	24302087	24302087	G	A	intronic	DCDC2	.	.	rs807703	.	ID=S2009_raw:GT=hom:DP=143:FQ=1:SQ=-1;ID=S2503_raw:GT=hom:DP=106:FQ=1:SQ=-1;ID=S2507_raw:GT=hom:DP=85:FQ=1:SQ=-1;ID=S2668_raw:GT=hom:DP=144:FQ=1:SQ=-1;ID=S2012_raw:GT=hom:DP=172:FQ=1:SQ=-1;ID=S2010_raw:GT=hom:DP=209:FQ=1:SQ=-1;ID=S2662_raw:GT=hom:DP=53:FQ=1:SQ=-1;ID=S2663_raw:GT=hom:DP=111:FQ=1:SQ=-1;ID=S2011_raw:GT=hom:DP=219:FQ=1:SQ=-1;ID=S2504_raw:GT=hom:DP=85:FQ=1:SQ=-1;ID=S2008_raw:GT=hom:DP=167:FQ=1:SQ=-1;ID=S2509_raw:GT=hom:DP=98:FQ=1:SQ=-1;ID=S2508_raw:GT=hom:DP=109:FQ=1:SQ=-1;ID=S2666_raw:GT=hom:DP=83:FQ=1:SQ=-1;ID=S2669_raw:GT=hom:DP=78:FQ=1:SQ=-1;ID=S2166_raw:GT=hom:DP=194:FQ=1:SQ=-1;ID=S1775_raw:GT=hom:DP=160:FQ=1:SQ=-1;ID=S2501_raw:GT=hom:DP=120:FQ=1:SQ=-1;ID=S2500_raw:GT=hom:DP=116:FQ=1:SQ=-1;ID=S2502_raw:GT=hom:DP=94:FQ=1:SQ=-1;ID=S2505_raw:GT=hom:DP=106:FQ=1:SQ=-1;ID=S2176_raw:GT=hom:DP=233:FQ=1:SQ=-1;ID=S2506_raw:GT=hom:DP=82:FQ=1:SQ=-1;ID=S2667_raw:GT=hom:DP=87:FQ=1:SQ=-1;
11	687699	687699	G	A	intronic	DEAF1	.	.	rs7481651	.	ID=S2009_raw:GT=hom:DP=8:FQ=1:SQ=-1;ID=S2503_raw:GT=hom:DP=10:FQ=1:SQ=-1;ID=S2507_raw:GT=hom:DP=12:FQ=1:SQ=-1;ID=S2668_raw:GT=hom:DP=5:FQ=1:SQ=-1;ID=S2012_raw:GT=hom:DP=9:FQ=1:SQ=-1;ID=S2010_raw:GT=hom:DP=6:FQ=1:SQ=-1;ID=S2662_raw:GT=hom:DP=9:FQ=1:SQ=-1;ID=S2663_raw:GT=hom:DP=7:FQ=1:SQ=-1;ID=S2011_raw:GT=hom:DP=14:FQ=1:SQ=-1;ID=S2504_raw:GT=hom:DP=13:FQ=1:SQ=-1;ID=S2008_raw:GT=hom:DP=10:FQ=1:SQ=-1;ID=S2509_raw:GT=hom:DP=6:FQ=1:SQ=-1;ID=S2508_raw:GT=hom:DP=9:FQ=1:SQ=-1;ID=S2666_raw:GT=hom:DP=1:FQ=1:SQ=-1;ID=S2669_raw:GT=hom:DP=5:FQ=1:SQ=-1;ID=S2166_raw:GT=hom:DP=10:FQ=1:SQ=-1;ID=S1775_raw:GT=hom:DP=7:FQ=1:SQ=-1;ID=S2501_raw:GT=hom:DP=3:FQ=1:SQ=-1;ID=S2500_raw:GT=hom:DP=5:FQ=1:SQ=-1;ID=S2502_raw:GT=hom:DP=7:FQ=1:SQ=-1;ID=S2505_raw:GT=hom:DP=9:FQ=1:SQ=-1;ID=S2176_raw:GT=hom:DP=8:FQ=1:SQ=-1;ID=S2506_raw:GT=hom:DP=13:FQ=1:SQ=-1;ID=S2667_raw:GT=hom:DP=7:FQ=1:SQ=-1;
22	18900669	18900669	A	G	UTR3	DGCR6;LOC102724788;PRODH	.	NM_005675:c.*1467A>G;NM_001368250:c.*19T>C;NM_001368249:c.*19T>C;NM_001195226:c.*19T>C;NM_016335:c.*19T>C	rs383964	.	ID=S2009_raw:GT=hom:DP=31:FQ=1:SQ=-1;ID=S2503_raw:GT=hom:DP=17:FQ=1:SQ=-1;ID=S2507_raw:GT=hom:DP=17:FQ=1:SQ=-1;ID=S2668_raw:GT=hom:DP=31:FQ=1:SQ=-1;ID=S2012_raw:GT=hom:DP=23:FQ=1:SQ=-1;ID=S2010_raw:GT=hom:DP=36:FQ=1:SQ=-1;ID=S2662_raw:GT=hom:DP=31:FQ=1:SQ=-1;ID=S2663_raw:GT=hom:DP=18:FQ=1:SQ=-1;ID=S2011_raw:GT=hom:DP=43:FQ=1:SQ=-1;ID=S2504_raw:GT=hom:DP=30:FQ=1:SQ=-1;ID=S2008_raw:GT=hom:DP=28:FQ=1:SQ=-1;ID=S2509_raw:GT=hom:DP=24:FQ=1:SQ=-1;ID=S2508_raw:GT=hom:DP=27:FQ=1:SQ=-1;ID=S2666_raw:GT=hom:DP=12:FQ=1:SQ=-1;ID=S2669_raw:GT=hom:DP=24:FQ=1:SQ=-1;ID=S2166_raw:GT=hom:DP=16:FQ=1:SQ=-1;ID=S1775_raw:GT=hom:DP=15:FQ=1:SQ=-1;ID=S2501_raw:GT=hom:DP=24:FQ=1:SQ=-1;ID=S2500_raw:GT=hom:DP=21:FQ=1:SQ=-1;ID=S2502_raw:GT=hom:DP=29:FQ=1:SQ=-1;ID=S2505_raw:GT=hom:DP=13:FQ=1:SQ=-1;ID=S2176_raw:GT=hom:DP=20:FQ=1:SQ=-1;ID=S2506_raw:GT=hom:DP=16:FQ=1:SQ=-1;ID=S2667_raw:GT=hom:DP=32:FQ=1:SQ=-1;
16	72050885	72050885	G	C	intronic	DHODH	.	.	rs11075914	.	ID=S2009_raw:GT=hom:DP=13:FQ=1:SQ=-1;ID=S2503_raw:GT=hom:DP=48:FQ=1:SQ=-1;ID=S2507_raw:GT=hom:DP=55:FQ=1:SQ=-1;ID=S2668_raw:GT=hom:DP=75:FQ=1:SQ=-1;ID=S2012_raw:GT=hom:DP=17:FQ=1:SQ=-1;ID=S2010_raw:GT=hom:DP=12:FQ=1:SQ=-1;ID=S2662_raw:GT=hom:DP=59:FQ=1:SQ=-1;ID=S2663_raw:GT=hom:DP=30:FQ=1:SQ=-1;ID=S2011_raw:GT=hom:DP=30:FQ=0.97:SQ=-1;ID=S2504_raw:GT=hom:DP=58:FQ=1:SQ=-1;ID=S2008_raw:GT=hom:DP=16:FQ=1:SQ=-1;ID=S2509_raw:GT=hom:DP=32:FQ=1:SQ=-1;ID=S2508_raw:GT=hom:DP=56:FQ=1:SQ=-1;ID=S2666_raw:GT=hom:DP=45:FQ=1:SQ=-1;ID=S2669_raw:GT=hom:DP=47:FQ=1:SQ=-1;ID=S2166_raw:GT=hom:DP=38:FQ=1:SQ=-1;ID=S1775_raw:GT=hom:DP=39:FQ=1:SQ=-1;ID=S2501_raw:GT=hom:DP=52:FQ=1:SQ=-1;ID=S2500_raw:GT=hom:DP=45:FQ=1:SQ=-1;ID=S2502_raw:GT=hom:DP=48:FQ=1:SQ=-1;ID=S2505_raw:GT=hom:DP=40:FQ=1:SQ=-1;ID=S2176_raw:GT=hom:DP=60:FQ=1:SQ=-1;ID=S2506_raw:GT=hom:DP=42:FQ=1:SQ=-1;ID=S2667_raw:GT=hom:DP=45:FQ=1:SQ=-1;
1	2,23E+08	2,23E+08	C	T	intronic	DISP1	.	.	rs2926159	.	ID=S2009_raw:GT=hom:DP=39:FQ=1:SQ=-1;ID=S2503_raw:GT=hom:DP=42:FQ=1:SQ=-1;ID=S2507_raw:GT=hom:DP=19:FQ=1:SQ=-1;ID=S2668_raw:GT=hom:DP=22:FQ=1:SQ=-1;ID=S2012_raw:GT=hom:DP=40:FQ=1:SQ=-1;ID=S2010_raw:GT=hom:DP=40:FQ=1:SQ=-1;ID=S2662_raw:GT=hom:DP=2:FQ=1:SQ=-1;ID=S2663_raw:GT=hom:DP=27:FQ=1:SQ=-1;ID=S2011_raw:GT=hom:DP=70:FQ=1:SQ=-1;ID=S2504_raw:GT=hom:DP=20:FQ=1:SQ=-1;ID=S2008_raw:GT=hom:DP=37:FQ=1:SQ=-1;ID=S2509_raw:GT=hom:DP=17:FQ=1:SQ=-1;ID=S2508_raw:GT=hom:DP=22:FQ=1:SQ=-1;ID=S2666_raw:GT=hom:DP=17:FQ=1:SQ=-1;ID=S2669_raw:GT=hom:DP=15:FQ=1:SQ=-1;ID=S2166_raw:GT=hom:DP=22:FQ=1:SQ=-1;ID=S1775_raw:GT=hom:DP=43:FQ=1:SQ=-1;ID=S2501_raw:GT=hom:DP=30:FQ=1:SQ=-1;ID=S2500_raw:GT=hom:DP=16:FQ=1:SQ=-1;ID=S2502_raw:GT=hom:DP=28:FQ=1:SQ=-1;ID=S2505_raw:GT=hom:DP=38:FQ=1:SQ=-1;ID=S2176_raw:GT=hom:DP=25:FQ=1:SQ=-1;ID=S2506_raw:GT=hom:DP=16:FQ=1:SQ=-1;ID=S2667_raw:GT=hom:DP=22:FQ=1:SQ=-1;
X	1,54E+08	1,54E+08	G	T	exonic	DKC1	synonymous SNV	DKC1:NM_001142463:exon5:c.369G>T:p.T123T,DKC1:NM_001288747:exon5:c.369G>T:p.T123T,DKC1:NM_001363:exon5:c.369G>T:p.T123T	rs2728532	Benign	ID=S2009_raw:GT=hom:DP=16:FQ=1:SQ=-1;ID=S2503_raw:GT=hom:DP=62:FQ=1:SQ=-1;ID=S2507_raw:GT=hom:DP=70:FQ=1:SQ=-1;ID=S2668_raw:GT=hom:DP=181:FQ=1:SQ=-1;ID=S2012_raw:GT=hom:DP=13:FQ=1:SQ=-1;ID=S2010_raw:GT=hom:DP=44:FQ=1:SQ=-1;ID=S2662_raw:GT=hom:DP=110:FQ=1:SQ=-1;ID=S2663_raw:GT=hom:DP=123:FQ=1:SQ=-1;ID=S2011_raw:GT=hom:DP=16:FQ=1:SQ=-1;ID=S2504_raw:GT=hom:DP=116:FQ=1:SQ=-1;ID=S2008_raw:GT=hom:DP=26:FQ=1:SQ=-1;ID=S2509_raw:GT=hom:DP=51:FQ=1:SQ=-1;ID=S2508_raw:GT=hom:DP=58:FQ=1:SQ=-1;ID=S2666_raw:GT=hom:DP=81:FQ=1:SQ=-1;ID=S2669_raw:GT=hom:DP=70:FQ=1:SQ=-1;ID=S2166_raw:GT=hom:DP=41:FQ=1:SQ=-1;ID=S1775_raw:GT=hom:DP=24:FQ=1:SQ=-1;ID=S2501_raw:GT=hom:DP=59:FQ=1:SQ=-1;ID=S2500_raw:GT=hom:DP=83:FQ=1:SQ=-1;ID=S2502_raw:GT=hom:DP=142:FQ=1:SQ=-1;ID=S2505_raw:GT=hom:DP=60:FQ=1:SQ=-1;ID=S2176_raw:GT=hom:DP=45:FQ=1:SQ=-1;ID=S2506_raw:GT=hom:DP=50:FQ=1:SQ=-1;ID=S2667_raw:GT=hom:DP=137:FQ=1:SQ=-1;
8	13258936	13258936	T	A	intronic	DLC1	.	.	rs289583	.	ID=S2009_raw:GT=hom:DP=12:FQ=1:SQ=-1;ID=S2503_raw:GT=hom:DP=19:FQ=1:SQ=-1;ID=S2507_raw:GT=hom:DP=12:FQ=1:SQ=-1;ID=S2668_raw:GT=hom:DP=18:FQ=1:SQ=-1;ID=S2012_raw:GT=hom:DP=15:FQ=1:SQ=-1;ID=S2010_raw:GT=hom:DP=10:FQ=1:SQ=-1;ID=S2662_raw:GT=hom:DP=8:FQ=1:SQ=-1;ID=S2663_raw:GT=hom:DP=10:FQ=1:SQ=-1;ID=S2011_raw:GT=hom:DP=20:FQ=1:SQ=-1;ID=S2504_raw:GT=hom:DP=17:FQ=1:SQ=-1;ID=S2008_raw:GT=hom:DP=16:FQ=1:SQ=-1;ID=S2509_raw:GT=hom:DP=9:FQ=1:SQ=-1;ID=S2508_raw:GT=hom:DP=23:FQ=1:SQ=-1;ID=S2666_raw:GT=hom:DP=40:FQ=1:SQ=-1;ID=S2669_raw:GT=hom:DP=13:FQ=1:SQ=-1;ID=S2166_raw:GT=hom:DP=8:FQ=1:SQ=-1;ID=S1775_raw:GT=hom:DP=4:FQ=1:SQ=-1;ID=S2501_raw:GT=hom:DP=16:FQ=1:SQ=-1;ID=S2500_raw:GT=hom:DP=21:FQ=1:SQ=-1;ID=S2502_raw:GT=hom:DP=17:FQ=1:SQ=-1;ID=S2505_raw:GT=hom:DP=14:FQ=1:SQ=-1;ID=S2176_raw:GT=hom:DP=6:FQ=1:SQ=-1;ID=S2506_raw:GT=hom:DP=13:FQ=1:SQ=-1;ID=S2667_raw:GT=hom:DP=6:FQ=1:SQ=-1;
14	1,01E+08	1,01E+08	A	G	exonic	DLK1	nonsynonymous SNV	DLK1:NM_001317172:exon4:c.301A>G:p.R101G,DLK1:NM_003836:exon4:c.301A>G:p.R101G	rs6575799	.	ID=S2009_raw:GT=hom:DP=85:FQ=1:SQ=-1;ID=S2503_raw:GT=hom:DP=83:FQ=1:SQ=-1;ID=S2507_raw:GT=hom:DP=89:FQ=1:SQ=-1;ID=S2668_raw:GT=hom:DP=103:FQ=1:SQ=-1;ID=S2012_raw:GT=hom:DP=70:FQ=1:SQ=-1;ID=S2010_raw:GT=hom:DP=85:FQ=1:SQ=-1;ID=S2662_raw:GT=hom:DP=182:FQ=1:SQ=-1;ID=S2663_raw:GT=hom:DP=74:FQ=1:SQ=-1;ID=S2011_raw:GT=hom:DP=121:FQ=1:SQ=-1;ID=S2504_raw:GT=hom:DP=97:FQ=1:SQ=-1;ID=S2008_raw:GT=hom:DP=103:FQ=1:SQ=-1;ID=S2509_raw:GT=hom:DP=117:FQ=1:SQ=-1;ID=S2508_raw:GT=hom:DP=93:FQ=1:SQ=-1;ID=S2666_raw:GT=hom:DP=90:FQ=1:SQ=-1;ID=S2669_raw:GT=hom:DP=109:FQ=1:SQ=-1;ID=S2166_raw:GT=hom:DP=121:FQ=1:SQ=-1;ID=S1775_raw:GT=hom:DP=77:FQ=1:SQ=-1;ID=S2501_raw:GT=hom:DP=92:FQ=1:SQ=-1;ID=S2500_raw:GT=hom:DP=115:FQ=1:SQ=-1;ID=S2502_raw:GT=hom:DP=118:FQ=1:SQ=-1;ID=S2505_raw:GT=hom:DP=91:FQ=1:SQ=-1;ID=S2176_raw:GT=hom:DP=145:FQ=1:SQ=-1;ID=S2506_raw:GT=hom:DP=100:FQ=1:SQ=-1;ID=S2667_raw:GT=hom:DP=81:FQ=1:SQ=-1;
21	45683447	45683447	A	G	intergenic	DNMT3L;AIRE	.	dist=1348;dist=22312	rs2850160	.	ID=S2009_raw:GT=hom:DP=6:FQ=1:SQ=-1;ID=S2503_raw:GT=hom:DP=19:FQ=1:SQ=-1;ID=S2507_raw:GT=hom:DP=15:FQ=1:SQ=-1;ID=S2668_raw:GT=hom:DP=32:FQ=1:SQ=-1;ID=S2012_raw:GT=hom:DP=4:FQ=1:SQ=-1;ID=S2010_raw:GT=hom:DP=3:FQ=1:SQ=-1;ID=S2662_raw:GT=hom:DP=43:FQ=1:SQ=-1;ID=S2663_raw:GT=hom:DP=16:FQ=1:SQ=-1;ID=S2011_raw:GT=hom:DP=2:FQ=1:SQ=-1;ID=S2504_raw:GT=hom:DP=19:FQ=1:SQ=-1;ID=S2008_raw:GT=hom:DP=3:FQ=1:SQ=-1;ID=S2509_raw:GT=hom:DP=26:FQ=1:SQ=-1;ID=S2508_raw:GT=hom:DP=18:FQ=1:SQ=-1;ID=S2666_raw:GT=hom:DP=9:FQ=1:SQ=-1;ID=S2669_raw:GT=hom:DP=19:FQ=1:SQ=-1;ID=S2166_raw:GT=hom:DP=14:FQ=1:SQ=-1;ID=S1775_raw:GT=hom:DP=10:FQ=1:SQ=-1;ID=S2501_raw:GT=hom:DP=20:FQ=1:SQ=-1;ID=S2500_raw:GT=hom:DP=24:FQ=1:SQ=-1;ID=S2502_raw:GT=hom:DP=12:FQ=1:SQ=-1;ID=S2505_raw:GT=hom:DP=15:FQ=1:SQ=-1;ID=S2176_raw:GT=hom:DP=20:FQ=1:SQ=-1;ID=S2506_raw:GT=hom:DP=17:FQ=1:SQ=-1;ID=S2667_raw:GT=hom:DP=14:FQ=1:SQ=-1;
11	65118971	65118971	T	C	intronic	DPF2	.	.	rs620888	.	ID=S2009_raw:GT=hom:DP=6:FQ=1:SQ=-1;ID=S2503_raw:GT=hom:DP=56:FQ=1:SQ=-1;ID=S2507_raw:GT=hom:DP=41:FQ=1:SQ=-1;ID=S2668_raw:GT=hom:DP=72:FQ=0.99:SQ=-1;ID=S2012_raw:GT=hom:DP=1:FQ=1:SQ=-1;ID=S2010_raw:GT=hom:DP=3:FQ=1:SQ=-1;ID=S2662_raw:GT=hom:DP=5:FQ=1:SQ=-1;ID=S2663_raw:GT=hom:DP=33:FQ=1:SQ=-1;ID=S2011_raw:GT=hom:DP=2:FQ=1:SQ=-1;ID=S2504_raw:GT=hom:DP=57:FQ=1:SQ=-1;ID=S2008_raw:GT=hom:DP=1:FQ=1:SQ=-1;ID=S2509_raw:GT=hom:DP=39:FQ=1:SQ=-1;ID=S2508_raw:GT=hom:DP=55:FQ=1:SQ=-1;ID=S2666_raw:GT=hom:DP=53:FQ=1:SQ=-1;ID=S2669_raw:GT=hom:DP=25:FQ=1:SQ=-1;ID=S2166_raw:GT=hom:DP=14:FQ=1:SQ=-1;ID=S1775_raw:GT=hom:DP=12:FQ=1:SQ=-1;ID=S2501_raw:GT=hom:DP=60:FQ=1:SQ=-1;ID=S2500_raw:GT=hom:DP=59:FQ=1:SQ=-1;ID=S2502_raw:GT=hom:DP=64:FQ=1:SQ=-1;ID=S2505_raw:GT=hom:DP=38:FQ=1:SQ=-1;ID=S2176_raw:GT=hom:DP=24:FQ=1:SQ=-1;ID=S2506_raw:GT=hom:DP=54:FQ=1:SQ=-1;ID=S2667_raw:GT=hom:DP=69:FQ=1:SQ=-1;
6	1,17E+08	1,17E+08	T	C	exonic	DSE	synonymous SNV	DSE:NM_001080976:exon6:c.1926T>C:p.N642N,DSE:NM_001322939:exon6:c.1983T>C:p.N661N,DSE:NM_001322941:exon6:c.1365T>C:p.N455N,DSE:NM_013352:exon6:c.1926T>C:p.N642N,DSE:NM_001322937:exon7:c.1926T>C:p.N642N,DSE:NM_001322938:exon7:c.1926T>C:p.N642N,DSE:NM_001322940:exon7:c.1365T>C:p.N455N	rs560644	.	ID=S2009_raw:GT=hom:DP=82:FQ=1:SQ=-1;ID=S2503_raw:GT=hom:DP=154:FQ=1:SQ=-1;ID=S2507_raw:GT=hom:DP=122:FQ=1:SQ=-1;ID=S2668_raw:GT=hom:DP=189:FQ=1:SQ=-1;ID=S2012_raw:GT=hom:DP=102:FQ=1:SQ=-1;ID=S2010_raw:GT=hom:DP=119:FQ=1:SQ=-1;ID=S2662_raw:GT=hom:DP=61:FQ=1:SQ=-1;ID=S2663_raw:GT=hom:DP=166:FQ=1:SQ=-1;ID=S2011_raw:GT=hom:DP=182:FQ=1:SQ=-1;ID=S2504_raw:GT=hom:DP=167:FQ=1:SQ=-1;ID=S2008_raw:GT=hom:DP=101:FQ=1:SQ=-1;ID=S2509_raw:GT=hom:DP=117:FQ=1:SQ=-1;ID=S2508_raw:GT=hom:DP=178:FQ=1:SQ=-1;ID=S2666_raw:GT=hom:DP=200:FQ=1:SQ=-1;ID=S2669_raw:GT=hom:DP=144:FQ=1:SQ=-1;ID=S2166_raw:GT=hom:DP=169:FQ=1:SQ=-1;ID=S1775_raw:GT=hom:DP=115:FQ=1:SQ=-1;ID=S2501_raw:GT=hom:DP=133:FQ=1:SQ=-1;ID=S2500_raw:GT=hom:DP=134:FQ=1:SQ=-1;ID=S2502_raw:GT=hom:DP=168:FQ=1:SQ=-1;ID=S2505_raw:GT=hom:DP=163:FQ=1:SQ=-1;ID=S2176_raw:GT=hom:DP=200:FQ=1:SQ=-1;ID=S2506_raw:GT=hom:DP=123:FQ=1:SQ=-1;ID=S2667_raw:GT=hom:DP=145:FQ=1:SQ=-1;
12	89743223	89743223	G	A	exonic	DUSP6	synonymous SNV	DUSP6:NM_022652:exon2:c.516C>T:p.D172D,DUSP6:NM_001946:exon3:c.954C>T:p.D318D	rs704075	.	ID=S2009_raw:GT=hom:DP=56:FQ=1:SQ=-1;ID=S2503_raw:GT=hom:DP=79:FQ=1:SQ=-1;ID=S2507_raw:GT=hom:DP=95:FQ=1:SQ=-1;ID=S2668_raw:GT=hom:DP=97:FQ=1:SQ=-1;ID=S2012_raw:GT=hom:DP=80:FQ=1:SQ=-1;ID=S2010_raw:GT=hom:DP=112:FQ=1:SQ=-1;ID=S2662_raw:GT=hom:DP=75:FQ=1:SQ=-1;ID=S2663_raw:GT=hom:DP=83:FQ=1:SQ=-1;ID=S2011_raw:GT=hom:DP=137:FQ=1:SQ=-1;ID=S2504_raw:GT=hom:DP=86:FQ=1:SQ=-1;ID=S2008_raw:GT=hom:DP=106:FQ=1:SQ=-1;ID=S2509_raw:GT=hom:DP=90:FQ=1:SQ=-1;ID=S2508_raw:GT=hom:DP=92:FQ=1:SQ=-1;ID=S2666_raw:GT=hom:DP=90:FQ=1:SQ=-1;ID=S2669_raw:GT=hom:DP=86:FQ=1:SQ=-1;ID=S2166_raw:GT=hom:DP=194:FQ=1:SQ=-1;ID=S1775_raw:GT=hom:DP=96:FQ=1:SQ=-1;ID=S2501_raw:GT=hom:DP=75:FQ=1:SQ=-1;ID=S2500_raw:GT=hom:DP=88:FQ=1:SQ=-1;ID=S2502_raw:GT=hom:DP=134:FQ=1:SQ=-1;ID=S2505_raw:GT=hom:DP=104:FQ=1:SQ=-1;ID=S2176_raw:GT=hom:DP=184:FQ=1:SQ=-1;ID=S2506_raw:GT=hom:DP=40:FQ=1:SQ=-1;ID=S2667_raw:GT=hom:DP=73:FQ=1:SQ=-1;
1	1277533	1277533	T	C	exonic;splicing	DVL1	synonymous SNV	DVL1:NM_001330311:exon4:c.366A>G:p.P122P,DVL1:NM_004421:exon4:c.366A>G:p.P122P	rs307362	.	ID=S2009_raw:GT=hom:DP=140:FQ=1:SQ=-1;ID=S2503_raw:GT=hom:DP=106:FQ=1:SQ=-1;ID=S2507_raw:GT=hom:DP=88:FQ=1:SQ=-1;ID=S2668_raw:GT=hom:DP=88:FQ=1:SQ=-1;ID=S2012_raw:GT=hom:DP=105:FQ=1:SQ=-1;ID=S2010_raw:GT=hom:DP=106:FQ=1:SQ=-1;ID=S2662_raw:GT=hom:DP=171:FQ=1:SQ=-1;ID=S2663_raw:GT=hom:DP=79:FQ=1:SQ=-1;ID=S2011_raw:GT=hom:DP=186:FQ=1:SQ=-1;ID=S2504_raw:GT=hom:DP=101:FQ=1:SQ=-1;ID=S2008_raw:GT=hom:DP=176:FQ=1:SQ=-1;ID=S2509_raw:GT=hom:DP=95:FQ=1:SQ=-1;ID=S2508_raw:GT=hom:DP=124:FQ=1:SQ=-1;ID=S2666_raw:GT=hom:DP=65:FQ=1:SQ=-1;ID=S2669_raw:GT=hom:DP=90:FQ=1:SQ=-1;ID=S2166_raw:GT=hom:DP=88:FQ=1:SQ=-1;ID=S1775_raw:GT=hom:DP=57:FQ=1:SQ=-1;ID=S2501_raw:GT=hom:DP=116:FQ=1:SQ=-1;ID=S2500_raw:GT=hom:DP=87:FQ=1:SQ=-1;ID=S2502_raw:GT=hom:DP=110:FQ=1:SQ=-1;ID=S2505_raw:GT=hom:DP=120:FQ=1:SQ=-1;ID=S2176_raw:GT=hom:DP=54:FQ=1:SQ=-1;ID=S2506_raw:GT=hom:DP=116:FQ=1:SQ=-1;ID=S2667_raw:GT=hom:DP=122:FQ=1:SQ=-1;
3	1,38E+08	1,38E+08	T	C	intronic	DZIP1L	.	.	rs446847	.	ID=S2009_raw:GT=hom:DP=1:FQ=1:SQ=-1;ID=S2503_raw:GT=hom:DP=25:FQ=1:SQ=-1;ID=S2507_raw:GT=hom:DP=14:FQ=1:SQ=-1;ID=S2668_raw:GT=hom:DP=15:FQ=1:SQ=-1;ID=S2012_raw:GT=hom:DP=8:FQ=1:SQ=-1;ID=S2010_raw:GT=hom:DP=4:FQ=1:SQ=-1;ID=S2662_raw:GT=hom:DP=18:FQ=1:SQ=-1;ID=S2663_raw:GT=hom:DP=19:FQ=1:SQ=-1;ID=S2011_raw:GT=hom:DP=4:FQ=1:SQ=-1;ID=S2504_raw:GT=hom:DP=26:FQ=1:SQ=-1;ID=S2008_raw:GT=hom:DP=2:FQ=1:SQ=-1;ID=S2509_raw:GT=hom:DP=23:FQ=1:SQ=-1;ID=S2508_raw:GT=hom:DP=18:FQ=1:SQ=-1;ID=S2666_raw:GT=hom:DP=18:FQ=1:SQ=-1;ID=S2669_raw:GT=hom:DP=27:FQ=1:SQ=-1;ID=S2166_raw:GT=hom:DP=25:FQ=1:SQ=-1;ID=S1775_raw:GT=hom:DP=13:FQ=1:SQ=-1;ID=S2501_raw:GT=hom:DP=22:FQ=1:SQ=-1;ID=S2500_raw:GT=hom:DP=20:FQ=1:SQ=-1;ID=S2502_raw:GT=hom:DP=22:FQ=1:SQ=-1;ID=S2505_raw:GT=hom:DP=22:FQ=1:SQ=-1;ID=S2176_raw:GT=hom:DP=30:FQ=1:SQ=-1;ID=S2506_raw:GT=hom:DP=17:FQ=1:SQ=-1;ID=S2667_raw:GT=hom:DP=19:FQ=1:SQ=-1;
13	78477674	78477674	A	G	exonic	EDNRB	synonymous SNV	EDNRB:NM_001122659:exon2:c.552T>C:p.S184S,EDNRB:NM_003991:exon2:c.552T>C:p.S184S,EDNRB:NM_000115:exon3:c.552T>C:p.S184S,EDNRB:NM_001201397:exon3:c.822T>C:p.S274S	rs5348	Benign	ID=S2009_raw:GT=hom:DP=31:FQ=1:SQ=-1;ID=S2503_raw:GT=hom:DP=119:FQ=1:SQ=-1;ID=S2507_raw:GT=hom:DP=118:FQ=1:SQ=-1;ID=S2668_raw:GT=hom:DP=142:FQ=1:SQ=-1;ID=S2012_raw:GT=hom:DP=32:FQ=1:SQ=-1;ID=S2010_raw:GT=hom:DP=37:FQ=1:SQ=-1;ID=S2662_raw:GT=hom:DP=14:FQ=1:SQ=-1;ID=S2663_raw:GT=hom:DP=120:FQ=1:SQ=-1;ID=S2011_raw:GT=hom:DP=74:FQ=1:SQ=-1;ID=S2504_raw:GT=hom:DP=128:FQ=1:SQ=-1;ID=S2008_raw:GT=hom:DP=40:FQ=1:SQ=-1;ID=S2509_raw:GT=hom:DP=137:FQ=1:SQ=-1;ID=S2508_raw:GT=hom:DP=125:FQ=1:SQ=-1;ID=S2666_raw:GT=hom:DP=139:FQ=1:SQ=-1;ID=S2669_raw:GT=hom:DP=79:FQ=1:SQ=-1;ID=S2166_raw:GT=hom:DP=158:FQ=1:SQ=-1;ID=S1775_raw:GT=hom:DP=98:FQ=1:SQ=-1;ID=S2501_raw:GT=hom:DP=154:FQ=1:SQ=-1;ID=S2500_raw:GT=hom:DP=118:FQ=1:SQ=-1;ID=S2502_raw:GT=hom:DP=124:FQ=1:SQ=-1;ID=S2505_raw:GT=hom:DP=150:FQ=1:SQ=-1;ID=S2176_raw:GT=hom:DP=146:FQ=1:SQ=-1;ID=S2506_raw:GT=hom:DP=116:FQ=1:SQ=-1;ID=S2667_raw:GT=hom:DP=126:FQ=1:SQ=-1;
8	49781875	49781875	A	G	intergenic	EFCAB1;SNAI2	.	dist=134005;dist=48282	rs6415601	.	ID=S2009_raw:GT=hom:DP=1:FQ=1:SQ=-1;ID=S2503_raw:GT=hom:DP=13:FQ=1:SQ=-1;ID=S2507_raw:GT=hom:DP=6:FQ=1:SQ=-1;ID=S2668_raw:GT=hom:DP=7:FQ=1:SQ=-1;ID=S2012_raw:GT=hom:DP=4:FQ=1:SQ=-1;ID=S2010_raw:GT=hom:DP=1:FQ=1:SQ=-1;ID=S2662_raw:GT=hom:DP=6:FQ=1:SQ=-1;ID=S2663_raw:GT=hom:DP=8:FQ=1:SQ=-1;ID=S2011_raw:GT=hom:DP=5:FQ=1:SQ=-1;ID=S2504_raw:GT=hom:DP=5:FQ=1:SQ=-1;ID=S2008_raw:GT=hom:DP=3:FQ=1:SQ=-1;ID=S2509_raw:GT=hom:DP=7:FQ=1:SQ=-1;ID=S2508_raw:GT=hom:DP=12:FQ=1:SQ=-1;ID=S2666_raw:GT=hom:DP=4:FQ=1:SQ=-1;ID=S2669_raw:GT=hom:DP=10:FQ=1:SQ=-1;ID=S2166_raw:GT=hom:DP=4:FQ=1:SQ=-1;ID=S1775_raw:GT=hom:DP=3:FQ=1:SQ=-1;ID=S2501_raw:GT=hom:DP=4:FQ=1:SQ=-1;ID=S2500_raw:GT=hom:DP=5:FQ=1:SQ=-1;ID=S2502_raw:GT=hom:DP=6:FQ=1:SQ=-1;ID=S2505_raw:GT=hom:DP=13:FQ=1:SQ=-1;ID=S2176_raw:GT=hom:DP=4:FQ=1:SQ=-1;ID=S2506_raw:GT=hom:DP=5:FQ=1:SQ=-1;ID=S2667_raw:GT=hom:DP=8:FQ=1:SQ=-1;
11	65636053	65636053	T	C	exonic	EFEMP2	nonsynonymous SNV	EFEMP2:NM_016938:exon8:c.775A>G:p.I259V	rs601314	Benign	ID=S2009_raw:GT=hom:DP=63:FQ=1:SQ=-1;ID=S2503_raw:GT=hom:DP=132:FQ=1:SQ=-1;ID=S2507_raw:GT=hom:DP=91:FQ=1:SQ=-1;ID=S2668_raw:GT=hom:DP=164:FQ=1:SQ=-1;ID=S2012_raw:GT=hom:DP=43:FQ=1:SQ=-1;ID=S2010_raw:GT=hom:DP=60:FQ=1:SQ=-1;ID=S2662_raw:GT=hom:DP=210:FQ=1:SQ=-1;ID=S2663_raw:GT=hom:DP=107:FQ=1:SQ=-1;ID=S2011_raw:GT=hom:DP=95:FQ=1:SQ=-1;ID=S2504_raw:GT=hom:DP=166:FQ=1:SQ=-1;ID=S2008_raw:GT=hom:DP=83:FQ=1:SQ=-1;ID=S2509_raw:GT=hom:DP=118:FQ=1:SQ=-1;ID=S2508_raw:GT=hom:DP=123:FQ=1:SQ=-1;ID=S2666_raw:GT=hom:DP=99:FQ=0.99:SQ=-1;ID=S2669_raw:GT=hom:DP=107:FQ=1:SQ=-1;ID=S2166_raw:GT=hom:DP=92:FQ=1:SQ=-1;ID=S1775_raw:GT=hom:DP=82:FQ=1:SQ=-1;ID=S2501_raw:GT=hom:DP=137:FQ=1:SQ=-1;ID=S2500_raw:GT=hom:DP=135:FQ=1:SQ=-1;ID=S2502_raw:GT=hom:DP=160:FQ=1:SQ=-1;ID=S2505_raw:GT=hom:DP=120:FQ=1:SQ=-1;ID=S2176_raw:GT=hom:DP=125:FQ=1:SQ=-1;ID=S2506_raw:GT=hom:DP=126:FQ=1:SQ=-1;ID=S2667_raw:GT=hom:DP=135:FQ=1:SQ=-1;
9	1,31E+08	1,31E+08	A	C	intergenic	ENG;AK1	.	dist=2416;dist=9291	rs7864893	.	ID=S2009_raw:GT=hom:DP=4:FQ=1:SQ=-1;ID=S2503_raw:GT=hom:DP=8:FQ=1:SQ=-1;ID=S2507_raw:GT=hom:DP=7:FQ=1:SQ=-1;ID=S2668_raw:GT=hom:DP=8:FQ=1:SQ=-1;ID=S2012_raw:GT=hom:DP=9:FQ=1:SQ=-1;ID=S2010_raw:GT=hom:DP=7:FQ=1:SQ=-1;ID=S2662_raw:GT=hom:DP=11:FQ=1:SQ=-1;ID=S2663_raw:GT=hom:DP=6:FQ=1:SQ=-1;ID=S2011_raw:GT=hom:DP=8:FQ=1:SQ=-1;ID=S2504_raw:GT=hom:DP=3:FQ=1:SQ=-1;ID=S2008_raw:GT=hom:DP=10:FQ=1:SQ=-1;ID=S2509_raw:GT=hom:DP=7:FQ=1:SQ=-1;ID=S2508_raw:GT=hom:DP=8:FQ=1:SQ=-1;ID=S2666_raw:GT=hom:DP=6:FQ=1:SQ=-1;ID=S2669_raw:GT=hom:DP=1:FQ=1:SQ=-1;ID=S2166_raw:GT=hom:DP=1:FQ=1:SQ=-1;ID=S1775_raw:GT=hom:DP=5:FQ=1:SQ=-1;ID=S2501_raw:GT=hom:DP=6:FQ=1:SQ=-1;ID=S2500_raw:GT=hom:DP=6:FQ=1:SQ=-1;ID=S2502_raw:GT=hom:DP=8:FQ=1:SQ=-1;ID=S2505_raw:GT=hom:DP=6:FQ=1:SQ=-1;ID=S2176_raw:GT=hom:DP=10:FQ=1:SQ=-1;ID=S2506_raw:GT=hom:DP=3:FQ=1:SQ=-1;ID=S2667_raw:GT=hom:DP=4:FQ=1:SQ=-1;
22	41537234	41537234	G	T	splicing	EP300	.	NM_001429:exon10:c.2053+8G>T;NM_001362843:exon10:c.2053+8G>T	rs6002267	Benign	ID=S2009_raw:GT=hom:DP=51:FQ=1:SQ=-1;ID=S2503_raw:GT=hom:DP=67:FQ=1:SQ=-1;ID=S2507_raw:GT=hom:DP=62:FQ=1:SQ=-1;ID=S2668_raw:GT=hom:DP=99:FQ=1:SQ=-1;ID=S2012_raw:GT=hom:DP=49:FQ=1:SQ=-1;ID=S2010_raw:GT=hom:DP=53:FQ=1:SQ=-1;ID=S2662_raw:GT=hom:DP=2:FQ=1:SQ=-1;ID=S2663_raw:GT=hom:DP=58:FQ=1:SQ=-1;ID=S2011_raw:GT=hom:DP=73:FQ=1:SQ=-1;ID=S2504_raw:GT=hom:DP=55:FQ=1:SQ=-1;ID=S2008_raw:GT=hom:DP=47:FQ=1:SQ=-1;ID=S2509_raw:GT=hom:DP=57:FQ=1:SQ=-1;ID=S2508_raw:GT=hom:DP=58:FQ=1:SQ=-1;ID=S2666_raw:GT=hom:DP=72:FQ=1:SQ=-1;ID=S2669_raw:GT=hom:DP=47:FQ=1:SQ=-1;ID=S2166_raw:GT=hom:DP=102:FQ=1:SQ=-1;ID=S1775_raw:GT=hom:DP=121:FQ=1:SQ=-1;ID=S2501_raw:GT=hom:DP=66:FQ=1:SQ=-1;ID=S2500_raw:GT=hom:DP=58:FQ=1:SQ=-1;ID=S2502_raw:GT=hom:DP=59:FQ=1:SQ=-1;ID=S2505_raw:GT=hom:DP=66:FQ=1:SQ=-1;ID=S2176_raw:GT=hom:DP=135:FQ=1:SQ=-1;ID=S2506_raw:GT=hom:DP=72:FQ=1:SQ=-1;ID=S2667_raw:GT=hom:DP=63:FQ=1:SQ=-1;
18	43547132	43547132	G	-	intronic	EPG5	.	.	.	.	ID=S2009_raw:GT=hom:DP=89:FQ=1:SQ=-1;ID=S2503_raw:GT=hom:DP=113:FQ=1:SQ=-1;ID=S2507_raw:GT=hom:DP=95:FQ=1:SQ=-1;ID=S2668_raw:GT=hom:DP=157:FQ=1:SQ=-1;ID=S2012_raw:GT=hom:DP=93:FQ=1:SQ=-1;ID=S2010_raw:GT=hom:DP=111:FQ=1:SQ=-1;ID=S2662_raw:GT=hom:DP=234:FQ=1:SQ=-1;ID=S2663_raw:GT=hom:DP=109:FQ=1:SQ=-1;ID=S2011_raw:GT=hom:DP=122:FQ=1:SQ=-1;ID=S2504_raw:GT=hom:DP=118:FQ=1:SQ=-1;ID=S2008_raw:GT=hom:DP=104:FQ=1:SQ=-1;ID=S2509_raw:GT=hom:DP=108:FQ=1:SQ=-1;ID=S2508_raw:GT=hom:DP=116:FQ=1:SQ=-1;ID=S2666_raw:GT=hom:DP=117:FQ=1:SQ=-1;ID=S2669_raw:GT=hom:DP=125:FQ=1:SQ=-1;ID=S2166_raw:GT=hom:DP=93:FQ=1:SQ=-1;ID=S1775_raw:GT=hom:DP=80:FQ=1:SQ=-1;ID=S2501_raw:GT=hom:DP=85:FQ=1:SQ=-1;ID=S2500_raw:GT=hom:DP=105:FQ=1:SQ=-1;ID=S2502_raw:GT=hom:DP=131:FQ=1:SQ=-1;ID=S2505_raw:GT=hom:DP=114:FQ=1:SQ=-1;ID=S2176_raw:GT=hom:DP=84:FQ=1:SQ=-1;ID=S2506_raw:GT=hom:DP=96:FQ=1:SQ=-1;ID=S2667_raw:GT=hom:DP=92:FQ=1:SQ=-1;
10	50680685	50680685	A	C	intronic	ERCC6	.	.	rs10437449	.	ID=S2009_raw:GT=hom:DP=1:FQ=1:SQ=-1;ID=S2503_raw:GT=hom:DP=8:FQ=1:SQ=-1;ID=S2507_raw:GT=hom:DP=4:FQ=1:SQ=-1;ID=S2668_raw:GT=hom:DP=9:FQ=1:SQ=-1;ID=S2012_raw:GT=hom:DP=2:FQ=1:SQ=-1;ID=S2010_raw:GT=hom:DP=1:FQ=1:SQ=-1;ID=S2662_raw:GT=hom:DP=1:FQ=1:SQ=-1;ID=S2663_raw:GT=hom:DP=9:FQ=1:SQ=-1;ID=S2011_raw:GT=hom:DP=2:FQ=1:SQ=-1;ID=S2504_raw:GT=hom:DP=8:FQ=1:SQ=-1;ID=S2008_raw:GT=hom:DP=1:FQ=1:SQ=-1;ID=S2509_raw:GT=hom:DP=6:FQ=1:SQ=-1;ID=S2508_raw:GT=hom:DP=9:FQ=1:SQ=-1;ID=S2666_raw:GT=hom:DP=11:FQ=1:SQ=-1;ID=S2669_raw:GT=hom:DP=6:FQ=1:SQ=-1;ID=S2166_raw:GT=hom:DP=12:FQ=1:SQ=-1;ID=S1775_raw:GT=hom:DP=8:FQ=1:SQ=-1;ID=S2501_raw:GT=hom:DP=7:FQ=1:SQ=-1;ID=S2500_raw:GT=hom:DP=10:FQ=1:SQ=-1;ID=S2502_raw:GT=hom:DP=14:FQ=1:SQ=-1;ID=S2505_raw:GT=hom:DP=12:FQ=1:SQ=-1;ID=S2176_raw:GT=hom:DP=11:FQ=1:SQ=-1;ID=S2506_raw:GT=hom:DP=9:FQ=1:SQ=-1;ID=S2667_raw:GT=hom:DP=13:FQ=1:SQ=-1;
8	27641609	27641609	G	A	intronic	ESCO2	.	.	rs1824449	Benign	ID=S2009_raw:GT=hom:DP=170:FQ=1:SQ=-1;ID=S2503_raw:GT=hom:DP=66:FQ=1:SQ=-1;ID=S2507_raw:GT=hom:DP=88:FQ=1:SQ=-1;ID=S2668_raw:GT=hom:DP=104:FQ=1:SQ=-1;ID=S2012_raw:GT=hom:DP=258:FQ=1:SQ=-1;ID=S2010_raw:GT=hom:DP=248:FQ=1:SQ=-1;ID=S2662_raw:GT=hom:DP=6:FQ=1:SQ=-1;ID=S2663_raw:GT=hom:DP=73:FQ=1:SQ=-1;ID=S2011_raw:GT=hom:DP=309:FQ=1:SQ=-1;ID=S2504_raw:GT=hom:DP=110:FQ=1:SQ=-1;ID=S2008_raw:GT=hom:DP=223:FQ=1:SQ=-1;ID=S2509_raw:GT=hom:DP=95:FQ=1:SQ=-1;ID=S2508_raw:GT=hom:DP=93:FQ=1:SQ=-1;ID=S2666_raw:GT=hom:DP=76:FQ=1:SQ=-1;ID=S2669_raw:GT=hom:DP=25:FQ=1:SQ=-1;ID=S2166_raw:GT=hom:DP=102:FQ=1:SQ=-1;ID=S1775_raw:GT=hom:DP=82:FQ=1:SQ=-1;ID=S2501_raw:GT=hom:DP=102:FQ=1:SQ=-1;ID=S2500_raw:GT=hom:DP=100:FQ=1:SQ=-1;ID=S2502_raw:GT=hom:DP=108:FQ=1:SQ=-1;ID=S2505_raw:GT=hom:DP=109:FQ=1:SQ=-1;ID=S2176_raw:GT=hom:DP=88:FQ=1:SQ=-1;ID=S2506_raw:GT=hom:DP=89:FQ=1:SQ=-1;ID=S2667_raw:GT=hom:DP=96:FQ=1:SQ=-1;
4	5577884	5577884	T	C	intronic	EVC2	.	.	rs6840591	.	ID=S2009_raw:GT=hom:DP=18:FQ=1:SQ=-1;ID=S2503_raw:GT=hom:DP=24:FQ=1:SQ=-1;ID=S2507_raw:GT=hom:DP=39:FQ=1:SQ=-1;ID=S2668_raw:GT=hom:DP=41:FQ=1:SQ=-1;ID=S2012_raw:GT=hom:DP=22:FQ=1:SQ=-1;ID=S2010_raw:GT=hom:DP=27:FQ=1:SQ=-1;ID=S2662_raw:GT=hom:DP=30:FQ=1:SQ=-1;ID=S2663_raw:GT=hom:DP=28:FQ=1:SQ=-1;ID=S2011_raw:GT=hom:DP=37:FQ=1:SQ=-1;ID=S2504_raw:GT=hom:DP=34:FQ=1:SQ=-1;ID=S2008_raw:GT=hom:DP=17:FQ=1:SQ=-1;ID=S2509_raw:GT=hom:DP=18:FQ=1:SQ=-1;ID=S2508_raw:GT=hom:DP=28:FQ=1:SQ=-1;ID=S2666_raw:GT=hom:DP=19:FQ=1:SQ=-1;ID=S2669_raw:GT=hom:DP=31:FQ=1:SQ=-1;ID=S2166_raw:GT=hom:DP=23:FQ=1:SQ=-1;ID=S1775_raw:GT=hom:DP=21:FQ=1:SQ=-1;ID=S2501_raw:GT=hom:DP=28:FQ=1:SQ=-1;ID=S2500_raw:GT=hom:DP=36:FQ=1:SQ=-1;ID=S2502_raw:GT=hom:DP=37:FQ=1:SQ=-1;ID=S2505_raw:GT=hom:DP=34:FQ=1:SQ=-1;ID=S2176_raw:GT=hom:DP=31:FQ=1:SQ=-1;ID=S2506_raw:GT=hom:DP=24:FQ=1:SQ=-1;ID=S2667_raw:GT=hom:DP=33:FQ=1:SQ=-1;
11	46760466	46760466	G	T	intronic	F2	.	.	rs3136515	.	ID=S2009_raw:GT=hom:DP=9:FQ=1:SQ=-1;ID=S2503_raw:GT=hom:DP=12:FQ=1:SQ=-1;ID=S2507_raw:GT=hom:DP=19:FQ=1:SQ=-1;ID=S2668_raw:GT=hom:DP=22:FQ=1:SQ=-1;ID=S2012_raw:GT=hom:DP=6:FQ=1:SQ=-1;ID=S2010_raw:GT=hom:DP=6:FQ=1:SQ=-1;ID=S2662_raw:GT=hom:DP=4:FQ=1:SQ=-1;ID=S2663_raw:GT=hom:DP=11:FQ=1:SQ=-1;ID=S2011_raw:GT=hom:DP=12:FQ=1:SQ=-1;ID=S2504_raw:GT=hom:DP=27:FQ=1:SQ=-1;ID=S2008_raw:GT=hom:DP=8:FQ=1:SQ=-1;ID=S2509_raw:GT=hom:DP=12:FQ=1:SQ=-1;ID=S2508_raw:GT=hom:DP=14:FQ=1:SQ=-1;ID=S2666_raw:GT=hom:DP=15:FQ=1:SQ=-1;ID=S2669_raw:GT=hom:DP=6:FQ=1:SQ=-1;ID=S2166_raw:GT=hom:DP=10:FQ=1:SQ=-1;ID=S1775_raw:GT=hom:DP=10:FQ=1:SQ=-1;ID=S2501_raw:GT=hom:DP=14:FQ=1:SQ=-1;ID=S2500_raw:GT=hom:DP=18:FQ=1:SQ=-1;ID=S2502_raw:GT=hom:DP=13:FQ=1:SQ=-1;ID=S2505_raw:GT=hom:DP=18:FQ=1:SQ=-1;ID=S2176_raw:GT=hom:DP=21:FQ=1:SQ=-1;ID=S2506_raw:GT=hom:DP=20:FQ=1:SQ=-1;ID=S2667_raw:GT=hom:DP=16:FQ=1:SQ=-1;
13	1,14E+08	1,14E+08	A	G	intronic	F10	.	.	rs2031184	.	ID=S2009_raw:GT=hom:DP=62:FQ=1:SQ=-1;ID=S2503_raw:GT=hom:DP=199:FQ=1:SQ=-1;ID=S2507_raw:GT=hom:DP=209:FQ=1:SQ=-1;ID=S2668_raw:GT=hom:DP=228:FQ=1:SQ=-1;ID=S2012_raw:GT=hom:DP=69:FQ=1:SQ=-1;ID=S2010_raw:GT=hom:DP=52:FQ=1:SQ=-1;ID=S2662_raw:GT=hom:DP=317:FQ=1:SQ=-1;ID=S2663_raw:GT=hom:DP=211:FQ=1:SQ=-1;ID=S2011_raw:GT=hom:DP=68:FQ=1:SQ=-1;ID=S2504_raw:GT=hom:DP=229:FQ=1:SQ=-1;ID=S2008_raw:GT=hom:DP=78:FQ=1:SQ=-1;ID=S2509_raw:GT=hom:DP=164:FQ=1:SQ=-1;ID=S2508_raw:GT=hom:DP=243:FQ=1:SQ=-1;ID=S2666_raw:GT=hom:DP=185:FQ=1:SQ=-1;ID=S2669_raw:GT=hom:DP=213:FQ=1:SQ=-1;ID=S2166_raw:GT=hom:DP=77:FQ=1:SQ=-1;ID=S1775_raw:GT=hom:DP=66:FQ=1:SQ=-1;ID=S2501_raw:GT=hom:DP=186:FQ=1:SQ=-1;ID=S2500_raw:GT=hom:DP=208:FQ=1:SQ=-1;ID=S2502_raw:GT=hom:DP=195:FQ=1:SQ=-1;ID=S2505_raw:GT=hom:DP=175:FQ=1:SQ=-1;ID=S2176_raw:GT=hom:DP=74:FQ=1:SQ=-1;ID=S2506_raw:GT=hom:DP=198:FQ=1:SQ=-1;ID=S2667_raw:GT=hom:DP=253:FQ=1:SQ=-1;
1	1,73E+08	1,73E+08	T	C	intronic	FASLG	.	.	rs2639653	.	ID=S2009_raw:GT=hom:DP=33:FQ=1:SQ=-1;ID=S2503_raw:GT=hom:DP=81:FQ=1:SQ=-1;ID=S2507_raw:GT=hom:DP=67:FQ=1:SQ=-1;ID=S2668_raw:GT=hom:DP=110:FQ=1:SQ=-1;ID=S2012_raw:GT=hom:DP=39:FQ=1:SQ=-1;ID=S2010_raw:GT=hom:DP=53:FQ=1:SQ=-1;ID=S2662_raw:GT=hom:DP=25:FQ=1:SQ=-1;ID=S2663_raw:GT=hom:DP=74:FQ=1:SQ=-1;ID=S2011_raw:GT=hom:DP=69:FQ=1:SQ=-1;ID=S2504_raw:GT=hom:DP=113:FQ=1:SQ=-1;ID=S2008_raw:GT=hom:DP=47:FQ=1:SQ=-1;ID=S2509_raw:GT=hom:DP=78:FQ=1:SQ=-1;ID=S2508_raw:GT=hom:DP=95:FQ=0.99:SQ=-1;ID=S2666_raw:GT=hom:DP=113:FQ=1:SQ=-1;ID=S2669_raw:GT=hom:DP=87:FQ=1:SQ=-1;ID=S2166_raw:GT=hom:DP=83:FQ=1:SQ=-1;ID=S1775_raw:GT=hom:DP=51:FQ=1:SQ=-1;ID=S2501_raw:GT=hom:DP=89:FQ=1:SQ=-1;ID=S2500_raw:GT=hom:DP=65:FQ=1:SQ=-1;ID=S2502_raw:GT=hom:DP=84:FQ=1:SQ=-1;ID=S2505_raw:GT=hom:DP=73:FQ=1:SQ=-1;ID=S2176_raw:GT=hom:DP=78:FQ=1:SQ=-1;ID=S2506_raw:GT=hom:DP=67:FQ=1:SQ=-1;ID=S2667_raw:GT=hom:DP=78:FQ=1:SQ=-1;
4	1,26E+08	1,26E+08	A	G	intronic	FAT4	.	.	rs925242	.	ID=S2009_raw:GT=hom:DP=5:FQ=1:SQ=-1;ID=S2503_raw:GT=hom:DP=4:FQ=1:SQ=-1;ID=S2507_raw:GT=hom:DP=7:FQ=1:SQ=-1;ID=S2668_raw:GT=hom:DP=4:FQ=1:SQ=-1;ID=S2012_raw:GT=hom:DP=4:FQ=1:SQ=-1;ID=S2010_raw:GT=hom:DP=6:FQ=1:SQ=-1;ID=S2662_raw:GT=hom:DP=2:FQ=1:SQ=-1;ID=S2663_raw:GT=hom:DP=5:FQ=1:SQ=-1;ID=S2011_raw:GT=hom:DP=10:FQ=1:SQ=-1;ID=S2504_raw:GT=hom:DP=11:FQ=1:SQ=-1;ID=S2008_raw:GT=hom:DP=10:FQ=1:SQ=-1;ID=S2509_raw:GT=hom:DP=8:FQ=1:SQ=-1;ID=S2508_raw:GT=hom:DP=10:FQ=1:SQ=-1;ID=S2666_raw:GT=hom:DP=3:FQ=1:SQ=-1;ID=S2669_raw:GT=hom:DP=2:FQ=1:SQ=-1;ID=S2166_raw:GT=hom:DP=24:FQ=1:SQ=-1;ID=S1775_raw:GT=hom:DP=6:FQ=1:SQ=-1;ID=S2501_raw:GT=hom:DP=7:FQ=1:SQ=-1;ID=S2500_raw:GT=hom:DP=13:FQ=1:SQ=-1;ID=S2502_raw:GT=hom:DP=11:FQ=1:SQ=-1;ID=S2505_raw:GT=hom:DP=10:FQ=1:SQ=-1;ID=S2176_raw:GT=hom:DP=32:FQ=1:SQ=-1;ID=S2506_raw:GT=hom:DP=7:FQ=1:SQ=-1;ID=S2667_raw:GT=hom:DP=8:FQ=1:SQ=-1;
4	1,26E+08	1,26E+08	T	C	exonic	FAT4	synonymous SNV	FAT4:NM_001291285:exon4:c.5760T>C:p.D1920D,FAT4:NM_001291303:exon4:c.5760T>C:p.D1920D,FAT4:NM_024582:exon4:c.5760T>C:p.D1920D	rs958415	Benign	ID=S2009_raw:GT=hom:DP=62:FQ=1:SQ=-1;ID=S2503_raw:GT=hom:DP=73:FQ=0.99:SQ=-1;ID=S2507_raw:GT=hom:DP=73:FQ=1:SQ=-1;ID=S2668_raw:GT=hom:DP=110:FQ=1:SQ=-1;ID=S2012_raw:GT=hom:DP=58:FQ=1:SQ=-1;ID=S2010_raw:GT=hom:DP=72:FQ=1:SQ=-1;ID=S2662_raw:GT=hom:DP=1:FQ=1:SQ=-1;ID=S2663_raw:GT=hom:DP=71:FQ=1:SQ=-1;ID=S2011_raw:GT=hom:DP=121:FQ=1:SQ=-1;ID=S2504_raw:GT=hom:DP=85:FQ=1:SQ=-1;ID=S2008_raw:GT=hom:DP=87:FQ=1:SQ=-1;ID=S2509_raw:GT=hom:DP=80:FQ=1:SQ=-1;ID=S2508_raw:GT=hom:DP=72:FQ=1:SQ=-1;ID=S2666_raw:GT=hom:DP=147:FQ=1:SQ=-1;ID=S2669_raw:GT=hom:DP=17:FQ=1:SQ=-1;ID=S2166_raw:GT=hom:DP=110:FQ=1:SQ=-1;ID=S1775_raw:GT=hom:DP=85:FQ=1:SQ=-1;ID=S2501_raw:GT=hom:DP=63:FQ=1:SQ=-1;ID=S2500_raw:GT=hom:DP=53:FQ=1:SQ=-1;ID=S2502_raw:GT=hom:DP=71:FQ=1:SQ=-1;ID=S2505_raw:GT=hom:DP=65:FQ=1:SQ=-1;ID=S2176_raw:GT=hom:DP=114:FQ=1:SQ=-1;ID=S2506_raw:GT=hom:DP=58:FQ=1:SQ=-1;ID=S2667_raw:GT=hom:DP=68:FQ=1:SQ=-1;
4	1,26E+08	1,26E+08	G	C	intronic	FAT4	.	.	rs4605670	.	ID=S2009_raw:GT=hom:DP=13:FQ=1:SQ=-1;ID=S2503_raw:GT=hom:DP=51:FQ=1:SQ=-1;ID=S2507_raw:GT=hom:DP=29:FQ=1:SQ=-1;ID=S2668_raw:GT=hom:DP=51:FQ=1:SQ=-1;ID=S2012_raw:GT=hom:DP=29:FQ=1:SQ=-1;ID=S2010_raw:GT=hom:DP=30:FQ=1:SQ=-1;ID=S2662_raw:GT=hom:DP=2:FQ=1:SQ=-1;ID=S2663_raw:GT=hom:DP=38:FQ=1:SQ=-1;ID=S2011_raw:GT=hom:DP=36:FQ=1:SQ=-1;ID=S2504_raw:GT=hom:DP=52:FQ=1:SQ=-1;ID=S2008_raw:GT=hom:DP=22:FQ=1:SQ=-1;ID=S2509_raw:GT=hom:DP=37:FQ=1:SQ=-1;ID=S2508_raw:GT=hom:DP=50:FQ=1:SQ=-1;ID=S2666_raw:GT=hom:DP=48:FQ=1:SQ=-1;ID=S2669_raw:GT=hom:DP=26:FQ=1:SQ=-1;ID=S2166_raw:GT=hom:DP=58:FQ=1:SQ=-1;ID=S1775_raw:GT=hom:DP=22:FQ=1:SQ=-1;ID=S2501_raw:GT=hom:DP=55:FQ=1:SQ=-1;ID=S2500_raw:GT=hom:DP=49:FQ=1:SQ=-1;ID=S2502_raw:GT=hom:DP=45:FQ=1:SQ=-1;ID=S2505_raw:GT=hom:DP=49:FQ=1:SQ=-1;ID=S2176_raw:GT=hom:DP=64:FQ=1:SQ=-1;ID=S2506_raw:GT=hom:DP=39:FQ=1:SQ=-1;ID=S2667_raw:GT=hom:DP=36:FQ=1:SQ=-1;
4	1,26E+08	1,26E+08	T	G	intronic	FAT4	.	.	rs7656135	.	ID=S2009_raw:GT=hom:DP=36:FQ=1:SQ=-1;ID=S2503_raw:GT=hom:DP=24:FQ=1:SQ=-1;ID=S2507_raw:GT=hom:DP=22:FQ=1:SQ=-1;ID=S2668_raw:GT=hom:DP=33:FQ=1:SQ=-1;ID=S2012_raw:GT=hom:DP=47:FQ=1:SQ=-1;ID=S2010_raw:GT=hom:DP=63:FQ=1:SQ=-1;ID=S2662_raw:GT=hom:DP=1:FQ=1:SQ=-1;ID=S2663_raw:GT=hom:DP=15:FQ=1:SQ=-1;ID=S2011_raw:GT=hom:DP=78:FQ=1:SQ=-1;ID=S2504_raw:GT=hom:DP=24:FQ=1:SQ=-1;ID=S2008_raw:GT=hom:DP=57:FQ=1:SQ=-1;ID=S2509_raw:GT=hom:DP=38:FQ=1:SQ=-1;ID=S2508_raw:GT=hom:DP=40:FQ=1:SQ=-1;ID=S2666_raw:GT=hom:DP=34:FQ=1:SQ=-1;ID=S2669_raw:GT=hom:DP=6:FQ=1:SQ=-1;ID=S2166_raw:GT=hom:DP=111:FQ=1:SQ=-1;ID=S1775_raw:GT=hom:DP=64:FQ=1:SQ=-1;ID=S2501_raw:GT=hom:DP=29:FQ=1:SQ=-1;ID=S2500_raw:GT=hom:DP=29:FQ=1:SQ=-1;ID=S2502_raw:GT=hom:DP=28:FQ=1:SQ=-1;ID=S2505_raw:GT=hom:DP=40:FQ=1:SQ=-1;ID=S2176_raw:GT=hom:DP=120:FQ=1:SQ=-1;ID=S2506_raw:GT=hom:DP=17:FQ=1:SQ=-1;ID=S2667_raw:GT=hom:DP=30:FQ=1:SQ=-1;
4	1,26E+08	1,26E+08	G	C	exonic	FAT4	synonymous SNV	FAT4:NM_001291285:exon9:c.7707G>C:p.V2569V,FAT4:NM_001291303:exon9:c.7707G>C:p.V2569V,FAT4:NM_024582:exon9:c.7701G>C:p.V2567V	rs988863	Benign	ID=S2009_raw:GT=hom:DP=116:FQ=1:SQ=-1;ID=S2503_raw:GT=hom:DP=126:FQ=1:SQ=-1;ID=S2507_raw:GT=hom:DP=128:FQ=1:SQ=-1;ID=S2668_raw:GT=hom:DP=147:FQ=1:SQ=-1;ID=S2012_raw:GT=hom:DP=135:FQ=1:SQ=-1;ID=S2010_raw:GT=hom:DP=161:FQ=1:SQ=-1;ID=S2662_raw:GT=hom:DP=11:FQ=1:SQ=-1;ID=S2663_raw:GT=hom:DP=122:FQ=1:SQ=-1;ID=S2011_raw:GT=hom:DP=228:FQ=1:SQ=-1;ID=S2504_raw:GT=hom:DP=141:FQ=1:SQ=-1;ID=S2008_raw:GT=hom:DP=153:FQ=0.99:SQ=-1;ID=S2509_raw:GT=hom:DP=115:FQ=1:SQ=-1;ID=S2508_raw:GT=hom:DP=137:FQ=1:SQ=-1;ID=S2666_raw:GT=hom:DP=134:FQ=1:SQ=-1;ID=S2669_raw:GT=hom:DP=100:FQ=1:SQ=-1;ID=S2166_raw:GT=hom:DP=367:FQ=1:SQ=-1;ID=S1775_raw:GT=hom:DP=227:FQ=1:SQ=-1;ID=S2501_raw:GT=hom:DP=126:FQ=1:SQ=-1;ID=S2500_raw:GT=hom:DP=116:FQ=1:SQ=-1;ID=S2502_raw:GT=hom:DP=136:FQ=1:SQ=-1;ID=S2505_raw:GT=hom:DP=113:FQ=0.99:SQ=-1;ID=S2176_raw:GT=hom:DP=393:FQ=1:SQ=-1;ID=S2506_raw:GT=hom:DP=90:FQ=1:SQ=-1;ID=S2667_raw:GT=hom:DP=94:FQ=1:SQ=-1;
4	1,26E+08	1,26E+08	G	A	exonic	FAT4	nonsynonymous SNV	FAT4:NM_001291285:exon9:c.11624G>A:p.S3875N,FAT4:NM_001291303:exon9:c.11624G>A:p.S3875N,FAT4:NM_024582:exon9:c.11618G>A:p.S3873N	rs12650153	Benign	ID=S2009_raw:GT=hom:DP=54:FQ=1:SQ=-1;ID=S2503_raw:GT=hom:DP=66:FQ=1:SQ=-1;ID=S2507_raw:GT=hom:DP=49:FQ=1:SQ=-1;ID=S2668_raw:GT=hom:DP=77:FQ=1:SQ=-1;ID=S2012_raw:GT=hom:DP=76:FQ=1:SQ=-1;ID=S2010_raw:GT=hom:DP=83:FQ=1:SQ=-1;ID=S2662_raw:GT=hom:DP=24:FQ=1:SQ=-1;ID=S2663_raw:GT=hom:DP=68:FQ=1:SQ=-1;ID=S2011_raw:GT=hom:DP=109:FQ=1:SQ=-1;ID=S2504_raw:GT=hom:DP=86:FQ=1:SQ=-1;ID=S2008_raw:GT=hom:DP=82:FQ=1:SQ=-1;ID=S2509_raw:GT=hom:DP=56:FQ=1:SQ=-1;ID=S2508_raw:GT=hom:DP=64:FQ=1:SQ=-1;ID=S2666_raw:GT=hom:DP=98:FQ=1:SQ=-1;ID=S2669_raw:GT=hom:DP=39:FQ=1:SQ=-1;ID=S2166_raw:GT=hom:DP=158:FQ=1:SQ=-1;ID=S1775_raw:GT=hom:DP=94:FQ=1:SQ=-1;ID=S2501_raw:GT=hom:DP=70:FQ=1:SQ=-1;ID=S2500_raw:GT=hom:DP=72:FQ=1:SQ=-1;ID=S2502_raw:GT=hom:DP=88:FQ=1:SQ=-1;ID=S2505_raw:GT=hom:DP=60:FQ=1:SQ=-1;ID=S2176_raw:GT=hom:DP=192:FQ=1:SQ=-1;ID=S2506_raw:GT=hom:DP=40:FQ=1:SQ=-1;ID=S2667_raw:GT=hom:DP=59:FQ=1:SQ=-1;
4	1,26E+08	1,26E+08	A	G	intronic	FAT4	.	.	rs6814158	.	ID=S2009_raw:GT=hom:DP=12:FQ=1:SQ=-1;ID=S2503_raw:GT=hom:DP=26:FQ=1:SQ=-1;ID=S2507_raw:GT=hom:DP=22:FQ=1:SQ=-1;ID=S2668_raw:GT=hom:DP=28:FQ=1:SQ=-1;ID=S2012_raw:GT=hom:DP=22:FQ=1:SQ=-1;ID=S2010_raw:GT=hom:DP=10:FQ=1:SQ=-1;ID=S2662_raw:GT=hom:DP=5:FQ=1:SQ=-1;ID=S2663_raw:GT=hom:DP=38:FQ=1:SQ=-1;ID=S2011_raw:GT=hom:DP=15:FQ=1:SQ=-1;ID=S2504_raw:GT=hom:DP=24:FQ=1:SQ=-1;ID=S2008_raw:GT=hom:DP=13:FQ=1:SQ=-1;ID=S2509_raw:GT=hom:DP=27:FQ=1:SQ=-1;ID=S2508_raw:GT=hom:DP=22:FQ=1:SQ=-1;ID=S2666_raw:GT=hom:DP=26:FQ=1:SQ=-1;ID=S2669_raw:GT=hom:DP=43:FQ=1:SQ=-1;ID=S2166_raw:GT=hom:DP=50:FQ=1:SQ=-1;ID=S1775_raw:GT=hom:DP=31:FQ=1:SQ=-1;ID=S2501_raw:GT=hom:DP=30:FQ=1:SQ=-1;ID=S2500_raw:GT=hom:DP=18:FQ=1:SQ=-1;ID=S2502_raw:GT=hom:DP=21:FQ=1:SQ=-1;ID=S2505_raw:GT=hom:DP=25:FQ=1:SQ=-1;ID=S2176_raw:GT=hom:DP=51:FQ=1:SQ=-1;ID=S2506_raw:GT=hom:DP=51:FQ=1:SQ=-1;ID=S2667_raw:GT=hom:DP=37:FQ=1:SQ=-1;
15	48807637	48807637	C	T	exonic	FBN1	nonsynonymous SNV	FBN1:NM_000138:exon12:c.1415G>A:p.C472Y	rs4775765	Benign	ID=S2009_raw:GT=hom:DP=90:FQ=1:SQ=-1;ID=S2503_raw:GT=hom:DP=115:FQ=1:SQ=-1;ID=S2507_raw:GT=hom:DP=122:FQ=1:SQ=-1;ID=S2668_raw:GT=hom:DP=184:FQ=1:SQ=-1;ID=S2012_raw:GT=hom:DP=119:FQ=1:SQ=-1;ID=S2010_raw:GT=hom:DP=129:FQ=1:SQ=-1;ID=S2662_raw:GT=hom:DP=64:FQ=1:SQ=-1;ID=S2663_raw:GT=hom:DP=117:FQ=1:SQ=-1;ID=S2011_raw:GT=hom:DP=155:FQ=1:SQ=-1;ID=S2504_raw:GT=hom:DP=131:FQ=1:SQ=-1;ID=S2008_raw:GT=hom:DP=136:FQ=1:SQ=-1;ID=S2509_raw:GT=hom:DP=103:FQ=1:SQ=-1;ID=S2508_raw:GT=hom:DP=125:FQ=1:SQ=-1;ID=S2666_raw:GT=hom:DP=144:FQ=1:SQ=-1;ID=S2669_raw:GT=hom:DP=135:FQ=1:SQ=-1;ID=S2166_raw:GT=hom:DP=140:FQ=1:SQ=-1;ID=S1775_raw:GT=hom:DP=140:FQ=1:SQ=-1;ID=S2501_raw:GT=hom:DP=116:FQ=1:SQ=-1;ID=S2500_raw:GT=hom:DP=131:FQ=0.99:SQ=-1;ID=S2502_raw:GT=hom:DP=121:FQ=1:SQ=-1;ID=S2505_raw:GT=hom:DP=131:FQ=1:SQ=-1;ID=S2176_raw:GT=hom:DP=148:FQ=1:SQ=-1;ID=S2506_raw:GT=hom:DP=112:FQ=1:SQ=-1;ID=S2667_raw:GT=hom:DP=134:FQ=1:SQ=-1;
1	1,62E+08	1,62E+08	G	C	UTR5	FCGR3B	.	NM_001271037:c.-620C>G;NM_001271036:c.-620C>G	rs3883928	.	ID=S2009_raw:GT=hom:DP=10:FQ=1:SQ=-1;ID=S2503_raw:GT=hom:DP=7:FQ=1:SQ=-1;ID=S2507_raw:GT=hom:DP=9:FQ=1:SQ=-1;ID=S2668_raw:GT=hom:DP=1:FQ=1:SQ=-1;ID=S2012_raw:GT=hom:DP=14:FQ=1:SQ=-1;ID=S2010_raw:GT=hom:DP=29:FQ=1:SQ=-1;ID=S2662_raw:GT=hom:DP=3:FQ=1:SQ=-1;ID=S2663_raw:GT=hom:DP=6:FQ=1:SQ=-1;ID=S2011_raw:GT=hom:DP=18:FQ=1:SQ=-1;ID=S2504_raw:GT=hom:DP=1:FQ=1:SQ=-1;ID=S2008_raw:GT=hom:DP=17:FQ=1:SQ=-1;ID=S2509_raw:GT=hom:DP=5:FQ=1:SQ=-1;ID=S2508_raw:GT=hom:DP=6:FQ=1:SQ=-1;ID=S2666_raw:GT=hom:DP=5:FQ=1:SQ=-1;ID=S2669_raw:GT=hom:DP=5:FQ=1:SQ=-1;ID=S2166_raw:GT=hom:DP=17:FQ=1:SQ=-1;ID=S1775_raw:GT=hom:DP=8:FQ=1:SQ=-1;ID=S2501_raw:GT=hom:DP=10:FQ=1:SQ=-1;ID=S2500_raw:GT=hom:DP=11:FQ=1:SQ=-1;ID=S2502_raw:GT=hom:DP=9:FQ=1:SQ=-1;ID=S2505_raw:GT=hom:DP=3:FQ=1:SQ=-1;ID=S2176_raw:GT=hom:DP=7:FQ=1:SQ=-1;ID=S2506_raw:GT=hom:DP=4:FQ=1:SQ=-1;ID=S2667_raw:GT=hom:DP=4:FQ=1:SQ=-1;
1	1,62E+08	1,62E+08	T	C	intronic	FCGR3B	.	.	rs61803027	.	ID=S2009_raw:GT=hom:DP=30:FQ=1:SQ=-1;ID=S2503_raw:GT=hom:DP=30:FQ=1:SQ=-1;ID=S2507_raw:GT=hom:DP=29:FQ=1:SQ=-1;ID=S2668_raw:GT=hom:DP=15:FQ=1:SQ=-1;ID=S2012_raw:GT=hom:DP=106:FQ=1:SQ=-1;ID=S2010_raw:GT=hom:DP=122:FQ=1:SQ=-1;ID=S2662_raw:GT=hom:DP=7:FQ=1:SQ=-1;ID=S2663_raw:GT=hom:DP=32:FQ=1:SQ=-1;ID=S2011_raw:GT=hom:DP=123:FQ=1:SQ=-1;ID=S2504_raw:GT=hom:DP=11:FQ=1:SQ=-1;ID=S2008_raw:GT=hom:DP=94:FQ=1:SQ=-1;ID=S2509_raw:GT=hom:DP=18:FQ=1:SQ=-1;ID=S2508_raw:GT=hom:DP=22:FQ=1:SQ=-1;ID=S2666_raw:GT=hom:DP=12:FQ=1:SQ=-1;ID=S2669_raw:GT=hom:DP=17:FQ=1:SQ=-1;ID=S2166_raw:GT=hom:DP=27:FQ=1:SQ=-1;ID=S1775_raw:GT=hom:DP=32:FQ=1:SQ=-1;ID=S2501_raw:GT=hom:DP=29:FQ=1:SQ=-1;ID=S2500_raw:GT=hom:DP=22:FQ=1:SQ=-1;ID=S2502_raw:GT=hom:DP=18:FQ=1:SQ=-1;ID=S2505_raw:GT=hom:DP=21:FQ=1:SQ=-1;ID=S2176_raw:GT=hom:DP=37:FQ=1:SQ=-1;ID=S2506_raw:GT=hom:DP=19:FQ=1:SQ=-1;ID=S2667_raw:GT=hom:DP=3:FQ=1:SQ=-1;
7	1,22E+08	1,22E+08	-	ACA	intronic	FEZF1	.	.	.	.	ID=S2009_raw:GT=hom:DP=124:FQ=1:SQ=-1;ID=S2503_raw:GT=hom:DP=66:FQ=1:SQ=-1;ID=S2507_raw:GT=hom:DP=77:FQ=1:SQ=-1;ID=S2668_raw:GT=hom:DP=58:FQ=1:SQ=-1;ID=S2012_raw:GT=hom:DP=109:FQ=1:SQ=-1;ID=S2010_raw:GT=hom:DP=128:FQ=1:SQ=-1;ID=S2662_raw:GT=hom:DP=20:FQ=1:SQ=-1;ID=S2663_raw:GT=hom:DP=54:FQ=1:SQ=-1;ID=S2011_raw:GT=hom:DP=166:FQ=1:SQ=-1;ID=S2504_raw:GT=hom:DP=80:FQ=1:SQ=-1;ID=S2008_raw:GT=hom:DP=125:FQ=1:SQ=-1;ID=S2509_raw:GT=hom:DP=78:FQ=1:SQ=-1;ID=S2508_raw:GT=hom:DP=87:FQ=1:SQ=-1;ID=S2666_raw:GT=hom:DP=99:FQ=1:SQ=-1;ID=S2669_raw:GT=hom:DP=50:FQ=1:SQ=-1;ID=S2166_raw:GT=hom:DP=191:FQ=1:SQ=-1;ID=S1775_raw:GT=hom:DP=129:FQ=1:SQ=-1;ID=S2501_raw:GT=hom:DP=70:FQ=1:SQ=-1;ID=S2500_raw:GT=hom:DP=59:FQ=1:SQ=-1;ID=S2502_raw:GT=hom:DP=100:FQ=1:SQ=-1;ID=S2505_raw:GT=hom:DP=83:FQ=1:SQ=-1;ID=S2176_raw:GT=hom:DP=199:FQ=1:SQ=-1;ID=S2506_raw:GT=hom:DP=70:FQ=1:SQ=-1;ID=S2667_raw:GT=hom:DP=47:FQ=1:SQ=-1;
X	1,38E+08	1,38E+08	A	T	intronic	FGF13	.	.	rs5976185	.	ID=S2009_raw:GT=hom:DP=30:FQ=1:SQ=-1;ID=S2503_raw:GT=hom:DP=16:FQ=1:SQ=-1;ID=S2507_raw:GT=hom:DP=18:FQ=1:SQ=-1;ID=S2668_raw:GT=hom:DP=103:FQ=1:SQ=-1;ID=S2012_raw:GT=hom:DP=35:FQ=1:SQ=-1;ID=S2010_raw:GT=hom:DP=60:FQ=1:SQ=-1;ID=S2662_raw:GT=hom:DP=8:FQ=1:SQ=-1;ID=S2663_raw:GT=hom:DP=56:FQ=1:SQ=-1;ID=S2011_raw:GT=hom:DP=62:FQ=1:SQ=-1;ID=S2504_raw:GT=hom:DP=29:FQ=1:SQ=-1;ID=S2008_raw:GT=hom:DP=70:FQ=1:SQ=-1;ID=S2509_raw:GT=hom:DP=21:FQ=1:SQ=-1;ID=S2508_raw:GT=hom:DP=25:FQ=1:SQ=-1;ID=S2666_raw:GT=hom:DP=51:FQ=1:SQ=-1;ID=S2669_raw:GT=hom:DP=22:FQ=1:SQ=-1;ID=S2166_raw:GT=hom:DP=12:FQ=1:SQ=-1;ID=S1775_raw:GT=hom:DP=13:FQ=1:SQ=-1;ID=S2501_raw:GT=hom:DP=21:FQ=1:SQ=-1;ID=S2500_raw:GT=hom:DP=31:FQ=1:SQ=-1;ID=S2502_raw:GT=hom:DP=75:FQ=1:SQ=-1;ID=S2505_raw:GT=hom:DP=40:FQ=1:SQ=-1;ID=S2176_raw:GT=hom:DP=18:FQ=1:SQ=-1;ID=S2506_raw:GT=hom:DP=23:FQ=1:SQ=-1;ID=S2667_raw:GT=hom:DP=52:FQ=1:SQ=-1;
10	1,23E+08	1,23E+08	C	T	intronic	FGFR2	.	.	rs2981448	.	ID=S2009_raw:GT=hom:DP=8:FQ=1:SQ=-1;ID=S2503_raw:GT=hom:DP=38:FQ=1:SQ=-1;ID=S2507_raw:GT=hom:DP=39:FQ=1:SQ=-1;ID=S2668_raw:GT=hom:DP=39:FQ=1:SQ=-1;ID=S2012_raw:GT=hom:DP=5:FQ=1:SQ=-1;ID=S2010_raw:GT=hom:DP=6:FQ=1:SQ=-1;ID=S2662_raw:GT=hom:DP=50:FQ=1:SQ=-1;ID=S2663_raw:GT=hom:DP=50:FQ=1:SQ=-1;ID=S2011_raw:GT=hom:DP=11:FQ=1:SQ=-1;ID=S2504_raw:GT=hom:DP=51:FQ=1:SQ=-1;ID=S2008_raw:GT=hom:DP=12:FQ=1:SQ=-1;ID=S2509_raw:GT=hom:DP=25:FQ=1:SQ=-1;ID=S2508_raw:GT=hom:DP=49:FQ=1:SQ=-1;ID=S2666_raw:GT=hom:DP=45:FQ=1:SQ=-1;ID=S2669_raw:GT=hom:DP=35:FQ=1:SQ=-1;ID=S2166_raw:GT=hom:DP=9:FQ=1:SQ=-1;ID=S1775_raw:GT=hom:DP=8:FQ=1:SQ=-1;ID=S2501_raw:GT=hom:DP=36:FQ=1:SQ=-1;ID=S2500_raw:GT=hom:DP=39:FQ=1:SQ=-1;ID=S2502_raw:GT=hom:DP=48:FQ=1:SQ=-1;ID=S2505_raw:GT=hom:DP=58:FQ=1:SQ=-1;ID=S2176_raw:GT=hom:DP=13:FQ=1:SQ=-1;ID=S2506_raw:GT=hom:DP=33:FQ=1:SQ=-1;ID=S2667_raw:GT=hom:DP=36:FQ=1:SQ=-1;
4	1807894	1807894	G	A	exonic;splicing	FGFR3	synonymous SNV	FGFR3:NM_022965:exon12:c.1617G>A:p.T539T,FGFR3:NM_000142:exon14:c.1953G>A:p.T651T,FGFR3:NM_001163213:exon14:c.1959G>A:p.T653T,FGFR3:NM_001354809:exon14:c.1956G>A:p.T652T,FGFR3:NM_001354810:exon14:c.1956G>A:p.T652T	rs7688609	.	ID=S2009_raw:GT=hom:DP=129:FQ=1:SQ=-1;ID=S2503_raw:GT=hom:DP=203:FQ=1:SQ=-1;ID=S2507_raw:GT=hom:DP=272:FQ=1:SQ=-1;ID=S2668_raw:GT=hom:DP=278:FQ=1:SQ=-1;ID=S2012_raw:GT=hom:DP=158:FQ=0.99:SQ=-1;ID=S2010_raw:GT=hom:DP=154:FQ=1:SQ=-1;ID=S2662_raw:GT=hom:DP=504:FQ=1:SQ=-1;ID=S2663_raw:GT=hom:DP=286:FQ=1:SQ=-1;ID=S2011_raw:GT=hom:DP=180:FQ=1:SQ=-1;ID=S2504_raw:GT=hom:DP=292:FQ=1:SQ=-1;ID=S2008_raw:GT=hom:DP=152:FQ=1:SQ=-1;ID=S2509_raw:GT=hom:DP=237:FQ=1:SQ=-1;ID=S2508_raw:GT=hom:DP=297:FQ=1:SQ=-1;ID=S2666_raw:GT=hom:DP=253:FQ=1:SQ=-1;ID=S2669_raw:GT=hom:DP=261:FQ=1:SQ=-1;ID=S2166_raw:GT=hom:DP=164:FQ=1:SQ=-1;ID=S1775_raw:GT=hom:DP=105:FQ=1:SQ=-1;ID=S2501_raw:GT=hom:DP=243:FQ=1:SQ=-1;ID=S2500_raw:GT=hom:DP=213:FQ=1:SQ=-1;ID=S2502_raw:GT=hom:DP=246:FQ=1:SQ=-1;ID=S2505_raw:GT=hom:DP=246:FQ=1:SQ=-1;ID=S2176_raw:GT=hom:DP=213:FQ=1:SQ=-1;ID=S2506_raw:GT=hom:DP=258:FQ=1:SQ=-1;ID=S2667_raw:GT=hom:DP=254:FQ=1:SQ=-1;
11	1,29E+08	1,29E+08	G	T	intronic	FLI1	.	.	rs639336	.	ID=S2009_raw:GT=hom:DP=99:FQ=1:SQ=-1;ID=S2503_raw:GT=hom:DP=60:FQ=1:SQ=-1;ID=S2507_raw:GT=hom:DP=75:FQ=1:SQ=-1;ID=S2668_raw:GT=hom:DP=51:FQ=1:SQ=-1;ID=S2012_raw:GT=hom:DP=104:FQ=1:SQ=-1;ID=S2010_raw:GT=hom:DP=119:FQ=1:SQ=-1;ID=S2662_raw:GT=hom:DP=27:FQ=1:SQ=-1;ID=S2663_raw:GT=hom:DP=43:FQ=1:SQ=-1;ID=S2011_raw:GT=hom:DP=132:FQ=1:SQ=-1;ID=S2504_raw:GT=hom:DP=58:FQ=1:SQ=-1;ID=S2008_raw:GT=hom:DP=106:FQ=1:SQ=-1;ID=S2509_raw:GT=hom:DP=52:FQ=1:SQ=-1;ID=S2508_raw:GT=hom:DP=78:FQ=1:SQ=-1;ID=S2666_raw:GT=hom:DP=53:FQ=1:SQ=-1;ID=S2669_raw:GT=hom:DP=34:FQ=1:SQ=-1;ID=S2166_raw:GT=hom:DP=145:FQ=1:SQ=-1;ID=S1775_raw:GT=hom:DP=98:FQ=1:SQ=-1;ID=S2501_raw:GT=hom:DP=58:FQ=1:SQ=-1;ID=S2500_raw:GT=hom:DP=63:FQ=1:SQ=-1;ID=S2502_raw:GT=hom:DP=61:FQ=1:SQ=-1;ID=S2505_raw:GT=hom:DP=44:FQ=1:SQ=-1;ID=S2176_raw:GT=hom:DP=158:FQ=1:SQ=-1;ID=S2506_raw:GT=hom:DP=32:FQ=1:SQ=-1;ID=S2667_raw:GT=hom:DP=50:FQ=1:SQ=-1;
13	28886113	28886113	A	G	intronic	FLT1	.	.	rs7328692	.	ID=S2009_raw:GT=hom:DP=28:FQ=1:SQ=-1;ID=S2503_raw:GT=hom:DP=19:FQ=1:SQ=-1;ID=S2507_raw:GT=hom:DP=18:FQ=1:SQ=-1;ID=S2668_raw:GT=hom:DP=24:FQ=1:SQ=-1;ID=S2012_raw:GT=hom:DP=37:FQ=1:SQ=-1;ID=S2010_raw:GT=hom:DP=23:FQ=1:SQ=-1;ID=S2662_raw:GT=hom:DP=10:FQ=1:SQ=-1;ID=S2663_raw:GT=hom:DP=19:FQ=1:SQ=-1;ID=S2011_raw:GT=hom:DP=41:FQ=1:SQ=-1;ID=S2504_raw:GT=hom:DP=19:FQ=1:SQ=-1;ID=S2008_raw:GT=hom:DP=33:FQ=1:SQ=-1;ID=S2509_raw:GT=hom:DP=14:FQ=1:SQ=-1;ID=S2508_raw:GT=hom:DP=18:FQ=1:SQ=-1;ID=S2666_raw:GT=hom:DP=20:FQ=1:SQ=-1;ID=S2669_raw:GT=hom:DP=14:FQ=1:SQ=-1;ID=S2166_raw:GT=hom:DP=26:FQ=1:SQ=-1;ID=S1775_raw:GT=hom:DP=19:FQ=1:SQ=-1;ID=S2501_raw:GT=hom:DP=19:FQ=1:SQ=-1;ID=S2500_raw:GT=hom:DP=16:FQ=1:SQ=-1;ID=S2502_raw:GT=hom:DP=17:FQ=0.94:SQ=-1;ID=S2505_raw:GT=hom:DP=26:FQ=1:SQ=-1;ID=S2176_raw:GT=hom:DP=19:FQ=1:SQ=-1;ID=S2506_raw:GT=hom:DP=20:FQ=1:SQ=-1;ID=S2667_raw:GT=hom:DP=24:FQ=1:SQ=-1;
2	2,16E+08	2,16E+08	C	T	exonic	FN1	nonsynonymous SNV	FN1:NM_001365523:exon39:c.6163G>A:p.V2055I,FN1:NM_212474:exon39:c.5878G>A:p.V1960I,FN1:NM_212476:exon39:c.6238G>A:p.V2080I,FN1:NM_001306130:exon40:c.6151G>A:p.V2051I,FN1:NM_001306131:exon40:c.6145G>A:p.V2049I,FN1:NM_001306132:exon40:c.6070G>A:p.V2024I,FN1:NM_001365517:exon40:c.6511G>A:p.V2171I,FN1:NM_001365518:exon40:c.6508G>A:p.V2170I,FN1:NM_001365519:exon40:c.6436G>A:p.V2146I,FN1:NM_001365520:exon40:c.6433G>A:p.V2145I,FN1:NM_001365524:exon40:c.6148G>A:p.V2050I,FN1:NM_001365521:exon41:c.6418G>A:p.V2140I,FN1:NM_001365522:exon41:c.6343G>A:p.V2115I,FN1:NM_002026:exon41:c.6415G>A:p.V2139I,FN1:NM_212478:exon41:c.6340G>A:p.V2114I,FN1:NM_212482:exon41:c.6781G>A:p.V2261I,FN1:NM_001306129:exon42:c.6688G>A:p.V2230I	rs1250209	.	ID=S2009_raw:GT=hom:DP=144:FQ=1:SQ=-1;ID=S2503_raw:GT=hom:DP=152:FQ=1:SQ=-1;ID=S2507_raw:GT=hom:DP=153:FQ=1:SQ=-1;ID=S2668_raw:GT=hom:DP=196:FQ=1:SQ=-1;ID=S2012_raw:GT=hom:DP=174:FQ=1:SQ=-1;ID=S2010_raw:GT=hom:DP=206:FQ=1:SQ=-1;ID=S2662_raw:GT=hom:DP=85:FQ=1:SQ=-1;ID=S2663_raw:GT=hom:DP=147:FQ=1:SQ=-1;ID=S2011_raw:GT=hom:DP=251:FQ=1:SQ=-1;ID=S2504_raw:GT=hom:DP=142:FQ=1:SQ=-1;ID=S2008_raw:GT=hom:DP=187:FQ=1:SQ=-1;ID=S2509_raw:GT=hom:DP=149:FQ=1:SQ=-1;ID=S2508_raw:GT=hom:DP=160:FQ=1:SQ=-1;ID=S2666_raw:GT=hom:DP=198:FQ=1:SQ=-1;ID=S2669_raw:GT=hom:DP=161:FQ=1:SQ=-1;ID=S2166_raw:GT=hom:DP=130:FQ=1:SQ=-1;ID=S1775_raw:GT=hom:DP=102:FQ=1:SQ=-1;ID=S2501_raw:GT=hom:DP=166:FQ=1:SQ=-1;ID=S2500_raw:GT=hom:DP=138:FQ=1:SQ=-1;ID=S2502_raw:GT=hom:DP=218:FQ=1:SQ=-1;ID=S2505_raw:GT=hom:DP=139:FQ=1:SQ=-1;ID=S2176_raw:GT=hom:DP=142:FQ=1:SQ=-1;ID=S2506_raw:GT=hom:DP=110:FQ=1:SQ=-1;ID=S2667_raw:GT=hom:DP=173:FQ=1:SQ=-1;
2	2,16E+08	2,16E+08	T	G	exonic	FN1	nonsynonymous SNV	FN1:NM_001306129:exon17:c.2449A>C:p.T817P,FN1:NM_001306130:exon17:c.2449A>C:p.T817P,FN1:NM_001306131:exon17:c.2449A>C:p.T817P,FN1:NM_001306132:exon17:c.2449A>C:p.T817P,FN1:NM_001365517:exon17:c.2449A>C:p.T817P,FN1:NM_001365518:exon17:c.2449A>C:p.T817P,FN1:NM_001365519:exon17:c.2449A>C:p.T817P,FN1:NM_001365520:exon17:c.2449A>C:p.T817P,FN1:NM_001365521:exon17:c.2449A>C:p.T817P,FN1:NM_001365522:exon17:c.2449A>C:p.T817P,FN1:NM_001365523:exon17:c.2449A>C:p.T817P,FN1:NM_001365524:exon17:c.2449A>C:p.T817P,FN1:NM_002026:exon17:c.2449A>C:p.T817P,FN1:NM_212474:exon17:c.2449A>C:p.T817P,FN1:NM_212476:exon17:c.2449A>C:p.T817P,FN1:NM_212478:exon17:c.2449A>C:p.T817P,FN1:NM_212482:exon17:c.2449A>C:p.T817P	rs2577301	.	ID=S2009_raw:GT=hom:DP=26:FQ=1:SQ=-1;ID=S2503_raw:GT=hom:DP=108:FQ=1:SQ=-1;ID=S2507_raw:GT=hom:DP=85:FQ=1:SQ=-1;ID=S2668_raw:GT=hom:DP=89:FQ=1:SQ=-1;ID=S2012_raw:GT=hom:DP=28:FQ=1:SQ=-1;ID=S2010_raw:GT=hom:DP=41:FQ=1:SQ=-1;ID=S2662_raw:GT=hom:DP=53:FQ=1:SQ=-1;ID=S2663_raw:GT=hom:DP=74:FQ=1:SQ=-1;ID=S2011_raw:GT=hom:DP=56:FQ=1:SQ=-1;ID=S2504_raw:GT=hom:DP=107:FQ=1:SQ=-1;ID=S2008_raw:GT=hom:DP=45:FQ=1:SQ=-1;ID=S2509_raw:GT=hom:DP=77:FQ=0.99:SQ=-1;ID=S2508_raw:GT=hom:DP=94:FQ=1:SQ=-1;ID=S2666_raw:GT=hom:DP=119:FQ=1:SQ=-1;ID=S2669_raw:GT=hom:DP=78:FQ=1:SQ=-1;ID=S2166_raw:GT=hom:DP=98:FQ=1:SQ=-1;ID=S1775_raw:GT=hom:DP=108:FQ=1:SQ=-1;ID=S2501_raw:GT=hom:DP=93:FQ=1:SQ=-1;ID=S2500_raw:GT=hom:DP=79:FQ=1:SQ=-1;ID=S2502_raw:GT=hom:DP=97:FQ=1:SQ=-1;ID=S2505_raw:GT=hom:DP=119:FQ=1:SQ=-1;ID=S2176_raw:GT=hom:DP=136:FQ=1:SQ=-1;ID=S2506_raw:GT=hom:DP=87:FQ=1:SQ=-1;ID=S2667_raw:GT=hom:DP=88:FQ=1:SQ=-1;
9	20866974	20866974	C	G	exonic	FOCAD	nonsynonymous SNV	FOCAD:NM_017794:exon20:c.2153C>G:p.T718S	rs7875872	.	ID=S2009_raw:GT=hom:DP=41:FQ=1:SQ=-1;ID=S2503_raw:GT=hom:DP=72:FQ=1:SQ=-1;ID=S2507_raw:GT=hom:DP=39:FQ=1:SQ=-1;ID=S2668_raw:GT=hom:DP=107:FQ=1:SQ=-1;ID=S2012_raw:GT=hom:DP=56:FQ=1:SQ=-1;ID=S2010_raw:GT=hom:DP=48:FQ=1:SQ=-1;ID=S2662_raw:GT=hom:DP=7:FQ=1:SQ=-1;ID=S2663_raw:GT=hom:DP=61:FQ=1:SQ=-1;ID=S2011_raw:GT=hom:DP=67:FQ=1:SQ=-1;ID=S2504_raw:GT=hom:DP=66:FQ=1:SQ=-1;ID=S2008_raw:GT=hom:DP=59:FQ=1:SQ=-1;ID=S2509_raw:GT=hom:DP=69:FQ=1:SQ=-1;ID=S2508_raw:GT=hom:DP=63:FQ=1:SQ=-1;ID=S2666_raw:GT=hom:DP=124:FQ=1:SQ=-1;ID=S2669_raw:GT=hom:DP=44:FQ=1:SQ=-1;ID=S2166_raw:GT=hom:DP=172:FQ=1:SQ=-1;ID=S1775_raw:GT=hom:DP=149:FQ=1:SQ=-1;ID=S2501_raw:GT=hom:DP=56:FQ=1:SQ=-1;ID=S2500_raw:GT=hom:DP=53:FQ=1:SQ=-1;ID=S2502_raw:GT=hom:DP=68:FQ=1:SQ=-1;ID=S2505_raw:GT=hom:DP=51:FQ=1:SQ=-1;ID=S2176_raw:GT=hom:DP=176:FQ=1:SQ=-1;ID=S2506_raw:GT=hom:DP=29:FQ=1:SQ=-1;ID=S2667_raw:GT=hom:DP=70:FQ=1:SQ=-1;
9	20978312	20978312	A	G	intronic	FOCAD	.	.	rs4977616	.	ID=S2009_raw:GT=hom:DP=10:FQ=1:SQ=-1;ID=S2503_raw:GT=hom:DP=38:FQ=1:SQ=-1;ID=S2507_raw:GT=hom:DP=33:FQ=1:SQ=-1;ID=S2668_raw:GT=hom:DP=65:FQ=1:SQ=-1;ID=S2012_raw:GT=hom:DP=11:FQ=1:SQ=-1;ID=S2010_raw:GT=hom:DP=23:FQ=1:SQ=-1;ID=S2662_raw:GT=hom:DP=1:FQ=1:SQ=-1;ID=S2663_raw:GT=hom:DP=38:FQ=1:SQ=-1;ID=S2011_raw:GT=hom:DP=29:FQ=1:SQ=-1;ID=S2504_raw:GT=hom:DP=49:FQ=1:SQ=-1;ID=S2008_raw:GT=hom:DP=20:FQ=1:SQ=-1;ID=S2509_raw:GT=hom:DP=42:FQ=1:SQ=-1;ID=S2508_raw:GT=hom:DP=33:FQ=1:SQ=-1;ID=S2666_raw:GT=hom:DP=53:FQ=1:SQ=-1;ID=S2669_raw:GT=hom:DP=38:FQ=1:SQ=-1;ID=S2166_raw:GT=hom:DP=61:FQ=1:SQ=-1;ID=S1775_raw:GT=hom:DP=57:FQ=1:SQ=-1;ID=S2501_raw:GT=hom:DP=54:FQ=1:SQ=-1;ID=S2500_raw:GT=hom:DP=52:FQ=1:SQ=-1;ID=S2502_raw:GT=hom:DP=40:FQ=1:SQ=-1;ID=S2505_raw:GT=hom:DP=31:FQ=1:SQ=-1;ID=S2176_raw:GT=hom:DP=72:FQ=1:SQ=-1;ID=S2506_raw:GT=hom:DP=40:FQ=1:SQ=-1;ID=S2667_raw:GT=hom:DP=41:FQ=1:SQ=-1;
9	20982503	20982503	T	G	intronic	FOCAD	.	.	rs4304410	.	ID=S2009_raw:GT=hom:DP=29:FQ=1:SQ=-1;ID=S2503_raw:GT=hom:DP=50:FQ=1:SQ=-1;ID=S2507_raw:GT=hom:DP=43:FQ=1:SQ=-1;ID=S2668_raw:GT=hom:DP=76:FQ=1:SQ=-1;ID=S2012_raw:GT=hom:DP=50:FQ=1:SQ=-1;ID=S2010_raw:GT=hom:DP=38:FQ=1:SQ=-1;ID=S2662_raw:GT=hom:DP=3:FQ=1:SQ=-1;ID=S2663_raw:GT=hom:DP=43:FQ=1:SQ=-1;ID=S2011_raw:GT=hom:DP=72:FQ=1:SQ=-1;ID=S2504_raw:GT=hom:DP=56:FQ=1:SQ=-1;ID=S2008_raw:GT=hom:DP=43:FQ=1:SQ=-1;ID=S2509_raw:GT=hom:DP=41:FQ=1:SQ=-1;ID=S2508_raw:GT=hom:DP=50:FQ=1:SQ=-1;ID=S2666_raw:GT=hom:DP=59:FQ=1:SQ=-1;ID=S2669_raw:GT=hom:DP=35:FQ=1:SQ=-1;ID=S2166_raw:GT=hom:DP=87:FQ=1:SQ=-1;ID=S1775_raw:GT=hom:DP=48:FQ=1:SQ=-1;ID=S2501_raw:GT=hom:DP=46:FQ=1:SQ=-1;ID=S2500_raw:GT=hom:DP=47:FQ=1:SQ=-1;ID=S2502_raw:GT=hom:DP=51:FQ=1:SQ=-1;ID=S2505_raw:GT=hom:DP=51:FQ=1:SQ=-1;ID=S2176_raw:GT=hom:DP=88:FQ=1:SQ=-1;ID=S2506_raw:GT=hom:DP=38:FQ=1:SQ=-1;ID=S2667_raw:GT=hom:DP=44:FQ=1:SQ=-1;
8	1,46E+08	1,46E+08	T	C	UTR3	FOXH1	.	NM_003923:c.*20A>G	rs2721176	Benign	ID=S2009_raw:GT=hom:DP=25:FQ=1:SQ=-1;ID=S2503_raw:GT=hom:DP=81:FQ=1:SQ=-1;ID=S2507_raw:GT=hom:DP=111:FQ=1:SQ=-1;ID=S2668_raw:GT=hom:DP=72:FQ=1:SQ=-1;ID=S2012_raw:GT=hom:DP=32:FQ=1:SQ=-1;ID=S2010_raw:GT=hom:DP=36:FQ=1:SQ=-1;ID=S2662_raw:GT=hom:DP=185:FQ=1:SQ=-1;ID=S2663_raw:GT=hom:DP=78:FQ=1:SQ=-1;ID=S2011_raw:GT=hom:DP=38:FQ=1:SQ=-1;ID=S2504_raw:GT=hom:DP=109:FQ=1:SQ=-1;ID=S2008_raw:GT=hom:DP=27:FQ=1:SQ=-1;ID=S2509_raw:GT=hom:DP=73:FQ=1:SQ=-1;ID=S2508_raw:GT=hom:DP=101:FQ=1:SQ=-1;ID=S2666_raw:GT=hom:DP=44:FQ=1:SQ=-1;ID=S2669_raw:GT=hom:DP=124:FQ=1:SQ=-1;ID=S2166_raw:GT=hom:DP=55:FQ=1:SQ=-1;ID=S1775_raw:GT=hom:DP=45:FQ=1:SQ=-1;ID=S2501_raw:GT=hom:DP=95:FQ=1:SQ=-1;ID=S2500_raw:GT=hom:DP=85:FQ=1:SQ=-1;ID=S2502_raw:GT=hom:DP=102:FQ=1:SQ=-1;ID=S2505_raw:GT=hom:DP=65:FQ=1:SQ=-1;ID=S2176_raw:GT=hom:DP=67:FQ=1:SQ=-1;ID=S2506_raw:GT=hom:DP=105:FQ=1:SQ=-1;ID=S2667_raw:GT=hom:DP=103:FQ=0.99:SQ=-1;
11	1,26E+08	1,26E+08	C	T	intronic	FOXRED1	.	.	rs56321210	.	ID=S2009_raw:GT=hom:DP=13:FQ=1:SQ=-1;ID=S2503_raw:GT=hom:DP=32:FQ=1:SQ=-1;ID=S2507_raw:GT=hom:DP=32:FQ=1:SQ=-1;ID=S2668_raw:GT=hom:DP=54:FQ=1:SQ=-1;ID=S2012_raw:GT=hom:DP=11:FQ=1:SQ=-1;ID=S2010_raw:GT=hom:DP=18:FQ=1:SQ=-1;ID=S2662_raw:GT=hom:DP=67:FQ=1:SQ=-1;ID=S2663_raw:GT=hom:DP=42:FQ=1:SQ=-1;ID=S2011_raw:GT=hom:DP=17:FQ=1:SQ=-1;ID=S2504_raw:GT=hom:DP=35:FQ=1:SQ=-1;ID=S2008_raw:GT=hom:DP=16:FQ=1:SQ=-1;ID=S2509_raw:GT=hom:DP=35:FQ=1:SQ=-1;ID=S2508_raw:GT=hom:DP=44:FQ=1:SQ=-1;ID=S2666_raw:GT=hom:DP=32:FQ=1:SQ=-1;ID=S2669_raw:GT=hom:DP=44:FQ=1:SQ=-1;ID=S2166_raw:GT=hom:DP=27:FQ=1:SQ=-1;ID=S1775_raw:GT=hom:DP=22:FQ=1:SQ=-1;ID=S2501_raw:GT=hom:DP=37:FQ=1:SQ=-1;ID=S2500_raw:GT=hom:DP=24:FQ=1:SQ=-1;ID=S2502_raw:GT=hom:DP=48:FQ=1:SQ=-1;ID=S2505_raw:GT=hom:DP=30:FQ=1:SQ=-1;ID=S2176_raw:GT=hom:DP=38:FQ=1:SQ=-1;ID=S2506_raw:GT=hom:DP=39:FQ=1:SQ=-1;ID=S2667_raw:GT=hom:DP=34:FQ=1:SQ=-1;
4	79176325	79176325	C	T	intronic	FRAS1	.	.	rs1017647	.	ID=S2009_raw:GT=hom:DP=2:FQ=1:SQ=-1;ID=S2503_raw:GT=hom:DP=109:FQ=1:SQ=-1;ID=S2507_raw:GT=hom:DP=94:FQ=1:SQ=-1;ID=S2668_raw:GT=hom:DP=132:FQ=1:SQ=-1;ID=S2012_raw:GT=hom:DP=5:FQ=1:SQ=-1;ID=S2010_raw:GT=hom:DP=3:FQ=1:SQ=-1;ID=S2662_raw:GT=hom:DP=71:FQ=1:SQ=-1;ID=S2663_raw:GT=hom:DP=108:FQ=1:SQ=-1;ID=S2011_raw:GT=hom:DP=6:FQ=1:SQ=-1;ID=S2504_raw:GT=hom:DP=112:FQ=1:SQ=-1;ID=S2008_raw:GT=hom:DP=7:FQ=1:SQ=-1;ID=S2509_raw:GT=hom:DP=94:FQ=1:SQ=-1;ID=S2508_raw:GT=hom:DP=130:FQ=1:SQ=-1;ID=S2666_raw:GT=hom:DP=119:FQ=1:SQ=-1;ID=S2669_raw:GT=hom:DP=96:FQ=1:SQ=-1;ID=S2166_raw:GT=hom:DP=25:FQ=1:SQ=-1;ID=S1775_raw:GT=hom:DP=16:FQ=1:SQ=-1;ID=S2501_raw:GT=hom:DP=96:FQ=1:SQ=-1;ID=S2500_raw:GT=hom:DP=111:FQ=1:SQ=-1;ID=S2502_raw:GT=hom:DP=115:FQ=1:SQ=-1;ID=S2505_raw:GT=hom:DP=104:FQ=1:SQ=-1;ID=S2176_raw:GT=hom:DP=30:FQ=1:SQ=-1;ID=S2506_raw:GT=hom:DP=79:FQ=1:SQ=-1;ID=S2667_raw:GT=hom:DP=87:FQ=1:SQ=-1;
4	79176361	79176361	T	C	intronic	FRAS1	.	.	rs1017646	.	ID=S2009_raw:GT=hom:DP=4:FQ=1:SQ=-1;ID=S2503_raw:GT=hom:DP=136:FQ=1:SQ=-1;ID=S2507_raw:GT=hom:DP=121:FQ=1:SQ=-1;ID=S2668_raw:GT=hom:DP=174:FQ=1:SQ=-1;ID=S2012_raw:GT=hom:DP=14:FQ=1:SQ=-1;ID=S2010_raw:GT=hom:DP=5:FQ=1:SQ=-1;ID=S2662_raw:GT=hom:DP=92:FQ=0.98:SQ=-1;ID=S2663_raw:GT=hom:DP=132:FQ=1:SQ=-1;ID=S2011_raw:GT=hom:DP=12:FQ=1:SQ=-1;ID=S2504_raw:GT=hom:DP=151:FQ=1:SQ=-1;ID=S2008_raw:GT=hom:DP=12:FQ=1:SQ=-1;ID=S2509_raw:GT=hom:DP=120:FQ=1:SQ=-1;ID=S2508_raw:GT=hom:DP=175:FQ=1:SQ=-1;ID=S2666_raw:GT=hom:DP=131:FQ=1:SQ=-1;ID=S2669_raw:GT=hom:DP=148:FQ=1:SQ=-1;ID=S2166_raw:GT=hom:DP=44:FQ=1:SQ=-1;ID=S1775_raw:GT=hom:DP=25:FQ=1:SQ=-1;ID=S2501_raw:GT=hom:DP=135:FQ=1:SQ=-1;ID=S2500_raw:GT=hom:DP=141:FQ=1:SQ=-1;ID=S2502_raw:GT=hom:DP=145:FQ=1:SQ=-1;ID=S2505_raw:GT=hom:DP=144:FQ=1:SQ=-1;ID=S2176_raw:GT=hom:DP=51:FQ=1:SQ=-1;ID=S2506_raw:GT=hom:DP=110:FQ=1:SQ=-1;ID=S2667_raw:GT=hom:DP=115:FQ=1:SQ=-1;
4	79186171	79186171	G	A	splicing	FRAS1	.	NM_025074:exon7:c.604-8G>A;NM_001166133:exon7:c.604-8G>A	rs2867014	Benign	ID=S2009_raw:GT=hom:DP=50:FQ=1:SQ=-1;ID=S2503_raw:GT=hom:DP=103:FQ=1:SQ=-1;ID=S2507_raw:GT=hom:DP=92:FQ=1:SQ=-1;ID=S2668_raw:GT=hom:DP=171:FQ=0.99:SQ=-1;ID=S2012_raw:GT=hom:DP=32:FQ=1:SQ=-1;ID=S2010_raw:GT=hom:DP=56:FQ=1:SQ=-1;ID=S2662_raw:GT=hom:DP=31:FQ=1:SQ=-1;ID=S2663_raw:GT=hom:DP=126:FQ=1:SQ=-1;ID=S2011_raw:GT=hom:DP=68:FQ=1:SQ=-1;ID=S2504_raw:GT=hom:DP=141:FQ=1:SQ=-1;ID=S2008_raw:GT=hom:DP=46:FQ=1:SQ=-1;ID=S2509_raw:GT=hom:DP=124:FQ=1:SQ=-1;ID=S2508_raw:GT=hom:DP=133:FQ=1:SQ=-1;ID=S2666_raw:GT=hom:DP=171:FQ=1:SQ=-1;ID=S2669_raw:GT=hom:DP=90:FQ=1:SQ=-1;ID=S2166_raw:GT=hom:DP=172:FQ=1:SQ=-1;ID=S1775_raw:GT=hom:DP=154:FQ=1:SQ=-1;ID=S2501_raw:GT=hom:DP=132:FQ=1:SQ=-1;ID=S2500_raw:GT=hom:DP=119:FQ=1:SQ=-1;ID=S2502_raw:GT=hom:DP=145:FQ=1:SQ=-1;ID=S2505_raw:GT=hom:DP=138:FQ=1:SQ=-1;ID=S2176_raw:GT=hom:DP=186:FQ=1:SQ=-1;ID=S2506_raw:GT=hom:DP=77:FQ=1:SQ=-1;ID=S2667_raw:GT=hom:DP=121:FQ=1:SQ=-1;
4	79187724	79187724	G	A	intronic	FRAS1	.	.	rs6820900	.	ID=S2009_raw:GT=hom:DP=2:FQ=1:SQ=-1;ID=S2503_raw:GT=hom:DP=1:FQ=1:SQ=-1;ID=S2507_raw:GT=hom:DP=3:FQ=1:SQ=-1;ID=S2668_raw:GT=hom:DP=6:FQ=1:SQ=-1;ID=S2012_raw:GT=hom:DP=2:FQ=1:SQ=-1;ID=S2010_raw:GT=hom:DP=2:FQ=1:SQ=-1;ID=S2662_raw:GT=hom:DP=3:FQ=1:SQ=-1;ID=S2663_raw:GT=hom:DP=7:FQ=1:SQ=-1;ID=S2011_raw:GT=hom:DP=2:FQ=1:SQ=-1;ID=S2504_raw:GT=hom:DP=6:FQ=1:SQ=-1;ID=S2008_raw:GT=hom:DP=2:FQ=1:SQ=-1;ID=S2509_raw:GT=hom:DP=2:FQ=1:SQ=-1;ID=S2508_raw:GT=hom:DP=8:FQ=1:SQ=-1;ID=S2666_raw:GT=hom:DP=4:FQ=1:SQ=-1;ID=S2669_raw:GT=hom:DP=7:FQ=1:SQ=-1;ID=S2166_raw:GT=hom:DP=5:FQ=1:SQ=-1;ID=S1775_raw:GT=hom:DP=6:FQ=1:SQ=-1;ID=S2501_raw:GT=hom:DP=3:FQ=1:SQ=-1;ID=S2500_raw:GT=hom:DP=4:FQ=1:SQ=-1;ID=S2502_raw:GT=hom:DP=5:FQ=1:SQ=-1;ID=S2505_raw:GT=hom:DP=4:FQ=1:SQ=-1;ID=S2176_raw:GT=hom:DP=1:FQ=1:SQ=-1;ID=S2506_raw:GT=hom:DP=3:FQ=1:SQ=-1;ID=S2667_raw:GT=hom:DP=3:FQ=1:SQ=-1;
4	79387464	79387464	A	G	exonic	FRAS1	nonsynonymous SNV	FRAS1:NM_025074:exon50:c.7132A>G:p.K2378E	rs7684722	Benign	ID=S2009_raw:GT=hom:DP=75:FQ=1:SQ=-1;ID=S2503_raw:GT=hom:DP=149:FQ=1:SQ=-1;ID=S2507_raw:GT=hom:DP=140:FQ=1:SQ=-1;ID=S2668_raw:GT=hom:DP=184:FQ=1:SQ=-1;ID=S2012_raw:GT=hom:DP=121:FQ=1:SQ=-1;ID=S2010_raw:GT=hom:DP=117:FQ=1:SQ=-1;ID=S2662_raw:GT=hom:DP=71:FQ=1:SQ=-1;ID=S2663_raw:GT=hom:DP=161:FQ=1:SQ=-1;ID=S2011_raw:GT=hom:DP=162:FQ=1:SQ=-1;ID=S2504_raw:GT=hom:DP=200:FQ=1:SQ=-1;ID=S2008_raw:GT=hom:DP=92:FQ=1:SQ=-1;ID=S2509_raw:GT=hom:DP=164:FQ=1:SQ=-1;ID=S2508_raw:GT=hom:DP=218:FQ=1:SQ=-1;ID=S2666_raw:GT=hom:DP=161:FQ=1:SQ=-1;ID=S2669_raw:GT=hom:DP=116:FQ=1:SQ=-1;ID=S2166_raw:GT=hom:DP=297:FQ=1:SQ=-1;ID=S1775_raw:GT=hom:DP=300:FQ=1:SQ=-1;ID=S2501_raw:GT=hom:DP=185:FQ=1:SQ=-1;ID=S2500_raw:GT=hom:DP=166:FQ=1:SQ=-1;ID=S2502_raw:GT=hom:DP=199:FQ=1:SQ=-1;ID=S2505_raw:GT=hom:DP=149:FQ=1:SQ=-1;ID=S2176_raw:GT=hom:DP=340:FQ=1:SQ=-1;ID=S2506_raw:GT=hom:DP=142:FQ=1:SQ=-1;ID=S2667_raw:GT=hom:DP=156:FQ=1:SQ=-1;
4	79391020	79391020	C	T	intronic	FRAS1	.	.	rs7681619	.	ID=S2009_raw:GT=hom:DP=51:FQ=1:SQ=-1;ID=S2503_raw:GT=hom:DP=32:FQ=1:SQ=-1;ID=S2507_raw:GT=hom:DP=35:FQ=1:SQ=-1;ID=S2668_raw:GT=hom:DP=24:FQ=1:SQ=-1;ID=S2012_raw:GT=hom:DP=88:FQ=1:SQ=-1;ID=S2010_raw:GT=hom:DP=87:FQ=1:SQ=-1;ID=S2662_raw:GT=hom:DP=6:FQ=1:SQ=-1;ID=S2663_raw:GT=hom:DP=22:FQ=1:SQ=-1;ID=S2011_raw:GT=hom:DP=124:FQ=1:SQ=-1;ID=S2504_raw:GT=hom:DP=46:FQ=1:SQ=-1;ID=S2008_raw:GT=hom:DP=95:FQ=1:SQ=-1;ID=S2509_raw:GT=hom:DP=25:FQ=1:SQ=-1;ID=S2508_raw:GT=hom:DP=19:FQ=1:SQ=-1;ID=S2666_raw:GT=hom:DP=24:FQ=1:SQ=-1;ID=S2669_raw:GT=hom:DP=15:FQ=1:SQ=-1;ID=S2166_raw:GT=hom:DP=40:FQ=1:SQ=-1;ID=S1775_raw:GT=hom:DP=30:FQ=1:SQ=-1;ID=S2501_raw:GT=hom:DP=22:FQ=1:SQ=-1;ID=S2500_raw:GT=hom:DP=23:FQ=1:SQ=-1;ID=S2502_raw:GT=hom:DP=36:FQ=1:SQ=-1;ID=S2505_raw:GT=hom:DP=30:FQ=1:SQ=-1;ID=S2176_raw:GT=hom:DP=23:FQ=1:SQ=-1;ID=S2506_raw:GT=hom:DP=22:FQ=1:SQ=-1;ID=S2667_raw:GT=hom:DP=29:FQ=1:SQ=-1;
4	79391036	79391036	T	C	intronic	FRAS1	.	.	rs1496602	.	ID=S2009_raw:GT=hom:DP=75:FQ=1:SQ=-1;ID=S2503_raw:GT=hom:DP=45:FQ=1:SQ=-1;ID=S2507_raw:GT=hom:DP=48:FQ=1:SQ=-1;ID=S2668_raw:GT=hom:DP=51:FQ=1:SQ=-1;ID=S2012_raw:GT=hom:DP=99:FQ=1:SQ=-1;ID=S2010_raw:GT=hom:DP=102:FQ=1:SQ=-1;ID=S2662_raw:GT=hom:DP=9:FQ=1:SQ=-1;ID=S2663_raw:GT=hom:DP=36:FQ=1:SQ=-1;ID=S2011_raw:GT=hom:DP=157:FQ=1:SQ=-1;ID=S2504_raw:GT=hom:DP=59:FQ=1:SQ=-1;ID=S2008_raw:GT=hom:DP=117:FQ=1:SQ=-1;ID=S2509_raw:GT=hom:DP=41:FQ=1:SQ=-1;ID=S2508_raw:GT=hom:DP=32:FQ=1:SQ=-1;ID=S2666_raw:GT=hom:DP=39:FQ=1:SQ=-1;ID=S2669_raw:GT=hom:DP=22:FQ=1:SQ=-1;ID=S2166_raw:GT=hom:DP=62:FQ=1:SQ=-1;ID=S1775_raw:GT=hom:DP=42:FQ=1:SQ=-1;ID=S2501_raw:GT=hom:DP=31:FQ=1:SQ=-1;ID=S2500_raw:GT=hom:DP=32:FQ=1:SQ=-1;ID=S2502_raw:GT=hom:DP=62:FQ=1:SQ=-1;ID=S2505_raw:GT=hom:DP=41:FQ=1:SQ=-1;ID=S2176_raw:GT=hom:DP=38:FQ=1:SQ=-1;ID=S2506_raw:GT=hom:DP=26:FQ=1:SQ=-1;ID=S2667_raw:GT=hom:DP=41:FQ=1:SQ=-1;
4	79391256	79391256	T	C	intronic	FRAS1	.	.	rs7664505	Benign	ID=S2009_raw:GT=hom:DP=89:FQ=1:SQ=-1;ID=S2503_raw:GT=hom:DP=148:FQ=1:SQ=-1;ID=S2507_raw:GT=hom:DP=151:FQ=1:SQ=-1;ID=S2668_raw:GT=hom:DP=248:FQ=1:SQ=-1;ID=S2012_raw:GT=hom:DP=96:FQ=1:SQ=-1;ID=S2010_raw:GT=hom:DP=102:FQ=1:SQ=-1;ID=S2662_raw:GT=hom:DP=65:FQ=1:SQ=-1;ID=S2663_raw:GT=hom:DP=166:FQ=1:SQ=-1;ID=S2011_raw:GT=hom:DP=171:FQ=1:SQ=-1;ID=S2504_raw:GT=hom:DP=189:FQ=1:SQ=-1;ID=S2008_raw:GT=hom:DP=99:FQ=1:SQ=-1;ID=S2509_raw:GT=hom:DP=144:FQ=1:SQ=-1;ID=S2508_raw:GT=hom:DP=178:FQ=1:SQ=-1;ID=S2666_raw:GT=hom:DP=232:FQ=1:SQ=-1;ID=S2669_raw:GT=hom:DP=152:FQ=1:SQ=-1;ID=S2166_raw:GT=hom:DP=98:FQ=1:SQ=-1;ID=S1775_raw:GT=hom:DP=80:FQ=1:SQ=-1;ID=S2501_raw:GT=hom:DP=134:FQ=1:SQ=-1;ID=S2500_raw:GT=hom:DP=158:FQ=1:SQ=-1;ID=S2502_raw:GT=hom:DP=189:FQ=1:SQ=-1;ID=S2505_raw:GT=hom:DP=149:FQ=1:SQ=-1;ID=S2176_raw:GT=hom:DP=108:FQ=1:SQ=-1;ID=S2506_raw:GT=hom:DP=122:FQ=1:SQ=-1;ID=S2667_raw:GT=hom:DP=157:FQ=1:SQ=-1;
4	79455858	79455858	T	A	intronic	FRAS1	.	.	rs4975140	.	ID=S2009_raw:GT=hom:DP=8:FQ=1:SQ=-1;ID=S2503_raw:GT=hom:DP=24:FQ=1:SQ=-1;ID=S2507_raw:GT=hom:DP=17:FQ=1:SQ=-1;ID=S2668_raw:GT=hom:DP=29:FQ=1:SQ=-1;ID=S2012_raw:GT=hom:DP=5:FQ=1:SQ=-1;ID=S2010_raw:GT=hom:DP=11:FQ=1:SQ=-1;ID=S2662_raw:GT=hom:DP=8:FQ=1:SQ=-1;ID=S2663_raw:GT=hom:DP=25:FQ=1:SQ=-1;ID=S2011_raw:GT=hom:DP=11:FQ=1:SQ=-1;ID=S2504_raw:GT=hom:DP=10:FQ=1:SQ=-1;ID=S2008_raw:GT=hom:DP=4:FQ=1:SQ=-1;ID=S2509_raw:GT=hom:DP=27:FQ=1:SQ=-1;ID=S2508_raw:GT=hom:DP=26:FQ=1:SQ=-1;ID=S2666_raw:GT=hom:DP=26:FQ=1:SQ=-1;ID=S2669_raw:GT=hom:DP=17:FQ=1:SQ=-1;ID=S2166_raw:GT=hom:DP=25:FQ=1:SQ=-1;ID=S1775_raw:GT=hom:DP=12:FQ=1:SQ=-1;ID=S2501_raw:GT=hom:DP=21:FQ=1:SQ=-1;ID=S2500_raw:GT=hom:DP=22:FQ=1:SQ=-1;ID=S2502_raw:GT=hom:DP=25:FQ=1:SQ=-1;ID=S2505_raw:GT=hom:DP=18:FQ=1:SQ=-1;ID=S2176_raw:GT=hom:DP=18:FQ=1:SQ=-1;ID=S2506_raw:GT=hom:DP=18:FQ=1:SQ=-1;ID=S2667_raw:GT=hom:DP=28:FQ=1:SQ=-1;
9	14759725	14759725	A	G	intronic	FREM1	.	.	rs7045154	.	ID=S2009_raw:GT=hom:DP=47:FQ=1:SQ=-1;ID=S2503_raw:GT=hom:DP=52:FQ=1:SQ=-1;ID=S2507_raw:GT=hom:DP=44:FQ=1:SQ=-1;ID=S2668_raw:GT=hom:DP=44:FQ=1:SQ=-1;ID=S2012_raw:GT=hom:DP=46:FQ=1:SQ=-1;ID=S2010_raw:GT=hom:DP=62:FQ=1:SQ=-1;ID=S2662_raw:GT=hom:DP=2:FQ=1:SQ=-1;ID=S2663_raw:GT=hom:DP=23:FQ=1:SQ=-1;ID=S2011_raw:GT=hom:DP=79:FQ=1:SQ=-1;ID=S2504_raw:GT=hom:DP=31:FQ=1:SQ=-1;ID=S2008_raw:GT=hom:DP=48:FQ=1:SQ=-1;ID=S2509_raw:GT=hom:DP=51:FQ=0.98:SQ=-1;ID=S2508_raw:GT=hom:DP=24:FQ=1:SQ=-1;ID=S2666_raw:GT=hom:DP=34:FQ=1:SQ=-1;ID=S2669_raw:GT=hom:DP=32:FQ=1:SQ=-1;ID=S2166_raw:GT=hom:DP=117:FQ=1:SQ=-1;ID=S1775_raw:GT=hom:DP=91:FQ=1:SQ=-1;ID=S2501_raw:GT=hom:DP=45:FQ=1:SQ=-1;ID=S2500_raw:GT=hom:DP=46:FQ=1:SQ=-1;ID=S2502_raw:GT=hom:DP=26:FQ=1:SQ=-1;ID=S2505_raw:GT=hom:DP=34:FQ=1:SQ=-1;ID=S2176_raw:GT=hom:DP=131:FQ=1:SQ=-1;ID=S2506_raw:GT=hom:DP=30:FQ=1:SQ=-1;ID=S2667_raw:GT=hom:DP=31:FQ=1:SQ=-1;
9	14774329	14774329	T	C	intronic	FREM1	.	.	rs7018782	.	ID=S2009_raw:GT=hom:DP=5:FQ=1:SQ=-1;ID=S2503_raw:GT=hom:DP=17:FQ=1:SQ=-1;ID=S2507_raw:GT=hom:DP=12:FQ=1:SQ=-1;ID=S2668_raw:GT=hom:DP=9:FQ=1:SQ=-1;ID=S2012_raw:GT=hom:DP=6:FQ=1:SQ=-1;ID=S2010_raw:GT=hom:DP=17:FQ=1:SQ=-1;ID=S2662_raw:GT=hom:DP=1:FQ=1:SQ=-1;ID=S2663_raw:GT=hom:DP=7:FQ=1:SQ=-1;ID=S2011_raw:GT=hom:DP=28:FQ=1:SQ=-1;ID=S2504_raw:GT=hom:DP=19:FQ=1:SQ=-1;ID=S2008_raw:GT=hom:DP=15:FQ=1:SQ=-1;ID=S2509_raw:GT=hom:DP=12:FQ=1:SQ=-1;ID=S2508_raw:GT=hom:DP=10:FQ=1:SQ=-1;ID=S2666_raw:GT=hom:DP=7:FQ=1:SQ=-1;ID=S2669_raw:GT=hom:DP=13:FQ=1:SQ=-1;ID=S2166_raw:GT=hom:DP=27:FQ=1:SQ=-1;ID=S1775_raw:GT=hom:DP=15:FQ=1:SQ=-1;ID=S2501_raw:GT=hom:DP=13:FQ=1:SQ=-1;ID=S2500_raw:GT=hom:DP=17:FQ=1:SQ=-1;ID=S2502_raw:GT=hom:DP=15:FQ=1:SQ=-1;ID=S2505_raw:GT=hom:DP=21:FQ=1:SQ=-1;ID=S2176_raw:GT=hom:DP=35:FQ=1:SQ=-1;ID=S2506_raw:GT=hom:DP=23:FQ=1:SQ=-1;ID=S2667_raw:GT=hom:DP=14:FQ=1:SQ=-1;
13	39262057	39262057	G	A	exonic	FREM2	synonymous SNV	FREM2:NM_207361:exon1:c.576G>A:p.E192E	rs1868464	Benign	ID=S2009_raw:GT=hom:DP=122:FQ=1:SQ=-1;ID=S2503_raw:GT=hom:DP=215:FQ=1:SQ=-1;ID=S2507_raw:GT=hom:DP=220:FQ=1:SQ=-1;ID=S2668_raw:GT=hom:DP=290:FQ=1:SQ=-1;ID=S2012_raw:GT=hom:DP=89:FQ=1:SQ=-1;ID=S2010_raw:GT=hom:DP=132:FQ=1:SQ=-1;ID=S2662_raw:GT=hom:DP=399:FQ=1:SQ=-1;ID=S2663_raw:GT=hom:DP=290:FQ=1:SQ=-1;ID=S2011_raw:GT=hom:DP=170:FQ=1:SQ=-1;ID=S2504_raw:GT=hom:DP=246:FQ=1:SQ=-1;ID=S2008_raw:GT=hom:DP=141:FQ=1:SQ=-1;ID=S2509_raw:GT=hom:DP=241:FQ=1:SQ=-1;ID=S2508_raw:GT=hom:DP=292:FQ=1:SQ=-1;ID=S2666_raw:GT=hom:DP=233:FQ=1:SQ=-1;ID=S2669_raw:GT=hom:DP=264:FQ=1:SQ=-1;ID=S2166_raw:GT=hom:DP=201:FQ=1:SQ=-1;ID=S1775_raw:GT=hom:DP=87:FQ=1:SQ=-1;ID=S2501_raw:GT=hom:DP=212:FQ=1:SQ=-1;ID=S2500_raw:GT=hom:DP=180:FQ=1:SQ=-1;ID=S2502_raw:GT=hom:DP=230:FQ=1:SQ=-1;ID=S2505_raw:GT=hom:DP=212:FQ=1:SQ=-1;ID=S2176_raw:GT=hom:DP=172:FQ=1:SQ=-1;ID=S2506_raw:GT=hom:DP=207:FQ=1:SQ=-1;ID=S2667_raw:GT=hom:DP=288:FQ=1:SQ=-1;
13	39263714	39263714	T	C	exonic	FREM2	nonsynonymous SNV	FREM2:NM_207361:exon1:c.2233T>C:p.S745P	rs2496423	Benign	ID=S2009_raw:GT=hom:DP=151:FQ=1:SQ=-1;ID=S2503_raw:GT=hom:DP=458:FQ=1:SQ=-1;ID=S2507_raw:GT=hom:DP=342:FQ=1:SQ=-1;ID=S2668_raw:GT=hom:DP=471:FQ=1:SQ=-1;ID=S2012_raw:GT=hom:DP=155:FQ=1:SQ=-1;ID=S2010_raw:GT=hom:DP=160:FQ=1:SQ=-1;ID=S2662_raw:GT=hom:DP=529:FQ=1:SQ=-1;ID=S2663_raw:GT=hom:DP=391:FQ=1:SQ=-1;ID=S2011_raw:GT=hom:DP=225:FQ=1:SQ=-1;ID=S2504_raw:GT=hom:DP=555:FQ=1:SQ=-1;ID=S2008_raw:GT=hom:DP=212:FQ=1:SQ=-1;ID=S2509_raw:GT=hom:DP=452:FQ=1:SQ=-1;ID=S2508_raw:GT=hom:DP=503:FQ=1:SQ=-1;ID=S2666_raw:GT=hom:DP=438:FQ=1:SQ=-1;ID=S2669_raw:GT=hom:DP=364:FQ=1:SQ=-1;ID=S2166_raw:GT=hom:DP=501:FQ=1:SQ=-1;ID=S1775_raw:GT=hom:DP=337:FQ=1:SQ=-1;ID=S2501_raw:GT=hom:DP=432:FQ=1:SQ=-1;ID=S2500_raw:GT=hom:DP=441:FQ=1:SQ=-1;ID=S2502_raw:GT=hom:DP=425:FQ=1:SQ=-1;ID=S2505_raw:GT=hom:DP=463:FQ=1:SQ=-1;ID=S2176_raw:GT=hom:DP=453:FQ=1:SQ=-1;ID=S2506_raw:GT=hom:DP=357:FQ=1:SQ=-1;ID=S2667_raw:GT=hom:DP=383:FQ=1:SQ=-1;
21	34915324	34915324	C	G	upstream	GART;SON	.	dist=20	rs1041745	.	ID=S2009_raw:GT=hom:DP=43:FQ=1:SQ=-1;ID=S2503_raw:GT=hom:DP=70:FQ=1:SQ=-1;ID=S2507_raw:GT=hom:DP=59:FQ=1:SQ=-1;ID=S2668_raw:GT=hom:DP=57:FQ=1:SQ=-1;ID=S2012_raw:GT=hom:DP=58:FQ=1:SQ=-1;ID=S2010_raw:GT=hom:DP=75:FQ=1:SQ=-1;ID=S2662_raw:GT=hom:DP=114:FQ=1:SQ=-1;ID=S2663_raw:GT=hom:DP=51:FQ=1:SQ=-1;ID=S2011_raw:GT=hom:DP=84:FQ=1:SQ=-1;ID=S2504_raw:GT=hom:DP=87:FQ=1:SQ=-1;ID=S2008_raw:GT=hom:DP=69:FQ=1:SQ=-1;ID=S2509_raw:GT=hom:DP=54:FQ=1:SQ=-1;ID=S2508_raw:GT=hom:DP=72:FQ=1:SQ=-1;ID=S2666_raw:GT=hom:DP=53:FQ=1:SQ=-1;ID=S2669_raw:GT=hom:DP=54:FQ=1:SQ=-1;ID=S2166_raw:GT=hom:DP=45:FQ=1:SQ=-1;ID=S1775_raw:GT=hom:DP=34:FQ=1:SQ=-1;ID=S2501_raw:GT=hom:DP=76:FQ=1:SQ=-1;ID=S2500_raw:GT=hom:DP=56:FQ=1:SQ=-1;ID=S2502_raw:GT=hom:DP=74:FQ=1:SQ=-1;ID=S2505_raw:GT=hom:DP=63:FQ=1:SQ=-1;ID=S2176_raw:GT=hom:DP=58:FQ=1:SQ=-1;ID=S2506_raw:GT=hom:DP=40:FQ=1:SQ=-1;ID=S2667_raw:GT=hom:DP=60:FQ=1:SQ=-1;
21	34915325	34915325	G	C	upstream	GART;SON	.	dist=19	rs1041746	.	ID=S2009_raw:GT=hom:DP=43:FQ=1:SQ=-1;ID=S2503_raw:GT=hom:DP=70:FQ=0.99:SQ=-1;ID=S2507_raw:GT=hom:DP=59:FQ=1:SQ=-1;ID=S2668_raw:GT=hom:DP=57:FQ=1:SQ=-1;ID=S2012_raw:GT=hom:DP=57:FQ=1:SQ=-1;ID=S2010_raw:GT=hom:DP=75:FQ=1:SQ=-1;ID=S2662_raw:GT=hom:DP=114:FQ=1:SQ=-1;ID=S2663_raw:GT=hom:DP=52:FQ=1:SQ=-1;ID=S2011_raw:GT=hom:DP=84:FQ=1:SQ=-1;ID=S2504_raw:GT=hom:DP=87:FQ=1:SQ=-1;ID=S2008_raw:GT=hom:DP=69:FQ=1:SQ=-1;ID=S2509_raw:GT=hom:DP=52:FQ=1:SQ=-1;ID=S2508_raw:GT=hom:DP=72:FQ=1:SQ=-1;ID=S2666_raw:GT=hom:DP=53:FQ=1:SQ=-1;ID=S2669_raw:GT=hom:DP=54:FQ=1:SQ=-1;ID=S2166_raw:GT=hom:DP=45:FQ=1:SQ=-1;ID=S1775_raw:GT=hom:DP=34:FQ=1:SQ=-1;ID=S2501_raw:GT=hom:DP=75:FQ=1:SQ=-1;ID=S2500_raw:GT=hom:DP=56:FQ=1:SQ=-1;ID=S2502_raw:GT=hom:DP=73:FQ=1:SQ=-1;ID=S2505_raw:GT=hom:DP=63:FQ=1:SQ=-1;ID=S2176_raw:GT=hom:DP=58:FQ=1:SQ=-1;ID=S2506_raw:GT=hom:DP=40:FQ=1:SQ=-1;ID=S2667_raw:GT=hom:DP=59:FQ=1:SQ=-1;
X	48649428	48649428	-	G	intronic	GATA1	.	.	.	.	ID=S2009_raw:GT=hom:DP=4:FQ=1:SQ=-1;ID=S2503_raw:GT=hom:DP=9:FQ=1:SQ=-1;ID=S2507_raw:GT=hom:DP=7:FQ=1:SQ=-1;ID=S2668_raw:GT=hom:DP=31:FQ=1:SQ=-1;ID=S2012_raw:GT=hom:DP=4:FQ=1:SQ=-1;ID=S2010_raw:GT=hom:DP=2:FQ=1:SQ=-1;ID=S2662_raw:GT=hom:DP=29:FQ=1:SQ=-1;ID=S2663_raw:GT=hom:DP=21:FQ=1:SQ=-1;ID=S2011_raw:GT=hom:DP=3:FQ=1:SQ=-1;ID=S2504_raw:GT=hom:DP=6:FQ=1:SQ=-1;ID=S2008_raw:GT=hom:DP=2:FQ=1:SQ=-1;ID=S2509_raw:GT=hom:DP=12:FQ=1:SQ=-1;ID=S2508_raw:GT=hom:DP=13:FQ=1:SQ=-1;ID=S2666_raw:GT=hom:DP=19:FQ=1:SQ=-1;ID=S2669_raw:GT=hom:DP=9:FQ=1:SQ=-1;ID=S2166_raw:GT=hom:DP=14:FQ=1:SQ=-1;ID=S1775_raw:GT=hom:DP=6:FQ=1:SQ=-1;ID=S2501_raw:GT=hom:DP=5:FQ=1:SQ=-1;ID=S2500_raw:GT=hom:DP=14:FQ=1:SQ=-1;ID=S2502_raw:GT=hom:DP=28:FQ=1:SQ=-1;ID=S2505_raw:GT=hom:DP=17:FQ=1:SQ=-1;ID=S2176_raw:GT=hom:DP=12:FQ=1:SQ=-1;ID=S2506_raw:GT=hom:DP=12:FQ=1:SQ=-1;ID=S2667_raw:GT=hom:DP=26:FQ=1:SQ=-1;
X	48649449	48649449	T	G	intronic	GATA1	.	.	rs62600348	.	ID=S2009_raw:GT=hom:DP=5:FQ=1:SQ=-1;ID=S2503_raw:GT=hom:DP=18:FQ=1:SQ=-1;ID=S2507_raw:GT=hom:DP=17:FQ=1:SQ=-1;ID=S2668_raw:GT=hom:DP=43:FQ=1:SQ=-1;ID=S2012_raw:GT=hom:DP=3:FQ=1:SQ=-1;ID=S2010_raw:GT=hom:DP=3:FQ=1:SQ=-1;ID=S2662_raw:GT=hom:DP=60:FQ=1:SQ=-1;ID=S2663_raw:GT=hom:DP=29:FQ=1:SQ=-1;ID=S2011_raw:GT=hom:DP=6:FQ=1:SQ=-1;ID=S2504_raw:GT=hom:DP=22:FQ=1:SQ=-1;ID=S2008_raw:GT=hom:DP=4:FQ=1:SQ=-1;ID=S2509_raw:GT=hom:DP=18:FQ=1:SQ=-1;ID=S2508_raw:GT=hom:DP=22:FQ=1:SQ=-1;ID=S2666_raw:GT=hom:DP=38:FQ=1:SQ=-1;ID=S2669_raw:GT=hom:DP=15:FQ=1:SQ=-1;ID=S2166_raw:GT=hom:DP=23:FQ=1:SQ=-1;ID=S1775_raw:GT=hom:DP=10:FQ=1:SQ=-1;ID=S2501_raw:GT=hom:DP=16:FQ=1:SQ=-1;ID=S2500_raw:GT=hom:DP=25:FQ=1:SQ=-1;ID=S2502_raw:GT=hom:DP=48:FQ=1:SQ=-1;ID=S2505_raw:GT=hom:DP=30:FQ=1:SQ=-1;ID=S2176_raw:GT=hom:DP=22:FQ=1:SQ=-1;ID=S2506_raw:GT=hom:DP=24:FQ=1:SQ=-1;ID=S2667_raw:GT=hom:DP=45:FQ=1:SQ=-1;
X	48649456	48649456	T	G	intronic	GATA1	.	.	rs66717003	.	ID=S2009_raw:GT=hom:DP=5:FQ=1:SQ=-1;ID=S2503_raw:GT=hom:DP=19:FQ=1:SQ=-1;ID=S2507_raw:GT=hom:DP=20:FQ=1:SQ=-1;ID=S2668_raw:GT=hom:DP=47:FQ=1:SQ=-1;ID=S2012_raw:GT=hom:DP=3:FQ=1:SQ=-1;ID=S2010_raw:GT=hom:DP=4:FQ=1:SQ=-1;ID=S2662_raw:GT=hom:DP=63:FQ=1:SQ=-1;ID=S2663_raw:GT=hom:DP=32:FQ=1:SQ=-1;ID=S2011_raw:GT=hom:DP=8:FQ=1:SQ=-1;ID=S2504_raw:GT=hom:DP=26:FQ=1:SQ=-1;ID=S2008_raw:GT=hom:DP=4:FQ=1:SQ=-1;ID=S2509_raw:GT=hom:DP=18:FQ=1:SQ=-1;ID=S2508_raw:GT=hom:DP=31:FQ=1:SQ=-1;ID=S2666_raw:GT=hom:DP=44:FQ=1:SQ=-1;ID=S2669_raw:GT=hom:DP=19:FQ=1:SQ=-1;ID=S2166_raw:GT=hom:DP=22:FQ=1:SQ=-1;ID=S1775_raw:GT=hom:DP=11:FQ=1:SQ=-1;ID=S2501_raw:GT=hom:DP=17:FQ=1:SQ=-1;ID=S2500_raw:GT=hom:DP=24:FQ=1:SQ=-1;ID=S2502_raw:GT=hom:DP=53:FQ=1:SQ=-1;ID=S2505_raw:GT=hom:DP=33:FQ=1:SQ=-1;ID=S2176_raw:GT=hom:DP=22:FQ=1:SQ=-1;ID=S2506_raw:GT=hom:DP=26:FQ=1:SQ=-1;ID=S2667_raw:GT=hom:DP=48:FQ=1:SQ=-1;
3	81643167	81643167	T	C	exonic;splicing	GBE1	nonsynonymous SNV	GBE1:NM_000158:exon8:c.1000A>G:p.I334V	rs2172397	.	ID=S2009_raw:GT=hom:DP=31:FQ=1:SQ=-1;ID=S2503_raw:GT=hom:DP=67:FQ=1:SQ=-1;ID=S2507_raw:GT=hom:DP=67:FQ=1:SQ=-1;ID=S2668_raw:GT=hom:DP=98:FQ=1:SQ=-1;ID=S2012_raw:GT=hom:DP=42:FQ=1:SQ=-1;ID=S2010_raw:GT=hom:DP=46:FQ=1:SQ=-1;ID=S2662_raw:GT=hom:DP=1:FQ=1:SQ=-1;ID=S2663_raw:GT=hom:DP=96:FQ=1:SQ=-1;ID=S2011_raw:GT=hom:DP=57:FQ=1:SQ=-1;ID=S2504_raw:GT=hom:DP=65:FQ=1:SQ=-1;ID=S2008_raw:GT=hom:DP=43:FQ=1:SQ=-1;ID=S2509_raw:GT=hom:DP=56:FQ=1:SQ=-1;ID=S2508_raw:GT=hom:DP=91:FQ=1:SQ=-1;ID=S2666_raw:GT=hom:DP=108:FQ=1:SQ=-1;ID=S2669_raw:GT=hom:DP=38:FQ=1:SQ=-1;ID=S2166_raw:GT=hom:DP=165:FQ=1:SQ=-1;ID=S1775_raw:GT=hom:DP=139:FQ=1:SQ=-1;ID=S2501_raw:GT=hom:DP=77:FQ=1:SQ=-1;ID=S2500_raw:GT=hom:DP=71:FQ=1:SQ=-1;ID=S2502_raw:GT=hom:DP=69:FQ=1:SQ=-1;ID=S2505_raw:GT=hom:DP=59:FQ=1:SQ=-1;ID=S2176_raw:GT=hom:DP=172:FQ=1:SQ=-1;ID=S2506_raw:GT=hom:DP=49:FQ=1:SQ=-1;ID=S2667_raw:GT=hom:DP=64:FQ=1:SQ=-1;
3	81810704	81810704	-	G	UTR5	GBE1	.	NM_000158:c.-37_-36insC	.	.	ID=S2009_raw:GT=hom:DP=8:FQ=1:SQ=-1;ID=S2503_raw:GT=hom:DP=26:FQ=1:SQ=-1;ID=S2507_raw:GT=hom:DP=38:FQ=1:SQ=-1;ID=S2668_raw:GT=hom:DP=52:FQ=1:SQ=-1;ID=S2012_raw:GT=hom:DP=10:FQ=1:SQ=-1;ID=S2010_raw:GT=hom:DP=13:FQ=1:SQ=-1;ID=S2662_raw:GT=hom:DP=123:FQ=1:SQ=-1;ID=S2663_raw:GT=hom:DP=71:FQ=1:SQ=-1;ID=S2011_raw:GT=hom:DP=15:FQ=1:SQ=-1;ID=S2504_raw:GT=hom:DP=38:FQ=1:SQ=-1;ID=S2008_raw:GT=hom:DP=18:FQ=1:SQ=-1;ID=S2509_raw:GT=hom:DP=35:FQ=1:SQ=-1;ID=S2508_raw:GT=hom:DP=44:FQ=1:SQ=-1;ID=S2666_raw:GT=hom:DP=18:FQ=1:SQ=-1;ID=S2669_raw:GT=hom:DP=61:FQ=1:SQ=-1;ID=S2166_raw:GT=hom:DP=34:FQ=1:SQ=-1;ID=S1775_raw:GT=hom:DP=16:FQ=1:SQ=-1;ID=S2501_raw:GT=hom:DP=41:FQ=1:SQ=-1;ID=S2500_raw:GT=hom:DP=27:FQ=1:SQ=-1;ID=S2502_raw:GT=hom:DP=34:FQ=1:SQ=-1;ID=S2505_raw:GT=hom:DP=46:FQ=1:SQ=-1;ID=S2176_raw:GT=hom:DP=23:FQ=1:SQ=-1;ID=S2506_raw:GT=hom:DP=36:FQ=1:SQ=-1;ID=S2667_raw:GT=hom:DP=66:FQ=1:SQ=-1;
6	53365019	53365019	G	C	intronic	GCLC	.	.	rs661346	.	ID=S2009_raw:GT=hom:DP=59:FQ=1:SQ=-1;ID=S2503_raw:GT=hom:DP=186:FQ=1:SQ=-1;ID=S2507_raw:GT=hom:DP=171:FQ=1:SQ=-1;ID=S2668_raw:GT=hom:DP=220:FQ=1:SQ=-1;ID=S2012_raw:GT=hom:DP=76:FQ=1:SQ=-1;ID=S2010_raw:GT=hom:DP=78:FQ=1:SQ=-1;ID=S2662_raw:GT=hom:DP=55:FQ=1:SQ=-1;ID=S2663_raw:GT=hom:DP=166:FQ=1:SQ=-1;ID=S2011_raw:GT=hom:DP=95:FQ=1:SQ=-1;ID=S2504_raw:GT=hom:DP=216:FQ=1:SQ=-1;ID=S2008_raw:GT=hom:DP=88:FQ=1:SQ=-1;ID=S2509_raw:GT=hom:DP=167:FQ=1:SQ=-1;ID=S2508_raw:GT=hom:DP=180:FQ=1:SQ=-1;ID=S2666_raw:GT=hom:DP=279:FQ=1:SQ=-1;ID=S2669_raw:GT=hom:DP=153:FQ=1:SQ=-1;ID=S2166_raw:GT=hom:DP=244:FQ=1:SQ=-1;ID=S1775_raw:GT=hom:DP=164:FQ=1:SQ=-1;ID=S2501_raw:GT=hom:DP=179:FQ=1:SQ=-1;ID=S2500_raw:GT=hom:DP=167:FQ=1:SQ=-1;ID=S2502_raw:GT=hom:DP=163:FQ=1:SQ=-1;ID=S2505_raw:GT=hom:DP=169:FQ=1:SQ=-1;ID=S2176_raw:GT=hom:DP=227:FQ=1:SQ=-1;ID=S2506_raw:GT=hom:DP=117:FQ=1:SQ=-1;ID=S2667_raw:GT=hom:DP=163:FQ=1:SQ=-1;
6	10882100	10882100	G	A	upstream	GCM2	.	dist=2	rs2153156	Benign	ID=S2009_raw:GT=hom:DP=69:FQ=1:SQ=-1;ID=S2503_raw:GT=hom:DP=74:FQ=1:SQ=-1;ID=S2507_raw:GT=hom:DP=82:FQ=1:SQ=-1;ID=S2668_raw:GT=hom:DP=50:FQ=1:SQ=-1;ID=S2012_raw:GT=hom:DP=58:FQ=1:SQ=-1;ID=S2010_raw:GT=hom:DP=58:FQ=1:SQ=-1;ID=S2662_raw:GT=hom:DP=85:FQ=1:SQ=-1;ID=S2663_raw:GT=hom:DP=56:FQ=1:SQ=-1;ID=S2011_raw:GT=hom:DP=83:FQ=1:SQ=-1;ID=S2504_raw:GT=hom:DP=66:FQ=1:SQ=-1;ID=S2008_raw:GT=hom:DP=69:FQ=1:SQ=-1;ID=S2509_raw:GT=hom:DP=87:FQ=1:SQ=-1;ID=S2508_raw:GT=hom:DP=73:FQ=0.99:SQ=-1;ID=S2666_raw:GT=hom:DP=58:FQ=1:SQ=-1;ID=S2669_raw:GT=hom:DP=73:FQ=1:SQ=-1;ID=S2166_raw:GT=hom:DP=50:FQ=1:SQ=-1;ID=S1775_raw:GT=hom:DP=43:FQ=1:SQ=-1;ID=S2501_raw:GT=hom:DP=77:FQ=1:SQ=-1;ID=S2500_raw:GT=hom:DP=48:FQ=1:SQ=-1;ID=S2502_raw:GT=hom:DP=72:FQ=1:SQ=-1;ID=S2505_raw:GT=hom:DP=72:FQ=1:SQ=-1;ID=S2176_raw:GT=hom:DP=57:FQ=1:SQ=-1;ID=S2506_raw:GT=hom:DP=72:FQ=1:SQ=-1;ID=S2667_raw:GT=hom:DP=84:FQ=1:SQ=-1;
12	7843473	7843473	T	C	intronic	GDF3	.	.	rs7957693	.	ID=S2009_raw:GT=hom:DP=1:FQ=1:SQ=-1;ID=S2503_raw:GT=hom:DP=4:FQ=1:SQ=-1;ID=S2507_raw:GT=hom:DP=8:FQ=1:SQ=-1;ID=S2668_raw:GT=hom:DP=2:FQ=1:SQ=-1;ID=S2012_raw:GT=hom:DP=1:FQ=1:SQ=-1;ID=S2010_raw:GT=hom:DP=1:FQ=1:SQ=-1;ID=S2662_raw:GT=hom:DP=9:FQ=1:SQ=-1;ID=S2663_raw:GT=hom:DP=9:FQ=1:SQ=-1;ID=S2011_raw:GT=hom:DP=1:FQ=1:SQ=-1;ID=S2504_raw:GT=hom:DP=11:FQ=1:SQ=-1;ID=S2008_raw:GT=hom:DP=6:FQ=1:SQ=-1;ID=S2509_raw:GT=hom:DP=6:FQ=1:SQ=-1;ID=S2508_raw:GT=hom:DP=5:FQ=1:SQ=-1;ID=S2666_raw:GT=hom:DP=1:FQ=1:SQ=-1;ID=S2669_raw:GT=hom:DP=3:FQ=1:SQ=-1;ID=S2166_raw:GT=hom:DP=6:FQ=1:SQ=-1;ID=S1775_raw:GT=hom:DP=5:FQ=1:SQ=-1;ID=S2501_raw:GT=hom:DP=4:FQ=1:SQ=-1;ID=S2500_raw:GT=hom:DP=8:FQ=1:SQ=-1;ID=S2502_raw:GT=hom:DP=7:FQ=1:SQ=-1;ID=S2505_raw:GT=hom:DP=8:FQ=1:SQ=-1;ID=S2176_raw:GT=hom:DP=7:FQ=1:SQ=-1;ID=S2506_raw:GT=hom:DP=5:FQ=1:SQ=-1;ID=S2667_raw:GT=hom:DP=2:FQ=1:SQ=-1;
12	7843478	7843478	C	T	intronic	GDF3	.	.	rs7976851	.	ID=S2009_raw:GT=hom:DP=1:FQ=1:SQ=-1;ID=S2503_raw:GT=hom:DP=3:FQ=1:SQ=-1;ID=S2507_raw:GT=hom:DP=8:FQ=1:SQ=-1;ID=S2668_raw:GT=hom:DP=1:FQ=1:SQ=-1;ID=S2012_raw:GT=hom:DP=1:FQ=1:SQ=-1;ID=S2010_raw:GT=hom:DP=1:FQ=1:SQ=-1;ID=S2662_raw:GT=hom:DP=9:FQ=1:SQ=-1;ID=S2663_raw:GT=hom:DP=9:FQ=1:SQ=-1;ID=S2011_raw:GT=hom:DP=1:FQ=1:SQ=-1;ID=S2504_raw:GT=hom:DP=9:FQ=1:SQ=-1;ID=S2008_raw:GT=hom:DP=5:FQ=1:SQ=-1;ID=S2509_raw:GT=hom:DP=5:FQ=1:SQ=-1;ID=S2508_raw:GT=hom:DP=5:FQ=1:SQ=-1;ID=S2666_raw:GT=hom:DP=1:FQ=1:SQ=-1;ID=S2669_raw:GT=hom:DP=3:FQ=1:SQ=-1;ID=S2166_raw:GT=hom:DP=6:FQ=1:SQ=-1;ID=S1775_raw:GT=hom:DP=3:FQ=1:SQ=-1;ID=S2501_raw:GT=hom:DP=3:FQ=1:SQ=-1;ID=S2500_raw:GT=hom:DP=8:FQ=1:SQ=-1;ID=S2502_raw:GT=hom:DP=6:FQ=1:SQ=-1;ID=S2505_raw:GT=hom:DP=7:FQ=1:SQ=-1;ID=S2176_raw:GT=hom:DP=2:FQ=1:SQ=-1;ID=S2506_raw:GT=hom:DP=5:FQ=1:SQ=-1;ID=S2667_raw:GT=hom:DP=2:FQ=1:SQ=-1;
12	7847940	7847940	C	T	intronic	GDF3	.	.	rs6488591	.	ID=S2009_raw:GT=hom:DP=7:FQ=1:SQ=-1;ID=S2503_raw:GT=hom:DP=64:FQ=1:SQ=-1;ID=S2507_raw:GT=hom:DP=53:FQ=1:SQ=-1;ID=S2668_raw:GT=hom:DP=80:FQ=1:SQ=-1;ID=S2012_raw:GT=hom:DP=9:FQ=1:SQ=-1;ID=S2010_raw:GT=hom:DP=12:FQ=1:SQ=-1;ID=S2662_raw:GT=hom:DP=5:FQ=1:SQ=-1;ID=S2663_raw:GT=hom:DP=55:FQ=1:SQ=-1;ID=S2011_raw:GT=hom:DP=16:FQ=1:SQ=-1;ID=S2504_raw:GT=hom:DP=53:FQ=1:SQ=-1;ID=S2008_raw:GT=hom:DP=15:FQ=1:SQ=-1;ID=S2509_raw:GT=hom:DP=46:FQ=1:SQ=-1;ID=S2508_raw:GT=hom:DP=47:FQ=1:SQ=-1;ID=S2666_raw:GT=hom:DP=97:FQ=1:SQ=-1;ID=S2669_raw:GT=hom:DP=40:FQ=1:SQ=-1;ID=S2166_raw:GT=hom:DP=9:FQ=1:SQ=-1;ID=S1775_raw:GT=hom:DP=9:FQ=1:SQ=-1;ID=S2501_raw:GT=hom:DP=38:FQ=1:SQ=-1;ID=S2500_raw:GT=hom:DP=47:FQ=1:SQ=-1;ID=S2502_raw:GT=hom:DP=63:FQ=1:SQ=-1;ID=S2505_raw:GT=hom:DP=59:FQ=1:SQ=-1;ID=S2176_raw:GT=hom:DP=16:FQ=1:SQ=-1;ID=S2506_raw:GT=hom:DP=51:FQ=1:SQ=-1;ID=S2667_raw:GT=hom:DP=69:FQ=1:SQ=-1;
10	1,18E+08	1,18E+08	C	A	intronic	GFRA1	.	.	rs2532691	.	ID=S2009_raw:GT=hom:DP=2:FQ=1:SQ=-1;ID=S2503_raw:GT=hom:DP=85:FQ=1:SQ=-1;ID=S2507_raw:GT=hom:DP=64:FQ=1:SQ=-1;ID=S2668_raw:GT=hom:DP=76:FQ=1:SQ=-1;ID=S2012_raw:GT=hom:DP=3:FQ=1:SQ=-1;ID=S2010_raw:GT=hom:DP=3:FQ=1:SQ=-1;ID=S2662_raw:GT=hom:DP=130:FQ=0.99:SQ=-1;ID=S2663_raw:GT=hom:DP=67:FQ=1:SQ=-1;ID=S2011_raw:GT=hom:DP=5:FQ=1:SQ=-1;ID=S2504_raw:GT=hom:DP=83:FQ=1:SQ=-1;ID=S2008_raw:GT=hom:DP=4:FQ=1:SQ=-1;ID=S2509_raw:GT=hom:DP=87:FQ=1:SQ=-1;ID=S2508_raw:GT=hom:DP=91:FQ=1:SQ=-1;ID=S2666_raw:GT=hom:DP=39:FQ=1:SQ=-1;ID=S2669_raw:GT=hom:DP=73:FQ=1:SQ=-1;ID=S2166_raw:GT=hom:DP=84:FQ=1:SQ=-1;ID=S1775_raw:GT=hom:DP=67:FQ=1:SQ=-1;ID=S2501_raw:GT=hom:DP=79:FQ=1:SQ=-1;ID=S2500_raw:GT=hom:DP=63:FQ=1:SQ=-1;ID=S2502_raw:GT=hom:DP=65:FQ=1:SQ=-1;ID=S2505_raw:GT=hom:DP=66:FQ=1:SQ=-1;ID=S2176_raw:GT=hom:DP=79:FQ=1:SQ=-1;ID=S2506_raw:GT=hom:DP=96:FQ=1:SQ=-1;ID=S2667_raw:GT=hom:DP=125:FQ=1:SQ=-1;
12	57865928	57865928	A	C	UTR3	GLI1	.	NM_005269:c.*84A>C;NM_001160045:c.*84A>C;NM_001167609:c.*84A>C	rs474058	.	ID=S2009_raw:GT=hom:DP=67:FQ=1:SQ=-1;ID=S2503_raw:GT=hom:DP=33:FQ=1:SQ=-1;ID=S2507_raw:GT=hom:DP=39:FQ=1:SQ=-1;ID=S2668_raw:GT=hom:DP=40:FQ=1:SQ=-1;ID=S2012_raw:GT=hom:DP=83:FQ=1:SQ=-1;ID=S2010_raw:GT=hom:DP=82:FQ=1:SQ=-1;ID=S2662_raw:GT=hom:DP=25:FQ=1:SQ=-1;ID=S2663_raw:GT=hom:DP=25:FQ=1:SQ=-1;ID=S2011_raw:GT=hom:DP=134:FQ=1:SQ=-1;ID=S2504_raw:GT=hom:DP=26:FQ=1:SQ=-1;ID=S2008_raw:GT=hom:DP=106:FQ=1:SQ=-1;ID=S2509_raw:GT=hom:DP=27:FQ=1:SQ=-1;ID=S2508_raw:GT=hom:DP=33:FQ=1:SQ=-1;ID=S2666_raw:GT=hom:DP=35:FQ=1:SQ=-1;ID=S2669_raw:GT=hom:DP=37:FQ=1:SQ=-1;ID=S2166_raw:GT=hom:DP=8:FQ=1:SQ=-1;ID=S1775_raw:GT=hom:DP=7:FQ=1:SQ=-1;ID=S2501_raw:GT=hom:DP=27:FQ=1:SQ=-1;ID=S2500_raw:GT=hom:DP=33:FQ=1:SQ=-1;ID=S2502_raw:GT=hom:DP=30:FQ=1:SQ=-1;ID=S2505_raw:GT=hom:DP=34:FQ=1:SQ=-1;ID=S2176_raw:GT=hom:DP=15:FQ=1:SQ=-1;ID=S2506_raw:GT=hom:DP=29:FQ=1:SQ=-1;ID=S2667_raw:GT=hom:DP=31:FQ=1:SQ=-1;
2	1,22E+08	1,22E+08	C	G	intronic	GLI2	.	.	rs6718382	Benign	ID=S2009_raw:GT=hom:DP=15:FQ=1:SQ=-1;ID=S2503_raw:GT=hom:DP=47:FQ=1:SQ=-1;ID=S2507_raw:GT=hom:DP=55:FQ=1:SQ=-1;ID=S2668_raw:GT=hom:DP=39:FQ=1:SQ=-1;ID=S2012_raw:GT=hom:DP=13:FQ=1:SQ=-1;ID=S2010_raw:GT=hom:DP=13:FQ=0.92:SQ=-1;ID=S2662_raw:GT=hom:DP=93:FQ=1:SQ=-1;ID=S2663_raw:GT=hom:DP=44:FQ=1:SQ=-1;ID=S2011_raw:GT=hom:DP=21:FQ=1:SQ=-1;ID=S2504_raw:GT=hom:DP=49:FQ=1:SQ=-1;ID=S2008_raw:GT=hom:DP=18:FQ=1:SQ=-1;ID=S2509_raw:GT=hom:DP=41:FQ=1:SQ=-1;ID=S2508_raw:GT=hom:DP=58:FQ=1:SQ=-1;ID=S2666_raw:GT=hom:DP=41:FQ=1:SQ=-1;ID=S2669_raw:GT=hom:DP=37:FQ=1:SQ=-1;ID=S2166_raw:GT=hom:DP=52:FQ=1:SQ=-1;ID=S1775_raw:GT=hom:DP=46:FQ=1:SQ=-1;ID=S2501_raw:GT=hom:DP=42:FQ=1:SQ=-1;ID=S2500_raw:GT=hom:DP=61:FQ=1:SQ=-1;ID=S2502_raw:GT=hom:DP=63:FQ=1:SQ=-1;ID=S2505_raw:GT=hom:DP=47:FQ=1:SQ=-1;ID=S2176_raw:GT=hom:DP=58:FQ=1:SQ=-1;ID=S2506_raw:GT=hom:DP=56:FQ=1:SQ=-1;ID=S2667_raw:GT=hom:DP=61:FQ=1:SQ=-1;
2	1,22E+08	1,22E+08	T	C	intronic	GLI2	.	.	rs6740871	.	ID=S2009_raw:GT=hom:DP=1:FQ=1:SQ=-1;ID=S2503_raw:GT=hom:DP=5:FQ=1:SQ=-1;ID=S2507_raw:GT=hom:DP=5:FQ=1:SQ=-1;ID=S2668_raw:GT=hom:DP=6:FQ=1:SQ=-1;ID=S2012_raw:GT=hom:DP=2:FQ=1:SQ=-1;ID=S2010_raw:GT=hom:DP=3:FQ=1:SQ=-1;ID=S2662_raw:GT=hom:DP=17:FQ=1:SQ=-1;ID=S2663_raw:GT=hom:DP=6:FQ=1:SQ=-1;ID=S2011_raw:GT=hom:DP=4:FQ=1:SQ=-1;ID=S2504_raw:GT=hom:DP=1:FQ=1:SQ=-1;ID=S2008_raw:GT=hom:DP=2:FQ=1:SQ=-1;ID=S2509_raw:GT=hom:DP=7:FQ=1:SQ=-1;ID=S2508_raw:GT=hom:DP=6:FQ=1:SQ=-1;ID=S2666_raw:GT=hom:DP=2:FQ=1:SQ=-1;ID=S2669_raw:GT=hom:DP=8:FQ=1:SQ=-1;ID=S2166_raw:GT=hom:DP=4:FQ=1:SQ=-1;ID=S1775_raw:GT=hom:DP=1:FQ=1:SQ=-1;ID=S2501_raw:GT=hom:DP=7:FQ=1:SQ=-1;ID=S2500_raw:GT=hom:DP=10:FQ=1:SQ=-1;ID=S2502_raw:GT=hom:DP=6:FQ=1:SQ=-1;ID=S2505_raw:GT=hom:DP=7:FQ=1:SQ=-1;ID=S2176_raw:GT=hom:DP=6:FQ=1:SQ=-1;ID=S2506_raw:GT=hom:DP=5:FQ=1:SQ=-1;ID=S2667_raw:GT=hom:DP=7:FQ=1:SQ=-1;
2	1,22E+08	1,22E+08	G	A	intronic	GLI2	.	.	rs1867906	.	ID=S2009_raw:GT=hom:DP=2:FQ=1:SQ=-1;ID=S2503_raw:GT=hom:DP=7:FQ=1:SQ=-1;ID=S2507_raw:GT=hom:DP=5:FQ=1:SQ=-1;ID=S2668_raw:GT=hom:DP=2:FQ=1:SQ=-1;ID=S2012_raw:GT=hom:DP=2:FQ=1:SQ=-1;ID=S2010_raw:GT=hom:DP=4:FQ=1:SQ=-1;ID=S2662_raw:GT=hom:DP=13:FQ=1:SQ=-1;ID=S2663_raw:GT=hom:DP=2:FQ=1:SQ=-1;ID=S2011_raw:GT=hom:DP=6:FQ=1:SQ=-1;ID=S2504_raw:GT=hom:DP=3:FQ=1:SQ=-1;ID=S2008_raw:GT=hom:DP=2:FQ=1:SQ=-1;ID=S2509_raw:GT=hom:DP=4:FQ=1:SQ=-1;ID=S2508_raw:GT=hom:DP=4:FQ=1:SQ=-1;ID=S2666_raw:GT=hom:DP=4:FQ=1:SQ=-1;ID=S2669_raw:GT=hom:DP=3:FQ=1:SQ=-1;ID=S2166_raw:GT=hom:DP=2:FQ=1:SQ=-1;ID=S1775_raw:GT=hom:DP=4:FQ=1:SQ=-1;ID=S2501_raw:GT=hom:DP=8:FQ=1:SQ=-1;ID=S2500_raw:GT=hom:DP=5:FQ=1:SQ=-1;ID=S2502_raw:GT=hom:DP=11:FQ=1:SQ=-1;ID=S2505_raw:GT=hom:DP=5:FQ=1:SQ=-1;ID=S2176_raw:GT=hom:DP=7:FQ=1:SQ=-1;ID=S2506_raw:GT=hom:DP=7:FQ=1:SQ=-1;ID=S2667_raw:GT=hom:DP=7:FQ=1:SQ=-1;
2	1,22E+08	1,22E+08	T	G	intronic	GLI2	.	.	rs1530580	.	ID=S2009_raw:GT=hom:DP=19:FQ=1:SQ=-1;ID=S2503_raw:GT=hom:DP=47:FQ=1:SQ=-1;ID=S2507_raw:GT=hom:DP=44:FQ=1:SQ=-1;ID=S2668_raw:GT=hom:DP=33:FQ=1:SQ=-1;ID=S2012_raw:GT=hom:DP=17:FQ=1:SQ=-1;ID=S2010_raw:GT=hom:DP=15:FQ=1:SQ=-1;ID=S2662_raw:GT=hom:DP=51:FQ=1:SQ=-1;ID=S2663_raw:GT=hom:DP=35:FQ=1:SQ=-1;ID=S2011_raw:GT=hom:DP=27:FQ=1:SQ=-1;ID=S2504_raw:GT=hom:DP=36:FQ=1:SQ=-1;ID=S2008_raw:GT=hom:DP=14:FQ=1:SQ=-1;ID=S2509_raw:GT=hom:DP=48:FQ=1:SQ=-1;ID=S2508_raw:GT=hom:DP=45:FQ=1:SQ=-1;ID=S2666_raw:GT=hom:DP=29:FQ=1:SQ=-1;ID=S2669_raw:GT=hom:DP=35:FQ=1:SQ=-1;ID=S2166_raw:GT=hom:DP=47:FQ=1:SQ=-1;ID=S1775_raw:GT=hom:DP=27:FQ=1:SQ=-1;ID=S2501_raw:GT=hom:DP=28:FQ=1:SQ=-1;ID=S2500_raw:GT=hom:DP=39:FQ=1:SQ=-1;ID=S2502_raw:GT=hom:DP=60:FQ=1:SQ=-1;ID=S2505_raw:GT=hom:DP=45:FQ=1:SQ=-1;ID=S2176_raw:GT=hom:DP=48:FQ=1:SQ=-1;ID=S2506_raw:GT=hom:DP=49:FQ=1:SQ=-1;ID=S2667_raw:GT=hom:DP=38:FQ=1:SQ=-1;
9	3829631	3829631	C	T	intronic	GLIS3	.	.	rs2181578	.	ID=S2009_raw:GT=hom:DP=3:FQ=1:SQ=-1;ID=S2503_raw:GT=hom:DP=69:FQ=1:SQ=-1;ID=S2507_raw:GT=hom:DP=63:FQ=1:SQ=-1;ID=S2668_raw:GT=hom:DP=67:FQ=1:SQ=-1;ID=S2012_raw:GT=hom:DP=4:FQ=1:SQ=-1;ID=S2010_raw:GT=hom:DP=7:FQ=1:SQ=-1;ID=S2662_raw:GT=hom:DP=12:FQ=1:SQ=-1;ID=S2663_raw:GT=hom:DP=48:FQ=1:SQ=-1;ID=S2011_raw:GT=hom:DP=7:FQ=1:SQ=-1;ID=S2504_raw:GT=hom:DP=42:FQ=1:SQ=-1;ID=S2008_raw:GT=hom:DP=4:FQ=1:SQ=-1;ID=S2509_raw:GT=hom:DP=64:FQ=1:SQ=-1;ID=S2508_raw:GT=hom:DP=78:FQ=1:SQ=-1;ID=S2666_raw:GT=hom:DP=73:FQ=1:SQ=-1;ID=S2669_raw:GT=hom:DP=57:FQ=1:SQ=-1;ID=S2166_raw:GT=hom:DP=11:FQ=1:SQ=-1;ID=S1775_raw:GT=hom:DP=8:FQ=1:SQ=-1;ID=S2501_raw:GT=hom:DP=71:FQ=1:SQ=-1;ID=S2500_raw:GT=hom:DP=69:FQ=1:SQ=-1;ID=S2502_raw:GT=hom:DP=76:FQ=1:SQ=-1;ID=S2505_raw:GT=hom:DP=54:FQ=1:SQ=-1;ID=S2176_raw:GT=hom:DP=11:FQ=1:SQ=-1;ID=S2506_raw:GT=hom:DP=42:FQ=1:SQ=-1;ID=S2667_raw:GT=hom:DP=91:FQ=1:SQ=-1;
9	4118208	4118208	A	G	exonic	GLIS3	nonsynonymous SNV	GLIS3:NM_152629:exon3:c.805T>C:p.S269P,GLIS3:NM_001042413:exon4:c.1270T>C:p.S424P	rs806052	Benign	ID=S2009_raw:GT=hom:DP=29:FQ=1:SQ=-1;ID=S2503_raw:GT=hom:DP=73:FQ=1:SQ=-1;ID=S2507_raw:GT=hom:DP=81:FQ=1:SQ=-1;ID=S2668_raw:GT=hom:DP=136:FQ=1:SQ=-1;ID=S2012_raw:GT=hom:DP=21:FQ=1:SQ=-1;ID=S2010_raw:GT=hom:DP=28:FQ=1:SQ=-1;ID=S2662_raw:GT=hom:DP=184:FQ=1:SQ=-1;ID=S2663_raw:GT=hom:DP=124:FQ=1:SQ=-1;ID=S2011_raw:GT=hom:DP=54:FQ=1:SQ=-1;ID=S2504_raw:GT=hom:DP=115:FQ=1:SQ=-1;ID=S2008_raw:GT=hom:DP=42:FQ=1:SQ=-1;ID=S2509_raw:GT=hom:DP=78:FQ=1:SQ=-1;ID=S2508_raw:GT=hom:DP=110:FQ=1:SQ=-1;ID=S2666_raw:GT=hom:DP=72:FQ=1:SQ=-1;ID=S2669_raw:GT=hom:DP=91:FQ=0.98:SQ=-1;ID=S2166_raw:GT=hom:DP=96:FQ=1:SQ=-1;ID=S1775_raw:GT=hom:DP=52:FQ=1:SQ=-1;ID=S2501_raw:GT=hom:DP=83:FQ=1:SQ=-1;ID=S2500_raw:GT=hom:DP=75:FQ=1:SQ=-1;ID=S2502_raw:GT=hom:DP=103:FQ=1:SQ=-1;ID=S2505_raw:GT=hom:DP=81:FQ=0.99:SQ=-1;ID=S2176_raw:GT=hom:DP=62:FQ=1:SQ=-1;ID=S2506_raw:GT=hom:DP=71:FQ=1:SQ=-1;ID=S2667_raw:GT=hom:DP=102:FQ=1:SQ=-1;
10	75492698	75492698	-	C	intergenic	GLUD1P3;SEC24C	.	dist=1169;dist=11432	.	.	ID=S2009_raw:GT=hom:DP=18:FQ=1:SQ=-1;ID=S2503_raw:GT=hom:DP=14:FQ=1:SQ=-1;ID=S2507_raw:GT=hom:DP=16:FQ=1:SQ=-1;ID=S2668_raw:GT=hom:DP=7:FQ=1:SQ=-1;ID=S2012_raw:GT=hom:DP=4:FQ=1:SQ=-1;ID=S2010_raw:GT=hom:DP=11:FQ=1:SQ=-1;ID=S2662_raw:GT=hom:DP=26:FQ=1:SQ=-1;ID=S2663_raw:GT=hom:DP=12:FQ=1:SQ=-1;ID=S2011_raw:GT=hom:DP=23:FQ=1:SQ=-1;ID=S2504_raw:GT=hom:DP=7:FQ=1:SQ=-1;ID=S2008_raw:GT=hom:DP=13:FQ=1:SQ=-1;ID=S2509_raw:GT=hom:DP=12:FQ=1:SQ=-1;ID=S2508_raw:GT=hom:DP=13:FQ=1:SQ=-1;ID=S2666_raw:GT=hom:DP=12:FQ=1:SQ=-1;ID=S2669_raw:GT=hom:DP=21:FQ=1:SQ=-1;ID=S2166_raw:GT=hom:DP=14:FQ=1:SQ=-1;ID=S1775_raw:GT=hom:DP=3:FQ=1:SQ=-1;ID=S2501_raw:GT=hom:DP=9:FQ=1:SQ=-1;ID=S2500_raw:GT=hom:DP=7:FQ=1:SQ=-1;ID=S2502_raw:GT=hom:DP=7:FQ=1:SQ=-1;ID=S2505_raw:GT=hom:DP=12:FQ=1:SQ=-1;ID=S2176_raw:GT=hom:DP=12:FQ=1:SQ=-1;ID=S2506_raw:GT=hom:DP=9:FQ=1:SQ=-1;ID=S2667_raw:GT=hom:DP=19:FQ=1:SQ=-1;
10	75492712	75492712	-	C	intergenic	GLUD1P3;SEC24C	.	dist=1183;dist=11418	.	.	ID=S2009_raw:GT=hom:DP=15:FQ=1:SQ=-1;ID=S2503_raw:GT=hom:DP=18:FQ=1:SQ=-1;ID=S2507_raw:GT=hom:DP=14:FQ=1:SQ=-1;ID=S2668_raw:GT=hom:DP=6:FQ=1:SQ=-1;ID=S2012_raw:GT=hom:DP=10:FQ=1:SQ=-1;ID=S2010_raw:GT=hom:DP=21:FQ=1:SQ=-1;ID=S2662_raw:GT=hom:DP=23:FQ=1:SQ=-1;ID=S2663_raw:GT=hom:DP=14:FQ=1:SQ=-1;ID=S2011_raw:GT=hom:DP=28:FQ=1:SQ=-1;ID=S2504_raw:GT=hom:DP=14:FQ=1:SQ=-1;ID=S2008_raw:GT=hom:DP=18:FQ=1:SQ=-1;ID=S2509_raw:GT=hom:DP=8:FQ=1:SQ=-1;ID=S2508_raw:GT=hom:DP=8:FQ=1:SQ=-1;ID=S2666_raw:GT=hom:DP=12:FQ=1:SQ=-1;ID=S2669_raw:GT=hom:DP=21:FQ=1:SQ=-1;ID=S2166_raw:GT=hom:DP=16:FQ=1:SQ=-1;ID=S1775_raw:GT=hom:DP=4:FQ=1:SQ=-1;ID=S2501_raw:GT=hom:DP=9:FQ=1:SQ=-1;ID=S2500_raw:GT=hom:DP=10:FQ=1:SQ=-1;ID=S2502_raw:GT=hom:DP=8:FQ=1:SQ=-1;ID=S2505_raw:GT=hom:DP=13:FQ=1:SQ=-1;ID=S2176_raw:GT=hom:DP=16:FQ=1:SQ=-1;ID=S2506_raw:GT=hom:DP=12:FQ=1:SQ=-1;ID=S2667_raw:GT=hom:DP=19:FQ=1:SQ=-1;
17	4837955	4837955	-	G	UTR3	GP1BA	.	NM_000173:c.*97_*98insG	.	.	ID=S2009_raw:GT=hom:DP=12:FQ=1:SQ=-1;ID=S2503_raw:GT=hom:DP=12:FQ=1:SQ=-1;ID=S2507_raw:GT=hom:DP=13:FQ=1:SQ=-1;ID=S2668_raw:GT=hom:DP=8:FQ=1:SQ=-1;ID=S2012_raw:GT=hom:DP=15:FQ=1:SQ=-1;ID=S2010_raw:GT=hom:DP=12:FQ=1:SQ=-1;ID=S2662_raw:GT=hom:DP=18:FQ=1:SQ=-1;ID=S2663_raw:GT=hom:DP=7:FQ=1:SQ=-1;ID=S2011_raw:GT=hom:DP=17:FQ=1:SQ=-1;ID=S2504_raw:GT=hom:DP=17:FQ=1:SQ=-1;ID=S2008_raw:GT=hom:DP=15:FQ=1:SQ=-1;ID=S2509_raw:GT=hom:DP=17:FQ=1:SQ=-1;ID=S2508_raw:GT=hom:DP=16:FQ=1:SQ=-1;ID=S2666_raw:GT=hom:DP=20:FQ=1:SQ=-1;ID=S2669_raw:GT=hom:DP=22:FQ=1:SQ=-1;ID=S2166_raw:GT=hom:DP=21:FQ=1:SQ=-1;ID=S1775_raw:GT=hom:DP=13:FQ=1:SQ=-1;ID=S2501_raw:GT=hom:DP=12:FQ=1:SQ=-1;ID=S2500_raw:GT=hom:DP=14:FQ=1:SQ=-1;ID=S2502_raw:GT=hom:DP=12:FQ=1:SQ=-1;ID=S2505_raw:GT=hom:DP=12:FQ=1:SQ=-1;ID=S2176_raw:GT=hom:DP=25:FQ=1:SQ=-1;ID=S2506_raw:GT=hom:DP=10:FQ=1:SQ=-1;ID=S2667_raw:GT=hom:DP=16:FQ=1:SQ=-1;
7	51037728	51037728	T	-	intergenic	GRB10;COBL	.	dist=176569;dist=46181	.	.	ID=S2009_raw:GT=hom:DP=9:FQ=1:SQ=-1;ID=S2503_raw:GT=hom:DP=10:FQ=1:SQ=-1;ID=S2507_raw:GT=hom:DP=20:FQ=1:SQ=-1;ID=S2668_raw:GT=hom:DP=3:FQ=1:SQ=-1;ID=S2012_raw:GT=hom:DP=8:FQ=1:SQ=-1;ID=S2010_raw:GT=hom:DP=3:FQ=1:SQ=-1;ID=S2662_raw:GT=hom:DP=8:FQ=1:SQ=-1;ID=S2663_raw:GT=hom:DP=9:FQ=1:SQ=-1;ID=S2011_raw:GT=hom:DP=6:FQ=1:SQ=-1;ID=S2504_raw:GT=hom:DP=24:FQ=1:SQ=-1;ID=S2008_raw:GT=hom:DP=4:FQ=1:SQ=-1;ID=S2509_raw:GT=hom:DP=19:FQ=1:SQ=-1;ID=S2508_raw:GT=hom:DP=26:FQ=1:SQ=-1;ID=S2666_raw:GT=hom:DP=7:FQ=1:SQ=-1;ID=S2669_raw:GT=hom:DP=7:FQ=1:SQ=-1;ID=S2166_raw:GT=hom:DP=24:FQ=1:SQ=-1;ID=S1775_raw:GT=hom:DP=3:FQ=1:SQ=-1;ID=S2501_raw:GT=hom:DP=8:FQ=1:SQ=-1;ID=S2500_raw:GT=hom:DP=8:FQ=1:SQ=-1;ID=S2502_raw:GT=hom:DP=17:FQ=1:SQ=-1;ID=S2505_raw:GT=hom:DP=16:FQ=1:SQ=-1;ID=S2176_raw:GT=hom:DP=22:FQ=1:SQ=-1;ID=S2506_raw:GT=hom:DP=8:FQ=1:SQ=-1;ID=S2667_raw:GT=hom:DP=8:FQ=1:SQ=-1;
7	51037784	51037784	T	C	intergenic	GRB10;COBL	.	dist=176625;dist=46125	rs13310888	.	ID=S2009_raw:GT=hom:DP=11:FQ=1:SQ=-1;ID=S2503_raw:GT=hom:DP=15:FQ=1:SQ=-1;ID=S2507_raw:GT=hom:DP=19:FQ=1:SQ=-1;ID=S2668_raw:GT=hom:DP=11:FQ=1:SQ=-1;ID=S2012_raw:GT=hom:DP=12:FQ=1:SQ=-1;ID=S2010_raw:GT=hom:DP=3:FQ=1:SQ=-1;ID=S2662_raw:GT=hom:DP=8:FQ=1:SQ=-1;ID=S2663_raw:GT=hom:DP=16:FQ=1:SQ=-1;ID=S2011_raw:GT=hom:DP=9:FQ=1:SQ=-1;ID=S2504_raw:GT=hom:DP=22:FQ=1:SQ=-1;ID=S2008_raw:GT=hom:DP=9:FQ=1:SQ=-1;ID=S2509_raw:GT=hom:DP=20:FQ=1:SQ=-1;ID=S2508_raw:GT=hom:DP=27:FQ=1:SQ=-1;ID=S2666_raw:GT=hom:DP=5:FQ=1:SQ=-1;ID=S2669_raw:GT=hom:DP=7:FQ=1:SQ=-1;ID=S2166_raw:GT=hom:DP=27:FQ=1:SQ=-1;ID=S1775_raw:GT=hom:DP=5:FQ=1:SQ=-1;ID=S2501_raw:GT=hom:DP=13:FQ=1:SQ=-1;ID=S2500_raw:GT=hom:DP=6:FQ=1:SQ=-1;ID=S2502_raw:GT=hom:DP=15:FQ=1:SQ=-1;ID=S2505_raw:GT=hom:DP=22:FQ=1:SQ=-1;ID=S2176_raw:GT=hom:DP=30:FQ=1:SQ=-1;ID=S2506_raw:GT=hom:DP=9:FQ=1:SQ=-1;ID=S2667_raw:GT=hom:DP=4:FQ=1:SQ=-1;
18	19065494	19065494	C	G	intronic	GREB1L	.	.	rs7234335	.	ID=S2009_raw:GT=hom:DP=2:FQ=1:SQ=-1;ID=S2503_raw:GT=hom:DP=97:FQ=1:SQ=-1;ID=S2507_raw:GT=hom:DP=110:FQ=1:SQ=-1;ID=S2668_raw:GT=hom:DP=144:FQ=1:SQ=-1;ID=S2012_raw:GT=hom:DP=2:FQ=1:SQ=-1;ID=S2010_raw:GT=hom:DP=3:FQ=1:SQ=-1;ID=S2662_raw:GT=hom:DP=29:FQ=1:SQ=-1;ID=S2663_raw:GT=hom:DP=102:FQ=1:SQ=-1;ID=S2011_raw:GT=hom:DP=6:FQ=1:SQ=-1;ID=S2504_raw:GT=hom:DP=120:FQ=1:SQ=-1;ID=S2008_raw:GT=hom:DP=2:FQ=1:SQ=-1;ID=S2509_raw:GT=hom:DP=118:FQ=1:SQ=-1;ID=S2508_raw:GT=hom:DP=88:FQ=1:SQ=-1;ID=S2666_raw:GT=hom:DP=132:FQ=1:SQ=-1;ID=S2669_raw:GT=hom:DP=70:FQ=1:SQ=-1;ID=S2166_raw:GT=hom:DP=56:FQ=1:SQ=-1;ID=S1775_raw:GT=hom:DP=19:FQ=1:SQ=-1;ID=S2501_raw:GT=hom:DP=99:FQ=1:SQ=-1;ID=S2500_raw:GT=hom:DP=118:FQ=1:SQ=-1;ID=S2502_raw:GT=hom:DP=118:FQ=1:SQ=-1;ID=S2505_raw:GT=hom:DP=130:FQ=1:SQ=-1;ID=S2176_raw:GT=hom:DP=62:FQ=1:SQ=-1;ID=S2506_raw:GT=hom:DP=91:FQ=1:SQ=-1;ID=S2667_raw:GT=hom:DP=107:FQ=1:SQ=-1;
18	19079537	19079537	A	G	intronic	GREB1L	.	.	rs8087553	.	ID=S2009_raw:GT=hom:DP=11:FQ=1:SQ=-1;ID=S2503_raw:GT=hom:DP=15:FQ=1:SQ=-1;ID=S2507_raw:GT=hom:DP=26:FQ=1:SQ=-1;ID=S2668_raw:GT=hom:DP=45:FQ=1:SQ=-1;ID=S2012_raw:GT=hom:DP=14:FQ=1:SQ=-1;ID=S2010_raw:GT=hom:DP=14:FQ=1:SQ=-1;ID=S2662_raw:GT=hom:DP=42:FQ=1:SQ=-1;ID=S2663_raw:GT=hom:DP=18:FQ=1:SQ=-1;ID=S2011_raw:GT=hom:DP=25:FQ=1:SQ=-1;ID=S2504_raw:GT=hom:DP=30:FQ=1:SQ=-1;ID=S2008_raw:GT=hom:DP=22:FQ=1:SQ=-1;ID=S2509_raw:GT=hom:DP=32:FQ=1:SQ=-1;ID=S2508_raw:GT=hom:DP=43:FQ=1:SQ=-1;ID=S2666_raw:GT=hom:DP=27:FQ=1:SQ=-1;ID=S2669_raw:GT=hom:DP=31:FQ=1:SQ=-1;ID=S2166_raw:GT=hom:DP=19:FQ=1:SQ=-1;ID=S1775_raw:GT=hom:DP=14:FQ=1:SQ=-1;ID=S2501_raw:GT=hom:DP=27:FQ=1:SQ=-1;ID=S2500_raw:GT=hom:DP=21:FQ=1:SQ=-1;ID=S2502_raw:GT=hom:DP=23:FQ=1:SQ=-1;ID=S2505_raw:GT=hom:DP=27:FQ=1:SQ=-1;ID=S2176_raw:GT=hom:DP=26:FQ=1:SQ=-1;ID=S2506_raw:GT=hom:DP=25:FQ=1:SQ=-1;ID=S2667_raw:GT=hom:DP=27:FQ=1:SQ=-1;
18	19079853	19079853	A	G	exonic	GREB1L	synonymous SNV	GREB1L:NM_001142966:exon22:c.3555A>G:p.E1185E	rs4800747	.	ID=S2009_raw:GT=hom:DP=76:FQ=1:SQ=-1;ID=S2503_raw:GT=hom:DP=139:FQ=1:SQ=-1;ID=S2507_raw:GT=hom:DP=154:FQ=1:SQ=-1;ID=S2668_raw:GT=hom:DP=157:FQ=1:SQ=-1;ID=S2012_raw:GT=hom:DP=69:FQ=1:SQ=-1;ID=S2010_raw:GT=hom:DP=64:FQ=1:SQ=-1;ID=S2662_raw:GT=hom:DP=213:FQ=1:SQ=-1;ID=S2663_raw:GT=hom:DP=130:FQ=1:SQ=-1;ID=S2011_raw:GT=hom:DP=116:FQ=1:SQ=-1;ID=S2504_raw:GT=hom:DP=192:FQ=1:SQ=-1;ID=S2008_raw:GT=hom:DP=90:FQ=1:SQ=-1;ID=S2509_raw:GT=hom:DP=230:FQ=1:SQ=-1;ID=S2508_raw:GT=hom:DP=195:FQ=1:SQ=-1;ID=S2666_raw:GT=hom:DP=113:FQ=1:SQ=-1;ID=S2669_raw:GT=hom:DP=162:FQ=1:SQ=-1;ID=S2166_raw:GT=hom:DP=161:FQ=0.99:SQ=-1;ID=S1775_raw:GT=hom:DP=129:FQ=1:SQ=-1;ID=S2501_raw:GT=hom:DP=129:FQ=1:SQ=-1;ID=S2500_raw:GT=hom:DP=148:FQ=1:SQ=-1;ID=S2502_raw:GT=hom:DP=166:FQ=1:SQ=-1;ID=S2505_raw:GT=hom:DP=217:FQ=1:SQ=-1;ID=S2176_raw:GT=hom:DP=141:FQ=1:SQ=-1;ID=S2506_raw:GT=hom:DP=158:FQ=1:SQ=-1;ID=S2667_raw:GT=hom:DP=169:FQ=1:SQ=-1;
18	19100517	19100517	T	C	intronic	GREB1L	.	.	rs1395251	.	ID=S2009_raw:GT=hom:DP=33:FQ=1:SQ=-1;ID=S2503_raw:GT=hom:DP=43:FQ=1:SQ=-1;ID=S2507_raw:GT=hom:DP=27:FQ=1:SQ=-1;ID=S2668_raw:GT=hom:DP=30:FQ=1:SQ=-1;ID=S2012_raw:GT=hom:DP=24:FQ=1:SQ=-1;ID=S2010_raw:GT=hom:DP=35:FQ=1:SQ=-1;ID=S2662_raw:GT=hom:DP=4:FQ=1:SQ=-1;ID=S2663_raw:GT=hom:DP=28:FQ=1:SQ=-1;ID=S2011_raw:GT=hom:DP=37:FQ=1:SQ=-1;ID=S2504_raw:GT=hom:DP=33:FQ=1:SQ=-1;ID=S2008_raw:GT=hom:DP=40:FQ=1:SQ=-1;ID=S2509_raw:GT=hom:DP=21:FQ=1:SQ=-1;ID=S2508_raw:GT=hom:DP=20:FQ=1:SQ=-1;ID=S2666_raw:GT=hom:DP=26:FQ=1:SQ=-1;ID=S2669_raw:GT=hom:DP=18:FQ=1:SQ=-1;ID=S2166_raw:GT=hom:DP=104:FQ=1:SQ=-1;ID=S1775_raw:GT=hom:DP=39:FQ=1:SQ=-1;ID=S2501_raw:GT=hom:DP=26:FQ=1:SQ=-1;ID=S2500_raw:GT=hom:DP=34:FQ=1:SQ=-1;ID=S2502_raw:GT=hom:DP=33:FQ=1:SQ=-1;ID=S2505_raw:GT=hom:DP=40:FQ=1:SQ=-1;ID=S2176_raw:GT=hom:DP=99:FQ=1:SQ=-1;ID=S2506_raw:GT=hom:DP=23:FQ=1:SQ=-1;ID=S2667_raw:GT=hom:DP=29:FQ=1:SQ=-1;
18	19100762	19100762	-	CTT	exonic	GREB1L	nonframeshift insertion	GREB1L:NM_001142966:exon32:c.5586_5587insCTT:p.L1862delinsLL	.	.	ID=S2009_raw:GT=hom:DP=31:FQ=1:SQ=-1;ID=S2503_raw:GT=hom:DP=56:FQ=1:SQ=-1;ID=S2507_raw:GT=hom:DP=53:FQ=1:SQ=-1;ID=S2668_raw:GT=hom:DP=58:FQ=1:SQ=-1;ID=S2012_raw:GT=hom:DP=35:FQ=1:SQ=-1;ID=S2010_raw:GT=hom:DP=38:FQ=1:SQ=-1;ID=S2662_raw:GT=hom:DP=8:FQ=1:SQ=-1;ID=S2663_raw:GT=hom:DP=59:FQ=1:SQ=-1;ID=S2011_raw:GT=hom:DP=63:FQ=1:SQ=-1;ID=S2504_raw:GT=hom:DP=70:FQ=1:SQ=-1;ID=S2008_raw:GT=hom:DP=49:FQ=1:SQ=-1;ID=S2509_raw:GT=hom:DP=45:FQ=1:SQ=-1;ID=S2508_raw:GT=hom:DP=55:FQ=1:SQ=-1;ID=S2666_raw:GT=hom:DP=48:FQ=1:SQ=-1;ID=S2669_raw:GT=hom:DP=37:FQ=1:SQ=-1;ID=S2166_raw:GT=hom:DP=112:FQ=1:SQ=-1;ID=S1775_raw:GT=hom:DP=65:FQ=1:SQ=-1;ID=S2501_raw:GT=hom:DP=68:FQ=1:SQ=-1;ID=S2500_raw:GT=hom:DP=54:FQ=1:SQ=-1;ID=S2502_raw:GT=hom:DP=59:FQ=1:SQ=-1;ID=S2505_raw:GT=hom:DP=70:FQ=1:SQ=-1;ID=S2176_raw:GT=hom:DP=118:FQ=1:SQ=-1;ID=S2506_raw:GT=hom:DP=46:FQ=1:SQ=-1;ID=S2667_raw:GT=hom:DP=64:FQ=1:SQ=-1;
9	37426524	37426524	C	T	intronic	GRHPR	.	.	rs2736664	Benign	ID=S2009_raw:GT=hom:DP=164:FQ=1:SQ=-1;ID=S2503_raw:GT=hom:DP=245:FQ=1:SQ=-1;ID=S2507_raw:GT=hom:DP=215:FQ=1:SQ=-1;ID=S2668_raw:GT=hom:DP=273:FQ=1:SQ=-1;ID=S2012_raw:GT=hom:DP=192:FQ=1:SQ=-1;ID=S2010_raw:GT=hom:DP=199:FQ=1:SQ=-1;ID=S2662_raw:GT=hom:DP=249:FQ=1:SQ=-1;ID=S2663_raw:GT=hom:DP=209:FQ=1:SQ=-1;ID=S2011_raw:GT=hom:DP=261:FQ=1:SQ=-1;ID=S2504_raw:GT=hom:DP=199:FQ=1:SQ=-1;ID=S2008_raw:GT=hom:DP=211:FQ=1:SQ=-1;ID=S2509_raw:GT=hom:DP=227:FQ=1:SQ=-1;ID=S2508_raw:GT=hom:DP=226:FQ=1:SQ=-1;ID=S2666_raw:GT=hom:DP=288:FQ=1:SQ=-1;ID=S2669_raw:GT=hom:DP=216:FQ=1:SQ=-1;ID=S2166_raw:GT=hom:DP=168:FQ=1:SQ=-1;ID=S1775_raw:GT=hom:DP=139:FQ=1:SQ=-1;ID=S2501_raw:GT=hom:DP=213:FQ=1:SQ=-1;ID=S2500_raw:GT=hom:DP=192:FQ=0.99:SQ=-1;ID=S2502_raw:GT=hom:DP=205:FQ=1:SQ=-1;ID=S2505_raw:GT=hom:DP=177:FQ=1:SQ=-1;ID=S2176_raw:GT=hom:DP=228:FQ=1:SQ=-1;ID=S2506_raw:GT=hom:DP=163:FQ=1:SQ=-1;ID=S2667_raw:GT=hom:DP=221:FQ=1:SQ=-1;
X	1,22E+08	1,22E+08	-	G	UTR5	GRIA3	.	NM_001256743:c.-2_-1insG;NM_000828:c.-2_-1insG;NM_007325:c.-2_-1insG	rs58044961	.	ID=S2009_raw:GT=hom:DP=18:FQ=1:SQ=-1;ID=S2503_raw:GT=hom:DP=55:FQ=1:SQ=-1;ID=S2507_raw:GT=hom:DP=44:FQ=1:SQ=-1;ID=S2668_raw:GT=hom:DP=96:FQ=1:SQ=-1;ID=S2012_raw:GT=hom:DP=26:FQ=1:SQ=-1;ID=S2010_raw:GT=hom:DP=60:FQ=1:SQ=-1;ID=S2662_raw:GT=hom:DP=158:FQ=1:SQ=-1;ID=S2663_raw:GT=hom:DP=113:FQ=1:SQ=-1;ID=S2011_raw:GT=hom:DP=30:FQ=1:SQ=-1;ID=S2504_raw:GT=hom:DP=59:FQ=1:SQ=-1;ID=S2008_raw:GT=hom:DP=79:FQ=1:SQ=-1;ID=S2509_raw:GT=hom:DP=60:FQ=1:SQ=-1;ID=S2508_raw:GT=hom:DP=68:FQ=1:SQ=-1;ID=S2666_raw:GT=hom:DP=33:FQ=1:SQ=-1;ID=S2669_raw:GT=hom:DP=63:FQ=1:SQ=-1;ID=S2166_raw:GT=hom:DP=78:FQ=1:SQ=-1;ID=S1775_raw:GT=hom:DP=57:FQ=1:SQ=-1;ID=S2501_raw:GT=hom:DP=61:FQ=1:SQ=-1;ID=S2500_raw:GT=hom:DP=69:FQ=1:SQ=-1;ID=S2502_raw:GT=hom:DP=100:FQ=1:SQ=-1;ID=S2505_raw:GT=hom:DP=62:FQ=1:SQ=-1;ID=S2176_raw:GT=hom:DP=83:FQ=1:SQ=-1;ID=S2506_raw:GT=hom:DP=68:FQ=1:SQ=-1;ID=S2667_raw:GT=hom:DP=122:FQ=1:SQ=-1;
X	1,22E+08	1,22E+08	-	G	exonic;splicing	GRIA3	frameshift insertion	GRIA3:NM_001256743:exon4:c.384dupG:p.G128fs	.	.	ID=S2009_raw:GT=hom:DP=14:FQ=1:SQ=-1;ID=S2503_raw:GT=hom:DP=32:FQ=1:SQ=-1;ID=S2507_raw:GT=hom:DP=47:FQ=1:SQ=-1;ID=S2668_raw:GT=hom:DP=119:FQ=1:SQ=-1;ID=S2012_raw:GT=hom:DP=20:FQ=1:SQ=-1;ID=S2010_raw:GT=hom:DP=49:FQ=1:SQ=-1;ID=S2662_raw:GT=hom:DP=51:FQ=1:SQ=-1;ID=S2663_raw:GT=hom:DP=93:FQ=1:SQ=-1;ID=S2011_raw:GT=hom:DP=22:FQ=1:SQ=-1;ID=S2504_raw:GT=hom:DP=40:FQ=1:SQ=-1;ID=S2008_raw:GT=hom:DP=57:FQ=1:SQ=-1;ID=S2509_raw:GT=hom:DP=50:FQ=1:SQ=-1;ID=S2508_raw:GT=hom:DP=52:FQ=1:SQ=-1;ID=S2666_raw:GT=hom:DP=50:FQ=1:SQ=-1;ID=S2669_raw:GT=hom:DP=52:FQ=1:SQ=-1;ID=S2166_raw:GT=hom:DP=41:FQ=1:SQ=-1;ID=S1775_raw:GT=hom:DP=35:FQ=1:SQ=-1;ID=S2501_raw:GT=hom:DP=39:FQ=1:SQ=-1;ID=S2500_raw:GT=hom:DP=73:FQ=1:SQ=-1;ID=S2502_raw:GT=hom:DP=82:FQ=1:SQ=-1;ID=S2505_raw:GT=hom:DP=63:FQ=1:SQ=-1;ID=S2176_raw:GT=hom:DP=26:FQ=1:SQ=-1;ID=S2506_raw:GT=hom:DP=37:FQ=1:SQ=-1;ID=S2667_raw:GT=hom:DP=88:FQ=1:SQ=-1;
12	66786073	66786073	A	G	intronic	GRIP1	.	.	rs7970076	Benign	ID=S2009_raw:GT=hom:DP=5:FQ=1:SQ=-1;ID=S2503_raw:GT=hom:DP=107:FQ=1:SQ=-1;ID=S2507_raw:GT=hom:DP=92:FQ=1:SQ=-1;ID=S2668_raw:GT=hom:DP=161:FQ=1:SQ=-1;ID=S2012_raw:GT=hom:DP=7:FQ=1:SQ=-1;ID=S2010_raw:GT=hom:DP=6:FQ=1:SQ=-1;ID=S2662_raw:GT=hom:DP=24:FQ=1:SQ=-1;ID=S2663_raw:GT=hom:DP=101:FQ=1:SQ=-1;ID=S2011_raw:GT=hom:DP=9:FQ=1:SQ=-1;ID=S2504_raw:GT=hom:DP=129:FQ=1:SQ=-1;ID=S2008_raw:GT=hom:DP=8:FQ=1:SQ=-1;ID=S2509_raw:GT=hom:DP=90:FQ=1:SQ=-1;ID=S2508_raw:GT=hom:DP=94:FQ=1:SQ=-1;ID=S2666_raw:GT=hom:DP=155:FQ=1:SQ=-1;ID=S2669_raw:GT=hom:DP=95:FQ=1:SQ=-1;ID=S2166_raw:GT=hom:DP=107:FQ=1:SQ=-1;ID=S1775_raw:GT=hom:DP=73:FQ=1:SQ=-1;ID=S2501_raw:GT=hom:DP=84:FQ=1:SQ=-1;ID=S2500_raw:GT=hom:DP=96:FQ=1:SQ=-1;ID=S2502_raw:GT=hom:DP=120:FQ=1:SQ=-1;ID=S2505_raw:GT=hom:DP=106:FQ=1:SQ=-1;ID=S2176_raw:GT=hom:DP=108:FQ=1:SQ=-1;ID=S2506_raw:GT=hom:DP=46:FQ=1:SQ=-1;ID=S2667_raw:GT=hom:DP=83:FQ=1:SQ=-1;
12	66786341	66786341	G	A	intronic	GRIP1	.	.	rs7970771	.	ID=S2009_raw:GT=hom:DP=45:FQ=1:SQ=-1;ID=S2503_raw:GT=hom:DP=40:FQ=1:SQ=-1;ID=S2507_raw:GT=hom:DP=23:FQ=1:SQ=-1;ID=S2668_raw:GT=hom:DP=26:FQ=1:SQ=-1;ID=S2012_raw:GT=hom:DP=68:FQ=1:SQ=-1;ID=S2010_raw:GT=hom:DP=69:FQ=1:SQ=-1;ID=S2662_raw:GT=hom:DP=4:FQ=1:SQ=-1;ID=S2663_raw:GT=hom:DP=39:FQ=1:SQ=-1;ID=S2011_raw:GT=hom:DP=89:FQ=1:SQ=-1;ID=S2504_raw:GT=hom:DP=33:FQ=1:SQ=-1;ID=S2008_raw:GT=hom:DP=54:FQ=1:SQ=-1;ID=S2509_raw:GT=hom:DP=35:FQ=1:SQ=-1;ID=S2508_raw:GT=hom:DP=40:FQ=1:SQ=-1;ID=S2666_raw:GT=hom:DP=32:FQ=1:SQ=-1;ID=S2669_raw:GT=hom:DP=37:FQ=1:SQ=-1;ID=S2166_raw:GT=hom:DP=41:FQ=1:SQ=-1;ID=S1775_raw:GT=hom:DP=36:FQ=1:SQ=-1;ID=S2501_raw:GT=hom:DP=28:FQ=1:SQ=-1;ID=S2500_raw:GT=hom:DP=33:FQ=1:SQ=-1;ID=S2502_raw:GT=hom:DP=33:FQ=1:SQ=-1;ID=S2505_raw:GT=hom:DP=47:FQ=1:SQ=-1;ID=S2176_raw:GT=hom:DP=46:FQ=1:SQ=-1;ID=S2506_raw:GT=hom:DP=39:FQ=1:SQ=-1;ID=S2667_raw:GT=hom:DP=31:FQ=1:SQ=-1;
3	7302823	7302823	C	G	intronic	GRM7	.	.	rs4470504	.	ID=S2009_raw:GT=hom:DP=5:FQ=1:SQ=-1;ID=S2503_raw:GT=hom:DP=30:FQ=1:SQ=-1;ID=S2507_raw:GT=hom:DP=28:FQ=1:SQ=-1;ID=S2668_raw:GT=hom:DP=37:FQ=1:SQ=-1;ID=S2012_raw:GT=hom:DP=5:FQ=1:SQ=-1;ID=S2010_raw:GT=hom:DP=7:FQ=1:SQ=-1;ID=S2662_raw:GT=hom:DP=15:FQ=1:SQ=-1;ID=S2663_raw:GT=hom:DP=31:FQ=1:SQ=-1;ID=S2011_raw:GT=hom:DP=10:FQ=1:SQ=-1;ID=S2504_raw:GT=hom:DP=22:FQ=1:SQ=-1;ID=S2008_raw:GT=hom:DP=13:FQ=1:SQ=-1;ID=S2509_raw:GT=hom:DP=44:FQ=1:SQ=-1;ID=S2508_raw:GT=hom:DP=51:FQ=1:SQ=-1;ID=S2666_raw:GT=hom:DP=16:FQ=1:SQ=-1;ID=S2669_raw:GT=hom:DP=28:FQ=1:SQ=-1;ID=S2166_raw:GT=hom:DP=21:FQ=1:SQ=-1;ID=S1775_raw:GT=hom:DP=15:FQ=1:SQ=-1;ID=S2501_raw:GT=hom:DP=40:FQ=1:SQ=-1;ID=S2500_raw:GT=hom:DP=41:FQ=1:SQ=-1;ID=S2502_raw:GT=hom:DP=23:FQ=1:SQ=-1;ID=S2505_raw:GT=hom:DP=36:FQ=1:SQ=-1;ID=S2176_raw:GT=hom:DP=27:FQ=1:SQ=-1;ID=S2506_raw:GT=hom:DP=28:FQ=1:SQ=-1;ID=S2667_raw:GT=hom:DP=20:FQ=1:SQ=-1;
10	1,15E+08	1,15E+08	A	G	intronic	HABP2	.	.	rs911709	.	ID=S2009_raw:GT=hom:DP=24:FQ=1:SQ=-1;ID=S2503_raw:GT=hom:DP=104:FQ=1:SQ=-1;ID=S2507_raw:GT=hom:DP=83:FQ=1:SQ=-1;ID=S2668_raw:GT=hom:DP=120:FQ=1:SQ=-1;ID=S2012_raw:GT=hom:DP=26:FQ=1:SQ=-1;ID=S2010_raw:GT=hom:DP=31:FQ=1:SQ=-1;ID=S2662_raw:GT=hom:DP=48:FQ=1:SQ=-1;ID=S2663_raw:GT=hom:DP=69:FQ=1:SQ=-1;ID=S2011_raw:GT=hom:DP=32:FQ=1:SQ=-1;ID=S2504_raw:GT=hom:DP=85:FQ=1:SQ=-1;ID=S2008_raw:GT=hom:DP=18:FQ=1:SQ=-1;ID=S2509_raw:GT=hom:DP=100:FQ=1:SQ=-1;ID=S2508_raw:GT=hom:DP=102:FQ=1:SQ=-1;ID=S2666_raw:GT=hom:DP=111:FQ=1:SQ=-1;ID=S2669_raw:GT=hom:DP=78:FQ=1:SQ=-1;ID=S2166_raw:GT=hom:DP=47:FQ=1:SQ=-1;ID=S1775_raw:GT=hom:DP=47:FQ=1:SQ=-1;ID=S2501_raw:GT=hom:DP=97:FQ=1:SQ=-1;ID=S2500_raw:GT=hom:DP=86:FQ=1:SQ=-1;ID=S2502_raw:GT=hom:DP=92:FQ=1:SQ=-1;ID=S2505_raw:GT=hom:DP=101:FQ=1:SQ=-1;ID=S2176_raw:GT=hom:DP=68:FQ=1:SQ=-1;ID=S2506_raw:GT=hom:DP=70:FQ=1:SQ=-1;ID=S2667_raw:GT=hom:DP=108:FQ=1:SQ=-1;
2	2,4E+08	2,4E+08	-	C	intronic	HDAC4	.	.	.	.	ID=S2009_raw:GT=hom:DP=1:FQ=1:SQ=-1;ID=S2503_raw:GT=hom:DP=10:FQ=1:SQ=-1;ID=S2507_raw:GT=hom:DP=16:FQ=1:SQ=-1;ID=S2668_raw:GT=hom:DP=7:FQ=1:SQ=-1;ID=S2012_raw:GT=hom:DP=3:FQ=1:SQ=-1;ID=S2010_raw:GT=hom:DP=4:FQ=1:SQ=-1;ID=S2662_raw:GT=hom:DP=11:FQ=1:SQ=-1;ID=S2663_raw:GT=hom:DP=2:FQ=1:SQ=-1;ID=S2011_raw:GT=hom:DP=7:FQ=1:SQ=-1;ID=S2504_raw:GT=hom:DP=4:FQ=1:SQ=-1;ID=S2008_raw:GT=hom:DP=7:FQ=1:SQ=-1;ID=S2509_raw:GT=hom:DP=3:FQ=1:SQ=-1;ID=S2508_raw:GT=hom:DP=6:FQ=1:SQ=-1;ID=S2666_raw:GT=hom:DP=2:FQ=1:SQ=-1;ID=S2669_raw:GT=hom:DP=3:FQ=1:SQ=-1;ID=S2166_raw:GT=hom:DP=4:FQ=1:SQ=-1;ID=S1775_raw:GT=hom:DP=1:FQ=1:SQ=-1;ID=S2501_raw:GT=hom:DP=8:FQ=1:SQ=-1;ID=S2500_raw:GT=hom:DP=8:FQ=1:SQ=-1;ID=S2502_raw:GT=hom:DP=12:FQ=1:SQ=-1;ID=S2505_raw:GT=hom:DP=11:FQ=1:SQ=-1;ID=S2176_raw:GT=hom:DP=4:FQ=1:SQ=-1;ID=S2506_raw:GT=hom:DP=5:FQ=1:SQ=-1;ID=S2667_raw:GT=hom:DP=14:FQ=1:SQ=-1;
16	50102622	50102622	-	AAAT	intronic	HEATR3	.	.	.	.	ID=S2009_raw:GT=hom:DP=15:FQ=1:SQ=-1;ID=S2503_raw:GT=hom:DP=32:FQ=1:SQ=-1;ID=S2507_raw:GT=hom:DP=28:FQ=1:SQ=-1;ID=S2668_raw:GT=hom:DP=35:FQ=1:SQ=-1;ID=S2012_raw:GT=hom:DP=28:FQ=1:SQ=-1;ID=S2010_raw:GT=hom:DP=22:FQ=1:SQ=-1;ID=S2662_raw:GT=hom:DP=4:FQ=1:SQ=-1;ID=S2663_raw:GT=hom:DP=24:FQ=1:SQ=-1;ID=S2011_raw:GT=hom:DP=31:FQ=1:SQ=-1;ID=S2504_raw:GT=hom:DP=35:FQ=1:SQ=-1;ID=S2008_raw:GT=hom:DP=32:FQ=1:SQ=-1;ID=S2509_raw:GT=hom:DP=24:FQ=1:SQ=-1;ID=S2508_raw:GT=hom:DP=41:FQ=1:SQ=-1;ID=S2666_raw:GT=hom:DP=60:FQ=1:SQ=-1;ID=S2669_raw:GT=hom:DP=25:FQ=1:SQ=-1;ID=S2166_raw:GT=hom:DP=105:FQ=1:SQ=-1;ID=S1775_raw:GT=hom:DP=89:FQ=1:SQ=-1;ID=S2501_raw:GT=hom:DP=32:FQ=1:SQ=-1;ID=S2500_raw:GT=hom:DP=38:FQ=1:SQ=-1;ID=S2502_raw:GT=hom:DP=39:FQ=1:SQ=-1;ID=S2505_raw:GT=hom:DP=42:FQ=1:SQ=-1;ID=S2176_raw:GT=hom:DP=122:FQ=1:SQ=-1;ID=S2506_raw:GT=hom:DP=23:FQ=1:SQ=-1;ID=S2667_raw:GT=hom:DP=31:FQ=1:SQ=-1;
12	1,21E+08	1,21E+08	A	G	exonic	HNF1A	nonsynonymous SNV	HNF1A:NM_000545:exon9:c.1720A>G:p.S574G,HNF1A:NM_001306179:exon9:c.1741A>G:p.S581G	rs1169305	Benign	ID=S2009_raw:GT=hom:DP=109:FQ=1:SQ=-1;ID=S2503_raw:GT=hom:DP=29:FQ=1:SQ=-1;ID=S2507_raw:GT=hom:DP=36:FQ=1:SQ=-1;ID=S2668_raw:GT=hom:DP=35:FQ=1:SQ=-1;ID=S2012_raw:GT=hom:DP=63:FQ=1:SQ=-1;ID=S2010_raw:GT=hom:DP=79:FQ=1:SQ=-1;ID=S2662_raw:GT=hom:DP=47:FQ=1:SQ=-1;ID=S2663_raw:GT=hom:DP=28:FQ=1:SQ=-1;ID=S2011_raw:GT=hom:DP=130:FQ=1:SQ=-1;ID=S2504_raw:GT=hom:DP=33:FQ=1:SQ=-1;ID=S2008_raw:GT=hom:DP=103:FQ=1:SQ=-1;ID=S2509_raw:GT=hom:DP=42:FQ=1:SQ=-1;ID=S2508_raw:GT=hom:DP=45:FQ=1:SQ=-1;ID=S2666_raw:GT=hom:DP=19:FQ=1:SQ=-1;ID=S2669_raw:GT=hom:DP=30:FQ=1:SQ=-1;ID=S2166_raw:GT=hom:DP=43:FQ=0.95:SQ=-1;ID=S1775_raw:GT=hom:DP=51:FQ=1:SQ=-1;ID=S2501_raw:GT=hom:DP=36:FQ=1:SQ=-1;ID=S2500_raw:GT=hom:DP=32:FQ=1:SQ=-1;ID=S2502_raw:GT=hom:DP=31:FQ=1:SQ=-1;ID=S2505_raw:GT=hom:DP=24:FQ=1:SQ=-1;ID=S2176_raw:GT=hom:DP=35:FQ=1:SQ=-1;ID=S2506_raw:GT=hom:DP=41:FQ=1:SQ=-1;ID=S2667_raw:GT=hom:DP=27:FQ=1:SQ=-1;
5	1,79E+08	1,79E+08	T	C	intronic	HNRNPH1	.	.	rs4515257	.	ID=S2009_raw:GT=hom:DP=19:FQ=1:SQ=-1;ID=S2503_raw:GT=hom:DP=111:FQ=1:SQ=-1;ID=S2507_raw:GT=hom:DP=133:FQ=1:SQ=-1;ID=S2668_raw:GT=hom:DP=111:FQ=1:SQ=-1;ID=S2012_raw:GT=hom:DP=17:FQ=1:SQ=-1;ID=S2010_raw:GT=hom:DP=31:FQ=1:SQ=-1;ID=S2662_raw:GT=hom:DP=58:FQ=1:SQ=-1;ID=S2663_raw:GT=hom:DP=96:FQ=1:SQ=-1;ID=S2011_raw:GT=hom:DP=24:FQ=1:SQ=-1;ID=S2504_raw:GT=hom:DP=160:FQ=1:SQ=-1;ID=S2008_raw:GT=hom:DP=31:FQ=1:SQ=-1;ID=S2509_raw:GT=hom:DP=131:FQ=1:SQ=-1;ID=S2508_raw:GT=hom:DP=139:FQ=1:SQ=-1;ID=S2666_raw:GT=hom:DP=135:FQ=1:SQ=-1;ID=S2669_raw:GT=hom:DP=120:FQ=1:SQ=-1;ID=S2166_raw:GT=hom:DP=166:FQ=1:SQ=-1;ID=S1775_raw:GT=hom:DP=124:FQ=1:SQ=-1;ID=S2501_raw:GT=hom:DP=120:FQ=1:SQ=-1;ID=S2500_raw:GT=hom:DP=124:FQ=1:SQ=-1;ID=S2502_raw:GT=hom:DP=155:FQ=1:SQ=-1;ID=S2505_raw:GT=hom:DP=138:FQ=1:SQ=-1;ID=S2176_raw:GT=hom:DP=208:FQ=1:SQ=-1;ID=S2506_raw:GT=hom:DP=85:FQ=1:SQ=-1;ID=S2667_raw:GT=hom:DP=122:FQ=1:SQ=-1;
5	1,79E+08	1,79E+08	C	A	intronic	HNRNPH1	.	.	rs4301193	.	ID=S2009_raw:GT=hom:DP=21:FQ=1:SQ=-1;ID=S2503_raw:GT=hom:DP=9:FQ=1:SQ=-1;ID=S2507_raw:GT=hom:DP=9:FQ=1:SQ=-1;ID=S2668_raw:GT=hom:DP=10:FQ=1:SQ=-1;ID=S2012_raw:GT=hom:DP=19:FQ=1:SQ=-1;ID=S2010_raw:GT=hom:DP=28:FQ=1:SQ=-1;ID=S2662_raw:GT=hom:DP=2:FQ=1:SQ=-1;ID=S2663_raw:GT=hom:DP=5:FQ=1:SQ=-1;ID=S2011_raw:GT=hom:DP=41:FQ=1:SQ=-1;ID=S2504_raw:GT=hom:DP=12:FQ=1:SQ=-1;ID=S2008_raw:GT=hom:DP=25:FQ=1:SQ=-1;ID=S2509_raw:GT=hom:DP=7:FQ=1:SQ=-1;ID=S2508_raw:GT=hom:DP=6:FQ=1:SQ=-1;ID=S2666_raw:GT=hom:DP=3:FQ=1:SQ=-1;ID=S2669_raw:GT=hom:DP=6:FQ=1:SQ=-1;ID=S2166_raw:GT=hom:DP=17:FQ=1:SQ=-1;ID=S1775_raw:GT=hom:DP=3:FQ=1:SQ=-1;ID=S2501_raw:GT=hom:DP=7:FQ=1:SQ=-1;ID=S2500_raw:GT=hom:DP=17:FQ=1:SQ=-1;ID=S2502_raw:GT=hom:DP=6:FQ=1:SQ=-1;ID=S2505_raw:GT=hom:DP=11:FQ=1:SQ=-1;ID=S2176_raw:GT=hom:DP=29:FQ=1:SQ=-1;ID=S2506_raw:GT=hom:DP=6:FQ=1:SQ=-1;ID=S2667_raw:GT=hom:DP=12:FQ=1:SQ=-1;
12	57234766	57234766	-	C	intergenic	HSD17B6;SDR9C7	.	dist=53192;dist=82151	rs5798397	.	ID=S2009_raw:GT=hom:DP=3:FQ=1:SQ=-1;ID=S2503_raw:GT=hom:DP=11:FQ=1:SQ=-1;ID=S2507_raw:GT=hom:DP=9:FQ=1:SQ=-1;ID=S2668_raw:GT=hom:DP=12:FQ=1:SQ=-1;ID=S2012_raw:GT=hom:DP=8:FQ=1:SQ=-1;ID=S2010_raw:GT=hom:DP=10:FQ=1:SQ=-1;ID=S2662_raw:GT=hom:DP=3:FQ=1:SQ=-1;ID=S2663_raw:GT=hom:DP=7:FQ=1:SQ=-1;ID=S2011_raw:GT=hom:DP=14:FQ=1:SQ=-1;ID=S2504_raw:GT=hom:DP=13:FQ=1:SQ=-1;ID=S2008_raw:GT=hom:DP=8:FQ=1:SQ=-1;ID=S2509_raw:GT=hom:DP=8:FQ=1:SQ=-1;ID=S2508_raw:GT=hom:DP=15:FQ=1:SQ=-1;ID=S2666_raw:GT=hom:DP=18:FQ=1:SQ=-1;ID=S2669_raw:GT=hom:DP=16:FQ=1:SQ=-1;ID=S2166_raw:GT=hom:DP=27:FQ=1:SQ=-1;ID=S1775_raw:GT=hom:DP=5:FQ=1:SQ=-1;ID=S2501_raw:GT=hom:DP=13:FQ=1:SQ=-1;ID=S2500_raw:GT=hom:DP=14:FQ=1:SQ=-1;ID=S2502_raw:GT=hom:DP=15:FQ=1:SQ=-1;ID=S2505_raw:GT=hom:DP=11:FQ=1:SQ=-1;ID=S2176_raw:GT=hom:DP=37:FQ=1:SQ=-1;ID=S2506_raw:GT=hom:DP=11:FQ=1:SQ=-1;ID=S2667_raw:GT=hom:DP=12:FQ=1:SQ=-1;
1	22199081	22199081	A	T	intronic	HSPG2	.	.	rs2501266	.	ID=S2009_raw:GT=hom:DP=3:FQ=1:SQ=-1;ID=S2503_raw:GT=hom:DP=64:FQ=1:SQ=-1;ID=S2507_raw:GT=hom:DP=83:FQ=1:SQ=-1;ID=S2668_raw:GT=hom:DP=65:FQ=1:SQ=-1;ID=S2012_raw:GT=hom:DP=7:FQ=1:SQ=-1;ID=S2010_raw:GT=hom:DP=11:FQ=1:SQ=-1;ID=S2662_raw:GT=hom:DP=111:FQ=1:SQ=-1;ID=S2663_raw:GT=hom:DP=73:FQ=1:SQ=-1;ID=S2011_raw:GT=hom:DP=10:FQ=1:SQ=-1;ID=S2504_raw:GT=hom:DP=70:FQ=1:SQ=-1;ID=S2008_raw:GT=hom:DP=4:FQ=1:SQ=-1;ID=S2509_raw:GT=hom:DP=59:FQ=1:SQ=-1;ID=S2508_raw:GT=hom:DP=87:FQ=1:SQ=-1;ID=S2666_raw:GT=hom:DP=87:FQ=1:SQ=-1;ID=S2669_raw:GT=hom:DP=73:FQ=1:SQ=-1;ID=S2166_raw:GT=hom:DP=20:FQ=1:SQ=-1;ID=S1775_raw:GT=hom:DP=11:FQ=1:SQ=-1;ID=S2501_raw:GT=hom:DP=61:FQ=1:SQ=-1;ID=S2500_raw:GT=hom:DP=62:FQ=1:SQ=-1;ID=S2502_raw:GT=hom:DP=76:FQ=1:SQ=-1;ID=S2505_raw:GT=hom:DP=62:FQ=1:SQ=-1;ID=S2176_raw:GT=hom:DP=22:FQ=1:SQ=-1;ID=S2506_raw:GT=hom:DP=58:FQ=1:SQ=-1;ID=S2667_raw:GT=hom:DP=66:FQ=1:SQ=-1;
1	22200082	22200082	T	C	intronic	HSPG2	.	.	rs2445136	.	ID=S2009_raw:GT=hom:DP=21:FQ=1:SQ=-1;ID=S2503_raw:GT=hom:DP=55:FQ=1:SQ=-1;ID=S2507_raw:GT=hom:DP=56:FQ=1:SQ=-1;ID=S2668_raw:GT=hom:DP=58:FQ=0.98:SQ=-1;ID=S2012_raw:GT=hom:DP=34:FQ=1:SQ=-1;ID=S2010_raw:GT=hom:DP=31:FQ=1:SQ=-1;ID=S2662_raw:GT=hom:DP=111:FQ=1:SQ=-1;ID=S2663_raw:GT=hom:DP=61:FQ=1:SQ=-1;ID=S2011_raw:GT=hom:DP=46:FQ=1:SQ=-1;ID=S2504_raw:GT=hom:DP=55:FQ=1:SQ=-1;ID=S2008_raw:GT=hom:DP=39:FQ=1:SQ=-1;ID=S2509_raw:GT=hom:DP=76:FQ=1:SQ=-1;ID=S2508_raw:GT=hom:DP=76:FQ=1:SQ=-1;ID=S2666_raw:GT=hom:DP=24:FQ=1:SQ=-1;ID=S2669_raw:GT=hom:DP=57:FQ=1:SQ=-1;ID=S2166_raw:GT=hom:DP=43:FQ=1:SQ=-1;ID=S1775_raw:GT=hom:DP=24:FQ=1:SQ=-1;ID=S2501_raw:GT=hom:DP=57:FQ=1:SQ=-1;ID=S2500_raw:GT=hom:DP=64:FQ=1:SQ=-1;ID=S2502_raw:GT=hom:DP=43:FQ=1:SQ=-1;ID=S2505_raw:GT=hom:DP=55:FQ=1:SQ=-1;ID=S2176_raw:GT=hom:DP=33:FQ=1:SQ=-1;ID=S2506_raw:GT=hom:DP=57:FQ=1:SQ=-1;ID=S2667_raw:GT=hom:DP=73:FQ=1:SQ=-1;
1	22202109	22202109	C	T	intronic	HSPG2	.	.	rs2501265	Benign	ID=S2009_raw:GT=hom:DP=19:FQ=1:SQ=-1;ID=S2503_raw:GT=hom:DP=51:FQ=1:SQ=-1;ID=S2507_raw:GT=hom:DP=51:FQ=1:SQ=-1;ID=S2668_raw:GT=hom:DP=86:FQ=1:SQ=-1;ID=S2012_raw:GT=hom:DP=29:FQ=1:SQ=-1;ID=S2010_raw:GT=hom:DP=30:FQ=1:SQ=-1;ID=S2662_raw:GT=hom:DP=135:FQ=1:SQ=-1;ID=S2663_raw:GT=hom:DP=67:FQ=1:SQ=-1;ID=S2011_raw:GT=hom:DP=28:FQ=1:SQ=-1;ID=S2504_raw:GT=hom:DP=70:FQ=1:SQ=-1;ID=S2008_raw:GT=hom:DP=31:FQ=1:SQ=-1;ID=S2509_raw:GT=hom:DP=51:FQ=1:SQ=-1;ID=S2508_raw:GT=hom:DP=71:FQ=1:SQ=-1;ID=S2666_raw:GT=hom:DP=56:FQ=1:SQ=-1;ID=S2669_raw:GT=hom:DP=73:FQ=1:SQ=-1;ID=S2166_raw:GT=hom:DP=47:FQ=1:SQ=-1;ID=S1775_raw:GT=hom:DP=33:FQ=1:SQ=-1;ID=S2501_raw:GT=hom:DP=59:FQ=1:SQ=-1;ID=S2500_raw:GT=hom:DP=53:FQ=1:SQ=-1;ID=S2502_raw:GT=hom:DP=75:FQ=1:SQ=-1;ID=S2505_raw:GT=hom:DP=72:FQ=1:SQ=-1;ID=S2176_raw:GT=hom:DP=62:FQ=1:SQ=-1;ID=S2506_raw:GT=hom:DP=46:FQ=1:SQ=-1;ID=S2667_raw:GT=hom:DP=63:FQ=1:SQ=-1;
1	22205408	22205408	T	C	intronic	HSPG2	.	.	rs2454294	.	ID=S2009_raw:GT=hom:DP=8:FQ=1:SQ=-1;ID=S2503_raw:GT=hom:DP=15:FQ=1:SQ=-1;ID=S2507_raw:GT=hom:DP=10:FQ=1:SQ=-1;ID=S2668_raw:GT=hom:DP=11:FQ=1:SQ=-1;ID=S2012_raw:GT=hom:DP=9:FQ=1:SQ=-1;ID=S2010_raw:GT=hom:DP=18:FQ=1:SQ=-1;ID=S2662_raw:GT=hom:DP=15:FQ=1:SQ=-1;ID=S2663_raw:GT=hom:DP=20:FQ=1:SQ=-1;ID=S2011_raw:GT=hom:DP=13:FQ=1:SQ=-1;ID=S2504_raw:GT=hom:DP=16:FQ=1:SQ=-1;ID=S2008_raw:GT=hom:DP=9:FQ=1:SQ=-1;ID=S2509_raw:GT=hom:DP=8:FQ=1:SQ=-1;ID=S2508_raw:GT=hom:DP=21:FQ=1:SQ=-1;ID=S2666_raw:GT=hom:DP=15:FQ=1:SQ=-1;ID=S2669_raw:GT=hom:DP=15:FQ=1:SQ=-1;ID=S2166_raw:GT=hom:DP=8:FQ=1:SQ=-1;ID=S1775_raw:GT=hom:DP=10:FQ=1:SQ=-1;ID=S2501_raw:GT=hom:DP=12:FQ=1:SQ=-1;ID=S2500_raw:GT=hom:DP=17:FQ=1:SQ=-1;ID=S2502_raw:GT=hom:DP=18:FQ=1:SQ=-1;ID=S2505_raw:GT=hom:DP=10:FQ=1:SQ=-1;ID=S2176_raw:GT=hom:DP=16:FQ=1:SQ=-1;ID=S2506_raw:GT=hom:DP=17:FQ=1:SQ=-1;ID=S2667_raw:GT=hom:DP=14:FQ=1:SQ=-1;
1	22205466	22205466	T	C	intronic	HSPG2	.	.	rs2454293	.	ID=S2009_raw:GT=hom:DP=23:FQ=1:SQ=-1;ID=S2503_raw:GT=hom:DP=26:FQ=1:SQ=-1;ID=S2507_raw:GT=hom:DP=25:FQ=1:SQ=-1;ID=S2668_raw:GT=hom:DP=27:FQ=1:SQ=-1;ID=S2012_raw:GT=hom:DP=28:FQ=1:SQ=-1;ID=S2010_raw:GT=hom:DP=31:FQ=1:SQ=-1;ID=S2662_raw:GT=hom:DP=53:FQ=1:SQ=-1;ID=S2663_raw:GT=hom:DP=24:FQ=1:SQ=-1;ID=S2011_raw:GT=hom:DP=30:FQ=1:SQ=-1;ID=S2504_raw:GT=hom:DP=34:FQ=1:SQ=-1;ID=S2008_raw:GT=hom:DP=20:FQ=1:SQ=-1;ID=S2509_raw:GT=hom:DP=30:FQ=1:SQ=-1;ID=S2508_raw:GT=hom:DP=42:FQ=1:SQ=-1;ID=S2666_raw:GT=hom:DP=30:FQ=1:SQ=-1;ID=S2669_raw:GT=hom:DP=23:FQ=1:SQ=-1;ID=S2166_raw:GT=hom:DP=19:FQ=1:SQ=-1;ID=S1775_raw:GT=hom:DP=29:FQ=1:SQ=-1;ID=S2501_raw:GT=hom:DP=25:FQ=1:SQ=-1;ID=S2500_raw:GT=hom:DP=37:FQ=1:SQ=-1;ID=S2502_raw:GT=hom:DP=39:FQ=1:SQ=-1;ID=S2505_raw:GT=hom:DP=20:FQ=1:SQ=-1;ID=S2176_raw:GT=hom:DP=28:FQ=1:SQ=-1;ID=S2506_raw:GT=hom:DP=25:FQ=1:SQ=-1;ID=S2667_raw:GT=hom:DP=38:FQ=1:SQ=-1;
1	22206649	22206649	T	C	exonic	HSPG2	nonsynonymous SNV	HSPG2:NM_001291860:exon17:c.2297A>G:p.N766S,HSPG2:NM_005529:exon17:c.2294A>G:p.N765S	rs989994	Benign	ID=S2009_raw:GT=hom:DP=54:FQ=1:SQ=-1;ID=S2503_raw:GT=hom:DP=116:FQ=1:SQ=-1;ID=S2507_raw:GT=hom:DP=139:FQ=1:SQ=-1;ID=S2668_raw:GT=hom:DP=149:FQ=1:SQ=-1;ID=S2012_raw:GT=hom:DP=71:FQ=1:SQ=-1;ID=S2010_raw:GT=hom:DP=76:FQ=1:SQ=-1;ID=S2662_raw:GT=hom:DP=243:FQ=1:SQ=-1;ID=S2663_raw:GT=hom:DP=134:FQ=1:SQ=-1;ID=S2011_raw:GT=hom:DP=84:FQ=1:SQ=-1;ID=S2504_raw:GT=hom:DP=145:FQ=1:SQ=-1;ID=S2008_raw:GT=hom:DP=87:FQ=1:SQ=-1;ID=S2509_raw:GT=hom:DP=137:FQ=1:SQ=-1;ID=S2508_raw:GT=hom:DP=128:FQ=1:SQ=-1;ID=S2666_raw:GT=hom:DP=112:FQ=1:SQ=-1;ID=S2669_raw:GT=hom:DP=119:FQ=1:SQ=-1;ID=S2166_raw:GT=hom:DP=89:FQ=1:SQ=-1;ID=S1775_raw:GT=hom:DP=76:FQ=1:SQ=-1;ID=S2501_raw:GT=hom:DP=120:FQ=1:SQ=-1;ID=S2500_raw:GT=hom:DP=116:FQ=1:SQ=-1;ID=S2502_raw:GT=hom:DP=161:FQ=1:SQ=-1;ID=S2505_raw:GT=hom:DP=114:FQ=1:SQ=-1;ID=S2176_raw:GT=hom:DP=87:FQ=1:SQ=-1;ID=S2506_raw:GT=hom:DP=142:FQ=1:SQ=-1;ID=S2667_raw:GT=hom:DP=145:FQ=1:SQ=-1;
1	22206942	22206942	G	A	exonic	HSPG2	synonymous SNV	HSPG2:NM_001291860:exon16:c.2112C>T:p.A704A,HSPG2:NM_005529:exon16:c.2109C>T:p.A703A	rs1874793	Benign	ID=S2009_raw:GT=hom:DP=107:FQ=1:SQ=-1;ID=S2503_raw:GT=hom:DP=201:FQ=1:SQ=-1;ID=S2507_raw:GT=hom:DP=168:FQ=1:SQ=-1;ID=S2668_raw:GT=hom:DP=194:FQ=1:SQ=-1;ID=S2012_raw:GT=hom:DP=120:FQ=1:SQ=-1;ID=S2010_raw:GT=hom:DP=108:FQ=1:SQ=-1;ID=S2662_raw:GT=hom:DP=352:FQ=1:SQ=-1;ID=S2663_raw:GT=hom:DP=176:FQ=0.99:SQ=-1;ID=S2011_raw:GT=hom:DP=174:FQ=1:SQ=-1;ID=S2504_raw:GT=hom:DP=213:FQ=1:SQ=-1;ID=S2008_raw:GT=hom:DP=125:FQ=1:SQ=-1;ID=S2509_raw:GT=hom:DP=182:FQ=1:SQ=-1;ID=S2508_raw:GT=hom:DP=197:FQ=1:SQ=-1;ID=S2666_raw:GT=hom:DP=178:FQ=1:SQ=-1;ID=S2669_raw:GT=hom:DP=146:FQ=1:SQ=-1;ID=S2166_raw:GT=hom:DP=161:FQ=1:SQ=-1;ID=S1775_raw:GT=hom:DP=108:FQ=1:SQ=-1;ID=S2501_raw:GT=hom:DP=198:FQ=1:SQ=-1;ID=S2500_raw:GT=hom:DP=180:FQ=0.99:SQ=-1;ID=S2502_raw:GT=hom:DP=208:FQ=1:SQ=-1;ID=S2505_raw:GT=hom:DP=204:FQ=1:SQ=-1;ID=S2176_raw:GT=hom:DP=153:FQ=1:SQ=-1;ID=S2506_raw:GT=hom:DP=176:FQ=1:SQ=-1;ID=S2667_raw:GT=hom:DP=192:FQ=1:SQ=-1;
1	22207235	22207235	T	C	exonic	HSPG2	nonsynonymous SNV	HSPG2:NM_001291860:exon15:c.1915A>G:p.M639V,HSPG2:NM_005529:exon15:c.1912A>G:p.M638V	rs1874792	Benign	ID=S2009_raw:GT=hom:DP=84:FQ=1:SQ=-1;ID=S2503_raw:GT=hom:DP=63:FQ=1:SQ=-1;ID=S2507_raw:GT=hom:DP=77:FQ=1:SQ=-1;ID=S2668_raw:GT=hom:DP=94:FQ=1:SQ=-1;ID=S2012_raw:GT=hom:DP=61:FQ=1:SQ=-1;ID=S2010_raw:GT=hom:DP=82:FQ=1:SQ=-1;ID=S2662_raw:GT=hom:DP=178:FQ=1:SQ=-1;ID=S2663_raw:GT=hom:DP=93:FQ=1:SQ=-1;ID=S2011_raw:GT=hom:DP=100:FQ=1:SQ=-1;ID=S2504_raw:GT=hom:DP=71:FQ=1:SQ=-1;ID=S2008_raw:GT=hom:DP=89:FQ=1:SQ=-1;ID=S2509_raw:GT=hom:DP=68:FQ=1:SQ=-1;ID=S2508_raw:GT=hom:DP=71:FQ=1:SQ=-1;ID=S2666_raw:GT=hom:DP=61:FQ=1:SQ=-1;ID=S2669_raw:GT=hom:DP=97:FQ=1:SQ=-1;ID=S2166_raw:GT=hom:DP=78:FQ=1:SQ=-1;ID=S1775_raw:GT=hom:DP=52:FQ=1:SQ=-1;ID=S2501_raw:GT=hom:DP=77:FQ=1:SQ=-1;ID=S2500_raw:GT=hom:DP=49:FQ=1:SQ=-1;ID=S2502_raw:GT=hom:DP=86:FQ=1:SQ=-1;ID=S2505_raw:GT=hom:DP=76:FQ=1:SQ=-1;ID=S2176_raw:GT=hom:DP=76:FQ=1:SQ=-1;ID=S2506_raw:GT=hom:DP=61:FQ=1:SQ=-1;ID=S2667_raw:GT=hom:DP=88:FQ=1:SQ=-1;
1	22207811	22207823	GTGGCAGCCCAGG	-	splicing	HSPG2	.	.	.	.	ID=S2009_raw:GT=hom:DP=74:FQ=1:SQ=-1;ID=S2503_raw:GT=hom:DP=45:FQ=1:SQ=-1;ID=S2507_raw:GT=hom:DP=39:FQ=1:SQ=-1;ID=S2668_raw:GT=hom:DP=34:FQ=1:SQ=-1;ID=S2012_raw:GT=hom:DP=74:FQ=1:SQ=-1;ID=S2010_raw:GT=hom:DP=77:FQ=1:SQ=-1;ID=S2662_raw:GT=hom:DP=45:FQ=1:SQ=-1;ID=S2663_raw:GT=hom:DP=33:FQ=1:SQ=-1;ID=S2011_raw:GT=hom:DP=113:FQ=1:SQ=-1;ID=S2504_raw:GT=hom:DP=33:FQ=1:SQ=-1;ID=S2008_raw:GT=hom:DP=58:FQ=1:SQ=-1;ID=S2509_raw:GT=hom:DP=23:FQ=1:SQ=-1;ID=S2508_raw:GT=hom:DP=31:FQ=1:SQ=-1;ID=S2666_raw:GT=hom:DP=23:FQ=1:SQ=-1;ID=S2669_raw:GT=hom:DP=39:FQ=1:SQ=-1;ID=S2166_raw:GT=hom:DP=58:FQ=1:SQ=-1;ID=S1775_raw:GT=hom:DP=39:FQ=1:SQ=-1;ID=S2501_raw:GT=hom:DP=33:FQ=1:SQ=-1;ID=S2500_raw:GT=hom:DP=46:FQ=1:SQ=-1;ID=S2502_raw:GT=hom:DP=44:FQ=1:SQ=-1;ID=S2505_raw:GT=hom:DP=38:FQ=1:SQ=-1;ID=S2176_raw:GT=hom:DP=47:FQ=1:SQ=-1;ID=S2506_raw:GT=hom:DP=49:FQ=1:SQ=-1;ID=S2667_raw:GT=hom:DP=62:FQ=1:SQ=-1;
1	22208030	22208030	C	T	intronic	HSPG2	.	.	rs921847	Benign	ID=S2009_raw:GT=hom:DP=15:FQ=1:SQ=-1;ID=S2503_raw:GT=hom:DP=33:FQ=1:SQ=-1;ID=S2507_raw:GT=hom:DP=11:FQ=1:SQ=-1;ID=S2668_raw:GT=hom:DP=35:FQ=1:SQ=-1;ID=S2012_raw:GT=hom:DP=20:FQ=1:SQ=-1;ID=S2010_raw:GT=hom:DP=13:FQ=1:SQ=-1;ID=S2662_raw:GT=hom:DP=59:FQ=1:SQ=-1;ID=S2663_raw:GT=hom:DP=39:FQ=1:SQ=-1;ID=S2011_raw:GT=hom:DP=11:FQ=1:SQ=-1;ID=S2504_raw:GT=hom:DP=23:FQ=1:SQ=-1;ID=S2008_raw:GT=hom:DP=14:FQ=1:SQ=-1;ID=S2509_raw:GT=hom:DP=26:FQ=1:SQ=-1;ID=S2508_raw:GT=hom:DP=19:FQ=1:SQ=-1;ID=S2666_raw:GT=hom:DP=29:FQ=1:SQ=-1;ID=S2669_raw:GT=hom:DP=27:FQ=1:SQ=-1;ID=S2166_raw:GT=hom:DP=18:FQ=1:SQ=-1;ID=S1775_raw:GT=hom:DP=4:FQ=1:SQ=-1;ID=S2501_raw:GT=hom:DP=24:FQ=1:SQ=-1;ID=S2500_raw:GT=hom:DP=31:FQ=1:SQ=-1;ID=S2502_raw:GT=hom:DP=35:FQ=1:SQ=-1;ID=S2505_raw:GT=hom:DP=31:FQ=1:SQ=-1;ID=S2176_raw:GT=hom:DP=20:FQ=1:SQ=-1;ID=S2506_raw:GT=hom:DP=34:FQ=1:SQ=-1;ID=S2667_raw:GT=hom:DP=39:FQ=1:SQ=-1;
1	22215088	22215088	T	C	intronic	HSPG2	.	.	rs2445140	.	ID=S2009_raw:GT=hom:DP=34:FQ=1:SQ=-1;ID=S2503_raw:GT=hom:DP=117:FQ=1:SQ=-1;ID=S2507_raw:GT=hom:DP=89:FQ=1:SQ=-1;ID=S2668_raw:GT=hom:DP=89:FQ=1:SQ=-1;ID=S2012_raw:GT=hom:DP=36:FQ=1:SQ=-1;ID=S2010_raw:GT=hom:DP=28:FQ=1:SQ=-1;ID=S2662_raw:GT=hom:DP=201:FQ=1:SQ=-1;ID=S2663_raw:GT=hom:DP=110:FQ=0.98:SQ=-1;ID=S2011_raw:GT=hom:DP=43:FQ=1:SQ=-1;ID=S2504_raw:GT=hom:DP=98:FQ=1:SQ=-1;ID=S2008_raw:GT=hom:DP=51:FQ=1:SQ=-1;ID=S2509_raw:GT=hom:DP=128:FQ=1:SQ=-1;ID=S2508_raw:GT=hom:DP=123:FQ=1:SQ=-1;ID=S2666_raw:GT=hom:DP=64:FQ=1:SQ=-1;ID=S2669_raw:GT=hom:DP=143:FQ=1:SQ=-1;ID=S2166_raw:GT=hom:DP=70:FQ=1:SQ=-1;ID=S1775_raw:GT=hom:DP=43:FQ=1:SQ=-1;ID=S2501_raw:GT=hom:DP=98:FQ=1:SQ=-1;ID=S2500_raw:GT=hom:DP=110:FQ=1:SQ=-1;ID=S2502_raw:GT=hom:DP=116:FQ=1:SQ=-1;ID=S2505_raw:GT=hom:DP=107:FQ=1:SQ=-1;ID=S2176_raw:GT=hom:DP=39:FQ=1:SQ=-1;ID=S2506_raw:GT=hom:DP=139:FQ=1:SQ=-1;ID=S2667_raw:GT=hom:DP=146:FQ=1:SQ=-1;
1	22217108	22217108	G	A	exonic	HSPG2	synonymous SNV	HSPG2:NM_001291860:exon4:c.324C>T:p.F108F,HSPG2:NM_005529:exon4:c.324C>T:p.F108F	rs2501260	Benign	ID=S2009_raw:GT=hom:DP=109:FQ=1:SQ=-1;ID=S2503_raw:GT=hom:DP=176:FQ=1:SQ=-1;ID=S2507_raw:GT=hom:DP=170:FQ=1:SQ=-1;ID=S2668_raw:GT=hom:DP=168:FQ=1:SQ=-1;ID=S2012_raw:GT=hom:DP=131:FQ=1:SQ=-1;ID=S2010_raw:GT=hom:DP=116:FQ=1:SQ=-1;ID=S2662_raw:GT=hom:DP=216:FQ=1:SQ=-1;ID=S2663_raw:GT=hom:DP=128:FQ=1:SQ=-1;ID=S2011_raw:GT=hom:DP=161:FQ=1:SQ=-1;ID=S2504_raw:GT=hom:DP=161:FQ=1:SQ=-1;ID=S2008_raw:GT=hom:DP=107:FQ=1:SQ=-1;ID=S2509_raw:GT=hom:DP=237:FQ=1:SQ=-1;ID=S2508_raw:GT=hom:DP=229:FQ=1:SQ=-1;ID=S2666_raw:GT=hom:DP=105:FQ=1:SQ=-1;ID=S2669_raw:GT=hom:DP=135:FQ=1:SQ=-1;ID=S2166_raw:GT=hom:DP=176:FQ=1:SQ=-1;ID=S1775_raw:GT=hom:DP=142:FQ=1:SQ=-1;ID=S2501_raw:GT=hom:DP=189:FQ=1:SQ=-1;ID=S2500_raw:GT=hom:DP=142:FQ=1:SQ=-1;ID=S2502_raw:GT=hom:DP=247:FQ=1:SQ=-1;ID=S2505_raw:GT=hom:DP=203:FQ=1:SQ=-1;ID=S2176_raw:GT=hom:DP=182:FQ=1:SQ=-1;ID=S2506_raw:GT=hom:DP=200:FQ=1:SQ=-1;ID=S2667_raw:GT=hom:DP=132:FQ=1:SQ=-1;
2	2,09E+08	2,09E+08	A	-	intronic	IDH1	.	.	.	.	ID=S2009_raw:GT=hom:DP=18:FQ=1:SQ=-1;ID=S2503_raw:GT=hom:DP=91:FQ=1:SQ=-1;ID=S2507_raw:GT=hom:DP=86:FQ=0.98:SQ=-1;ID=S2668_raw:GT=hom:DP=131:FQ=1:SQ=-1;ID=S2012_raw:GT=hom:DP=24:FQ=1:SQ=-1;ID=S2010_raw:GT=hom:DP=18:FQ=1:SQ=-1;ID=S2662_raw:GT=hom:DP=5:FQ=1:SQ=-1;ID=S2663_raw:GT=hom:DP=83:FQ=1:SQ=-1;ID=S2011_raw:GT=hom:DP=29:FQ=0.97:SQ=-1;ID=S2504_raw:GT=hom:DP=115:FQ=1:SQ=-1;ID=S2008_raw:GT=hom:DP=21:FQ=1:SQ=-1;ID=S2509_raw:GT=hom:DP=122:FQ=1:SQ=-1;ID=S2508_raw:GT=hom:DP=106:FQ=1:SQ=-1;ID=S2666_raw:GT=hom:DP=91:FQ=1:SQ=-1;ID=S2669_raw:GT=hom:DP=38:FQ=1:SQ=-1;ID=S2166_raw:GT=hom:DP=66:FQ=1:SQ=-1;ID=S1775_raw:GT=hom:DP=42:FQ=1:SQ=-1;ID=S2501_raw:GT=hom:DP=76:FQ=1:SQ=-1;ID=S2500_raw:GT=hom:DP=107:FQ=1:SQ=-1;ID=S2502_raw:GT=hom:DP=82:FQ=1:SQ=-1;ID=S2505_raw:GT=hom:DP=107:FQ=1:SQ=-1;ID=S2176_raw:GT=hom:DP=65:FQ=0.98:SQ=-1;ID=S2506_raw:GT=hom:DP=97:FQ=0.98:SQ=-1;ID=S2667_raw:GT=hom:DP=99:FQ=0.98:SQ=-1;
3	1,29E+08	1,29E+08	C	A	intronic	IFT122	.	.	rs6805886	.	ID=S2009_raw:GT=hom:DP=8:FQ=1:SQ=-1;ID=S2503_raw:GT=hom:DP=21:FQ=1:SQ=-1;ID=S2507_raw:GT=hom:DP=30:FQ=1:SQ=-1;ID=S2668_raw:GT=hom:DP=21:FQ=1:SQ=-1;ID=S2012_raw:GT=hom:DP=5:FQ=1:SQ=-1;ID=S2010_raw:GT=hom:DP=12:FQ=1:SQ=-1;ID=S2662_raw:GT=hom:DP=3:FQ=1:SQ=-1;ID=S2663_raw:GT=hom:DP=36:FQ=1:SQ=-1;ID=S2011_raw:GT=hom:DP=14:FQ=1:SQ=-1;ID=S2504_raw:GT=hom:DP=17:FQ=1:SQ=-1;ID=S2008_raw:GT=hom:DP=10:FQ=1:SQ=-1;ID=S2509_raw:GT=hom:DP=22:FQ=1:SQ=-1;ID=S2508_raw:GT=hom:DP=23:FQ=1:SQ=-1;ID=S2666_raw:GT=hom:DP=11:FQ=1:SQ=-1;ID=S2669_raw:GT=hom:DP=24:FQ=1:SQ=-1;ID=S2166_raw:GT=hom:DP=21:FQ=1:SQ=-1;ID=S1775_raw:GT=hom:DP=17:FQ=1:SQ=-1;ID=S2501_raw:GT=hom:DP=28:FQ=1:SQ=-1;ID=S2500_raw:GT=hom:DP=20:FQ=1:SQ=-1;ID=S2502_raw:GT=hom:DP=36:FQ=1:SQ=-1;ID=S2505_raw:GT=hom:DP=28:FQ=1:SQ=-1;ID=S2176_raw:GT=hom:DP=27:FQ=1:SQ=-1;ID=S2506_raw:GT=hom:DP=26:FQ=1:SQ=-1;ID=S2667_raw:GT=hom:DP=26:FQ=1:SQ=-1;
1	2,07E+08	2,07E+08	T	C	intronic	IL10	.	.	rs3024494	.	ID=S2009_raw:GT=hom:DP=8:FQ=1:SQ=-1;ID=S2503_raw:GT=hom:DP=17:FQ=1:SQ=-1;ID=S2507_raw:GT=hom:DP=31:FQ=1:SQ=-1;ID=S2668_raw:GT=hom:DP=35:FQ=1:SQ=-1;ID=S2012_raw:GT=hom:DP=6:FQ=1:SQ=-1;ID=S2010_raw:GT=hom:DP=7:FQ=1:SQ=-1;ID=S2662_raw:GT=hom:DP=30:FQ=1:SQ=-1;ID=S2663_raw:GT=hom:DP=17:FQ=1:SQ=-1;ID=S2011_raw:GT=hom:DP=9:FQ=1:SQ=-1;ID=S2504_raw:GT=hom:DP=29:FQ=1:SQ=-1;ID=S2008_raw:GT=hom:DP=3:FQ=1:SQ=-1;ID=S2509_raw:GT=hom:DP=21:FQ=1:SQ=-1;ID=S2508_raw:GT=hom:DP=28:FQ=1:SQ=-1;ID=S2666_raw:GT=hom:DP=27:FQ=1:SQ=-1;ID=S2669_raw:GT=hom:DP=24:FQ=1:SQ=-1;ID=S2166_raw:GT=hom:DP=14:FQ=1:SQ=-1;ID=S1775_raw:GT=hom:DP=20:FQ=1:SQ=-1;ID=S2501_raw:GT=hom:DP=27:FQ=1:SQ=-1;ID=S2500_raw:GT=hom:DP=24:FQ=1:SQ=-1;ID=S2502_raw:GT=hom:DP=25:FQ=1:SQ=-1;ID=S2505_raw:GT=hom:DP=34:FQ=1:SQ=-1;ID=S2176_raw:GT=hom:DP=13:FQ=1:SQ=-1;ID=S2506_raw:GT=hom:DP=15:FQ=1:SQ=-1;ID=S2667_raw:GT=hom:DP=26:FQ=1:SQ=-1;
3	9975247	9975247	C	G	exonic	IL17RC	synonymous SNV	IL17RC:NM_001367279:exon16:c.1974C>G:p.G658G,IL17RC:NM_001203264:exon17:c.2043C>G:p.G681G,IL17RC:NM_001203265:exon17:c.2037C>G:p.G679G,IL17RC:NM_001367280:exon17:c.2049C>G:p.G683G,IL17RC:NM_001203263:exon18:c.2094C>G:p.G698G,IL17RC:NM_001367278:exon18:c.2055C>G:p.G685G,IL17RC:NM_032732:exon18:c.2088C>G:p.G696G,IL17RC:NM_153460:exon19:c.2133C>G:p.G711G,IL17RC:NM_153461:exon19:c.2346C>G:p.G782G	rs183956	.	ID=S2009_raw:GT=hom:DP=47:FQ=1:SQ=-1;ID=S2503_raw:GT=hom:DP=78:FQ=1:SQ=-1;ID=S2507_raw:GT=hom:DP=61:FQ=1:SQ=-1;ID=S2668_raw:GT=hom:DP=43:FQ=1:SQ=-1;ID=S2012_raw:GT=hom:DP=41:FQ=1:SQ=-1;ID=S2010_raw:GT=hom:DP=45:FQ=1:SQ=-1;ID=S2662_raw:GT=hom:DP=87:FQ=1:SQ=-1;ID=S2663_raw:GT=hom:DP=52:FQ=1:SQ=-1;ID=S2011_raw:GT=hom:DP=79:FQ=1:SQ=-1;ID=S2504_raw:GT=hom:DP=68:FQ=1:SQ=-1;ID=S2008_raw:GT=hom:DP=47:FQ=1:SQ=-1;ID=S2509_raw:GT=hom:DP=79:FQ=1:SQ=-1;ID=S2508_raw:GT=hom:DP=58:FQ=1:SQ=-1;ID=S2666_raw:GT=hom:DP=21:FQ=1:SQ=-1;ID=S2669_raw:GT=hom:DP=72:FQ=1:SQ=-1;ID=S2166_raw:GT=hom:DP=64:FQ=1:SQ=-1;ID=S1775_raw:GT=hom:DP=53:FQ=1:SQ=-1;ID=S2501_raw:GT=hom:DP=52:FQ=1:SQ=-1;ID=S2500_raw:GT=hom:DP=59:FQ=1:SQ=-1;ID=S2502_raw:GT=hom:DP=43:FQ=1:SQ=-1;ID=S2505_raw:GT=hom:DP=73:FQ=0.99:SQ=-1;ID=S2176_raw:GT=hom:DP=35:FQ=1:SQ=-1;ID=S2506_raw:GT=hom:DP=84:FQ=1:SQ=-1;ID=S2667_raw:GT=hom:DP=71:FQ=1:SQ=-1;
17	48155909	48155909	C	T	intronic	ITGA3	.	.	rs1858902	.	ID=S2009_raw:GT=hom:DP=3:FQ=1:SQ=-1;ID=S2503_raw:GT=hom:DP=7:FQ=1:SQ=-1;ID=S2507_raw:GT=hom:DP=9:FQ=1:SQ=-1;ID=S2668_raw:GT=hom:DP=10:FQ=1:SQ=-1;ID=S2012_raw:GT=hom:DP=3:FQ=1:SQ=-1;ID=S2010_raw:GT=hom:DP=1:FQ=1:SQ=-1;ID=S2662_raw:GT=hom:DP=2:FQ=1:SQ=-1;ID=S2663_raw:GT=hom:DP=19:FQ=1:SQ=-1;ID=S2011_raw:GT=hom:DP=8:FQ=1:SQ=-1;ID=S2504_raw:GT=hom:DP=9:FQ=1:SQ=-1;ID=S2008_raw:GT=hom:DP=2:FQ=1:SQ=-1;ID=S2509_raw:GT=hom:DP=10:FQ=1:SQ=-1;ID=S2508_raw:GT=hom:DP=23:FQ=1:SQ=-1;ID=S2666_raw:GT=hom:DP=8:FQ=1:SQ=-1;ID=S2669_raw:GT=hom:DP=15:FQ=1:SQ=-1;ID=S2166_raw:GT=hom:DP=4:FQ=1:SQ=-1;ID=S1775_raw:GT=hom:DP=2:FQ=1:SQ=-1;ID=S2501_raw:GT=hom:DP=3:FQ=1:SQ=-1;ID=S2500_raw:GT=hom:DP=13:FQ=1:SQ=-1;ID=S2502_raw:GT=hom:DP=10:FQ=1:SQ=-1;ID=S2505_raw:GT=hom:DP=10:FQ=1:SQ=-1;ID=S2176_raw:GT=hom:DP=2:FQ=1:SQ=-1;ID=S2506_raw:GT=hom:DP=10:FQ=1:SQ=-1;ID=S2667_raw:GT=hom:DP=18:FQ=1:SQ=-1;
10	15573050	15573050	A	G	exonic;splicing	ITGA8	nonsynonymous SNV	ITGA8:NM_001291494:exon27:c.2936T>C:p.V979A,ITGA8:NM_003638:exon28:c.2981T>C:p.V994A	rs1041135	.	ID=S2009_raw:GT=hom:DP=61:FQ=1:SQ=-1;ID=S2503_raw:GT=hom:DP=93:FQ=1:SQ=-1;ID=S2507_raw:GT=hom:DP=100:FQ=1:SQ=-1;ID=S2668_raw:GT=hom:DP=131:FQ=1:SQ=-1;ID=S2012_raw:GT=hom:DP=80:FQ=1:SQ=-1;ID=S2010_raw:GT=hom:DP=100:FQ=1:SQ=-1;ID=S2662_raw:GT=hom:DP=2:FQ=1:SQ=-1;ID=S2663_raw:GT=hom:DP=94:FQ=1:SQ=-1;ID=S2011_raw:GT=hom:DP=90:FQ=1:SQ=-1;ID=S2504_raw:GT=hom:DP=76:FQ=1:SQ=-1;ID=S2008_raw:GT=hom:DP=68:FQ=1:SQ=-1;ID=S2509_raw:GT=hom:DP=78:FQ=1:SQ=-1;ID=S2508_raw:GT=hom:DP=95:FQ=1:SQ=-1;ID=S2666_raw:GT=hom:DP=91:FQ=1:SQ=-1;ID=S2669_raw:GT=hom:DP=46:FQ=1:SQ=-1;ID=S2166_raw:GT=hom:DP=129:FQ=1:SQ=-1;ID=S1775_raw:GT=hom:DP=87:FQ=1:SQ=-1;ID=S2501_raw:GT=hom:DP=80:FQ=1:SQ=-1;ID=S2500_raw:GT=hom:DP=66:FQ=1:SQ=-1;ID=S2502_raw:GT=hom:DP=64:FQ=1:SQ=-1;ID=S2505_raw:GT=hom:DP=85:FQ=1:SQ=-1;ID=S2176_raw:GT=hom:DP=145:FQ=1:SQ=-1;ID=S2506_raw:GT=hom:DP=132:FQ=1:SQ=-1;ID=S2667_raw:GT=hom:DP=104:FQ=1:SQ=-1;
10	15590372	15590372	G	T	intronic	ITGA8	.	.	rs9333229	.	ID=S2009_raw:GT=hom:DP=16:FQ=1:SQ=-1;ID=S2503_raw:GT=hom:DP=11:FQ=1:SQ=-1;ID=S2507_raw:GT=hom:DP=11:FQ=1:SQ=-1;ID=S2668_raw:GT=hom:DP=24:FQ=1:SQ=-1;ID=S2012_raw:GT=hom:DP=40:FQ=1:SQ=-1;ID=S2010_raw:GT=hom:DP=40:FQ=1:SQ=-1;ID=S2662_raw:GT=hom:DP=4:FQ=1:SQ=-1;ID=S2663_raw:GT=hom:DP=14:FQ=1:SQ=-1;ID=S2011_raw:GT=hom:DP=35:FQ=1:SQ=-1;ID=S2504_raw:GT=hom:DP=20:FQ=1:SQ=-1;ID=S2008_raw:GT=hom:DP=28:FQ=1:SQ=-1;ID=S2509_raw:GT=hom:DP=15:FQ=1:SQ=-1;ID=S2508_raw:GT=hom:DP=13:FQ=1:SQ=-1;ID=S2666_raw:GT=hom:DP=16:FQ=1:SQ=-1;ID=S2669_raw:GT=hom:DP=15:FQ=1:SQ=-1;ID=S2166_raw:GT=hom:DP=11:FQ=1:SQ=-1;ID=S1775_raw:GT=hom:DP=6:FQ=1:SQ=-1;ID=S2501_raw:GT=hom:DP=12:FQ=1:SQ=-1;ID=S2500_raw:GT=hom:DP=21:FQ=1:SQ=-1;ID=S2502_raw:GT=hom:DP=16:FQ=1:SQ=-1;ID=S2505_raw:GT=hom:DP=16:FQ=1:SQ=-1;ID=S2176_raw:GT=hom:DP=8:FQ=1:SQ=-1;ID=S2506_raw:GT=hom:DP=13:FQ=1:SQ=-1;ID=S2667_raw:GT=hom:DP=13:FQ=1:SQ=-1;
17	73720900	73720900	A	G	intronic	ITGB4	.	.	rs820167	.	ID=S2009_raw:GT=hom:DP=6:FQ=1:SQ=-1;ID=S2503_raw:GT=hom:DP=84:FQ=1:SQ=-1;ID=S2507_raw:GT=hom:DP=77:FQ=1:SQ=-1;ID=S2668_raw:GT=hom:DP=89:FQ=1:SQ=-1;ID=S2012_raw:GT=hom:DP=7:FQ=1:SQ=-1;ID=S2010_raw:GT=hom:DP=12:FQ=1:SQ=-1;ID=S2662_raw:GT=hom:DP=188:FQ=0.99:SQ=-1;ID=S2663_raw:GT=hom:DP=92:FQ=1:SQ=-1;ID=S2011_raw:GT=hom:DP=15:FQ=1:SQ=-1;ID=S2504_raw:GT=hom:DP=97:FQ=1:SQ=-1;ID=S2008_raw:GT=hom:DP=16:FQ=1:SQ=-1;ID=S2509_raw:GT=hom:DP=101:FQ=1:SQ=-1;ID=S2508_raw:GT=hom:DP=113:FQ=1:SQ=-1;ID=S2666_raw:GT=hom:DP=115:FQ=1:SQ=-1;ID=S2669_raw:GT=hom:DP=80:FQ=1:SQ=-1;ID=S2166_raw:GT=hom:DP=76:FQ=1:SQ=-1;ID=S1775_raw:GT=hom:DP=56:FQ=1:SQ=-1;ID=S2501_raw:GT=hom:DP=72:FQ=1:SQ=-1;ID=S2500_raw:GT=hom:DP=84:FQ=1:SQ=-1;ID=S2502_raw:GT=hom:DP=96:FQ=1:SQ=-1;ID=S2505_raw:GT=hom:DP=105:FQ=1:SQ=-1;ID=S2176_raw:GT=hom:DP=97:FQ=1:SQ=-1;ID=S2506_raw:GT=hom:DP=72:FQ=1:SQ=-1;ID=S2667_raw:GT=hom:DP=99:FQ=1:SQ=-1;
17	73723459	73723459	C	-	intronic	ITGB4	.	.	.	.	ID=S2009_raw:GT=hom:DP=50:FQ=1:SQ=-1;ID=S2503_raw:GT=hom:DP=153:FQ=1:SQ=-1;ID=S2507_raw:GT=hom:DP=149:FQ=1:SQ=-1;ID=S2668_raw:GT=hom:DP=196:FQ=1:SQ=-1;ID=S2012_raw:GT=hom:DP=47:FQ=1:SQ=-1;ID=S2010_raw:GT=hom:DP=63:FQ=1:SQ=-1;ID=S2662_raw:GT=hom:DP=315:FQ=1:SQ=-1;ID=S2663_raw:GT=hom:DP=171:FQ=1:SQ=-1;ID=S2011_raw:GT=hom:DP=69:FQ=1:SQ=-1;ID=S2504_raw:GT=hom:DP=167:FQ=1:SQ=-1;ID=S2008_raw:GT=hom:DP=55:FQ=1:SQ=-1;ID=S2509_raw:GT=hom:DP=161:FQ=1:SQ=-1;ID=S2508_raw:GT=hom:DP=158:FQ=1:SQ=-1;ID=S2666_raw:GT=hom:DP=161:FQ=1:SQ=-1;ID=S2669_raw:GT=hom:DP=148:FQ=1:SQ=-1;ID=S2166_raw:GT=hom:DP=129:FQ=1:SQ=-1;ID=S1775_raw:GT=hom:DP=148:FQ=1:SQ=-1;ID=S2501_raw:GT=hom:DP=136:FQ=1:SQ=-1;ID=S2500_raw:GT=hom:DP=177:FQ=1:SQ=-1;ID=S2502_raw:GT=hom:DP=162:FQ=1:SQ=-1;ID=S2505_raw:GT=hom:DP=149:FQ=1:SQ=-1;ID=S2176_raw:GT=hom:DP=180:FQ=1:SQ=-1;ID=S2506_raw:GT=hom:DP=147:FQ=1:SQ=-1;ID=S2667_raw:GT=hom:DP=180:FQ=1:SQ=-1;
7	27886171	27886171	A	G	intronic	JAZF1	.	.	rs6462054	.	ID=S2009_raw:GT=hom:DP=60:FQ=1:SQ=-1;ID=S2503_raw:GT=hom:DP=93:FQ=1:SQ=-1;ID=S2507_raw:GT=hom:DP=115:FQ=1:SQ=-1;ID=S2668_raw:GT=hom:DP=193:FQ=1:SQ=-1;ID=S2012_raw:GT=hom:DP=56:FQ=1:SQ=-1;ID=S2010_raw:GT=hom:DP=72:FQ=1:SQ=-1;ID=S2662_raw:GT=hom:DP=18:FQ=1:SQ=-1;ID=S2663_raw:GT=hom:DP=99:FQ=1:SQ=-1;ID=S2011_raw:GT=hom:DP=108:FQ=1:SQ=-1;ID=S2504_raw:GT=hom:DP=107:FQ=1:SQ=-1;ID=S2008_raw:GT=hom:DP=94:FQ=1:SQ=-1;ID=S2509_raw:GT=hom:DP=146:FQ=1:SQ=-1;ID=S2508_raw:GT=hom:DP=128:FQ=1:SQ=-1;ID=S2666_raw:GT=hom:DP=221:FQ=1:SQ=-1;ID=S2669_raw:GT=hom:DP=77:FQ=1:SQ=-1;ID=S2166_raw:GT=hom:DP=205:FQ=1:SQ=-1;ID=S1775_raw:GT=hom:DP=142:FQ=1:SQ=-1;ID=S2501_raw:GT=hom:DP=99:FQ=1:SQ=-1;ID=S2500_raw:GT=hom:DP=113:FQ=1:SQ=-1;ID=S2502_raw:GT=hom:DP=116:FQ=1:SQ=-1;ID=S2505_raw:GT=hom:DP=124:FQ=1:SQ=-1;ID=S2176_raw:GT=hom:DP=208:FQ=1:SQ=-1;ID=S2506_raw:GT=hom:DP=88:FQ=1:SQ=-1;ID=S2667_raw:GT=hom:DP=126:FQ=1:SQ=-1;
10	64967445	64967445	A	T	exonic	JMJD1C	synonymous SNV	JMJD1C:NM_001322254:exon8:c.3327T>A:p.R1109R,JMJD1C:NM_001282948:exon9:c.3438T>A:p.R1146R,JMJD1C:NM_001318153:exon9:c.3120T>A:p.R1040R,JMJD1C:NM_001322252:exon9:c.3870T>A:p.R1290R,JMJD1C:NM_001322258:exon9:c.3327T>A:p.R1109R,JMJD1C:NM_001318154:exon10:c.3438T>A:p.R1146R,JMJD1C:NM_032776:exon10:c.3984T>A:p.R1328R	rs1904294	.	ID=S2009_raw:GT=hom:DP=137:FQ=1:SQ=-1;ID=S2503_raw:GT=hom:DP=116:FQ=1:SQ=-1;ID=S2507_raw:GT=hom:DP=81:FQ=1:SQ=-1;ID=S2668_raw:GT=hom:DP=96:FQ=1:SQ=-1;ID=S2012_raw:GT=hom:DP=170:FQ=1:SQ=-1;ID=S2010_raw:GT=hom:DP=208:FQ=1:SQ=-1;ID=S2662_raw:GT=hom:DP=9:FQ=1:SQ=-1;ID=S2663_raw:GT=hom:DP=88:FQ=1:SQ=-1;ID=S2011_raw:GT=hom:DP=233:FQ=1:SQ=-1;ID=S2504_raw:GT=hom:DP=101:FQ=1:SQ=-1;ID=S2008_raw:GT=hom:DP=172:FQ=1:SQ=-1;ID=S2509_raw:GT=hom:DP=96:FQ=1:SQ=-1;ID=S2508_raw:GT=hom:DP=107:FQ=1:SQ=-1;ID=S2666_raw:GT=hom:DP=85:FQ=1:SQ=-1;ID=S2669_raw:GT=hom:DP=74:FQ=1:SQ=-1;ID=S2166_raw:GT=hom:DP=382:FQ=1:SQ=-1;ID=S1775_raw:GT=hom:DP=172:FQ=1:SQ=-1;ID=S2501_raw:GT=hom:DP=90:FQ=0.98:SQ=-1;ID=S2500_raw:GT=hom:DP=81:FQ=1:SQ=-1;ID=S2502_raw:GT=hom:DP=112:FQ=1:SQ=-1;ID=S2505_raw:GT=hom:DP=116:FQ=1:SQ=-1;ID=S2176_raw:GT=hom:DP=383:FQ=1:SQ=-1;ID=S2506_raw:GT=hom:DP=64:FQ=1:SQ=-1;ID=S2667_raw:GT=hom:DP=83:FQ=1:SQ=-1;
10	65139945	65139945	T	C	intronic	JMJD1C	.	.	rs7477425	.	ID=S2009_raw:GT=hom:DP=16:FQ=1:SQ=-1;ID=S2503_raw:GT=hom:DP=44:FQ=1:SQ=-1;ID=S2507_raw:GT=hom:DP=22:FQ=1:SQ=-1;ID=S2668_raw:GT=hom:DP=30:FQ=1:SQ=-1;ID=S2012_raw:GT=hom:DP=35:FQ=1:SQ=-1;ID=S2010_raw:GT=hom:DP=29:FQ=1:SQ=-1;ID=S2662_raw:GT=hom:DP=14:FQ=1:SQ=-1;ID=S2663_raw:GT=hom:DP=25:FQ=1:SQ=-1;ID=S2011_raw:GT=hom:DP=43:FQ=1:SQ=-1;ID=S2504_raw:GT=hom:DP=32:FQ=1:SQ=-1;ID=S2008_raw:GT=hom:DP=31:FQ=1:SQ=-1;ID=S2509_raw:GT=hom:DP=34:FQ=1:SQ=-1;ID=S2508_raw:GT=hom:DP=32:FQ=1:SQ=-1;ID=S2666_raw:GT=hom:DP=34:FQ=1:SQ=-1;ID=S2669_raw:GT=hom:DP=37:FQ=1:SQ=-1;ID=S2166_raw:GT=hom:DP=19:FQ=1:SQ=-1;ID=S1775_raw:GT=hom:DP=11:FQ=1:SQ=-1;ID=S2501_raw:GT=hom:DP=34:FQ=1:SQ=-1;ID=S2500_raw:GT=hom:DP=24:FQ=1:SQ=-1;ID=S2502_raw:GT=hom:DP=34:FQ=1:SQ=-1;ID=S2505_raw:GT=hom:DP=31:FQ=1:SQ=-1;ID=S2176_raw:GT=hom:DP=29:FQ=1:SQ=-1;ID=S2506_raw:GT=hom:DP=27:FQ=1:SQ=-1;ID=S2667_raw:GT=hom:DP=41:FQ=1:SQ=-1;
10	65225245	65225245	A	G	ncRNA_exonic;splicing	JMJD1C-AS1;JMJD1C	.	NM_001322252:exon1:c.168+10T>C;NM_032776:exon1:c.168+10T>C	rs10761770	.	ID=S2009_raw:GT=hom:DP=9:FQ=1:SQ=-1;ID=S2503_raw:GT=hom:DP=68:FQ=1:SQ=-1;ID=S2507_raw:GT=hom:DP=72:FQ=1:SQ=-1;ID=S2668_raw:GT=hom:DP=54:FQ=1:SQ=-1;ID=S2012_raw:GT=hom:DP=7:FQ=1:SQ=-1;ID=S2010_raw:GT=hom:DP=9:FQ=1:SQ=-1;ID=S2662_raw:GT=hom:DP=115:FQ=1:SQ=-1;ID=S2663_raw:GT=hom:DP=82:FQ=1:SQ=-1;ID=S2011_raw:GT=hom:DP=11:FQ=1:SQ=-1;ID=S2504_raw:GT=hom:DP=88:FQ=1:SQ=-1;ID=S2008_raw:GT=hom:DP=11:FQ=1:SQ=-1;ID=S2509_raw:GT=hom:DP=53:FQ=1:SQ=-1;ID=S2508_raw:GT=hom:DP=78:FQ=1:SQ=-1;ID=S2666_raw:GT=hom:DP=39:FQ=1:SQ=-1;ID=S2669_raw:GT=hom:DP=78:FQ=1:SQ=-1;ID=S2166_raw:GT=hom:DP=52:FQ=1:SQ=-1;ID=S1775_raw:GT=hom:DP=43:FQ=1:SQ=-1;ID=S2501_raw:GT=hom:DP=72:FQ=1:SQ=-1;ID=S2500_raw:GT=hom:DP=45:FQ=1:SQ=-1;ID=S2502_raw:GT=hom:DP=49:FQ=1:SQ=-1;ID=S2505_raw:GT=hom:DP=73:FQ=1:SQ=-1;ID=S2176_raw:GT=hom:DP=64:FQ=1:SQ=-1;ID=S2506_raw:GT=hom:DP=47:FQ=1:SQ=-1;ID=S2667_raw:GT=hom:DP=58:FQ=1:SQ=-1;
17	44144993	44144993	C	G	exonic	KANSL1	nonsynonymous SNV	KANSL1:NM_001193466:exon5:c.1574G>C:p.R525P,KANSL1:NM_015443:exon5:c.1574G>C:p.R525P,KANSL1:NM_001193465:exon6:c.1574G>C:p.R525P	rs144838667	.	ID=S2009_raw:GT=hom:DP=45:FQ=0.98:SQ=-1;ID=S2503_raw:GT=hom:DP=134:FQ=1:SQ=-1;ID=S2507_raw:GT=hom:DP=116:FQ=1:SQ=-1;ID=S2668_raw:GT=hom:DP=180:FQ=1:SQ=-1;ID=S2012_raw:GT=hom:DP=72:FQ=1:SQ=-1;ID=S2010_raw:GT=hom:DP=103:FQ=1:SQ=-1;ID=S2662_raw:GT=hom:DP=23:FQ=1:SQ=-1;ID=S2663_raw:GT=hom:DP=114:FQ=1:SQ=-1;ID=S2011_raw:GT=hom:DP=74:FQ=1:SQ=-1;ID=S2504_raw:GT=hom:DP=170:FQ=1:SQ=-1;ID=S2008_raw:GT=hom:DP=59:FQ=1:SQ=-1;ID=S2509_raw:GT=hom:DP=111:FQ=1:SQ=-1;ID=S2508_raw:GT=hom:DP=113:FQ=1:SQ=-1;ID=S2666_raw:GT=hom:DP=173:FQ=1:SQ=-1;ID=S2669_raw:GT=hom:DP=94:FQ=1:SQ=-1;ID=S2166_raw:GT=hom:DP=266:FQ=1:SQ=-1;ID=S1775_raw:GT=hom:DP=229:FQ=1:SQ=-1;ID=S2501_raw:GT=hom:DP=117:FQ=1:SQ=-1;ID=S2500_raw:GT=hom:DP=134:FQ=1:SQ=-1;ID=S2502_raw:GT=hom:DP=127:FQ=1:SQ=-1;ID=S2505_raw:GT=hom:DP=121:FQ=1:SQ=-1;ID=S2176_raw:GT=hom:DP=328:FQ=1:SQ=-1;ID=S2506_raw:GT=hom:DP=111:FQ=1:SQ=-1;ID=S2667_raw:GT=hom:DP=126:FQ=1:SQ=-1;
17	44336866	44336866	T	C	intergenic	KANSL1;ARL17B	.	dist=34126;dist=14684	rs2429443	.	ID=S2009_raw:GT=hom:DP=4:FQ=1:SQ=-1;ID=S2503_raw:GT=hom:DP=59:FQ=1:SQ=-1;ID=S2507_raw:GT=hom:DP=69:FQ=1:SQ=-1;ID=S2668_raw:GT=hom:DP=74:FQ=1:SQ=-1;ID=S2012_raw:GT=hom:DP=4:FQ=1:SQ=-1;ID=S2010_raw:GT=hom:DP=9:FQ=1:SQ=-1;ID=S2662_raw:GT=hom:DP=82:FQ=1:SQ=-1;ID=S2663_raw:GT=hom:DP=61:FQ=1:SQ=-1;ID=S2011_raw:GT=hom:DP=5:FQ=1:SQ=-1;ID=S2504_raw:GT=hom:DP=59:FQ=1:SQ=-1;ID=S2008_raw:GT=hom:DP=5:FQ=1:SQ=-1;ID=S2509_raw:GT=hom:DP=57:FQ=1:SQ=-1;ID=S2508_raw:GT=hom:DP=68:FQ=1:SQ=-1;ID=S2666_raw:GT=hom:DP=52:FQ=1:SQ=-1;ID=S2669_raw:GT=hom:DP=52:FQ=1:SQ=-1;ID=S2166_raw:GT=hom:DP=30:FQ=1:SQ=-1;ID=S1775_raw:GT=hom:DP=23:FQ=1:SQ=-1;ID=S2501_raw:GT=hom:DP=59:FQ=1:SQ=-1;ID=S2500_raw:GT=hom:DP=62:FQ=1:SQ=-1;ID=S2502_raw:GT=hom:DP=65:FQ=1:SQ=-1;ID=S2505_raw:GT=hom:DP=63:FQ=1:SQ=-1;ID=S2176_raw:GT=hom:DP=48:FQ=1:SQ=-1;ID=S2506_raw:GT=hom:DP=104:FQ=1:SQ=-1;ID=S2667_raw:GT=hom:DP=36:FQ=1:SQ=-1;
11	65480596	65480596	A	G	intronic	KAT5	.	.	rs616250	.	ID=S2009_raw:GT=hom:DP=7:FQ=1:SQ=-1;ID=S2503_raw:GT=hom:DP=10:FQ=1:SQ=-1;ID=S2507_raw:GT=hom:DP=18:FQ=1:SQ=-1;ID=S2668_raw:GT=hom:DP=14:FQ=1:SQ=-1;ID=S2012_raw:GT=hom:DP=10:FQ=1:SQ=-1;ID=S2010_raw:GT=hom:DP=10:FQ=1:SQ=-1;ID=S2662_raw:GT=hom:DP=37:FQ=1:SQ=-1;ID=S2663_raw:GT=hom:DP=17:FQ=1:SQ=-1;ID=S2011_raw:GT=hom:DP=11:FQ=1:SQ=-1;ID=S2504_raw:GT=hom:DP=26:FQ=1:SQ=-1;ID=S2008_raw:GT=hom:DP=13:FQ=1:SQ=-1;ID=S2509_raw:GT=hom:DP=20:FQ=1:SQ=-1;ID=S2508_raw:GT=hom:DP=19:FQ=1:SQ=-1;ID=S2666_raw:GT=hom:DP=17:FQ=1:SQ=-1;ID=S2669_raw:GT=hom:DP=16:FQ=1:SQ=-1;ID=S2166_raw:GT=hom:DP=11:FQ=1:SQ=-1;ID=S1775_raw:GT=hom:DP=4:FQ=1:SQ=-1;ID=S2501_raw:GT=hom:DP=17:FQ=1:SQ=-1;ID=S2500_raw:GT=hom:DP=18:FQ=1:SQ=-1;ID=S2502_raw:GT=hom:DP=26:FQ=1:SQ=-1;ID=S2505_raw:GT=hom:DP=18:FQ=1:SQ=-1;ID=S2176_raw:GT=hom:DP=10:FQ=1:SQ=-1;ID=S2506_raw:GT=hom:DP=23:FQ=1:SQ=-1;ID=S2667_raw:GT=hom:DP=15:FQ=1:SQ=-1;
11	65480791	65480791	A	G	intronic	KAT5	.	.	rs551115	.	ID=S2009_raw:GT=hom:DP=26:FQ=1:SQ=-1;ID=S2503_raw:GT=hom:DP=89:FQ=1:SQ=-1;ID=S2507_raw:GT=hom:DP=87:FQ=1:SQ=-1;ID=S2668_raw:GT=hom:DP=68:FQ=1:SQ=-1;ID=S2012_raw:GT=hom:DP=30:FQ=1:SQ=-1;ID=S2010_raw:GT=hom:DP=24:FQ=1:SQ=-1;ID=S2662_raw:GT=hom:DP=123:FQ=1:SQ=-1;ID=S2663_raw:GT=hom:DP=73:FQ=1:SQ=-1;ID=S2011_raw:GT=hom:DP=34:FQ=1:SQ=-1;ID=S2504_raw:GT=hom:DP=90:FQ=1:SQ=-1;ID=S2008_raw:GT=hom:DP=46:FQ=1:SQ=-1;ID=S2509_raw:GT=hom:DP=68:FQ=1:SQ=-1;ID=S2508_raw:GT=hom:DP=55:FQ=1:SQ=-1;ID=S2666_raw:GT=hom:DP=51:FQ=1:SQ=-1;ID=S2669_raw:GT=hom:DP=77:FQ=1:SQ=-1;ID=S2166_raw:GT=hom:DP=85:FQ=1:SQ=-1;ID=S1775_raw:GT=hom:DP=56:FQ=1:SQ=-1;ID=S2501_raw:GT=hom:DP=73:FQ=1:SQ=-1;ID=S2500_raw:GT=hom:DP=61:FQ=1:SQ=-1;ID=S2502_raw:GT=hom:DP=81:FQ=1:SQ=-1;ID=S2505_raw:GT=hom:DP=57:FQ=1:SQ=-1;ID=S2176_raw:GT=hom:DP=96:FQ=1:SQ=-1;ID=S2506_raw:GT=hom:DP=77:FQ=1:SQ=-1;ID=S2667_raw:GT=hom:DP=70:FQ=1:SQ=-1;
1	2,11E+08	2,11E+08	G	T	intronic	KCNH1	.	.	rs4951484	.	ID=S2009_raw:GT=hom:DP=63:FQ=1:SQ=-1;ID=S2503_raw:GT=hom:DP=30:FQ=1:SQ=-1;ID=S2507_raw:GT=hom:DP=19:FQ=1:SQ=-1;ID=S2668_raw:GT=hom:DP=33:FQ=1:SQ=-1;ID=S2012_raw:GT=hom:DP=48:FQ=1:SQ=-1;ID=S2010_raw:GT=hom:DP=78:FQ=1:SQ=-1;ID=S2662_raw:GT=hom:DP=2:FQ=1:SQ=-1;ID=S2663_raw:GT=hom:DP=26:FQ=1:SQ=-1;ID=S2011_raw:GT=hom:DP=75:FQ=1:SQ=-1;ID=S2504_raw:GT=hom:DP=32:FQ=1:SQ=-1;ID=S2008_raw:GT=hom:DP=69:FQ=1:SQ=-1;ID=S2509_raw:GT=hom:DP=35:FQ=1:SQ=-1;ID=S2508_raw:GT=hom:DP=15:FQ=1:SQ=-1;ID=S2666_raw:GT=hom:DP=33:FQ=1:SQ=-1;ID=S2669_raw:GT=hom:DP=17:FQ=1:SQ=-1;ID=S2166_raw:GT=hom:DP=64:FQ=1:SQ=-1;ID=S1775_raw:GT=hom:DP=26:FQ=1:SQ=-1;ID=S2501_raw:GT=hom:DP=34:FQ=1:SQ=-1;ID=S2500_raw:GT=hom:DP=29:FQ=1:SQ=-1;ID=S2502_raw:GT=hom:DP=38:FQ=1:SQ=-1;ID=S2505_raw:GT=hom:DP=24:FQ=1:SQ=-1;ID=S2176_raw:GT=hom:DP=51:FQ=1:SQ=-1;ID=S2506_raw:GT=hom:DP=36:FQ=1:SQ=-1;ID=S2667_raw:GT=hom:DP=33:FQ=1:SQ=-1;
1	2,11E+08	2,11E+08	G	C	intronic	KCNH1	.	.	rs4951671	.	ID=S2009_raw:GT=hom:DP=49:FQ=1:SQ=-1;ID=S2503_raw:GT=hom:DP=22:FQ=1:SQ=-1;ID=S2507_raw:GT=hom:DP=21:FQ=1:SQ=-1;ID=S2668_raw:GT=hom:DP=37:FQ=1:SQ=-1;ID=S2012_raw:GT=hom:DP=39:FQ=1:SQ=-1;ID=S2010_raw:GT=hom:DP=55:FQ=1:SQ=-1;ID=S2662_raw:GT=hom:DP=9:FQ=1:SQ=-1;ID=S2663_raw:GT=hom:DP=22:FQ=1:SQ=-1;ID=S2011_raw:GT=hom:DP=61:FQ=1:SQ=-1;ID=S2504_raw:GT=hom:DP=26:FQ=1:SQ=-1;ID=S2008_raw:GT=hom:DP=46:FQ=1:SQ=-1;ID=S2509_raw:GT=hom:DP=22:FQ=1:SQ=-1;ID=S2508_raw:GT=hom:DP=12:FQ=1:SQ=-1;ID=S2666_raw:GT=hom:DP=31:FQ=1:SQ=-1;ID=S2669_raw:GT=hom:DP=25:FQ=1:SQ=-1;ID=S2166_raw:GT=hom:DP=54:FQ=1:SQ=-1;ID=S1775_raw:GT=hom:DP=30:FQ=1:SQ=-1;ID=S2501_raw:GT=hom:DP=21:FQ=1:SQ=-1;ID=S2500_raw:GT=hom:DP=28:FQ=1:SQ=-1;ID=S2502_raw:GT=hom:DP=38:FQ=1:SQ=-1;ID=S2505_raw:GT=hom:DP=25:FQ=1:SQ=-1;ID=S2176_raw:GT=hom:DP=52:FQ=1:SQ=-1;ID=S2506_raw:GT=hom:DP=25:FQ=1:SQ=-1;ID=S2667_raw:GT=hom:DP=21:FQ=1:SQ=-1;
1	2,11E+08	2,11E+08	T	C	intronic	KCNH1	.	.	rs4951672	.	ID=S2009_raw:GT=hom:DP=21:FQ=1:SQ=-1;ID=S2503_raw:GT=hom:DP=18:FQ=1:SQ=-1;ID=S2507_raw:GT=hom:DP=15:FQ=1:SQ=-1;ID=S2668_raw:GT=hom:DP=35:FQ=1:SQ=-1;ID=S2012_raw:GT=hom:DP=22:FQ=1:SQ=-1;ID=S2010_raw:GT=hom:DP=34:FQ=1:SQ=-1;ID=S2662_raw:GT=hom:DP=9:FQ=1:SQ=-1;ID=S2663_raw:GT=hom:DP=18:FQ=1:SQ=-1;ID=S2011_raw:GT=hom:DP=35:FQ=1:SQ=-1;ID=S2504_raw:GT=hom:DP=18:FQ=1:SQ=-1;ID=S2008_raw:GT=hom:DP=22:FQ=1:SQ=-1;ID=S2509_raw:GT=hom:DP=14:FQ=1:SQ=-1;ID=S2508_raw:GT=hom:DP=11:FQ=1:SQ=-1;ID=S2666_raw:GT=hom:DP=21:FQ=1:SQ=-1;ID=S2669_raw:GT=hom:DP=18:FQ=1:SQ=-1;ID=S2166_raw:GT=hom:DP=49:FQ=1:SQ=-1;ID=S1775_raw:GT=hom:DP=33:FQ=1:SQ=-1;ID=S2501_raw:GT=hom:DP=20:FQ=1:SQ=-1;ID=S2500_raw:GT=hom:DP=18:FQ=1:SQ=-1;ID=S2502_raw:GT=hom:DP=24:FQ=1:SQ=-1;ID=S2505_raw:GT=hom:DP=19:FQ=1:SQ=-1;ID=S2176_raw:GT=hom:DP=45:FQ=1:SQ=-1;ID=S2506_raw:GT=hom:DP=20:FQ=1:SQ=-1;ID=S2667_raw:GT=hom:DP=16:FQ=1:SQ=-1;
1	2,11E+08	2,11E+08	A	C	intronic	KCNH1	.	.	rs4951485	.	ID=S2009_raw:GT=hom:DP=14:FQ=1:SQ=-1;ID=S2503_raw:GT=hom:DP=18:FQ=1:SQ=-1;ID=S2507_raw:GT=hom:DP=12:FQ=1:SQ=-1;ID=S2668_raw:GT=hom:DP=30:FQ=1:SQ=-1;ID=S2012_raw:GT=hom:DP=12:FQ=1:SQ=-1;ID=S2010_raw:GT=hom:DP=21:FQ=1:SQ=-1;ID=S2662_raw:GT=hom:DP=5:FQ=1:SQ=-1;ID=S2663_raw:GT=hom:DP=13:FQ=1:SQ=-1;ID=S2011_raw:GT=hom:DP=22:FQ=1:SQ=-1;ID=S2504_raw:GT=hom:DP=16:FQ=1:SQ=-1;ID=S2008_raw:GT=hom:DP=19:FQ=1:SQ=-1;ID=S2509_raw:GT=hom:DP=12:FQ=1:SQ=-1;ID=S2508_raw:GT=hom:DP=10:FQ=1:SQ=-1;ID=S2666_raw:GT=hom:DP=19:FQ=1:SQ=-1;ID=S2669_raw:GT=hom:DP=16:FQ=1:SQ=-1;ID=S2166_raw:GT=hom:DP=40:FQ=1:SQ=-1;ID=S1775_raw:GT=hom:DP=32:FQ=1:SQ=-1;ID=S2501_raw:GT=hom:DP=13:FQ=1:SQ=-1;ID=S2500_raw:GT=hom:DP=18:FQ=1:SQ=-1;ID=S2502_raw:GT=hom:DP=21:FQ=1:SQ=-1;ID=S2505_raw:GT=hom:DP=14:FQ=1:SQ=-1;ID=S2176_raw:GT=hom:DP=41:FQ=1:SQ=-1;ID=S2506_raw:GT=hom:DP=10:FQ=1:SQ=-1;ID=S2667_raw:GT=hom:DP=9:FQ=1:SQ=-1;
1	2,11E+08	2,11E+08	G	T	intronic	KCNH1	.	.	rs4951673	.	ID=S2009_raw:GT=hom:DP=12:FQ=1:SQ=-1;ID=S2503_raw:GT=hom:DP=20:FQ=1:SQ=-1;ID=S2507_raw:GT=hom:DP=12:FQ=1:SQ=-1;ID=S2668_raw:GT=hom:DP=32:FQ=1:SQ=-1;ID=S2012_raw:GT=hom:DP=12:FQ=1:SQ=-1;ID=S2010_raw:GT=hom:DP=21:FQ=1:SQ=-1;ID=S2662_raw:GT=hom:DP=4:FQ=1:SQ=-1;ID=S2663_raw:GT=hom:DP=14:FQ=1:SQ=-1;ID=S2011_raw:GT=hom:DP=19:FQ=1:SQ=-1;ID=S2504_raw:GT=hom:DP=16:FQ=1:SQ=-1;ID=S2008_raw:GT=hom:DP=17:FQ=1:SQ=-1;ID=S2509_raw:GT=hom:DP=10:FQ=1:SQ=-1;ID=S2508_raw:GT=hom:DP=8:FQ=1:SQ=-1;ID=S2666_raw:GT=hom:DP=18:FQ=1:SQ=-1;ID=S2669_raw:GT=hom:DP=13:FQ=1:SQ=-1;ID=S2166_raw:GT=hom:DP=41:FQ=1:SQ=-1;ID=S1775_raw:GT=hom:DP=30:FQ=1:SQ=-1;ID=S2501_raw:GT=hom:DP=15:FQ=1:SQ=-1;ID=S2500_raw:GT=hom:DP=18:FQ=1:SQ=-1;ID=S2502_raw:GT=hom:DP=20:FQ=1:SQ=-1;ID=S2505_raw:GT=hom:DP=13:FQ=1:SQ=-1;ID=S2176_raw:GT=hom:DP=40:FQ=1:SQ=-1;ID=S2506_raw:GT=hom:DP=9:FQ=1:SQ=-1;ID=S2667_raw:GT=hom:DP=8:FQ=1:SQ=-1;
1	2,11E+08	2,11E+08	G	-	intronic	KCNH1	.	.	rs55659105	.	ID=S2009_raw:GT=hom:DP=8:FQ=1:SQ=-1;ID=S2503_raw:GT=hom:DP=16:FQ=1:SQ=-1;ID=S2507_raw:GT=hom:DP=8:FQ=1:SQ=-1;ID=S2668_raw:GT=hom:DP=24:FQ=1:SQ=-1;ID=S2012_raw:GT=hom:DP=8:FQ=1:SQ=-1;ID=S2010_raw:GT=hom:DP=17:FQ=1:SQ=-1;ID=S2662_raw:GT=hom:DP=3:FQ=1:SQ=-1;ID=S2663_raw:GT=hom:DP=11:FQ=1:SQ=-1;ID=S2011_raw:GT=hom:DP=13:FQ=1:SQ=-1;ID=S2504_raw:GT=hom:DP=14:FQ=1:SQ=-1;ID=S2008_raw:GT=hom:DP=14:FQ=1:SQ=-1;ID=S2509_raw:GT=hom:DP=9:FQ=1:SQ=-1;ID=S2508_raw:GT=hom:DP=6:FQ=1:SQ=-1;ID=S2666_raw:GT=hom:DP=16:FQ=1:SQ=-1;ID=S2669_raw:GT=hom:DP=11:FQ=1:SQ=-1;ID=S2166_raw:GT=hom:DP=34:FQ=1:SQ=-1;ID=S1775_raw:GT=hom:DP=22:FQ=1:SQ=-1;ID=S2501_raw:GT=hom:DP=12:FQ=1:SQ=-1;ID=S2500_raw:GT=hom:DP=14:FQ=1:SQ=-1;ID=S2502_raw:GT=hom:DP=18:FQ=1:SQ=-1;ID=S2505_raw:GT=hom:DP=11:FQ=1:SQ=-1;ID=S2176_raw:GT=hom:DP=36:FQ=1:SQ=-1;ID=S2506_raw:GT=hom:DP=7:FQ=1:SQ=-1;ID=S2667_raw:GT=hom:DP=6:FQ=1:SQ=-1;
11	1,29E+08	1,29E+08	C	G	exonic	KCNJ5	nonsynonymous SNV	KCNJ5:NM_000890:exon2:c.844C>G:p.Q282E,KCNJ5:NM_001354169:exon3:c.844C>G:p.Q282E	rs7102584	Benign	ID=S2009_raw:GT=hom:DP=58:FQ=1:SQ=-1;ID=S2503_raw:GT=hom:DP=116:FQ=1:SQ=-1;ID=S2507_raw:GT=hom:DP=95:FQ=1:SQ=-1;ID=S2668_raw:GT=hom:DP=126:FQ=1:SQ=-1;ID=S2012_raw:GT=hom:DP=88:FQ=1:SQ=-1;ID=S2010_raw:GT=hom:DP=91:FQ=1:SQ=-1;ID=S2662_raw:GT=hom:DP=183:FQ=0.99:SQ=-1;ID=S2663_raw:GT=hom:DP=116:FQ=1:SQ=-1;ID=S2011_raw:GT=hom:DP=105:FQ=1:SQ=-1;ID=S2504_raw:GT=hom:DP=138:FQ=1:SQ=-1;ID=S2008_raw:GT=hom:DP=89:FQ=1:SQ=-1;ID=S2509_raw:GT=hom:DP=105:FQ=1:SQ=-1;ID=S2508_raw:GT=hom:DP=147:FQ=1:SQ=-1;ID=S2666_raw:GT=hom:DP=106:FQ=1:SQ=-1;ID=S2669_raw:GT=hom:DP=115:FQ=1:SQ=-1;ID=S2166_raw:GT=hom:DP=140:FQ=1:SQ=-1;ID=S1775_raw:GT=hom:DP=134:FQ=1:SQ=-1;ID=S2501_raw:GT=hom:DP=100:FQ=1:SQ=-1;ID=S2500_raw:GT=hom:DP=104:FQ=1:SQ=-1;ID=S2502_raw:GT=hom:DP=139:FQ=1:SQ=-1;ID=S2505_raw:GT=hom:DP=96:FQ=1:SQ=-1;ID=S2176_raw:GT=hom:DP=149:FQ=1:SQ=-1;ID=S2506_raw:GT=hom:DP=103:FQ=1:SQ=-1;ID=S2667_raw:GT=hom:DP=109:FQ=1:SQ=-1;
11	2789856	2789856	A	G	intronic	KCNQ1	.	.	rs163161	.	ID=S2009_raw:GT=hom:DP=3:FQ=1:SQ=-1;ID=S2503_raw:GT=hom:DP=16:FQ=1:SQ=-1;ID=S2507_raw:GT=hom:DP=13:FQ=1:SQ=-1;ID=S2668_raw:GT=hom:DP=13:FQ=1:SQ=-1;ID=S2012_raw:GT=hom:DP=4:FQ=1:SQ=-1;ID=S2010_raw:GT=hom:DP=6:FQ=1:SQ=-1;ID=S2662_raw:GT=hom:DP=14:FQ=1:SQ=-1;ID=S2663_raw:GT=hom:DP=12:FQ=1:SQ=-1;ID=S2011_raw:GT=hom:DP=12:FQ=1:SQ=-1;ID=S2504_raw:GT=hom:DP=17:FQ=1:SQ=-1;ID=S2008_raw:GT=hom:DP=9:FQ=1:SQ=-1;ID=S2509_raw:GT=hom:DP=25:FQ=1:SQ=-1;ID=S2508_raw:GT=hom:DP=17:FQ=1:SQ=-1;ID=S2666_raw:GT=hom:DP=11:FQ=1:SQ=-1;ID=S2669_raw:GT=hom:DP=16:FQ=1:SQ=-1;ID=S2166_raw:GT=hom:DP=17:FQ=1:SQ=-1;ID=S1775_raw:GT=hom:DP=11:FQ=1:SQ=-1;ID=S2501_raw:GT=hom:DP=15:FQ=1:SQ=-1;ID=S2500_raw:GT=hom:DP=20:FQ=1:SQ=-1;ID=S2502_raw:GT=hom:DP=27:FQ=1:SQ=-1;ID=S2505_raw:GT=hom:DP=15:FQ=1:SQ=-1;ID=S2176_raw:GT=hom:DP=16:FQ=1:SQ=-1;ID=S2506_raw:GT=hom:DP=21:FQ=1:SQ=-1;ID=S2667_raw:GT=hom:DP=15:FQ=1:SQ=-1;
11	2798448	2798448	A	G	intronic	KCNQ1	.	.	rs163148	.	ID=S2009_raw:GT=hom:DP=17:FQ=1:SQ=-1;ID=S2503_raw:GT=hom:DP=51:FQ=1:SQ=-1;ID=S2507_raw:GT=hom:DP=43:FQ=1:SQ=-1;ID=S2668_raw:GT=hom:DP=43:FQ=1:SQ=-1;ID=S2012_raw:GT=hom:DP=11:FQ=1:SQ=-1;ID=S2010_raw:GT=hom:DP=14:FQ=1:SQ=-1;ID=S2662_raw:GT=hom:DP=70:FQ=1:SQ=-1;ID=S2663_raw:GT=hom:DP=57:FQ=1:SQ=-1;ID=S2011_raw:GT=hom:DP=18:FQ=1:SQ=-1;ID=S2504_raw:GT=hom:DP=54:FQ=1:SQ=-1;ID=S2008_raw:GT=hom:DP=14:FQ=1:SQ=-1;ID=S2509_raw:GT=hom:DP=50:FQ=1:SQ=-1;ID=S2508_raw:GT=hom:DP=59:FQ=1:SQ=-1;ID=S2666_raw:GT=hom:DP=39:FQ=1:SQ=-1;ID=S2669_raw:GT=hom:DP=53:FQ=1:SQ=-1;ID=S2166_raw:GT=hom:DP=28:FQ=1:SQ=-1;ID=S1775_raw:GT=hom:DP=15:FQ=1:SQ=-1;ID=S2501_raw:GT=hom:DP=53:FQ=1:SQ=-1;ID=S2500_raw:GT=hom:DP=60:FQ=1:SQ=-1;ID=S2502_raw:GT=hom:DP=60:FQ=1:SQ=-1;ID=S2505_raw:GT=hom:DP=46:FQ=1:SQ=-1;ID=S2176_raw:GT=hom:DP=27:FQ=1:SQ=-1;ID=S2506_raw:GT=hom:DP=37:FQ=1:SQ=-1;ID=S2667_raw:GT=hom:DP=60:FQ=1:SQ=-1;
14	58920240	58920240	A	C	intronic	KIAA0586	.	.	rs1748975	.	ID=S2009_raw:GT=hom:DP=12:FQ=1:SQ=-1;ID=S2503_raw:GT=hom:DP=30:FQ=1:SQ=-1;ID=S2507_raw:GT=hom:DP=30:FQ=1:SQ=-1;ID=S2668_raw:GT=hom:DP=37:FQ=1:SQ=-1;ID=S2012_raw:GT=hom:DP=8:FQ=1:SQ=-1;ID=S2010_raw:GT=hom:DP=8:FQ=1:SQ=-1;ID=S2662_raw:GT=hom:DP=1:FQ=1:SQ=-1;ID=S2663_raw:GT=hom:DP=26:FQ=1:SQ=-1;ID=S2011_raw:GT=hom:DP=16:FQ=1:SQ=-1;ID=S2504_raw:GT=hom:DP=27:FQ=1:SQ=-1;ID=S2008_raw:GT=hom:DP=15:FQ=1:SQ=-1;ID=S2509_raw:GT=hom:DP=31:FQ=1:SQ=-1;ID=S2508_raw:GT=hom:DP=26:FQ=1:SQ=-1;ID=S2666_raw:GT=hom:DP=33:FQ=1:SQ=-1;ID=S2669_raw:GT=hom:DP=11:FQ=1:SQ=-1;ID=S2166_raw:GT=hom:DP=30:FQ=1:SQ=-1;ID=S1775_raw:GT=hom:DP=13:FQ=1:SQ=-1;ID=S2501_raw:GT=hom:DP=25:FQ=1:SQ=-1;ID=S2500_raw:GT=hom:DP=28:FQ=1:SQ=-1;ID=S2502_raw:GT=hom:DP=21:FQ=1:SQ=-1;ID=S2505_raw:GT=hom:DP=25:FQ=1:SQ=-1;ID=S2176_raw:GT=hom:DP=21:FQ=1:SQ=-1;ID=S2506_raw:GT=hom:DP=28:FQ=1:SQ=-1;ID=S2667_raw:GT=hom:DP=25:FQ=0.96:SQ=-1;
14	58932772	58932772	A	G	intronic	KIAA0586	.	.	rs1743716	.	ID=S2009_raw:GT=hom:DP=38:FQ=1:SQ=-1;ID=S2503_raw:GT=hom:DP=26:FQ=1:SQ=-1;ID=S2507_raw:GT=hom:DP=29:FQ=1:SQ=-1;ID=S2668_raw:GT=hom:DP=32:FQ=1:SQ=-1;ID=S2012_raw:GT=hom:DP=34:FQ=1:SQ=-1;ID=S2010_raw:GT=hom:DP=29:FQ=1:SQ=-1;ID=S2662_raw:GT=hom:DP=6:FQ=1:SQ=-1;ID=S2663_raw:GT=hom:DP=24:FQ=1:SQ=-1;ID=S2011_raw:GT=hom:DP=46:FQ=1:SQ=-1;ID=S2504_raw:GT=hom:DP=16:FQ=1:SQ=-1;ID=S2008_raw:GT=hom:DP=38:FQ=1:SQ=-1;ID=S2509_raw:GT=hom:DP=26:FQ=1:SQ=-1;ID=S2508_raw:GT=hom:DP=23:FQ=1:SQ=-1;ID=S2666_raw:GT=hom:DP=42:FQ=1:SQ=-1;ID=S2669_raw:GT=hom:DP=21:FQ=1:SQ=-1;ID=S2166_raw:GT=hom:DP=91:FQ=1:SQ=-1;ID=S1775_raw:GT=hom:DP=68:FQ=1:SQ=-1;ID=S2501_raw:GT=hom:DP=31:FQ=1:SQ=-1;ID=S2500_raw:GT=hom:DP=24:FQ=1:SQ=-1;ID=S2502_raw:GT=hom:DP=21:FQ=1:SQ=-1;ID=S2505_raw:GT=hom:DP=42:FQ=1:SQ=-1;ID=S2176_raw:GT=hom:DP=107:FQ=1:SQ=-1;ID=S2506_raw:GT=hom:DP=29:FQ=1:SQ=-1;ID=S2667_raw:GT=hom:DP=33:FQ=1:SQ=-1;
14	58953746	58953746	C	G	exonic	KIAA0586	nonsynonymous SNV	KIAA0586:NM_001244193:exon19:c.2746C>G:p.P916A,KIAA0586:NM_014749:exon21:c.2938C>G:p.P980A,KIAA0586:NM_001244191:exon22:c.2911C>G:p.P971A,KIAA0586:NM_001329943:exon22:c.3166C>G:p.P1056A,KIAA0586:NM_001329944:exon22:c.3166C>G:p.P1056A,KIAA0586:NM_001329945:exon22:c.2911C>G:p.P971A,KIAA0586:NM_001329946:exon22:c.3166C>G:p.P1056A,KIAA0586:NM_001329947:exon22:c.3166C>G:p.P1056A,KIAA0586:NM_001364700:exon22:c.2911C>G:p.P971A,KIAA0586:NM_001364701:exon22:c.2911C>G:p.P971A,KIAA0586:NM_001244190:exon23:c.3121C>G:p.P1041A,KIAA0586:NM_001244192:exon23:c.3034C>G:p.P1012A,KIAA0586:NM_001244189:exon24:c.3325C>G:p.P1109A	rs1617510	.	ID=S2009_raw:GT=hom:DP=150:FQ=1:SQ=-1;ID=S2503_raw:GT=hom:DP=84:FQ=1:SQ=-1;ID=S2507_raw:GT=hom:DP=120:FQ=1:SQ=-1;ID=S2668_raw:GT=hom:DP=113:FQ=1:SQ=-1;ID=S2012_raw:GT=hom:DP=213:FQ=1:SQ=-1;ID=S2010_raw:GT=hom:DP=209:FQ=1:SQ=-1;ID=S2662_raw:GT=hom:DP=12:FQ=1:SQ=-1;ID=S2663_raw:GT=hom:DP=89:FQ=1:SQ=-1;ID=S2011_raw:GT=hom:DP=294:FQ=1:SQ=-1;ID=S2504_raw:GT=hom:DP=113:FQ=1:SQ=-1;ID=S2008_raw:GT=hom:DP=210:FQ=1:SQ=-1;ID=S2509_raw:GT=hom:DP=96:FQ=1:SQ=-1;ID=S2508_raw:GT=hom:DP=95:FQ=1:SQ=-1;ID=S2666_raw:GT=hom:DP=111:FQ=1:SQ=-1;ID=S2669_raw:GT=hom:DP=88:FQ=1:SQ=-1;ID=S2166_raw:GT=hom:DP=139:FQ=1:SQ=-1;ID=S1775_raw:GT=hom:DP=81:FQ=1:SQ=-1;ID=S2501_raw:GT=hom:DP=83:FQ=1:SQ=-1;ID=S2500_raw:GT=hom:DP=93:FQ=1:SQ=-1;ID=S2502_raw:GT=hom:DP=84:FQ=1:SQ=-1;ID=S2505_raw:GT=hom:DP=97:FQ=1:SQ=-1;ID=S2176_raw:GT=hom:DP=152:FQ=1:SQ=-1;ID=S2506_raw:GT=hom:DP=99:FQ=1:SQ=-1;ID=S2667_raw:GT=hom:DP=97:FQ=1:SQ=-1;
1	10335315	10335315	G	A	intronic	KIF1B	.	.	rs6685773	.	ID=S2009_raw:GT=hom:DP=4:FQ=1:SQ=-1;ID=S2503_raw:GT=hom:DP=13:FQ=1:SQ=-1;ID=S2507_raw:GT=hom:DP=31:FQ=1:SQ=-1;ID=S2668_raw:GT=hom:DP=35:FQ=1:SQ=-1;ID=S2012_raw:GT=hom:DP=2:FQ=1:SQ=-1;ID=S2010_raw:GT=hom:DP=4:FQ=1:SQ=-1;ID=S2662_raw:GT=hom:DP=4:FQ=1:SQ=-1;ID=S2663_raw:GT=hom:DP=26:FQ=1:SQ=-1;ID=S2011_raw:GT=hom:DP=4:FQ=1:SQ=-1;ID=S2504_raw:GT=hom:DP=33:FQ=1:SQ=-1;ID=S2008_raw:GT=hom:DP=4:FQ=1:SQ=-1;ID=S2509_raw:GT=hom:DP=21:FQ=1:SQ=-1;ID=S2508_raw:GT=hom:DP=23:FQ=1:SQ=-1;ID=S2666_raw:GT=hom:DP=36:FQ=1:SQ=-1;ID=S2669_raw:GT=hom:DP=12:FQ=1:SQ=-1;ID=S2166_raw:GT=hom:DP=15:FQ=1:SQ=-1;ID=S1775_raw:GT=hom:DP=13:FQ=1:SQ=-1;ID=S2501_raw:GT=hom:DP=31:FQ=1:SQ=-1;ID=S2500_raw:GT=hom:DP=17:FQ=1:SQ=-1;ID=S2502_raw:GT=hom:DP=30:FQ=1:SQ=-1;ID=S2505_raw:GT=hom:DP=25:FQ=1:SQ=-1;ID=S2176_raw:GT=hom:DP=30:FQ=1:SQ=-1;ID=S2506_raw:GT=hom:DP=23:FQ=1:SQ=-1;ID=S2667_raw:GT=hom:DP=28:FQ=1:SQ=-1;
15	90188514	90188514	A	G	intronic	KIF7	.	.	rs894159	.	ID=S2009_raw:GT=hom:DP=72:FQ=1:SQ=-1;ID=S2503_raw:GT=hom:DP=115:FQ=1:SQ=-1;ID=S2507_raw:GT=hom:DP=105:FQ=1:SQ=-1;ID=S2668_raw:GT=hom:DP=167:FQ=1:SQ=-1;ID=S2012_raw:GT=hom:DP=67:FQ=1:SQ=-1;ID=S2010_raw:GT=hom:DP=73:FQ=1:SQ=-1;ID=S2662_raw:GT=hom:DP=202:FQ=1:SQ=-1;ID=S2663_raw:GT=hom:DP=117:FQ=1:SQ=-1;ID=S2011_raw:GT=hom:DP=114:FQ=1:SQ=-1;ID=S2504_raw:GT=hom:DP=157:FQ=1:SQ=-1;ID=S2008_raw:GT=hom:DP=88:FQ=1:SQ=-1;ID=S2509_raw:GT=hom:DP=115:FQ=1:SQ=-1;ID=S2508_raw:GT=hom:DP=149:FQ=1:SQ=-1;ID=S2666_raw:GT=hom:DP=122:FQ=1:SQ=-1;ID=S2669_raw:GT=hom:DP=117:FQ=1:SQ=-1;ID=S2166_raw:GT=hom:DP=83:FQ=1:SQ=-1;ID=S1775_raw:GT=hom:DP=78:FQ=1:SQ=-1;ID=S2501_raw:GT=hom:DP=102:FQ=1:SQ=-1;ID=S2500_raw:GT=hom:DP=108:FQ=1:SQ=-1;ID=S2502_raw:GT=hom:DP=111:FQ=1:SQ=-1;ID=S2505_raw:GT=hom:DP=117:FQ=1:SQ=-1;ID=S2176_raw:GT=hom:DP=89:FQ=1:SQ=-1;ID=S2506_raw:GT=hom:DP=101:FQ=1:SQ=-1;ID=S2667_raw:GT=hom:DP=148:FQ=1:SQ=-1;
13	33590851	33590851	T	C	exonic	KL	synonymous SNV	KL:NM_004795:exon1:c.273T>C:p.D91D	rs2772364	Benign	ID=S2009_raw:GT=hom:DP=64:FQ=1:SQ=-1;ID=S2503_raw:GT=hom:DP=144:FQ=1:SQ=-1;ID=S2507_raw:GT=hom:DP=190:FQ=1:SQ=-1;ID=S2668_raw:GT=hom:DP=136:FQ=1:SQ=-1;ID=S2012_raw:GT=hom:DP=71:FQ=1:SQ=-1;ID=S2010_raw:GT=hom:DP=68:FQ=1:SQ=-1;ID=S2662_raw:GT=hom:DP=244:FQ=1:SQ=-1;ID=S2663_raw:GT=hom:DP=171:FQ=1:SQ=-1;ID=S2011_raw:GT=hom:DP=97:FQ=1:SQ=-1;ID=S2504_raw:GT=hom:DP=150:FQ=1:SQ=-1;ID=S2008_raw:GT=hom:DP=68:FQ=1:SQ=-1;ID=S2509_raw:GT=hom:DP=184:FQ=1:SQ=-1;ID=S2508_raw:GT=hom:DP=224:FQ=1:SQ=-1;ID=S2666_raw:GT=hom:DP=62:FQ=1:SQ=-1;ID=S2669_raw:GT=hom:DP=180:FQ=1:SQ=-1;ID=S2166_raw:GT=hom:DP=165:FQ=1:SQ=-1;ID=S1775_raw:GT=hom:DP=96:FQ=0.98:SQ=-1;ID=S2501_raw:GT=hom:DP=167:FQ=1:SQ=-1;ID=S2500_raw:GT=hom:DP=178:FQ=1:SQ=-1;ID=S2502_raw:GT=hom:DP=182:FQ=1:SQ=-1;ID=S2505_raw:GT=hom:DP=150:FQ=1:SQ=-1;ID=S2176_raw:GT=hom:DP=144:FQ=1:SQ=-1;ID=S2506_raw:GT=hom:DP=152:FQ=1:SQ=-1;ID=S2667_raw:GT=hom:DP=199:FQ=1:SQ=-1;
13	33635835	33635835	T	C	exonic	KL	synonymous SNV	KL:NM_004795:exon4:c.2619T>C:p.N873N	rs649964	Benign	ID=S2009_raw:GT=hom:DP=48:FQ=1:SQ=-1;ID=S2503_raw:GT=hom:DP=78:FQ=1:SQ=-1;ID=S2507_raw:GT=hom:DP=75:FQ=1:SQ=-1;ID=S2668_raw:GT=hom:DP=116:FQ=1:SQ=-1;ID=S2012_raw:GT=hom:DP=50:FQ=1:SQ=-1;ID=S2010_raw:GT=hom:DP=55:FQ=1:SQ=-1;ID=S2662_raw:GT=hom:DP=75:FQ=1:SQ=-1;ID=S2663_raw:GT=hom:DP=91:FQ=1:SQ=-1;ID=S2011_raw:GT=hom:DP=84:FQ=1:SQ=-1;ID=S2504_raw:GT=hom:DP=104:FQ=1:SQ=-1;ID=S2008_raw:GT=hom:DP=48:FQ=1:SQ=-1;ID=S2509_raw:GT=hom:DP=89:FQ=1:SQ=-1;ID=S2508_raw:GT=hom:DP=121:FQ=1:SQ=-1;ID=S2666_raw:GT=hom:DP=87:FQ=1:SQ=-1;ID=S2669_raw:GT=hom:DP=82:FQ=1:SQ=-1;ID=S2166_raw:GT=hom:DP=110:FQ=1:SQ=-1;ID=S1775_raw:GT=hom:DP=102:FQ=1:SQ=-1;ID=S2501_raw:GT=hom:DP=60:FQ=1:SQ=-1;ID=S2500_raw:GT=hom:DP=82:FQ=1:SQ=-1;ID=S2502_raw:GT=hom:DP=105:FQ=1:SQ=-1;ID=S2505_raw:GT=hom:DP=100:FQ=1:SQ=-1;ID=S2176_raw:GT=hom:DP=107:FQ=1:SQ=-1;ID=S2506_raw:GT=hom:DP=80:FQ=1:SQ=-1;ID=S2667_raw:GT=hom:DP=85:FQ=1:SQ=-1;
12	49439659	49439659	C	T	intronic	KMT2D	.	.	rs833818	.	ID=S2009_raw:GT=hom:DP=76:FQ=1:SQ=-1;ID=S2503_raw:GT=hom:DP=47:FQ=1:SQ=-1;ID=S2507_raw:GT=hom:DP=47:FQ=1:SQ=-1;ID=S2668_raw:GT=hom:DP=60:FQ=1:SQ=-1;ID=S2012_raw:GT=hom:DP=67:FQ=1:SQ=-1;ID=S2010_raw:GT=hom:DP=85:FQ=1:SQ=-1;ID=S2662_raw:GT=hom:DP=90:FQ=1:SQ=-1;ID=S2663_raw:GT=hom:DP=47:FQ=1:SQ=-1;ID=S2011_raw:GT=hom:DP=90:FQ=1:SQ=-1;ID=S2504_raw:GT=hom:DP=52:FQ=1:SQ=-1;ID=S2008_raw:GT=hom:DP=71:FQ=1:SQ=-1;ID=S2509_raw:GT=hom:DP=47:FQ=1:SQ=-1;ID=S2508_raw:GT=hom:DP=66:FQ=1:SQ=-1;ID=S2666_raw:GT=hom:DP=54:FQ=1:SQ=-1;ID=S2669_raw:GT=hom:DP=35:FQ=1:SQ=-1;ID=S2166_raw:GT=hom:DP=41:FQ=1:SQ=-1;ID=S1775_raw:GT=hom:DP=31:FQ=1:SQ=-1;ID=S2501_raw:GT=hom:DP=46:FQ=1:SQ=-1;ID=S2500_raw:GT=hom:DP=47:FQ=1:SQ=-1;ID=S2502_raw:GT=hom:DP=60:FQ=1:SQ=-1;ID=S2505_raw:GT=hom:DP=47:FQ=1:SQ=-1;ID=S2176_raw:GT=hom:DP=43:FQ=1:SQ=-1;ID=S2506_raw:GT=hom:DP=46:FQ=1:SQ=-1;ID=S2667_raw:GT=hom:DP=44:FQ=1:SQ=-1;
15	40937727	40937727	C	T	intronic	KNL1	.	.	rs6492959	.	ID=S2009_raw:GT=hom:DP=21:FQ=1:SQ=-1;ID=S2503_raw:GT=hom:DP=24:FQ=1:SQ=-1;ID=S2507_raw:GT=hom:DP=27:FQ=1:SQ=-1;ID=S2668_raw:GT=hom:DP=31:FQ=1:SQ=-1;ID=S2012_raw:GT=hom:DP=26:FQ=1:SQ=-1;ID=S2010_raw:GT=hom:DP=20:FQ=1:SQ=-1;ID=S2662_raw:GT=hom:DP=2:FQ=1:SQ=-1;ID=S2663_raw:GT=hom:DP=41:FQ=1:SQ=-1;ID=S2011_raw:GT=hom:DP=32:FQ=1:SQ=-1;ID=S2504_raw:GT=hom:DP=32:FQ=1:SQ=-1;ID=S2008_raw:GT=hom:DP=35:FQ=1:SQ=-1;ID=S2509_raw:GT=hom:DP=29:FQ=1:SQ=-1;ID=S2508_raw:GT=hom:DP=29:FQ=1:SQ=-1;ID=S2666_raw:GT=hom:DP=34:FQ=1:SQ=-1;ID=S2669_raw:GT=hom:DP=7:FQ=1:SQ=-1;ID=S2166_raw:GT=hom:DP=63:FQ=1:SQ=-1;ID=S1775_raw:GT=hom:DP=46:FQ=1:SQ=-1;ID=S2501_raw:GT=hom:DP=35:FQ=1:SQ=-1;ID=S2500_raw:GT=hom:DP=28:FQ=1:SQ=-1;ID=S2502_raw:GT=hom:DP=28:FQ=1:SQ=-1;ID=S2505_raw:GT=hom:DP=34:FQ=1:SQ=-1;ID=S2176_raw:GT=hom:DP=59:FQ=1:SQ=-1;ID=S2506_raw:GT=hom:DP=33:FQ=1:SQ=-1;ID=S2667_raw:GT=hom:DP=34:FQ=1:SQ=-1;
12	25368462	25368462	C	T	exonic	KRAS	synonymous SNV	KRAS:NM_033360:exon5:c.483G>A:p.R161R	rs4362222	Benign	ID=S2009_raw:GT=hom:DP=71:FQ=1:SQ=-1;ID=S2503_raw:GT=hom:DP=92:FQ=1:SQ=-1;ID=S2507_raw:GT=hom:DP=96:FQ=1:SQ=-1;ID=S2668_raw:GT=hom:DP=107:FQ=1:SQ=-1;ID=S2012_raw:GT=hom:DP=109:FQ=1:SQ=-1;ID=S2010_raw:GT=hom:DP=98:FQ=1:SQ=-1;ID=S2662_raw:GT=hom:DP=7:FQ=1:SQ=-1;ID=S2663_raw:GT=hom:DP=84:FQ=1:SQ=-1;ID=S2011_raw:GT=hom:DP=134:FQ=1:SQ=-1;ID=S2504_raw:GT=hom:DP=120:FQ=1:SQ=-1;ID=S2008_raw:GT=hom:DP=97:FQ=1:SQ=-1;ID=S2509_raw:GT=hom:DP=79:FQ=1:SQ=-1;ID=S2508_raw:GT=hom:DP=87:FQ=1:SQ=-1;ID=S2666_raw:GT=hom:DP=104:FQ=1:SQ=-1;ID=S2669_raw:GT=hom:DP=74:FQ=1:SQ=-1;ID=S2166_raw:GT=hom:DP=152:FQ=0.99:SQ=-1;ID=S1775_raw:GT=hom:DP=95:FQ=1:SQ=-1;ID=S2501_raw:GT=hom:DP=74:FQ=1:SQ=-1;ID=S2500_raw:GT=hom:DP=110:FQ=1:SQ=-1;ID=S2502_raw:GT=hom:DP=108:FQ=1:SQ=-1;ID=S2505_raw:GT=hom:DP=108:FQ=1:SQ=-1;ID=S2176_raw:GT=hom:DP=194:FQ=1:SQ=-1;ID=S2506_raw:GT=hom:DP=62:FQ=1:SQ=-1;ID=S2667_raw:GT=hom:DP=93:FQ=1:SQ=-1;
2	1,44E+08	1,44E+08	C	T	intronic	KYNU	.	.	rs6430000	.	ID=S2009_raw:GT=hom:DP=74:FQ=1:SQ=-1;ID=S2503_raw:GT=hom:DP=64:FQ=1:SQ=-1;ID=S2507_raw:GT=hom:DP=66:FQ=1:SQ=-1;ID=S2668_raw:GT=hom:DP=74:FQ=1:SQ=-1;ID=S2012_raw:GT=hom:DP=72:FQ=1:SQ=-1;ID=S2010_raw:GT=hom:DP=102:FQ=1:SQ=-1;ID=S2662_raw:GT=hom:DP=3:FQ=1:SQ=-1;ID=S2663_raw:GT=hom:DP=98:FQ=1:SQ=-1;ID=S2011_raw:GT=hom:DP=114:FQ=1:SQ=-1;ID=S2504_raw:GT=hom:DP=56:FQ=1:SQ=-1;ID=S2008_raw:GT=hom:DP=93:FQ=1:SQ=-1;ID=S2509_raw:GT=hom:DP=72:FQ=1:SQ=-1;ID=S2508_raw:GT=hom:DP=67:FQ=1:SQ=-1;ID=S2666_raw:GT=hom:DP=47:FQ=1:SQ=-1;ID=S2669_raw:GT=hom:DP=29:FQ=1:SQ=-1;ID=S2166_raw:GT=hom:DP=53:FQ=1:SQ=-1;ID=S1775_raw:GT=hom:DP=23:FQ=1:SQ=-1;ID=S2501_raw:GT=hom:DP=61:FQ=1:SQ=-1;ID=S2500_raw:GT=hom:DP=70:FQ=1:SQ=-1;ID=S2502_raw:GT=hom:DP=51:FQ=1:SQ=-1;ID=S2505_raw:GT=hom:DP=71:FQ=1:SQ=-1;ID=S2176_raw:GT=hom:DP=30:FQ=1:SQ=-1;ID=S2506_raw:GT=hom:DP=76:FQ=1:SQ=-1;ID=S2667_raw:GT=hom:DP=53:FQ=1:SQ=-1;
18	21338521	21338521	C	-	intronic	LAMA3	.	.	.	.	ID=S2009_raw:GT=hom:DP=8:FQ=1:SQ=-1;ID=S2503_raw:GT=hom:DP=36:FQ=1:SQ=-1;ID=S2507_raw:GT=hom:DP=27:FQ=1:SQ=-1;ID=S2668_raw:GT=hom:DP=41:FQ=1:SQ=-1;ID=S2012_raw:GT=hom:DP=11:FQ=1:SQ=-1;ID=S2010_raw:GT=hom:DP=15:FQ=1:SQ=-1;ID=S2662_raw:GT=hom:DP=59:FQ=1:SQ=-1;ID=S2663_raw:GT=hom:DP=55:FQ=1:SQ=-1;ID=S2011_raw:GT=hom:DP=21:FQ=1:SQ=-1;ID=S2504_raw:GT=hom:DP=42:FQ=1:SQ=-1;ID=S2008_raw:GT=hom:DP=13:FQ=1:SQ=-1;ID=S2509_raw:GT=hom:DP=42:FQ=1:SQ=-1;ID=S2508_raw:GT=hom:DP=41:FQ=1:SQ=-1;ID=S2666_raw:GT=hom:DP=39:FQ=1:SQ=-1;ID=S2669_raw:GT=hom:DP=35:FQ=1:SQ=-1;ID=S2166_raw:GT=hom:DP=27:FQ=1:SQ=-1;ID=S1775_raw:GT=hom:DP=18:FQ=1:SQ=-1;ID=S2501_raw:GT=hom:DP=35:FQ=1:SQ=-1;ID=S2500_raw:GT=hom:DP=31:FQ=1:SQ=-1;ID=S2502_raw:GT=hom:DP=28:FQ=1:SQ=-1;ID=S2505_raw:GT=hom:DP=36:FQ=1:SQ=-1;ID=S2176_raw:GT=hom:DP=28:FQ=1:SQ=-1;ID=S2506_raw:GT=hom:DP=22:FQ=1:SQ=-1;ID=S2667_raw:GT=hom:DP=29:FQ=1:SQ=-1;
18	21511089	21511089	A	G	exonic	LAMA3	nonsynonymous SNV	LAMA3:NM_001127718:exon27:c.3505A>G:p.S1169G,LAMA3:NM_000227:exon28:c.3673A>G:p.S1225G,LAMA3:NM_001127717:exon64:c.8332A>G:p.S2778G,LAMA3:NM_198129:exon65:c.8500A>G:p.S2834G	rs1154233	Benign	ID=S2009_raw:GT=hom:DP=89:FQ=1:SQ=-1;ID=S2503_raw:GT=hom:DP=108:FQ=1:SQ=-1;ID=S2507_raw:GT=hom:DP=79:FQ=1:SQ=-1;ID=S2668_raw:GT=hom:DP=113:FQ=1:SQ=-1;ID=S2012_raw:GT=hom:DP=111:FQ=1:SQ=-1;ID=S2010_raw:GT=hom:DP=101:FQ=1:SQ=-1;ID=S2662_raw:GT=hom:DP=19:FQ=1:SQ=-1;ID=S2663_raw:GT=hom:DP=57:FQ=1:SQ=-1;ID=S2011_raw:GT=hom:DP=147:FQ=0.99:SQ=-1;ID=S2504_raw:GT=hom:DP=98:FQ=1:SQ=-1;ID=S2008_raw:GT=hom:DP=128:FQ=1:SQ=-1;ID=S2509_raw:GT=hom:DP=82:FQ=1:SQ=-1;ID=S2508_raw:GT=hom:DP=100:FQ=1:SQ=-1;ID=S2666_raw:GT=hom:DP=111:FQ=1:SQ=-1;ID=S2669_raw:GT=hom:DP=72:FQ=1:SQ=-1;ID=S2166_raw:GT=hom:DP=273:FQ=1:SQ=-1;ID=S1775_raw:GT=hom:DP=202:FQ=1:SQ=-1;ID=S2501_raw:GT=hom:DP=86:FQ=1:SQ=-1;ID=S2500_raw:GT=hom:DP=93:FQ=1:SQ=-1;ID=S2502_raw:GT=hom:DP=72:FQ=1:SQ=-1;ID=S2505_raw:GT=hom:DP=101:FQ=1:SQ=-1;ID=S2176_raw:GT=hom:DP=286:FQ=1:SQ=-1;ID=S2506_raw:GT=hom:DP=61:FQ=1:SQ=-1;ID=S2667_raw:GT=hom:DP=75:FQ=1:SQ=-1;
3	45559519	45559519	T	C	exonic	LARS2	synonymous SNV	LARS2:NM_001368263:exon17:c.2169T>C:p.A723A,LARS2:NM_015340:exon18:c.2169T>C:p.A723A	rs2170549	Benign	ID=S2009_raw:GT=hom:DP=45:FQ=1:SQ=-1;ID=S2503_raw:GT=hom:DP=84:FQ=1:SQ=-1;ID=S2507_raw:GT=hom:DP=94:FQ=1:SQ=-1;ID=S2668_raw:GT=hom:DP=129:FQ=1:SQ=-1;ID=S2012_raw:GT=hom:DP=67:FQ=1:SQ=-1;ID=S2010_raw:GT=hom:DP=59:FQ=1:SQ=-1;ID=S2662_raw:GT=hom:DP=60:FQ=1:SQ=-1;ID=S2663_raw:GT=hom:DP=88:FQ=1:SQ=-1;ID=S2011_raw:GT=hom:DP=93:FQ=0.99:SQ=-1;ID=S2504_raw:GT=hom:DP=106:FQ=1:SQ=-1;ID=S2008_raw:GT=hom:DP=69:FQ=1:SQ=-1;ID=S2509_raw:GT=hom:DP=57:FQ=1:SQ=-1;ID=S2508_raw:GT=hom:DP=101:FQ=1:SQ=-1;ID=S2666_raw:GT=hom:DP=81:FQ=1:SQ=-1;ID=S2669_raw:GT=hom:DP=98:FQ=1:SQ=-1;ID=S2166_raw:GT=hom:DP=76:FQ=1:SQ=-1;ID=S1775_raw:GT=hom:DP=85:FQ=1:SQ=-1;ID=S2501_raw:GT=hom:DP=79:FQ=1:SQ=-1;ID=S2500_raw:GT=hom:DP=118:FQ=1:SQ=-1;ID=S2502_raw:GT=hom:DP=102:FQ=1:SQ=-1;ID=S2505_raw:GT=hom:DP=119:FQ=1:SQ=-1;ID=S2176_raw:GT=hom:DP=112:FQ=1:SQ=-1;ID=S2506_raw:GT=hom:DP=73:FQ=1:SQ=-1;ID=S2667_raw:GT=hom:DP=97:FQ=1:SQ=-1;
3	45565554	45565554	A	G	exonic	LARS2	synonymous SNV	LARS2:NM_001368263:exon19:c.2358A>G:p.V786V,LARS2:NM_015340:exon20:c.2358A>G:p.V786V	rs267220	Benign	ID=S2009_raw:GT=hom:DP=164:FQ=1:SQ=-1;ID=S2503_raw:GT=hom:DP=113:FQ=1:SQ=-1;ID=S2507_raw:GT=hom:DP=112:FQ=1:SQ=-1;ID=S2668_raw:GT=hom:DP=113:FQ=1:SQ=-1;ID=S2012_raw:GT=hom:DP=207:FQ=1:SQ=-1;ID=S2010_raw:GT=hom:DP=217:FQ=1:SQ=-1;ID=S2662_raw:GT=hom:DP=53:FQ=1:SQ=-1;ID=S2663_raw:GT=hom:DP=85:FQ=1:SQ=-1;ID=S2011_raw:GT=hom:DP=292:FQ=1:SQ=-1;ID=S2504_raw:GT=hom:DP=129:FQ=1:SQ=-1;ID=S2008_raw:GT=hom:DP=225:FQ=1:SQ=-1;ID=S2509_raw:GT=hom:DP=137:FQ=1:SQ=-1;ID=S2508_raw:GT=hom:DP=118:FQ=1:SQ=-1;ID=S2666_raw:GT=hom:DP=63:FQ=1:SQ=-1;ID=S2669_raw:GT=hom:DP=80:FQ=1:SQ=-1;ID=S2166_raw:GT=hom:DP=125:FQ=1:SQ=-1;ID=S1775_raw:GT=hom:DP=65:FQ=1:SQ=-1;ID=S2501_raw:GT=hom:DP=107:FQ=1:SQ=-1;ID=S2500_raw:GT=hom:DP=117:FQ=1:SQ=-1;ID=S2502_raw:GT=hom:DP=118:FQ=1:SQ=-1;ID=S2505_raw:GT=hom:DP=97:FQ=1:SQ=-1;ID=S2176_raw:GT=hom:DP=114:FQ=1:SQ=-1;ID=S2506_raw:GT=hom:DP=106:FQ=1:SQ=-1;ID=S2667_raw:GT=hom:DP=91:FQ=1:SQ=-1;
19	11221454	11221454	T	C	splicing	LDLR	.	NM_000527:exon7:c.1060+7T>C;NM_001195798:exon7:c.1060+7T>C;NM_001195799:exon6:c.937+7T>C;NM_001195800:exon5:c.556+7T>C;NM_001195803:exon6:c.679+7T>C	rs2738442	Conflicting_interpretations_of_pathogenicity	ID=S2009_raw:GT=hom:DP=119:FQ=1:SQ=-1;ID=S2503_raw:GT=hom:DP=301:FQ=1:SQ=-1;ID=S2507_raw:GT=hom:DP=270:FQ=1:SQ=-1;ID=S2668_raw:GT=hom:DP=314:FQ=1:SQ=-1;ID=S2012_raw:GT=hom:DP=125:FQ=1:SQ=-1;ID=S2010_raw:GT=hom:DP=148:FQ=1:SQ=-1;ID=S2662_raw:GT=hom:DP=414:FQ=1:SQ=-1;ID=S2663_raw:GT=hom:DP=201:FQ=1:SQ=-1;ID=S2011_raw:GT=hom:DP=163:FQ=1:SQ=-1;ID=S2504_raw:GT=hom:DP=322:FQ=1:SQ=-1;ID=S2008_raw:GT=hom:DP=170:FQ=1:SQ=-1;ID=S2509_raw:GT=hom:DP=281:FQ=1:SQ=-1;ID=S2508_raw:GT=hom:DP=294:FQ=1:SQ=-1;ID=S2666_raw:GT=hom:DP=250:FQ=1:SQ=-1;ID=S2669_raw:GT=hom:DP=233:FQ=1:SQ=-1;ID=S2166_raw:GT=hom:DP=289:FQ=1:SQ=-1;ID=S1775_raw:GT=hom:DP=247:FQ=1:SQ=-1;ID=S2501_raw:GT=hom:DP=258:FQ=1:SQ=-1;ID=S2500_raw:GT=hom:DP=243:FQ=1:SQ=-1;ID=S2502_raw:GT=hom:DP=287:FQ=1:SQ=-1;ID=S2505_raw:GT=hom:DP=261:FQ=1:SQ=-1;ID=S2176_raw:GT=hom:DP=404:FQ=1:SQ=-1;ID=S2506_raw:GT=hom:DP=256:FQ=1:SQ=-1;ID=S2667_raw:GT=hom:DP=238:FQ=1:SQ=-1;
1	25870018	25870018	C	G	upstream	LDLRAP1	.	dist=58	rs61776820	.	ID=S2009_raw:GT=hom:DP=12:FQ=1:SQ=-1;ID=S2503_raw:GT=hom:DP=8:FQ=1:SQ=-1;ID=S2507_raw:GT=hom:DP=11:FQ=1:SQ=-1;ID=S2668_raw:GT=hom:DP=7:FQ=1:SQ=-1;ID=S2012_raw:GT=hom:DP=10:FQ=1:SQ=-1;ID=S2010_raw:GT=hom:DP=6:FQ=1:SQ=-1;ID=S2662_raw:GT=hom:DP=13:FQ=1:SQ=-1;ID=S2663_raw:GT=hom:DP=14:FQ=1:SQ=-1;ID=S2011_raw:GT=hom:DP=10:FQ=1:SQ=-1;ID=S2504_raw:GT=hom:DP=3:FQ=1:SQ=-1;ID=S2008_raw:GT=hom:DP=16:FQ=1:SQ=-1;ID=S2509_raw:GT=hom:DP=11:FQ=1:SQ=-1;ID=S2508_raw:GT=hom:DP=10:FQ=1:SQ=-1;ID=S2666_raw:GT=hom:DP=4:FQ=1:SQ=-1;ID=S2669_raw:GT=hom:DP=10:FQ=1:SQ=-1;ID=S2166_raw:GT=hom:DP=13:FQ=1:SQ=-1;ID=S1775_raw:GT=hom:DP=4:FQ=1:SQ=-1;ID=S2501_raw:GT=hom:DP=8:FQ=1:SQ=-1;ID=S2500_raw:GT=hom:DP=7:FQ=1:SQ=-1;ID=S2502_raw:GT=hom:DP=8:FQ=1:SQ=-1;ID=S2505_raw:GT=hom:DP=9:FQ=1:SQ=-1;ID=S2176_raw:GT=hom:DP=4:FQ=1:SQ=-1;ID=S2506_raw:GT=hom:DP=11:FQ=1:SQ=-1;ID=S2667_raw:GT=hom:DP=17:FQ=1:SQ=-1;
4	1815990	1815990	G	A	UTR3	LETM1	.	NM_012318:c.*161C>T	rs4865477	.	ID=S2009_raw:GT=hom:DP=25:FQ=1:SQ=-1;ID=S2503_raw:GT=hom:DP=2:FQ=1:SQ=-1;ID=S2507_raw:GT=hom:DP=4:FQ=1:SQ=-1;ID=S2668_raw:GT=hom:DP=4:FQ=1:SQ=-1;ID=S2012_raw:GT=hom:DP=21:FQ=1:SQ=-1;ID=S2010_raw:GT=hom:DP=19:FQ=0.95:SQ=-1;ID=S2662_raw:GT=hom:DP=21:FQ=1:SQ=-1;ID=S2663_raw:GT=hom:DP=4:FQ=1:SQ=-1;ID=S2011_raw:GT=hom:DP=31:FQ=1:SQ=-1;ID=S2504_raw:GT=hom:DP=13:FQ=1:SQ=-1;ID=S2008_raw:GT=hom:DP=31:FQ=0.97:SQ=-1;ID=S2509_raw:GT=hom:DP=11:FQ=1:SQ=-1;ID=S2508_raw:GT=hom:DP=8:FQ=1:SQ=-1;ID=S2666_raw:GT=hom:DP=3:FQ=1:SQ=-1;ID=S2669_raw:GT=hom:DP=7:FQ=1:SQ=-1;ID=S2166_raw:GT=hom:DP=3:FQ=1:SQ=-1;ID=S1775_raw:GT=hom:DP=4:FQ=1:SQ=-1;ID=S2501_raw:GT=hom:DP=9:FQ=1:SQ=-1;ID=S2500_raw:GT=hom:DP=12:FQ=1:SQ=-1;ID=S2502_raw:GT=hom:DP=4:FQ=1:SQ=-1;ID=S2505_raw:GT=hom:DP=8:FQ=1:SQ=-1;ID=S2176_raw:GT=hom:DP=6:FQ=1:SQ=-1;ID=S2506_raw:GT=hom:DP=8:FQ=1:SQ=-1;ID=S2667_raw:GT=hom:DP=8:FQ=1:SQ=-1;
4	1818594	1818594	C	T	exonic	LETM1	synonymous SNV	LETM1:NM_012318:exon12:c.1791G>A:p.K597K	rs9328763	.	ID=S2009_raw:GT=hom:DP=52:FQ=1:SQ=-1;ID=S2503_raw:GT=hom:DP=116:FQ=1:SQ=-1;ID=S2507_raw:GT=hom:DP=136:FQ=1:SQ=-1;ID=S2668_raw:GT=hom:DP=111:FQ=1:SQ=-1;ID=S2012_raw:GT=hom:DP=63:FQ=1:SQ=-1;ID=S2010_raw:GT=hom:DP=67:FQ=1:SQ=-1;ID=S2662_raw:GT=hom:DP=156:FQ=1:SQ=-1;ID=S2663_raw:GT=hom:DP=103:FQ=1:SQ=-1;ID=S2011_raw:GT=hom:DP=102:FQ=1:SQ=-1;ID=S2504_raw:GT=hom:DP=115:FQ=1:SQ=-1;ID=S2008_raw:GT=hom:DP=70:FQ=1:SQ=-1;ID=S2509_raw:GT=hom:DP=157:FQ=1:SQ=-1;ID=S2508_raw:GT=hom:DP=167:FQ=1:SQ=-1;ID=S2666_raw:GT=hom:DP=80:FQ=1:SQ=-1;ID=S2669_raw:GT=hom:DP=109:FQ=1:SQ=-1;ID=S2166_raw:GT=hom:DP=178:FQ=1:SQ=-1;ID=S1775_raw:GT=hom:DP=112:FQ=1:SQ=-1;ID=S2501_raw:GT=hom:DP=123:FQ=1:SQ=-1;ID=S2500_raw:GT=hom:DP=130:FQ=1:SQ=-1;ID=S2502_raw:GT=hom:DP=140:FQ=1:SQ=-1;ID=S2505_raw:GT=hom:DP=119:FQ=1:SQ=-1;ID=S2176_raw:GT=hom:DP=182:FQ=1:SQ=-1;ID=S2506_raw:GT=hom:DP=143:FQ=1:SQ=-1;ID=S2667_raw:GT=hom:DP=137:FQ=1:SQ=-1;
19	48646993	48646993	C	T	intronic	LIG1	.	.	rs415465	.	ID=S2009_raw:GT=hom:DP=50:FQ=1:SQ=-1;ID=S2503_raw:GT=hom:DP=144:FQ=1:SQ=-1;ID=S2507_raw:GT=hom:DP=111:FQ=1:SQ=-1;ID=S2668_raw:GT=hom:DP=182:FQ=1:SQ=-1;ID=S2012_raw:GT=hom:DP=87:FQ=1:SQ=-1;ID=S2010_raw:GT=hom:DP=61:FQ=1:SQ=-1;ID=S2662_raw:GT=hom:DP=183:FQ=1:SQ=-1;ID=S2663_raw:GT=hom:DP=104:FQ=1:SQ=-1;ID=S2011_raw:GT=hom:DP=107:FQ=1:SQ=-1;ID=S2504_raw:GT=hom:DP=144:FQ=1:SQ=-1;ID=S2008_raw:GT=hom:DP=77:FQ=1:SQ=-1;ID=S2509_raw:GT=hom:DP=135:FQ=0.99:SQ=-1;ID=S2508_raw:GT=hom:DP=147:FQ=1:SQ=-1;ID=S2666_raw:GT=hom:DP=202:FQ=1:SQ=-1;ID=S2669_raw:GT=hom:DP=157:FQ=1:SQ=-1;ID=S2166_raw:GT=hom:DP=145:FQ=1:SQ=-1;ID=S1775_raw:GT=hom:DP=121:FQ=1:SQ=-1;ID=S2501_raw:GT=hom:DP=138:FQ=1:SQ=-1;ID=S2500_raw:GT=hom:DP=136:FQ=1:SQ=-1;ID=S2502_raw:GT=hom:DP=166:FQ=1:SQ=-1;ID=S2505_raw:GT=hom:DP=136:FQ=1:SQ=-1;ID=S2176_raw:GT=hom:DP=170:FQ=1:SQ=-1;ID=S2506_raw:GT=hom:DP=139:FQ=1:SQ=-1;ID=S2667_raw:GT=hom:DP=134:FQ=1:SQ=-1;
8	11453535	11453535	G	C	intergenic	LINC00208;GATA4	.	dist=14685;dist=80893	rs2244242	.	ID=S2009_raw:GT=hom:DP=4:FQ=1:SQ=-1;ID=S2503_raw:GT=hom:DP=2:FQ=1:SQ=-1;ID=S2507_raw:GT=hom:DP=4:FQ=1:SQ=-1;ID=S2668_raw:GT=hom:DP=7:FQ=1:SQ=-1;ID=S2012_raw:GT=hom:DP=6:FQ=1:SQ=-1;ID=S2010_raw:GT=hom:DP=6:FQ=1:SQ=-1;ID=S2662_raw:GT=hom:DP=3:FQ=1:SQ=-1;ID=S2663_raw:GT=hom:DP=1:FQ=1:SQ=-1;ID=S2011_raw:GT=hom:DP=4:FQ=1:SQ=-1;ID=S2504_raw:GT=hom:DP=1:FQ=1:SQ=-1;ID=S2008_raw:GT=hom:DP=6:FQ=1:SQ=-1;ID=S2509_raw:GT=hom:DP=2:FQ=1:SQ=-1;ID=S2508_raw:GT=hom:DP=8:FQ=1:SQ=-1;ID=S2666_raw:GT=hom:DP=13:FQ=1:SQ=-1;ID=S2669_raw:GT=hom:DP=4:FQ=1:SQ=-1;ID=S2166_raw:GT=hom:DP=7:FQ=1:SQ=-1;ID=S1775_raw:GT=hom:DP=2:FQ=1:SQ=-1;ID=S2501_raw:GT=hom:DP=1:FQ=1:SQ=-1;ID=S2500_raw:GT=hom:DP=5:FQ=1:SQ=-1;ID=S2502_raw:GT=hom:DP=6:FQ=1:SQ=-1;ID=S2505_raw:GT=hom:DP=5:FQ=1:SQ=-1;ID=S2176_raw:GT=hom:DP=8:FQ=1:SQ=-1;ID=S2506_raw:GT=hom:DP=5:FQ=1:SQ=-1;ID=S2667_raw:GT=hom:DP=1:FQ=1:SQ=-1;
1	2,04E+08	2,04E+08	C	G	intergenic	LINC00628;PPP1R15B	.	dist=7750;dist=25895	rs3014611	.	ID=S2009_raw:GT=hom:DP=1:FQ=1:SQ=-1;ID=S2503_raw:GT=hom:DP=9:FQ=1:SQ=-1;ID=S2507_raw:GT=hom:DP=4:FQ=1:SQ=-1;ID=S2668_raw:GT=hom:DP=4:FQ=1:SQ=-1;ID=S2012_raw:GT=hom:DP=1:FQ=1:SQ=-1;ID=S2010_raw:GT=hom:DP=1:FQ=1:SQ=-1;ID=S2662_raw:GT=hom:DP=7:FQ=1:SQ=-1;ID=S2663_raw:GT=hom:DP=3:FQ=1:SQ=-1;ID=S2011_raw:GT=hom:DP=2:FQ=1:SQ=-1;ID=S2504_raw:GT=hom:DP=12:FQ=1:SQ=-1;ID=S2008_raw:GT=hom:DP=3:FQ=1:SQ=-1;ID=S2509_raw:GT=hom:DP=1:FQ=1:SQ=-1;ID=S2508_raw:GT=hom:DP=6:FQ=1:SQ=-1;ID=S2666_raw:GT=hom:DP=5:FQ=1:SQ=-1;ID=S2669_raw:GT=hom:DP=6:FQ=1:SQ=-1;ID=S2166_raw:GT=hom:DP=4:FQ=1:SQ=-1;ID=S1775_raw:GT=hom:DP=4:FQ=1:SQ=-1;ID=S2501_raw:GT=hom:DP=12:FQ=1:SQ=-1;ID=S2500_raw:GT=hom:DP=2:FQ=1:SQ=-1;ID=S2502_raw:GT=hom:DP=4:FQ=1:SQ=-1;ID=S2505_raw:GT=hom:DP=8:FQ=1:SQ=-1;ID=S2176_raw:GT=hom:DP=5:FQ=1:SQ=-1;ID=S2506_raw:GT=hom:DP=3:FQ=1:SQ=-1;ID=S2667_raw:GT=hom:DP=4:FQ=1:SQ=-1;
6	8887269	8887269	A	C	intergenic	LOC100506207;TFAP2A	.	dist=101591;dist=1509641	rs9379256	.	ID=S2009_raw:GT=hom:DP=1:FQ=1:SQ=-1;ID=S2503_raw:GT=hom:DP=4:FQ=1:SQ=-1;ID=S2507_raw:GT=hom:DP=1:FQ=1:SQ=-1;ID=S2668_raw:GT=hom:DP=8:FQ=1:SQ=-1;ID=S2012_raw:GT=hom:DP=3:FQ=1:SQ=-1;ID=S2010_raw:GT=hom:DP=1:FQ=1:SQ=-1;ID=S2662_raw:GT=hom:DP=2:FQ=1:SQ=-1;ID=S2663_raw:GT=hom:DP=6:FQ=1:SQ=-1;ID=S2011_raw:GT=hom:DP=2:FQ=1:SQ=-1;ID=S2504_raw:GT=hom:DP=9:FQ=1:SQ=-1;ID=S2008_raw:GT=hom:DP=3:FQ=1:SQ=-1;ID=S2509_raw:GT=hom:DP=7:FQ=1:SQ=-1;ID=S2508_raw:GT=hom:DP=5:FQ=1:SQ=-1;ID=S2666_raw:GT=hom:DP=3:FQ=1:SQ=-1;ID=S2669_raw:GT=hom:DP=2:FQ=1:SQ=-1;ID=S2166_raw:GT=hom:DP=4:FQ=1:SQ=-1;ID=S1775_raw:GT=hom:DP=1:FQ=1:SQ=-1;ID=S2501_raw:GT=hom:DP=1:FQ=1:SQ=-1;ID=S2500_raw:GT=hom:DP=3:FQ=1:SQ=-1;ID=S2502_raw:GT=hom:DP=5:FQ=1:SQ=-1;ID=S2505_raw:GT=hom:DP=5:FQ=1:SQ=-1;ID=S2176_raw:GT=hom:DP=10:FQ=1:SQ=-1;ID=S2506_raw:GT=hom:DP=9:FQ=1:SQ=-1;ID=S2667_raw:GT=hom:DP=2:FQ=1:SQ=-1;
6	9922006	9922006	A	G	intergenic	LOC100506207;TFAP2A	.	dist=1136328;dist=474904	rs1925775	.	ID=S2009_raw:GT=hom:DP=56:FQ=1:SQ=-1;ID=S2503_raw:GT=hom:DP=36:FQ=1:SQ=-1;ID=S2507_raw:GT=hom:DP=50:FQ=1:SQ=-1;ID=S2668_raw:GT=hom:DP=85:FQ=1:SQ=-1;ID=S2012_raw:GT=hom:DP=70:FQ=1:SQ=-1;ID=S2010_raw:GT=hom:DP=71:FQ=1:SQ=-1;ID=S2662_raw:GT=hom:DP=3:FQ=1:SQ=-1;ID=S2663_raw:GT=hom:DP=42:FQ=1:SQ=-1;ID=S2011_raw:GT=hom:DP=92:FQ=1:SQ=-1;ID=S2504_raw:GT=hom:DP=63:FQ=1:SQ=-1;ID=S2008_raw:GT=hom:DP=68:FQ=1:SQ=-1;ID=S2509_raw:GT=hom:DP=63:FQ=1:SQ=-1;ID=S2508_raw:GT=hom:DP=65:FQ=1:SQ=-1;ID=S2666_raw:GT=hom:DP=91:FQ=1:SQ=-1;ID=S2669_raw:GT=hom:DP=21:FQ=1:SQ=-1;ID=S2166_raw:GT=hom:DP=15:FQ=1:SQ=-1;ID=S1775_raw:GT=hom:DP=11:FQ=1:SQ=-1;ID=S2501_raw:GT=hom:DP=51:FQ=1:SQ=-1;ID=S2500_raw:GT=hom:DP=53:FQ=1:SQ=-1;ID=S2502_raw:GT=hom:DP=56:FQ=1:SQ=-1;ID=S2505_raw:GT=hom:DP=43:FQ=1:SQ=-1;ID=S2176_raw:GT=hom:DP=16:FQ=1:SQ=-1;ID=S2506_raw:GT=hom:DP=36:FQ=1:SQ=-1;ID=S2667_raw:GT=hom:DP=68:FQ=1:SQ=-1;
22	18901004	18901004	C	T	exonic	LOC102724788;PRODH	nonsynonymous SNV	PRODH:NM_001195226:exon13:c.1238G>A:p.R413Q,LOC102724788:NM_001368250:exon13:c.1238G>A:p.R413Q,LOC102724788:NM_001368249:exon14:c.1562G>A:p.R521Q,PRODH:NM_016335:exon14:c.1562G>A:p.R521Q	rs450046	.	ID=S2009_raw:GT=hom:DP=53:FQ=1:SQ=-1;ID=S2503_raw:GT=hom:DP=111:FQ=1:SQ=-1;ID=S2507_raw:GT=hom:DP=111:FQ=1:SQ=-1;ID=S2668_raw:GT=hom:DP=144:FQ=1:SQ=-1;ID=S2012_raw:GT=hom:DP=52:FQ=1:SQ=-1;ID=S2010_raw:GT=hom:DP=45:FQ=1:SQ=-1;ID=S2662_raw:GT=hom:DP=245:FQ=1:SQ=-1;ID=S2663_raw:GT=hom:DP=109:FQ=1:SQ=-1;ID=S2011_raw:GT=hom:DP=53:FQ=1:SQ=-1;ID=S2504_raw:GT=hom:DP=138:FQ=1:SQ=-1;ID=S2008_raw:GT=hom:DP=75:FQ=1:SQ=-1;ID=S2509_raw:GT=hom:DP=162:FQ=1:SQ=-1;ID=S2508_raw:GT=hom:DP=174:FQ=1:SQ=-1;ID=S2666_raw:GT=hom:DP=129:FQ=1:SQ=-1;ID=S2669_raw:GT=hom:DP=94:FQ=1:SQ=-1;ID=S2166_raw:GT=hom:DP=134:FQ=1:SQ=-1;ID=S1775_raw:GT=hom:DP=85:FQ=1:SQ=-1;ID=S2501_raw:GT=hom:DP=136:FQ=1:SQ=-1;ID=S2500_raw:GT=hom:DP=132:FQ=1:SQ=-1;ID=S2502_raw:GT=hom:DP=142:FQ=0.99:SQ=-1;ID=S2505_raw:GT=hom:DP=118:FQ=0.99:SQ=-1;ID=S2176_raw:GT=hom:DP=133:FQ=1:SQ=-1;ID=S2506_raw:GT=hom:DP=120:FQ=1:SQ=-1;ID=S2667_raw:GT=hom:DP=150:FQ=0.99:SQ=-1;
1	1,14E+08	1,14E+08	T	C	intronic	LRIG2	.	.	rs1216802	.	ID=S2009_raw:GT=hom:DP=14:FQ=1:SQ=-1;ID=S2503_raw:GT=hom:DP=10:FQ=1:SQ=-1;ID=S2507_raw:GT=hom:DP=8:FQ=1:SQ=-1;ID=S2668_raw:GT=hom:DP=5:FQ=1:SQ=-1;ID=S2012_raw:GT=hom:DP=17:FQ=1:SQ=-1;ID=S2010_raw:GT=hom:DP=14:FQ=1:SQ=-1;ID=S2662_raw:GT=hom:DP=1:FQ=1:SQ=-1;ID=S2663_raw:GT=hom:DP=6:FQ=1:SQ=-1;ID=S2011_raw:GT=hom:DP=13:FQ=1:SQ=-1;ID=S2504_raw:GT=hom:DP=6:FQ=1:SQ=-1;ID=S2008_raw:GT=hom:DP=22:FQ=1:SQ=-1;ID=S2509_raw:GT=hom:DP=6:FQ=1:SQ=-1;ID=S2508_raw:GT=hom:DP=15:FQ=1:SQ=-1;ID=S2666_raw:GT=hom:DP=1:FQ=1:SQ=-1;ID=S2669_raw:GT=hom:DP=8:FQ=1:SQ=-1;ID=S2166_raw:GT=hom:DP=35:FQ=1:SQ=-1;ID=S1775_raw:GT=hom:DP=23:FQ=1:SQ=-1;ID=S2501_raw:GT=hom:DP=9:FQ=1:SQ=-1;ID=S2500_raw:GT=hom:DP=4:FQ=1:SQ=-1;ID=S2502_raw:GT=hom:DP=16:FQ=1:SQ=-1;ID=S2505_raw:GT=hom:DP=14:FQ=1:SQ=-1;ID=S2176_raw:GT=hom:DP=32:FQ=1:SQ=-1;ID=S2506_raw:GT=hom:DP=3:FQ=1:SQ=-1;ID=S2667_raw:GT=hom:DP=8:FQ=1:SQ=-1;
1	1,14E+08	1,14E+08	A	C	exonic	LRIG2	synonymous SNV	LRIG2:NM_014813:exon15:c.2124A>C:p.T708T,LRIG2:NM_001312686:exon16:c.1815A>C:p.T605T	rs1216793	.	ID=S2009_raw:GT=hom:DP=74:FQ=1:SQ=-1;ID=S2503_raw:GT=hom:DP=153:FQ=1:SQ=-1;ID=S2507_raw:GT=hom:DP=109:FQ=1:SQ=-1;ID=S2668_raw:GT=hom:DP=128:FQ=1:SQ=-1;ID=S2012_raw:GT=hom:DP=86:FQ=1:SQ=-1;ID=S2010_raw:GT=hom:DP=106:FQ=1:SQ=-1;ID=S2662_raw:GT=hom:DP=69:FQ=1:SQ=-1;ID=S2663_raw:GT=hom:DP=103:FQ=1:SQ=-1;ID=S2011_raw:GT=hom:DP=112:FQ=1:SQ=-1;ID=S2504_raw:GT=hom:DP=130:FQ=1:SQ=-1;ID=S2008_raw:GT=hom:DP=96:FQ=0.99:SQ=-1;ID=S2509_raw:GT=hom:DP=127:FQ=1:SQ=-1;ID=S2508_raw:GT=hom:DP=117:FQ=1:SQ=-1;ID=S2666_raw:GT=hom:DP=126:FQ=1:SQ=-1;ID=S2669_raw:GT=hom:DP=102:FQ=1:SQ=-1;ID=S2166_raw:GT=hom:DP=146:FQ=1:SQ=-1;ID=S1775_raw:GT=hom:DP=125:FQ=1:SQ=-1;ID=S2501_raw:GT=hom:DP=105:FQ=1:SQ=-1;ID=S2500_raw:GT=hom:DP=149:FQ=1:SQ=-1;ID=S2502_raw:GT=hom:DP=128:FQ=1:SQ=-1;ID=S2505_raw:GT=hom:DP=133:FQ=1:SQ=-1;ID=S2176_raw:GT=hom:DP=160:FQ=0.99:SQ=-1;ID=S2506_raw:GT=hom:DP=91:FQ=1:SQ=-1;ID=S2667_raw:GT=hom:DP=111:FQ=1:SQ=-1;
2	1,7E+08	1,7E+08	T	C	intronic	LRP2	.	.	rs1559013	Benign	ID=S2009_raw:GT=hom:DP=23:FQ=1:SQ=-1;ID=S2503_raw:GT=hom:DP=34:FQ=1:SQ=-1;ID=S2507_raw:GT=hom:DP=34:FQ=1:SQ=-1;ID=S2668_raw:GT=hom:DP=20:FQ=1:SQ=-1;ID=S2012_raw:GT=hom:DP=14:FQ=0.93:SQ=-1;ID=S2010_raw:GT=hom:DP=33:FQ=1:SQ=-1;ID=S2662_raw:GT=hom:DP=71:FQ=1:SQ=-1;ID=S2663_raw:GT=hom:DP=42:FQ=1:SQ=-1;ID=S2011_raw:GT=hom:DP=26:FQ=1:SQ=-1;ID=S2504_raw:GT=hom:DP=34:FQ=1:SQ=-1;ID=S2008_raw:GT=hom:DP=24:FQ=1:SQ=-1;ID=S2509_raw:GT=hom:DP=37:FQ=1:SQ=-1;ID=S2508_raw:GT=hom:DP=34:FQ=1:SQ=-1;ID=S2666_raw:GT=hom:DP=17:FQ=1:SQ=-1;ID=S2669_raw:GT=hom:DP=37:FQ=1:SQ=-1;ID=S2166_raw:GT=hom:DP=37:FQ=1:SQ=-1;ID=S1775_raw:GT=hom:DP=41:FQ=1:SQ=-1;ID=S2501_raw:GT=hom:DP=31:FQ=1:SQ=-1;ID=S2500_raw:GT=hom:DP=33:FQ=1:SQ=-1;ID=S2502_raw:GT=hom:DP=40:FQ=1:SQ=-1;ID=S2505_raw:GT=hom:DP=29:FQ=1:SQ=-1;ID=S2176_raw:GT=hom:DP=30:FQ=1:SQ=-1;ID=S2506_raw:GT=hom:DP=33:FQ=1:SQ=-1;ID=S2667_raw:GT=hom:DP=46:FQ=1:SQ=-1;
2	1,7E+08	1,7E+08	C	G	exonic	LRP2	synonymous SNV	LRP2:NM_004525:exon1:c.63G>C:p.A21A	rs1559014	Benign	ID=S2009_raw:GT=hom:DP=37:FQ=1:SQ=-1;ID=S2503_raw:GT=hom:DP=46:FQ=1:SQ=-1;ID=S2507_raw:GT=hom:DP=49:FQ=1:SQ=-1;ID=S2668_raw:GT=hom:DP=37:FQ=1:SQ=-1;ID=S2012_raw:GT=hom:DP=27:FQ=1:SQ=-1;ID=S2010_raw:GT=hom:DP=57:FQ=1:SQ=-1;ID=S2662_raw:GT=hom:DP=106:FQ=1:SQ=-1;ID=S2663_raw:GT=hom:DP=59:FQ=1:SQ=-1;ID=S2011_raw:GT=hom:DP=43:FQ=1:SQ=-1;ID=S2504_raw:GT=hom:DP=50:FQ=1:SQ=-1;ID=S2008_raw:GT=hom:DP=43:FQ=1:SQ=-1;ID=S2509_raw:GT=hom:DP=52:FQ=1:SQ=-1;ID=S2508_raw:GT=hom:DP=51:FQ=1:SQ=-1;ID=S2666_raw:GT=hom:DP=29:FQ=1:SQ=-1;ID=S2669_raw:GT=hom:DP=69:FQ=1:SQ=-1;ID=S2166_raw:GT=hom:DP=59:FQ=1:SQ=-1;ID=S1775_raw:GT=hom:DP=56:FQ=1:SQ=-1;ID=S2501_raw:GT=hom:DP=41:FQ=1:SQ=-1;ID=S2500_raw:GT=hom:DP=49:FQ=1:SQ=-1;ID=S2502_raw:GT=hom:DP=59:FQ=1:SQ=-1;ID=S2505_raw:GT=hom:DP=46:FQ=1:SQ=-1;ID=S2176_raw:GT=hom:DP=44:FQ=1:SQ=-1;ID=S2506_raw:GT=hom:DP=55:FQ=1:SQ=-1;ID=S2667_raw:GT=hom:DP=62:FQ=1:SQ=-1;
2	1,7E+08	1,7E+08	C	T	intergenic	LRP2;BBS5	.	dist=1771;dist=115189	rs4668138	.	ID=S2009_raw:GT=hom:DP=1:FQ=1:SQ=-1;ID=S2503_raw:GT=hom:DP=3:FQ=1:SQ=-1;ID=S2507_raw:GT=hom:DP=4:FQ=1:SQ=-1;ID=S2668_raw:GT=hom:DP=9:FQ=1:SQ=-1;ID=S2012_raw:GT=hom:DP=2:FQ=1:SQ=-1;ID=S2010_raw:GT=hom:DP=2:FQ=1:SQ=-1;ID=S2662_raw:GT=hom:DP=6:FQ=1:SQ=-1;ID=S2663_raw:GT=hom:DP=13:FQ=1:SQ=-1;ID=S2011_raw:GT=hom:DP=5:FQ=1:SQ=-1;ID=S2504_raw:GT=hom:DP=10:FQ=1:SQ=-1;ID=S2008_raw:GT=hom:DP=1:FQ=1:SQ=-1;ID=S2509_raw:GT=hom:DP=3:FQ=1:SQ=-1;ID=S2508_raw:GT=hom:DP=4:FQ=1:SQ=-1;ID=S2666_raw:GT=hom:DP=2:FQ=1:SQ=-1;ID=S2669_raw:GT=hom:DP=6:FQ=1:SQ=-1;ID=S2166_raw:GT=hom:DP=15:FQ=1:SQ=-1;ID=S1775_raw:GT=hom:DP=5:FQ=1:SQ=-1;ID=S2501_raw:GT=hom:DP=5:FQ=1:SQ=-1;ID=S2500_raw:GT=hom:DP=2:FQ=1:SQ=-1;ID=S2502_raw:GT=hom:DP=5:FQ=1:SQ=-1;ID=S2505_raw:GT=hom:DP=8:FQ=1:SQ=-1;ID=S2176_raw:GT=hom:DP=7:FQ=1:SQ=-1;ID=S2506_raw:GT=hom:DP=5:FQ=1:SQ=-1;ID=S2667_raw:GT=hom:DP=6:FQ=1:SQ=-1;
11	46914598	46914598	A	G	exonic	LRP4	synonymous SNV	LRP4:NM_002334:exon13:c.1623T>C:p.D541D	rs10769215	Benign	ID=S2009_raw:GT=hom:DP=39:FQ=1:SQ=-1;ID=S2503_raw:GT=hom:DP=63:FQ=1:SQ=-1;ID=S2507_raw:GT=hom:DP=83:FQ=1:SQ=-1;ID=S2668_raw:GT=hom:DP=97:FQ=1:SQ=-1;ID=S2012_raw:GT=hom:DP=49:FQ=1:SQ=-1;ID=S2010_raw:GT=hom:DP=49:FQ=1:SQ=-1;ID=S2662_raw:GT=hom:DP=141:FQ=1:SQ=-1;ID=S2663_raw:GT=hom:DP=95:FQ=1:SQ=-1;ID=S2011_raw:GT=hom:DP=65:FQ=1:SQ=-1;ID=S2504_raw:GT=hom:DP=99:FQ=1:SQ=-1;ID=S2008_raw:GT=hom:DP=51:FQ=1:SQ=-1;ID=S2509_raw:GT=hom:DP=98:FQ=1:SQ=-1;ID=S2508_raw:GT=hom:DP=105:FQ=1:SQ=-1;ID=S2666_raw:GT=hom:DP=56:FQ=1:SQ=-1;ID=S2669_raw:GT=hom:DP=92:FQ=1:SQ=-1;ID=S2166_raw:GT=hom:DP=81:FQ=1:SQ=-1;ID=S1775_raw:GT=hom:DP=62:FQ=1:SQ=-1;ID=S2501_raw:GT=hom:DP=74:FQ=1:SQ=-1;ID=S2500_raw:GT=hom:DP=69:FQ=1:SQ=-1;ID=S2502_raw:GT=hom:DP=95:FQ=1:SQ=-1;ID=S2505_raw:GT=hom:DP=78:FQ=1:SQ=-1;ID=S2176_raw:GT=hom:DP=80:FQ=1:SQ=-1;ID=S2506_raw:GT=hom:DP=75:FQ=1:SQ=-1;ID=S2667_raw:GT=hom:DP=85:FQ=1:SQ=-1;
11	68131167	68131167	A	T	intronic	LRP5	.	.	rs10791978	.	ID=S2009_raw:GT=hom:DP=28:FQ=1:SQ=-1;ID=S2503_raw:GT=hom:DP=35:FQ=1:SQ=-1;ID=S2507_raw:GT=hom:DP=25:FQ=1:SQ=-1;ID=S2668_raw:GT=hom:DP=49:FQ=1:SQ=-1;ID=S2012_raw:GT=hom:DP=23:FQ=1:SQ=-1;ID=S2010_raw:GT=hom:DP=27:FQ=1:SQ=-1;ID=S2662_raw:GT=hom:DP=88:FQ=1:SQ=-1;ID=S2663_raw:GT=hom:DP=39:FQ=1:SQ=-1;ID=S2011_raw:GT=hom:DP=29:FQ=1:SQ=-1;ID=S2504_raw:GT=hom:DP=40:FQ=1:SQ=-1;ID=S2008_raw:GT=hom:DP=24:FQ=1:SQ=-1;ID=S2509_raw:GT=hom:DP=37:FQ=1:SQ=-1;ID=S2508_raw:GT=hom:DP=37:FQ=0.97:SQ=-1;ID=S2666_raw:GT=hom:DP=44:FQ=1:SQ=-1;ID=S2669_raw:GT=hom:DP=27:FQ=1:SQ=-1;ID=S2166_raw:GT=hom:DP=38:FQ=1:SQ=-1;ID=S1775_raw:GT=hom:DP=37:FQ=1:SQ=-1;ID=S2501_raw:GT=hom:DP=39:FQ=1:SQ=-1;ID=S2500_raw:GT=hom:DP=41:FQ=1:SQ=-1;ID=S2502_raw:GT=hom:DP=33:FQ=1:SQ=-1;ID=S2505_raw:GT=hom:DP=30:FQ=1:SQ=-1;ID=S2176_raw:GT=hom:DP=34:FQ=1:SQ=-1;ID=S2506_raw:GT=hom:DP=38:FQ=1:SQ=-1;ID=S2667_raw:GT=hom:DP=52:FQ=1:SQ=-1;
11	68170825	68170825	A	G	intronic	LRP5	.	.	rs687482	.	ID=S2009_raw:GT=hom:DP=12:FQ=1:SQ=-1;ID=S2503_raw:GT=hom:DP=26:FQ=1:SQ=-1;ID=S2507_raw:GT=hom:DP=22:FQ=1:SQ=-1;ID=S2668_raw:GT=hom:DP=26:FQ=1:SQ=-1;ID=S2012_raw:GT=hom:DP=22:FQ=1:SQ=-1;ID=S2010_raw:GT=hom:DP=19:FQ=1:SQ=-1;ID=S2662_raw:GT=hom:DP=21:FQ=1:SQ=-1;ID=S2663_raw:GT=hom:DP=12:FQ=1:SQ=-1;ID=S2011_raw:GT=hom:DP=27:FQ=1:SQ=-1;ID=S2504_raw:GT=hom:DP=20:FQ=1:SQ=-1;ID=S2008_raw:GT=hom:DP=19:FQ=1:SQ=-1;ID=S2509_raw:GT=hom:DP=7:FQ=1:SQ=-1;ID=S2508_raw:GT=hom:DP=20:FQ=1:SQ=-1;ID=S2666_raw:GT=hom:DP=20:FQ=1:SQ=-1;ID=S2669_raw:GT=hom:DP=13:FQ=1:SQ=-1;ID=S2166_raw:GT=hom:DP=23:FQ=1:SQ=-1;ID=S1775_raw:GT=hom:DP=9:FQ=1:SQ=-1;ID=S2501_raw:GT=hom:DP=27:FQ=1:SQ=-1;ID=S2500_raw:GT=hom:DP=22:FQ=1:SQ=-1;ID=S2502_raw:GT=hom:DP=24:FQ=1:SQ=-1;ID=S2505_raw:GT=hom:DP=23:FQ=1:SQ=-1;ID=S2176_raw:GT=hom:DP=14:FQ=1:SQ=-1;ID=S2506_raw:GT=hom:DP=19:FQ=1:SQ=-1;ID=S2667_raw:GT=hom:DP=12:FQ=1:SQ=-1;
2	33585796	33585796	T	C	exonic	LTBP1	nonsynonymous SNV	LTBP1:NM_001166264:exon22:c.3029T>C:p.V1010A,LTBP1:NM_001166266:exon22:c.2870T>C:p.V957A,LTBP1:NM_000627:exon23:c.3155T>C:p.V1052A,LTBP1:NM_001166265:exon23:c.2996T>C:p.V999A,LTBP1:NM_206943:exon27:c.4133T>C:p.V1378A	rs4422143	.	ID=S2009_raw:GT=hom:DP=39:FQ=1:SQ=-1;ID=S2503_raw:GT=hom:DP=202:FQ=1:SQ=-1;ID=S2507_raw:GT=hom:DP=204:FQ=1:SQ=-1;ID=S2668_raw:GT=hom:DP=314:FQ=1:SQ=-1;ID=S2012_raw:GT=hom:DP=53:FQ=1:SQ=-1;ID=S2010_raw:GT=hom:DP=77:FQ=1:SQ=-1;ID=S2662_raw:GT=hom:DP=78:FQ=1:SQ=-1;ID=S2663_raw:GT=hom:DP=191:FQ=1:SQ=-1;ID=S2011_raw:GT=hom:DP=74:FQ=1:SQ=-1;ID=S2504_raw:GT=hom:DP=194:FQ=1:SQ=-1;ID=S2008_raw:GT=hom:DP=57:FQ=1:SQ=-1;ID=S2509_raw:GT=hom:DP=173:FQ=1:SQ=-1;ID=S2508_raw:GT=hom:DP=177:FQ=1:SQ=-1;ID=S2666_raw:GT=hom:DP=379:FQ=1:SQ=-1;ID=S2669_raw:GT=hom:DP=214:FQ=1:SQ=-1;ID=S2166_raw:GT=hom:DP=154:FQ=1:SQ=-1;ID=S1775_raw:GT=hom:DP=171:FQ=1:SQ=-1;ID=S2501_raw:GT=hom:DP=200:FQ=1:SQ=-1;ID=S2500_raw:GT=hom:DP=198:FQ=1:SQ=-1;ID=S2502_raw:GT=hom:DP=213:FQ=1:SQ=-1;ID=S2505_raw:GT=hom:DP=174:FQ=1:SQ=-1;ID=S2176_raw:GT=hom:DP=195:FQ=1:SQ=-1;ID=S2506_raw:GT=hom:DP=160:FQ=1:SQ=-1;ID=S2667_raw:GT=hom:DP=197:FQ=1:SQ=-1;
19	41123095	41123095	-	G	exonic;splicing	LTBP4	unknown	UNKNOWN	.	.	ID=S2009_raw:GT=hom:DP=28:FQ=1:SQ=-1;ID=S2503_raw:GT=hom:DP=102:FQ=1:SQ=-1;ID=S2507_raw:GT=hom:DP=115:FQ=1:SQ=-1;ID=S2668_raw:GT=hom:DP=132:FQ=1:SQ=-1;ID=S2012_raw:GT=hom:DP=21:FQ=1:SQ=-1;ID=S2010_raw:GT=hom:DP=21:FQ=1:SQ=-1;ID=S2662_raw:GT=hom:DP=227:FQ=1:SQ=-1;ID=S2663_raw:GT=hom:DP=97:FQ=1:SQ=-1;ID=S2011_raw:GT=hom:DP=29:FQ=1:SQ=-1;ID=S2504_raw:GT=hom:DP=116:FQ=1:SQ=-1;ID=S2008_raw:GT=hom:DP=36:FQ=1:SQ=-1;ID=S2509_raw:GT=hom:DP=92:FQ=1:SQ=-1;ID=S2508_raw:GT=hom:DP=164:FQ=1:SQ=-1;ID=S2666_raw:GT=hom:DP=127:FQ=1:SQ=-1;ID=S2669_raw:GT=hom:DP=113:FQ=1:SQ=-1;ID=S2166_raw:GT=hom:DP=111:FQ=1:SQ=-1;ID=S1775_raw:GT=hom:DP=81:FQ=1:SQ=-1;ID=S2501_raw:GT=hom:DP=90:FQ=1:SQ=-1;ID=S2500_raw:GT=hom:DP=106:FQ=1:SQ=-1;ID=S2502_raw:GT=hom:DP=145:FQ=1:SQ=-1;ID=S2505_raw:GT=hom:DP=98:FQ=1:SQ=-1;ID=S2176_raw:GT=hom:DP=142:FQ=1:SQ=-1;ID=S2506_raw:GT=hom:DP=118:FQ=1:SQ=-1;ID=S2667_raw:GT=hom:DP=134:FQ=1:SQ=-1;
15	66735551	66735551	C	T	intronic	MAP2K1	.	.	rs6494573	.	ID=S2009_raw:GT=hom:DP=6:FQ=1:SQ=-1;ID=S2503_raw:GT=hom:DP=24:FQ=1:SQ=-1;ID=S2507_raw:GT=hom:DP=39:FQ=1:SQ=-1;ID=S2668_raw:GT=hom:DP=31:FQ=1:SQ=-1;ID=S2012_raw:GT=hom:DP=6:FQ=1:SQ=-1;ID=S2010_raw:GT=hom:DP=5:FQ=1:SQ=-1;ID=S2662_raw:GT=hom:DP=3:FQ=1:SQ=-1;ID=S2663_raw:GT=hom:DP=31:FQ=1:SQ=-1;ID=S2011_raw:GT=hom:DP=9:FQ=1:SQ=-1;ID=S2504_raw:GT=hom:DP=43:FQ=1:SQ=-1;ID=S2008_raw:GT=hom:DP=1:FQ=1:SQ=-1;ID=S2509_raw:GT=hom:DP=54:FQ=1:SQ=-1;ID=S2508_raw:GT=hom:DP=39:FQ=1:SQ=-1;ID=S2666_raw:GT=hom:DP=31:FQ=1:SQ=-1;ID=S2669_raw:GT=hom:DP=40:FQ=1:SQ=-1;ID=S2166_raw:GT=hom:DP=83:FQ=1:SQ=-1;ID=S1775_raw:GT=hom:DP=48:FQ=0.98:SQ=-1;ID=S2501_raw:GT=hom:DP=19:FQ=1:SQ=-1;ID=S2500_raw:GT=hom:DP=35:FQ=1:SQ=-1;ID=S2502_raw:GT=hom:DP=25:FQ=1:SQ=-1;ID=S2505_raw:GT=hom:DP=42:FQ=1:SQ=-1;ID=S2176_raw:GT=hom:DP=80:FQ=1:SQ=-1;ID=S2506_raw:GT=hom:DP=37:FQ=1:SQ=-1;ID=S2667_raw:GT=hom:DP=41:FQ=1:SQ=-1;
3	1,87E+08	1,87E+08	C	T	intronic	MASP1	.	.	rs850318	.	ID=S2009_raw:GT=hom:DP=30:FQ=1:SQ=-1;ID=S2503_raw:GT=hom:DP=46:FQ=1:SQ=-1;ID=S2507_raw:GT=hom:DP=52:FQ=1:SQ=-1;ID=S2668_raw:GT=hom:DP=41:FQ=1:SQ=-1;ID=S2012_raw:GT=hom:DP=13:FQ=1:SQ=-1;ID=S2010_raw:GT=hom:DP=26:FQ=1:SQ=-1;ID=S2662_raw:GT=hom:DP=37:FQ=1:SQ=-1;ID=S2663_raw:GT=hom:DP=35:FQ=1:SQ=-1;ID=S2011_raw:GT=hom:DP=38:FQ=1:SQ=-1;ID=S2504_raw:GT=hom:DP=50:FQ=1:SQ=-1;ID=S2008_raw:GT=hom:DP=23:FQ=1:SQ=-1;ID=S2509_raw:GT=hom:DP=37:FQ=1:SQ=-1;ID=S2508_raw:GT=hom:DP=60:FQ=1:SQ=-1;ID=S2666_raw:GT=hom:DP=33:FQ=1:SQ=-1;ID=S2669_raw:GT=hom:DP=70:FQ=1:SQ=-1;ID=S2166_raw:GT=hom:DP=17:FQ=1:SQ=-1;ID=S1775_raw:GT=hom:DP=15:FQ=1:SQ=-1;ID=S2501_raw:GT=hom:DP=38:FQ=1:SQ=-1;ID=S2500_raw:GT=hom:DP=35:FQ=1:SQ=-1;ID=S2502_raw:GT=hom:DP=58:FQ=1:SQ=-1;ID=S2505_raw:GT=hom:DP=40:FQ=1:SQ=-1;ID=S2176_raw:GT=hom:DP=10:FQ=1:SQ=-1;ID=S2506_raw:GT=hom:DP=35:FQ=1:SQ=-1;ID=S2667_raw:GT=hom:DP=59:FQ=1:SQ=-1;
3	1,87E+08	1,87E+08	A	G	exonic	MASP1	synonymous SNV	MASP1:NM_139125:exon11:c.1374T>C:p.P458P	rs710452	.	ID=S2009_raw:GT=hom:DP=67:FQ=1:SQ=-1;ID=S2503_raw:GT=hom:DP=189:FQ=1:SQ=-1;ID=S2507_raw:GT=hom:DP=228:FQ=1:SQ=-1;ID=S2668_raw:GT=hom:DP=251:FQ=1:SQ=-1;ID=S2012_raw:GT=hom:DP=88:FQ=1:SQ=-1;ID=S2010_raw:GT=hom:DP=100:FQ=1:SQ=-1;ID=S2662_raw:GT=hom:DP=252:FQ=1:SQ=-1;ID=S2663_raw:GT=hom:DP=225:FQ=1:SQ=-1;ID=S2011_raw:GT=hom:DP=119:FQ=1:SQ=-1;ID=S2504_raw:GT=hom:DP=212:FQ=1:SQ=-1;ID=S2008_raw:GT=hom:DP=73:FQ=1:SQ=-1;ID=S2509_raw:GT=hom:DP=215:FQ=1:SQ=-1;ID=S2508_raw:GT=hom:DP=244:FQ=1:SQ=-1;ID=S2666_raw:GT=hom:DP=179:FQ=1:SQ=-1;ID=S2669_raw:GT=hom:DP=213:FQ=1:SQ=-1;ID=S2166_raw:GT=hom:DP=323:FQ=1:SQ=-1;ID=S1775_raw:GT=hom:DP=219:FQ=1:SQ=-1;ID=S2501_raw:GT=hom:DP=191:FQ=1:SQ=-1;ID=S2500_raw:GT=hom:DP=222:FQ=1:SQ=-1;ID=S2502_raw:GT=hom:DP=234:FQ=1:SQ=-1;ID=S2505_raw:GT=hom:DP=234:FQ=1:SQ=-1;ID=S2176_raw:GT=hom:DP=298:FQ=1:SQ=-1;ID=S2506_raw:GT=hom:DP=174:FQ=1:SQ=-1;ID=S2667_raw:GT=hom:DP=208:FQ=1:SQ=-1;
3	1,87E+08	1,87E+08	A	G	intronic	MASP1	.	.	rs710460	.	ID=S2009_raw:GT=hom:DP=16:FQ=1:SQ=-1;ID=S2503_raw:GT=hom:DP=35:FQ=1:SQ=-1;ID=S2507_raw:GT=hom:DP=19:FQ=1:SQ=-1;ID=S2668_raw:GT=hom:DP=20:FQ=1:SQ=-1;ID=S2012_raw:GT=hom:DP=19:FQ=1:SQ=-1;ID=S2010_raw:GT=hom:DP=13:FQ=1:SQ=-1;ID=S2662_raw:GT=hom:DP=6:FQ=1:SQ=-1;ID=S2663_raw:GT=hom:DP=21:FQ=1:SQ=-1;ID=S2011_raw:GT=hom:DP=15:FQ=1:SQ=-1;ID=S2504_raw:GT=hom:DP=22:FQ=1:SQ=-1;ID=S2008_raw:GT=hom:DP=16:FQ=1:SQ=-1;ID=S2509_raw:GT=hom:DP=24:FQ=1:SQ=-1;ID=S2508_raw:GT=hom:DP=25:FQ=1:SQ=-1;ID=S2666_raw:GT=hom:DP=14:FQ=1:SQ=-1;ID=S2669_raw:GT=hom:DP=19:FQ=1:SQ=-1;ID=S2166_raw:GT=hom:DP=28:FQ=1:SQ=-1;ID=S1775_raw:GT=hom:DP=18:FQ=1:SQ=-1;ID=S2501_raw:GT=hom:DP=21:FQ=1:SQ=-1;ID=S2500_raw:GT=hom:DP=27:FQ=1:SQ=-1;ID=S2502_raw:GT=hom:DP=35:FQ=1:SQ=-1;ID=S2505_raw:GT=hom:DP=25:FQ=1:SQ=-1;ID=S2176_raw:GT=hom:DP=17:FQ=1:SQ=-1;ID=S2506_raw:GT=hom:DP=10:FQ=1:SQ=-1;ID=S2667_raw:GT=hom:DP=17:FQ=1:SQ=-1;
5	1,12E+08	1,12E+08	A	G	intronic	MCC	.	.	rs4705534	.	ID=S2009_raw:GT=hom:DP=1:FQ=1:SQ=-1;ID=S2503_raw:GT=hom:DP=6:FQ=1:SQ=-1;ID=S2507_raw:GT=hom:DP=2:FQ=1:SQ=-1;ID=S2668_raw:GT=hom:DP=1:FQ=1:SQ=-1;ID=S2012_raw:GT=hom:DP=3:FQ=1:SQ=-1;ID=S2010_raw:GT=hom:DP=2:FQ=1:SQ=-1;ID=S2662_raw:GT=hom:DP=6:FQ=1:SQ=-1;ID=S2663_raw:GT=hom:DP=5:FQ=1:SQ=-1;ID=S2011_raw:GT=hom:DP=2:FQ=1:SQ=-1;ID=S2504_raw:GT=hom:DP=3:FQ=1:SQ=-1;ID=S2008_raw:GT=hom:DP=1:FQ=1:SQ=-1;ID=S2509_raw:GT=hom:DP=4:FQ=1:SQ=-1;ID=S2508_raw:GT=hom:DP=2:FQ=1:SQ=-1;ID=S2666_raw:GT=hom:DP=1:FQ=1:SQ=-1;ID=S2669_raw:GT=hom:DP=5:FQ=1:SQ=-1;ID=S2166_raw:GT=hom:DP=4:FQ=1:SQ=-1;ID=S1775_raw:GT=hom:DP=1:FQ=1:SQ=-1;ID=S2501_raw:GT=hom:DP=2:FQ=1:SQ=-1;ID=S2500_raw:GT=hom:DP=7:FQ=1:SQ=-1;ID=S2502_raw:GT=hom:DP=6:FQ=1:SQ=-1;ID=S2505_raw:GT=hom:DP=4:FQ=1:SQ=-1;ID=S2176_raw:GT=hom:DP=5:FQ=1:SQ=-1;ID=S2506_raw:GT=hom:DP=6:FQ=1:SQ=-1;ID=S2667_raw:GT=hom:DP=4:FQ=1:SQ=-1;
5	1,12E+08	1,12E+08	T	C	intronic	MCC	.	.	rs4295378	.	ID=S2009_raw:GT=hom:DP=2:FQ=1:SQ=-1;ID=S2503_raw:GT=hom:DP=17:FQ=1:SQ=-1;ID=S2507_raw:GT=hom:DP=22:FQ=1:SQ=-1;ID=S2668_raw:GT=hom:DP=20:FQ=1:SQ=-1;ID=S2012_raw:GT=hom:DP=2:FQ=1:SQ=-1;ID=S2010_raw:GT=hom:DP=7:FQ=1:SQ=-1;ID=S2662_raw:GT=hom:DP=16:FQ=1:SQ=-1;ID=S2663_raw:GT=hom:DP=21:FQ=1:SQ=-1;ID=S2011_raw:GT=hom:DP=1:FQ=1:SQ=-1;ID=S2504_raw:GT=hom:DP=27:FQ=1:SQ=-1;ID=S2008_raw:GT=hom:DP=2:FQ=1:SQ=-1;ID=S2509_raw:GT=hom:DP=13:FQ=1:SQ=-1;ID=S2508_raw:GT=hom:DP=15:FQ=1:SQ=-1;ID=S2666_raw:GT=hom:DP=22:FQ=1:SQ=-1;ID=S2669_raw:GT=hom:DP=11:FQ=1:SQ=-1;ID=S2166_raw:GT=hom:DP=47:FQ=1:SQ=-1;ID=S1775_raw:GT=hom:DP=17:FQ=1:SQ=-1;ID=S2501_raw:GT=hom:DP=26:FQ=1:SQ=-1;ID=S2500_raw:GT=hom:DP=12:FQ=1:SQ=-1;ID=S2502_raw:GT=hom:DP=23:FQ=1:SQ=-1;ID=S2505_raw:GT=hom:DP=17:FQ=1:SQ=-1;ID=S2176_raw:GT=hom:DP=40:FQ=1:SQ=-1;ID=S2506_raw:GT=hom:DP=21:FQ=1:SQ=-1;ID=S2667_raw:GT=hom:DP=6:FQ=1:SQ=-1;
5	1,12E+08	1,12E+08	C	A	intronic	MCC	.	.	rs4705538	.	ID=S2009_raw:GT=hom:DP=18:FQ=1:SQ=-1;ID=S2503_raw:GT=hom:DP=13:FQ=1:SQ=-1;ID=S2507_raw:GT=hom:DP=12:FQ=1:SQ=-1;ID=S2668_raw:GT=hom:DP=9:FQ=1:SQ=-1;ID=S2012_raw:GT=hom:DP=14:FQ=1:SQ=-1;ID=S2010_raw:GT=hom:DP=13:FQ=1:SQ=-1;ID=S2662_raw:GT=hom:DP=8:FQ=1:SQ=-1;ID=S2663_raw:GT=hom:DP=9:FQ=1:SQ=-1;ID=S2011_raw:GT=hom:DP=22:FQ=1:SQ=-1;ID=S2504_raw:GT=hom:DP=12:FQ=1:SQ=-1;ID=S2008_raw:GT=hom:DP=15:FQ=1:SQ=-1;ID=S2509_raw:GT=hom:DP=11:FQ=1:SQ=-1;ID=S2508_raw:GT=hom:DP=12:FQ=1:SQ=-1;ID=S2666_raw:GT=hom:DP=7:FQ=1:SQ=-1;ID=S2669_raw:GT=hom:DP=4:FQ=1:SQ=-1;ID=S2166_raw:GT=hom:DP=10:FQ=1:SQ=-1;ID=S1775_raw:GT=hom:DP=1:FQ=1:SQ=-1;ID=S2501_raw:GT=hom:DP=7:FQ=1:SQ=-1;ID=S2500_raw:GT=hom:DP=10:FQ=1:SQ=-1;ID=S2502_raw:GT=hom:DP=14:FQ=1:SQ=-1;ID=S2505_raw:GT=hom:DP=12:FQ=1:SQ=-1;ID=S2176_raw:GT=hom:DP=14:FQ=1:SQ=-1;ID=S2506_raw:GT=hom:DP=2:FQ=1:SQ=-1;ID=S2667_raw:GT=hom:DP=17:FQ=1:SQ=-1;
5	1,12E+08	1,12E+08	T	C	exonic	MCC	nonsynonymous SNV	MCC:NM_002387:exon5:c.569A>G:p.K190R,MCC:NM_001085377:exon7:c.1139A>G:p.K380R	rs6594681	.	ID=S2009_raw:GT=hom:DP=24:FQ=1:SQ=-1;ID=S2503_raw:GT=hom:DP=62:FQ=1:SQ=-1;ID=S2507_raw:GT=hom:DP=47:FQ=1:SQ=-1;ID=S2668_raw:GT=hom:DP=53:FQ=1:SQ=-1;ID=S2012_raw:GT=hom:DP=22:FQ=1:SQ=-1;ID=S2010_raw:GT=hom:DP=24:FQ=1:SQ=-1;ID=S2662_raw:GT=hom:DP=60:FQ=1:SQ=-1;ID=S2663_raw:GT=hom:DP=54:FQ=1:SQ=-1;ID=S2011_raw:GT=hom:DP=28:FQ=1:SQ=-1;ID=S2504_raw:GT=hom:DP=78:FQ=1:SQ=-1;ID=S2008_raw:GT=hom:DP=38:FQ=1:SQ=-1;ID=S2509_raw:GT=hom:DP=62:FQ=1:SQ=-1;ID=S2508_raw:GT=hom:DP=91:FQ=1:SQ=-1;ID=S2666_raw:GT=hom:DP=93:FQ=1:SQ=-1;ID=S2669_raw:GT=hom:DP=49:FQ=1:SQ=-1;ID=S2166_raw:GT=hom:DP=68:FQ=1:SQ=-1;ID=S1775_raw:GT=hom:DP=44:FQ=1:SQ=-1;ID=S2501_raw:GT=hom:DP=60:FQ=1:SQ=-1;ID=S2500_raw:GT=hom:DP=46:FQ=1:SQ=-1;ID=S2502_raw:GT=hom:DP=44:FQ=1:SQ=-1;ID=S2505_raw:GT=hom:DP=54:FQ=1:SQ=-1;ID=S2176_raw:GT=hom:DP=111:FQ=1:SQ=-1;ID=S2506_raw:GT=hom:DP=48:FQ=1:SQ=-1;ID=S2667_raw:GT=hom:DP=50:FQ=1:SQ=-1;
5	1,12E+08	1,12E+08	T	C	exonic	MCC	synonymous SNV	MCC:NM_002387:exon3:c.225A>G:p.T75T,MCC:NM_001085377:exon5:c.795A>G:p.T265T	rs2227949	.	ID=S2009_raw:GT=hom:DP=49:FQ=1:SQ=-1;ID=S2503_raw:GT=hom:DP=128:FQ=1:SQ=-1;ID=S2507_raw:GT=hom:DP=160:FQ=1:SQ=-1;ID=S2668_raw:GT=hom:DP=198:FQ=1:SQ=-1;ID=S2012_raw:GT=hom:DP=55:FQ=1:SQ=-1;ID=S2010_raw:GT=hom:DP=50:FQ=1:SQ=-1;ID=S2662_raw:GT=hom:DP=84:FQ=1:SQ=-1;ID=S2663_raw:GT=hom:DP=134:FQ=1:SQ=-1;ID=S2011_raw:GT=hom:DP=63:FQ=1:SQ=-1;ID=S2504_raw:GT=hom:DP=155:FQ=1:SQ=-1;ID=S2008_raw:GT=hom:DP=49:FQ=1:SQ=-1;ID=S2509_raw:GT=hom:DP=142:FQ=1:SQ=-1;ID=S2508_raw:GT=hom:DP=134:FQ=1:SQ=-1;ID=S2666_raw:GT=hom:DP=171:FQ=1:SQ=-1;ID=S2669_raw:GT=hom:DP=131:FQ=0.98:SQ=-1;ID=S2166_raw:GT=hom:DP=151:FQ=1:SQ=-1;ID=S1775_raw:GT=hom:DP=80:FQ=1:SQ=-1;ID=S2501_raw:GT=hom:DP=141:FQ=1:SQ=-1;ID=S2500_raw:GT=hom:DP=149:FQ=1:SQ=-1;ID=S2502_raw:GT=hom:DP=158:FQ=1:SQ=-1;ID=S2505_raw:GT=hom:DP=123:FQ=1:SQ=-1;ID=S2176_raw:GT=hom:DP=127:FQ=1:SQ=-1;ID=S2506_raw:GT=hom:DP=110:FQ=1:SQ=-1;ID=S2667_raw:GT=hom:DP=115:FQ=1:SQ=-1;
7	99693337	99693337	T	C	intronic	MCM7	.	.	rs7799115	.	ID=S2009_raw:GT=hom:DP=106:FQ=1:SQ=-1;ID=S2503_raw:GT=hom:DP=35:FQ=1:SQ=-1;ID=S2507_raw:GT=hom:DP=42:FQ=1:SQ=-1;ID=S2668_raw:GT=hom:DP=34:FQ=1:SQ=-1;ID=S2012_raw:GT=hom:DP=116:FQ=1:SQ=-1;ID=S2010_raw:GT=hom:DP=130:FQ=1:SQ=-1;ID=S2662_raw:GT=hom:DP=67:FQ=1:SQ=-1;ID=S2663_raw:GT=hom:DP=54:FQ=1:SQ=-1;ID=S2011_raw:GT=hom:DP=176:FQ=1:SQ=-1;ID=S2504_raw:GT=hom:DP=43:FQ=1:SQ=-1;ID=S2008_raw:GT=hom:DP=141:FQ=1:SQ=-1;ID=S2509_raw:GT=hom:DP=35:FQ=1:SQ=-1;ID=S2508_raw:GT=hom:DP=32:FQ=1:SQ=-1;ID=S2666_raw:GT=hom:DP=38:FQ=1:SQ=-1;ID=S2669_raw:GT=hom:DP=34:FQ=1:SQ=-1;ID=S2166_raw:GT=hom:DP=19:FQ=1:SQ=-1;ID=S1775_raw:GT=hom:DP=12:FQ=1:SQ=-1;ID=S2501_raw:GT=hom:DP=44:FQ=1:SQ=-1;ID=S2500_raw:GT=hom:DP=32:FQ=1:SQ=-1;ID=S2502_raw:GT=hom:DP=47:FQ=1:SQ=-1;ID=S2505_raw:GT=hom:DP=37:FQ=1:SQ=-1;ID=S2176_raw:GT=hom:DP=16:FQ=1:SQ=-1;ID=S2506_raw:GT=hom:DP=45:FQ=1:SQ=-1;ID=S2667_raw:GT=hom:DP=54:FQ=1:SQ=-1;
8	6272185	6272185	G	T	intronic	MCPH1	.	.	rs2440437	.	ID=S2009_raw:GT=hom:DP=2:FQ=1:SQ=-1;ID=S2503_raw:GT=hom:DP=9:FQ=1:SQ=-1;ID=S2507_raw:GT=hom:DP=7:FQ=1:SQ=-1;ID=S2668_raw:GT=hom:DP=8:FQ=1:SQ=-1;ID=S2012_raw:GT=hom:DP=5:FQ=1:SQ=-1;ID=S2010_raw:GT=hom:DP=6:FQ=1:SQ=-1;ID=S2662_raw:GT=hom:DP=2:FQ=1:SQ=-1;ID=S2663_raw:GT=hom:DP=8:FQ=1:SQ=-1;ID=S2011_raw:GT=hom:DP=7:FQ=1:SQ=-1;ID=S2504_raw:GT=hom:DP=7:FQ=1:SQ=-1;ID=S2008_raw:GT=hom:DP=7:FQ=1:SQ=-1;ID=S2509_raw:GT=hom:DP=2:FQ=1:SQ=-1;ID=S2508_raw:GT=hom:DP=10:FQ=1:SQ=-1;ID=S2666_raw:GT=hom:DP=4:FQ=1:SQ=-1;ID=S2669_raw:GT=hom:DP=6:FQ=1:SQ=-1;ID=S2166_raw:GT=hom:DP=19:FQ=1:SQ=-1;ID=S1775_raw:GT=hom:DP=11:FQ=1:SQ=-1;ID=S2501_raw:GT=hom:DP=8:FQ=1:SQ=-1;ID=S2500_raw:GT=hom:DP=8:FQ=1:SQ=-1;ID=S2502_raw:GT=hom:DP=10:FQ=1:SQ=-1;ID=S2505_raw:GT=hom:DP=10:FQ=1:SQ=-1;ID=S2176_raw:GT=hom:DP=22:FQ=1:SQ=-1;ID=S2506_raw:GT=hom:DP=8:FQ=1:SQ=-1;ID=S2667_raw:GT=hom:DP=6:FQ=1:SQ=-1;
8	6272477	6272477	A	G	intronic	MCPH1	.	.	rs2515415	.	ID=S2009_raw:GT=hom:DP=19:FQ=1:SQ=-1;ID=S2503_raw:GT=hom:DP=21:FQ=1:SQ=-1;ID=S2507_raw:GT=hom:DP=18:FQ=1:SQ=-1;ID=S2668_raw:GT=hom:DP=13:FQ=1:SQ=-1;ID=S2012_raw:GT=hom:DP=23:FQ=1:SQ=-1;ID=S2010_raw:GT=hom:DP=27:FQ=1:SQ=-1;ID=S2662_raw:GT=hom:DP=8:FQ=1:SQ=-1;ID=S2663_raw:GT=hom:DP=11:FQ=1:SQ=-1;ID=S2011_raw:GT=hom:DP=38:FQ=1:SQ=-1;ID=S2504_raw:GT=hom:DP=10:FQ=1:SQ=-1;ID=S2008_raw:GT=hom:DP=33:FQ=1:SQ=-1;ID=S2509_raw:GT=hom:DP=13:FQ=1:SQ=-1;ID=S2508_raw:GT=hom:DP=10:FQ=1:SQ=-1;ID=S2666_raw:GT=hom:DP=9:FQ=1:SQ=-1;ID=S2669_raw:GT=hom:DP=17:FQ=1:SQ=-1;ID=S2166_raw:GT=hom:DP=28:FQ=1:SQ=-1;ID=S1775_raw:GT=hom:DP=21:FQ=1:SQ=-1;ID=S2501_raw:GT=hom:DP=18:FQ=1:SQ=-1;ID=S2500_raw:GT=hom:DP=14:FQ=1:SQ=-1;ID=S2502_raw:GT=hom:DP=19:FQ=1:SQ=-1;ID=S2505_raw:GT=hom:DP=8:FQ=1:SQ=-1;ID=S2176_raw:GT=hom:DP=27:FQ=1:SQ=-1;ID=S2506_raw:GT=hom:DP=17:FQ=1:SQ=-1;ID=S2667_raw:GT=hom:DP=11:FQ=1:SQ=-1;
8	6272481	6272481	C	G	intronic	MCPH1	.	.	rs2442545	.	ID=S2009_raw:GT=hom:DP=18:FQ=1:SQ=-1;ID=S2503_raw:GT=hom:DP=19:FQ=1:SQ=-1;ID=S2507_raw:GT=hom:DP=18:FQ=1:SQ=-1;ID=S2668_raw:GT=hom:DP=14:FQ=1:SQ=-1;ID=S2012_raw:GT=hom:DP=22:FQ=1:SQ=-1;ID=S2010_raw:GT=hom:DP=27:FQ=1:SQ=-1;ID=S2662_raw:GT=hom:DP=8:FQ=1:SQ=-1;ID=S2663_raw:GT=hom:DP=11:FQ=1:SQ=-1;ID=S2011_raw:GT=hom:DP=35:FQ=1:SQ=-1;ID=S2504_raw:GT=hom:DP=9:FQ=1:SQ=-1;ID=S2008_raw:GT=hom:DP=29:FQ=1:SQ=-1;ID=S2509_raw:GT=hom:DP=13:FQ=1:SQ=-1;ID=S2508_raw:GT=hom:DP=9:FQ=1:SQ=-1;ID=S2666_raw:GT=hom:DP=9:FQ=1:SQ=-1;ID=S2669_raw:GT=hom:DP=16:FQ=1:SQ=-1;ID=S2166_raw:GT=hom:DP=25:FQ=1:SQ=-1;ID=S1775_raw:GT=hom:DP=18:FQ=1:SQ=-1;ID=S2501_raw:GT=hom:DP=17:FQ=1:SQ=-1;ID=S2500_raw:GT=hom:DP=9:FQ=1:SQ=-1;ID=S2502_raw:GT=hom:DP=19:FQ=1:SQ=-1;ID=S2505_raw:GT=hom:DP=7:FQ=1:SQ=-1;ID=S2176_raw:GT=hom:DP=23:FQ=1:SQ=-1;ID=S2506_raw:GT=hom:DP=16:FQ=1:SQ=-1;ID=S2667_raw:GT=hom:DP=10:FQ=1:SQ=-1;
8	6302418	6302418	A	G	exonic	MCPH1	nonsynonymous SNV	MCPH1:NM_001172575:exon7:c.1031A>G:p.D344G,MCPH1:NM_001172574:exon8:c.1175A>G:p.D392G,MCPH1:NM_001322042:exon8:c.1175A>G:p.D392G,MCPH1:NM_001322043:exon8:c.1169A>G:p.D390G,MCPH1:NM_001322045:exon8:c.1073A>G:p.D358G,MCPH1:NM_001363979:exon8:c.1175A>G:p.D392G,MCPH1:NM_001363980:exon8:c.1175A>G:p.D392G,MCPH1:NM_024596:exon8:c.1175A>G:p.D392G	rs2515569	Benign	ID=S2009_raw:GT=hom:DP=66:FQ=1:SQ=-1;ID=S2503_raw:GT=hom:DP=120:FQ=1:SQ=-1;ID=S2507_raw:GT=hom:DP=104:FQ=1:SQ=-1;ID=S2668_raw:GT=hom:DP=109:FQ=1:SQ=-1;ID=S2012_raw:GT=hom:DP=76:FQ=1:SQ=-1;ID=S2010_raw:GT=hom:DP=95:FQ=1:SQ=-1;ID=S2662_raw:GT=hom:DP=61:FQ=1:SQ=-1;ID=S2663_raw:GT=hom:DP=79:FQ=1:SQ=-1;ID=S2011_raw:GT=hom:DP=134:FQ=1:SQ=-1;ID=S2504_raw:GT=hom:DP=115:FQ=1:SQ=-1;ID=S2008_raw:GT=hom:DP=112:FQ=1:SQ=-1;ID=S2509_raw:GT=hom:DP=89:FQ=1:SQ=-1;ID=S2508_raw:GT=hom:DP=94:FQ=1:SQ=-1;ID=S2666_raw:GT=hom:DP=120:FQ=1:SQ=-1;ID=S2669_raw:GT=hom:DP=104:FQ=1:SQ=-1;ID=S2166_raw:GT=hom:DP=180:FQ=1:SQ=-1;ID=S1775_raw:GT=hom:DP=117:FQ=1:SQ=-1;ID=S2501_raw:GT=hom:DP=78:FQ=1:SQ=-1;ID=S2500_raw:GT=hom:DP=112:FQ=1:SQ=-1;ID=S2502_raw:GT=hom:DP=119:FQ=0.99:SQ=-1;ID=S2505_raw:GT=hom:DP=112:FQ=1:SQ=-1;ID=S2176_raw:GT=hom:DP=190:FQ=1:SQ=-1;ID=S2506_raw:GT=hom:DP=85:FQ=1:SQ=-1;ID=S2667_raw:GT=hom:DP=96:FQ=0.99:SQ=-1;
8	6312880	6312880	C	T	intronic	MCPH1	.	.	rs2515575	.	ID=S2009_raw:GT=hom:DP=8:FQ=1:SQ=-1;ID=S2503_raw:GT=hom:DP=21:FQ=1:SQ=-1;ID=S2507_raw:GT=hom:DP=24:FQ=1:SQ=-1;ID=S2668_raw:GT=hom:DP=19:FQ=1:SQ=-1;ID=S2012_raw:GT=hom:DP=1:FQ=1:SQ=-1;ID=S2010_raw:GT=hom:DP=4:FQ=1:SQ=-1;ID=S2662_raw:GT=hom:DP=3:FQ=1:SQ=-1;ID=S2663_raw:GT=hom:DP=15:FQ=1:SQ=-1;ID=S2011_raw:GT=hom:DP=5:FQ=1:SQ=-1;ID=S2504_raw:GT=hom:DP=20:FQ=1:SQ=-1;ID=S2008_raw:GT=hom:DP=3:FQ=1:SQ=-1;ID=S2509_raw:GT=hom:DP=19:FQ=1:SQ=-1;ID=S2508_raw:GT=hom:DP=25:FQ=1:SQ=-1;ID=S2666_raw:GT=hom:DP=4:FQ=1:SQ=-1;ID=S2669_raw:GT=hom:DP=7:FQ=1:SQ=-1;ID=S2166_raw:GT=hom:DP=23:FQ=1:SQ=-1;ID=S1775_raw:GT=hom:DP=15:FQ=1:SQ=-1;ID=S2501_raw:GT=hom:DP=19:FQ=1:SQ=-1;ID=S2500_raw:GT=hom:DP=24:FQ=1:SQ=-1;ID=S2502_raw:GT=hom:DP=21:FQ=1:SQ=-1;ID=S2505_raw:GT=hom:DP=15:FQ=1:SQ=-1;ID=S2176_raw:GT=hom:DP=36:FQ=1:SQ=-1;ID=S2506_raw:GT=hom:DP=14:FQ=1:SQ=-1;ID=S2667_raw:GT=hom:DP=14:FQ=1:SQ=-1;
7	75677430	75677430	C	G	UTR5	MDH2	.	NM_001282404:c.-9859C>G;NM_001282403:c.-49C>G;NM_005918:c.-49C>G	rs10231092	.	ID=S2009_raw:GT=hom:DP=29:FQ=1:SQ=-1;ID=S2503_raw:GT=hom:DP=47:FQ=1:SQ=-1;ID=S2507_raw:GT=hom:DP=67:FQ=1:SQ=-1;ID=S2668_raw:GT=hom:DP=101:FQ=1:SQ=-1;ID=S2012_raw:GT=hom:DP=43:FQ=1:SQ=-1;ID=S2010_raw:GT=hom:DP=36:FQ=1:SQ=-1;ID=S2662_raw:GT=hom:DP=177:FQ=1:SQ=-1;ID=S2663_raw:GT=hom:DP=92:FQ=1:SQ=-1;ID=S2011_raw:GT=hom:DP=43:FQ=1:SQ=-1;ID=S2504_raw:GT=hom:DP=53:FQ=1:SQ=-1;ID=S2008_raw:GT=hom:DP=51:FQ=1:SQ=-1;ID=S2509_raw:GT=hom:DP=78:FQ=1:SQ=-1;ID=S2508_raw:GT=hom:DP=65:FQ=1:SQ=-1;ID=S2666_raw:GT=hom:DP=33:FQ=1:SQ=-1;ID=S2669_raw:GT=hom:DP=83:FQ=1:SQ=-1;ID=S2166_raw:GT=hom:DP=66:FQ=1:SQ=-1;ID=S1775_raw:GT=hom:DP=78:FQ=1:SQ=-1;ID=S2501_raw:GT=hom:DP=65:FQ=1:SQ=-1;ID=S2500_raw:GT=hom:DP=35:FQ=1:SQ=-1;ID=S2502_raw:GT=hom:DP=83:FQ=1:SQ=-1;ID=S2505_raw:GT=hom:DP=63:FQ=1:SQ=-1;ID=S2176_raw:GT=hom:DP=47:FQ=1:SQ=-1;ID=S2506_raw:GT=hom:DP=43:FQ=1:SQ=-1;ID=S2667_raw:GT=hom:DP=65:FQ=1:SQ=-1;
7	75703166	75703166	T	G	intergenic	MDH2;GTF2IP7	.	dist=6339;dist=18215	rs4732557	.	ID=S2009_raw:GT=hom:DP=1:FQ=1:SQ=-1;ID=S2503_raw:GT=hom:DP=20:FQ=1:SQ=-1;ID=S2507_raw:GT=hom:DP=14:FQ=1:SQ=-1;ID=S2668_raw:GT=hom:DP=15:FQ=1:SQ=-1;ID=S2012_raw:GT=hom:DP=2:FQ=1:SQ=-1;ID=S2010_raw:GT=hom:DP=1:FQ=1:SQ=-1;ID=S2662_raw:GT=hom:DP=35:FQ=1:SQ=-1;ID=S2663_raw:GT=hom:DP=29:FQ=1:SQ=-1;ID=S2011_raw:GT=hom:DP=1:FQ=1:SQ=-1;ID=S2504_raw:GT=hom:DP=18:FQ=1:SQ=-1;ID=S2008_raw:GT=hom:DP=3:FQ=1:SQ=-1;ID=S2509_raw:GT=hom:DP=17:FQ=1:SQ=-1;ID=S2508_raw:GT=hom:DP=17:FQ=1:SQ=-1;ID=S2666_raw:GT=hom:DP=3:FQ=1:SQ=-1;ID=S2669_raw:GT=hom:DP=18:FQ=1:SQ=-1;ID=S2166_raw:GT=hom:DP=12:FQ=1:SQ=-1;ID=S1775_raw:GT=hom:DP=12:FQ=1:SQ=-1;ID=S2501_raw:GT=hom:DP=12:FQ=1:SQ=-1;ID=S2500_raw:GT=hom:DP=14:FQ=1:SQ=-1;ID=S2502_raw:GT=hom:DP=17:FQ=1:SQ=-1;ID=S2505_raw:GT=hom:DP=12:FQ=1:SQ=-1;ID=S2176_raw:GT=hom:DP=10:FQ=1:SQ=-1;ID=S2506_raw:GT=hom:DP=10:FQ=1:SQ=-1;ID=S2667_raw:GT=hom:DP=27:FQ=1:SQ=-1;
11	64572557	64572557	A	G	exonic	MEN1	synonymous SNV	MEN1:NM_000244:exon9:c.1314T>C:p.H438H,MEN1:NM_130799:exon9:c.1299T>C:p.H433H,MEN1:NM_130800:exon9:c.1314T>C:p.H438H,MEN1:NM_130801:exon9:c.1314T>C:p.H438H,MEN1:NM_130802:exon9:c.1314T>C:p.H438H,MEN1:NM_130803:exon9:c.1314T>C:p.H438H,MEN1:NM_130804:exon10:c.1314T>C:p.H438H	rs540012	Benign	ID=S2009_raw:GT=hom:DP=111:FQ=1:SQ=-1;ID=S2503_raw:GT=hom:DP=99:FQ=1:SQ=-1;ID=S2507_raw:GT=hom:DP=111:FQ=1:SQ=-1;ID=S2668_raw:GT=hom:DP=168:FQ=1:SQ=-1;ID=S2012_raw:GT=hom:DP=109:FQ=1:SQ=-1;ID=S2010_raw:GT=hom:DP=97:FQ=1:SQ=-1;ID=S2662_raw:GT=hom:DP=245:FQ=1:SQ=-1;ID=S2663_raw:GT=hom:DP=133:FQ=1:SQ=-1;ID=S2011_raw:GT=hom:DP=127:FQ=1:SQ=-1;ID=S2504_raw:GT=hom:DP=121:FQ=1:SQ=-1;ID=S2008_raw:GT=hom:DP=98:FQ=1:SQ=-1;ID=S2509_raw:GT=hom:DP=128:FQ=1:SQ=-1;ID=S2508_raw:GT=hom:DP=131:FQ=1:SQ=-1;ID=S2666_raw:GT=hom:DP=114:FQ=1:SQ=-1;ID=S2669_raw:GT=hom:DP=126:FQ=1:SQ=-1;ID=S2166_raw:GT=hom:DP=78:FQ=1:SQ=-1;ID=S1775_raw:GT=hom:DP=36:FQ=1:SQ=-1;ID=S2501_raw:GT=hom:DP=113:FQ=1:SQ=-1;ID=S2500_raw:GT=hom:DP=113:FQ=1:SQ=-1;ID=S2502_raw:GT=hom:DP=109:FQ=1:SQ=-1;ID=S2505_raw:GT=hom:DP=109:FQ=1:SQ=-1;ID=S2176_raw:GT=hom:DP=63:FQ=1:SQ=-1;ID=S2506_raw:GT=hom:DP=112:FQ=1:SQ=-1;ID=S2667_raw:GT=hom:DP=117:FQ=1:SQ=-1;
4	88813503	88813503	T	C	intergenic	MEPE;SPP1	.	dist=45535;dist=83299	rs6532031	.	ID=S2009_raw:GT=hom:DP=93:FQ=1:SQ=-1;ID=S2503_raw:GT=hom:DP=215:FQ=1:SQ=-1;ID=S2507_raw:GT=hom:DP=199:FQ=0.99:SQ=-1;ID=S2668_raw:GT=hom:DP=246:FQ=1:SQ=-1;ID=S2012_raw:GT=hom:DP=125:FQ=1:SQ=-1;ID=S2010_raw:GT=hom:DP=130:FQ=1:SQ=-1;ID=S2662_raw:GT=hom:DP=71:FQ=1:SQ=-1;ID=S2663_raw:GT=hom:DP=215:FQ=1:SQ=-1;ID=S2011_raw:GT=hom:DP=161:FQ=1:SQ=-1;ID=S2504_raw:GT=hom:DP=281:FQ=1:SQ=-1;ID=S2008_raw:GT=hom:DP=139:FQ=1:SQ=-1;ID=S2509_raw:GT=hom:DP=196:FQ=1:SQ=-1;ID=S2508_raw:GT=hom:DP=246:FQ=0.99:SQ=-1;ID=S2666_raw:GT=hom:DP=168:FQ=1:SQ=-1;ID=S2669_raw:GT=hom:DP=205:FQ=1:SQ=-1;ID=S2166_raw:GT=hom:DP=287:FQ=1:SQ=-1;ID=S1775_raw:GT=hom:DP=226:FQ=1:SQ=-1;ID=S2501_raw:GT=hom:DP=218:FQ=1:SQ=-1;ID=S2500_raw:GT=hom:DP=252:FQ=1:SQ=-1;ID=S2502_raw:GT=hom:DP=238:FQ=1:SQ=-1;ID=S2505_raw:GT=hom:DP=207:FQ=1:SQ=-1;ID=S2176_raw:GT=hom:DP=265:FQ=1:SQ=-1;ID=S2506_raw:GT=hom:DP=207:FQ=0.99:SQ=-1;ID=S2667_raw:GT=hom:DP=237:FQ=1:SQ=-1;
1	2,23E+08	2,23E+08	G	A	splicing	MIA3	.	NM_198551:exon3:c.268-4G>A;NM_001324063:exon3:c.268-4G>A;NM_001324064:exon4:UTR5;NM_001324062:exon3:c.268-4G>A	rs3008617	.	ID=S2009_raw:GT=hom:DP=26:FQ=1:SQ=-1;ID=S2503_raw:GT=hom:DP=108:FQ=1:SQ=-1;ID=S2507_raw:GT=hom:DP=88:FQ=1:SQ=-1;ID=S2668_raw:GT=hom:DP=118:FQ=1:SQ=-1;ID=S2012_raw:GT=hom:DP=29:FQ=1:SQ=-1;ID=S2010_raw:GT=hom:DP=31:FQ=1:SQ=-1;ID=S2662_raw:GT=hom:DP=2:FQ=1:SQ=-1;ID=S2663_raw:GT=hom:DP=83:FQ=1:SQ=-1;ID=S2011_raw:GT=hom:DP=41:FQ=1:SQ=-1;ID=S2504_raw:GT=hom:DP=111:FQ=1:SQ=-1;ID=S2008_raw:GT=hom:DP=29:FQ=1:SQ=-1;ID=S2509_raw:GT=hom:DP=88:FQ=1:SQ=-1;ID=S2508_raw:GT=hom:DP=86:FQ=1:SQ=-1;ID=S2666_raw:GT=hom:DP=107:FQ=1:SQ=-1;ID=S2669_raw:GT=hom:DP=64:FQ=1:SQ=-1;ID=S2166_raw:GT=hom:DP=106:FQ=1:SQ=-1;ID=S1775_raw:GT=hom:DP=38:FQ=1:SQ=-1;ID=S2501_raw:GT=hom:DP=98:FQ=1:SQ=-1;ID=S2500_raw:GT=hom:DP=80:FQ=1:SQ=-1;ID=S2502_raw:GT=hom:DP=116:FQ=1:SQ=-1;ID=S2505_raw:GT=hom:DP=110:FQ=1:SQ=-1;ID=S2176_raw:GT=hom:DP=75:FQ=1:SQ=-1;ID=S2506_raw:GT=hom:DP=67:FQ=1:SQ=-1;ID=S2667_raw:GT=hom:DP=104:FQ=1:SQ=-1;
1	2,23E+08	2,23E+08	A	G	exonic	MIA3	nonsynonymous SNV	MIA3:NM_001324062:exon4:c.1444A>G:p.K482E,MIA3:NM_001324063:exon4:c.1444A>G:p.K482E,MIA3:NM_198551:exon4:c.1444A>G:p.K482E,MIA3:NM_001324064:exon5:c.952A>G:p.K318E	rs2936053	.	ID=S2009_raw:GT=hom:DP=27:FQ=1:SQ=-1;ID=S2503_raw:GT=hom:DP=80:FQ=1:SQ=-1;ID=S2507_raw:GT=hom:DP=63:FQ=1:SQ=-1;ID=S2668_raw:GT=hom:DP=99:FQ=1:SQ=-1;ID=S2012_raw:GT=hom:DP=26:FQ=1:SQ=-1;ID=S2010_raw:GT=hom:DP=25:FQ=1:SQ=-1;ID=S2662_raw:GT=hom:DP=5:FQ=1:SQ=-1;ID=S2663_raw:GT=hom:DP=80:FQ=1:SQ=-1;ID=S2011_raw:GT=hom:DP=44:FQ=1:SQ=-1;ID=S2504_raw:GT=hom:DP=84:FQ=1:SQ=-1;ID=S2008_raw:GT=hom:DP=32:FQ=1:SQ=-1;ID=S2509_raw:GT=hom:DP=67:FQ=1:SQ=-1;ID=S2508_raw:GT=hom:DP=69:FQ=1:SQ=-1;ID=S2666_raw:GT=hom:DP=103:FQ=1:SQ=-1;ID=S2669_raw:GT=hom:DP=62:FQ=1:SQ=-1;ID=S2166_raw:GT=hom:DP=193:FQ=1:SQ=-1;ID=S1775_raw:GT=hom:DP=120:FQ=1:SQ=-1;ID=S2501_raw:GT=hom:DP=68:FQ=1:SQ=-1;ID=S2500_raw:GT=hom:DP=69:FQ=1:SQ=-1;ID=S2502_raw:GT=hom:DP=75:FQ=1:SQ=-1;ID=S2505_raw:GT=hom:DP=85:FQ=1:SQ=-1;ID=S2176_raw:GT=hom:DP=247:FQ=1:SQ=-1;ID=S2506_raw:GT=hom:DP=69:FQ=1:SQ=-1;ID=S2667_raw:GT=hom:DP=66:FQ=0.98:SQ=-1;
1	2,23E+08	2,23E+08	T	C	exonic	MIA3	synonymous SNV	MIA3:NM_001300867:exon19:c.1692T>C:p.P564P,MIA3:NM_001324065:exon19:c.1692T>C:p.P564P,MIA3:NM_001324063:exon22:c.4881T>C:p.P1627P,MIA3:NM_001324062:exon24:c.5058T>C:p.P1686P,MIA3:NM_198551:exon24:c.5058T>C:p.P1686P,MIA3:NM_001324064:exon25:c.4566T>C:p.P1522P	rs3002153	.	ID=S2009_raw:GT=hom:DP=83:FQ=1:SQ=-1;ID=S2503_raw:GT=hom:DP=71:FQ=1:SQ=-1;ID=S2507_raw:GT=hom:DP=93:FQ=1:SQ=-1;ID=S2668_raw:GT=hom:DP=128:FQ=1:SQ=-1;ID=S2012_raw:GT=hom:DP=114:FQ=1:SQ=-1;ID=S2010_raw:GT=hom:DP=124:FQ=1:SQ=-1;ID=S2662_raw:GT=hom:DP=17:FQ=1:SQ=-1;ID=S2663_raw:GT=hom:DP=79:FQ=1:SQ=-1;ID=S2011_raw:GT=hom:DP=140:FQ=1:SQ=-1;ID=S2504_raw:GT=hom:DP=99:FQ=1:SQ=-1;ID=S2008_raw:GT=hom:DP=94:FQ=1:SQ=-1;ID=S2509_raw:GT=hom:DP=81:FQ=1:SQ=-1;ID=S2508_raw:GT=hom:DP=101:FQ=1:SQ=-1;ID=S2666_raw:GT=hom:DP=114:FQ=1:SQ=-1;ID=S2669_raw:GT=hom:DP=70:FQ=1:SQ=-1;ID=S2166_raw:GT=hom:DP=89:FQ=1:SQ=-1;ID=S1775_raw:GT=hom:DP=56:FQ=1:SQ=-1;ID=S2501_raw:GT=hom:DP=90:FQ=1:SQ=-1;ID=S2500_raw:GT=hom:DP=83:FQ=1:SQ=-1;ID=S2502_raw:GT=hom:DP=103:FQ=1:SQ=-1;ID=S2505_raw:GT=hom:DP=84:FQ=1:SQ=-1;ID=S2176_raw:GT=hom:DP=84:FQ=1:SQ=-1;ID=S2506_raw:GT=hom:DP=80:FQ=1:SQ=-1;ID=S2667_raw:GT=hom:DP=93:FQ=1:SQ=-1;
14	75484057	75484057	T	C	intronic	MLH3	.	.	rs175050	.	ID=S2009_raw:GT=hom:DP=2:FQ=1:SQ=-1;ID=S2503_raw:GT=hom:DP=5:FQ=1:SQ=-1;ID=S2507_raw:GT=hom:DP=5:FQ=1:SQ=-1;ID=S2668_raw:GT=hom:DP=13:FQ=1:SQ=-1;ID=S2012_raw:GT=hom:DP=3:FQ=1:SQ=-1;ID=S2010_raw:GT=hom:DP=3:FQ=1:SQ=-1;ID=S2662_raw:GT=hom:DP=6:FQ=1:SQ=-1;ID=S2663_raw:GT=hom:DP=8:FQ=1:SQ=-1;ID=S2011_raw:GT=hom:DP=1:FQ=1:SQ=-1;ID=S2504_raw:GT=hom:DP=11:FQ=1:SQ=-1;ID=S2008_raw:GT=hom:DP=6:FQ=1:SQ=-1;ID=S2509_raw:GT=hom:DP=7:FQ=1:SQ=-1;ID=S2508_raw:GT=hom:DP=9:FQ=1:SQ=-1;ID=S2666_raw:GT=hom:DP=6:FQ=1:SQ=-1;ID=S2669_raw:GT=hom:DP=13:FQ=1:SQ=-1;ID=S2166_raw:GT=hom:DP=10:FQ=1:SQ=-1;ID=S1775_raw:GT=hom:DP=6:FQ=1:SQ=-1;ID=S2501_raw:GT=hom:DP=10:FQ=1:SQ=-1;ID=S2500_raw:GT=hom:DP=7:FQ=1:SQ=-1;ID=S2502_raw:GT=hom:DP=12:FQ=1:SQ=-1;ID=S2505_raw:GT=hom:DP=15:FQ=1:SQ=-1;ID=S2176_raw:GT=hom:DP=10:FQ=1:SQ=-1;ID=S2506_raw:GT=hom:DP=14:FQ=1:SQ=-1;ID=S2667_raw:GT=hom:DP=4:FQ=1:SQ=-1;
14	75485489	75485489	A	G	intronic	MLH3	.	.	rs175053	.	ID=S2009_raw:GT=hom:DP=305:FQ=1:SQ=-1;ID=S2503_raw:GT=hom:DP=55:FQ=1:SQ=-1;ID=S2507_raw:GT=hom:DP=52:FQ=1:SQ=-1;ID=S2668_raw:GT=hom:DP=77:FQ=1:SQ=-1;ID=S2012_raw:GT=hom:DP=384:FQ=1:SQ=-1;ID=S2010_raw:GT=hom:DP=368:FQ=1:SQ=-1;ID=S2662_raw:GT=hom:DP=8:FQ=1:SQ=-1;ID=S2663_raw:GT=hom:DP=44:FQ=1:SQ=-1;ID=S2011_raw:GT=hom:DP=502:FQ=1:SQ=-1;ID=S2504_raw:GT=hom:DP=65:FQ=1:SQ=-1;ID=S2008_raw:GT=hom:DP=352:FQ=1:SQ=-1;ID=S2509_raw:GT=hom:DP=66:FQ=1:SQ=-1;ID=S2508_raw:GT=hom:DP=48:FQ=1:SQ=-1;ID=S2666_raw:GT=hom:DP=77:FQ=1:SQ=-1;ID=S2669_raw:GT=hom:DP=47:FQ=1:SQ=-1;ID=S2166_raw:GT=hom:DP=82:FQ=1:SQ=-1;ID=S1775_raw:GT=hom:DP=49:FQ=1:SQ=-1;ID=S2501_raw:GT=hom:DP=72:FQ=1:SQ=-1;ID=S2500_raw:GT=hom:DP=49:FQ=1:SQ=-1;ID=S2502_raw:GT=hom:DP=62:FQ=1:SQ=-1;ID=S2505_raw:GT=hom:DP=62:FQ=1:SQ=-1;ID=S2176_raw:GT=hom:DP=89:FQ=1:SQ=-1;ID=S2506_raw:GT=hom:DP=67:FQ=1:SQ=-1;ID=S2667_raw:GT=hom:DP=32:FQ=1:SQ=-1;
14	75489446	75489446	T	C	intronic	MLH3	.	.	rs175056	.	ID=S2009_raw:GT=hom:DP=6:FQ=1:SQ=-1;ID=S2503_raw:GT=hom:DP=12:FQ=1:SQ=-1;ID=S2507_raw:GT=hom:DP=9:FQ=1:SQ=-1;ID=S2668_raw:GT=hom:DP=5:FQ=1:SQ=-1;ID=S2012_raw:GT=hom:DP=6:FQ=1:SQ=-1;ID=S2010_raw:GT=hom:DP=6:FQ=1:SQ=-1;ID=S2662_raw:GT=hom:DP=3:FQ=1:SQ=-1;ID=S2663_raw:GT=hom:DP=10:FQ=1:SQ=-1;ID=S2011_raw:GT=hom:DP=4:FQ=1:SQ=-1;ID=S2504_raw:GT=hom:DP=4:FQ=1:SQ=-1;ID=S2008_raw:GT=hom:DP=2:FQ=1:SQ=-1;ID=S2509_raw:GT=hom:DP=1:FQ=1:SQ=-1;ID=S2508_raw:GT=hom:DP=6:FQ=1:SQ=-1;ID=S2666_raw:GT=hom:DP=4:FQ=1:SQ=-1;ID=S2669_raw:GT=hom:DP=2:FQ=1:SQ=-1;ID=S2166_raw:GT=hom:DP=16:FQ=1:SQ=-1;ID=S1775_raw:GT=hom:DP=8:FQ=1:SQ=-1;ID=S2501_raw:GT=hom:DP=5:FQ=1:SQ=-1;ID=S2500_raw:GT=hom:DP=5:FQ=1:SQ=-1;ID=S2502_raw:GT=hom:DP=8:FQ=1:SQ=-1;ID=S2505_raw:GT=hom:DP=5:FQ=1:SQ=-1;ID=S2176_raw:GT=hom:DP=16:FQ=1:SQ=-1;ID=S2506_raw:GT=hom:DP=2:FQ=1:SQ=-1;ID=S2667_raw:GT=hom:DP=5:FQ=1:SQ=-1;
14	75497130	75497130	C	A	intronic	MLH3	.	.	rs175070	.	ID=S2009_raw:GT=hom:DP=37:FQ=1:SQ=-1;ID=S2503_raw:GT=hom:DP=24:FQ=1:SQ=-1;ID=S2507_raw:GT=hom:DP=20:FQ=1:SQ=-1;ID=S2668_raw:GT=hom:DP=18:FQ=1:SQ=-1;ID=S2012_raw:GT=hom:DP=47:FQ=1:SQ=-1;ID=S2010_raw:GT=hom:DP=41:FQ=1:SQ=-1;ID=S2662_raw:GT=hom:DP=2:FQ=1:SQ=-1;ID=S2663_raw:GT=hom:DP=8:FQ=1:SQ=-1;ID=S2011_raw:GT=hom:DP=65:FQ=1:SQ=-1;ID=S2504_raw:GT=hom:DP=30:FQ=1:SQ=-1;ID=S2008_raw:GT=hom:DP=50:FQ=1:SQ=-1;ID=S2509_raw:GT=hom:DP=12:FQ=1:SQ=-1;ID=S2508_raw:GT=hom:DP=16:FQ=1:SQ=-1;ID=S2666_raw:GT=hom:DP=13:FQ=1:SQ=-1;ID=S2669_raw:GT=hom:DP=13:FQ=1:SQ=-1;ID=S2166_raw:GT=hom:DP=55:FQ=1:SQ=-1;ID=S1775_raw:GT=hom:DP=34:FQ=1:SQ=-1;ID=S2501_raw:GT=hom:DP=16:FQ=1:SQ=-1;ID=S2500_raw:GT=hom:DP=19:FQ=1:SQ=-1;ID=S2502_raw:GT=hom:DP=22:FQ=1:SQ=-1;ID=S2505_raw:GT=hom:DP=17:FQ=1:SQ=-1;ID=S2176_raw:GT=hom:DP=82:FQ=1:SQ=-1;ID=S2506_raw:GT=hom:DP=18:FQ=1:SQ=-1;ID=S2667_raw:GT=hom:DP=11:FQ=1:SQ=-1;
14	75505016	75505016	A	G	intronic	MLH3	.	.	rs175075	.	ID=S2009_raw:GT=hom:DP=89:FQ=1:SQ=-1;ID=S2503_raw:GT=hom:DP=74:FQ=1:SQ=-1;ID=S2507_raw:GT=hom:DP=81:FQ=1:SQ=-1;ID=S2668_raw:GT=hom:DP=100:FQ=1:SQ=-1;ID=S2012_raw:GT=hom:DP=115:FQ=1:SQ=-1;ID=S2010_raw:GT=hom:DP=106:FQ=1:SQ=-1;ID=S2662_raw:GT=hom:DP=2:FQ=1:SQ=-1;ID=S2663_raw:GT=hom:DP=65:FQ=1:SQ=-1;ID=S2011_raw:GT=hom:DP=190:FQ=1:SQ=-1;ID=S2504_raw:GT=hom:DP=76:FQ=1:SQ=-1;ID=S2008_raw:GT=hom:DP=128:FQ=1:SQ=-1;ID=S2509_raw:GT=hom:DP=74:FQ=1:SQ=-1;ID=S2508_raw:GT=hom:DP=87:FQ=1:SQ=-1;ID=S2666_raw:GT=hom:DP=82:FQ=1:SQ=-1;ID=S2669_raw:GT=hom:DP=50:FQ=1:SQ=-1;ID=S2166_raw:GT=hom:DP=160:FQ=1:SQ=-1;ID=S1775_raw:GT=hom:DP=160:FQ=1:SQ=-1;ID=S2501_raw:GT=hom:DP=77:FQ=1:SQ=-1;ID=S2500_raw:GT=hom:DP=64:FQ=1:SQ=-1;ID=S2502_raw:GT=hom:DP=74:FQ=1:SQ=-1;ID=S2505_raw:GT=hom:DP=69:FQ=1:SQ=-1;ID=S2176_raw:GT=hom:DP=181:FQ=1:SQ=-1;ID=S2506_raw:GT=hom:DP=77:FQ=1:SQ=-1;ID=S2667_raw:GT=hom:DP=65:FQ=1:SQ=-1;
18	33778607	33778607	C	A	intronic	MOCOS	.	.	rs596644	.	ID=S2009_raw:GT=hom:DP=66:FQ=1:SQ=-1;ID=S2503_raw:GT=hom:DP=100:FQ=1:SQ=-1;ID=S2507_raw:GT=hom:DP=98:FQ=1:SQ=-1;ID=S2668_raw:GT=hom:DP=143:FQ=1:SQ=-1;ID=S2012_raw:GT=hom:DP=86:FQ=1:SQ=-1;ID=S2010_raw:GT=hom:DP=69:FQ=1:SQ=-1;ID=S2662_raw:GT=hom:DP=50:FQ=1:SQ=-1;ID=S2663_raw:GT=hom:DP=114:FQ=1:SQ=-1;ID=S2011_raw:GT=hom:DP=102:FQ=1:SQ=-1;ID=S2504_raw:GT=hom:DP=100:FQ=1:SQ=-1;ID=S2008_raw:GT=hom:DP=78:FQ=1:SQ=-1;ID=S2509_raw:GT=hom:DP=107:FQ=1:SQ=-1;ID=S2508_raw:GT=hom:DP=134:FQ=1:SQ=-1;ID=S2666_raw:GT=hom:DP=183:FQ=1:SQ=-1;ID=S2669_raw:GT=hom:DP=93:FQ=1:SQ=-1;ID=S2166_raw:GT=hom:DP=226:FQ=1:SQ=-1;ID=S1775_raw:GT=hom:DP=201:FQ=1:SQ=-1;ID=S2501_raw:GT=hom:DP=79:FQ=1:SQ=-1;ID=S2500_raw:GT=hom:DP=91:FQ=1:SQ=-1;ID=S2502_raw:GT=hom:DP=111:FQ=1:SQ=-1;ID=S2505_raw:GT=hom:DP=106:FQ=1:SQ=-1;ID=S2176_raw:GT=hom:DP=257:FQ=1:SQ=-1;ID=S2506_raw:GT=hom:DP=78:FQ=1:SQ=-1;ID=S2667_raw:GT=hom:DP=108:FQ=1:SQ=-1;
18	33779821	33779821	C	A	exonic	MOCOS	synonymous SNV	MOCOS:NM_017947:exon4:c.475C>A:p.R159R	rs646424	.	ID=S2009_raw:GT=hom:DP=92:FQ=1:SQ=-1;ID=S2503_raw:GT=hom:DP=236:FQ=1:SQ=-1;ID=S2507_raw:GT=hom:DP=210:FQ=1:SQ=-1;ID=S2668_raw:GT=hom:DP=292:FQ=1:SQ=-1;ID=S2012_raw:GT=hom:DP=92:FQ=1:SQ=-1;ID=S2010_raw:GT=hom:DP=114:FQ=1:SQ=-1;ID=S2662_raw:GT=hom:DP=217:FQ=1:SQ=-1;ID=S2663_raw:GT=hom:DP=193:FQ=1:SQ=-1;ID=S2011_raw:GT=hom:DP=140:FQ=1:SQ=-1;ID=S2504_raw:GT=hom:DP=312:FQ=1:SQ=-1;ID=S2008_raw:GT=hom:DP=100:FQ=1:SQ=-1;ID=S2509_raw:GT=hom:DP=193:FQ=1:SQ=-1;ID=S2508_raw:GT=hom:DP=270:FQ=1:SQ=-1;ID=S2666_raw:GT=hom:DP=215:FQ=1:SQ=-1;ID=S2669_raw:GT=hom:DP=215:FQ=1:SQ=-1;ID=S2166_raw:GT=hom:DP=175:FQ=1:SQ=-1;ID=S1775_raw:GT=hom:DP=102:FQ=1:SQ=-1;ID=S2501_raw:GT=hom:DP=193:FQ=1:SQ=-1;ID=S2500_raw:GT=hom:DP=224:FQ=1:SQ=-1;ID=S2502_raw:GT=hom:DP=269:FQ=1:SQ=-1;ID=S2505_raw:GT=hom:DP=200:FQ=1:SQ=-1;ID=S2176_raw:GT=hom:DP=165:FQ=1:SQ=-1;ID=S2506_raw:GT=hom:DP=222:FQ=1:SQ=-1;ID=S2667_raw:GT=hom:DP=235:FQ=1:SQ=-1;
18	33779896	33779896	A	G	exonic	MOCOS	nonsynonymous SNV	MOCOS:NM_017947:exon4:c.550A>G:p.S184G	rs540967	.	ID=S2009_raw:GT=hom:DP=133:FQ=1:SQ=-1;ID=S2503_raw:GT=hom:DP=345:FQ=1:SQ=-1;ID=S2507_raw:GT=hom:DP=297:FQ=1:SQ=-1;ID=S2668_raw:GT=hom:DP=352:FQ=1:SQ=-1;ID=S2012_raw:GT=hom:DP=147:FQ=1:SQ=-1;ID=S2010_raw:GT=hom:DP=158:FQ=1:SQ=-1;ID=S2662_raw:GT=hom:DP=273:FQ=1:SQ=-1;ID=S2663_raw:GT=hom:DP=242:FQ=1:SQ=-1;ID=S2011_raw:GT=hom:DP=193:FQ=1:SQ=-1;ID=S2504_raw:GT=hom:DP=387:FQ=1:SQ=-1;ID=S2008_raw:GT=hom:DP=130:FQ=1:SQ=-1;ID=S2509_raw:GT=hom:DP=294:FQ=1:SQ=-1;ID=S2508_raw:GT=hom:DP=368:FQ=1:SQ=-1;ID=S2666_raw:GT=hom:DP=299:FQ=1:SQ=-1;ID=S2669_raw:GT=hom:DP=283:FQ=1:SQ=-1;ID=S2166_raw:GT=hom:DP=309:FQ=1:SQ=-1;ID=S1775_raw:GT=hom:DP=183:FQ=1:SQ=-1;ID=S2501_raw:GT=hom:DP=300:FQ=1:SQ=-1;ID=S2500_raw:GT=hom:DP=295:FQ=1:SQ=-1;ID=S2502_raw:GT=hom:DP=413:FQ=1:SQ=-1;ID=S2505_raw:GT=hom:DP=300:FQ=1:SQ=-1;ID=S2176_raw:GT=hom:DP=327:FQ=1:SQ=-1;ID=S2506_raw:GT=hom:DP=289:FQ=1:SQ=-1;ID=S2667_raw:GT=hom:DP=307:FQ=1:SQ=-1;
18	33780020	33780020	A	G	exonic	MOCOS	nonsynonymous SNV	MOCOS:NM_017947:exon4:c.674A>G:p.H225R	rs623558	.	ID=S2009_raw:GT=hom:DP=167:FQ=1:SQ=-1;ID=S2503_raw:GT=hom:DP=362:FQ=1:SQ=-1;ID=S2507_raw:GT=hom:DP=411:FQ=1:SQ=-1;ID=S2668_raw:GT=hom:DP=411:FQ=1:SQ=-1;ID=S2012_raw:GT=hom:DP=246:FQ=1:SQ=-1;ID=S2010_raw:GT=hom:DP=258:FQ=1:SQ=-1;ID=S2662_raw:GT=hom:DP=382:FQ=1:SQ=-1;ID=S2663_raw:GT=hom:DP=382:FQ=1:SQ=-1;ID=S2011_raw:GT=hom:DP=338:FQ=1:SQ=-1;ID=S2504_raw:GT=hom:DP=457:FQ=1:SQ=-1;ID=S2008_raw:GT=hom:DP=282:FQ=1:SQ=-1;ID=S2509_raw:GT=hom:DP=400:FQ=1:SQ=-1;ID=S2508_raw:GT=hom:DP=439:FQ=1:SQ=-1;ID=S2666_raw:GT=hom:DP=416:FQ=1:SQ=-1;ID=S2669_raw:GT=hom:DP=405:FQ=1:SQ=-1;ID=S2166_raw:GT=hom:DP=406:FQ=1:SQ=-1;ID=S1775_raw:GT=hom:DP=328:FQ=1:SQ=-1;ID=S2501_raw:GT=hom:DP=361:FQ=1:SQ=-1;ID=S2500_raw:GT=hom:DP=358:FQ=1:SQ=-1;ID=S2502_raw:GT=hom:DP=476:FQ=1:SQ=-1;ID=S2505_raw:GT=hom:DP=436:FQ=1:SQ=-1;ID=S2176_raw:GT=hom:DP=485:FQ=1:SQ=-1;ID=S2506_raw:GT=hom:DP=354:FQ=1:SQ=-1;ID=S2667_raw:GT=hom:DP=387:FQ=1:SQ=-1;
18	33785185	33785185	A	G	exonic	MOCOS	synonymous SNV	MOCOS:NM_017947:exon6:c.1164A>G:p.P388P	rs667667	.	ID=S2009_raw:GT=hom:DP=50:FQ=1:SQ=-1;ID=S2503_raw:GT=hom:DP=86:FQ=1:SQ=-1;ID=S2507_raw:GT=hom:DP=89:FQ=1:SQ=-1;ID=S2668_raw:GT=hom:DP=104:FQ=1:SQ=-1;ID=S2012_raw:GT=hom:DP=62:FQ=1:SQ=-1;ID=S2010_raw:GT=hom:DP=80:FQ=1:SQ=-1;ID=S2662_raw:GT=hom:DP=33:FQ=1:SQ=-1;ID=S2663_raw:GT=hom:DP=102:FQ=1:SQ=-1;ID=S2011_raw:GT=hom:DP=95:FQ=1:SQ=-1;ID=S2504_raw:GT=hom:DP=114:FQ=1:SQ=-1;ID=S2008_raw:GT=hom:DP=68:FQ=1:SQ=-1;ID=S2509_raw:GT=hom:DP=84:FQ=1:SQ=-1;ID=S2508_raw:GT=hom:DP=92:FQ=1:SQ=-1;ID=S2666_raw:GT=hom:DP=127:FQ=1:SQ=-1;ID=S2669_raw:GT=hom:DP=102:FQ=1:SQ=-1;ID=S2166_raw:GT=hom:DP=40:FQ=1:SQ=-1;ID=S1775_raw:GT=hom:DP=27:FQ=1:SQ=-1;ID=S2501_raw:GT=hom:DP=89:FQ=1:SQ=-1;ID=S2500_raw:GT=hom:DP=82:FQ=1:SQ=-1;ID=S2502_raw:GT=hom:DP=105:FQ=1:SQ=-1;ID=S2505_raw:GT=hom:DP=90:FQ=1:SQ=-1;ID=S2176_raw:GT=hom:DP=56:FQ=1:SQ=-1;ID=S2506_raw:GT=hom:DP=66:FQ=1:SQ=-1;ID=S2667_raw:GT=hom:DP=88:FQ=1:SQ=-1;
18	33785304	33785304	T	C	intronic	MOCOS	.	.	rs667195	.	ID=S2009_raw:GT=hom:DP=44:FQ=1:SQ=-1;ID=S2503_raw:GT=hom:DP=75:FQ=1:SQ=-1;ID=S2507_raw:GT=hom:DP=41:FQ=1:SQ=-1;ID=S2668_raw:GT=hom:DP=79:FQ=1:SQ=-1;ID=S2012_raw:GT=hom:DP=63:FQ=1:SQ=-1;ID=S2010_raw:GT=hom:DP=78:FQ=1:SQ=-1;ID=S2662_raw:GT=hom:DP=25:FQ=1:SQ=-1;ID=S2663_raw:GT=hom:DP=67:FQ=1:SQ=-1;ID=S2011_raw:GT=hom:DP=74:FQ=1:SQ=-1;ID=S2504_raw:GT=hom:DP=44:FQ=1:SQ=-1;ID=S2008_raw:GT=hom:DP=58:FQ=1:SQ=-1;ID=S2509_raw:GT=hom:DP=34:FQ=1:SQ=-1;ID=S2508_raw:GT=hom:DP=61:FQ=1:SQ=-1;ID=S2666_raw:GT=hom:DP=94:FQ=1:SQ=-1;ID=S2669_raw:GT=hom:DP=65:FQ=1:SQ=-1;ID=S2166_raw:GT=hom:DP=9:FQ=1:SQ=-1;ID=S1775_raw:GT=hom:DP=2:FQ=1:SQ=-1;ID=S2501_raw:GT=hom:DP=47:FQ=1:SQ=-1;ID=S2500_raw:GT=hom:DP=43:FQ=1:SQ=-1;ID=S2502_raw:GT=hom:DP=49:FQ=1:SQ=-1;ID=S2505_raw:GT=hom:DP=49:FQ=1:SQ=-1;ID=S2176_raw:GT=hom:DP=14:FQ=1:SQ=-1;ID=S2506_raw:GT=hom:DP=33:FQ=1:SQ=-1;ID=S2667_raw:GT=hom:DP=65:FQ=1:SQ=-1;
3	1,31E+08	1,31E+08	A	C	intergenic	MRPL3;CPNE4	.	dist=23681;dist=6908	rs9839353	.	ID=S2009_raw:GT=hom:DP=20:FQ=1:SQ=-1;ID=S2503_raw:GT=hom:DP=10:FQ=1:SQ=-1;ID=S2507_raw:GT=hom:DP=11:FQ=1:SQ=-1;ID=S2668_raw:GT=hom:DP=4:FQ=1:SQ=-1;ID=S2012_raw:GT=hom:DP=24:FQ=1:SQ=-1;ID=S2010_raw:GT=hom:DP=32:FQ=1:SQ=-1;ID=S2662_raw:GT=hom:DP=7:FQ=1:SQ=-1;ID=S2663_raw:GT=hom:DP=3:FQ=1:SQ=-1;ID=S2011_raw:GT=hom:DP=26:FQ=1:SQ=-1;ID=S2504_raw:GT=hom:DP=7:FQ=1:SQ=-1;ID=S2008_raw:GT=hom:DP=19:FQ=1:SQ=-1;ID=S2509_raw:GT=hom:DP=3:FQ=1:SQ=-1;ID=S2508_raw:GT=hom:DP=12:FQ=1:SQ=-1;ID=S2666_raw:GT=hom:DP=1:FQ=1:SQ=-1;ID=S2669_raw:GT=hom:DP=4:FQ=1:SQ=-1;ID=S2166_raw:GT=hom:DP=5:FQ=1:SQ=-1;ID=S1775_raw:GT=hom:DP=4:FQ=1:SQ=-1;ID=S2501_raw:GT=hom:DP=5:FQ=1:SQ=-1;ID=S2500_raw:GT=hom:DP=6:FQ=1:SQ=-1;ID=S2502_raw:GT=hom:DP=6:FQ=1:SQ=-1;ID=S2505_raw:GT=hom:DP=9:FQ=1:SQ=-1;ID=S2176_raw:GT=hom:DP=4:FQ=1:SQ=-1;ID=S2506_raw:GT=hom:DP=8:FQ=1:SQ=-1;ID=S2667_raw:GT=hom:DP=12:FQ=1:SQ=-1;
5	79974646	79974646	T	C	intronic	MSH3	.	.	rs836807	.	ID=S2009_raw:GT=hom:DP=21:FQ=1:SQ=-1;ID=S2503_raw:GT=hom:DP=35:FQ=1:SQ=-1;ID=S2507_raw:GT=hom:DP=37:FQ=1:SQ=-1;ID=S2668_raw:GT=hom:DP=35:FQ=1:SQ=-1;ID=S2012_raw:GT=hom:DP=35:FQ=1:SQ=-1;ID=S2010_raw:GT=hom:DP=36:FQ=1:SQ=-1;ID=S2662_raw:GT=hom:DP=2:FQ=1:SQ=-1;ID=S2663_raw:GT=hom:DP=32:FQ=1:SQ=-1;ID=S2011_raw:GT=hom:DP=51:FQ=1:SQ=-1;ID=S2504_raw:GT=hom:DP=59:FQ=1:SQ=-1;ID=S2008_raw:GT=hom:DP=25:FQ=1:SQ=-1;ID=S2509_raw:GT=hom:DP=23:FQ=1:SQ=-1;ID=S2508_raw:GT=hom:DP=43:FQ=1:SQ=-1;ID=S2666_raw:GT=hom:DP=36:FQ=1:SQ=-1;ID=S2669_raw:GT=hom:DP=26:FQ=1:SQ=-1;ID=S2166_raw:GT=hom:DP=13:FQ=1:SQ=-1;ID=S1775_raw:GT=hom:DP=6:FQ=1:SQ=-1;ID=S2501_raw:GT=hom:DP=56:FQ=1:SQ=-1;ID=S2500_raw:GT=hom:DP=35:FQ=1:SQ=-1;ID=S2502_raw:GT=hom:DP=56:FQ=1:SQ=-1;ID=S2505_raw:GT=hom:DP=44:FQ=1:SQ=-1;ID=S2176_raw:GT=hom:DP=26:FQ=1:SQ=-1;ID=S2506_raw:GT=hom:DP=35:FQ=1:SQ=-1;ID=S2667_raw:GT=hom:DP=28:FQ=1:SQ=-1;
1	1,56E+08	1,56E+08	T	-	intergenic	MSTO1;YY1AP1	.	dist=33661;dist=10814	.	.	ID=S2009_raw:GT=hom:DP=170:FQ=1:SQ=-1;ID=S2503_raw:GT=hom:DP=125:FQ=1:SQ=-1;ID=S2507_raw:GT=hom:DP=135:FQ=1:SQ=-1;ID=S2668_raw:GT=hom:DP=132:FQ=1:SQ=-1;ID=S2012_raw:GT=hom:DP=190:FQ=1:SQ=-1;ID=S2010_raw:GT=hom:DP=173:FQ=1:SQ=-1;ID=S2662_raw:GT=hom:DP=55:FQ=1:SQ=-1;ID=S2663_raw:GT=hom:DP=111:FQ=1:SQ=-1;ID=S2011_raw:GT=hom:DP=278:FQ=1:SQ=-1;ID=S2504_raw:GT=hom:DP=134:FQ=1:SQ=-1;ID=S2008_raw:GT=hom:DP=224:FQ=1:SQ=-1;ID=S2509_raw:GT=hom:DP=135:FQ=1:SQ=-1;ID=S2508_raw:GT=hom:DP=149:FQ=1:SQ=-1;ID=S2666_raw:GT=hom:DP=77:FQ=1:SQ=-1;ID=S2669_raw:GT=hom:DP=154:FQ=1:SQ=-1;ID=S2166_raw:GT=hom:DP=175:FQ=1:SQ=-1;ID=S1775_raw:GT=hom:DP=86:FQ=1:SQ=-1;ID=S2501_raw:GT=hom:DP=121:FQ=1:SQ=-1;ID=S2500_raw:GT=hom:DP=133:FQ=1:SQ=-1;ID=S2502_raw:GT=hom:DP=135:FQ=1:SQ=-1;ID=S2505_raw:GT=hom:DP=124:FQ=1:SQ=-1;ID=S2176_raw:GT=hom:DP=209:FQ=1:SQ=-1;ID=S2506_raw:GT=hom:DP=143:FQ=1:SQ=-1;ID=S2667_raw:GT=hom:DP=144:FQ=1:SQ=-1;
22	36722534	36722534	G	A	intronic	MYH9	.	.	rs8136336	.	ID=S2009_raw:GT=hom:DP=21:FQ=1:SQ=-1;ID=S2503_raw:GT=hom:DP=94:FQ=1:SQ=-1;ID=S2507_raw:GT=hom:DP=82:FQ=1:SQ=-1;ID=S2668_raw:GT=hom:DP=67:FQ=1:SQ=-1;ID=S2012_raw:GT=hom:DP=23:FQ=1:SQ=-1;ID=S2010_raw:GT=hom:DP=27:FQ=1:SQ=-1;ID=S2662_raw:GT=hom:DP=94:FQ=1:SQ=-1;ID=S2663_raw:GT=hom:DP=51:FQ=1:SQ=-1;ID=S2011_raw:GT=hom:DP=34:FQ=0.97:SQ=-1;ID=S2504_raw:GT=hom:DP=87:FQ=1:SQ=-1;ID=S2008_raw:GT=hom:DP=28:FQ=1:SQ=-1;ID=S2509_raw:GT=hom:DP=63:FQ=1:SQ=-1;ID=S2508_raw:GT=hom:DP=55:FQ=1:SQ=-1;ID=S2666_raw:GT=hom:DP=114:FQ=1:SQ=-1;ID=S2669_raw:GT=hom:DP=73:FQ=1:SQ=-1;ID=S2166_raw:GT=hom:DP=87:FQ=1:SQ=-1;ID=S1775_raw:GT=hom:DP=53:FQ=1:SQ=-1;ID=S2501_raw:GT=hom:DP=81:FQ=1:SQ=-1;ID=S2500_raw:GT=hom:DP=65:FQ=1:SQ=-1;ID=S2502_raw:GT=hom:DP=93:FQ=1:SQ=-1;ID=S2505_raw:GT=hom:DP=102:FQ=1:SQ=-1;ID=S2176_raw:GT=hom:DP=71:FQ=1:SQ=-1;ID=S2506_raw:GT=hom:DP=88:FQ=1:SQ=-1;ID=S2667_raw:GT=hom:DP=78:FQ=1:SQ=-1;
3	1,23E+08	1,23E+08	GATG	-	intronic	MYLK	.	.	.	.	ID=S2009_raw:GT=hom:DP=2:FQ=1:SQ=-1;ID=S2503_raw:GT=hom:DP=4:FQ=1:SQ=-1;ID=S2507_raw:GT=hom:DP=3:FQ=1:SQ=-1;ID=S2668_raw:GT=hom:DP=8:FQ=1:SQ=-1;ID=S2012_raw:GT=hom:DP=3:FQ=1:SQ=-1;ID=S2010_raw:GT=hom:DP=2:FQ=1:SQ=-1;ID=S2662_raw:GT=hom:DP=4:FQ=1:SQ=-1;ID=S2663_raw:GT=hom:DP=4:FQ=1:SQ=-1;ID=S2011_raw:GT=hom:DP=4:FQ=1:SQ=-1;ID=S2504_raw:GT=hom:DP=3:FQ=1:SQ=-1;ID=S2008_raw:GT=hom:DP=3:FQ=1:SQ=-1;ID=S2509_raw:GT=hom:DP=4:FQ=1:SQ=-1;ID=S2508_raw:GT=hom:DP=4:FQ=1:SQ=-1;ID=S2666_raw:GT=hom:DP=3:FQ=1:SQ=-1;ID=S2669_raw:GT=hom:DP=4:FQ=1:SQ=-1;ID=S2166_raw:GT=hom:DP=1:FQ=1:SQ=-1;ID=S1775_raw:GT=hom:DP=3:FQ=1:SQ=-1;ID=S2501_raw:GT=hom:DP=2:FQ=1:SQ=-1;ID=S2500_raw:GT=hom:DP=1:FQ=1:SQ=-1;ID=S2502_raw:GT=hom:DP=3:FQ=1:SQ=-1;ID=S2505_raw:GT=hom:DP=8:FQ=1:SQ=-1;ID=S2176_raw:GT=hom:DP=2:FQ=1:SQ=-1;ID=S2506_raw:GT=hom:DP=7:FQ=1:SQ=-1;ID=S2667_raw:GT=hom:DP=2:FQ=1:SQ=-1;
3	1,23E+08	1,23E+08	G	A	splicing	MYLK	.	NM_001321309:exon12:c.1276+8C>T;NM_053028:exon12:c.1597+8C>T;NM_053027:exon13:c.1804+8C>T;NM_053026:exon12:c.1597+8C>T;NM_053025:exon13:c.1804+8C>T	rs820355	Benign/Likely_benign	ID=S2009_raw:GT=hom:DP=45:FQ=1:SQ=-1;ID=S2503_raw:GT=hom:DP=122:FQ=1:SQ=-1;ID=S2507_raw:GT=hom:DP=126:FQ=1:SQ=-1;ID=S2668_raw:GT=hom:DP=151:FQ=1:SQ=-1;ID=S2012_raw:GT=hom:DP=55:FQ=1:SQ=-1;ID=S2010_raw:GT=hom:DP=69:FQ=1:SQ=-1;ID=S2662_raw:GT=hom:DP=155:FQ=1:SQ=-1;ID=S2663_raw:GT=hom:DP=173:FQ=0.99:SQ=-1;ID=S2011_raw:GT=hom:DP=76:FQ=1:SQ=-1;ID=S2504_raw:GT=hom:DP=174:FQ=1:SQ=-1;ID=S2008_raw:GT=hom:DP=69:FQ=1:SQ=-1;ID=S2509_raw:GT=hom:DP=167:FQ=1:SQ=-1;ID=S2508_raw:GT=hom:DP=182:FQ=1:SQ=-1;ID=S2666_raw:GT=hom:DP=151:FQ=1:SQ=-1;ID=S2669_raw:GT=hom:DP=185:FQ=1:SQ=-1;ID=S2166_raw:GT=hom:DP=218:FQ=1:SQ=-1;ID=S1775_raw:GT=hom:DP=172:FQ=1:SQ=-1;ID=S2501_raw:GT=hom:DP=146:FQ=1:SQ=-1;ID=S2500_raw:GT=hom:DP=159:FQ=1:SQ=-1;ID=S2502_raw:GT=hom:DP=156:FQ=1:SQ=-1;ID=S2505_raw:GT=hom:DP=149:FQ=0.99:SQ=-1;ID=S2176_raw:GT=hom:DP=204:FQ=1:SQ=-1;ID=S2506_raw:GT=hom:DP=123:FQ=1:SQ=-1;ID=S2667_raw:GT=hom:DP=162:FQ=1:SQ=-1;
3	1,23E+08	1,23E+08	T	C	intronic	MYLK	.	.	rs820330	.	ID=S2009_raw:GT=hom:DP=4:FQ=1:SQ=-1;ID=S2503_raw:GT=hom:DP=17:FQ=1:SQ=-1;ID=S2507_raw:GT=hom:DP=18:FQ=1:SQ=-1;ID=S2668_raw:GT=hom:DP=15:FQ=1:SQ=-1;ID=S2012_raw:GT=hom:DP=2:FQ=1:SQ=-1;ID=S2010_raw:GT=hom:DP=4:FQ=1:SQ=-1;ID=S2662_raw:GT=hom:DP=16:FQ=1:SQ=-1;ID=S2663_raw:GT=hom:DP=21:FQ=1:SQ=-1;ID=S2011_raw:GT=hom:DP=9:FQ=1:SQ=-1;ID=S2504_raw:GT=hom:DP=14:FQ=1:SQ=-1;ID=S2008_raw:GT=hom:DP=8:FQ=1:SQ=-1;ID=S2509_raw:GT=hom:DP=11:FQ=1:SQ=-1;ID=S2508_raw:GT=hom:DP=12:FQ=1:SQ=-1;ID=S2666_raw:GT=hom:DP=12:FQ=1:SQ=-1;ID=S2669_raw:GT=hom:DP=10:FQ=1:SQ=-1;ID=S2166_raw:GT=hom:DP=15:FQ=1:SQ=-1;ID=S1775_raw:GT=hom:DP=19:FQ=1:SQ=-1;ID=S2501_raw:GT=hom:DP=13:FQ=1:SQ=-1;ID=S2500_raw:GT=hom:DP=14:FQ=1:SQ=-1;ID=S2502_raw:GT=hom:DP=29:FQ=1:SQ=-1;ID=S2505_raw:GT=hom:DP=21:FQ=1:SQ=-1;ID=S2176_raw:GT=hom:DP=35:FQ=0.97:SQ=-1;ID=S2506_raw:GT=hom:DP=10:FQ=1:SQ=-1;ID=S2667_raw:GT=hom:DP=15:FQ=1:SQ=-1;
3	1,23E+08	1,23E+08	G	C	exonic	MYLK	nonsynonymous SNV	MYLK:NM_001321309:exon10:c.958C>G:p.L320V,MYLK:NM_053025:exon11:c.1486C>G:p.L496V,MYLK:NM_053027:exon11:c.1486C>G:p.L496V	rs9833275	Benign/Likely_benign	ID=S2009_raw:GT=hom:DP=47:FQ=1:SQ=-1;ID=S2503_raw:GT=hom:DP=51:FQ=1:SQ=-1;ID=S2507_raw:GT=hom:DP=87:FQ=1:SQ=-1;ID=S2668_raw:GT=hom:DP=109:FQ=1:SQ=-1;ID=S2012_raw:GT=hom:DP=49:FQ=1:SQ=-1;ID=S2010_raw:GT=hom:DP=48:FQ=1:SQ=-1;ID=S2662_raw:GT=hom:DP=61:FQ=1:SQ=-1;ID=S2663_raw:GT=hom:DP=64:FQ=1:SQ=-1;ID=S2011_raw:GT=hom:DP=71:FQ=1:SQ=-1;ID=S2504_raw:GT=hom:DP=64:FQ=1:SQ=-1;ID=S2008_raw:GT=hom:DP=40:FQ=1:SQ=-1;ID=S2509_raw:GT=hom:DP=84:FQ=1:SQ=-1;ID=S2508_raw:GT=hom:DP=77:FQ=1:SQ=-1;ID=S2666_raw:GT=hom:DP=98:FQ=1:SQ=-1;ID=S2669_raw:GT=hom:DP=58:FQ=1:SQ=-1;ID=S2166_raw:GT=hom:DP=55:FQ=1:SQ=-1;ID=S1775_raw:GT=hom:DP=46:FQ=1:SQ=-1;ID=S2501_raw:GT=hom:DP=74:FQ=1:SQ=-1;ID=S2500_raw:GT=hom:DP=84:FQ=1:SQ=-1;ID=S2502_raw:GT=hom:DP=93:FQ=1:SQ=-1;ID=S2505_raw:GT=hom:DP=70:FQ=1:SQ=-1;ID=S2176_raw:GT=hom:DP=57:FQ=1:SQ=-1;ID=S2506_raw:GT=hom:DP=41:FQ=1:SQ=-1;ID=S2667_raw:GT=hom:DP=55:FQ=1:SQ=-1;
3	1,23E+08	1,23E+08	C	T	intronic	MYLK	.	.	rs2168439	.	ID=S2009_raw:GT=hom:DP=91:FQ=1:SQ=-1;ID=S2503_raw:GT=hom:DP=144:FQ=1:SQ=-1;ID=S2507_raw:GT=hom:DP=109:FQ=1:SQ=-1;ID=S2668_raw:GT=hom:DP=155:FQ=0.99:SQ=-1;ID=S2012_raw:GT=hom:DP=106:FQ=1:SQ=-1;ID=S2010_raw:GT=hom:DP=110:FQ=1:SQ=-1;ID=S2662_raw:GT=hom:DP=142:FQ=1:SQ=-1;ID=S2663_raw:GT=hom:DP=146:FQ=1:SQ=-1;ID=S2011_raw:GT=hom:DP=170:FQ=1:SQ=-1;ID=S2504_raw:GT=hom:DP=170:FQ=1:SQ=-1;ID=S2008_raw:GT=hom:DP=117:FQ=1:SQ=-1;ID=S2509_raw:GT=hom:DP=125:FQ=1:SQ=-1;ID=S2508_raw:GT=hom:DP=161:FQ=1:SQ=-1;ID=S2666_raw:GT=hom:DP=185:FQ=1:SQ=-1;ID=S2669_raw:GT=hom:DP=138:FQ=1:SQ=-1;ID=S2166_raw:GT=hom:DP=100:FQ=1:SQ=-1;ID=S1775_raw:GT=hom:DP=75:FQ=1:SQ=-1;ID=S2501_raw:GT=hom:DP=134:FQ=1:SQ=-1;ID=S2500_raw:GT=hom:DP=131:FQ=1:SQ=-1;ID=S2502_raw:GT=hom:DP=163:FQ=1:SQ=-1;ID=S2505_raw:GT=hom:DP=121:FQ=1:SQ=-1;ID=S2176_raw:GT=hom:DP=117:FQ=1:SQ=-1;ID=S2506_raw:GT=hom:DP=88:FQ=1:SQ=-1;ID=S2667_raw:GT=hom:DP=138:FQ=1:SQ=-1;
3	1,23E+08	1,23E+08	C	A	intronic	MYLK	.	.	rs2198766	.	ID=S2009_raw:GT=hom:DP=12:FQ=1:SQ=-1;ID=S2503_raw:GT=hom:DP=42:FQ=1:SQ=-1;ID=S2507_raw:GT=hom:DP=36:FQ=1:SQ=-1;ID=S2668_raw:GT=hom:DP=44:FQ=1:SQ=-1;ID=S2012_raw:GT=hom:DP=9:FQ=1:SQ=-1;ID=S2010_raw:GT=hom:DP=6:FQ=1:SQ=-1;ID=S2662_raw:GT=hom:DP=21:FQ=1:SQ=-1;ID=S2663_raw:GT=hom:DP=37:FQ=1:SQ=-1;ID=S2011_raw:GT=hom:DP=4:FQ=1:SQ=-1;ID=S2504_raw:GT=hom:DP=37:FQ=1:SQ=-1;ID=S2008_raw:GT=hom:DP=7:FQ=1:SQ=-1;ID=S2509_raw:GT=hom:DP=28:FQ=1:SQ=-1;ID=S2508_raw:GT=hom:DP=41:FQ=1:SQ=-1;ID=S2666_raw:GT=hom:DP=53:FQ=1:SQ=-1;ID=S2669_raw:GT=hom:DP=34:FQ=1:SQ=-1;ID=S2166_raw:GT=hom:DP=52:FQ=1:SQ=-1;ID=S1775_raw:GT=hom:DP=34:FQ=1:SQ=-1;ID=S2501_raw:GT=hom:DP=40:FQ=1:SQ=-1;ID=S2500_raw:GT=hom:DP=37:FQ=1:SQ=-1;ID=S2502_raw:GT=hom:DP=41:FQ=1:SQ=-1;ID=S2505_raw:GT=hom:DP=47:FQ=1:SQ=-1;ID=S2176_raw:GT=hom:DP=61:FQ=1:SQ=-1;ID=S2506_raw:GT=hom:DP=49:FQ=1:SQ=-1;ID=S2667_raw:GT=hom:DP=43:FQ=1:SQ=-1;
3	1,23E+08	1,23E+08	G	A	exonic	MYLK	nonsynonymous SNV	MYLK:NM_053025:exon7:c.439C>T:p.P147S,MYLK:NM_053026:exon7:c.439C>T:p.P147S,MYLK:NM_053027:exon7:c.439C>T:p.P147S,MYLK:NM_053028:exon7:c.439C>T:p.P147S	rs9840993	Benign/Likely_benign	ID=S2009_raw:GT=hom:DP=52:FQ=1:SQ=-1;ID=S2503_raw:GT=hom:DP=169:FQ=1:SQ=-1;ID=S2507_raw:GT=hom:DP=158:FQ=1:SQ=-1;ID=S2668_raw:GT=hom:DP=221:FQ=1:SQ=-1;ID=S2012_raw:GT=hom:DP=52:FQ=1:SQ=-1;ID=S2010_raw:GT=hom:DP=38:FQ=1:SQ=-1;ID=S2662_raw:GT=hom:DP=123:FQ=1:SQ=-1;ID=S2663_raw:GT=hom:DP=179:FQ=1:SQ=-1;ID=S2011_raw:GT=hom:DP=47:FQ=1:SQ=-1;ID=S2504_raw:GT=hom:DP=197:FQ=0.99:SQ=-1;ID=S2008_raw:GT=hom:DP=47:FQ=1:SQ=-1;ID=S2509_raw:GT=hom:DP=183:FQ=1:SQ=-1;ID=S2508_raw:GT=hom:DP=168:FQ=1:SQ=-1;ID=S2666_raw:GT=hom:DP=161:FQ=1:SQ=-1;ID=S2669_raw:GT=hom:DP=141:FQ=1:SQ=-1;ID=S2166_raw:GT=hom:DP=167:FQ=1:SQ=-1;ID=S1775_raw:GT=hom:DP=120:FQ=1:SQ=-1;ID=S2501_raw:GT=hom:DP=147:FQ=1:SQ=-1;ID=S2500_raw:GT=hom:DP=152:FQ=1:SQ=-1;ID=S2502_raw:GT=hom:DP=184:FQ=1:SQ=-1;ID=S2505_raw:GT=hom:DP=158:FQ=1:SQ=-1;ID=S2176_raw:GT=hom:DP=197:FQ=1:SQ=-1;ID=S2506_raw:GT=hom:DP=138:FQ=1:SQ=-1;ID=S2667_raw:GT=hom:DP=133:FQ=1:SQ=-1;
3	1,23E+08	1,23E+08	T	C	intronic	MYLK	.	.	rs9825707	.	ID=S2009_raw:GT=hom:DP=1:FQ=1:SQ=-1;ID=S2503_raw:GT=hom:DP=6:FQ=1:SQ=-1;ID=S2507_raw:GT=hom:DP=9:FQ=1:SQ=-1;ID=S2668_raw:GT=hom:DP=7:FQ=1:SQ=-1;ID=S2012_raw:GT=hom:DP=1:FQ=1:SQ=-1;ID=S2010_raw:GT=hom:DP=2:FQ=1:SQ=-1;ID=S2662_raw:GT=hom:DP=5:FQ=1:SQ=-1;ID=S2663_raw:GT=hom:DP=7:FQ=1:SQ=-1;ID=S2011_raw:GT=hom:DP=2:FQ=1:SQ=-1;ID=S2504_raw:GT=hom:DP=2:FQ=1:SQ=-1;ID=S2008_raw:GT=hom:DP=2:FQ=1:SQ=-1;ID=S2509_raw:GT=hom:DP=5:FQ=1:SQ=-1;ID=S2508_raw:GT=hom:DP=4:FQ=1:SQ=-1;ID=S2666_raw:GT=hom:DP=4:FQ=1:SQ=-1;ID=S2669_raw:GT=hom:DP=5:FQ=1:SQ=-1;ID=S2166_raw:GT=hom:DP=10:FQ=1:SQ=-1;ID=S1775_raw:GT=hom:DP=2:FQ=1:SQ=-1;ID=S2501_raw:GT=hom:DP=5:FQ=1:SQ=-1;ID=S2500_raw:GT=hom:DP=6:FQ=1:SQ=-1;ID=S2502_raw:GT=hom:DP=3:FQ=1:SQ=-1;ID=S2505_raw:GT=hom:DP=3:FQ=1:SQ=-1;ID=S2176_raw:GT=hom:DP=10:FQ=0.9:SQ=-1;ID=S2506_raw:GT=hom:DP=3:FQ=1:SQ=-1;ID=S2667_raw:GT=hom:DP=4:FQ=1:SQ=-1;
15	59524056	59524056	T	C	intronic	MYO1E	.	.	rs7165587	.	ID=S2009_raw:GT=hom:DP=7:FQ=1:SQ=-1;ID=S2503_raw:GT=hom:DP=53:FQ=1:SQ=-1;ID=S2507_raw:GT=hom:DP=33:FQ=1:SQ=-1;ID=S2668_raw:GT=hom:DP=57:FQ=1:SQ=-1;ID=S2012_raw:GT=hom:DP=12:FQ=1:SQ=-1;ID=S2010_raw:GT=hom:DP=12:FQ=1:SQ=-1;ID=S2662_raw:GT=hom:DP=40:FQ=1:SQ=-1;ID=S2663_raw:GT=hom:DP=33:FQ=1:SQ=-1;ID=S2011_raw:GT=hom:DP=18:FQ=1:SQ=-1;ID=S2504_raw:GT=hom:DP=34:FQ=1:SQ=-1;ID=S2008_raw:GT=hom:DP=6:FQ=1:SQ=-1;ID=S2509_raw:GT=hom:DP=44:FQ=1:SQ=-1;ID=S2508_raw:GT=hom:DP=40:FQ=1:SQ=-1;ID=S2666_raw:GT=hom:DP=36:FQ=1:SQ=-1;ID=S2669_raw:GT=hom:DP=37:FQ=1:SQ=-1;ID=S2166_raw:GT=hom:DP=38:FQ=1:SQ=-1;ID=S1775_raw:GT=hom:DP=34:FQ=1:SQ=-1;ID=S2501_raw:GT=hom:DP=30:FQ=1:SQ=-1;ID=S2500_raw:GT=hom:DP=49:FQ=1:SQ=-1;ID=S2502_raw:GT=hom:DP=62:FQ=1:SQ=-1;ID=S2505_raw:GT=hom:DP=58:FQ=0.98:SQ=-1;ID=S2176_raw:GT=hom:DP=45:FQ=1:SQ=-1;ID=S2506_raw:GT=hom:DP=27:FQ=1:SQ=-1;ID=S2667_raw:GT=hom:DP=24:FQ=1:SQ=-1;
15	59548475	59548475	C	T	splicing	MYO1E	.	NM_004998:exon4:c.332+8G>A	rs4508371	.	ID=S2009_raw:GT=hom:DP=96:FQ=1:SQ=-1;ID=S2503_raw:GT=hom:DP=171:FQ=1:SQ=-1;ID=S2507_raw:GT=hom:DP=150:FQ=1:SQ=-1;ID=S2668_raw:GT=hom:DP=227:FQ=1:SQ=-1;ID=S2012_raw:GT=hom:DP=103:FQ=1:SQ=-1;ID=S2010_raw:GT=hom:DP=130:FQ=1:SQ=-1;ID=S2662_raw:GT=hom:DP=13:FQ=1:SQ=-1;ID=S2663_raw:GT=hom:DP=160:FQ=1:SQ=-1;ID=S2011_raw:GT=hom:DP=119:FQ=1:SQ=-1;ID=S2504_raw:GT=hom:DP=168:FQ=1:SQ=-1;ID=S2008_raw:GT=hom:DP=95:FQ=1:SQ=-1;ID=S2509_raw:GT=hom:DP=171:FQ=1:SQ=-1;ID=S2508_raw:GT=hom:DP=174:FQ=1:SQ=-1;ID=S2666_raw:GT=hom:DP=251:FQ=1:SQ=-1;ID=S2669_raw:GT=hom:DP=130:FQ=1:SQ=-1;ID=S2166_raw:GT=hom:DP=316:FQ=1:SQ=-1;ID=S1775_raw:GT=hom:DP=204:FQ=1:SQ=-1;ID=S2501_raw:GT=hom:DP=146:FQ=1:SQ=-1;ID=S2500_raw:GT=hom:DP=161:FQ=1:SQ=-1;ID=S2502_raw:GT=hom:DP=190:FQ=1:SQ=-1;ID=S2505_raw:GT=hom:DP=126:FQ=1:SQ=-1;ID=S2176_raw:GT=hom:DP=351:FQ=1:SQ=-1;ID=S2506_raw:GT=hom:DP=182:FQ=1:SQ=-1;ID=S2667_raw:GT=hom:DP=165:FQ=1:SQ=-1;
15	59664607	59664607	C	A	intronic	MYO1E	.	.	rs7175010	.	ID=S2009_raw:GT=hom:DP=8:FQ=1:SQ=-1;ID=S2503_raw:GT=hom:DP=8:FQ=1:SQ=-1;ID=S2507_raw:GT=hom:DP=15:FQ=1:SQ=-1;ID=S2668_raw:GT=hom:DP=3:FQ=1:SQ=-1;ID=S2012_raw:GT=hom:DP=2:FQ=1:SQ=-1;ID=S2010_raw:GT=hom:DP=6:FQ=1:SQ=-1;ID=S2662_raw:GT=hom:DP=18:FQ=1:SQ=-1;ID=S2663_raw:GT=hom:DP=5:FQ=1:SQ=-1;ID=S2011_raw:GT=hom:DP=4:FQ=1:SQ=-1;ID=S2504_raw:GT=hom:DP=8:FQ=1:SQ=-1;ID=S2008_raw:GT=hom:DP=9:FQ=1:SQ=-1;ID=S2509_raw:GT=hom:DP=9:FQ=1:SQ=-1;ID=S2508_raw:GT=hom:DP=10:FQ=1:SQ=-1;ID=S2666_raw:GT=hom:DP=4:FQ=1:SQ=-1;ID=S2669_raw:GT=hom:DP=13:FQ=1:SQ=-1;ID=S2166_raw:GT=hom:DP=14:FQ=1:SQ=-1;ID=S1775_raw:GT=hom:DP=3:FQ=1:SQ=-1;ID=S2501_raw:GT=hom:DP=3:FQ=1:SQ=-1;ID=S2500_raw:GT=hom:DP=10:FQ=1:SQ=-1;ID=S2502_raw:GT=hom:DP=10:FQ=1:SQ=-1;ID=S2505_raw:GT=hom:DP=11:FQ=1:SQ=-1;ID=S2176_raw:GT=hom:DP=16:FQ=1:SQ=-1;ID=S2506_raw:GT=hom:DP=8:FQ=1:SQ=-1;ID=S2667_raw:GT=hom:DP=10:FQ=1:SQ=-1;
18	47563299	47563299	T	C	exonic	MYO5B	nonsynonymous SNV	MYO5B:NM_001080467:exon4:c.376A>G:p.T126A	rs1815930	Benign	ID=S2009_raw:GT=hom:DP=29:FQ=1:SQ=-1;ID=S2503_raw:GT=hom:DP=105:FQ=1:SQ=-1;ID=S2507_raw:GT=hom:DP=92:FQ=1:SQ=-1;ID=S2668_raw:GT=hom:DP=124:FQ=1:SQ=-1;ID=S2012_raw:GT=hom:DP=36:FQ=1:SQ=-1;ID=S2010_raw:GT=hom:DP=43:FQ=1:SQ=-1;ID=S2662_raw:GT=hom:DP=19:FQ=1:SQ=-1;ID=S2663_raw:GT=hom:DP=80:FQ=1:SQ=-1;ID=S2011_raw:GT=hom:DP=41:FQ=1:SQ=-1;ID=S2504_raw:GT=hom:DP=92:FQ=1:SQ=-1;ID=S2008_raw:GT=hom:DP=27:FQ=1:SQ=-1;ID=S2509_raw:GT=hom:DP=92:FQ=1:SQ=-1;ID=S2508_raw:GT=hom:DP=93:FQ=1:SQ=-1;ID=S2666_raw:GT=hom:DP=86:FQ=1:SQ=-1;ID=S2669_raw:GT=hom:DP=96:FQ=1:SQ=-1;ID=S2166_raw:GT=hom:DP=176:FQ=1:SQ=-1;ID=S1775_raw:GT=hom:DP=109:FQ=1:SQ=-1;ID=S2501_raw:GT=hom:DP=79:FQ=1:SQ=-1;ID=S2500_raw:GT=hom:DP=99:FQ=1:SQ=-1;ID=S2502_raw:GT=hom:DP=103:FQ=1:SQ=-1;ID=S2505_raw:GT=hom:DP=81:FQ=1:SQ=-1;ID=S2176_raw:GT=hom:DP=184:FQ=0.99:SQ=-1;ID=S2506_raw:GT=hom:DP=69:FQ=1:SQ=-1;ID=S2667_raw:GT=hom:DP=79:FQ=0.99:SQ=-1;
18	47563534	47563534	A	G	intronic	MYO5B	.	.	rs9955800	.	ID=S2009_raw:GT=hom:DP=4:FQ=1:SQ=-1;ID=S2503_raw:GT=hom:DP=6:FQ=1:SQ=-1;ID=S2507_raw:GT=hom:DP=5:FQ=1:SQ=-1;ID=S2668_raw:GT=hom:DP=4:FQ=1:SQ=-1;ID=S2012_raw:GT=hom:DP=5:FQ=1:SQ=-1;ID=S2010_raw:GT=hom:DP=3:FQ=1:SQ=-1;ID=S2662_raw:GT=hom:DP=1:FQ=1:SQ=-1;ID=S2663_raw:GT=hom:DP=2:FQ=1:SQ=-1;ID=S2011_raw:GT=hom:DP=8:FQ=1:SQ=-1;ID=S2504_raw:GT=hom:DP=3:FQ=1:SQ=-1;ID=S2008_raw:GT=hom:DP=10:FQ=1:SQ=-1;ID=S2509_raw:GT=hom:DP=2:FQ=1:SQ=-1;ID=S2508_raw:GT=hom:DP=4:FQ=1:SQ=-1;ID=S2666_raw:GT=hom:DP=6:FQ=1:SQ=-1;ID=S2669_raw:GT=hom:DP=6:FQ=1:SQ=-1;ID=S2166_raw:GT=hom:DP=24:FQ=1:SQ=-1;ID=S1775_raw:GT=hom:DP=14:FQ=1:SQ=-1;ID=S2501_raw:GT=hom:DP=5:FQ=1:SQ=-1;ID=S2500_raw:GT=hom:DP=9:FQ=1:SQ=-1;ID=S2502_raw:GT=hom:DP=6:FQ=1:SQ=-1;ID=S2505_raw:GT=hom:DP=3:FQ=1:SQ=-1;ID=S2176_raw:GT=hom:DP=22:FQ=1:SQ=-1;ID=S2506_raw:GT=hom:DP=7:FQ=1:SQ=-1;ID=S2667_raw:GT=hom:DP=5:FQ=1:SQ=-1;
11	71174589	71174589	T	C	intronic	NADSYN1	.	.	rs1629304	.	ID=S2009_raw:GT=hom:DP=21:FQ=1:SQ=-1;ID=S2503_raw:GT=hom:DP=71:FQ=1:SQ=-1;ID=S2507_raw:GT=hom:DP=58:FQ=1:SQ=-1;ID=S2668_raw:GT=hom:DP=62:FQ=1:SQ=-1;ID=S2012_raw:GT=hom:DP=17:FQ=1:SQ=-1;ID=S2010_raw:GT=hom:DP=26:FQ=1:SQ=-1;ID=S2662_raw:GT=hom:DP=80:FQ=1:SQ=-1;ID=S2663_raw:GT=hom:DP=33:FQ=1:SQ=-1;ID=S2011_raw:GT=hom:DP=32:FQ=1:SQ=-1;ID=S2504_raw:GT=hom:DP=53:FQ=1:SQ=-1;ID=S2008_raw:GT=hom:DP=24:FQ=1:SQ=-1;ID=S2509_raw:GT=hom:DP=76:FQ=1:SQ=-1;ID=S2508_raw:GT=hom:DP=60:FQ=1:SQ=-1;ID=S2666_raw:GT=hom:DP=31:FQ=1:SQ=-1;ID=S2669_raw:GT=hom:DP=43:FQ=1:SQ=-1;ID=S2166_raw:GT=hom:DP=52:FQ=1:SQ=-1;ID=S1775_raw:GT=hom:DP=32:FQ=1:SQ=-1;ID=S2501_raw:GT=hom:DP=61:FQ=1:SQ=-1;ID=S2500_raw:GT=hom:DP=64:FQ=1:SQ=-1;ID=S2502_raw:GT=hom:DP=61:FQ=1:SQ=-1;ID=S2505_raw:GT=hom:DP=59:FQ=1:SQ=-1;ID=S2176_raw:GT=hom:DP=58:FQ=1:SQ=-1;ID=S2506_raw:GT=hom:DP=56:FQ=1:SQ=-1;ID=S2667_raw:GT=hom:DP=45:FQ=1:SQ=-1;
11	71184678	71184678	A	C	exonic	NADSYN1	nonsynonymous SNV	NADSYN1:NM_018161:exon8:c.612A>C:p.Q204H	rs7950441	.	ID=S2009_raw:GT=hom:DP=80:FQ=1:SQ=-1;ID=S2503_raw:GT=hom:DP=157:FQ=1:SQ=-1;ID=S2507_raw:GT=hom:DP=163:FQ=1:SQ=-1;ID=S2668_raw:GT=hom:DP=169:FQ=1:SQ=-1;ID=S2012_raw:GT=hom:DP=90:FQ=1:SQ=-1;ID=S2010_raw:GT=hom:DP=77:FQ=1:SQ=-1;ID=S2662_raw:GT=hom:DP=287:FQ=1:SQ=-1;ID=S2663_raw:GT=hom:DP=155:FQ=1:SQ=-1;ID=S2011_raw:GT=hom:DP=117:FQ=1:SQ=-1;ID=S2504_raw:GT=hom:DP=159:FQ=1:SQ=-1;ID=S2008_raw:GT=hom:DP=94:FQ=1:SQ=-1;ID=S2509_raw:GT=hom:DP=173:FQ=1:SQ=-1;ID=S2508_raw:GT=hom:DP=170:FQ=1:SQ=-1;ID=S2666_raw:GT=hom:DP=141:FQ=1:SQ=-1;ID=S2669_raw:GT=hom:DP=125:FQ=1:SQ=-1;ID=S2166_raw:GT=hom:DP=89:FQ=1:SQ=-1;ID=S1775_raw:GT=hom:DP=80:FQ=1:SQ=-1;ID=S2501_raw:GT=hom:DP=159:FQ=1:SQ=-1;ID=S2500_raw:GT=hom:DP=139:FQ=1:SQ=-1;ID=S2502_raw:GT=hom:DP=199:FQ=1:SQ=-1;ID=S2505_raw:GT=hom:DP=125:FQ=1:SQ=-1;ID=S2176_raw:GT=hom:DP=107:FQ=1:SQ=-1;ID=S2506_raw:GT=hom:DP=149:FQ=1:SQ=-1;ID=S2667_raw:GT=hom:DP=130:FQ=1:SQ=-1;
11	67799731	67799731	C	G	intronic	NDUFS8	.	.	rs3133266	.	ID=S2009_raw:GT=hom:DP=59:FQ=1:SQ=-1;ID=S2503_raw:GT=hom:DP=55:FQ=1:SQ=-1;ID=S2507_raw:GT=hom:DP=50:FQ=1:SQ=-1;ID=S2668_raw:GT=hom:DP=115:FQ=1:SQ=-1;ID=S2012_raw:GT=hom:DP=59:FQ=1:SQ=-1;ID=S2010_raw:GT=hom:DP=65:FQ=1:SQ=-1;ID=S2662_raw:GT=hom:DP=198:FQ=1:SQ=-1;ID=S2663_raw:GT=hom:DP=92:FQ=1:SQ=-1;ID=S2011_raw:GT=hom:DP=89:FQ=1:SQ=-1;ID=S2504_raw:GT=hom:DP=58:FQ=1:SQ=-1;ID=S2008_raw:GT=hom:DP=88:FQ=1:SQ=-1;ID=S2509_raw:GT=hom:DP=51:FQ=1:SQ=-1;ID=S2508_raw:GT=hom:DP=51:FQ=1:SQ=-1;ID=S2666_raw:GT=hom:DP=76:FQ=1:SQ=-1;ID=S2669_raw:GT=hom:DP=96:FQ=1:SQ=-1;ID=S2166_raw:GT=hom:DP=84:FQ=1:SQ=-1;ID=S1775_raw:GT=hom:DP=26:FQ=1:SQ=-1;ID=S2501_raw:GT=hom:DP=71:FQ=1:SQ=-1;ID=S2500_raw:GT=hom:DP=58:FQ=1:SQ=-1;ID=S2502_raw:GT=hom:DP=58:FQ=1:SQ=-1;ID=S2505_raw:GT=hom:DP=55:FQ=1:SQ=-1;ID=S2176_raw:GT=hom:DP=69:FQ=1:SQ=-1;ID=S2506_raw:GT=hom:DP=55:FQ=1:SQ=-1;ID=S2667_raw:GT=hom:DP=91:FQ=1:SQ=-1;
11	67800494	67800494	T	G	intronic	NDUFS8	.	.	rs3115545	Benign	ID=S2009_raw:GT=hom:DP=70:FQ=1:SQ=-1;ID=S2503_raw:GT=hom:DP=147:FQ=1:SQ=-1;ID=S2507_raw:GT=hom:DP=173:FQ=0.99:SQ=-1;ID=S2668_raw:GT=hom:DP=188:FQ=1:SQ=-1;ID=S2012_raw:GT=hom:DP=72:FQ=1:SQ=-1;ID=S2010_raw:GT=hom:DP=89:FQ=1:SQ=-1;ID=S2662_raw:GT=hom:DP=321:FQ=1:SQ=-1;ID=S2663_raw:GT=hom:DP=165:FQ=1:SQ=-1;ID=S2011_raw:GT=hom:DP=114:FQ=1:SQ=-1;ID=S2504_raw:GT=hom:DP=197:FQ=1:SQ=-1;ID=S2008_raw:GT=hom:DP=128:FQ=1:SQ=-1;ID=S2509_raw:GT=hom:DP=181:FQ=1:SQ=-1;ID=S2508_raw:GT=hom:DP=183:FQ=1:SQ=-1;ID=S2666_raw:GT=hom:DP=164:FQ=1:SQ=-1;ID=S2669_raw:GT=hom:DP=198:FQ=0.99:SQ=-1;ID=S2166_raw:GT=hom:DP=156:FQ=1:SQ=-1;ID=S1775_raw:GT=hom:DP=86:FQ=1:SQ=-1;ID=S2501_raw:GT=hom:DP=137:FQ=1:SQ=-1;ID=S2500_raw:GT=hom:DP=171:FQ=1:SQ=-1;ID=S2502_raw:GT=hom:DP=183:FQ=1:SQ=-1;ID=S2505_raw:GT=hom:DP=165:FQ=1:SQ=-1;ID=S2176_raw:GT=hom:DP=132:FQ=1:SQ=-1;ID=S2506_raw:GT=hom:DP=133:FQ=1:SQ=-1;ID=S2667_raw:GT=hom:DP=185:FQ=0.99:SQ=-1;
11	67801282	67801282	C	T	intronic	NDUFS8	.	.	rs3133267	.	ID=S2009_raw:GT=hom:DP=7:FQ=1:SQ=-1;ID=S2503_raw:GT=hom:DP=17:FQ=1:SQ=-1;ID=S2507_raw:GT=hom:DP=19:FQ=1:SQ=-1;ID=S2668_raw:GT=hom:DP=9:FQ=1:SQ=-1;ID=S2012_raw:GT=hom:DP=11:FQ=1:SQ=-1;ID=S2010_raw:GT=hom:DP=6:FQ=1:SQ=-1;ID=S2662_raw:GT=hom:DP=35:FQ=1:SQ=-1;ID=S2663_raw:GT=hom:DP=15:FQ=1:SQ=-1;ID=S2011_raw:GT=hom:DP=9:FQ=1:SQ=-1;ID=S2504_raw:GT=hom:DP=25:FQ=1:SQ=-1;ID=S2008_raw:GT=hom:DP=7:FQ=1:SQ=-1;ID=S2509_raw:GT=hom:DP=16:FQ=1:SQ=-1;ID=S2508_raw:GT=hom:DP=22:FQ=1:SQ=-1;ID=S2666_raw:GT=hom:DP=13:FQ=1:SQ=-1;ID=S2669_raw:GT=hom:DP=14:FQ=1:SQ=-1;ID=S2166_raw:GT=hom:DP=32:FQ=1:SQ=-1;ID=S1775_raw:GT=hom:DP=18:FQ=1:SQ=-1;ID=S2501_raw:GT=hom:DP=25:FQ=1:SQ=-1;ID=S2500_raw:GT=hom:DP=21:FQ=1:SQ=-1;ID=S2502_raw:GT=hom:DP=28:FQ=1:SQ=-1;ID=S2505_raw:GT=hom:DP=25:FQ=1:SQ=-1;ID=S2176_raw:GT=hom:DP=28:FQ=1:SQ=-1;ID=S2506_raw:GT=hom:DP=21:FQ=1:SQ=-1;ID=S2667_raw:GT=hom:DP=13:FQ=1:SQ=-1;
11	67801517	67801517	G	T	intronic	NDUFS8	.	.	rs3115546	.	ID=S2009_raw:GT=hom:DP=66:FQ=1:SQ=-1;ID=S2503_raw:GT=hom:DP=170:FQ=1:SQ=-1;ID=S2507_raw:GT=hom:DP=160:FQ=1:SQ=-1;ID=S2668_raw:GT=hom:DP=176:FQ=1:SQ=-1;ID=S2012_raw:GT=hom:DP=56:FQ=1:SQ=-1;ID=S2010_raw:GT=hom:DP=51:FQ=1:SQ=-1;ID=S2662_raw:GT=hom:DP=238:FQ=1:SQ=-1;ID=S2663_raw:GT=hom:DP=117:FQ=1:SQ=-1;ID=S2011_raw:GT=hom:DP=66:FQ=1:SQ=-1;ID=S2504_raw:GT=hom:DP=162:FQ=1:SQ=-1;ID=S2008_raw:GT=hom:DP=56:FQ=1:SQ=-1;ID=S2509_raw:GT=hom:DP=132:FQ=1:SQ=-1;ID=S2508_raw:GT=hom:DP=168:FQ=1:SQ=-1;ID=S2666_raw:GT=hom:DP=182:FQ=1:SQ=-1;ID=S2669_raw:GT=hom:DP=123:FQ=1:SQ=-1;ID=S2166_raw:GT=hom:DP=155:FQ=1:SQ=-1;ID=S1775_raw:GT=hom:DP=109:FQ=1:SQ=-1;ID=S2501_raw:GT=hom:DP=170:FQ=1:SQ=-1;ID=S2500_raw:GT=hom:DP=127:FQ=1:SQ=-1;ID=S2502_raw:GT=hom:DP=166:FQ=1:SQ=-1;ID=S2505_raw:GT=hom:DP=130:FQ=1:SQ=-1;ID=S2176_raw:GT=hom:DP=174:FQ=1:SQ=-1;ID=S2506_raw:GT=hom:DP=134:FQ=1:SQ=-1;ID=S2667_raw:GT=hom:DP=206:FQ=1:SQ=-1;
11	67375162	67375162	T	C	intronic	NDUFV1	.	.	rs1382089	.	ID=S2009_raw:GT=hom:DP=3:FQ=1:SQ=-1;ID=S2503_raw:GT=hom:DP=29:FQ=1:SQ=-1;ID=S2507_raw:GT=hom:DP=17:FQ=1:SQ=-1;ID=S2668_raw:GT=hom:DP=21:FQ=1:SQ=-1;ID=S2012_raw:GT=hom:DP=2:FQ=1:SQ=-1;ID=S2010_raw:GT=hom:DP=1:FQ=1:SQ=-1;ID=S2662_raw:GT=hom:DP=50:FQ=1:SQ=-1;ID=S2663_raw:GT=hom:DP=23:FQ=1:SQ=-1;ID=S2011_raw:GT=hom:DP=3:FQ=1:SQ=-1;ID=S2504_raw:GT=hom:DP=27:FQ=1:SQ=-1;ID=S2008_raw:GT=hom:DP=1:FQ=1:SQ=-1;ID=S2509_raw:GT=hom:DP=25:FQ=1:SQ=-1;ID=S2508_raw:GT=hom:DP=41:FQ=1:SQ=-1;ID=S2666_raw:GT=hom:DP=18:FQ=1:SQ=-1;ID=S2669_raw:GT=hom:DP=30:FQ=1:SQ=-1;ID=S2166_raw:GT=hom:DP=13:FQ=1:SQ=-1;ID=S1775_raw:GT=hom:DP=6:FQ=1:SQ=-1;ID=S2501_raw:GT=hom:DP=24:FQ=1:SQ=-1;ID=S2500_raw:GT=hom:DP=31:FQ=1:SQ=-1;ID=S2502_raw:GT=hom:DP=13:FQ=1:SQ=-1;ID=S2505_raw:GT=hom:DP=36:FQ=1:SQ=-1;ID=S2176_raw:GT=hom:DP=20:FQ=1:SQ=-1;ID=S2506_raw:GT=hom:DP=23:FQ=1:SQ=-1;ID=S2667_raw:GT=hom:DP=27:FQ=1:SQ=-1;
11	67378229	67378229	T	G	intronic	NDUFV1	.	.	rs3016861	.	ID=S2009_raw:GT=hom:DP=1:FQ=1:SQ=-1;ID=S2503_raw:GT=hom:DP=3:FQ=1:SQ=-1;ID=S2507_raw:GT=hom:DP=3:FQ=1:SQ=-1;ID=S2668_raw:GT=hom:DP=5:FQ=1:SQ=-1;ID=S2012_raw:GT=hom:DP=2:FQ=1:SQ=-1;ID=S2010_raw:GT=hom:DP=1:FQ=1:SQ=-1;ID=S2662_raw:GT=hom:DP=11:FQ=1:SQ=-1;ID=S2663_raw:GT=hom:DP=6:FQ=1:SQ=-1;ID=S2011_raw:GT=hom:DP=3:FQ=1:SQ=-1;ID=S2504_raw:GT=hom:DP=6:FQ=1:SQ=-1;ID=S2008_raw:GT=hom:DP=6:FQ=1:SQ=-1;ID=S2509_raw:GT=hom:DP=6:FQ=1:SQ=-1;ID=S2508_raw:GT=hom:DP=10:FQ=1:SQ=-1;ID=S2666_raw:GT=hom:DP=3:FQ=1:SQ=-1;ID=S2669_raw:GT=hom:DP=2:FQ=1:SQ=-1;ID=S2166_raw:GT=hom:DP=8:FQ=1:SQ=-1;ID=S1775_raw:GT=hom:DP=4:FQ=1:SQ=-1;ID=S2501_raw:GT=hom:DP=2:FQ=1:SQ=-1;ID=S2500_raw:GT=hom:DP=3:FQ=1:SQ=-1;ID=S2502_raw:GT=hom:DP=4:FQ=1:SQ=-1;ID=S2505_raw:GT=hom:DP=3:FQ=1:SQ=-1;ID=S2176_raw:GT=hom:DP=8:FQ=1:SQ=-1;ID=S2506_raw:GT=hom:DP=1:FQ=1:SQ=-1;ID=S2667_raw:GT=hom:DP=1:FQ=1:SQ=-1;
15	56299364	56299364	G	A	intergenic	NEDD4;RFX7	.	dist=13420;dist=80114	rs62046380	.	ID=S2009_raw:GT=hom:DP=29:FQ=1:SQ=-1;ID=S2503_raw:GT=hom:DP=17:FQ=1:SQ=-1;ID=S2507_raw:GT=hom:DP=3:FQ=1:SQ=-1;ID=S2668_raw:GT=hom:DP=9:FQ=1:SQ=-1;ID=S2012_raw:GT=hom:DP=28:FQ=1:SQ=-1;ID=S2010_raw:GT=hom:DP=38:FQ=1:SQ=-1;ID=S2662_raw:GT=hom:DP=14:FQ=1:SQ=-1;ID=S2663_raw:GT=hom:DP=10:FQ=1:SQ=-1;ID=S2011_raw:GT=hom:DP=47:FQ=1:SQ=-1;ID=S2504_raw:GT=hom:DP=11:FQ=1:SQ=-1;ID=S2008_raw:GT=hom:DP=28:FQ=1:SQ=-1;ID=S2509_raw:GT=hom:DP=14:FQ=1:SQ=-1;ID=S2508_raw:GT=hom:DP=6:FQ=1:SQ=-1;ID=S2666_raw:GT=hom:DP=4:FQ=1:SQ=-1;ID=S2669_raw:GT=hom:DP=11:FQ=1:SQ=-1;ID=S2166_raw:GT=hom:DP=6:FQ=1:SQ=-1;ID=S1775_raw:GT=hom:DP=5:FQ=1:SQ=-1;ID=S2501_raw:GT=hom:DP=8:FQ=1:SQ=-1;ID=S2500_raw:GT=hom:DP=14:FQ=1:SQ=-1;ID=S2502_raw:GT=hom:DP=11:FQ=1:SQ=-1;ID=S2505_raw:GT=hom:DP=9:FQ=1:SQ=-1;ID=S2176_raw:GT=hom:DP=12:FQ=1:SQ=-1;ID=S2506_raw:GT=hom:DP=8:FQ=1:SQ=-1;ID=S2667_raw:GT=hom:DP=12:FQ=1:SQ=-1;
4	1,7E+08	1,7E+08	C	T	intronic	NEK1	.	.	rs7661095	.	ID=S2009_raw:GT=hom:DP=3:FQ=1:SQ=-1;ID=S2503_raw:GT=hom:DP=8:FQ=1:SQ=-1;ID=S2507_raw:GT=hom:DP=5:FQ=1:SQ=-1;ID=S2668_raw:GT=hom:DP=4:FQ=1:SQ=-1;ID=S2012_raw:GT=hom:DP=1:FQ=1:SQ=-1;ID=S2010_raw:GT=hom:DP=2:FQ=1:SQ=-1;ID=S2662_raw:GT=hom:DP=2:FQ=1:SQ=-1;ID=S2663_raw:GT=hom:DP=5:FQ=1:SQ=-1;ID=S2011_raw:GT=hom:DP=3:FQ=1:SQ=-1;ID=S2504_raw:GT=hom:DP=7:FQ=1:SQ=-1;ID=S2008_raw:GT=hom:DP=1:FQ=1:SQ=-1;ID=S2509_raw:GT=hom:DP=7:FQ=1:SQ=-1;ID=S2508_raw:GT=hom:DP=4:FQ=1:SQ=-1;ID=S2666_raw:GT=hom:DP=1:FQ=1:SQ=-1;ID=S2669_raw:GT=hom:DP=3:FQ=1:SQ=-1;ID=S2166_raw:GT=hom:DP=5:FQ=1:SQ=-1;ID=S1775_raw:GT=hom:DP=1:FQ=1:SQ=-1;ID=S2501_raw:GT=hom:DP=3:FQ=1:SQ=-1;ID=S2500_raw:GT=hom:DP=5:FQ=1:SQ=-1;ID=S2502_raw:GT=hom:DP=6:FQ=1:SQ=-1;ID=S2505_raw:GT=hom:DP=8:FQ=1:SQ=-1;ID=S2176_raw:GT=hom:DP=4:FQ=1:SQ=-1;ID=S2506_raw:GT=hom:DP=3:FQ=1:SQ=-1;ID=S2667_raw:GT=hom:DP=8:FQ=1:SQ=-1;
1	61892302	61892302	-	G	intronic	NFIA	.	.	.	.	ID=S2009_raw:GT=hom:DP=199:FQ=1:SQ=-1;ID=S2503_raw:GT=hom:DP=63:FQ=1:SQ=-1;ID=S2507_raw:GT=hom:DP=76:FQ=1:SQ=-1;ID=S2668_raw:GT=hom:DP=93:FQ=1:SQ=-1;ID=S2012_raw:GT=hom:DP=198:FQ=1:SQ=-1;ID=S2010_raw:GT=hom:DP=227:FQ=1:SQ=-1;ID=S2662_raw:GT=hom:DP=29:FQ=1:SQ=-1;ID=S2663_raw:GT=hom:DP=68:FQ=1:SQ=-1;ID=S2011_raw:GT=hom:DP=288:FQ=1:SQ=-1;ID=S2504_raw:GT=hom:DP=78:FQ=1:SQ=-1;ID=S2008_raw:GT=hom:DP=234:FQ=1:SQ=-1;ID=S2509_raw:GT=hom:DP=59:FQ=1:SQ=-1;ID=S2508_raw:GT=hom:DP=78:FQ=1:SQ=-1;ID=S2666_raw:GT=hom:DP=78:FQ=1:SQ=-1;ID=S2669_raw:GT=hom:DP=55:FQ=1:SQ=-1;ID=S2166_raw:GT=hom:DP=160:FQ=1:SQ=-1;ID=S1775_raw:GT=hom:DP=113:FQ=1:SQ=-1;ID=S2501_raw:GT=hom:DP=75:FQ=1:SQ=-1;ID=S2500_raw:GT=hom:DP=79:FQ=1:SQ=-1;ID=S2502_raw:GT=hom:DP=74:FQ=1:SQ=-1;ID=S2505_raw:GT=hom:DP=78:FQ=1:SQ=-1;ID=S2176_raw:GT=hom:DP=180:FQ=1:SQ=-1;ID=S2506_raw:GT=hom:DP=40:FQ=1:SQ=-1;ID=S2667_raw:GT=hom:DP=79:FQ=1:SQ=-1;
5	1,57E+08	1,57E+08	G	A	intronic	NIPAL4	.	.	rs4704869	.	ID=S2009_raw:GT=hom:DP=27:FQ=1:SQ=-1;ID=S2503_raw:GT=hom:DP=51:FQ=1:SQ=-1;ID=S2507_raw:GT=hom:DP=76:FQ=1:SQ=-1;ID=S2668_raw:GT=hom:DP=70:FQ=1:SQ=-1;ID=S2012_raw:GT=hom:DP=25:FQ=1:SQ=-1;ID=S2010_raw:GT=hom:DP=33:FQ=1:SQ=-1;ID=S2662_raw:GT=hom:DP=30:FQ=1:SQ=-1;ID=S2663_raw:GT=hom:DP=57:FQ=1:SQ=-1;ID=S2011_raw:GT=hom:DP=38:FQ=1:SQ=-1;ID=S2504_raw:GT=hom:DP=62:FQ=1:SQ=-1;ID=S2008_raw:GT=hom:DP=41:FQ=1:SQ=-1;ID=S2509_raw:GT=hom:DP=47:FQ=1:SQ=-1;ID=S2508_raw:GT=hom:DP=64:FQ=1:SQ=-1;ID=S2666_raw:GT=hom:DP=86:FQ=1:SQ=-1;ID=S2669_raw:GT=hom:DP=54:FQ=1:SQ=-1;ID=S2166_raw:GT=hom:DP=34:FQ=1:SQ=-1;ID=S1775_raw:GT=hom:DP=31:FQ=1:SQ=-1;ID=S2501_raw:GT=hom:DP=58:FQ=1:SQ=-1;ID=S2500_raw:GT=hom:DP=74:FQ=1:SQ=-1;ID=S2502_raw:GT=hom:DP=56:FQ=1:SQ=-1;ID=S2505_raw:GT=hom:DP=71:FQ=1:SQ=-1;ID=S2176_raw:GT=hom:DP=35:FQ=1:SQ=-1;ID=S2506_raw:GT=hom:DP=53:FQ=1:SQ=-1;ID=S2667_raw:GT=hom:DP=67:FQ=1:SQ=-1;
5	1,57E+08	1,57E+08	T	C	exonic	NIPAL4	synonymous SNV	NIPAL4:NM_001172292:exon5:c.1245T>C:p.V415V,NIPAL4:NM_001099287:exon6:c.1302T>C:p.V434V	rs4704870	Benign	ID=S2009_raw:GT=hom:DP=108:FQ=1:SQ=-1;ID=S2503_raw:GT=hom:DP=150:FQ=1:SQ=-1;ID=S2507_raw:GT=hom:DP=160:FQ=0.99:SQ=-1;ID=S2668_raw:GT=hom:DP=168:FQ=1:SQ=-1;ID=S2012_raw:GT=hom:DP=154:FQ=1:SQ=-1;ID=S2010_raw:GT=hom:DP=136:FQ=1:SQ=-1;ID=S2662_raw:GT=hom:DP=141:FQ=1:SQ=-1;ID=S2663_raw:GT=hom:DP=171:FQ=1:SQ=-1;ID=S2011_raw:GT=hom:DP=216:FQ=1:SQ=-1;ID=S2504_raw:GT=hom:DP=208:FQ=1:SQ=-1;ID=S2008_raw:GT=hom:DP=166:FQ=1:SQ=-1;ID=S2509_raw:GT=hom:DP=166:FQ=1:SQ=-1;ID=S2508_raw:GT=hom:DP=159:FQ=1:SQ=-1;ID=S2666_raw:GT=hom:DP=156:FQ=1:SQ=-1;ID=S2669_raw:GT=hom:DP=131:FQ=1:SQ=-1;ID=S2166_raw:GT=hom:DP=260:FQ=1:SQ=-1;ID=S1775_raw:GT=hom:DP=192:FQ=1:SQ=-1;ID=S2501_raw:GT=hom:DP=167:FQ=1:SQ=-1;ID=S2500_raw:GT=hom:DP=177:FQ=1:SQ=-1;ID=S2502_raw:GT=hom:DP=202:FQ=1:SQ=-1;ID=S2505_raw:GT=hom:DP=151:FQ=0.99:SQ=-1;ID=S2176_raw:GT=hom:DP=239:FQ=1:SQ=-1;ID=S2506_raw:GT=hom:DP=146:FQ=1:SQ=-1;ID=S2667_raw:GT=hom:DP=156:FQ=1:SQ=-1;
1	1,62E+08	1,62E+08	T	C	intronic	NOS1AP	.	.	rs348623	.	ID=S2009_raw:GT=hom:DP=6:FQ=1:SQ=-1;ID=S2503_raw:GT=hom:DP=6:FQ=1:SQ=-1;ID=S2507_raw:GT=hom:DP=3:FQ=1:SQ=-1;ID=S2668_raw:GT=hom:DP=10:FQ=1:SQ=-1;ID=S2012_raw:GT=hom:DP=8:FQ=1:SQ=-1;ID=S2010_raw:GT=hom:DP=12:FQ=1:SQ=-1;ID=S2662_raw:GT=hom:DP=3:FQ=1:SQ=-1;ID=S2663_raw:GT=hom:DP=2:FQ=1:SQ=-1;ID=S2011_raw:GT=hom:DP=12:FQ=1:SQ=-1;ID=S2504_raw:GT=hom:DP=4:FQ=1:SQ=-1;ID=S2008_raw:GT=hom:DP=10:FQ=1:SQ=-1;ID=S2509_raw:GT=hom:DP=1:FQ=1:SQ=-1;ID=S2508_raw:GT=hom:DP=7:FQ=1:SQ=-1;ID=S2666_raw:GT=hom:DP=6:FQ=1:SQ=-1;ID=S2669_raw:GT=hom:DP=4:FQ=1:SQ=-1;ID=S2166_raw:GT=hom:DP=6:FQ=1:SQ=-1;ID=S1775_raw:GT=hom:DP=1:FQ=1:SQ=-1;ID=S2501_raw:GT=hom:DP=4:FQ=1:SQ=-1;ID=S2500_raw:GT=hom:DP=7:FQ=1:SQ=-1;ID=S2502_raw:GT=hom:DP=1:FQ=1:SQ=-1;ID=S2505_raw:GT=hom:DP=6:FQ=1:SQ=-1;ID=S2176_raw:GT=hom:DP=8:FQ=1:SQ=-1;ID=S2506_raw:GT=hom:DP=13:FQ=1:SQ=-1;ID=S2667_raw:GT=hom:DP=7:FQ=1:SQ=-1;
1	1,2E+08	1,2E+08	T	C	intronic	NOTCH2	.	.	rs2453057	.	ID=S2009_raw:GT=hom:DP=18:FQ=1:SQ=-1;ID=S2503_raw:GT=hom:DP=9:FQ=1:SQ=-1;ID=S2507_raw:GT=hom:DP=4:FQ=1:SQ=-1;ID=S2668_raw:GT=hom:DP=5:FQ=1:SQ=-1;ID=S2012_raw:GT=hom:DP=22:FQ=1:SQ=-1;ID=S2010_raw:GT=hom:DP=21:FQ=1:SQ=-1;ID=S2662_raw:GT=hom:DP=1:FQ=1:SQ=-1;ID=S2663_raw:GT=hom:DP=4:FQ=1:SQ=-1;ID=S2011_raw:GT=hom:DP=33:FQ=1:SQ=-1;ID=S2504_raw:GT=hom:DP=8:FQ=1:SQ=-1;ID=S2008_raw:GT=hom:DP=22:FQ=1:SQ=-1;ID=S2509_raw:GT=hom:DP=8:FQ=1:SQ=-1;ID=S2508_raw:GT=hom:DP=12:FQ=1:SQ=-1;ID=S2666_raw:GT=hom:DP=4:FQ=1:SQ=-1;ID=S2669_raw:GT=hom:DP=4:FQ=1:SQ=-1;ID=S2166_raw:GT=hom:DP=12:FQ=1:SQ=-1;ID=S1775_raw:GT=hom:DP=8:FQ=1:SQ=-1;ID=S2501_raw:GT=hom:DP=8:FQ=1:SQ=-1;ID=S2500_raw:GT=hom:DP=9:FQ=1:SQ=-1;ID=S2502_raw:GT=hom:DP=10:FQ=1:SQ=-1;ID=S2505_raw:GT=hom:DP=8:FQ=1:SQ=-1;ID=S2176_raw:GT=hom:DP=17:FQ=0.94:SQ=-1;ID=S2506_raw:GT=hom:DP=5:FQ=1:SQ=-1;ID=S2667_raw:GT=hom:DP=4:FQ=1:SQ=-1;
1	1,21E+08	1,21E+08	T	C	intronic	NOTCH2	.	.	rs2493409	.	ID=S2009_raw:GT=hom:DP=12:FQ=1:SQ=-1;ID=S2503_raw:GT=hom:DP=48:FQ=1:SQ=-1;ID=S2507_raw:GT=hom:DP=41:FQ=1:SQ=-1;ID=S2668_raw:GT=hom:DP=61:FQ=1:SQ=-1;ID=S2012_raw:GT=hom:DP=16:FQ=1:SQ=-1;ID=S2010_raw:GT=hom:DP=15:FQ=1:SQ=-1;ID=S2662_raw:GT=hom:DP=13:FQ=1:SQ=-1;ID=S2663_raw:GT=hom:DP=34:FQ=1:SQ=-1;ID=S2011_raw:GT=hom:DP=31:FQ=1:SQ=-1;ID=S2504_raw:GT=hom:DP=54:FQ=1:SQ=-1;ID=S2008_raw:GT=hom:DP=22:FQ=1:SQ=-1;ID=S2509_raw:GT=hom:DP=31:FQ=1:SQ=-1;ID=S2508_raw:GT=hom:DP=46:FQ=1:SQ=-1;ID=S2666_raw:GT=hom:DP=47:FQ=1:SQ=-1;ID=S2669_raw:GT=hom:DP=27:FQ=1:SQ=-1;ID=S2166_raw:GT=hom:DP=39:FQ=1:SQ=-1;ID=S1775_raw:GT=hom:DP=40:FQ=1:SQ=-1;ID=S2501_raw:GT=hom:DP=45:FQ=1:SQ=-1;ID=S2500_raw:GT=hom:DP=40:FQ=1:SQ=-1;ID=S2502_raw:GT=hom:DP=35:FQ=1:SQ=-1;ID=S2505_raw:GT=hom:DP=37:FQ=1:SQ=-1;ID=S2176_raw:GT=hom:DP=57:FQ=1:SQ=-1;ID=S2506_raw:GT=hom:DP=33:FQ=1:SQ=-1;ID=S2667_raw:GT=hom:DP=40:FQ=1:SQ=-1;
19	36334594	36334594	G	A	intronic	NPHS1	.	.	rs436842	.	ID=S2009_raw:GT=hom:DP=10:FQ=1:SQ=-1;ID=S2503_raw:GT=hom:DP=9:FQ=1:SQ=-1;ID=S2507_raw:GT=hom:DP=7:FQ=1:SQ=-1;ID=S2668_raw:GT=hom:DP=8:FQ=1:SQ=-1;ID=S2012_raw:GT=hom:DP=9:FQ=1:SQ=-1;ID=S2010_raw:GT=hom:DP=12:FQ=1:SQ=-1;ID=S2662_raw:GT=hom:DP=24:FQ=1:SQ=-1;ID=S2663_raw:GT=hom:DP=4:FQ=1:SQ=-1;ID=S2011_raw:GT=hom:DP=15:FQ=1:SQ=-1;ID=S2504_raw:GT=hom:DP=9:FQ=1:SQ=-1;ID=S2008_raw:GT=hom:DP=14:FQ=1:SQ=-1;ID=S2509_raw:GT=hom:DP=11:FQ=1:SQ=-1;ID=S2508_raw:GT=hom:DP=11:FQ=1:SQ=-1;ID=S2666_raw:GT=hom:DP=9:FQ=1:SQ=-1;ID=S2669_raw:GT=hom:DP=8:FQ=1:SQ=-1;ID=S2166_raw:GT=hom:DP=16:FQ=1:SQ=-1;ID=S1775_raw:GT=hom:DP=9:FQ=1:SQ=-1;ID=S2501_raw:GT=hom:DP=10:FQ=1:SQ=-1;ID=S2500_raw:GT=hom:DP=20:FQ=1:SQ=-1;ID=S2502_raw:GT=hom:DP=11:FQ=1:SQ=-1;ID=S2505_raw:GT=hom:DP=14:FQ=1:SQ=-1;ID=S2176_raw:GT=hom:DP=23:FQ=1:SQ=-1;ID=S2506_raw:GT=hom:DP=7:FQ=1:SQ=-1;ID=S2667_raw:GT=hom:DP=8:FQ=1:SQ=-1;
1	1,8E+08	1,8E+08	T	C	exonic	NPHS2	synonymous SNV	NPHS2:NM_001297575:exon1:c.102A>G:p.G34G,NPHS2:NM_014625:exon1:c.102A>G:p.G34G	rs1079292	Benign	ID=S2009_raw:GT=hom:DP=131:FQ=1:SQ=-1;ID=S2503_raw:GT=hom:DP=69:FQ=1:SQ=-1;ID=S2507_raw:GT=hom:DP=113:FQ=1:SQ=-1;ID=S2668_raw:GT=hom:DP=79:FQ=1:SQ=-1;ID=S2012_raw:GT=hom:DP=110:FQ=1:SQ=-1;ID=S2010_raw:GT=hom:DP=106:FQ=1:SQ=-1;ID=S2662_raw:GT=hom:DP=159:FQ=1:SQ=-1;ID=S2663_raw:GT=hom:DP=111:FQ=1:SQ=-1;ID=S2011_raw:GT=hom:DP=150:FQ=1:SQ=-1;ID=S2504_raw:GT=hom:DP=82:FQ=1:SQ=-1;ID=S2008_raw:GT=hom:DP=140:FQ=1:SQ=-1;ID=S2509_raw:GT=hom:DP=133:FQ=1:SQ=-1;ID=S2508_raw:GT=hom:DP=109:FQ=1:SQ=-1;ID=S2666_raw:GT=hom:DP=23:FQ=1:SQ=-1;ID=S2669_raw:GT=hom:DP=106:FQ=1:SQ=-1;ID=S2166_raw:GT=hom:DP=108:FQ=1:SQ=-1;ID=S1775_raw:GT=hom:DP=51:FQ=1:SQ=-1;ID=S2501_raw:GT=hom:DP=63:FQ=1:SQ=-1;ID=S2500_raw:GT=hom:DP=71:FQ=1:SQ=-1;ID=S2502_raw:GT=hom:DP=90:FQ=1:SQ=-1;ID=S2505_raw:GT=hom:DP=114:FQ=1:SQ=-1;ID=S2176_raw:GT=hom:DP=119:FQ=1:SQ=-1;ID=S2506_raw:GT=hom:DP=94:FQ=1:SQ=-1;ID=S2667_raw:GT=hom:DP=99:FQ=1:SQ=-1;
1	1,8E+08	1,8E+08	T	C	intergenic	NPHS2;TDRD5	.	dist=11219;dist=4442	rs3926126	.	ID=S2009_raw:GT=hom:DP=31:FQ=1:SQ=-1;ID=S2503_raw:GT=hom:DP=105:FQ=1:SQ=-1;ID=S2507_raw:GT=hom:DP=105:FQ=1:SQ=-1;ID=S2668_raw:GT=hom:DP=93:FQ=1:SQ=-1;ID=S2012_raw:GT=hom:DP=31:FQ=1:SQ=-1;ID=S2010_raw:GT=hom:DP=53:FQ=1:SQ=-1;ID=S2662_raw:GT=hom:DP=123:FQ=1:SQ=-1;ID=S2663_raw:GT=hom:DP=70:FQ=1:SQ=-1;ID=S2011_raw:GT=hom:DP=57:FQ=1:SQ=-1;ID=S2504_raw:GT=hom:DP=112:FQ=1:SQ=-1;ID=S2008_raw:GT=hom:DP=41:FQ=1:SQ=-1;ID=S2509_raw:GT=hom:DP=85:FQ=1:SQ=-1;ID=S2508_raw:GT=hom:DP=94:FQ=1:SQ=-1;ID=S2666_raw:GT=hom:DP=114:FQ=1:SQ=-1;ID=S2669_raw:GT=hom:DP=106:FQ=1:SQ=-1;ID=S2166_raw:GT=hom:DP=91:FQ=1:SQ=-1;ID=S1775_raw:GT=hom:DP=48:FQ=1:SQ=-1;ID=S2501_raw:GT=hom:DP=81:FQ=1:SQ=-1;ID=S2500_raw:GT=hom:DP=106:FQ=1:SQ=-1;ID=S2502_raw:GT=hom:DP=131:FQ=1:SQ=-1;ID=S2505_raw:GT=hom:DP=120:FQ=1:SQ=-1;ID=S2176_raw:GT=hom:DP=121:FQ=1:SQ=-1;ID=S2506_raw:GT=hom:DP=93:FQ=1:SQ=-1;ID=S2667_raw:GT=hom:DP=75:FQ=1:SQ=-1;
4	1954945	1954945	A	G	intronic	NSD2	.	.	rs489550	.	ID=S2009_raw:GT=hom:DP=147:FQ=1:SQ=-1;ID=S2503_raw:GT=hom:DP=139:FQ=1:SQ=-1;ID=S2507_raw:GT=hom:DP=138:FQ=1:SQ=-1;ID=S2668_raw:GT=hom:DP=190:FQ=1:SQ=-1;ID=S2012_raw:GT=hom:DP=170:FQ=1:SQ=-1;ID=S2010_raw:GT=hom:DP=184:FQ=1:SQ=-1;ID=S2662_raw:GT=hom:DP=174:FQ=1:SQ=-1;ID=S2663_raw:GT=hom:DP=149:FQ=1:SQ=-1;ID=S2011_raw:GT=hom:DP=248:FQ=1:SQ=-1;ID=S2504_raw:GT=hom:DP=163:FQ=1:SQ=-1;ID=S2008_raw:GT=hom:DP=179:FQ=1:SQ=-1;ID=S2509_raw:GT=hom:DP=160:FQ=1:SQ=-1;ID=S2508_raw:GT=hom:DP=155:FQ=1:SQ=-1;ID=S2666_raw:GT=hom:DP=187:FQ=1:SQ=-1;ID=S2669_raw:GT=hom:DP=130:FQ=1:SQ=-1;ID=S2166_raw:GT=hom:DP=230:FQ=1:SQ=-1;ID=S1775_raw:GT=hom:DP=127:FQ=1:SQ=-1;ID=S2501_raw:GT=hom:DP=124:FQ=1:SQ=-1;ID=S2500_raw:GT=hom:DP=169:FQ=1:SQ=-1;ID=S2502_raw:GT=hom:DP=166:FQ=1:SQ=-1;ID=S2505_raw:GT=hom:DP=161:FQ=1:SQ=-1;ID=S2176_raw:GT=hom:DP=275:FQ=1:SQ=-1;ID=S2506_raw:GT=hom:DP=137:FQ=1:SQ=-1;ID=S2667_raw:GT=hom:DP=114:FQ=1:SQ=-1;
1	1,57E+08	1,57E+08	A	C	intronic	NTRK1	.	.	rs2644614	.	ID=S2009_raw:GT=hom:DP=11:FQ=1:SQ=-1;ID=S2503_raw:GT=hom:DP=11:FQ=1:SQ=-1;ID=S2507_raw:GT=hom:DP=9:FQ=1:SQ=-1;ID=S2668_raw:GT=hom:DP=7:FQ=1:SQ=-1;ID=S2012_raw:GT=hom:DP=20:FQ=1:SQ=-1;ID=S2010_raw:GT=hom:DP=12:FQ=1:SQ=-1;ID=S2662_raw:GT=hom:DP=9:FQ=1:SQ=-1;ID=S2663_raw:GT=hom:DP=6:FQ=1:SQ=-1;ID=S2011_raw:GT=hom:DP=19:FQ=1:SQ=-1;ID=S2504_raw:GT=hom:DP=7:FQ=1:SQ=-1;ID=S2008_raw:GT=hom:DP=19:FQ=1:SQ=-1;ID=S2509_raw:GT=hom:DP=10:FQ=1:SQ=-1;ID=S2508_raw:GT=hom:DP=20:FQ=1:SQ=-1;ID=S2666_raw:GT=hom:DP=13:FQ=1:SQ=-1;ID=S2669_raw:GT=hom:DP=10:FQ=1:SQ=-1;ID=S2166_raw:GT=hom:DP=19:FQ=1:SQ=-1;ID=S1775_raw:GT=hom:DP=12:FQ=1:SQ=-1;ID=S2501_raw:GT=hom:DP=9:FQ=1:SQ=-1;ID=S2500_raw:GT=hom:DP=10:FQ=1:SQ=-1;ID=S2502_raw:GT=hom:DP=13:FQ=1:SQ=-1;ID=S2505_raw:GT=hom:DP=16:FQ=1:SQ=-1;ID=S2176_raw:GT=hom:DP=24:FQ=1:SQ=-1;ID=S2506_raw:GT=hom:DP=7:FQ=1:SQ=-1;ID=S2667_raw:GT=hom:DP=16:FQ=1:SQ=-1;
14	32315901	32315901	A	G	intronic	NUBPL	.	.	rs8006872	.	ID=S2009_raw:GT=hom:DP=11:FQ=1:SQ=-1;ID=S2503_raw:GT=hom:DP=45:FQ=1:SQ=-1;ID=S2507_raw:GT=hom:DP=33:FQ=1:SQ=-1;ID=S2668_raw:GT=hom:DP=48:FQ=1:SQ=-1;ID=S2012_raw:GT=hom:DP=6:FQ=1:SQ=-1;ID=S2010_raw:GT=hom:DP=13:FQ=1:SQ=-1;ID=S2662_raw:GT=hom:DP=21:FQ=0.9:SQ=-1;ID=S2663_raw:GT=hom:DP=34:FQ=1:SQ=-1;ID=S2011_raw:GT=hom:DP=15:FQ=1:SQ=-1;ID=S2504_raw:GT=hom:DP=78:FQ=1:SQ=-1;ID=S2008_raw:GT=hom:DP=12:FQ=1:SQ=-1;ID=S2509_raw:GT=hom:DP=49:FQ=1:SQ=-1;ID=S2508_raw:GT=hom:DP=52:FQ=1:SQ=-1;ID=S2666_raw:GT=hom:DP=36:FQ=1:SQ=-1;ID=S2669_raw:GT=hom:DP=23:FQ=1:SQ=-1;ID=S2166_raw:GT=hom:DP=73:FQ=1:SQ=-1;ID=S1775_raw:GT=hom:DP=39:FQ=1:SQ=-1;ID=S2501_raw:GT=hom:DP=65:FQ=1:SQ=-1;ID=S2500_raw:GT=hom:DP=49:FQ=1:SQ=-1;ID=S2502_raw:GT=hom:DP=49:FQ=1:SQ=-1;ID=S2505_raw:GT=hom:DP=63:FQ=1:SQ=-1;ID=S2176_raw:GT=hom:DP=81:FQ=1:SQ=-1;ID=S2506_raw:GT=hom:DP=33:FQ=1:SQ=-1;ID=S2667_raw:GT=hom:DP=33:FQ=1:SQ=-1;
17	73221439	73221439	C	T	exonic;splicing	NUP85	synonymous SNV	NUP85:NM_001303276:exon8:c.597C>T:p.P199P,NUP85:NM_001330472:exon8:c.600C>T:p.P200P,NUP85:NM_024844:exon9:c.735C>T:p.P245P	rs2291029	.	ID=S2009_raw:GT=hom:DP=46:FQ=1:SQ=-1;ID=S2503_raw:GT=hom:DP=153:FQ=1:SQ=-1;ID=S2507_raw:GT=hom:DP=160:FQ=1:SQ=-1;ID=S2668_raw:GT=hom:DP=208:FQ=1:SQ=-1;ID=S2012_raw:GT=hom:DP=57:FQ=1:SQ=-1;ID=S2010_raw:GT=hom:DP=44:FQ=1:SQ=-1;ID=S2662_raw:GT=hom:DP=190:FQ=1:SQ=-1;ID=S2663_raw:GT=hom:DP=189:FQ=1:SQ=-1;ID=S2011_raw:GT=hom:DP=59:FQ=1:SQ=-1;ID=S2504_raw:GT=hom:DP=195:FQ=1:SQ=-1;ID=S2008_raw:GT=hom:DP=65:FQ=1:SQ=-1;ID=S2509_raw:GT=hom:DP=132:FQ=1:SQ=-1;ID=S2508_raw:GT=hom:DP=166:FQ=1:SQ=-1;ID=S2666_raw:GT=hom:DP=269:FQ=1:SQ=-1;ID=S2669_raw:GT=hom:DP=156:FQ=0.99:SQ=-1;ID=S2166_raw:GT=hom:DP=158:FQ=1:SQ=-1;ID=S1775_raw:GT=hom:DP=93:FQ=1:SQ=-1;ID=S2501_raw:GT=hom:DP=163:FQ=0.99:SQ=-1;ID=S2500_raw:GT=hom:DP=129:FQ=1:SQ=-1;ID=S2502_raw:GT=hom:DP=174:FQ=0.99:SQ=-1;ID=S2505_raw:GT=hom:DP=187:FQ=1:SQ=-1;ID=S2176_raw:GT=hom:DP=146:FQ=1:SQ=-1;ID=S2506_raw:GT=hom:DP=141:FQ=1:SQ=-1;ID=S2667_raw:GT=hom:DP=180:FQ=1:SQ=-1;
17	73228101	73228101	T	C	intronic	NUP85	.	.	rs899318	.	ID=S2009_raw:GT=hom:DP=24:FQ=1:SQ=-1;ID=S2503_raw:GT=hom:DP=73:FQ=1:SQ=-1;ID=S2507_raw:GT=hom:DP=73:FQ=1:SQ=-1;ID=S2668_raw:GT=hom:DP=49:FQ=1:SQ=-1;ID=S2012_raw:GT=hom:DP=26:FQ=1:SQ=-1;ID=S2010_raw:GT=hom:DP=18:FQ=1:SQ=-1;ID=S2662_raw:GT=hom:DP=70:FQ=1:SQ=-1;ID=S2663_raw:GT=hom:DP=49:FQ=1:SQ=-1;ID=S2011_raw:GT=hom:DP=37:FQ=1:SQ=-1;ID=S2504_raw:GT=hom:DP=75:FQ=1:SQ=-1;ID=S2008_raw:GT=hom:DP=22:FQ=1:SQ=-1;ID=S2509_raw:GT=hom:DP=64:FQ=1:SQ=-1;ID=S2508_raw:GT=hom:DP=62:FQ=1:SQ=-1;ID=S2666_raw:GT=hom:DP=24:FQ=1:SQ=-1;ID=S2669_raw:GT=hom:DP=35:FQ=1:SQ=-1;ID=S2166_raw:GT=hom:DP=58:FQ=1:SQ=-1;ID=S1775_raw:GT=hom:DP=34:FQ=1:SQ=-1;ID=S2501_raw:GT=hom:DP=54:FQ=1:SQ=-1;ID=S2500_raw:GT=hom:DP=56:FQ=1:SQ=-1;ID=S2502_raw:GT=hom:DP=65:FQ=1:SQ=-1;ID=S2505_raw:GT=hom:DP=58:FQ=1:SQ=-1;ID=S2176_raw:GT=hom:DP=76:FQ=1:SQ=-1;ID=S2506_raw:GT=hom:DP=83:FQ=1:SQ=-1;ID=S2667_raw:GT=hom:DP=73:FQ=1:SQ=-1;
11	47834592	47834592	G	A	exonic;splicing	NUP160	synonymous SNV	NUP160:NM_015231:exon15:c.1794C>T:p.D598D	rs7110289	.	ID=S2009_raw:GT=hom:DP=26:FQ=1:SQ=-1;ID=S2503_raw:GT=hom:DP=53:FQ=1:SQ=-1;ID=S2507_raw:GT=hom:DP=42:FQ=1:SQ=-1;ID=S2668_raw:GT=hom:DP=99:FQ=1:SQ=-1;ID=S2012_raw:GT=hom:DP=32:FQ=1:SQ=-1;ID=S2010_raw:GT=hom:DP=34:FQ=1:SQ=-1;ID=S2662_raw:GT=hom:DP=4:FQ=1:SQ=-1;ID=S2663_raw:GT=hom:DP=43:FQ=1:SQ=-1;ID=S2011_raw:GT=hom:DP=41:FQ=1:SQ=-1;ID=S2504_raw:GT=hom:DP=80:FQ=1:SQ=-1;ID=S2008_raw:GT=hom:DP=29:FQ=1:SQ=-1;ID=S2509_raw:GT=hom:DP=40:FQ=1:SQ=-1;ID=S2508_raw:GT=hom:DP=50:FQ=1:SQ=-1;ID=S2666_raw:GT=hom:DP=107:FQ=1:SQ=-1;ID=S2669_raw:GT=hom:DP=30:FQ=1:SQ=-1;ID=S2166_raw:GT=hom:DP=79:FQ=1:SQ=-1;ID=S1775_raw:GT=hom:DP=42:FQ=1:SQ=-1;ID=S2501_raw:GT=hom:DP=59:FQ=1:SQ=-1;ID=S2500_raw:GT=hom:DP=50:FQ=1:SQ=-1;ID=S2502_raw:GT=hom:DP=62:FQ=1:SQ=-1;ID=S2505_raw:GT=hom:DP=48:FQ=1:SQ=-1;ID=S2176_raw:GT=hom:DP=75:FQ=1:SQ=-1;ID=S2506_raw:GT=hom:DP=48:FQ=1:SQ=-1;ID=S2667_raw:GT=hom:DP=44:FQ=1:SQ=-1;
7	1,35E+08	1,35E+08	G	C	exonic	NUP205	nonsynonymous SNV	NUP205:NM_001329434:exon29:c.2992G>C:p.E998Q,NUP205:NM_015135:exon29:c.4066G>C:p.E1356Q	rs7810767	.	ID=S2009_raw:GT=hom:DP=109:FQ=1:SQ=-1;ID=S2503_raw:GT=hom:DP=84:FQ=1:SQ=-1;ID=S2507_raw:GT=hom:DP=70:FQ=1:SQ=-1;ID=S2668_raw:GT=hom:DP=122:FQ=1:SQ=-1;ID=S2012_raw:GT=hom:DP=120:FQ=1:SQ=-1;ID=S2010_raw:GT=hom:DP=142:FQ=1:SQ=-1;ID=S2662_raw:GT=hom:DP=37:FQ=1:SQ=-1;ID=S2663_raw:GT=hom:DP=70:FQ=1:SQ=-1;ID=S2011_raw:GT=hom:DP=195:FQ=1:SQ=-1;ID=S2504_raw:GT=hom:DP=81:FQ=1:SQ=-1;ID=S2008_raw:GT=hom:DP=158:FQ=1:SQ=-1;ID=S2509_raw:GT=hom:DP=85:FQ=1:SQ=-1;ID=S2508_raw:GT=hom:DP=105:FQ=1:SQ=-1;ID=S2666_raw:GT=hom:DP=133:FQ=1:SQ=-1;ID=S2669_raw:GT=hom:DP=75:FQ=1:SQ=-1;ID=S2166_raw:GT=hom:DP=72:FQ=1:SQ=-1;ID=S1775_raw:GT=hom:DP=33:FQ=1:SQ=-1;ID=S2501_raw:GT=hom:DP=72:FQ=0.99:SQ=-1;ID=S2500_raw:GT=hom:DP=94:FQ=1:SQ=-1;ID=S2502_raw:GT=hom:DP=83:FQ=1:SQ=-1;ID=S2505_raw:GT=hom:DP=87:FQ=1:SQ=-1;ID=S2176_raw:GT=hom:DP=70:FQ=1:SQ=-1;ID=S2506_raw:GT=hom:DP=65:FQ=1:SQ=-1;ID=S2667_raw:GT=hom:DP=74:FQ=1:SQ=-1;
1	2,28E+08	2,28E+08	T	A	exonic	OBSCN	nonsynonymous SNV	OBSCN:NM_001098623:exon15:c.4523T>A:p.V1508D,OBSCN:NM_052843:exon15:c.4523T>A:p.V1508D,OBSCN:NM_001271223:exon16:c.4799T>A:p.V1600D	rs7532342	.	ID=S2009_raw:GT=hom:DP=69:FQ=1:SQ=-1;ID=S2503_raw:GT=hom:DP=463:FQ=1:SQ=-1;ID=S2507_raw:GT=hom:DP=421:FQ=1:SQ=-1;ID=S2668_raw:GT=hom:DP=696:FQ=1:SQ=-1;ID=S2012_raw:GT=hom:DP=62:FQ=1:SQ=-1;ID=S2010_raw:GT=hom:DP=102:FQ=1:SQ=-1;ID=S2662_raw:GT=hom:DP=905:FQ=1:SQ=-1;ID=S2663_raw:GT=hom:DP=533:FQ=1:SQ=-1;ID=S2011_raw:GT=hom:DP=89:FQ=1:SQ=-1;ID=S2504_raw:GT=hom:DP=549:FQ=1:SQ=-1;ID=S2008_raw:GT=hom:DP=70:FQ=1:SQ=-1;ID=S2509_raw:GT=hom:DP=480:FQ=1:SQ=-1;ID=S2508_raw:GT=hom:DP=601:FQ=1:SQ=-1;ID=S2666_raw:GT=hom:DP=580:FQ=1:SQ=-1;ID=S2669_raw:GT=hom:DP=529:FQ=1:SQ=-1;ID=S2166_raw:GT=hom:DP=218:FQ=1:SQ=-1;ID=S1775_raw:GT=hom:DP=95:FQ=1:SQ=-1;ID=S2501_raw:GT=hom:DP=432:FQ=1:SQ=-1;ID=S2500_raw:GT=hom:DP=447:FQ=1:SQ=-1;ID=S2502_raw:GT=hom:DP=501:FQ=1:SQ=-1;ID=S2505_raw:GT=hom:DP=499:FQ=1:SQ=-1;ID=S2176_raw:GT=hom:DP=204:FQ=1:SQ=-1;ID=S2506_raw:GT=hom:DP=374:FQ=1:SQ=-1;ID=S2667_raw:GT=hom:DP=526:FQ=1:SQ=-1;
1	2,28E+08	2,28E+08	-	G	intronic	OBSCN	.	.	.	.	ID=S2009_raw:GT=hom:DP=2:FQ=1:SQ=-1;ID=S2503_raw:GT=hom:DP=9:FQ=1:SQ=-1;ID=S2507_raw:GT=hom:DP=14:FQ=1:SQ=-1;ID=S2668_raw:GT=hom:DP=8:FQ=1:SQ=-1;ID=S2012_raw:GT=hom:DP=5:FQ=1:SQ=-1;ID=S2010_raw:GT=hom:DP=3:FQ=1:SQ=-1;ID=S2662_raw:GT=hom:DP=8:FQ=1:SQ=-1;ID=S2663_raw:GT=hom:DP=16:FQ=1:SQ=-1;ID=S2011_raw:GT=hom:DP=4:FQ=1:SQ=-1;ID=S2504_raw:GT=hom:DP=13:FQ=1:SQ=-1;ID=S2008_raw:GT=hom:DP=7:FQ=1:SQ=-1;ID=S2509_raw:GT=hom:DP=12:FQ=1:SQ=-1;ID=S2508_raw:GT=hom:DP=7:FQ=1:SQ=-1;ID=S2666_raw:GT=hom:DP=23:FQ=1:SQ=-1;ID=S2669_raw:GT=hom:DP=9:FQ=1:SQ=-1;ID=S2166_raw:GT=hom:DP=7:FQ=1:SQ=-1;ID=S1775_raw:GT=hom:DP=4:FQ=1:SQ=-1;ID=S2501_raw:GT=hom:DP=8:FQ=1:SQ=-1;ID=S2500_raw:GT=hom:DP=8:FQ=1:SQ=-1;ID=S2502_raw:GT=hom:DP=15:FQ=1:SQ=-1;ID=S2505_raw:GT=hom:DP=10:FQ=1:SQ=-1;ID=S2176_raw:GT=hom:DP=8:FQ=1:SQ=-1;ID=S2506_raw:GT=hom:DP=4:FQ=1:SQ=-1;ID=S2667_raw:GT=hom:DP=12:FQ=1:SQ=-1;
1	2,28E+08	2,28E+08	-	G	intronic	OBSCN	.	.	.	.	ID=S2009_raw:GT=hom:DP=39:FQ=1:SQ=-1;ID=S2503_raw:GT=hom:DP=74:FQ=1:SQ=-1;ID=S2507_raw:GT=hom:DP=85:FQ=1:SQ=-1;ID=S2668_raw:GT=hom:DP=68:FQ=1:SQ=-1;ID=S2012_raw:GT=hom:DP=45:FQ=1:SQ=-1;ID=S2010_raw:GT=hom:DP=34:FQ=1:SQ=-1;ID=S2662_raw:GT=hom:DP=81:FQ=1:SQ=-1;ID=S2663_raw:GT=hom:DP=81:FQ=1:SQ=-1;ID=S2011_raw:GT=hom:DP=46:FQ=1:SQ=-1;ID=S2504_raw:GT=hom:DP=66:FQ=1:SQ=-1;ID=S2008_raw:GT=hom:DP=30:FQ=1:SQ=-1;ID=S2509_raw:GT=hom:DP=54:FQ=1:SQ=-1;ID=S2508_raw:GT=hom:DP=78:FQ=1:SQ=-1;ID=S2666_raw:GT=hom:DP=69:FQ=1:SQ=-1;ID=S2669_raw:GT=hom:DP=56:FQ=1:SQ=-1;ID=S2166_raw:GT=hom:DP=56:FQ=1:SQ=-1;ID=S1775_raw:GT=hom:DP=21:FQ=1:SQ=-1;ID=S2501_raw:GT=hom:DP=59:FQ=1:SQ=-1;ID=S2500_raw:GT=hom:DP=66:FQ=1:SQ=-1;ID=S2502_raw:GT=hom:DP=89:FQ=1:SQ=-1;ID=S2505_raw:GT=hom:DP=55:FQ=1:SQ=-1;ID=S2176_raw:GT=hom:DP=36:FQ=1:SQ=-1;ID=S2506_raw:GT=hom:DP=71:FQ=1:SQ=-1;ID=S2667_raw:GT=hom:DP=47:FQ=1:SQ=-1;
8	1,45E+08	1,45E+08	CC	-	exonic;splicing	OPLAH	frameshift deletion	OPLAH:NM_017570:exon25:c.3497_3498del:p.R1166fs	.	.	ID=S2009_raw:GT=hom:DP=73:FQ=1:SQ=-1;ID=S2503_raw:GT=hom:DP=81:FQ=1:SQ=-1;ID=S2507_raw:GT=hom:DP=85:FQ=1:SQ=-1;ID=S2668_raw:GT=hom:DP=108:FQ=1:SQ=-1;ID=S2012_raw:GT=hom:DP=56:FQ=1:SQ=-1;ID=S2010_raw:GT=hom:DP=54:FQ=1:SQ=-1;ID=S2662_raw:GT=hom:DP=201:FQ=1:SQ=-1;ID=S2663_raw:GT=hom:DP=114:FQ=1:SQ=-1;ID=S2011_raw:GT=hom:DP=72:FQ=1:SQ=-1;ID=S2504_raw:GT=hom:DP=95:FQ=1:SQ=-1;ID=S2008_raw:GT=hom:DP=75:FQ=1:SQ=-1;ID=S2509_raw:GT=hom:DP=112:FQ=1:SQ=-1;ID=S2508_raw:GT=hom:DP=126:FQ=1:SQ=-1;ID=S2666_raw:GT=hom:DP=57:FQ=1:SQ=-1;ID=S2669_raw:GT=hom:DP=101:FQ=1:SQ=-1;ID=S2166_raw:GT=hom:DP=70:FQ=1:SQ=-1;ID=S1775_raw:GT=hom:DP=66:FQ=1:SQ=-1;ID=S2501_raw:GT=hom:DP=83:FQ=1:SQ=-1;ID=S2500_raw:GT=hom:DP=57:FQ=1:SQ=-1;ID=S2502_raw:GT=hom:DP=103:FQ=1:SQ=-1;ID=S2505_raw:GT=hom:DP=65:FQ=1:SQ=-1;ID=S2176_raw:GT=hom:DP=76:FQ=1:SQ=-1;ID=S2506_raw:GT=hom:DP=95:FQ=1:SQ=-1;ID=S2667_raw:GT=hom:DP=109:FQ=1:SQ=-1;
8	1,45E+08	1,45E+08	G	A	exonic;splicing	OPLAH	synonymous SNV	OPLAH:NM_017570:exon22:c.3027C>T:p.A1009A	rs11136253	.	ID=S2009_raw:GT=hom:DP=63:FQ=1:SQ=-1;ID=S2503_raw:GT=hom:DP=147:FQ=1:SQ=-1;ID=S2507_raw:GT=hom:DP=171:FQ=1:SQ=-1;ID=S2668_raw:GT=hom:DP=178:FQ=1:SQ=-1;ID=S2012_raw:GT=hom:DP=55:FQ=1:SQ=-1;ID=S2010_raw:GT=hom:DP=75:FQ=1:SQ=-1;ID=S2662_raw:GT=hom:DP=352:FQ=1:SQ=-1;ID=S2663_raw:GT=hom:DP=174:FQ=1:SQ=-1;ID=S2011_raw:GT=hom:DP=105:FQ=1:SQ=-1;ID=S2504_raw:GT=hom:DP=220:FQ=1:SQ=-1;ID=S2008_raw:GT=hom:DP=83:FQ=1:SQ=-1;ID=S2509_raw:GT=hom:DP=149:FQ=1:SQ=-1;ID=S2508_raw:GT=hom:DP=181:FQ=1:SQ=-1;ID=S2666_raw:GT=hom:DP=191:FQ=1:SQ=-1;ID=S2669_raw:GT=hom:DP=180:FQ=1:SQ=-1;ID=S2166_raw:GT=hom:DP=168:FQ=1:SQ=-1;ID=S1775_raw:GT=hom:DP=87:FQ=1:SQ=-1;ID=S2501_raw:GT=hom:DP=162:FQ=1:SQ=-1;ID=S2500_raw:GT=hom:DP=183:FQ=1:SQ=-1;ID=S2502_raw:GT=hom:DP=147:FQ=1:SQ=-1;ID=S2505_raw:GT=hom:DP=146:FQ=1:SQ=-1;ID=S2176_raw:GT=hom:DP=139:FQ=1:SQ=-1;ID=S2506_raw:GT=hom:DP=170:FQ=1:SQ=-1;ID=S2667_raw:GT=hom:DP=213:FQ=1:SQ=-1;
11	65868310	65868310	T	A	intronic	PACS1	.	.	rs475817	.	ID=S2009_raw:GT=hom:DP=46:FQ=1:SQ=-1;ID=S2503_raw:GT=hom:DP=299:FQ=1:SQ=-1;ID=S2507_raw:GT=hom:DP=261:FQ=1:SQ=-1;ID=S2668_raw:GT=hom:DP=302:FQ=1:SQ=-1;ID=S2012_raw:GT=hom:DP=85:FQ=1:SQ=-1;ID=S2010_raw:GT=hom:DP=94:FQ=1:SQ=-1;ID=S2662_raw:GT=hom:DP=206:FQ=1:SQ=-1;ID=S2663_raw:GT=hom:DP=245:FQ=1:SQ=-1;ID=S2011_raw:GT=hom:DP=119:FQ=1:SQ=-1;ID=S2504_raw:GT=hom:DP=287:FQ=1:SQ=-1;ID=S2008_raw:GT=hom:DP=79:FQ=1:SQ=-1;ID=S2509_raw:GT=hom:DP=281:FQ=1:SQ=-1;ID=S2508_raw:GT=hom:DP=333:FQ=1:SQ=-1;ID=S2666_raw:GT=hom:DP=206:FQ=1:SQ=-1;ID=S2669_raw:GT=hom:DP=234:FQ=0.99:SQ=-1;ID=S2166_raw:GT=hom:DP=236:FQ=1:SQ=-1;ID=S1775_raw:GT=hom:DP=203:FQ=1:SQ=-1;ID=S2501_raw:GT=hom:DP=224:FQ=1:SQ=-1;ID=S2500_raw:GT=hom:DP=246:FQ=1:SQ=-1;ID=S2502_raw:GT=hom:DP=306:FQ=1:SQ=-1;ID=S2505_raw:GT=hom:DP=289:FQ=1:SQ=-1;ID=S2176_raw:GT=hom:DP=244:FQ=1:SQ=-1;ID=S2506_raw:GT=hom:DP=178:FQ=1:SQ=-1;ID=S2667_raw:GT=hom:DP=243:FQ=1:SQ=-1;
11	65973885	65973885	T	G	intronic	PACS1	.	.	rs1627404	.	ID=S2009_raw:GT=hom:DP=6:FQ=1:SQ=-1;ID=S2503_raw:GT=hom:DP=12:FQ=1:SQ=-1;ID=S2507_raw:GT=hom:DP=17:FQ=1:SQ=-1;ID=S2668_raw:GT=hom:DP=7:FQ=1:SQ=-1;ID=S2012_raw:GT=hom:DP=14:FQ=1:SQ=-1;ID=S2010_raw:GT=hom:DP=16:FQ=1:SQ=-1;ID=S2662_raw:GT=hom:DP=6:FQ=1:SQ=-1;ID=S2663_raw:GT=hom:DP=18:FQ=1:SQ=-1;ID=S2011_raw:GT=hom:DP=22:FQ=1:SQ=-1;ID=S2504_raw:GT=hom:DP=17:FQ=1:SQ=-1;ID=S2008_raw:GT=hom:DP=8:FQ=1:SQ=-1;ID=S2509_raw:GT=hom:DP=12:FQ=1:SQ=-1;ID=S2508_raw:GT=hom:DP=19:FQ=1:SQ=-1;ID=S2666_raw:GT=hom:DP=11:FQ=1:SQ=-1;ID=S2669_raw:GT=hom:DP=13:FQ=1:SQ=-1;ID=S2166_raw:GT=hom:DP=6:FQ=1:SQ=-1;ID=S1775_raw:GT=hom:DP=7:FQ=1:SQ=-1;ID=S2501_raw:GT=hom:DP=10:FQ=1:SQ=-1;ID=S2500_raw:GT=hom:DP=18:FQ=1:SQ=-1;ID=S2502_raw:GT=hom:DP=12:FQ=1:SQ=-1;ID=S2505_raw:GT=hom:DP=17:FQ=1:SQ=-1;ID=S2176_raw:GT=hom:DP=10:FQ=1:SQ=-1;ID=S2506_raw:GT=hom:DP=15:FQ=1:SQ=-1;ID=S2667_raw:GT=hom:DP=6:FQ=1:SQ=-1;
11	65983522	65983522	A	G	intronic	PACS1	.	.	rs548858	.	ID=S2009_raw:GT=hom:DP=6:FQ=1:SQ=-1;ID=S2503_raw:GT=hom:DP=17:FQ=1:SQ=-1;ID=S2507_raw:GT=hom:DP=14:FQ=1:SQ=-1;ID=S2668_raw:GT=hom:DP=17:FQ=1:SQ=-1;ID=S2012_raw:GT=hom:DP=9:FQ=1:SQ=-1;ID=S2010_raw:GT=hom:DP=7:FQ=1:SQ=-1;ID=S2662_raw:GT=hom:DP=22:FQ=1:SQ=-1;ID=S2663_raw:GT=hom:DP=10:FQ=1:SQ=-1;ID=S2011_raw:GT=hom:DP=13:FQ=1:SQ=-1;ID=S2504_raw:GT=hom:DP=11:FQ=1:SQ=-1;ID=S2008_raw:GT=hom:DP=11:FQ=1:SQ=-1;ID=S2509_raw:GT=hom:DP=23:FQ=1:SQ=-1;ID=S2508_raw:GT=hom:DP=20:FQ=1:SQ=-1;ID=S2666_raw:GT=hom:DP=17:FQ=1:SQ=-1;ID=S2669_raw:GT=hom:DP=16:FQ=1:SQ=-1;ID=S2166_raw:GT=hom:DP=31:FQ=1:SQ=-1;ID=S1775_raw:GT=hom:DP=8:FQ=1:SQ=-1;ID=S2501_raw:GT=hom:DP=12:FQ=1:SQ=-1;ID=S2500_raw:GT=hom:DP=27:FQ=1:SQ=-1;ID=S2502_raw:GT=hom:DP=28:FQ=1:SQ=-1;ID=S2505_raw:GT=hom:DP=17:FQ=1:SQ=-1;ID=S2176_raw:GT=hom:DP=22:FQ=1:SQ=-1;ID=S2506_raw:GT=hom:DP=16:FQ=1:SQ=-1;ID=S2667_raw:GT=hom:DP=12:FQ=1:SQ=-1;
11	65985000	65985000	A	C	intronic	PACS1	.	.	rs557082	.	ID=S2009_raw:GT=hom:DP=23:FQ=1:SQ=-1;ID=S2503_raw:GT=hom:DP=76:FQ=1:SQ=-1;ID=S2507_raw:GT=hom:DP=66:FQ=1:SQ=-1;ID=S2668_raw:GT=hom:DP=107:FQ=1:SQ=-1;ID=S2012_raw:GT=hom:DP=33:FQ=1:SQ=-1;ID=S2010_raw:GT=hom:DP=25:FQ=1:SQ=-1;ID=S2662_raw:GT=hom:DP=112:FQ=1:SQ=-1;ID=S2663_raw:GT=hom:DP=73:FQ=1:SQ=-1;ID=S2011_raw:GT=hom:DP=44:FQ=1:SQ=-1;ID=S2504_raw:GT=hom:DP=95:FQ=1:SQ=-1;ID=S2008_raw:GT=hom:DP=29:FQ=1:SQ=-1;ID=S2509_raw:GT=hom:DP=75:FQ=1:SQ=-1;ID=S2508_raw:GT=hom:DP=78:FQ=1:SQ=-1;ID=S2666_raw:GT=hom:DP=98:FQ=1:SQ=-1;ID=S2669_raw:GT=hom:DP=94:FQ=1:SQ=-1;ID=S2166_raw:GT=hom:DP=103:FQ=1:SQ=-1;ID=S1775_raw:GT=hom:DP=83:FQ=1:SQ=-1;ID=S2501_raw:GT=hom:DP=74:FQ=1:SQ=-1;ID=S2500_raw:GT=hom:DP=65:FQ=1:SQ=-1;ID=S2502_raw:GT=hom:DP=97:FQ=1:SQ=-1;ID=S2505_raw:GT=hom:DP=83:FQ=1:SQ=-1;ID=S2176_raw:GT=hom:DP=123:FQ=1:SQ=-1;ID=S2506_raw:GT=hom:DP=67:FQ=1:SQ=-1;ID=S2667_raw:GT=hom:DP=105:FQ=1:SQ=-1;
11	65997898	65997898	A	G	intronic	PACS1	.	.	rs561948	.	ID=S2009_raw:GT=hom:DP=15:FQ=1:SQ=-1;ID=S2503_raw:GT=hom:DP=30:FQ=1:SQ=-1;ID=S2507_raw:GT=hom:DP=26:FQ=1:SQ=-1;ID=S2668_raw:GT=hom:DP=46:FQ=1:SQ=-1;ID=S2012_raw:GT=hom:DP=17:FQ=1:SQ=-1;ID=S2010_raw:GT=hom:DP=15:FQ=1:SQ=-1;ID=S2662_raw:GT=hom:DP=28:FQ=1:SQ=-1;ID=S2663_raw:GT=hom:DP=28:FQ=1:SQ=-1;ID=S2011_raw:GT=hom:DP=22:FQ=1:SQ=-1;ID=S2504_raw:GT=hom:DP=35:FQ=1:SQ=-1;ID=S2008_raw:GT=hom:DP=17:FQ=1:SQ=-1;ID=S2509_raw:GT=hom:DP=25:FQ=1:SQ=-1;ID=S2508_raw:GT=hom:DP=43:FQ=1:SQ=-1;ID=S2666_raw:GT=hom:DP=25:FQ=1:SQ=-1;ID=S2669_raw:GT=hom:DP=24:FQ=1:SQ=-1;ID=S2166_raw:GT=hom:DP=20:FQ=1:SQ=-1;ID=S1775_raw:GT=hom:DP=13:FQ=1:SQ=-1;ID=S2501_raw:GT=hom:DP=26:FQ=1:SQ=-1;ID=S2500_raw:GT=hom:DP=27:FQ=1:SQ=-1;ID=S2502_raw:GT=hom:DP=27:FQ=1:SQ=-1;ID=S2505_raw:GT=hom:DP=34:FQ=1:SQ=-1;ID=S2176_raw:GT=hom:DP=21:FQ=1:SQ=-1;ID=S2506_raw:GT=hom:DP=33:FQ=1:SQ=-1;ID=S2667_raw:GT=hom:DP=35:FQ=1:SQ=-1;
11	66010633	66010633	T	C	splicing	PACS1	.	NM_018026:exon24:c.2777-3T>C	rs524268	Benign	ID=S2009_raw:GT=hom:DP=68:FQ=1:SQ=-1;ID=S2503_raw:GT=hom:DP=136:FQ=1:SQ=-1;ID=S2507_raw:GT=hom:DP=132:FQ=1:SQ=-1;ID=S2668_raw:GT=hom:DP=171:FQ=0.99:SQ=-1;ID=S2012_raw:GT=hom:DP=82:FQ=1:SQ=-1;ID=S2010_raw:GT=hom:DP=78:FQ=1:SQ=-1;ID=S2662_raw:GT=hom:DP=194:FQ=1:SQ=-1;ID=S2663_raw:GT=hom:DP=129:FQ=1:SQ=-1;ID=S2011_raw:GT=hom:DP=101:FQ=1:SQ=-1;ID=S2504_raw:GT=hom:DP=128:FQ=1:SQ=-1;ID=S2008_raw:GT=hom:DP=102:FQ=1:SQ=-1;ID=S2509_raw:GT=hom:DP=109:FQ=1:SQ=-1;ID=S2508_raw:GT=hom:DP=132:FQ=1:SQ=-1;ID=S2666_raw:GT=hom:DP=155:FQ=1:SQ=-1;ID=S2669_raw:GT=hom:DP=113:FQ=1:SQ=-1;ID=S2166_raw:GT=hom:DP=95:FQ=1:SQ=-1;ID=S1775_raw:GT=hom:DP=102:FQ=1:SQ=-1;ID=S2501_raw:GT=hom:DP=141:FQ=1:SQ=-1;ID=S2500_raw:GT=hom:DP=109:FQ=1:SQ=-1;ID=S2502_raw:GT=hom:DP=163:FQ=1:SQ=-1;ID=S2505_raw:GT=hom:DP=139:FQ=1:SQ=-1;ID=S2176_raw:GT=hom:DP=105:FQ=1:SQ=-1;ID=S2506_raw:GT=hom:DP=141:FQ=1:SQ=-1;ID=S2667_raw:GT=hom:DP=113:FQ=1:SQ=-1;
1	1,65E+08	1,65E+08	A	G	intronic	PBX1	.	.	rs1538488	.	ID=S2009_raw:GT=hom:DP=36:FQ=1:SQ=-1;ID=S2503_raw:GT=hom:DP=45:FQ=1:SQ=-1;ID=S2507_raw:GT=hom:DP=56:FQ=1:SQ=-1;ID=S2668_raw:GT=hom:DP=28:FQ=1:SQ=-1;ID=S2012_raw:GT=hom:DP=44:FQ=1:SQ=-1;ID=S2010_raw:GT=hom:DP=45:FQ=1:SQ=-1;ID=S2662_raw:GT=hom:DP=28:FQ=1:SQ=-1;ID=S2663_raw:GT=hom:DP=45:FQ=1:SQ=-1;ID=S2011_raw:GT=hom:DP=49:FQ=1:SQ=-1;ID=S2504_raw:GT=hom:DP=48:FQ=1:SQ=-1;ID=S2008_raw:GT=hom:DP=36:FQ=1:SQ=-1;ID=S2509_raw:GT=hom:DP=30:FQ=1:SQ=-1;ID=S2508_raw:GT=hom:DP=39:FQ=1:SQ=-1;ID=S2666_raw:GT=hom:DP=39:FQ=1:SQ=-1;ID=S2669_raw:GT=hom:DP=25:FQ=1:SQ=-1;ID=S2166_raw:GT=hom:DP=58:FQ=1:SQ=-1;ID=S1775_raw:GT=hom:DP=34:FQ=1:SQ=-1;ID=S2501_raw:GT=hom:DP=53:FQ=1:SQ=-1;ID=S2500_raw:GT=hom:DP=40:FQ=1:SQ=-1;ID=S2502_raw:GT=hom:DP=42:FQ=1:SQ=-1;ID=S2505_raw:GT=hom:DP=44:FQ=1:SQ=-1;ID=S2176_raw:GT=hom:DP=48:FQ=1:SQ=-1;ID=S2506_raw:GT=hom:DP=44:FQ=1:SQ=-1;ID=S2667_raw:GT=hom:DP=27:FQ=1:SQ=-1;
14	24567498	24567498	A	C	exonic	PCK2	nonsynonymous SNV	PCK2:NM_001018073:exon3:c.362A>C:p.Q121P,PCK2:NM_004563:exon3:c.362A>C:p.Q121P	rs3021119	.	ID=S2009_raw:GT=hom:DP=153:FQ=1:SQ=-1;ID=S2503_raw:GT=hom:DP=267:FQ=1:SQ=-1;ID=S2507_raw:GT=hom:DP=244:FQ=1:SQ=-1;ID=S2668_raw:GT=hom:DP=306:FQ=1:SQ=-1;ID=S2012_raw:GT=hom:DP=134:FQ=1:SQ=-1;ID=S2010_raw:GT=hom:DP=181:FQ=1:SQ=-1;ID=S2662_raw:GT=hom:DP=439:FQ=1:SQ=-1;ID=S2663_raw:GT=hom:DP=216:FQ=1:SQ=-1;ID=S2011_raw:GT=hom:DP=193:FQ=1:SQ=-1;ID=S2504_raw:GT=hom:DP=228:FQ=1:SQ=-1;ID=S2008_raw:GT=hom:DP=162:FQ=1:SQ=-1;ID=S2509_raw:GT=hom:DP=229:FQ=1:SQ=-1;ID=S2508_raw:GT=hom:DP=256:FQ=1:SQ=-1;ID=S2666_raw:GT=hom:DP=227:FQ=1:SQ=-1;ID=S2669_raw:GT=hom:DP=232:FQ=1:SQ=-1;ID=S2166_raw:GT=hom:DP=264:FQ=1:SQ=-1;ID=S1775_raw:GT=hom:DP=190:FQ=1:SQ=-1;ID=S2501_raw:GT=hom:DP=215:FQ=1:SQ=-1;ID=S2500_raw:GT=hom:DP=227:FQ=1:SQ=-1;ID=S2502_raw:GT=hom:DP=269:FQ=1:SQ=-1;ID=S2505_raw:GT=hom:DP=260:FQ=1:SQ=-1;ID=S2176_raw:GT=hom:DP=208:FQ=1:SQ=-1;ID=S2506_raw:GT=hom:DP=205:FQ=1:SQ=-1;ID=S2667_raw:GT=hom:DP=308:FQ=0.99:SQ=-1;
1	55505732	55505732	A	G	intronic	PCSK9	.	.	rs2495482	Benign/Likely_benign	ID=S2009_raw:GT=hom:DP=5:FQ=1:SQ=-1;ID=S2503_raw:GT=hom:DP=100:FQ=1:SQ=-1;ID=S2507_raw:GT=hom:DP=79:FQ=1:SQ=-1;ID=S2668_raw:GT=hom:DP=110:FQ=1:SQ=-1;ID=S2012_raw:GT=hom:DP=5:FQ=1:SQ=-1;ID=S2010_raw:GT=hom:DP=4:FQ=1:SQ=-1;ID=S2662_raw:GT=hom:DP=152:FQ=1:SQ=-1;ID=S2663_raw:GT=hom:DP=95:FQ=1:SQ=-1;ID=S2011_raw:GT=hom:DP=10:FQ=1:SQ=-1;ID=S2504_raw:GT=hom:DP=72:FQ=1:SQ=-1;ID=S2008_raw:GT=hom:DP=8:FQ=1:SQ=-1;ID=S2509_raw:GT=hom:DP=96:FQ=1:SQ=-1;ID=S2508_raw:GT=hom:DP=96:FQ=1:SQ=-1;ID=S2666_raw:GT=hom:DP=57:FQ=1:SQ=-1;ID=S2669_raw:GT=hom:DP=121:FQ=1:SQ=-1;ID=S2166_raw:GT=hom:DP=64:FQ=1:SQ=-1;ID=S1775_raw:GT=hom:DP=14:FQ=1:SQ=-1;ID=S2501_raw:GT=hom:DP=75:FQ=1:SQ=-1;ID=S2500_raw:GT=hom:DP=66:FQ=1:SQ=-1;ID=S2502_raw:GT=hom:DP=86:FQ=1:SQ=-1;ID=S2505_raw:GT=hom:DP=107:FQ=1:SQ=-1;ID=S2176_raw:GT=hom:DP=50:FQ=1:SQ=-1;ID=S2506_raw:GT=hom:DP=67:FQ=1:SQ=-1;ID=S2667_raw:GT=hom:DP=118:FQ=1:SQ=-1;
1	55516781	55516781	G	T	intronic	PCSK9	.	.	rs599037	.	ID=S2009_raw:GT=hom:DP=95:FQ=1:SQ=-1;ID=S2503_raw:GT=hom:DP=102:FQ=1:SQ=-1;ID=S2507_raw:GT=hom:DP=89:FQ=1:SQ=-1;ID=S2668_raw:GT=hom:DP=122:FQ=1:SQ=-1;ID=S2012_raw:GT=hom:DP=100:FQ=1:SQ=-1;ID=S2010_raw:GT=hom:DP=84:FQ=1:SQ=-1;ID=S2662_raw:GT=hom:DP=138:FQ=1:SQ=-1;ID=S2663_raw:GT=hom:DP=87:FQ=1:SQ=-1;ID=S2011_raw:GT=hom:DP=141:FQ=1:SQ=-1;ID=S2504_raw:GT=hom:DP=100:FQ=1:SQ=-1;ID=S2008_raw:GT=hom:DP=100:FQ=1:SQ=-1;ID=S2509_raw:GT=hom:DP=91:FQ=1:SQ=-1;ID=S2508_raw:GT=hom:DP=95:FQ=1:SQ=-1;ID=S2666_raw:GT=hom:DP=95:FQ=1:SQ=-1;ID=S2669_raw:GT=hom:DP=97:FQ=1:SQ=-1;ID=S2166_raw:GT=hom:DP=110:FQ=1:SQ=-1;ID=S1775_raw:GT=hom:DP=101:FQ=1:SQ=-1;ID=S2501_raw:GT=hom:DP=128:FQ=1:SQ=-1;ID=S2500_raw:GT=hom:DP=103:FQ=1:SQ=-1;ID=S2502_raw:GT=hom:DP=138:FQ=1:SQ=-1;ID=S2505_raw:GT=hom:DP=92:FQ=1:SQ=-1;ID=S2176_raw:GT=hom:DP=119:FQ=1:SQ=-1;ID=S2506_raw:GT=hom:DP=103:FQ=0.99:SQ=-1;ID=S2667_raw:GT=hom:DP=117:FQ=1:SQ=-1;
1	55523033	55523033	A	G	exonic	PCSK9	synonymous SNV	PCSK9:NM_174936:exon7:c.1026A>G:p.Q342Q	rs509504	Conflicting_interpretations_of_pathogenicity	ID=S2009_raw:GT=hom:DP=143:FQ=1:SQ=-1;ID=S2503_raw:GT=hom:DP=120:FQ=1:SQ=-1;ID=S2507_raw:GT=hom:DP=120:FQ=1:SQ=-1;ID=S2668_raw:GT=hom:DP=148:FQ=1:SQ=-1;ID=S2012_raw:GT=hom:DP=127:FQ=1:SQ=-1;ID=S2010_raw:GT=hom:DP=133:FQ=1:SQ=-1;ID=S2662_raw:GT=hom:DP=270:FQ=1:SQ=-1;ID=S2663_raw:GT=hom:DP=123:FQ=1:SQ=-1;ID=S2011_raw:GT=hom:DP=180:FQ=1:SQ=-1;ID=S2504_raw:GT=hom:DP=158:FQ=1:SQ=-1;ID=S2008_raw:GT=hom:DP=159:FQ=1:SQ=-1;ID=S2509_raw:GT=hom:DP=128:FQ=1:SQ=-1;ID=S2508_raw:GT=hom:DP=174:FQ=1:SQ=-1;ID=S2666_raw:GT=hom:DP=115:FQ=1:SQ=-1;ID=S2669_raw:GT=hom:DP=146:FQ=1:SQ=-1;ID=S2166_raw:GT=hom:DP=108:FQ=1:SQ=-1;ID=S1775_raw:GT=hom:DP=107:FQ=1:SQ=-1;ID=S2501_raw:GT=hom:DP=137:FQ=1:SQ=-1;ID=S2500_raw:GT=hom:DP=159:FQ=1:SQ=-1;ID=S2502_raw:GT=hom:DP=160:FQ=1:SQ=-1;ID=S2505_raw:GT=hom:DP=127:FQ=1:SQ=-1;ID=S2176_raw:GT=hom:DP=127:FQ=1:SQ=-1;ID=S2506_raw:GT=hom:DP=159:FQ=1:SQ=-1;ID=S2667_raw:GT=hom:DP=148:FQ=1:SQ=-1;
8	17486282	17486282	T	C	exonic;splicing	PDGFRL	synonymous SNV	PDGFRL:NM_006207:exon5:c.792T>C:p.Y264Y	rs2229084	.	ID=S2009_raw:GT=hom:DP=3:FQ=1:SQ=-1;ID=S2503_raw:GT=hom:DP=59:FQ=1:SQ=-1;ID=S2507_raw:GT=hom:DP=45:FQ=1:SQ=-1;ID=S2668_raw:GT=hom:DP=66:FQ=1:SQ=-1;ID=S2012_raw:GT=hom:DP=6:FQ=1:SQ=-1;ID=S2010_raw:GT=hom:DP=2:FQ=1:SQ=-1;ID=S2662_raw:GT=hom:DP=61:FQ=1:SQ=-1;ID=S2663_raw:GT=hom:DP=81:FQ=1:SQ=-1;ID=S2011_raw:GT=hom:DP=14:FQ=1:SQ=-1;ID=S2504_raw:GT=hom:DP=63:FQ=1:SQ=-1;ID=S2008_raw:GT=hom:DP=5:FQ=1:SQ=-1;ID=S2509_raw:GT=hom:DP=64:FQ=1:SQ=-1;ID=S2508_raw:GT=hom:DP=64:FQ=1:SQ=-1;ID=S2666_raw:GT=hom:DP=78:FQ=1:SQ=-1;ID=S2669_raw:GT=hom:DP=56:FQ=1:SQ=-1;ID=S2166_raw:GT=hom:DP=80:FQ=1:SQ=-1;ID=S1775_raw:GT=hom:DP=39:FQ=1:SQ=-1;ID=S2501_raw:GT=hom:DP=62:FQ=1:SQ=-1;ID=S2500_raw:GT=hom:DP=68:FQ=1:SQ=-1;ID=S2502_raw:GT=hom:DP=53:FQ=1:SQ=-1;ID=S2505_raw:GT=hom:DP=63:FQ=1:SQ=-1;ID=S2176_raw:GT=hom:DP=84:FQ=1:SQ=-1;ID=S2506_raw:GT=hom:DP=51:FQ=1:SQ=-1;ID=S2667_raw:GT=hom:DP=65:FQ=1:SQ=-1;
8	77895865	77895865	A	G	exonic	PEX2	nonsynonymous SNV	PEX2:NM_001079867:exon3:c.550T>C:p.C184R,PEX2:NM_001172087:exon3:c.550T>C:p.C184R,PEX2:NM_000318:exon4:c.550T>C:p.C184R,PEX2:NM_001172086:exon5:c.550T>C:p.C184R	rs10087163	Benign	ID=S2009_raw:GT=hom:DP=124:FQ=1:SQ=-1;ID=S2503_raw:GT=hom:DP=110:FQ=1:SQ=-1;ID=S2507_raw:GT=hom:DP=105:FQ=1:SQ=-1;ID=S2668_raw:GT=hom:DP=149:FQ=1:SQ=-1;ID=S2012_raw:GT=hom:DP=135:FQ=1:SQ=-1;ID=S2010_raw:GT=hom:DP=136:FQ=1:SQ=-1;ID=S2662_raw:GT=hom:DP=15:FQ=1:SQ=-1;ID=S2663_raw:GT=hom:DP=118:FQ=1:SQ=-1;ID=S2011_raw:GT=hom:DP=215:FQ=1:SQ=-1;ID=S2504_raw:GT=hom:DP=131:FQ=1:SQ=-1;ID=S2008_raw:GT=hom:DP=147:FQ=1:SQ=-1;ID=S2509_raw:GT=hom:DP=118:FQ=1:SQ=-1;ID=S2508_raw:GT=hom:DP=120:FQ=1:SQ=-1;ID=S2666_raw:GT=hom:DP=163:FQ=1:SQ=-1;ID=S2669_raw:GT=hom:DP=81:FQ=1:SQ=-1;ID=S2166_raw:GT=hom:DP=256:FQ=1:SQ=-1;ID=S1775_raw:GT=hom:DP=203:FQ=1:SQ=-1;ID=S2501_raw:GT=hom:DP=121:FQ=1:SQ=-1;ID=S2500_raw:GT=hom:DP=117:FQ=1:SQ=-1;ID=S2502_raw:GT=hom:DP=117:FQ=1:SQ=-1;ID=S2505_raw:GT=hom:DP=93:FQ=1:SQ=-1;ID=S2176_raw:GT=hom:DP=235:FQ=1:SQ=-1;ID=S2506_raw:GT=hom:DP=70:FQ=1:SQ=-1;ID=S2667_raw:GT=hom:DP=134:FQ=1:SQ=-1;
1	10596388	10596388	A	G	intronic	PEX14	.	.	rs2480779	.	ID=S2009_raw:GT=hom:DP=9:FQ=1:SQ=-1;ID=S2503_raw:GT=hom:DP=84:FQ=1:SQ=-1;ID=S2507_raw:GT=hom:DP=72:FQ=1:SQ=-1;ID=S2668_raw:GT=hom:DP=46:FQ=1:SQ=-1;ID=S2012_raw:GT=hom:DP=14:FQ=1:SQ=-1;ID=S2010_raw:GT=hom:DP=16:FQ=1:SQ=-1;ID=S2662_raw:GT=hom:DP=77:FQ=1:SQ=-1;ID=S2663_raw:GT=hom:DP=74:FQ=1:SQ=-1;ID=S2011_raw:GT=hom:DP=26:FQ=1:SQ=-1;ID=S2504_raw:GT=hom:DP=86:FQ=1:SQ=-1;ID=S2008_raw:GT=hom:DP=22:FQ=1:SQ=-1;ID=S2509_raw:GT=hom:DP=44:FQ=1:SQ=-1;ID=S2508_raw:GT=hom:DP=64:FQ=1:SQ=-1;ID=S2666_raw:GT=hom:DP=30:FQ=1:SQ=-1;ID=S2669_raw:GT=hom:DP=55:FQ=0.98:SQ=-1;ID=S2166_raw:GT=hom:DP=48:FQ=1:SQ=-1;ID=S1775_raw:GT=hom:DP=37:FQ=1:SQ=-1;ID=S2501_raw:GT=hom:DP=66:FQ=1:SQ=-1;ID=S2500_raw:GT=hom:DP=70:FQ=1:SQ=-1;ID=S2502_raw:GT=hom:DP=84:FQ=1:SQ=-1;ID=S2505_raw:GT=hom:DP=70:FQ=1:SQ=-1;ID=S2176_raw:GT=hom:DP=60:FQ=1:SQ=-1;ID=S2506_raw:GT=hom:DP=49:FQ=1:SQ=-1;ID=S2667_raw:GT=hom:DP=54:FQ=1:SQ=-1;
1	10678655	10678655	T	C	intronic	PEX14	.	.	rs284236	.	ID=S2009_raw:GT=hom:DP=13:FQ=1:SQ=-1;ID=S2503_raw:GT=hom:DP=16:FQ=1:SQ=-1;ID=S2507_raw:GT=hom:DP=16:FQ=1:SQ=-1;ID=S2668_raw:GT=hom:DP=19:FQ=1:SQ=-1;ID=S2012_raw:GT=hom:DP=9:FQ=1:SQ=-1;ID=S2010_raw:GT=hom:DP=21:FQ=1:SQ=-1;ID=S2662_raw:GT=hom:DP=20:FQ=1:SQ=-1;ID=S2663_raw:GT=hom:DP=7:FQ=1:SQ=-1;ID=S2011_raw:GT=hom:DP=24:FQ=1:SQ=-1;ID=S2504_raw:GT=hom:DP=19:FQ=1:SQ=-1;ID=S2008_raw:GT=hom:DP=19:FQ=1:SQ=-1;ID=S2509_raw:GT=hom:DP=14:FQ=1:SQ=-1;ID=S2508_raw:GT=hom:DP=26:FQ=1:SQ=-1;ID=S2666_raw:GT=hom:DP=7:FQ=1:SQ=-1;ID=S2669_raw:GT=hom:DP=13:FQ=1:SQ=-1;ID=S2166_raw:GT=hom:DP=31:FQ=1:SQ=-1;ID=S1775_raw:GT=hom:DP=19:FQ=1:SQ=-1;ID=S2501_raw:GT=hom:DP=10:FQ=1:SQ=-1;ID=S2500_raw:GT=hom:DP=21:FQ=1:SQ=-1;ID=S2502_raw:GT=hom:DP=30:FQ=1:SQ=-1;ID=S2505_raw:GT=hom:DP=16:FQ=1:SQ=-1;ID=S2176_raw:GT=hom:DP=33:FQ=1:SQ=-1;ID=S2506_raw:GT=hom:DP=15:FQ=1:SQ=-1;ID=S2667_raw:GT=hom:DP=16:FQ=1:SQ=-1;
11	45937267	45937267	C	T	exonic	PEX16	nonsynonymous SNV	PEX16:NM_004813:exon4:c.346G>A:p.V116I,PEX16:NM_057174:exon4:c.346G>A:p.V116I	rs10742772	.	ID=S2009_raw:GT=hom:DP=210:FQ=1:SQ=-1;ID=S2503_raw:GT=hom:DP=279:FQ=1:SQ=-1;ID=S2507_raw:GT=hom:DP=318:FQ=1:SQ=-1;ID=S2668_raw:GT=hom:DP=292:FQ=1:SQ=-1;ID=S2012_raw:GT=hom:DP=210:FQ=1:SQ=-1;ID=S2010_raw:GT=hom:DP=221:FQ=1:SQ=-1;ID=S2662_raw:GT=hom:DP=506:FQ=1:SQ=-1;ID=S2663_raw:GT=hom:DP=297:FQ=1:SQ=-1;ID=S2011_raw:GT=hom:DP=344:FQ=1:SQ=-1;ID=S2504_raw:GT=hom:DP=372:FQ=1:SQ=-1;ID=S2008_raw:GT=hom:DP=301:FQ=1:SQ=-1;ID=S2509_raw:GT=hom:DP=361:FQ=1:SQ=-1;ID=S2508_raw:GT=hom:DP=388:FQ=1:SQ=-1;ID=S2666_raw:GT=hom:DP=265:FQ=1:SQ=-1;ID=S2669_raw:GT=hom:DP=323:FQ=1:SQ=-1;ID=S2166_raw:GT=hom:DP=307:FQ=1:SQ=-1;ID=S1775_raw:GT=hom:DP=310:FQ=1:SQ=-1;ID=S2501_raw:GT=hom:DP=346:FQ=1:SQ=-1;ID=S2500_raw:GT=hom:DP=313:FQ=1:SQ=-1;ID=S2502_raw:GT=hom:DP=349:FQ=1:SQ=-1;ID=S2505_raw:GT=hom:DP=274:FQ=1:SQ=-1;ID=S2176_raw:GT=hom:DP=323:FQ=1:SQ=-1;ID=S2506_raw:GT=hom:DP=265:FQ=1:SQ=-1;ID=S2667_raw:GT=hom:DP=302:FQ=1:SQ=-1;
16	30762640	30762640	C	T	intronic	PHKG2	.	.	rs4889504	Benign	ID=S2009_raw:GT=hom:DP=63:FQ=1:SQ=-1;ID=S2503_raw:GT=hom:DP=63:FQ=1:SQ=-1;ID=S2507_raw:GT=hom:DP=67:FQ=1:SQ=-1;ID=S2668_raw:GT=hom:DP=114:FQ=1:SQ=-1;ID=S2012_raw:GT=hom:DP=50:FQ=1:SQ=-1;ID=S2010_raw:GT=hom:DP=68:FQ=1:SQ=-1;ID=S2662_raw:GT=hom:DP=109:FQ=1:SQ=-1;ID=S2663_raw:GT=hom:DP=58:FQ=1:SQ=-1;ID=S2011_raw:GT=hom:DP=63:FQ=1:SQ=-1;ID=S2504_raw:GT=hom:DP=91:FQ=1:SQ=-1;ID=S2008_raw:GT=hom:DP=50:FQ=1:SQ=-1;ID=S2509_raw:GT=hom:DP=67:FQ=1:SQ=-1;ID=S2508_raw:GT=hom:DP=71:FQ=1:SQ=-1;ID=S2666_raw:GT=hom:DP=86:FQ=1:SQ=-1;ID=S2669_raw:GT=hom:DP=60:FQ=1:SQ=-1;ID=S2166_raw:GT=hom:DP=81:FQ=1:SQ=-1;ID=S1775_raw:GT=hom:DP=49:FQ=1:SQ=-1;ID=S2501_raw:GT=hom:DP=57:FQ=1:SQ=-1;ID=S2500_raw:GT=hom:DP=68:FQ=1:SQ=-1;ID=S2502_raw:GT=hom:DP=86:FQ=1:SQ=-1;ID=S2505_raw:GT=hom:DP=68:FQ=1:SQ=-1;ID=S2176_raw:GT=hom:DP=50:FQ=1:SQ=-1;ID=S2506_raw:GT=hom:DP=80:FQ=1:SQ=-1;ID=S2667_raw:GT=hom:DP=84:FQ=1:SQ=-1;
16	30768503	30768509	TGGCCTC	-	UTR3	PHKG2	.	NM_000294:c.*85_*91delTGGCCTC	.	.	ID=S2009_raw:GT=hom:DP=5:FQ=1:SQ=-1;ID=S2503_raw:GT=hom:DP=60:FQ=1:SQ=-1;ID=S2507_raw:GT=hom:DP=54:FQ=1:SQ=-1;ID=S2668_raw:GT=hom:DP=64:FQ=1:SQ=-1;ID=S2012_raw:GT=hom:DP=9:FQ=1:SQ=-1;ID=S2010_raw:GT=hom:DP=12:FQ=1:SQ=-1;ID=S2662_raw:GT=hom:DP=93:FQ=1:SQ=-1;ID=S2663_raw:GT=hom:DP=60:FQ=1:SQ=-1;ID=S2011_raw:GT=hom:DP=3:FQ=1:SQ=-1;ID=S2504_raw:GT=hom:DP=66:FQ=1:SQ=-1;ID=S2008_raw:GT=hom:DP=7:FQ=1:SQ=-1;ID=S2509_raw:GT=hom:DP=51:FQ=1:SQ=-1;ID=S2508_raw:GT=hom:DP=50:FQ=1:SQ=-1;ID=S2666_raw:GT=hom:DP=55:FQ=1:SQ=-1;ID=S2669_raw:GT=hom:DP=59:FQ=1:SQ=-1;ID=S2166_raw:GT=hom:DP=23:FQ=1:SQ=-1;ID=S1775_raw:GT=hom:DP=22:FQ=1:SQ=-1;ID=S2501_raw:GT=hom:DP=57:FQ=1:SQ=-1;ID=S2500_raw:GT=hom:DP=50:FQ=1:SQ=-1;ID=S2502_raw:GT=hom:DP=70:FQ=1:SQ=-1;ID=S2505_raw:GT=hom:DP=44:FQ=1:SQ=-1;ID=S2176_raw:GT=hom:DP=23:FQ=1:SQ=-1;ID=S2506_raw:GT=hom:DP=49:FQ=1:SQ=-1;ID=S2667_raw:GT=hom:DP=55:FQ=1:SQ=-1;
16	30770267	30770267	C	G	UTR3	PHKG2	.	NM_000294:c.*1849C>G	rs7188077	.	ID=S2009_raw:GT=hom:DP=6:FQ=1:SQ=-1;ID=S2503_raw:GT=hom:DP=42:FQ=1:SQ=-1;ID=S2507_raw:GT=hom:DP=55:FQ=1:SQ=-1;ID=S2668_raw:GT=hom:DP=75:FQ=1:SQ=-1;ID=S2012_raw:GT=hom:DP=10:FQ=1:SQ=-1;ID=S2010_raw:GT=hom:DP=12:FQ=1:SQ=-1;ID=S2662_raw:GT=hom:DP=65:FQ=1:SQ=-1;ID=S2663_raw:GT=hom:DP=36:FQ=1:SQ=-1;ID=S2011_raw:GT=hom:DP=22:FQ=1:SQ=-1;ID=S2504_raw:GT=hom:DP=41:FQ=1:SQ=-1;ID=S2008_raw:GT=hom:DP=18:FQ=1:SQ=-1;ID=S2509_raw:GT=hom:DP=53:FQ=1:SQ=-1;ID=S2508_raw:GT=hom:DP=45:FQ=1:SQ=-1;ID=S2666_raw:GT=hom:DP=54:FQ=1:SQ=-1;ID=S2669_raw:GT=hom:DP=32:FQ=1:SQ=-1;ID=S2166_raw:GT=hom:DP=32:FQ=1:SQ=-1;ID=S1775_raw:GT=hom:DP=22:FQ=1:SQ=-1;ID=S2501_raw:GT=hom:DP=57:FQ=1:SQ=-1;ID=S2500_raw:GT=hom:DP=60:FQ=1:SQ=-1;ID=S2502_raw:GT=hom:DP=47:FQ=0.98:SQ=-1;ID=S2505_raw:GT=hom:DP=51:FQ=1:SQ=-1;ID=S2176_raw:GT=hom:DP=26:FQ=1:SQ=-1;ID=S2506_raw:GT=hom:DP=45:FQ=1:SQ=-1;ID=S2667_raw:GT=hom:DP=47:FQ=1:SQ=-1;
16	30772040	30772040	A	G	UTR3	PHKG2	.	NM_001172432:c.*559A>G;NM_000294:c.*3622A>G	rs4889505	.	ID=S2009_raw:GT=hom:DP=55:FQ=1:SQ=-1;ID=S2503_raw:GT=hom:DP=110:FQ=1:SQ=-1;ID=S2507_raw:GT=hom:DP=86:FQ=1:SQ=-1;ID=S2668_raw:GT=hom:DP=156:FQ=1:SQ=-1;ID=S2012_raw:GT=hom:DP=66:FQ=1:SQ=-1;ID=S2010_raw:GT=hom:DP=66:FQ=1:SQ=-1;ID=S2662_raw:GT=hom:DP=197:FQ=1:SQ=-1;ID=S2663_raw:GT=hom:DP=106:FQ=1:SQ=-1;ID=S2011_raw:GT=hom:DP=87:FQ=1:SQ=-1;ID=S2504_raw:GT=hom:DP=112:FQ=1:SQ=-1;ID=S2008_raw:GT=hom:DP=62:FQ=1:SQ=-1;ID=S2509_raw:GT=hom:DP=102:FQ=1:SQ=-1;ID=S2508_raw:GT=hom:DP=118:FQ=1:SQ=-1;ID=S2666_raw:GT=hom:DP=86:FQ=1:SQ=-1;ID=S2669_raw:GT=hom:DP=114:FQ=1:SQ=-1;ID=S2166_raw:GT=hom:DP=82:FQ=1:SQ=-1;ID=S1775_raw:GT=hom:DP=36:FQ=1:SQ=-1;ID=S2501_raw:GT=hom:DP=106:FQ=1:SQ=-1;ID=S2500_raw:GT=hom:DP=110:FQ=1:SQ=-1;ID=S2502_raw:GT=hom:DP=138:FQ=1:SQ=-1;ID=S2505_raw:GT=hom:DP=87:FQ=1:SQ=-1;ID=S2176_raw:GT=hom:DP=89:FQ=1:SQ=-1;ID=S2506_raw:GT=hom:DP=80:FQ=1:SQ=-1;ID=S2667_raw:GT=hom:DP=107:FQ=1:SQ=-1;
10	13334058	13334058	A	G	intronic	PHYH	.	.	rs1408364	.	ID=S2009_raw:GT=hom:DP=2:FQ=1:SQ=-1;ID=S2503_raw:GT=hom:DP=3:FQ=1:SQ=-1;ID=S2507_raw:GT=hom:DP=8:FQ=1:SQ=-1;ID=S2668_raw:GT=hom:DP=4:FQ=1:SQ=-1;ID=S2012_raw:GT=hom:DP=2:FQ=1:SQ=-1;ID=S2010_raw:GT=hom:DP=1:FQ=1:SQ=-1;ID=S2662_raw:GT=hom:DP=1:FQ=1:SQ=-1;ID=S2663_raw:GT=hom:DP=12:FQ=1:SQ=-1;ID=S2011_raw:GT=hom:DP=1:FQ=1:SQ=-1;ID=S2504_raw:GT=hom:DP=5:FQ=1:SQ=-1;ID=S2008_raw:GT=hom:DP=2:FQ=1:SQ=-1;ID=S2509_raw:GT=hom:DP=8:FQ=1:SQ=-1;ID=S2508_raw:GT=hom:DP=2:FQ=1:SQ=-1;ID=S2666_raw:GT=hom:DP=2:FQ=1:SQ=-1;ID=S2669_raw:GT=hom:DP=2:FQ=1:SQ=-1;ID=S2166_raw:GT=hom:DP=9:FQ=1:SQ=-1;ID=S1775_raw:GT=hom:DP=2:FQ=1:SQ=-1;ID=S2501_raw:GT=hom:DP=4:FQ=1:SQ=-1;ID=S2500_raw:GT=hom:DP=8:FQ=1:SQ=-1;ID=S2502_raw:GT=hom:DP=3:FQ=1:SQ=-1;ID=S2505_raw:GT=hom:DP=5:FQ=1:SQ=-1;ID=S2176_raw:GT=hom:DP=1:FQ=1:SQ=-1;ID=S2506_raw:GT=hom:DP=4:FQ=1:SQ=-1;ID=S2667_raw:GT=hom:DP=7:FQ=1:SQ=-1;
10	13341836	13341836	G	C	intronic	PHYH	.	.	rs511959	.	ID=S2009_raw:GT=hom:DP=5:FQ=1:SQ=-1;ID=S2503_raw:GT=hom:DP=11:FQ=1:SQ=-1;ID=S2507_raw:GT=hom:DP=7:FQ=1:SQ=-1;ID=S2668_raw:GT=hom:DP=2:FQ=1:SQ=-1;ID=S2012_raw:GT=hom:DP=8:FQ=1:SQ=-1;ID=S2010_raw:GT=hom:DP=7:FQ=1:SQ=-1;ID=S2662_raw:GT=hom:DP=14:FQ=1:SQ=-1;ID=S2663_raw:GT=hom:DP=9:FQ=1:SQ=-1;ID=S2011_raw:GT=hom:DP=9:FQ=1:SQ=-1;ID=S2504_raw:GT=hom:DP=5:FQ=1:SQ=-1;ID=S2008_raw:GT=hom:DP=4:FQ=1:SQ=-1;ID=S2509_raw:GT=hom:DP=13:FQ=1:SQ=-1;ID=S2508_raw:GT=hom:DP=5:FQ=1:SQ=-1;ID=S2666_raw:GT=hom:DP=4:FQ=1:SQ=-1;ID=S2669_raw:GT=hom:DP=6:FQ=1:SQ=-1;ID=S2166_raw:GT=hom:DP=9:FQ=1:SQ=-1;ID=S1775_raw:GT=hom:DP=3:FQ=1:SQ=-1;ID=S2501_raw:GT=hom:DP=4:FQ=1:SQ=-1;ID=S2500_raw:GT=hom:DP=7:FQ=1:SQ=-1;ID=S2502_raw:GT=hom:DP=3:FQ=1:SQ=-1;ID=S2505_raw:GT=hom:DP=7:FQ=1:SQ=-1;ID=S2176_raw:GT=hom:DP=5:FQ=1:SQ=-1;ID=S2506_raw:GT=hom:DP=6:FQ=1:SQ=-1;ID=S2667_raw:GT=hom:DP=5:FQ=1:SQ=-1;
10	13341900	13341900	A	G	intronic	PHYH	.	.	rs512109	.	ID=S2009_raw:GT=hom:DP=17:FQ=1:SQ=-1;ID=S2503_raw:GT=hom:DP=18:FQ=1:SQ=-1;ID=S2507_raw:GT=hom:DP=25:FQ=1:SQ=-1;ID=S2668_raw:GT=hom:DP=8:FQ=1:SQ=-1;ID=S2012_raw:GT=hom:DP=14:FQ=1:SQ=-1;ID=S2010_raw:GT=hom:DP=21:FQ=1:SQ=-1;ID=S2662_raw:GT=hom:DP=50:FQ=1:SQ=-1;ID=S2663_raw:GT=hom:DP=28:FQ=1:SQ=-1;ID=S2011_raw:GT=hom:DP=26:FQ=1:SQ=-1;ID=S2504_raw:GT=hom:DP=14:FQ=1:SQ=-1;ID=S2008_raw:GT=hom:DP=16:FQ=1:SQ=-1;ID=S2509_raw:GT=hom:DP=28:FQ=1:SQ=-1;ID=S2508_raw:GT=hom:DP=17:FQ=1:SQ=-1;ID=S2666_raw:GT=hom:DP=5:FQ=1:SQ=-1;ID=S2669_raw:GT=hom:DP=21:FQ=1:SQ=-1;ID=S2166_raw:GT=hom:DP=19:FQ=1:SQ=-1;ID=S1775_raw:GT=hom:DP=10:FQ=1:SQ=-1;ID=S2501_raw:GT=hom:DP=9:FQ=1:SQ=-1;ID=S2500_raw:GT=hom:DP=13:FQ=1:SQ=-1;ID=S2502_raw:GT=hom:DP=13:FQ=1:SQ=-1;ID=S2505_raw:GT=hom:DP=21:FQ=1:SQ=-1;ID=S2176_raw:GT=hom:DP=13:FQ=1:SQ=-1;ID=S2506_raw:GT=hom:DP=16:FQ=1:SQ=-1;ID=S2667_raw:GT=hom:DP=23:FQ=1:SQ=-1;
10	13344221	13344221	A	C	intergenic	PHYH;SEPHS1	.	dist=2091;dist=15208	rs825613	.	ID=S2009_raw:GT=hom:DP=11:FQ=1:SQ=-1;ID=S2503_raw:GT=hom:DP=21:FQ=1:SQ=-1;ID=S2507_raw:GT=hom:DP=12:FQ=1:SQ=-1;ID=S2668_raw:GT=hom:DP=16:FQ=1:SQ=-1;ID=S2012_raw:GT=hom:DP=18:FQ=1:SQ=-1;ID=S2010_raw:GT=hom:DP=17:FQ=1:SQ=-1;ID=S2662_raw:GT=hom:DP=20:FQ=1:SQ=-1;ID=S2663_raw:GT=hom:DP=16:FQ=1:SQ=-1;ID=S2011_raw:GT=hom:DP=20:FQ=1:SQ=-1;ID=S2504_raw:GT=hom:DP=21:FQ=1:SQ=-1;ID=S2008_raw:GT=hom:DP=14:FQ=1:SQ=-1;ID=S2509_raw:GT=hom:DP=9:FQ=1:SQ=-1;ID=S2508_raw:GT=hom:DP=19:FQ=1:SQ=-1;ID=S2666_raw:GT=hom:DP=12:FQ=1:SQ=-1;ID=S2669_raw:GT=hom:DP=9:FQ=1:SQ=-1;ID=S2166_raw:GT=hom:DP=6:FQ=1:SQ=-1;ID=S1775_raw:GT=hom:DP=2:FQ=1:SQ=-1;ID=S2501_raw:GT=hom:DP=18:FQ=1:SQ=-1;ID=S2500_raw:GT=hom:DP=17:FQ=1:SQ=-1;ID=S2502_raw:GT=hom:DP=12:FQ=1:SQ=-1;ID=S2505_raw:GT=hom:DP=17:FQ=1:SQ=-1;ID=S2176_raw:GT=hom:DP=7:FQ=1:SQ=-1;ID=S2506_raw:GT=hom:DP=15:FQ=1:SQ=-1;ID=S2667_raw:GT=hom:DP=9:FQ=1:SQ=-1;
22	21081964	21081964	C	T	intronic	PI4KA	.	.	rs4820583	.	ID=S2009_raw:GT=hom:DP=49:FQ=1:SQ=-1;ID=S2503_raw:GT=hom:DP=94:FQ=1:SQ=-1;ID=S2507_raw:GT=hom:DP=106:FQ=1:SQ=-1;ID=S2668_raw:GT=hom:DP=104:FQ=1:SQ=-1;ID=S2012_raw:GT=hom:DP=63:FQ=1:SQ=-1;ID=S2010_raw:GT=hom:DP=66:FQ=1:SQ=-1;ID=S2662_raw:GT=hom:DP=110:FQ=1:SQ=-1;ID=S2663_raw:GT=hom:DP=89:FQ=1:SQ=-1;ID=S2011_raw:GT=hom:DP=70:FQ=1:SQ=-1;ID=S2504_raw:GT=hom:DP=103:FQ=1:SQ=-1;ID=S2008_raw:GT=hom:DP=64:FQ=1:SQ=-1;ID=S2509_raw:GT=hom:DP=97:FQ=1:SQ=-1;ID=S2508_raw:GT=hom:DP=93:FQ=1:SQ=-1;ID=S2666_raw:GT=hom:DP=122:FQ=1:SQ=-1;ID=S2669_raw:GT=hom:DP=75:FQ=1:SQ=-1;ID=S2166_raw:GT=hom:DP=61:FQ=1:SQ=-1;ID=S1775_raw:GT=hom:DP=34:FQ=1:SQ=-1;ID=S2501_raw:GT=hom:DP=81:FQ=1:SQ=-1;ID=S2500_raw:GT=hom:DP=94:FQ=1:SQ=-1;ID=S2502_raw:GT=hom:DP=110:FQ=0.98:SQ=-1;ID=S2505_raw:GT=hom:DP=90:FQ=1:SQ=-1;ID=S2176_raw:GT=hom:DP=76:FQ=1:SQ=-1;ID=S2506_raw:GT=hom:DP=91:FQ=1:SQ=-1;ID=S2667_raw:GT=hom:DP=107:FQ=1:SQ=-1;
18	10763218	10763218	T	C	intronic	PIEZO2	.	.	rs2865131	.	ID=S2009_raw:GT=hom:DP=5:FQ=1:SQ=-1;ID=S2503_raw:GT=hom:DP=31:FQ=1:SQ=-1;ID=S2507_raw:GT=hom:DP=41:FQ=1:SQ=-1;ID=S2668_raw:GT=hom:DP=70:FQ=1:SQ=-1;ID=S2012_raw:GT=hom:DP=4:FQ=1:SQ=-1;ID=S2010_raw:GT=hom:DP=6:FQ=1:SQ=-1;ID=S2662_raw:GT=hom:DP=38:FQ=1:SQ=-1;ID=S2663_raw:GT=hom:DP=36:FQ=1:SQ=-1;ID=S2011_raw:GT=hom:DP=9:FQ=1:SQ=-1;ID=S2504_raw:GT=hom:DP=33:FQ=1:SQ=-1;ID=S2008_raw:GT=hom:DP=8:FQ=1:SQ=-1;ID=S2509_raw:GT=hom:DP=44:FQ=1:SQ=-1;ID=S2508_raw:GT=hom:DP=54:FQ=1:SQ=-1;ID=S2666_raw:GT=hom:DP=60:FQ=1:SQ=-1;ID=S2669_raw:GT=hom:DP=40:FQ=1:SQ=-1;ID=S2166_raw:GT=hom:DP=39:FQ=1:SQ=-1;ID=S1775_raw:GT=hom:DP=17:FQ=1:SQ=-1;ID=S2501_raw:GT=hom:DP=29:FQ=1:SQ=-1;ID=S2500_raw:GT=hom:DP=50:FQ=1:SQ=-1;ID=S2502_raw:GT=hom:DP=52:FQ=1:SQ=-1;ID=S2505_raw:GT=hom:DP=40:FQ=1:SQ=-1;ID=S2176_raw:GT=hom:DP=58:FQ=1:SQ=-1;ID=S2506_raw:GT=hom:DP=36:FQ=1:SQ=-1;ID=S2667_raw:GT=hom:DP=40:FQ=1:SQ=-1;
4	521068	521068	A	T	intronic	PIGG	.	.	rs7697650	.	ID=S2009_raw:GT=hom:DP=10:FQ=1:SQ=-1;ID=S2503_raw:GT=hom:DP=46:FQ=1:SQ=-1;ID=S2507_raw:GT=hom:DP=36:FQ=1:SQ=-1;ID=S2668_raw:GT=hom:DP=60:FQ=1:SQ=-1;ID=S2012_raw:GT=hom:DP=11:FQ=1:SQ=-1;ID=S2010_raw:GT=hom:DP=15:FQ=1:SQ=-1;ID=S2662_raw:GT=hom:DP=75:FQ=1:SQ=-1;ID=S2663_raw:GT=hom:DP=68:FQ=1:SQ=-1;ID=S2011_raw:GT=hom:DP=15:FQ=1:SQ=-1;ID=S2504_raw:GT=hom:DP=53:FQ=1:SQ=-1;ID=S2008_raw:GT=hom:DP=14:FQ=1:SQ=-1;ID=S2509_raw:GT=hom:DP=56:FQ=1:SQ=-1;ID=S2508_raw:GT=hom:DP=42:FQ=1:SQ=-1;ID=S2666_raw:GT=hom:DP=63:FQ=1:SQ=-1;ID=S2669_raw:GT=hom:DP=29:FQ=1:SQ=-1;ID=S2166_raw:GT=hom:DP=5:FQ=1:SQ=-1;ID=S1775_raw:GT=hom:DP=2:FQ=1:SQ=-1;ID=S2501_raw:GT=hom:DP=33:FQ=0.97:SQ=-1;ID=S2500_raw:GT=hom:DP=52:FQ=1:SQ=-1;ID=S2502_raw:GT=hom:DP=44:FQ=1:SQ=-1;ID=S2505_raw:GT=hom:DP=42:FQ=1:SQ=-1;ID=S2176_raw:GT=hom:DP=9:FQ=1:SQ=-1;ID=S2506_raw:GT=hom:DP=27:FQ=1:SQ=-1;ID=S2667_raw:GT=hom:DP=33:FQ=1:SQ=-1;
17	16221275	16221275	-	C	intronic	PIGL	.	.	.	.	ID=S2009_raw:GT=hom:DP=45:FQ=1:SQ=-1;ID=S2503_raw:GT=hom:DP=30:FQ=1:SQ=-1;ID=S2507_raw:GT=hom:DP=39:FQ=1:SQ=-1;ID=S2668_raw:GT=hom:DP=36:FQ=1:SQ=-1;ID=S2012_raw:GT=hom:DP=51:FQ=1:SQ=-1;ID=S2010_raw:GT=hom:DP=52:FQ=1:SQ=-1;ID=S2662_raw:GT=hom:DP=31:FQ=1:SQ=-1;ID=S2663_raw:GT=hom:DP=30:FQ=1:SQ=-1;ID=S2011_raw:GT=hom:DP=53:FQ=1:SQ=-1;ID=S2504_raw:GT=hom:DP=37:FQ=1:SQ=-1;ID=S2008_raw:GT=hom:DP=51:FQ=1:SQ=-1;ID=S2509_raw:GT=hom:DP=34:FQ=1:SQ=-1;ID=S2508_raw:GT=hom:DP=52:FQ=1:SQ=-1;ID=S2666_raw:GT=hom:DP=30:FQ=1:SQ=-1;ID=S2669_raw:GT=hom:DP=24:FQ=1:SQ=-1;ID=S2166_raw:GT=hom:DP=16:FQ=1:SQ=-1;ID=S1775_raw:GT=hom:DP=7:FQ=1:SQ=-1;ID=S2501_raw:GT=hom:DP=19:FQ=1:SQ=-1;ID=S2500_raw:GT=hom:DP=31:FQ=1:SQ=-1;ID=S2502_raw:GT=hom:DP=34:FQ=1:SQ=-1;ID=S2505_raw:GT=hom:DP=35:FQ=1:SQ=-1;ID=S2176_raw:GT=hom:DP=33:FQ=1:SQ=-1;ID=S2506_raw:GT=hom:DP=32:FQ=1:SQ=-1;ID=S2667_raw:GT=hom:DP=31:FQ=1:SQ=-1;
21	38444863	38444863	C	T	exonic	PIGP	nonsynonymous SNV	PIGP:NM_153681:exon1:c.25G>A:p.A9T	rs2507733	.	ID=S2009_raw:GT=hom:DP=135:FQ=1:SQ=-1;ID=S2503_raw:GT=hom:DP=112:FQ=1:SQ=-1;ID=S2507_raw:GT=hom:DP=112:FQ=1:SQ=-1;ID=S2668_raw:GT=hom:DP=154:FQ=1:SQ=-1;ID=S2012_raw:GT=hom:DP=142:FQ=1:SQ=-1;ID=S2010_raw:GT=hom:DP=137:FQ=1:SQ=-1;ID=S2662_raw:GT=hom:DP=183:FQ=1:SQ=-1;ID=S2663_raw:GT=hom:DP=134:FQ=1:SQ=-1;ID=S2011_raw:GT=hom:DP=226:FQ=1:SQ=-1;ID=S2504_raw:GT=hom:DP=132:FQ=1:SQ=-1;ID=S2008_raw:GT=hom:DP=153:FQ=1:SQ=-1;ID=S2509_raw:GT=hom:DP=119:FQ=1:SQ=-1;ID=S2508_raw:GT=hom:DP=108:FQ=1:SQ=-1;ID=S2666_raw:GT=hom:DP=117:FQ=1:SQ=-1;ID=S2669_raw:GT=hom:DP=121:FQ=1:SQ=-1;ID=S2166_raw:GT=hom:DP=107:FQ=1:SQ=-1;ID=S1775_raw:GT=hom:DP=96:FQ=1:SQ=-1;ID=S2501_raw:GT=hom:DP=109:FQ=1:SQ=-1;ID=S2500_raw:GT=hom:DP=122:FQ=1:SQ=-1;ID=S2502_raw:GT=hom:DP=116:FQ=1:SQ=-1;ID=S2505_raw:GT=hom:DP=94:FQ=1:SQ=-1;ID=S2176_raw:GT=hom:DP=116:FQ=1:SQ=-1;ID=S2506_raw:GT=hom:DP=85:FQ=1:SQ=-1;ID=S2667_raw:GT=hom:DP=124:FQ=1:SQ=-1;
11	17113483	17113483	C	T	intronic	PIK3C2A	.	.	rs7113396	.	ID=S2009_raw:GT=hom:DP=18:FQ=1:SQ=-1;ID=S2503_raw:GT=hom:DP=46:FQ=1:SQ=-1;ID=S2507_raw:GT=hom:DP=44:FQ=1:SQ=-1;ID=S2668_raw:GT=hom:DP=86:FQ=1:SQ=-1;ID=S2012_raw:GT=hom:DP=26:FQ=1:SQ=-1;ID=S2010_raw:GT=hom:DP=20:FQ=1:SQ=-1;ID=S2662_raw:GT=hom:DP=4:FQ=1:SQ=-1;ID=S2663_raw:GT=hom:DP=50:FQ=1:SQ=-1;ID=S2011_raw:GT=hom:DP=28:FQ=1:SQ=-1;ID=S2504_raw:GT=hom:DP=47:FQ=1:SQ=-1;ID=S2008_raw:GT=hom:DP=38:FQ=1:SQ=-1;ID=S2509_raw:GT=hom:DP=46:FQ=1:SQ=-1;ID=S2508_raw:GT=hom:DP=50:FQ=1:SQ=-1;ID=S2666_raw:GT=hom:DP=82:FQ=1:SQ=-1;ID=S2669_raw:GT=hom:DP=13:FQ=1:SQ=-1;ID=S2166_raw:GT=hom:DP=6:FQ=1:SQ=-1;ID=S1775_raw:GT=hom:DP=9:FQ=1:SQ=-1;ID=S2501_raw:GT=hom:DP=56:FQ=1:SQ=-1;ID=S2500_raw:GT=hom:DP=55:FQ=1:SQ=-1;ID=S2502_raw:GT=hom:DP=57:FQ=1:SQ=-1;ID=S2505_raw:GT=hom:DP=58:FQ=1:SQ=-1;ID=S2176_raw:GT=hom:DP=11:FQ=1:SQ=-1;ID=S2506_raw:GT=hom:DP=44:FQ=1:SQ=-1;ID=S2667_raw:GT=hom:DP=69:FQ=1:SQ=-1;
11	17172133	17172133	T	C	exonic	PIK3C2A	synonymous SNV	PIK3C2A:NM_001321380:exon3:c.99A>G:p.A33A,PIK3C2A:NM_002645:exon4:c.1239A>G:p.A413A,PIK3C2A:NM_001321378:exon5:c.1239A>G:p.A413A	rs2302511	.	ID=S2009_raw:GT=hom:DP=119:FQ=1:SQ=-1;ID=S2503_raw:GT=hom:DP=88:FQ=1:SQ=-1;ID=S2507_raw:GT=hom:DP=81:FQ=0.98:SQ=-1;ID=S2668_raw:GT=hom:DP=99:FQ=1:SQ=-1;ID=S2012_raw:GT=hom:DP=170:FQ=1:SQ=-1;ID=S2010_raw:GT=hom:DP=176:FQ=1:SQ=-1;ID=S2662_raw:GT=hom:DP=15:FQ=1:SQ=-1;ID=S2663_raw:GT=hom:DP=73:FQ=1:SQ=-1;ID=S2011_raw:GT=hom:DP=217:FQ=1:SQ=-1;ID=S2504_raw:GT=hom:DP=91:FQ=1:SQ=-1;ID=S2008_raw:GT=hom:DP=146:FQ=0.99:SQ=-1;ID=S2509_raw:GT=hom:DP=83:FQ=1:SQ=-1;ID=S2508_raw:GT=hom:DP=103:FQ=1:SQ=-1;ID=S2666_raw:GT=hom:DP=109:FQ=1:SQ=-1;ID=S2669_raw:GT=hom:DP=63:FQ=1:SQ=-1;ID=S2166_raw:GT=hom:DP=245:FQ=1:SQ=-1;ID=S1775_raw:GT=hom:DP=194:FQ=1:SQ=-1;ID=S2501_raw:GT=hom:DP=68:FQ=1:SQ=-1;ID=S2500_raw:GT=hom:DP=74:FQ=1:SQ=-1;ID=S2502_raw:GT=hom:DP=96:FQ=1:SQ=-1;ID=S2505_raw:GT=hom:DP=103:FQ=1:SQ=-1;ID=S2176_raw:GT=hom:DP=245:FQ=1:SQ=-1;ID=S2506_raw:GT=hom:DP=72:FQ=1:SQ=-1;ID=S2667_raw:GT=hom:DP=43:FQ=1:SQ=-1;
6	51875250	51875250	A	C	exonic;splicing	PKHD1	nonsynonymous SNV	PKHD1:NM_138694:exon35:c.5608T>G:p.L1870V,PKHD1:NM_170724:exon35:c.5608T>G:p.L1870V	rs2435322	Benign	ID=S2009_raw:GT=hom:DP=23:FQ=1:SQ=-1;ID=S2503_raw:GT=hom:DP=88:FQ=1:SQ=-1;ID=S2507_raw:GT=hom:DP=64:FQ=1:SQ=-1;ID=S2668_raw:GT=hom:DP=79:FQ=1:SQ=-1;ID=S2012_raw:GT=hom:DP=30:FQ=1:SQ=-1;ID=S2010_raw:GT=hom:DP=35:FQ=1:SQ=-1;ID=S2662_raw:GT=hom:DP=11:FQ=1:SQ=-1;ID=S2663_raw:GT=hom:DP=90:FQ=1:SQ=-1;ID=S2011_raw:GT=hom:DP=59:FQ=1:SQ=-1;ID=S2504_raw:GT=hom:DP=106:FQ=1:SQ=-1;ID=S2008_raw:GT=hom:DP=47:FQ=1:SQ=-1;ID=S2509_raw:GT=hom:DP=85:FQ=1:SQ=-1;ID=S2508_raw:GT=hom:DP=80:FQ=1:SQ=-1;ID=S2666_raw:GT=hom:DP=67:FQ=1:SQ=-1;ID=S2669_raw:GT=hom:DP=47:FQ=1:SQ=-1;ID=S2166_raw:GT=hom:DP=94:FQ=1:SQ=-1;ID=S1775_raw:GT=hom:DP=26:FQ=1:SQ=-1;ID=S2501_raw:GT=hom:DP=93:FQ=1:SQ=-1;ID=S2500_raw:GT=hom:DP=92:FQ=1:SQ=-1;ID=S2502_raw:GT=hom:DP=95:FQ=1:SQ=-1;ID=S2505_raw:GT=hom:DP=89:FQ=1:SQ=-1;ID=S2176_raw:GT=hom:DP=98:FQ=1:SQ=-1;ID=S2506_raw:GT=hom:DP=75:FQ=1:SQ=-1;ID=S2667_raw:GT=hom:DP=58:FQ=1:SQ=-1;
6	51914898	51914898	T	C	intronic	PKHD1	.	.	rs9395759	.	ID=S2009_raw:GT=hom:DP=7:FQ=1:SQ=-1;ID=S2503_raw:GT=hom:DP=40:FQ=1:SQ=-1;ID=S2507_raw:GT=hom:DP=34:FQ=1:SQ=-1;ID=S2668_raw:GT=hom:DP=33:FQ=1:SQ=-1;ID=S2012_raw:GT=hom:DP=10:FQ=1:SQ=-1;ID=S2010_raw:GT=hom:DP=4:FQ=1:SQ=-1;ID=S2662_raw:GT=hom:DP=18:FQ=1:SQ=-1;ID=S2663_raw:GT=hom:DP=43:FQ=1:SQ=-1;ID=S2011_raw:GT=hom:DP=13:FQ=1:SQ=-1;ID=S2504_raw:GT=hom:DP=34:FQ=1:SQ=-1;ID=S2008_raw:GT=hom:DP=9:FQ=1:SQ=-1;ID=S2509_raw:GT=hom:DP=36:FQ=1:SQ=-1;ID=S2508_raw:GT=hom:DP=42:FQ=1:SQ=-1;ID=S2666_raw:GT=hom:DP=24:FQ=1:SQ=-1;ID=S2669_raw:GT=hom:DP=40:FQ=1:SQ=-1;ID=S2166_raw:GT=hom:DP=20:FQ=1:SQ=-1;ID=S1775_raw:GT=hom:DP=9:FQ=1:SQ=-1;ID=S2501_raw:GT=hom:DP=31:FQ=1:SQ=-1;ID=S2500_raw:GT=hom:DP=33:FQ=1:SQ=-1;ID=S2502_raw:GT=hom:DP=40:FQ=1:SQ=-1;ID=S2505_raw:GT=hom:DP=43:FQ=1:SQ=-1;ID=S2176_raw:GT=hom:DP=21:FQ=1:SQ=-1;ID=S2506_raw:GT=hom:DP=22:FQ=1:SQ=-1;ID=S2667_raw:GT=hom:DP=25:FQ=1:SQ=-1;
6	51924774	51924774	A	G	exonic	PKHD1	synonymous SNV	PKHD1:NM_138694:exon15:c.1185T>C:p.D395D,PKHD1:NM_170724:exon15:c.1185T>C:p.D395D	rs1896976	Benign	ID=S2009_raw:GT=hom:DP=82:FQ=1:SQ=-1;ID=S2503_raw:GT=hom:DP=115:FQ=1:SQ=-1;ID=S2507_raw:GT=hom:DP=91:FQ=1:SQ=-1;ID=S2668_raw:GT=hom:DP=137:FQ=1:SQ=-1;ID=S2012_raw:GT=hom:DP=91:FQ=1:SQ=-1;ID=S2010_raw:GT=hom:DP=84:FQ=1:SQ=-1;ID=S2662_raw:GT=hom:DP=12:FQ=1:SQ=-1;ID=S2663_raw:GT=hom:DP=106:FQ=1:SQ=-1;ID=S2011_raw:GT=hom:DP=123:FQ=1:SQ=-1;ID=S2504_raw:GT=hom:DP=126:FQ=1:SQ=-1;ID=S2008_raw:GT=hom:DP=78:FQ=1:SQ=-1;ID=S2509_raw:GT=hom:DP=89:FQ=1:SQ=-1;ID=S2508_raw:GT=hom:DP=101:FQ=1:SQ=-1;ID=S2666_raw:GT=hom:DP=129:FQ=1:SQ=-1;ID=S2669_raw:GT=hom:DP=106:FQ=1:SQ=-1;ID=S2166_raw:GT=hom:DP=182:FQ=1:SQ=-1;ID=S1775_raw:GT=hom:DP=164:FQ=1:SQ=-1;ID=S2501_raw:GT=hom:DP=92:FQ=1:SQ=-1;ID=S2500_raw:GT=hom:DP=112:FQ=1:SQ=-1;ID=S2502_raw:GT=hom:DP=85:FQ=1:SQ=-1;ID=S2505_raw:GT=hom:DP=104:FQ=1:SQ=-1;ID=S2176_raw:GT=hom:DP=205:FQ=0.99:SQ=-1;ID=S2506_raw:GT=hom:DP=75:FQ=1:SQ=-1;ID=S2667_raw:GT=hom:DP=86:FQ=1:SQ=-1;
3	1,27E+08	1,27E+08	T	C	intronic	PLXNA1	.	.	rs6439030	.	ID=S2009_raw:GT=hom:DP=26:FQ=1:SQ=-1;ID=S2503_raw:GT=hom:DP=53:FQ=1:SQ=-1;ID=S2507_raw:GT=hom:DP=55:FQ=1:SQ=-1;ID=S2668_raw:GT=hom:DP=72:FQ=1:SQ=-1;ID=S2012_raw:GT=hom:DP=22:FQ=1:SQ=-1;ID=S2010_raw:GT=hom:DP=24:FQ=1:SQ=-1;ID=S2662_raw:GT=hom:DP=122:FQ=1:SQ=-1;ID=S2663_raw:GT=hom:DP=65:FQ=1:SQ=-1;ID=S2011_raw:GT=hom:DP=31:FQ=1:SQ=-1;ID=S2504_raw:GT=hom:DP=70:FQ=1:SQ=-1;ID=S2008_raw:GT=hom:DP=17:FQ=1:SQ=-1;ID=S2509_raw:GT=hom:DP=35:FQ=1:SQ=-1;ID=S2508_raw:GT=hom:DP=79:FQ=1:SQ=-1;ID=S2666_raw:GT=hom:DP=43:FQ=1:SQ=-1;ID=S2669_raw:GT=hom:DP=64:FQ=1:SQ=-1;ID=S2166_raw:GT=hom:DP=33:FQ=1:SQ=-1;ID=S1775_raw:GT=hom:DP=39:FQ=1:SQ=-1;ID=S2501_raw:GT=hom:DP=64:FQ=1:SQ=-1;ID=S2500_raw:GT=hom:DP=57:FQ=1:SQ=-1;ID=S2502_raw:GT=hom:DP=69:FQ=1:SQ=-1;ID=S2505_raw:GT=hom:DP=82:FQ=1:SQ=-1;ID=S2176_raw:GT=hom:DP=66:FQ=1:SQ=-1;ID=S2506_raw:GT=hom:DP=61:FQ=1:SQ=-1;ID=S2667_raw:GT=hom:DP=77:FQ=1:SQ=-1;
3	1,27E+08	1,27E+08	T	C	intronic	PLXNA1	.	.	rs6789728	.	ID=S2009_raw:GT=hom:DP=30:FQ=1:SQ=-1;ID=S2503_raw:GT=hom:DP=39:FQ=1:SQ=-1;ID=S2507_raw:GT=hom:DP=29:FQ=1:SQ=-1;ID=S2668_raw:GT=hom:DP=11:FQ=1:SQ=-1;ID=S2012_raw:GT=hom:DP=20:FQ=1:SQ=-1;ID=S2010_raw:GT=hom:DP=22:FQ=1:SQ=-1;ID=S2662_raw:GT=hom:DP=29:FQ=1:SQ=-1;ID=S2663_raw:GT=hom:DP=30:FQ=1:SQ=-1;ID=S2011_raw:GT=hom:DP=29:FQ=1:SQ=-1;ID=S2504_raw:GT=hom:DP=40:FQ=1:SQ=-1;ID=S2008_raw:GT=hom:DP=26:FQ=1:SQ=-1;ID=S2509_raw:GT=hom:DP=26:FQ=1:SQ=-1;ID=S2508_raw:GT=hom:DP=44:FQ=1:SQ=-1;ID=S2666_raw:GT=hom:DP=17:FQ=1:SQ=-1;ID=S2669_raw:GT=hom:DP=20:FQ=1:SQ=-1;ID=S2166_raw:GT=hom:DP=43:FQ=1:SQ=-1;ID=S1775_raw:GT=hom:DP=15:FQ=1:SQ=-1;ID=S2501_raw:GT=hom:DP=45:FQ=1:SQ=-1;ID=S2500_raw:GT=hom:DP=32:FQ=1:SQ=-1;ID=S2502_raw:GT=hom:DP=39:FQ=1:SQ=-1;ID=S2505_raw:GT=hom:DP=29:FQ=1:SQ=-1;ID=S2176_raw:GT=hom:DP=51:FQ=1:SQ=-1;ID=S2506_raw:GT=hom:DP=36:FQ=1:SQ=-1;ID=S2667_raw:GT=hom:DP=28:FQ=1:SQ=-1;
3	1,27E+08	1,27E+08	G	A	splicing	PLXNA1	.	NM_032242:exon30:c.5595+10G>A	rs6439031	.	ID=S2009_raw:GT=hom:DP=35:FQ=1:SQ=-1;ID=S2503_raw:GT=hom:DP=118:FQ=1:SQ=-1;ID=S2507_raw:GT=hom:DP=139:FQ=1:SQ=-1;ID=S2668_raw:GT=hom:DP=143:FQ=1:SQ=-1;ID=S2012_raw:GT=hom:DP=40:FQ=1:SQ=-1;ID=S2010_raw:GT=hom:DP=26:FQ=1:SQ=-1;ID=S2662_raw:GT=hom:DP=237:FQ=1:SQ=-1;ID=S2663_raw:GT=hom:DP=147:FQ=1:SQ=-1;ID=S2011_raw:GT=hom:DP=28:FQ=1:SQ=-1;ID=S2504_raw:GT=hom:DP=143:FQ=1:SQ=-1;ID=S2008_raw:GT=hom:DP=32:FQ=1:SQ=-1;ID=S2509_raw:GT=hom:DP=132:FQ=1:SQ=-1;ID=S2508_raw:GT=hom:DP=152:FQ=1:SQ=-1;ID=S2666_raw:GT=hom:DP=121:FQ=1:SQ=-1;ID=S2669_raw:GT=hom:DP=111:FQ=1:SQ=-1;ID=S2166_raw:GT=hom:DP=179:FQ=1:SQ=-1;ID=S1775_raw:GT=hom:DP=126:FQ=1:SQ=-1;ID=S2501_raw:GT=hom:DP=141:FQ=0.99:SQ=-1;ID=S2500_raw:GT=hom:DP=127:FQ=1:SQ=-1;ID=S2502_raw:GT=hom:DP=158:FQ=1:SQ=-1;ID=S2505_raw:GT=hom:DP=137:FQ=1:SQ=-1;ID=S2176_raw:GT=hom:DP=232:FQ=1:SQ=-1;ID=S2506_raw:GT=hom:DP=129:FQ=1:SQ=-1;ID=S2667_raw:GT=hom:DP=118:FQ=1:SQ=-1;
1	1,51E+08	1,51E+08	T	C	UTR3	POGZ	.	NM_207171:c.*30A>G;NM_001194938:c.*30A>G;NM_145796:c.*30A>G;NM_015100:c.*30A>G;NM_001194937:c.*30A>G	rs6667714	.	ID=S2009_raw:GT=hom:DP=275:FQ=1:SQ=-1;ID=S2503_raw:GT=hom:DP=20:FQ=1:SQ=-1;ID=S2507_raw:GT=hom:DP=18:FQ=1:SQ=-1;ID=S2668_raw:GT=hom:DP=18:FQ=1:SQ=-1;ID=S2012_raw:GT=hom:DP=304:FQ=1:SQ=-1;ID=S2010_raw:GT=hom:DP=334:FQ=1:SQ=-1;ID=S2662_raw:GT=hom:DP=17:FQ=1:SQ=-1;ID=S2663_raw:GT=hom:DP=14:FQ=1:SQ=-1;ID=S2011_raw:GT=hom:DP=476:FQ=1:SQ=-1;ID=S2504_raw:GT=hom:DP=23:FQ=1:SQ=-1;ID=S2008_raw:GT=hom:DP=360:FQ=1:SQ=-1;ID=S2509_raw:GT=hom:DP=21:FQ=1:SQ=-1;ID=S2508_raw:GT=hom:DP=15:FQ=1:SQ=-1;ID=S2666_raw:GT=hom:DP=13:FQ=1:SQ=-1;ID=S2669_raw:GT=hom:DP=20:FQ=1:SQ=-1;ID=S2166_raw:GT=hom:DP=40:FQ=1:SQ=-1;ID=S1775_raw:GT=hom:DP=29:FQ=1:SQ=-1;ID=S2501_raw:GT=hom:DP=24:FQ=1:SQ=-1;ID=S2500_raw:GT=hom:DP=19:FQ=1:SQ=-1;ID=S2502_raw:GT=hom:DP=17:FQ=1:SQ=-1;ID=S2505_raw:GT=hom:DP=17:FQ=1:SQ=-1;ID=S2176_raw:GT=hom:DP=48:FQ=1:SQ=-1;ID=S2506_raw:GT=hom:DP=17:FQ=1:SQ=-1;ID=S2667_raw:GT=hom:DP=20:FQ=1:SQ=-1;
12	1,33E+08	1,33E+08	T	C	intronic	POLE	.	.	rs5744889	Benign	ID=S2009_raw:GT=hom:DP=54:FQ=1:SQ=-1;ID=S2503_raw:GT=hom:DP=100:FQ=1:SQ=-1;ID=S2507_raw:GT=hom:DP=97:FQ=1:SQ=-1;ID=S2668_raw:GT=hom:DP=75:FQ=1:SQ=-1;ID=S2012_raw:GT=hom:DP=86:FQ=1:SQ=-1;ID=S2010_raw:GT=hom:DP=96:FQ=1:SQ=-1;ID=S2662_raw:GT=hom:DP=88:FQ=1:SQ=-1;ID=S2663_raw:GT=hom:DP=84:FQ=1:SQ=-1;ID=S2011_raw:GT=hom:DP=111:FQ=1:SQ=-1;ID=S2504_raw:GT=hom:DP=88:FQ=1:SQ=-1;ID=S2008_raw:GT=hom:DP=79:FQ=1:SQ=-1;ID=S2509_raw:GT=hom:DP=82:FQ=1:SQ=-1;ID=S2508_raw:GT=hom:DP=80:FQ=1:SQ=-1;ID=S2666_raw:GT=hom:DP=68:FQ=1:SQ=-1;ID=S2669_raw:GT=hom:DP=58:FQ=1:SQ=-1;ID=S2166_raw:GT=hom:DP=56:FQ=1:SQ=-1;ID=S1775_raw:GT=hom:DP=41:FQ=1:SQ=-1;ID=S2501_raw:GT=hom:DP=109:FQ=1:SQ=-1;ID=S2500_raw:GT=hom:DP=83:FQ=1:SQ=-1;ID=S2502_raw:GT=hom:DP=103:FQ=1:SQ=-1;ID=S2505_raw:GT=hom:DP=75:FQ=1:SQ=-1;ID=S2176_raw:GT=hom:DP=63:FQ=1:SQ=-1;ID=S2506_raw:GT=hom:DP=68:FQ=1:SQ=-1;ID=S2667_raw:GT=hom:DP=69:FQ=1:SQ=-1;
12	1,33E+08	1,33E+08	A	C	intronic	POLE	.	.	rs111877885	.	ID=S2009_raw:GT=hom:DP=6:FQ=1:SQ=-1;ID=S2503_raw:GT=hom:DP=7:FQ=1:SQ=-1;ID=S2507_raw:GT=hom:DP=9:FQ=1:SQ=-1;ID=S2668_raw:GT=hom:DP=5:FQ=1:SQ=-1;ID=S2012_raw:GT=hom:DP=4:FQ=1:SQ=-1;ID=S2010_raw:GT=hom:DP=3:FQ=1:SQ=-1;ID=S2662_raw:GT=hom:DP=15:FQ=1:SQ=-1;ID=S2663_raw:GT=hom:DP=5:FQ=1:SQ=-1;ID=S2011_raw:GT=hom:DP=1:FQ=1:SQ=-1;ID=S2504_raw:GT=hom:DP=7:FQ=1:SQ=-1;ID=S2008_raw:GT=hom:DP=5:FQ=1:SQ=-1;ID=S2509_raw:GT=hom:DP=11:FQ=1:SQ=-1;ID=S2508_raw:GT=hom:DP=3:FQ=1:SQ=-1;ID=S2666_raw:GT=hom:DP=1:FQ=1:SQ=-1;ID=S2669_raw:GT=hom:DP=8:FQ=1:SQ=-1;ID=S2166_raw:GT=hom:DP=5:FQ=1:SQ=-1;ID=S1775_raw:GT=hom:DP=4:FQ=1:SQ=-1;ID=S2501_raw:GT=hom:DP=6:FQ=1:SQ=-1;ID=S2500_raw:GT=hom:DP=5:FQ=1:SQ=-1;ID=S2502_raw:GT=hom:DP=5:FQ=1:SQ=-1;ID=S2505_raw:GT=hom:DP=4:FQ=1:SQ=-1;ID=S2176_raw:GT=hom:DP=3:FQ=1:SQ=-1;ID=S2506_raw:GT=hom:DP=6:FQ=1:SQ=-1;ID=S2667_raw:GT=hom:DP=9:FQ=1:SQ=-1;
9	1,34E+08	1,34E+08	T	G	intronic	POMT1	.	.	rs4740163	Benign	ID=S2009_raw:GT=hom:DP=4:FQ=1:SQ=-1;ID=S2503_raw:GT=hom:DP=52:FQ=1:SQ=-1;ID=S2507_raw:GT=hom:DP=70:FQ=1:SQ=-1;ID=S2668_raw:GT=hom:DP=78:FQ=1:SQ=-1;ID=S2012_raw:GT=hom:DP=3:FQ=1:SQ=-1;ID=S2010_raw:GT=hom:DP=8:FQ=1:SQ=-1;ID=S2662_raw:GT=hom:DP=107:FQ=1:SQ=-1;ID=S2663_raw:GT=hom:DP=52:FQ=1:SQ=-1;ID=S2011_raw:GT=hom:DP=11:FQ=1:SQ=-1;ID=S2504_raw:GT=hom:DP=73:FQ=1:SQ=-1;ID=S2008_raw:GT=hom:DP=12:FQ=1:SQ=-1;ID=S2509_raw:GT=hom:DP=74:FQ=1:SQ=-1;ID=S2508_raw:GT=hom:DP=61:FQ=1:SQ=-1;ID=S2666_raw:GT=hom:DP=106:FQ=1:SQ=-1;ID=S2669_raw:GT=hom:DP=52:FQ=1:SQ=-1;ID=S2166_raw:GT=hom:DP=49:FQ=1:SQ=-1;ID=S1775_raw:GT=hom:DP=44:FQ=1:SQ=-1;ID=S2501_raw:GT=hom:DP=82:FQ=1:SQ=-1;ID=S2500_raw:GT=hom:DP=80:FQ=1:SQ=-1;ID=S2502_raw:GT=hom:DP=97:FQ=1:SQ=-1;ID=S2505_raw:GT=hom:DP=58:FQ=1:SQ=-1;ID=S2176_raw:GT=hom:DP=54:FQ=1:SQ=-1;ID=S2506_raw:GT=hom:DP=62:FQ=1:SQ=-1;ID=S2667_raw:GT=hom:DP=60:FQ=1:SQ=-1;
7	75610763	75610763	G	A	intronic	POR	.	.	rs2286819	.	ID=S2009_raw:GT=hom:DP=47:FQ=1:SQ=-1;ID=S2503_raw:GT=hom:DP=56:FQ=1:SQ=-1;ID=S2507_raw:GT=hom:DP=64:FQ=1:SQ=-1;ID=S2668_raw:GT=hom:DP=74:FQ=0.97:SQ=-1;ID=S2012_raw:GT=hom:DP=44:FQ=1:SQ=-1;ID=S2010_raw:GT=hom:DP=34:FQ=1:SQ=-1;ID=S2662_raw:GT=hom:DP=146:FQ=0.99:SQ=-1;ID=S2663_raw:GT=hom:DP=103:FQ=1:SQ=-1;ID=S2011_raw:GT=hom:DP=65:FQ=1:SQ=-1;ID=S2504_raw:GT=hom:DP=81:FQ=1:SQ=-1;ID=S2008_raw:GT=hom:DP=46:FQ=1:SQ=-1;ID=S2509_raw:GT=hom:DP=72:FQ=1:SQ=-1;ID=S2508_raw:GT=hom:DP=73:FQ=1:SQ=-1;ID=S2666_raw:GT=hom:DP=74:FQ=0.99:SQ=-1;ID=S2669_raw:GT=hom:DP=68:FQ=1:SQ=-1;ID=S2166_raw:GT=hom:DP=65:FQ=1:SQ=-1;ID=S1775_raw:GT=hom:DP=44:FQ=1:SQ=-1;ID=S2501_raw:GT=hom:DP=83:FQ=1:SQ=-1;ID=S2500_raw:GT=hom:DP=59:FQ=1:SQ=-1;ID=S2502_raw:GT=hom:DP=87:FQ=1:SQ=-1;ID=S2505_raw:GT=hom:DP=83:FQ=1:SQ=-1;ID=S2176_raw:GT=hom:DP=60:FQ=1:SQ=-1;ID=S2506_raw:GT=hom:DP=54:FQ=1:SQ=-1;ID=S2667_raw:GT=hom:DP=91:FQ=1:SQ=-1;
7	75612770	75612770	C	G	intronic	POR	.	.	rs13223707	.	ID=S2009_raw:GT=hom:DP=14:FQ=1:SQ=-1;ID=S2503_raw:GT=hom:DP=58:FQ=1:SQ=-1;ID=S2507_raw:GT=hom:DP=50:FQ=1:SQ=-1;ID=S2668_raw:GT=hom:DP=72:FQ=1:SQ=-1;ID=S2012_raw:GT=hom:DP=13:FQ=1:SQ=-1;ID=S2010_raw:GT=hom:DP=23:FQ=1:SQ=-1;ID=S2662_raw:GT=hom:DP=76:FQ=1:SQ=-1;ID=S2663_raw:GT=hom:DP=40:FQ=1:SQ=-1;ID=S2011_raw:GT=hom:DP=15:FQ=1:SQ=-1;ID=S2504_raw:GT=hom:DP=46:FQ=1:SQ=-1;ID=S2008_raw:GT=hom:DP=28:FQ=1:SQ=-1;ID=S2509_raw:GT=hom:DP=70:FQ=1:SQ=-1;ID=S2508_raw:GT=hom:DP=48:FQ=1:SQ=-1;ID=S2666_raw:GT=hom:DP=47:FQ=1:SQ=-1;ID=S2669_raw:GT=hom:DP=40:FQ=1:SQ=-1;ID=S2166_raw:GT=hom:DP=46:FQ=1:SQ=-1;ID=S1775_raw:GT=hom:DP=25:FQ=1:SQ=-1;ID=S2501_raw:GT=hom:DP=57:FQ=1:SQ=-1;ID=S2500_raw:GT=hom:DP=51:FQ=1:SQ=-1;ID=S2502_raw:GT=hom:DP=50:FQ=0.98:SQ=-1;ID=S2505_raw:GT=hom:DP=60:FQ=1:SQ=-1;ID=S2176_raw:GT=hom:DP=54:FQ=1:SQ=-1;ID=S2506_raw:GT=hom:DP=66:FQ=1:SQ=-1;ID=S2667_raw:GT=hom:DP=50:FQ=1:SQ=-1;
7	75612783	75612783	G	A	intronic	POR	.	.	rs13240147	.	ID=S2009_raw:GT=hom:DP=14:FQ=1:SQ=-1;ID=S2503_raw:GT=hom:DP=64:FQ=1:SQ=-1;ID=S2507_raw:GT=hom:DP=59:FQ=1:SQ=-1;ID=S2668_raw:GT=hom:DP=79:FQ=1:SQ=-1;ID=S2012_raw:GT=hom:DP=16:FQ=1:SQ=-1;ID=S2010_raw:GT=hom:DP=28:FQ=1:SQ=-1;ID=S2662_raw:GT=hom:DP=91:FQ=1:SQ=-1;ID=S2663_raw:GT=hom:DP=50:FQ=1:SQ=-1;ID=S2011_raw:GT=hom:DP=18:FQ=1:SQ=-1;ID=S2504_raw:GT=hom:DP=56:FQ=0.98:SQ=-1;ID=S2008_raw:GT=hom:DP=30:FQ=1:SQ=-1;ID=S2509_raw:GT=hom:DP=77:FQ=1:SQ=-1;ID=S2508_raw:GT=hom:DP=55:FQ=1:SQ=-1;ID=S2666_raw:GT=hom:DP=55:FQ=1:SQ=-1;ID=S2669_raw:GT=hom:DP=41:FQ=1:SQ=-1;ID=S2166_raw:GT=hom:DP=60:FQ=1:SQ=-1;ID=S1775_raw:GT=hom:DP=28:FQ=1:SQ=-1;ID=S2501_raw:GT=hom:DP=66:FQ=1:SQ=-1;ID=S2500_raw:GT=hom:DP=60:FQ=1:SQ=-1;ID=S2502_raw:GT=hom:DP=61:FQ=1:SQ=-1;ID=S2505_raw:GT=hom:DP=69:FQ=1:SQ=-1;ID=S2176_raw:GT=hom:DP=72:FQ=1:SQ=-1;ID=S2506_raw:GT=hom:DP=77:FQ=1:SQ=-1;ID=S2667_raw:GT=hom:DP=56:FQ=1:SQ=-1;
7	75614029	75614029	T	C	intronic	POR	.	.	rs4732515	.	ID=S2009_raw:GT=hom:DP=27:FQ=1:SQ=-1;ID=S2503_raw:GT=hom:DP=44:FQ=1:SQ=-1;ID=S2507_raw:GT=hom:DP=31:FQ=1:SQ=-1;ID=S2668_raw:GT=hom:DP=42:FQ=1:SQ=-1;ID=S2012_raw:GT=hom:DP=21:FQ=1:SQ=-1;ID=S2010_raw:GT=hom:DP=38:FQ=1:SQ=-1;ID=S2662_raw:GT=hom:DP=52:FQ=1:SQ=-1;ID=S2663_raw:GT=hom:DP=27:FQ=1:SQ=-1;ID=S2011_raw:GT=hom:DP=32:FQ=1:SQ=-1;ID=S2504_raw:GT=hom:DP=47:FQ=1:SQ=-1;ID=S2008_raw:GT=hom:DP=33:FQ=1:SQ=-1;ID=S2509_raw:GT=hom:DP=33:FQ=1:SQ=-1;ID=S2508_raw:GT=hom:DP=49:FQ=1:SQ=-1;ID=S2666_raw:GT=hom:DP=19:FQ=1:SQ=-1;ID=S2669_raw:GT=hom:DP=25:FQ=1:SQ=-1;ID=S2166_raw:GT=hom:DP=46:FQ=1:SQ=-1;ID=S1775_raw:GT=hom:DP=51:FQ=1:SQ=-1;ID=S2501_raw:GT=hom:DP=45:FQ=1:SQ=-1;ID=S2500_raw:GT=hom:DP=20:FQ=1:SQ=-1;ID=S2502_raw:GT=hom:DP=41:FQ=1:SQ=-1;ID=S2505_raw:GT=hom:DP=38:FQ=1:SQ=-1;ID=S2176_raw:GT=hom:DP=62:FQ=1:SQ=-1;ID=S2506_raw:GT=hom:DP=36:FQ=1:SQ=-1;ID=S2667_raw:GT=hom:DP=31:FQ=1:SQ=-1;
7	75614082	75614082	C	G	intronic	POR	.	.	rs4732516	Benign	ID=S2009_raw:GT=hom:DP=52:FQ=1:SQ=-1;ID=S2503_raw:GT=hom:DP=116:FQ=1:SQ=-1;ID=S2507_raw:GT=hom:DP=90:FQ=1:SQ=-1;ID=S2668_raw:GT=hom:DP=91:FQ=1:SQ=-1;ID=S2012_raw:GT=hom:DP=57:FQ=1:SQ=-1;ID=S2010_raw:GT=hom:DP=82:FQ=1:SQ=-1;ID=S2662_raw:GT=hom:DP=167:FQ=1:SQ=-1;ID=S2663_raw:GT=hom:DP=75:FQ=1:SQ=-1;ID=S2011_raw:GT=hom:DP=88:FQ=1:SQ=-1;ID=S2504_raw:GT=hom:DP=127:FQ=0.99:SQ=-1;ID=S2008_raw:GT=hom:DP=64:FQ=1:SQ=-1;ID=S2509_raw:GT=hom:DP=108:FQ=1:SQ=-1;ID=S2508_raw:GT=hom:DP=120:FQ=1:SQ=-1;ID=S2666_raw:GT=hom:DP=68:FQ=1:SQ=-1;ID=S2669_raw:GT=hom:DP=61:FQ=1:SQ=-1;ID=S2166_raw:GT=hom:DP=130:FQ=1:SQ=-1;ID=S1775_raw:GT=hom:DP=126:FQ=1:SQ=-1;ID=S2501_raw:GT=hom:DP=104:FQ=1:SQ=-1;ID=S2500_raw:GT=hom:DP=75:FQ=1:SQ=-1;ID=S2502_raw:GT=hom:DP=115:FQ=1:SQ=-1;ID=S2505_raw:GT=hom:DP=117:FQ=1:SQ=-1;ID=S2176_raw:GT=hom:DP=143:FQ=1:SQ=-1;ID=S2506_raw:GT=hom:DP=133:FQ=1:SQ=-1;ID=S2667_raw:GT=hom:DP=105:FQ=1:SQ=-1;
7	75614724	75614724	G	A	intronic	POR	.	.	rs6961174	.	ID=S2009_raw:GT=hom:DP=8:FQ=1:SQ=-1;ID=S2503_raw:GT=hom:DP=3:FQ=1:SQ=-1;ID=S2507_raw:GT=hom:DP=14:FQ=1:SQ=-1;ID=S2668_raw:GT=hom:DP=3:FQ=1:SQ=-1;ID=S2012_raw:GT=hom:DP=5:FQ=1:SQ=-1;ID=S2010_raw:GT=hom:DP=5:FQ=1:SQ=-1;ID=S2662_raw:GT=hom:DP=13:FQ=1:SQ=-1;ID=S2663_raw:GT=hom:DP=6:FQ=1:SQ=-1;ID=S2011_raw:GT=hom:DP=10:FQ=1:SQ=-1;ID=S2504_raw:GT=hom:DP=9:FQ=1:SQ=-1;ID=S2008_raw:GT=hom:DP=5:FQ=1:SQ=-1;ID=S2509_raw:GT=hom:DP=4:FQ=1:SQ=-1;ID=S2508_raw:GT=hom:DP=18:FQ=1:SQ=-1;ID=S2666_raw:GT=hom:DP=2:FQ=1:SQ=-1;ID=S2669_raw:GT=hom:DP=6:FQ=1:SQ=-1;ID=S2166_raw:GT=hom:DP=11:FQ=1:SQ=-1;ID=S1775_raw:GT=hom:DP=6:FQ=1:SQ=-1;ID=S2501_raw:GT=hom:DP=7:FQ=1:SQ=-1;ID=S2500_raw:GT=hom:DP=10:FQ=1:SQ=-1;ID=S2502_raw:GT=hom:DP=14:FQ=1:SQ=-1;ID=S2505_raw:GT=hom:DP=15:FQ=1:SQ=-1;ID=S2176_raw:GT=hom:DP=13:FQ=1:SQ=-1;ID=S2506_raw:GT=hom:DP=11:FQ=1:SQ=-1;ID=S2667_raw:GT=hom:DP=13:FQ=0.92:SQ=-1;
7	75614863	75614863	T	C	intronic	POR	.	.	rs2302431	.	ID=S2009_raw:GT=hom:DP=5:FQ=1:SQ=-1;ID=S2503_raw:GT=hom:DP=58:FQ=1:SQ=-1;ID=S2507_raw:GT=hom:DP=73:FQ=1:SQ=-1;ID=S2668_raw:GT=hom:DP=106:FQ=1:SQ=-1;ID=S2012_raw:GT=hom:DP=4:FQ=1:SQ=-1;ID=S2010_raw:GT=hom:DP=6:FQ=1:SQ=-1;ID=S2662_raw:GT=hom:DP=158:FQ=1:SQ=-1;ID=S2663_raw:GT=hom:DP=70:FQ=1:SQ=-1;ID=S2011_raw:GT=hom:DP=13:FQ=1:SQ=-1;ID=S2504_raw:GT=hom:DP=73:FQ=1:SQ=-1;ID=S2008_raw:GT=hom:DP=15:FQ=1:SQ=-1;ID=S2509_raw:GT=hom:DP=75:FQ=1:SQ=-1;ID=S2508_raw:GT=hom:DP=56:FQ=1:SQ=-1;ID=S2666_raw:GT=hom:DP=48:FQ=1:SQ=-1;ID=S2669_raw:GT=hom:DP=58:FQ=1:SQ=-1;ID=S2166_raw:GT=hom:DP=39:FQ=1:SQ=-1;ID=S1775_raw:GT=hom:DP=35:FQ=1:SQ=-1;ID=S2501_raw:GT=hom:DP=67:FQ=1:SQ=-1;ID=S2500_raw:GT=hom:DP=57:FQ=1:SQ=-1;ID=S2502_raw:GT=hom:DP=70:FQ=1:SQ=-1;ID=S2505_raw:GT=hom:DP=63:FQ=1:SQ=-1;ID=S2176_raw:GT=hom:DP=58:FQ=1:SQ=-1;ID=S2506_raw:GT=hom:DP=77:FQ=1:SQ=-1;ID=S2667_raw:GT=hom:DP=86:FQ=0.99:SQ=-1;
7	75614864	75614864	G	T	intronic	POR	.	.	rs2302432	.	ID=S2009_raw:GT=hom:DP=5:FQ=1:SQ=-1;ID=S2503_raw:GT=hom:DP=57:FQ=0.98:SQ=-1;ID=S2507_raw:GT=hom:DP=71:FQ=1:SQ=-1;ID=S2668_raw:GT=hom:DP=106:FQ=1:SQ=-1;ID=S2012_raw:GT=hom:DP=4:FQ=1:SQ=-1;ID=S2010_raw:GT=hom:DP=6:FQ=1:SQ=-1;ID=S2662_raw:GT=hom:DP=159:FQ=1:SQ=-1;ID=S2663_raw:GT=hom:DP=71:FQ=1:SQ=-1;ID=S2011_raw:GT=hom:DP=13:FQ=1:SQ=-1;ID=S2504_raw:GT=hom:DP=72:FQ=1:SQ=-1;ID=S2008_raw:GT=hom:DP=15:FQ=1:SQ=-1;ID=S2509_raw:GT=hom:DP=72:FQ=1:SQ=-1;ID=S2508_raw:GT=hom:DP=56:FQ=1:SQ=-1;ID=S2666_raw:GT=hom:DP=48:FQ=1:SQ=-1;ID=S2669_raw:GT=hom:DP=57:FQ=1:SQ=-1;ID=S2166_raw:GT=hom:DP=40:FQ=1:SQ=-1;ID=S1775_raw:GT=hom:DP=35:FQ=1:SQ=-1;ID=S2501_raw:GT=hom:DP=67:FQ=1:SQ=-1;ID=S2500_raw:GT=hom:DP=56:FQ=1:SQ=-1;ID=S2502_raw:GT=hom:DP=70:FQ=1:SQ=-1;ID=S2505_raw:GT=hom:DP=62:FQ=1:SQ=-1;ID=S2176_raw:GT=hom:DP=60:FQ=1:SQ=-1;ID=S2506_raw:GT=hom:DP=76:FQ=1:SQ=-1;ID=S2667_raw:GT=hom:DP=84:FQ=1:SQ=-1;
7	75614953	75614953	T	C	exonic	POR	synonymous SNV	POR:NM_000941:exon13:c.1455T>C:p.A485A,POR:NM_001367562:exon14:c.1455T>C:p.A485A	rs2228104	Benign	ID=S2009_raw:GT=hom:DP=14:FQ=1:SQ=-1;ID=S2503_raw:GT=hom:DP=122:FQ=1:SQ=-1;ID=S2507_raw:GT=hom:DP=127:FQ=1:SQ=-1;ID=S2668_raw:GT=hom:DP=154:FQ=1:SQ=-1;ID=S2012_raw:GT=hom:DP=22:FQ=1:SQ=-1;ID=S2010_raw:GT=hom:DP=15:FQ=1:SQ=-1;ID=S2662_raw:GT=hom:DP=303:FQ=1:SQ=-1;ID=S2663_raw:GT=hom:DP=110:FQ=1:SQ=-1;ID=S2011_raw:GT=hom:DP=20:FQ=1:SQ=-1;ID=S2504_raw:GT=hom:DP=144:FQ=1:SQ=-1;ID=S2008_raw:GT=hom:DP=37:FQ=1:SQ=-1;ID=S2509_raw:GT=hom:DP=109:FQ=1:SQ=-1;ID=S2508_raw:GT=hom:DP=109:FQ=0.99:SQ=-1;ID=S2666_raw:GT=hom:DP=107:FQ=0.99:SQ=-1;ID=S2669_raw:GT=hom:DP=113:FQ=1:SQ=-1;ID=S2166_raw:GT=hom:DP=83:FQ=1:SQ=-1;ID=S1775_raw:GT=hom:DP=69:FQ=1:SQ=-1;ID=S2501_raw:GT=hom:DP=121:FQ=1:SQ=-1;ID=S2500_raw:GT=hom:DP=113:FQ=1:SQ=-1;ID=S2502_raw:GT=hom:DP=169:FQ=1:SQ=-1;ID=S2505_raw:GT=hom:DP=139:FQ=1:SQ=-1;ID=S2176_raw:GT=hom:DP=93:FQ=1:SQ=-1;ID=S2506_raw:GT=hom:DP=133:FQ=1:SQ=-1;ID=S2667_raw:GT=hom:DP=156:FQ=1:SQ=-1;
X	82764040	82764040	A	G	exonic	POU3F4	synonymous SNV	POU3F4:NM_000307:exon1:c.708A>G:p.E236E	rs5921978	Benign	ID=S2009_raw:GT=hom:DP=29:FQ=1:SQ=-1;ID=S2503_raw:GT=hom:DP=109:FQ=1:SQ=-1;ID=S2507_raw:GT=hom:DP=79:FQ=1:SQ=-1;ID=S2668_raw:GT=hom:DP=241:FQ=1:SQ=-1;ID=S2012_raw:GT=hom:DP=24:FQ=1:SQ=-1;ID=S2010_raw:GT=hom:DP=88:FQ=1:SQ=-1;ID=S2662_raw:GT=hom:DP=317:FQ=1:SQ=-1;ID=S2663_raw:GT=hom:DP=197:FQ=1:SQ=-1;ID=S2011_raw:GT=hom:DP=55:FQ=1:SQ=-1;ID=S2504_raw:GT=hom:DP=111:FQ=1:SQ=-1;ID=S2008_raw:GT=hom:DP=85:FQ=1:SQ=-1;ID=S2509_raw:GT=hom:DP=91:FQ=1:SQ=-1;ID=S2508_raw:GT=hom:DP=137:FQ=1:SQ=-1;ID=S2666_raw:GT=hom:DP=76:FQ=1:SQ=-1;ID=S2669_raw:GT=hom:DP=121:FQ=1:SQ=-1;ID=S2166_raw:GT=hom:DP=103:FQ=1:SQ=-1;ID=S1775_raw:GT=hom:DP=55:FQ=1:SQ=-1;ID=S2501_raw:GT=hom:DP=112:FQ=1:SQ=-1;ID=S2500_raw:GT=hom:DP=87:FQ=1:SQ=-1;ID=S2502_raw:GT=hom:DP=210:FQ=1:SQ=-1;ID=S2505_raw:GT=hom:DP=120:FQ=1:SQ=-1;ID=S2176_raw:GT=hom:DP=121:FQ=0.99:SQ=-1;ID=S2506_raw:GT=hom:DP=85:FQ=1:SQ=-1;ID=S2667_raw:GT=hom:DP=221:FQ=1:SQ=-1;
X	82764042	82764042	G	C	exonic	POU3F4	nonsynonymous SNV	POU3F4:NM_000307:exon1:c.710G>C:p.G237A	rs5921979	Benign	ID=S2009_raw:GT=hom:DP=29:FQ=1:SQ=-1;ID=S2503_raw:GT=hom:DP=109:FQ=1:SQ=-1;ID=S2507_raw:GT=hom:DP=78:FQ=1:SQ=-1;ID=S2668_raw:GT=hom:DP=242:FQ=1:SQ=-1;ID=S2012_raw:GT=hom:DP=24:FQ=1:SQ=-1;ID=S2010_raw:GT=hom:DP=88:FQ=1:SQ=-1;ID=S2662_raw:GT=hom:DP=315:FQ=1:SQ=-1;ID=S2663_raw:GT=hom:DP=199:FQ=1:SQ=-1;ID=S2011_raw:GT=hom:DP=55:FQ=1:SQ=-1;ID=S2504_raw:GT=hom:DP=110:FQ=1:SQ=-1;ID=S2008_raw:GT=hom:DP=84:FQ=1:SQ=-1;ID=S2509_raw:GT=hom:DP=90:FQ=1:SQ=-1;ID=S2508_raw:GT=hom:DP=138:FQ=1:SQ=-1;ID=S2666_raw:GT=hom:DP=76:FQ=1:SQ=-1;ID=S2669_raw:GT=hom:DP=120:FQ=1:SQ=-1;ID=S2166_raw:GT=hom:DP=103:FQ=1:SQ=-1;ID=S1775_raw:GT=hom:DP=54:FQ=1:SQ=-1;ID=S2501_raw:GT=hom:DP=110:FQ=1:SQ=-1;ID=S2500_raw:GT=hom:DP=87:FQ=1:SQ=-1;ID=S2502_raw:GT=hom:DP=208:FQ=1:SQ=-1;ID=S2505_raw:GT=hom:DP=119:FQ=1:SQ=-1;ID=S2176_raw:GT=hom:DP=121:FQ=1:SQ=-1;ID=S2506_raw:GT=hom:DP=85:FQ=1:SQ=-1;ID=S2667_raw:GT=hom:DP=221:FQ=1:SQ=-1;
7	39436608	39436608	A	T	intronic	POU6F2	.	.	rs4720323	.	ID=S2009_raw:GT=hom:DP=3:FQ=1:SQ=-1;ID=S2503_raw:GT=hom:DP=3:FQ=1:SQ=-1;ID=S2507_raw:GT=hom:DP=8:FQ=1:SQ=-1;ID=S2668_raw:GT=hom:DP=7:FQ=1:SQ=-1;ID=S2012_raw:GT=hom:DP=2:FQ=1:SQ=-1;ID=S2010_raw:GT=hom:DP=7:FQ=1:SQ=-1;ID=S2662_raw:GT=hom:DP=2:FQ=1:SQ=-1;ID=S2663_raw:GT=hom:DP=4:FQ=1:SQ=-1;ID=S2011_raw:GT=hom:DP=6:FQ=1:SQ=-1;ID=S2504_raw:GT=hom:DP=6:FQ=1:SQ=-1;ID=S2008_raw:GT=hom:DP=3:FQ=1:SQ=-1;ID=S2509_raw:GT=hom:DP=3:FQ=1:SQ=-1;ID=S2508_raw:GT=hom:DP=6:FQ=1:SQ=-1;ID=S2666_raw:GT=hom:DP=2:FQ=1:SQ=-1;ID=S2669_raw:GT=hom:DP=5:FQ=1:SQ=-1;ID=S2166_raw:GT=hom:DP=10:FQ=1:SQ=-1;ID=S1775_raw:GT=hom:DP=3:FQ=1:SQ=-1;ID=S2501_raw:GT=hom:DP=8:FQ=1:SQ=-1;ID=S2500_raw:GT=hom:DP=4:FQ=1:SQ=-1;ID=S2502_raw:GT=hom:DP=1:FQ=1:SQ=-1;ID=S2505_raw:GT=hom:DP=1:FQ=1:SQ=-1;ID=S2176_raw:GT=hom:DP=14:FQ=0.93:SQ=-1;ID=S2506_raw:GT=hom:DP=4:FQ=1:SQ=-1;ID=S2667_raw:GT=hom:DP=4:FQ=1:SQ=-1;
12	27800746	27800746	G	T	exonic	PPFIBP1	nonsynonymous SNV	PPFIBP1:NM_001198916:exon6:c.442G>T:p.V148L,PPFIBP1:NM_003622:exon6:c.442G>T:p.V148L,PPFIBP1:NM_177444:exon6:c.442G>T:p.V148L	rs2194816	.	ID=S2009_raw:GT=hom:DP=14:FQ=1:SQ=-1;ID=S2503_raw:GT=hom:DP=56:FQ=1:SQ=-1;ID=S2507_raw:GT=hom:DP=53:FQ=1:SQ=-1;ID=S2668_raw:GT=hom:DP=57:FQ=1:SQ=-1;ID=S2012_raw:GT=hom:DP=20:FQ=1:SQ=-1;ID=S2010_raw:GT=hom:DP=25:FQ=1:SQ=-1;ID=S2662_raw:GT=hom:DP=9:FQ=1:SQ=-1;ID=S2663_raw:GT=hom:DP=67:FQ=1:SQ=-1;ID=S2011_raw:GT=hom:DP=26:FQ=1:SQ=-1;ID=S2504_raw:GT=hom:DP=66:FQ=1:SQ=-1;ID=S2008_raw:GT=hom:DP=24:FQ=1:SQ=-1;ID=S2509_raw:GT=hom:DP=53:FQ=1:SQ=-1;ID=S2508_raw:GT=hom:DP=56:FQ=1:SQ=-1;ID=S2666_raw:GT=hom:DP=48:FQ=1:SQ=-1;ID=S2669_raw:GT=hom:DP=46:FQ=1:SQ=-1;ID=S2166_raw:GT=hom:DP=73:FQ=0.99:SQ=-1;ID=S1775_raw:GT=hom:DP=66:FQ=1:SQ=-1;ID=S2501_raw:GT=hom:DP=43:FQ=0.98:SQ=-1;ID=S2500_raw:GT=hom:DP=57:FQ=1:SQ=-1;ID=S2502_raw:GT=hom:DP=60:FQ=1:SQ=-1;ID=S2505_raw:GT=hom:DP=56:FQ=1:SQ=-1;ID=S2176_raw:GT=hom:DP=89:FQ=1:SQ=-1;ID=S2506_raw:GT=hom:DP=45:FQ=1:SQ=-1;ID=S2667_raw:GT=hom:DP=69:FQ=1:SQ=-1;
12	27813754	27813754	A	G	intronic	PPFIBP1	.	.	rs7132997	.	ID=S2009_raw:GT=hom:DP=21:FQ=1:SQ=-1;ID=S2503_raw:GT=hom:DP=28:FQ=1:SQ=-1;ID=S2507_raw:GT=hom:DP=25:FQ=1:SQ=-1;ID=S2668_raw:GT=hom:DP=34:FQ=1:SQ=-1;ID=S2012_raw:GT=hom:DP=16:FQ=1:SQ=-1;ID=S2010_raw:GT=hom:DP=34:FQ=1:SQ=-1;ID=S2662_raw:GT=hom:DP=2:FQ=1:SQ=-1;ID=S2663_raw:GT=hom:DP=21:FQ=1:SQ=-1;ID=S2011_raw:GT=hom:DP=33:FQ=1:SQ=-1;ID=S2504_raw:GT=hom:DP=17:FQ=1:SQ=-1;ID=S2008_raw:GT=hom:DP=26:FQ=1:SQ=-1;ID=S2509_raw:GT=hom:DP=14:FQ=1:SQ=-1;ID=S2508_raw:GT=hom:DP=18:FQ=1:SQ=-1;ID=S2666_raw:GT=hom:DP=27:FQ=1:SQ=-1;ID=S2669_raw:GT=hom:DP=12:FQ=1:SQ=-1;ID=S2166_raw:GT=hom:DP=13:FQ=1:SQ=-1;ID=S1775_raw:GT=hom:DP=14:FQ=1:SQ=-1;ID=S2501_raw:GT=hom:DP=27:FQ=1:SQ=-1;ID=S2500_raw:GT=hom:DP=23:FQ=1:SQ=-1;ID=S2502_raw:GT=hom:DP=16:FQ=1:SQ=-1;ID=S2505_raw:GT=hom:DP=36:FQ=1:SQ=-1;ID=S2176_raw:GT=hom:DP=27:FQ=1:SQ=-1;ID=S2506_raw:GT=hom:DP=19:FQ=1:SQ=-1;ID=S2667_raw:GT=hom:DP=23:FQ=1:SQ=-1;
12	27813903	27813903	C	T	intronic	PPFIBP1	.	.	rs7133222	.	ID=S2009_raw:GT=hom:DP=39:FQ=1:SQ=-1;ID=S2503_raw:GT=hom:DP=78:FQ=1:SQ=-1;ID=S2507_raw:GT=hom:DP=52:FQ=1:SQ=-1;ID=S2668_raw:GT=hom:DP=51:FQ=1:SQ=-1;ID=S2012_raw:GT=hom:DP=19:FQ=1:SQ=-1;ID=S2010_raw:GT=hom:DP=46:FQ=1:SQ=-1;ID=S2662_raw:GT=hom:DP=2:FQ=1:SQ=-1;ID=S2663_raw:GT=hom:DP=35:FQ=1:SQ=-1;ID=S2011_raw:GT=hom:DP=70:FQ=1:SQ=-1;ID=S2504_raw:GT=hom:DP=51:FQ=1:SQ=-1;ID=S2008_raw:GT=hom:DP=37:FQ=1:SQ=-1;ID=S2509_raw:GT=hom:DP=44:FQ=1:SQ=-1;ID=S2508_raw:GT=hom:DP=63:FQ=1:SQ=-1;ID=S2666_raw:GT=hom:DP=42:FQ=1:SQ=-1;ID=S2669_raw:GT=hom:DP=49:FQ=1:SQ=-1;ID=S2166_raw:GT=hom:DP=19:FQ=1:SQ=-1;ID=S1775_raw:GT=hom:DP=30:FQ=1:SQ=-1;ID=S2501_raw:GT=hom:DP=37:FQ=1:SQ=-1;ID=S2500_raw:GT=hom:DP=50:FQ=1:SQ=-1;ID=S2502_raw:GT=hom:DP=61:FQ=1:SQ=-1;ID=S2505_raw:GT=hom:DP=59:FQ=1:SQ=-1;ID=S2176_raw:GT=hom:DP=47:FQ=1:SQ=-1;ID=S2506_raw:GT=hom:DP=40:FQ=1:SQ=-1;ID=S2667_raw:GT=hom:DP=40:FQ=1:SQ=-1;
12	27813961	27813961	A	G	intronic	PPFIBP1	.	.	rs7133231	.	ID=S2009_raw:GT=hom:DP=20:FQ=1:SQ=-1;ID=S2503_raw:GT=hom:DP=70:FQ=1:SQ=-1;ID=S2507_raw:GT=hom:DP=62:FQ=1:SQ=-1;ID=S2668_raw:GT=hom:DP=67:FQ=1:SQ=-1;ID=S2012_raw:GT=hom:DP=8:FQ=1:SQ=-1;ID=S2010_raw:GT=hom:DP=18:FQ=1:SQ=-1;ID=S2662_raw:GT=hom:DP=5:FQ=1:SQ=-1;ID=S2663_raw:GT=hom:DP=36:FQ=1:SQ=-1;ID=S2011_raw:GT=hom:DP=27:FQ=1:SQ=-1;ID=S2504_raw:GT=hom:DP=61:FQ=1:SQ=-1;ID=S2008_raw:GT=hom:DP=22:FQ=1:SQ=-1;ID=S2509_raw:GT=hom:DP=37:FQ=1:SQ=-1;ID=S2508_raw:GT=hom:DP=61:FQ=1:SQ=-1;ID=S2666_raw:GT=hom:DP=38:FQ=1:SQ=-1;ID=S2669_raw:GT=hom:DP=40:FQ=1:SQ=-1;ID=S2166_raw:GT=hom:DP=31:FQ=1:SQ=-1;ID=S1775_raw:GT=hom:DP=24:FQ=1:SQ=-1;ID=S2501_raw:GT=hom:DP=40:FQ=1:SQ=-1;ID=S2500_raw:GT=hom:DP=57:FQ=1:SQ=-1;ID=S2502_raw:GT=hom:DP=63:FQ=0.97:SQ=-1;ID=S2505_raw:GT=hom:DP=71:FQ=1:SQ=-1;ID=S2176_raw:GT=hom:DP=44:FQ=1:SQ=-1;ID=S2506_raw:GT=hom:DP=36:FQ=1:SQ=-1;ID=S2667_raw:GT=hom:DP=40:FQ=1:SQ=-1;
12	27813973	27813973	C	T	intronic	PPFIBP1	.	.	rs7133328	.	ID=S2009_raw:GT=hom:DP=17:FQ=1:SQ=-1;ID=S2503_raw:GT=hom:DP=57:FQ=1:SQ=-1;ID=S2507_raw:GT=hom:DP=60:FQ=1:SQ=-1;ID=S2668_raw:GT=hom:DP=64:FQ=1:SQ=-1;ID=S2012_raw:GT=hom:DP=6:FQ=1:SQ=-1;ID=S2010_raw:GT=hom:DP=17:FQ=1:SQ=-1;ID=S2662_raw:GT=hom:DP=5:FQ=1:SQ=-1;ID=S2663_raw:GT=hom:DP=35:FQ=1:SQ=-1;ID=S2011_raw:GT=hom:DP=18:FQ=1:SQ=-1;ID=S2504_raw:GT=hom:DP=53:FQ=1:SQ=-1;ID=S2008_raw:GT=hom:DP=17:FQ=1:SQ=-1;ID=S2509_raw:GT=hom:DP=32:FQ=1:SQ=-1;ID=S2508_raw:GT=hom:DP=53:FQ=1:SQ=-1;ID=S2666_raw:GT=hom:DP=36:FQ=1:SQ=-1;ID=S2669_raw:GT=hom:DP=39:FQ=1:SQ=-1;ID=S2166_raw:GT=hom:DP=25:FQ=1:SQ=-1;ID=S1775_raw:GT=hom:DP=25:FQ=1:SQ=-1;ID=S2501_raw:GT=hom:DP=41:FQ=1:SQ=-1;ID=S2500_raw:GT=hom:DP=54:FQ=1:SQ=-1;ID=S2502_raw:GT=hom:DP=56:FQ=1:SQ=-1;ID=S2505_raw:GT=hom:DP=71:FQ=1:SQ=-1;ID=S2176_raw:GT=hom:DP=45:FQ=1:SQ=-1;ID=S2506_raw:GT=hom:DP=38:FQ=1:SQ=-1;ID=S2667_raw:GT=hom:DP=32:FQ=1:SQ=-1;
12	27813981	27813981	G	C	intronic	PPFIBP1	.	.	rs7133545	.	ID=S2009_raw:GT=hom:DP=13:FQ=1:SQ=-1;ID=S2503_raw:GT=hom:DP=52:FQ=1:SQ=-1;ID=S2507_raw:GT=hom:DP=60:FQ=1:SQ=-1;ID=S2668_raw:GT=hom:DP=59:FQ=1:SQ=-1;ID=S2012_raw:GT=hom:DP=4:FQ=1:SQ=-1;ID=S2010_raw:GT=hom:DP=13:FQ=1:SQ=-1;ID=S2662_raw:GT=hom:DP=6:FQ=1:SQ=-1;ID=S2663_raw:GT=hom:DP=31:FQ=1:SQ=-1;ID=S2011_raw:GT=hom:DP=16:FQ=1:SQ=-1;ID=S2504_raw:GT=hom:DP=51:FQ=1:SQ=-1;ID=S2008_raw:GT=hom:DP=15:FQ=1:SQ=-1;ID=S2509_raw:GT=hom:DP=30:FQ=1:SQ=-1;ID=S2508_raw:GT=hom:DP=47:FQ=1:SQ=-1;ID=S2666_raw:GT=hom:DP=34:FQ=1:SQ=-1;ID=S2669_raw:GT=hom:DP=35:FQ=1:SQ=-1;ID=S2166_raw:GT=hom:DP=23:FQ=1:SQ=-1;ID=S1775_raw:GT=hom:DP=25:FQ=1:SQ=-1;ID=S2501_raw:GT=hom:DP=40:FQ=1:SQ=-1;ID=S2500_raw:GT=hom:DP=50:FQ=1:SQ=-1;ID=S2502_raw:GT=hom:DP=55:FQ=1:SQ=-1;ID=S2505_raw:GT=hom:DP=68:FQ=1:SQ=-1;ID=S2176_raw:GT=hom:DP=38:FQ=1:SQ=-1;ID=S2506_raw:GT=hom:DP=37:FQ=1:SQ=-1;ID=S2667_raw:GT=hom:DP=33:FQ=1:SQ=-1;
1	2,04E+08	2,04E+08	T	C	exonic	PPP1R15B	nonsynonymous SNV	PPP1R15B:NM_032833:exon1:c.923A>G:p.N308S	rs3014626	.	ID=S2009_raw:GT=hom:DP=129:FQ=1:SQ=-1;ID=S2503_raw:GT=hom:DP=159:FQ=1:SQ=-1;ID=S2507_raw:GT=hom:DP=141:FQ=1:SQ=-1;ID=S2668_raw:GT=hom:DP=196:FQ=1:SQ=-1;ID=S2012_raw:GT=hom:DP=177:FQ=1:SQ=-1;ID=S2010_raw:GT=hom:DP=188:FQ=1:SQ=-1;ID=S2662_raw:GT=hom:DP=147:FQ=0.99:SQ=-1;ID=S2663_raw:GT=hom:DP=115:FQ=1:SQ=-1;ID=S2011_raw:GT=hom:DP=262:FQ=1:SQ=-1;ID=S2504_raw:GT=hom:DP=162:FQ=1:SQ=-1;ID=S2008_raw:GT=hom:DP=204:FQ=1:SQ=-1;ID=S2509_raw:GT=hom:DP=121:FQ=1:SQ=-1;ID=S2508_raw:GT=hom:DP=139:FQ=1:SQ=-1;ID=S2666_raw:GT=hom:DP=93:FQ=1:SQ=-1;ID=S2669_raw:GT=hom:DP=135:FQ=1:SQ=-1;ID=S2166_raw:GT=hom:DP=130:FQ=1:SQ=-1;ID=S1775_raw:GT=hom:DP=87:FQ=1:SQ=-1;ID=S2501_raw:GT=hom:DP=136:FQ=1:SQ=-1;ID=S2500_raw:GT=hom:DP=160:FQ=1:SQ=-1;ID=S2502_raw:GT=hom:DP=183:FQ=1:SQ=-1;ID=S2505_raw:GT=hom:DP=141:FQ=1:SQ=-1;ID=S2176_raw:GT=hom:DP=134:FQ=1:SQ=-1;ID=S2506_raw:GT=hom:DP=92:FQ=1:SQ=-1;ID=S2667_raw:GT=hom:DP=126:FQ=1:SQ=-1;
1	3310931	3310931	-	GC	intronic	PRDM16	.	.	.	.	ID=S2009_raw:GT=hom:DP=3:FQ=1:SQ=-1;ID=S2503_raw:GT=hom:DP=10:FQ=1:SQ=-1;ID=S2507_raw:GT=hom:DP=6:FQ=1:SQ=-1;ID=S2668_raw:GT=hom:DP=9:FQ=1:SQ=-1;ID=S2012_raw:GT=hom:DP=5:FQ=1:SQ=-1;ID=S2010_raw:GT=hom:DP=5:FQ=1:SQ=-1;ID=S2662_raw:GT=hom:DP=22:FQ=1:SQ=-1;ID=S2663_raw:GT=hom:DP=9:FQ=1:SQ=-1;ID=S2011_raw:GT=hom:DP=3:FQ=1:SQ=-1;ID=S2504_raw:GT=hom:DP=5:FQ=1:SQ=-1;ID=S2008_raw:GT=hom:DP=4:FQ=1:SQ=-1;ID=S2509_raw:GT=hom:DP=9:FQ=1:SQ=-1;ID=S2508_raw:GT=hom:DP=8:FQ=1:SQ=-1;ID=S2666_raw:GT=hom:DP=7:FQ=1:SQ=-1;ID=S2669_raw:GT=hom:DP=7:FQ=1:SQ=-1;ID=S2166_raw:GT=hom:DP=8:FQ=1:SQ=-1;ID=S1775_raw:GT=hom:DP=3:FQ=1:SQ=-1;ID=S2501_raw:GT=hom:DP=6:FQ=1:SQ=-1;ID=S2500_raw:GT=hom:DP=6:FQ=1:SQ=-1;ID=S2502_raw:GT=hom:DP=3:FQ=1:SQ=-1;ID=S2505_raw:GT=hom:DP=7:FQ=1:SQ=-1;ID=S2176_raw:GT=hom:DP=8:FQ=1:SQ=-1;ID=S2506_raw:GT=hom:DP=2:FQ=1:SQ=-1;ID=S2667_raw:GT=hom:DP=14:FQ=1:SQ=-1;
3	53216051	53216051	T	C	intronic	PRKCD	.	.	rs6445568	.	ID=S2009_raw:GT=hom:DP=55:FQ=1:SQ=-1;ID=S2503_raw:GT=hom:DP=12:FQ=1:SQ=-1;ID=S2507_raw:GT=hom:DP=15:FQ=1:SQ=-1;ID=S2668_raw:GT=hom:DP=8:FQ=1:SQ=-1;ID=S2012_raw:GT=hom:DP=51:FQ=1:SQ=-1;ID=S2010_raw:GT=hom:DP=67:FQ=1:SQ=-1;ID=S2662_raw:GT=hom:DP=20:FQ=1:SQ=-1;ID=S2663_raw:GT=hom:DP=8:FQ=1:SQ=-1;ID=S2011_raw:GT=hom:DP=73:FQ=1:SQ=-1;ID=S2504_raw:GT=hom:DP=13:FQ=1:SQ=-1;ID=S2008_raw:GT=hom:DP=53:FQ=1:SQ=-1;ID=S2509_raw:GT=hom:DP=13:FQ=1:SQ=-1;ID=S2508_raw:GT=hom:DP=13:FQ=1:SQ=-1;ID=S2666_raw:GT=hom:DP=14:FQ=1:SQ=-1;ID=S2669_raw:GT=hom:DP=7:FQ=1:SQ=-1;ID=S2166_raw:GT=hom:DP=19:FQ=1:SQ=-1;ID=S1775_raw:GT=hom:DP=7:FQ=1:SQ=-1;ID=S2501_raw:GT=hom:DP=11:FQ=1:SQ=-1;ID=S2500_raw:GT=hom:DP=9:FQ=1:SQ=-1;ID=S2502_raw:GT=hom:DP=19:FQ=1:SQ=-1;ID=S2505_raw:GT=hom:DP=19:FQ=1:SQ=-1;ID=S2176_raw:GT=hom:DP=6:FQ=1:SQ=-1;ID=S2506_raw:GT=hom:DP=21:FQ=1:SQ=-1;ID=S2667_raw:GT=hom:DP=10:FQ=1:SQ=-1;
3	53220444	53220444	T	C	intronic	PRKCD	.	.	rs6793352	.	ID=S2009_raw:GT=hom:DP=10:FQ=1:SQ=-1;ID=S2503_raw:GT=hom:DP=38:FQ=1:SQ=-1;ID=S2507_raw:GT=hom:DP=37:FQ=1:SQ=-1;ID=S2668_raw:GT=hom:DP=20:FQ=1:SQ=-1;ID=S2012_raw:GT=hom:DP=8:FQ=1:SQ=-1;ID=S2010_raw:GT=hom:DP=10:FQ=1:SQ=-1;ID=S2662_raw:GT=hom:DP=30:FQ=1:SQ=-1;ID=S2663_raw:GT=hom:DP=19:FQ=1:SQ=-1;ID=S2011_raw:GT=hom:DP=18:FQ=1:SQ=-1;ID=S2504_raw:GT=hom:DP=33:FQ=1:SQ=-1;ID=S2008_raw:GT=hom:DP=9:FQ=1:SQ=-1;ID=S2509_raw:GT=hom:DP=33:FQ=1:SQ=-1;ID=S2508_raw:GT=hom:DP=37:FQ=1:SQ=-1;ID=S2666_raw:GT=hom:DP=18:FQ=1:SQ=-1;ID=S2669_raw:GT=hom:DP=22:FQ=1:SQ=-1;ID=S2166_raw:GT=hom:DP=40:FQ=1:SQ=-1;ID=S1775_raw:GT=hom:DP=19:FQ=1:SQ=-1;ID=S2501_raw:GT=hom:DP=37:FQ=1:SQ=-1;ID=S2500_raw:GT=hom:DP=28:FQ=1:SQ=-1;ID=S2502_raw:GT=hom:DP=47:FQ=1:SQ=-1;ID=S2505_raw:GT=hom:DP=38:FQ=1:SQ=-1;ID=S2176_raw:GT=hom:DP=60:FQ=1:SQ=-1;ID=S2506_raw:GT=hom:DP=29:FQ=1:SQ=-1;ID=S2667_raw:GT=hom:DP=27:FQ=1:SQ=-1;
3	53223291	53223291	T	G	intronic	PRKCD	.	.	rs7630600	.	ID=S2009_raw:GT=hom:DP=18:FQ=1:SQ=-1;ID=S2503_raw:GT=hom:DP=39:FQ=1:SQ=-1;ID=S2507_raw:GT=hom:DP=15:FQ=1:SQ=-1;ID=S2668_raw:GT=hom:DP=14:FQ=1:SQ=-1;ID=S2012_raw:GT=hom:DP=8:FQ=1:SQ=-1;ID=S2010_raw:GT=hom:DP=25:FQ=1:SQ=-1;ID=S2662_raw:GT=hom:DP=87:FQ=1:SQ=-1;ID=S2663_raw:GT=hom:DP=46:FQ=1:SQ=-1;ID=S2011_raw:GT=hom:DP=38:FQ=1:SQ=-1;ID=S2504_raw:GT=hom:DP=48:FQ=1:SQ=-1;ID=S2008_raw:GT=hom:DP=31:FQ=1:SQ=-1;ID=S2509_raw:GT=hom:DP=31:FQ=1:SQ=-1;ID=S2508_raw:GT=hom:DP=37:FQ=1:SQ=-1;ID=S2666_raw:GT=hom:DP=37:FQ=1:SQ=-1;ID=S2669_raw:GT=hom:DP=34:FQ=1:SQ=-1;ID=S2166_raw:GT=hom:DP=54:FQ=1:SQ=-1;ID=S1775_raw:GT=hom:DP=15:FQ=1:SQ=-1;ID=S2501_raw:GT=hom:DP=55:FQ=1:SQ=-1;ID=S2500_raw:GT=hom:DP=29:FQ=1:SQ=-1;ID=S2502_raw:GT=hom:DP=66:FQ=1:SQ=-1;ID=S2505_raw:GT=hom:DP=10:FQ=1:SQ=-1;ID=S2176_raw:GT=hom:DP=42:FQ=1:SQ=-1;ID=S2506_raw:GT=hom:DP=37:FQ=1:SQ=-1;ID=S2667_raw:GT=hom:DP=41:FQ=1:SQ=-1;
3	53223927	53223927	C	G	exonic	PRKCD	synonymous SNV	PRKCD:NM_001354676:exon17:c.1839C>G:p.T613T,PRKCD:NM_001354678:exon17:c.1830C>G:p.T610T,PRKCD:NM_212539:exon17:c.1782C>G:p.T594T,PRKCD:NM_001316327:exon18:c.1782C>G:p.T594T,PRKCD:NM_001354679:exon18:c.1782C>G:p.T594T,PRKCD:NM_006254:exon18:c.1782C>G:p.T594T,PRKCD:NM_001354680:exon19:c.1782C>G:p.T594T	rs1075865	Benign	ID=S2009_raw:GT=hom:DP=92:FQ=1:SQ=-1;ID=S2503_raw:GT=hom:DP=166:FQ=1:SQ=-1;ID=S2507_raw:GT=hom:DP=162:FQ=1:SQ=-1;ID=S2668_raw:GT=hom:DP=199:FQ=1:SQ=-1;ID=S2012_raw:GT=hom:DP=113:FQ=1:SQ=-1;ID=S2010_raw:GT=hom:DP=116:FQ=1:SQ=-1;ID=S2662_raw:GT=hom:DP=273:FQ=1:SQ=-1;ID=S2663_raw:GT=hom:DP=165:FQ=1:SQ=-1;ID=S2011_raw:GT=hom:DP=140:FQ=1:SQ=-1;ID=S2504_raw:GT=hom:DP=184:FQ=1:SQ=-1;ID=S2008_raw:GT=hom:DP=125:FQ=1:SQ=-1;ID=S2509_raw:GT=hom:DP=174:FQ=1:SQ=-1;ID=S2508_raw:GT=hom:DP=162:FQ=1:SQ=-1;ID=S2666_raw:GT=hom:DP=138:FQ=1:SQ=-1;ID=S2669_raw:GT=hom:DP=165:FQ=1:SQ=-1;ID=S2166_raw:GT=hom:DP=117:FQ=1:SQ=-1;ID=S1775_raw:GT=hom:DP=85:FQ=1:SQ=-1;ID=S2501_raw:GT=hom:DP=168:FQ=1:SQ=-1;ID=S2500_raw:GT=hom:DP=170:FQ=1:SQ=-1;ID=S2502_raw:GT=hom:DP=207:FQ=1:SQ=-1;ID=S2505_raw:GT=hom:DP=158:FQ=1:SQ=-1;ID=S2176_raw:GT=hom:DP=130:FQ=1:SQ=-1;ID=S2506_raw:GT=hom:DP=150:FQ=1:SQ=-1;ID=S2667_raw:GT=hom:DP=163:FQ=1:SQ=-1;
3	53224002	53224002	T	C	exonic	PRKCD	synonymous SNV	PRKCD:NM_001354676:exon17:c.1914T>C:p.P638P,PRKCD:NM_001354678:exon17:c.1905T>C:p.P635P,PRKCD:NM_212539:exon17:c.1857T>C:p.P619P,PRKCD:NM_001316327:exon18:c.1857T>C:p.P619P,PRKCD:NM_001354679:exon18:c.1857T>C:p.P619P,PRKCD:NM_006254:exon18:c.1857T>C:p.P619P,PRKCD:NM_001354680:exon19:c.1857T>C:p.P619P	rs900495	Benign	ID=S2009_raw:GT=hom:DP=95:FQ=1:SQ=-1;ID=S2503_raw:GT=hom:DP=149:FQ=1:SQ=-1;ID=S2507_raw:GT=hom:DP=136:FQ=1:SQ=-1;ID=S2668_raw:GT=hom:DP=205:FQ=1:SQ=-1;ID=S2012_raw:GT=hom:DP=109:FQ=1:SQ=-1;ID=S2010_raw:GT=hom:DP=104:FQ=1:SQ=-1;ID=S2662_raw:GT=hom:DP=255:FQ=1:SQ=-1;ID=S2663_raw:GT=hom:DP=164:FQ=1:SQ=-1;ID=S2011_raw:GT=hom:DP=148:FQ=1:SQ=-1;ID=S2504_raw:GT=hom:DP=162:FQ=1:SQ=-1;ID=S2008_raw:GT=hom:DP=115:FQ=1:SQ=-1;ID=S2509_raw:GT=hom:DP=157:FQ=1:SQ=-1;ID=S2508_raw:GT=hom:DP=154:FQ=1:SQ=-1;ID=S2666_raw:GT=hom:DP=209:FQ=1:SQ=-1;ID=S2669_raw:GT=hom:DP=130:FQ=1:SQ=-1;ID=S2166_raw:GT=hom:DP=115:FQ=1:SQ=-1;ID=S1775_raw:GT=hom:DP=81:FQ=1:SQ=-1;ID=S2501_raw:GT=hom:DP=165:FQ=1:SQ=-1;ID=S2500_raw:GT=hom:DP=160:FQ=1:SQ=-1;ID=S2502_raw:GT=hom:DP=202:FQ=1:SQ=-1;ID=S2505_raw:GT=hom:DP=146:FQ=1:SQ=-1;ID=S2176_raw:GT=hom:DP=112:FQ=1:SQ=-1;ID=S2506_raw:GT=hom:DP=146:FQ=1:SQ=-1;ID=S2667_raw:GT=hom:DP=160:FQ=1:SQ=-1;
19	11558240	11558240	T	C	intronic	PRKCSH	.	.	rs186375	Benign	ID=S2009_raw:GT=hom:DP=39:FQ=1:SQ=-1;ID=S2503_raw:GT=hom:DP=132:FQ=1:SQ=-1;ID=S2507_raw:GT=hom:DP=82:FQ=1:SQ=-1;ID=S2668_raw:GT=hom:DP=118:FQ=1:SQ=-1;ID=S2012_raw:GT=hom:DP=34:FQ=1:SQ=-1;ID=S2010_raw:GT=hom:DP=33:FQ=1:SQ=-1;ID=S2662_raw:GT=hom:DP=197:FQ=1:SQ=-1;ID=S2663_raw:GT=hom:DP=115:FQ=1:SQ=-1;ID=S2011_raw:GT=hom:DP=30:FQ=1:SQ=-1;ID=S2504_raw:GT=hom:DP=142:FQ=1:SQ=-1;ID=S2008_raw:GT=hom:DP=34:FQ=1:SQ=-1;ID=S2509_raw:GT=hom:DP=108:FQ=1:SQ=-1;ID=S2508_raw:GT=hom:DP=121:FQ=1:SQ=-1;ID=S2666_raw:GT=hom:DP=69:FQ=1:SQ=-1;ID=S2669_raw:GT=hom:DP=128:FQ=1:SQ=-1;ID=S2166_raw:GT=hom:DP=118:FQ=1:SQ=-1;ID=S1775_raw:GT=hom:DP=39:FQ=1:SQ=-1;ID=S2501_raw:GT=hom:DP=140:FQ=1:SQ=-1;ID=S2500_raw:GT=hom:DP=91:FQ=1:SQ=-1;ID=S2502_raw:GT=hom:DP=131:FQ=1:SQ=-1;ID=S2505_raw:GT=hom:DP=116:FQ=1:SQ=-1;ID=S2176_raw:GT=hom:DP=90:FQ=1:SQ=-1;ID=S2506_raw:GT=hom:DP=129:FQ=1:SQ=-1;ID=S2667_raw:GT=hom:DP=141:FQ=1:SQ=-1;
1	2106840	2106840	T	C	intronic	PRKCZ	.	.	rs12135175	.	ID=S2009_raw:GT=hom:DP=17:FQ=1:SQ=-1;ID=S2503_raw:GT=hom:DP=7:FQ=1:SQ=-1;ID=S2507_raw:GT=hom:DP=10:FQ=1:SQ=-1;ID=S2668_raw:GT=hom:DP=8:FQ=1:SQ=-1;ID=S2012_raw:GT=hom:DP=17:FQ=1:SQ=-1;ID=S2010_raw:GT=hom:DP=22:FQ=1:SQ=-1;ID=S2662_raw:GT=hom:DP=21:FQ=1:SQ=-1;ID=S2663_raw:GT=hom:DP=12:FQ=1:SQ=-1;ID=S2011_raw:GT=hom:DP=17:FQ=1:SQ=-1;ID=S2504_raw:GT=hom:DP=9:FQ=1:SQ=-1;ID=S2008_raw:GT=hom:DP=23:FQ=1:SQ=-1;ID=S2509_raw:GT=hom:DP=11:FQ=1:SQ=-1;ID=S2508_raw:GT=hom:DP=11:FQ=1:SQ=-1;ID=S2666_raw:GT=hom:DP=5:FQ=1:SQ=-1;ID=S2669_raw:GT=hom:DP=7:FQ=1:SQ=-1;ID=S2166_raw:GT=hom:DP=16:FQ=1:SQ=-1;ID=S1775_raw:GT=hom:DP=9:FQ=1:SQ=-1;ID=S2501_raw:GT=hom:DP=13:FQ=1:SQ=-1;ID=S2500_raw:GT=hom:DP=6:FQ=1:SQ=-1;ID=S2502_raw:GT=hom:DP=9:FQ=1:SQ=-1;ID=S2505_raw:GT=hom:DP=9:FQ=1:SQ=-1;ID=S2176_raw:GT=hom:DP=13:FQ=1:SQ=-1;ID=S2506_raw:GT=hom:DP=10:FQ=1:SQ=-1;ID=S2667_raw:GT=hom:DP=7:FQ=1:SQ=-1;
14	90723109	90723109	T	G	intronic	PSMC1	.	.	rs6575119	.	ID=S2009_raw:GT=hom:DP=26:FQ=1:SQ=-1;ID=S2503_raw:GT=hom:DP=19:FQ=1:SQ=-1;ID=S2507_raw:GT=hom:DP=28:FQ=1:SQ=-1;ID=S2668_raw:GT=hom:DP=17:FQ=1:SQ=-1;ID=S2012_raw:GT=hom:DP=31:FQ=1:SQ=-1;ID=S2010_raw:GT=hom:DP=35:FQ=1:SQ=-1;ID=S2662_raw:GT=hom:DP=30:FQ=1:SQ=-1;ID=S2663_raw:GT=hom:DP=21:FQ=1:SQ=-1;ID=S2011_raw:GT=hom:DP=36:FQ=1:SQ=-1;ID=S2504_raw:GT=hom:DP=10:FQ=1:SQ=-1;ID=S2008_raw:GT=hom:DP=29:FQ=1:SQ=-1;ID=S2509_raw:GT=hom:DP=14:FQ=1:SQ=-1;ID=S2508_raw:GT=hom:DP=18:FQ=1:SQ=-1;ID=S2666_raw:GT=hom:DP=6:FQ=1:SQ=-1;ID=S2669_raw:GT=hom:DP=21:FQ=1:SQ=-1;ID=S2166_raw:GT=hom:DP=17:FQ=1:SQ=-1;ID=S1775_raw:GT=hom:DP=14:FQ=1:SQ=-1;ID=S2501_raw:GT=hom:DP=24:FQ=1:SQ=-1;ID=S2500_raw:GT=hom:DP=28:FQ=1:SQ=-1;ID=S2502_raw:GT=hom:DP=21:FQ=1:SQ=-1;ID=S2505_raw:GT=hom:DP=36:FQ=1:SQ=-1;ID=S2176_raw:GT=hom:DP=17:FQ=1:SQ=-1;ID=S2506_raw:GT=hom:DP=21:FQ=1:SQ=-1;ID=S2667_raw:GT=hom:DP=25:FQ=1:SQ=-1;
12	15731724	15731724	-	G	intronic	PTPRO	.	.	rs5796639	.	ID=S2009_raw:GT=hom:DP=52:FQ=1:SQ=-1;ID=S2503_raw:GT=hom:DP=32:FQ=1:SQ=-1;ID=S2507_raw:GT=hom:DP=26:FQ=1:SQ=-1;ID=S2668_raw:GT=hom:DP=34:FQ=1:SQ=-1;ID=S2012_raw:GT=hom:DP=69:FQ=1:SQ=-1;ID=S2010_raw:GT=hom:DP=80:FQ=1:SQ=-1;ID=S2662_raw:GT=hom:DP=3:FQ=1:SQ=-1;ID=S2663_raw:GT=hom:DP=30:FQ=1:SQ=-1;ID=S2011_raw:GT=hom:DP=104:FQ=1:SQ=-1;ID=S2504_raw:GT=hom:DP=33:FQ=1:SQ=-1;ID=S2008_raw:GT=hom:DP=67:FQ=1:SQ=-1;ID=S2509_raw:GT=hom:DP=25:FQ=1:SQ=-1;ID=S2508_raw:GT=hom:DP=41:FQ=1:SQ=-1;ID=S2666_raw:GT=hom:DP=46:FQ=1:SQ=-1;ID=S2669_raw:GT=hom:DP=12:FQ=1:SQ=-1;ID=S2166_raw:GT=hom:DP=105:FQ=1:SQ=-1;ID=S1775_raw:GT=hom:DP=59:FQ=1:SQ=-1;ID=S2501_raw:GT=hom:DP=33:FQ=1:SQ=-1;ID=S2500_raw:GT=hom:DP=21:FQ=1:SQ=-1;ID=S2502_raw:GT=hom:DP=35:FQ=1:SQ=-1;ID=S2505_raw:GT=hom:DP=25:FQ=1:SQ=-1;ID=S2176_raw:GT=hom:DP=101:FQ=1:SQ=-1;ID=S2506_raw:GT=hom:DP=26:FQ=1:SQ=-1;ID=S2667_raw:GT=hom:DP=26:FQ=1:SQ=-1;
14	51382358	51382358	T	C	intronic	PYGL	.	.	rs1890701	.	ID=S2009_raw:GT=hom:DP=60:FQ=1:SQ=-1;ID=S2503_raw:GT=hom:DP=24:FQ=1:SQ=-1;ID=S2507_raw:GT=hom:DP=19:FQ=1:SQ=-1;ID=S2668_raw:GT=hom:DP=28:FQ=1:SQ=-1;ID=S2012_raw:GT=hom:DP=74:FQ=1:SQ=-1;ID=S2010_raw:GT=hom:DP=88:FQ=1:SQ=-1;ID=S2662_raw:GT=hom:DP=6:FQ=1:SQ=-1;ID=S2663_raw:GT=hom:DP=21:FQ=1:SQ=-1;ID=S2011_raw:GT=hom:DP=123:FQ=1:SQ=-1;ID=S2504_raw:GT=hom:DP=31:FQ=1:SQ=-1;ID=S2008_raw:GT=hom:DP=92:FQ=1:SQ=-1;ID=S2509_raw:GT=hom:DP=28:FQ=1:SQ=-1;ID=S2508_raw:GT=hom:DP=19:FQ=1:SQ=-1;ID=S2666_raw:GT=hom:DP=19:FQ=1:SQ=-1;ID=S2669_raw:GT=hom:DP=16:FQ=1:SQ=-1;ID=S2166_raw:GT=hom:DP=52:FQ=1:SQ=-1;ID=S1775_raw:GT=hom:DP=21:FQ=1:SQ=-1;ID=S2501_raw:GT=hom:DP=19:FQ=1:SQ=-1;ID=S2500_raw:GT=hom:DP=28:FQ=1:SQ=-1;ID=S2502_raw:GT=hom:DP=33:FQ=1:SQ=-1;ID=S2505_raw:GT=hom:DP=25:FQ=1:SQ=-1;ID=S2176_raw:GT=hom:DP=41:FQ=1:SQ=-1;ID=S2506_raw:GT=hom:DP=33:FQ=1:SQ=-1;ID=S2667_raw:GT=hom:DP=14:FQ=1:SQ=-1;
11	64520374	64520374	T	A	intronic	PYGM	.	.	rs565688	.	ID=S2009_raw:GT=hom:DP=6:FQ=1:SQ=-1;ID=S2503_raw:GT=hom:DP=6:FQ=1:SQ=-1;ID=S2507_raw:GT=hom:DP=2:FQ=1:SQ=-1;ID=S2668_raw:GT=hom:DP=3:FQ=1:SQ=-1;ID=S2012_raw:GT=hom:DP=6:FQ=1:SQ=-1;ID=S2010_raw:GT=hom:DP=5:FQ=1:SQ=-1;ID=S2662_raw:GT=hom:DP=3:FQ=1:SQ=-1;ID=S2663_raw:GT=hom:DP=3:FQ=1:SQ=-1;ID=S2011_raw:GT=hom:DP=3:FQ=1:SQ=-1;ID=S2504_raw:GT=hom:DP=6:FQ=1:SQ=-1;ID=S2008_raw:GT=hom:DP=5:FQ=1:SQ=-1;ID=S2509_raw:GT=hom:DP=6:FQ=1:SQ=-1;ID=S2508_raw:GT=hom:DP=3:FQ=1:SQ=-1;ID=S2666_raw:GT=hom:DP=2:FQ=1:SQ=-1;ID=S2669_raw:GT=hom:DP=2:FQ=1:SQ=-1;ID=S2166_raw:GT=hom:DP=18:FQ=1:SQ=-1;ID=S1775_raw:GT=hom:DP=5:FQ=1:SQ=-1;ID=S2501_raw:GT=hom:DP=1:FQ=1:SQ=-1;ID=S2500_raw:GT=hom:DP=8:FQ=1:SQ=-1;ID=S2502_raw:GT=hom:DP=14:FQ=1:SQ=-1;ID=S2505_raw:GT=hom:DP=5:FQ=1:SQ=-1;ID=S2176_raw:GT=hom:DP=15:FQ=1:SQ=-1;ID=S2506_raw:GT=hom:DP=4:FQ=1:SQ=-1;ID=S2667_raw:GT=hom:DP=7:FQ=1:SQ=-1;
3	12650482	12650482	T	A	intronic	RAF1	.	.	rs2454440	.	ID=S2009_raw:GT=hom:DP=24:FQ=1:SQ=-1;ID=S2503_raw:GT=hom:DP=57:FQ=1:SQ=-1;ID=S2507_raw:GT=hom:DP=44:FQ=1:SQ=-1;ID=S2668_raw:GT=hom:DP=40:FQ=1:SQ=-1;ID=S2012_raw:GT=hom:DP=20:FQ=1:SQ=-1;ID=S2010_raw:GT=hom:DP=35:FQ=1:SQ=-1;ID=S2662_raw:GT=hom:DP=8:FQ=1:SQ=-1;ID=S2663_raw:GT=hom:DP=41:FQ=1:SQ=-1;ID=S2011_raw:GT=hom:DP=29:FQ=1:SQ=-1;ID=S2504_raw:GT=hom:DP=46:FQ=1:SQ=-1;ID=S2008_raw:GT=hom:DP=23:FQ=1:SQ=-1;ID=S2509_raw:GT=hom:DP=51:FQ=1:SQ=-1;ID=S2508_raw:GT=hom:DP=45:FQ=1:SQ=-1;ID=S2666_raw:GT=hom:DP=45:FQ=1:SQ=-1;ID=S2669_raw:GT=hom:DP=31:FQ=1:SQ=-1;ID=S2166_raw:GT=hom:DP=62:FQ=1:SQ=-1;ID=S1775_raw:GT=hom:DP=59:FQ=1:SQ=-1;ID=S2501_raw:GT=hom:DP=45:FQ=1:SQ=-1;ID=S2500_raw:GT=hom:DP=44:FQ=1:SQ=-1;ID=S2502_raw:GT=hom:DP=59:FQ=1:SQ=-1;ID=S2505_raw:GT=hom:DP=52:FQ=1:SQ=-1;ID=S2176_raw:GT=hom:DP=69:FQ=1:SQ=-1;ID=S2506_raw:GT=hom:DP=51:FQ=1:SQ=-1;ID=S2667_raw:GT=hom:DP=52:FQ=1:SQ=-1;
8	1,46E+08	1,46E+08	A	G	exonic	RECQL4	synonymous SNV	RECQL4:NM_004260:exon19:c.3127T>C:p.L1043L	rs4925828	Benign	ID=S2009_raw:GT=hom:DP=126:FQ=1:SQ=-1;ID=S2503_raw:GT=hom:DP=276:FQ=1:SQ=-1;ID=S2507_raw:GT=hom:DP=342:FQ=1:SQ=-1;ID=S2668_raw:GT=hom:DP=343:FQ=1:SQ=-1;ID=S2012_raw:GT=hom:DP=118:FQ=1:SQ=-1;ID=S2010_raw:GT=hom:DP=86:FQ=1:SQ=-1;ID=S2662_raw:GT=hom:DP=656:FQ=1:SQ=-1;ID=S2663_raw:GT=hom:DP=318:FQ=1:SQ=-1;ID=S2011_raw:GT=hom:DP=109:FQ=1:SQ=-1;ID=S2504_raw:GT=hom:DP=332:FQ=1:SQ=-1;ID=S2008_raw:GT=hom:DP=104:FQ=1:SQ=-1;ID=S2509_raw:GT=hom:DP=392:FQ=1:SQ=-1;ID=S2508_raw:GT=hom:DP=428:FQ=1:SQ=-1;ID=S2666_raw:GT=hom:DP=233:FQ=1:SQ=-1;ID=S2669_raw:GT=hom:DP=392:FQ=1:SQ=-1;ID=S2166_raw:GT=hom:DP=228:FQ=1:SQ=-1;ID=S1775_raw:GT=hom:DP=160:FQ=0.99:SQ=-1;ID=S2501_raw:GT=hom:DP=348:FQ=1:SQ=-1;ID=S2500_raw:GT=hom:DP=338:FQ=1:SQ=-1;ID=S2502_raw:GT=hom:DP=346:FQ=1:SQ=-1;ID=S2505_raw:GT=hom:DP=339:FQ=1:SQ=-1;ID=S2176_raw:GT=hom:DP=228:FQ=1:SQ=-1;ID=S2506_raw:GT=hom:DP=428:FQ=1:SQ=-1;ID=S2667_raw:GT=hom:DP=354:FQ=1:SQ=-1;
8	1,46E+08	1,46E+08	G	-	exonic;splicing	RECQL4	frameshift deletion	RECQL4:NM_004260:exon14:c.2296delC:p.R766fs	.	.	ID=S2009_raw:GT=hom:DP=135:FQ=1:SQ=-1;ID=S2503_raw:GT=hom:DP=146:FQ=1:SQ=-1;ID=S2507_raw:GT=hom:DP=162:FQ=1:SQ=-1;ID=S2668_raw:GT=hom:DP=176:FQ=1:SQ=-1;ID=S2012_raw:GT=hom:DP=110:FQ=1:SQ=-1;ID=S2010_raw:GT=hom:DP=116:FQ=1:SQ=-1;ID=S2662_raw:GT=hom:DP=290:FQ=1:SQ=-1;ID=S2663_raw:GT=hom:DP=152:FQ=1:SQ=-1;ID=S2011_raw:GT=hom:DP=185:FQ=1:SQ=-1;ID=S2504_raw:GT=hom:DP=151:FQ=1:SQ=-1;ID=S2008_raw:GT=hom:DP=157:FQ=1:SQ=-1;ID=S2509_raw:GT=hom:DP=169:FQ=1:SQ=-1;ID=S2508_raw:GT=hom:DP=184:FQ=1:SQ=-1;ID=S2666_raw:GT=hom:DP=162:FQ=1:SQ=-1;ID=S2669_raw:GT=hom:DP=175:FQ=1:SQ=-1;ID=S2166_raw:GT=hom:DP=117:FQ=1:SQ=-1;ID=S1775_raw:GT=hom:DP=95:FQ=1:SQ=-1;ID=S2501_raw:GT=hom:DP=169:FQ=1:SQ=-1;ID=S2500_raw:GT=hom:DP=139:FQ=1:SQ=-1;ID=S2502_raw:GT=hom:DP=184:FQ=1:SQ=-1;ID=S2505_raw:GT=hom:DP=147:FQ=1:SQ=-1;ID=S2176_raw:GT=hom:DP=162:FQ=1:SQ=-1;ID=S2506_raw:GT=hom:DP=199:FQ=1:SQ=-1;ID=S2667_raw:GT=hom:DP=183:FQ=1:SQ=-1;
8	1,46E+08	1,46E+08	A	G	exonic	RECQL4	nonsynonymous SNV	RECQL4:NM_004260:exon4:c.274T>C:p.S92P	rs2721190	Benign	ID=S2009_raw:GT=hom:DP=80:FQ=1:SQ=-1;ID=S2503_raw:GT=hom:DP=93:FQ=1:SQ=-1;ID=S2507_raw:GT=hom:DP=102:FQ=1:SQ=-1;ID=S2668_raw:GT=hom:DP=90:FQ=1:SQ=-1;ID=S2012_raw:GT=hom:DP=55:FQ=1:SQ=-1;ID=S2010_raw:GT=hom:DP=59:FQ=1:SQ=-1;ID=S2662_raw:GT=hom:DP=205:FQ=1:SQ=-1;ID=S2663_raw:GT=hom:DP=84:FQ=1:SQ=-1;ID=S2011_raw:GT=hom:DP=74:FQ=1:SQ=-1;ID=S2504_raw:GT=hom:DP=100:FQ=1:SQ=-1;ID=S2008_raw:GT=hom:DP=83:FQ=1:SQ=-1;ID=S2509_raw:GT=hom:DP=98:FQ=1:SQ=-1;ID=S2508_raw:GT=hom:DP=112:FQ=1:SQ=-1;ID=S2666_raw:GT=hom:DP=56:FQ=1:SQ=-1;ID=S2669_raw:GT=hom:DP=85:FQ=1:SQ=-1;ID=S2166_raw:GT=hom:DP=83:FQ=1:SQ=-1;ID=S1775_raw:GT=hom:DP=58:FQ=1:SQ=-1;ID=S2501_raw:GT=hom:DP=101:FQ=1:SQ=-1;ID=S2500_raw:GT=hom:DP=66:FQ=1:SQ=-1;ID=S2502_raw:GT=hom:DP=83:FQ=1:SQ=-1;ID=S2505_raw:GT=hom:DP=100:FQ=1:SQ=-1;ID=S2176_raw:GT=hom:DP=80:FQ=1:SQ=-1;ID=S2506_raw:GT=hom:DP=114:FQ=1:SQ=-1;ID=S2667_raw:GT=hom:DP=137:FQ=1:SQ=-1;
8	1,46E+08	1,46E+08	C	A	intronic	RECQL4	.	.	rs2721189	.	ID=S2009_raw:GT=hom:DP=8:FQ=1:SQ=-1;ID=S2503_raw:GT=hom:DP=20:FQ=0.95:SQ=-1;ID=S2507_raw:GT=hom:DP=22:FQ=1:SQ=-1;ID=S2668_raw:GT=hom:DP=11:FQ=1:SQ=-1;ID=S2012_raw:GT=hom:DP=10:FQ=1:SQ=-1;ID=S2010_raw:GT=hom:DP=6:FQ=1:SQ=-1;ID=S2662_raw:GT=hom:DP=32:FQ=1:SQ=-1;ID=S2663_raw:GT=hom:DP=15:FQ=1:SQ=-1;ID=S2011_raw:GT=hom:DP=13:FQ=1:SQ=-1;ID=S2504_raw:GT=hom:DP=12:FQ=1:SQ=-1;ID=S2008_raw:GT=hom:DP=12:FQ=1:SQ=-1;ID=S2509_raw:GT=hom:DP=21:FQ=1:SQ=-1;ID=S2508_raw:GT=hom:DP=16:FQ=1:SQ=-1;ID=S2666_raw:GT=hom:DP=8:FQ=1:SQ=-1;ID=S2669_raw:GT=hom:DP=19:FQ=1:SQ=-1;ID=S2166_raw:GT=hom:DP=30:FQ=1:SQ=-1;ID=S1775_raw:GT=hom:DP=9:FQ=1:SQ=-1;ID=S2501_raw:GT=hom:DP=16:FQ=1:SQ=-1;ID=S2500_raw:GT=hom:DP=11:FQ=1:SQ=-1;ID=S2502_raw:GT=hom:DP=20:FQ=1:SQ=-1;ID=S2505_raw:GT=hom:DP=12:FQ=1:SQ=-1;ID=S2176_raw:GT=hom:DP=17:FQ=1:SQ=-1;ID=S2506_raw:GT=hom:DP=27:FQ=1:SQ=-1;ID=S2667_raw:GT=hom:DP=29:FQ=1:SQ=-1;
15	56386577	56386577	T	G	exonic	RFX7	nonsynonymous SNV	RFX7:NM_001368073:exon8:c.3058A>C:p.N1020H,RFX7:NM_001368074:exon9:c.3058A>C:p.N1020H,RFX7:NM_022841:exon9:c.3349A>C:p.N1117H	rs7170589	.	ID=S2009_raw:GT=hom:DP=62:FQ=1:SQ=-1;ID=S2503_raw:GT=hom:DP=103:FQ=1:SQ=-1;ID=S2507_raw:GT=hom:DP=89:FQ=1:SQ=-1;ID=S2668_raw:GT=hom:DP=143:FQ=1:SQ=-1;ID=S2012_raw:GT=hom:DP=89:FQ=1:SQ=-1;ID=S2010_raw:GT=hom:DP=83:FQ=1:SQ=-1;ID=S2662_raw:GT=hom:DP=35:FQ=1:SQ=-1;ID=S2663_raw:GT=hom:DP=105:FQ=1:SQ=-1;ID=S2011_raw:GT=hom:DP=103:FQ=1:SQ=-1;ID=S2504_raw:GT=hom:DP=116:FQ=1:SQ=-1;ID=S2008_raw:GT=hom:DP=96:FQ=1:SQ=-1;ID=S2509_raw:GT=hom:DP=67:FQ=1:SQ=-1;ID=S2508_raw:GT=hom:DP=114:FQ=1:SQ=-1;ID=S2666_raw:GT=hom:DP=125:FQ=1:SQ=-1;ID=S2669_raw:GT=hom:DP=78:FQ=1:SQ=-1;ID=S2166_raw:GT=hom:DP=206:FQ=1:SQ=-1;ID=S1775_raw:GT=hom:DP=132:FQ=1:SQ=-1;ID=S2501_raw:GT=hom:DP=93:FQ=1:SQ=-1;ID=S2500_raw:GT=hom:DP=105:FQ=1:SQ=-1;ID=S2502_raw:GT=hom:DP=120:FQ=1:SQ=-1;ID=S2505_raw:GT=hom:DP=105:FQ=1:SQ=-1;ID=S2176_raw:GT=hom:DP=207:FQ=1:SQ=-1;ID=S2506_raw:GT=hom:DP=82:FQ=1:SQ=-1;ID=S2667_raw:GT=hom:DP=115:FQ=1:SQ=-1;
15	56387931	56387931	C	T	exonic	RFX7	synonymous SNV	RFX7:NM_001368073:exon8:c.1704G>A:p.Q568Q,RFX7:NM_001368074:exon9:c.1704G>A:p.Q568Q,RFX7:NM_022841:exon9:c.1995G>A:p.Q665Q	rs11071247	.	ID=S2009_raw:GT=hom:DP=115:FQ=1:SQ=-1;ID=S2503_raw:GT=hom:DP=111:FQ=1:SQ=-1;ID=S2507_raw:GT=hom:DP=100:FQ=1:SQ=-1;ID=S2668_raw:GT=hom:DP=119:FQ=1:SQ=-1;ID=S2012_raw:GT=hom:DP=129:FQ=1:SQ=-1;ID=S2010_raw:GT=hom:DP=152:FQ=1:SQ=-1;ID=S2662_raw:GT=hom:DP=40:FQ=1:SQ=-1;ID=S2663_raw:GT=hom:DP=115:FQ=1:SQ=-1;ID=S2011_raw:GT=hom:DP=231:FQ=1:SQ=-1;ID=S2504_raw:GT=hom:DP=117:FQ=1:SQ=-1;ID=S2008_raw:GT=hom:DP=154:FQ=1:SQ=-1;ID=S2509_raw:GT=hom:DP=94:FQ=1:SQ=-1;ID=S2508_raw:GT=hom:DP=119:FQ=1:SQ=-1;ID=S2666_raw:GT=hom:DP=97:FQ=1:SQ=-1;ID=S2669_raw:GT=hom:DP=89:FQ=1:SQ=-1;ID=S2166_raw:GT=hom:DP=340:FQ=1:SQ=-1;ID=S1775_raw:GT=hom:DP=304:FQ=1:SQ=-1;ID=S2501_raw:GT=hom:DP=86:FQ=1:SQ=-1;ID=S2500_raw:GT=hom:DP=113:FQ=1:SQ=-1;ID=S2502_raw:GT=hom:DP=129:FQ=1:SQ=-1;ID=S2505_raw:GT=hom:DP=113:FQ=1:SQ=-1;ID=S2176_raw:GT=hom:DP=378:FQ=1:SQ=-1;ID=S2506_raw:GT=hom:DP=119:FQ=1:SQ=-1;ID=S2667_raw:GT=hom:DP=98:FQ=1:SQ=-1;
9	94499964	94499964	T	C	intronic	ROR2	.	.	rs9409459	.	ID=S2009_raw:GT=hom:DP=9:FQ=1:SQ=-1;ID=S2503_raw:GT=hom:DP=5:FQ=1:SQ=-1;ID=S2507_raw:GT=hom:DP=5:FQ=1:SQ=-1;ID=S2668_raw:GT=hom:DP=1:FQ=1:SQ=-1;ID=S2012_raw:GT=hom:DP=17:FQ=1:SQ=-1;ID=S2010_raw:GT=hom:DP=13:FQ=1:SQ=-1;ID=S2662_raw:GT=hom:DP=1:FQ=1:SQ=-1;ID=S2663_raw:GT=hom:DP=1:FQ=1:SQ=-1;ID=S2011_raw:GT=hom:DP=17:FQ=1:SQ=-1;ID=S2504_raw:GT=hom:DP=6:FQ=1:SQ=-1;ID=S2008_raw:GT=hom:DP=11:FQ=1:SQ=-1;ID=S2509_raw:GT=hom:DP=4:FQ=1:SQ=-1;ID=S2508_raw:GT=hom:DP=3:FQ=1:SQ=-1;ID=S2666_raw:GT=hom:DP=2:FQ=1:SQ=-1;ID=S2669_raw:GT=hom:DP=4:FQ=1:SQ=-1;ID=S2166_raw:GT=hom:DP=9:FQ=1:SQ=-1;ID=S1775_raw:GT=hom:DP=4:FQ=1:SQ=-1;ID=S2501_raw:GT=hom:DP=2:FQ=1:SQ=-1;ID=S2500_raw:GT=hom:DP=8:FQ=1:SQ=-1;ID=S2502_raw:GT=hom:DP=6:FQ=1:SQ=-1;ID=S2505_raw:GT=hom:DP=4:FQ=1:SQ=-1;ID=S2176_raw:GT=hom:DP=5:FQ=1:SQ=-1;ID=S2506_raw:GT=hom:DP=2:FQ=1:SQ=-1;ID=S2667_raw:GT=hom:DP=2:FQ=1:SQ=-1;
15	60789798	60789798	G	T	exonic	RORA	synonymous SNV	RORA:NM_134262:exon10:c.1263C>A:p.T421T,RORA:NM_002943:exon11:c.1503C>A:p.T501T,RORA:NM_134261:exon11:c.1428C>A:p.T476T,RORA:NM_134260:exon12:c.1527C>A:p.T509T	rs11071539	.	ID=S2009_raw:GT=hom:DP=15:FQ=1:SQ=-1;ID=S2503_raw:GT=hom:DP=75:FQ=1:SQ=-1;ID=S2507_raw:GT=hom:DP=73:FQ=1:SQ=-1;ID=S2668_raw:GT=hom:DP=104:FQ=1:SQ=-1;ID=S2012_raw:GT=hom:DP=21:FQ=1:SQ=-1;ID=S2010_raw:GT=hom:DP=20:FQ=1:SQ=-1;ID=S2662_raw:GT=hom:DP=6:FQ=1:SQ=-1;ID=S2663_raw:GT=hom:DP=61:FQ=1:SQ=-1;ID=S2011_raw:GT=hom:DP=45:FQ=1:SQ=-1;ID=S2504_raw:GT=hom:DP=73:FQ=1:SQ=-1;ID=S2008_raw:GT=hom:DP=30:FQ=1:SQ=-1;ID=S2509_raw:GT=hom:DP=89:FQ=1:SQ=-1;ID=S2508_raw:GT=hom:DP=58:FQ=1:SQ=-1;ID=S2666_raw:GT=hom:DP=93:FQ=1:SQ=-1;ID=S2669_raw:GT=hom:DP=58:FQ=1:SQ=-1;ID=S2166_raw:GT=hom:DP=244:FQ=1:SQ=-1;ID=S1775_raw:GT=hom:DP=196:FQ=1:SQ=-1;ID=S2501_raw:GT=hom:DP=79:FQ=1:SQ=-1;ID=S2500_raw:GT=hom:DP=101:FQ=1:SQ=-1;ID=S2502_raw:GT=hom:DP=80:FQ=1:SQ=-1;ID=S2505_raw:GT=hom:DP=61:FQ=1:SQ=-1;ID=S2176_raw:GT=hom:DP=262:FQ=1:SQ=-1;ID=S2506_raw:GT=hom:DP=47:FQ=1:SQ=-1;ID=S2667_raw:GT=hom:DP=76:FQ=1:SQ=-1;
1	24018277	24018277	C	G	UTR5	RPL11	.	NM_001199802:c.-37C>G	rs10917413	Benign	ID=S2009_raw:GT=hom:DP=38:FQ=1:SQ=-1;ID=S2503_raw:GT=hom:DP=171:FQ=1:SQ=-1;ID=S2507_raw:GT=hom:DP=178:FQ=1:SQ=-1;ID=S2668_raw:GT=hom:DP=122:FQ=1:SQ=-1;ID=S2012_raw:GT=hom:DP=31:FQ=1:SQ=-1;ID=S2010_raw:GT=hom:DP=33:FQ=1:SQ=-1;ID=S2662_raw:GT=hom:DP=256:FQ=1:SQ=-1;ID=S2663_raw:GT=hom:DP=128:FQ=1:SQ=-1;ID=S2011_raw:GT=hom:DP=45:FQ=1:SQ=-1;ID=S2504_raw:GT=hom:DP=150:FQ=1:SQ=-1;ID=S2008_raw:GT=hom:DP=43:FQ=1:SQ=-1;ID=S2509_raw:GT=hom:DP=156:FQ=1:SQ=-1;ID=S2508_raw:GT=hom:DP=174:FQ=1:SQ=-1;ID=S2666_raw:GT=hom:DP=72:FQ=1:SQ=-1;ID=S2669_raw:GT=hom:DP=175:FQ=1:SQ=-1;ID=S2166_raw:GT=hom:DP=182:FQ=1:SQ=-1;ID=S1775_raw:GT=hom:DP=175:FQ=1:SQ=-1;ID=S2501_raw:GT=hom:DP=178:FQ=1:SQ=-1;ID=S2500_raw:GT=hom:DP=131:FQ=1:SQ=-1;ID=S2502_raw:GT=hom:DP=154:FQ=1:SQ=-1;ID=S2505_raw:GT=hom:DP=181:FQ=1:SQ=-1;ID=S2176_raw:GT=hom:DP=172:FQ=1:SQ=-1;ID=S2506_raw:GT=hom:DP=146:FQ=1:SQ=-1;ID=S2667_raw:GT=hom:DP=158:FQ=1:SQ=-1;
17	41150983	41150983	T	C	intronic	RPL27	.	.	rs691954	.	ID=S2009_raw:GT=hom:DP=22:FQ=1:SQ=-1;ID=S2503_raw:GT=hom:DP=89:FQ=1:SQ=-1;ID=S2507_raw:GT=hom:DP=69:FQ=1:SQ=-1;ID=S2668_raw:GT=hom:DP=75:FQ=1:SQ=-1;ID=S2012_raw:GT=hom:DP=19:FQ=1:SQ=-1;ID=S2010_raw:GT=hom:DP=20:FQ=1:SQ=-1;ID=S2662_raw:GT=hom:DP=108:FQ=1:SQ=-1;ID=S2663_raw:GT=hom:DP=95:FQ=1:SQ=-1;ID=S2011_raw:GT=hom:DP=26:FQ=1:SQ=-1;ID=S2504_raw:GT=hom:DP=106:FQ=1:SQ=-1;ID=S2008_raw:GT=hom:DP=14:FQ=1:SQ=-1;ID=S2509_raw:GT=hom:DP=52:FQ=0.94:SQ=-1;ID=S2508_raw:GT=hom:DP=79:FQ=1:SQ=-1;ID=S2666_raw:GT=hom:DP=53:FQ=1:SQ=-1;ID=S2669_raw:GT=hom:DP=105:FQ=1:SQ=-1;ID=S2166_raw:GT=hom:DP=54:FQ=1:SQ=-1;ID=S1775_raw:GT=hom:DP=46:FQ=1:SQ=-1;ID=S2501_raw:GT=hom:DP=75:FQ=1:SQ=-1;ID=S2500_raw:GT=hom:DP=92:FQ=1:SQ=-1;ID=S2502_raw:GT=hom:DP=89:FQ=1:SQ=-1;ID=S2505_raw:GT=hom:DP=95:FQ=1:SQ=-1;ID=S2176_raw:GT=hom:DP=86:FQ=1:SQ=-1;ID=S2506_raw:GT=hom:DP=92:FQ=1:SQ=-1;ID=S2667_raw:GT=hom:DP=99:FQ=1:SQ=-1;
12	56436472	56436472	T	G	intronic	RPS26	.	.	rs705706	.	ID=S2009_raw:GT=hom:DP=27:FQ=1:SQ=-1;ID=S2503_raw:GT=hom:DP=109:FQ=1:SQ=-1;ID=S2507_raw:GT=hom:DP=81:FQ=1:SQ=-1;ID=S2668_raw:GT=hom:DP=89:FQ=1:SQ=-1;ID=S2012_raw:GT=hom:DP=45:FQ=1:SQ=-1;ID=S2010_raw:GT=hom:DP=39:FQ=1:SQ=-1;ID=S2662_raw:GT=hom:DP=87:FQ=1:SQ=-1;ID=S2663_raw:GT=hom:DP=81:FQ=1:SQ=-1;ID=S2011_raw:GT=hom:DP=59:FQ=1:SQ=-1;ID=S2504_raw:GT=hom:DP=92:FQ=1:SQ=-1;ID=S2008_raw:GT=hom:DP=48:FQ=1:SQ=-1;ID=S2509_raw:GT=hom:DP=75:FQ=1:SQ=-1;ID=S2508_raw:GT=hom:DP=117:FQ=1:SQ=-1;ID=S2666_raw:GT=hom:DP=65:FQ=1:SQ=-1;ID=S2669_raw:GT=hom:DP=56:FQ=1:SQ=-1;ID=S2166_raw:GT=hom:DP=28:FQ=1:SQ=-1;ID=S1775_raw:GT=hom:DP=18:FQ=1:SQ=-1;ID=S2501_raw:GT=hom:DP=89:FQ=1:SQ=-1;ID=S2500_raw:GT=hom:DP=92:FQ=1:SQ=-1;ID=S2502_raw:GT=hom:DP=100:FQ=1:SQ=-1;ID=S2505_raw:GT=hom:DP=92:FQ=1:SQ=-1;ID=S2176_raw:GT=hom:DP=31:FQ=1:SQ=-1;ID=S2506_raw:GT=hom:DP=85:FQ=1:SQ=-1;ID=S2667_raw:GT=hom:DP=90:FQ=1:SQ=-1;
8	1,03E+08	1,03E+08	A	G	UTR3	RRM2B	.	NM_001172477:c.*10T>C;NM_001172478:c.*10T>C;NM_015713:c.*10T>C	rs1265117	.	ID=S2009_raw:GT=hom:DP=30:FQ=1:SQ=-1;ID=S2503_raw:GT=hom:DP=69:FQ=1:SQ=-1;ID=S2507_raw:GT=hom:DP=53:FQ=1:SQ=-1;ID=S2668_raw:GT=hom:DP=89:FQ=1:SQ=-1;ID=S2012_raw:GT=hom:DP=56:FQ=1:SQ=-1;ID=S2010_raw:GT=hom:DP=43:FQ=1:SQ=-1;ID=S2662_raw:GT=hom:DP=4:FQ=1:SQ=-1;ID=S2663_raw:GT=hom:DP=68:FQ=1:SQ=-1;ID=S2011_raw:GT=hom:DP=66:FQ=1:SQ=-1;ID=S2504_raw:GT=hom:DP=73:FQ=1:SQ=-1;ID=S2008_raw:GT=hom:DP=36:FQ=1:SQ=-1;ID=S2509_raw:GT=hom:DP=90:FQ=1:SQ=-1;ID=S2508_raw:GT=hom:DP=67:FQ=1:SQ=-1;ID=S2666_raw:GT=hom:DP=70:FQ=1:SQ=-1;ID=S2669_raw:GT=hom:DP=58:FQ=1:SQ=-1;ID=S2166_raw:GT=hom:DP=76:FQ=1:SQ=-1;ID=S1775_raw:GT=hom:DP=56:FQ=1:SQ=-1;ID=S2501_raw:GT=hom:DP=96:FQ=1:SQ=-1;ID=S2500_raw:GT=hom:DP=66:FQ=1:SQ=-1;ID=S2502_raw:GT=hom:DP=88:FQ=1:SQ=-1;ID=S2505_raw:GT=hom:DP=93:FQ=1:SQ=-1;ID=S2176_raw:GT=hom:DP=85:FQ=1:SQ=-1;ID=S2506_raw:GT=hom:DP=67:FQ=1:SQ=-1;ID=S2667_raw:GT=hom:DP=75:FQ=1:SQ=-1;
18	67860719	67860719	T	C	intronic	RTTN	.	.	rs283250	.	ID=S2009_raw:GT=hom:DP=19:FQ=1:SQ=-1;ID=S2503_raw:GT=hom:DP=38:FQ=1:SQ=-1;ID=S2507_raw:GT=hom:DP=31:FQ=1:SQ=-1;ID=S2668_raw:GT=hom:DP=37:FQ=1:SQ=-1;ID=S2012_raw:GT=hom:DP=24:FQ=1:SQ=-1;ID=S2010_raw:GT=hom:DP=28:FQ=1:SQ=-1;ID=S2662_raw:GT=hom:DP=2:FQ=1:SQ=-1;ID=S2663_raw:GT=hom:DP=31:FQ=1:SQ=-1;ID=S2011_raw:GT=hom:DP=43:FQ=1:SQ=-1;ID=S2504_raw:GT=hom:DP=34:FQ=1:SQ=-1;ID=S2008_raw:GT=hom:DP=28:FQ=1:SQ=-1;ID=S2509_raw:GT=hom:DP=45:FQ=1:SQ=-1;ID=S2508_raw:GT=hom:DP=34:FQ=1:SQ=-1;ID=S2666_raw:GT=hom:DP=23:FQ=1:SQ=-1;ID=S2669_raw:GT=hom:DP=16:FQ=1:SQ=-1;ID=S2166_raw:GT=hom:DP=27:FQ=1:SQ=-1;ID=S1775_raw:GT=hom:DP=18:FQ=1:SQ=-1;ID=S2501_raw:GT=hom:DP=25:FQ=1:SQ=-1;ID=S2500_raw:GT=hom:DP=25:FQ=1:SQ=-1;ID=S2502_raw:GT=hom:DP=28:FQ=1:SQ=-1;ID=S2505_raw:GT=hom:DP=29:FQ=1:SQ=-1;ID=S2176_raw:GT=hom:DP=27:FQ=1:SQ=-1;ID=S2506_raw:GT=hom:DP=19:FQ=1:SQ=-1;ID=S2667_raw:GT=hom:DP=27:FQ=1:SQ=-1;
18	67863854	67863854	-	TCC	exonic	RTTN	nonframeshift insertion	RTTN:NM_173630:exon7:c.723_724insGGA:p.G242delinsGG	.	.	ID=S2009_raw:GT=hom:DP=5:FQ=1:SQ=-1;ID=S2503_raw:GT=hom:DP=68:FQ=1:SQ=-1;ID=S2507_raw:GT=hom:DP=68:FQ=1:SQ=-1;ID=S2668_raw:GT=hom:DP=88:FQ=1:SQ=-1;ID=S2012_raw:GT=hom:DP=6:FQ=1:SQ=-1;ID=S2010_raw:GT=hom:DP=12:FQ=1:SQ=-1;ID=S2662_raw:GT=hom:DP=25:FQ=1:SQ=-1;ID=S2663_raw:GT=hom:DP=47:FQ=1:SQ=-1;ID=S2011_raw:GT=hom:DP=6:FQ=1:SQ=-1;ID=S2504_raw:GT=hom:DP=84:FQ=1:SQ=-1;ID=S2008_raw:GT=hom:DP=9:FQ=1:SQ=-1;ID=S2509_raw:GT=hom:DP=67:FQ=1:SQ=-1;ID=S2508_raw:GT=hom:DP=74:FQ=1:SQ=-1;ID=S2666_raw:GT=hom:DP=58:FQ=1:SQ=-1;ID=S2669_raw:GT=hom:DP=64:FQ=1:SQ=-1;ID=S2166_raw:GT=hom:DP=136:FQ=1:SQ=-1;ID=S1775_raw:GT=hom:DP=85:FQ=1:SQ=-1;ID=S2501_raw:GT=hom:DP=75:FQ=1:SQ=-1;ID=S2500_raw:GT=hom:DP=77:FQ=1:SQ=-1;ID=S2502_raw:GT=hom:DP=68:FQ=1:SQ=-1;ID=S2505_raw:GT=hom:DP=72:FQ=1:SQ=-1;ID=S2176_raw:GT=hom:DP=143:FQ=1:SQ=-1;ID=S2506_raw:GT=hom:DP=54:FQ=1:SQ=-1;ID=S2667_raw:GT=hom:DP=69:FQ=1:SQ=-1;
19	38939505	38939505	T	C	intronic	RYR1	.	.	rs4476278	not_provided	ID=S2009_raw:GT=hom:DP=5:FQ=1:SQ=-1;ID=S2503_raw:GT=hom:DP=6:FQ=1:SQ=-1;ID=S2507_raw:GT=hom:DP=11:FQ=1:SQ=-1;ID=S2668_raw:GT=hom:DP=3:FQ=1:SQ=-1;ID=S2012_raw:GT=hom:DP=7:FQ=1:SQ=-1;ID=S2010_raw:GT=hom:DP=7:FQ=1:SQ=-1;ID=S2662_raw:GT=hom:DP=10:FQ=1:SQ=-1;ID=S2663_raw:GT=hom:DP=5:FQ=1:SQ=-1;ID=S2011_raw:GT=hom:DP=9:FQ=1:SQ=-1;ID=S2504_raw:GT=hom:DP=13:FQ=1:SQ=-1;ID=S2008_raw:GT=hom:DP=7:FQ=1:SQ=-1;ID=S2509_raw:GT=hom:DP=5:FQ=1:SQ=-1;ID=S2508_raw:GT=hom:DP=4:FQ=1:SQ=-1;ID=S2666_raw:GT=hom:DP=6:FQ=1:SQ=-1;ID=S2669_raw:GT=hom:DP=6:FQ=1:SQ=-1;ID=S2166_raw:GT=hom:DP=8:FQ=1:SQ=-1;ID=S1775_raw:GT=hom:DP=7:FQ=1:SQ=-1;ID=S2501_raw:GT=hom:DP=7:FQ=1:SQ=-1;ID=S2500_raw:GT=hom:DP=11:FQ=1:SQ=-1;ID=S2502_raw:GT=hom:DP=6:FQ=1:SQ=-1;ID=S2505_raw:GT=hom:DP=11:FQ=1:SQ=-1;ID=S2176_raw:GT=hom:DP=12:FQ=1:SQ=-1;ID=S2506_raw:GT=hom:DP=14:FQ=1:SQ=-1;ID=S2667_raw:GT=hom:DP=7:FQ=1:SQ=-1;
11	18290880	18290880	C	G	exonic;splicing	SAA1	nonsynonymous SNV	SAA1:NM_199161:exon3:c.230C>G:p.T77S	rs1671926	.	ID=S2009_raw:GT=hom:DP=1:FQ=1:SQ=-1;ID=S2503_raw:GT=hom:DP=81:FQ=1:SQ=-1;ID=S2507_raw:GT=hom:DP=53:FQ=1:SQ=-1;ID=S2668_raw:GT=hom:DP=47:FQ=1:SQ=-1;ID=S2012_raw:GT=hom:DP=3:FQ=1:SQ=-1;ID=S2010_raw:GT=hom:DP=3:FQ=1:SQ=-1;ID=S2662_raw:GT=hom:DP=43:FQ=1:SQ=-1;ID=S2663_raw:GT=hom:DP=37:FQ=1:SQ=-1;ID=S2011_raw:GT=hom:DP=2:FQ=1:SQ=-1;ID=S2504_raw:GT=hom:DP=64:FQ=1:SQ=-1;ID=S2008_raw:GT=hom:DP=8:FQ=1:SQ=-1;ID=S2509_raw:GT=hom:DP=55:FQ=1:SQ=-1;ID=S2508_raw:GT=hom:DP=61:FQ=1:SQ=-1;ID=S2666_raw:GT=hom:DP=54:FQ=1:SQ=-1;ID=S2669_raw:GT=hom:DP=44:FQ=1:SQ=-1;ID=S2166_raw:GT=hom:DP=48:FQ=0.98:SQ=-1;ID=S1775_raw:GT=hom:DP=27:FQ=1:SQ=-1;ID=S2501_raw:GT=hom:DP=87:FQ=1:SQ=-1;ID=S2500_raw:GT=hom:DP=61:FQ=1:SQ=-1;ID=S2502_raw:GT=hom:DP=61:FQ=1:SQ=-1;ID=S2505_raw:GT=hom:DP=42:FQ=1:SQ=-1;ID=S2176_raw:GT=hom:DP=50:FQ=1:SQ=-1;ID=S2506_raw:GT=hom:DP=52:FQ=1:SQ=-1;ID=S2667_raw:GT=hom:DP=50:FQ=1:SQ=-1;
16	51171175	51171175	C	T	exonic	SALL1	nonsynonymous SNV	SALL1:NM_001127892:exon3:c.3532G>A:p.V1178I,SALL1:NM_002968:exon3:c.3823G>A:p.V1275I	rs4614723	Benign	ID=S2009_raw:GT=hom:DP=93:FQ=1:SQ=-1;ID=S2503_raw:GT=hom:DP=166:FQ=1:SQ=-1;ID=S2507_raw:GT=hom:DP=166:FQ=1:SQ=-1;ID=S2668_raw:GT=hom:DP=154:FQ=1:SQ=-1;ID=S2012_raw:GT=hom:DP=77:FQ=1:SQ=-1;ID=S2010_raw:GT=hom:DP=106:FQ=1:SQ=-1;ID=S2662_raw:GT=hom:DP=265:FQ=1:SQ=-1;ID=S2663_raw:GT=hom:DP=156:FQ=1:SQ=-1;ID=S2011_raw:GT=hom:DP=125:FQ=1:SQ=-1;ID=S2504_raw:GT=hom:DP=193:FQ=1:SQ=-1;ID=S2008_raw:GT=hom:DP=112:FQ=1:SQ=-1;ID=S2509_raw:GT=hom:DP=175:FQ=1:SQ=-1;ID=S2508_raw:GT=hom:DP=201:FQ=1:SQ=-1;ID=S2666_raw:GT=hom:DP=120:FQ=1:SQ=-1;ID=S2669_raw:GT=hom:DP=155:FQ=1:SQ=-1;ID=S2166_raw:GT=hom:DP=213:FQ=1:SQ=-1;ID=S1775_raw:GT=hom:DP=129:FQ=1:SQ=-1;ID=S2501_raw:GT=hom:DP=166:FQ=1:SQ=-1;ID=S2500_raw:GT=hom:DP=184:FQ=1:SQ=-1;ID=S2502_raw:GT=hom:DP=178:FQ=1:SQ=-1;ID=S2505_raw:GT=hom:DP=172:FQ=1:SQ=-1;ID=S2176_raw:GT=hom:DP=190:FQ=1:SQ=-1;ID=S2506_raw:GT=hom:DP=132:FQ=1:SQ=-1;ID=S2667_raw:GT=hom:DP=157:FQ=1:SQ=-1;
16	51383777	51383779	AGC	-	intergenic	SALL1;LINC01571	.	dist=198594;dist=412651	.	.	ID=S2009_raw:GT=hom:DP=3:FQ=1:SQ=-1;ID=S2503_raw:GT=hom:DP=10:FQ=1:SQ=-1;ID=S2507_raw:GT=hom:DP=14:FQ=1:SQ=-1;ID=S2668_raw:GT=hom:DP=5:FQ=1:SQ=-1;ID=S2012_raw:GT=hom:DP=9:FQ=1:SQ=-1;ID=S2010_raw:GT=hom:DP=12:FQ=1:SQ=-1;ID=S2662_raw:GT=hom:DP=22:FQ=1:SQ=-1;ID=S2663_raw:GT=hom:DP=9:FQ=1:SQ=-1;ID=S2011_raw:GT=hom:DP=15:FQ=1:SQ=-1;ID=S2504_raw:GT=hom:DP=15:FQ=1:SQ=-1;ID=S2008_raw:GT=hom:DP=13:FQ=1:SQ=-1;ID=S2509_raw:GT=hom:DP=7:FQ=1:SQ=-1;ID=S2508_raw:GT=hom:DP=13:FQ=1:SQ=-1;ID=S2666_raw:GT=hom:DP=10:FQ=1:SQ=-1;ID=S2669_raw:GT=hom:DP=9:FQ=1:SQ=-1;ID=S2166_raw:GT=hom:DP=10:FQ=1:SQ=-1;ID=S1775_raw:GT=hom:DP=11:FQ=1:SQ=-1;ID=S2501_raw:GT=hom:DP=13:FQ=1:SQ=-1;ID=S2500_raw:GT=hom:DP=11:FQ=1:SQ=-1;ID=S2502_raw:GT=hom:DP=16:FQ=1:SQ=-1;ID=S2505_raw:GT=hom:DP=12:FQ=1:SQ=-1;ID=S2176_raw:GT=hom:DP=13:FQ=1:SQ=-1;ID=S2506_raw:GT=hom:DP=5:FQ=1:SQ=-1;ID=S2667_raw:GT=hom:DP=8:FQ=1:SQ=-1;
20	50408482	50408482	A	G	exonic	SALL4	synonymous SNV	SALL4:NM_001318031:exon2:c.540T>C:p.N180N,SALL4:NM_020436:exon2:c.540T>C:p.N180N	rs6013281	Benign	ID=S2009_raw:GT=hom:DP=62:FQ=1:SQ=-1;ID=S2503_raw:GT=hom:DP=145:FQ=1:SQ=-1;ID=S2507_raw:GT=hom:DP=206:FQ=1:SQ=-1;ID=S2668_raw:GT=hom:DP=219:FQ=1:SQ=-1;ID=S2012_raw:GT=hom:DP=66:FQ=1:SQ=-1;ID=S2010_raw:GT=hom:DP=99:FQ=1:SQ=-1;ID=S2662_raw:GT=hom:DP=263:FQ=0.99:SQ=-1;ID=S2663_raw:GT=hom:DP=182:FQ=1:SQ=-1;ID=S2011_raw:GT=hom:DP=97:FQ=1:SQ=-1;ID=S2504_raw:GT=hom:DP=238:FQ=1:SQ=-1;ID=S2008_raw:GT=hom:DP=90:FQ=1:SQ=-1;ID=S2509_raw:GT=hom:DP=187:FQ=1:SQ=-1;ID=S2508_raw:GT=hom:DP=180:FQ=1:SQ=-1;ID=S2666_raw:GT=hom:DP=250:FQ=1:SQ=-1;ID=S2669_raw:GT=hom:DP=167:FQ=1:SQ=-1;ID=S2166_raw:GT=hom:DP=154:FQ=1:SQ=-1;ID=S1775_raw:GT=hom:DP=92:FQ=1:SQ=-1;ID=S2501_raw:GT=hom:DP=160:FQ=1:SQ=-1;ID=S2500_raw:GT=hom:DP=165:FQ=1:SQ=-1;ID=S2502_raw:GT=hom:DP=211:FQ=1:SQ=-1;ID=S2505_raw:GT=hom:DP=223:FQ=1:SQ=-1;ID=S2176_raw:GT=hom:DP=183:FQ=1:SQ=-1;ID=S2506_raw:GT=hom:DP=174:FQ=1:SQ=-1;ID=S2667_raw:GT=hom:DP=190:FQ=0.99:SQ=-1;
22	20779946	20779946	C	G	exonic	SCARF2	synonymous SNV	SCARF2:NM_153334:exon14:c.2331G>C:p.T777T,SCARF2:NM_182895:exon14:c.2316G>C:p.T772T	rs759612	.	ID=S2009_raw:GT=hom:DP=37:FQ=1:SQ=-1;ID=S2503_raw:GT=hom:DP=25:FQ=1:SQ=-1;ID=S2507_raw:GT=hom:DP=28:FQ=1:SQ=-1;ID=S2668_raw:GT=hom:DP=19:FQ=1:SQ=-1;ID=S2012_raw:GT=hom:DP=20:FQ=1:SQ=-1;ID=S2010_raw:GT=hom:DP=23:FQ=1:SQ=-1;ID=S2662_raw:GT=hom:DP=62:FQ=1:SQ=-1;ID=S2663_raw:GT=hom:DP=37:FQ=1:SQ=-1;ID=S2011_raw:GT=hom:DP=17:FQ=1:SQ=-1;ID=S2504_raw:GT=hom:DP=22:FQ=1:SQ=-1;ID=S2008_raw:GT=hom:DP=31:FQ=0.97:SQ=-1;ID=S2509_raw:GT=hom:DP=23:FQ=1:SQ=-1;ID=S2508_raw:GT=hom:DP=34:FQ=1:SQ=-1;ID=S2666_raw:GT=hom:DP=2:FQ=1:SQ=-1;ID=S2669_raw:GT=hom:DP=45:FQ=1:SQ=-1;ID=S2166_raw:GT=hom:DP=31:FQ=1:SQ=-1;ID=S1775_raw:GT=hom:DP=15:FQ=1:SQ=-1;ID=S2501_raw:GT=hom:DP=17:FQ=1:SQ=-1;ID=S2500_raw:GT=hom:DP=11:FQ=1:SQ=-1;ID=S2502_raw:GT=hom:DP=25:FQ=1:SQ=-1;ID=S2505_raw:GT=hom:DP=18:FQ=1:SQ=-1;ID=S2176_raw:GT=hom:DP=28:FQ=1:SQ=-1;ID=S2506_raw:GT=hom:DP=34:FQ=1:SQ=-1;ID=S2667_raw:GT=hom:DP=37:FQ=1:SQ=-1;
22	20779947	20779947	G	C	exonic	SCARF2	nonsynonymous SNV	SCARF2:NM_153334:exon14:c.2330C>G:p.T777R,SCARF2:NM_182895:exon14:c.2315C>G:p.T772R	rs759611	.	ID=S2009_raw:GT=hom:DP=36:FQ=1:SQ=-1;ID=S2503_raw:GT=hom:DP=26:FQ=1:SQ=-1;ID=S2507_raw:GT=hom:DP=28:FQ=1:SQ=-1;ID=S2668_raw:GT=hom:DP=20:FQ=1:SQ=-1;ID=S2012_raw:GT=hom:DP=20:FQ=1:SQ=-1;ID=S2010_raw:GT=hom:DP=23:FQ=1:SQ=-1;ID=S2662_raw:GT=hom:DP=61:FQ=1:SQ=-1;ID=S2663_raw:GT=hom:DP=37:FQ=1:SQ=-1;ID=S2011_raw:GT=hom:DP=18:FQ=1:SQ=-1;ID=S2504_raw:GT=hom:DP=22:FQ=1:SQ=-1;ID=S2008_raw:GT=hom:DP=32:FQ=1:SQ=-1;ID=S2509_raw:GT=hom:DP=23:FQ=1:SQ=-1;ID=S2508_raw:GT=hom:DP=34:FQ=1:SQ=-1;ID=S2666_raw:GT=hom:DP=2:FQ=1:SQ=-1;ID=S2669_raw:GT=hom:DP=44:FQ=1:SQ=-1;ID=S2166_raw:GT=hom:DP=31:FQ=1:SQ=-1;ID=S1775_raw:GT=hom:DP=15:FQ=1:SQ=-1;ID=S2501_raw:GT=hom:DP=17:FQ=1:SQ=-1;ID=S2500_raw:GT=hom:DP=10:FQ=1:SQ=-1;ID=S2502_raw:GT=hom:DP=24:FQ=1:SQ=-1;ID=S2505_raw:GT=hom:DP=18:FQ=1:SQ=-1;ID=S2176_raw:GT=hom:DP=28:FQ=1:SQ=-1;ID=S2506_raw:GT=hom:DP=34:FQ=1:SQ=-1;ID=S2667_raw:GT=hom:DP=36:FQ=1:SQ=-1;
22	20779975	20779975	-	G	exonic;splicing	SCARF2	frameshift insertion	SCARF2:NM_153334:exon13:c.2302dupC:p.P768fs,SCARF2:NM_182895:exon13:c.2287dupC:p.P763fs	.	.	ID=S2009_raw:GT=hom:DP=21:FQ=1:SQ=-1;ID=S2503_raw:GT=hom:DP=11:FQ=1:SQ=-1;ID=S2507_raw:GT=hom:DP=11:FQ=1:SQ=-1;ID=S2668_raw:GT=hom:DP=12:FQ=1:SQ=-1;ID=S2012_raw:GT=hom:DP=12:FQ=1:SQ=-1;ID=S2010_raw:GT=hom:DP=18:FQ=1:SQ=-1;ID=S2662_raw:GT=hom:DP=29:FQ=1:SQ=-1;ID=S2663_raw:GT=hom:DP=19:FQ=1:SQ=-1;ID=S2011_raw:GT=hom:DP=13:FQ=1:SQ=-1;ID=S2504_raw:GT=hom:DP=14:FQ=1:SQ=-1;ID=S2008_raw:GT=hom:DP=24:FQ=1:SQ=-1;ID=S2509_raw:GT=hom:DP=9:FQ=1:SQ=-1;ID=S2508_raw:GT=hom:DP=15:FQ=1:SQ=-1;ID=S2666_raw:GT=hom:DP=2:FQ=1:SQ=-1;ID=S2669_raw:GT=hom:DP=24:FQ=1:SQ=-1;ID=S2166_raw:GT=hom:DP=29:FQ=1:SQ=-1;ID=S1775_raw:GT=hom:DP=6:FQ=1:SQ=-1;ID=S2501_raw:GT=hom:DP=5:FQ=1:SQ=-1;ID=S2500_raw:GT=hom:DP=5:FQ=1:SQ=-1;ID=S2502_raw:GT=hom:DP=15:FQ=1:SQ=-1;ID=S2505_raw:GT=hom:DP=8:FQ=1:SQ=-1;ID=S2176_raw:GT=hom:DP=22:FQ=1:SQ=-1;ID=S2506_raw:GT=hom:DP=21:FQ=1:SQ=-1;ID=S2667_raw:GT=hom:DP=13:FQ=1:SQ=-1;
22	20780091	20780091	C	G	exonic	SCARF2	synonymous SNV	SCARF2:NM_153334:exon11:c.2187G>C:p.P729P,SCARF2:NM_182895:exon11:c.2172G>C:p.P724P	rs759610	.	ID=S2009_raw:GT=hom:DP=50:FQ=1:SQ=-1;ID=S2503_raw:GT=hom:DP=71:FQ=1:SQ=-1;ID=S2507_raw:GT=hom:DP=124:FQ=1:SQ=-1;ID=S2668_raw:GT=hom:DP=86:FQ=1:SQ=-1;ID=S2012_raw:GT=hom:DP=39:FQ=1:SQ=-1;ID=S2010_raw:GT=hom:DP=55:FQ=1:SQ=-1;ID=S2662_raw:GT=hom:DP=194:FQ=1:SQ=-1;ID=S2663_raw:GT=hom:DP=116:FQ=1:SQ=-1;ID=S2011_raw:GT=hom:DP=44:FQ=1:SQ=-1;ID=S2504_raw:GT=hom:DP=82:FQ=1:SQ=-1;ID=S2008_raw:GT=hom:DP=48:FQ=1:SQ=-1;ID=S2509_raw:GT=hom:DP=110:FQ=1:SQ=-1;ID=S2508_raw:GT=hom:DP=119:FQ=1:SQ=-1;ID=S2666_raw:GT=hom:DP=39:FQ=1:SQ=-1;ID=S2669_raw:GT=hom:DP=157:FQ=1:SQ=-1;ID=S2166_raw:GT=hom:DP=104:FQ=1:SQ=-1;ID=S1775_raw:GT=hom:DP=48:FQ=1:SQ=-1;ID=S2501_raw:GT=hom:DP=84:FQ=1:SQ=-1;ID=S2500_raw:GT=hom:DP=81:FQ=1:SQ=-1;ID=S2502_raw:GT=hom:DP=102:FQ=1:SQ=-1;ID=S2505_raw:GT=hom:DP=84:FQ=1:SQ=-1;ID=S2176_raw:GT=hom:DP=77:FQ=1:SQ=-1;ID=S2506_raw:GT=hom:DP=117:FQ=1:SQ=-1;ID=S2667_raw:GT=hom:DP=140:FQ=1:SQ=-1;
22	20780097	20780097	G	C	exonic	SCARF2	synonymous SNV	SCARF2:NM_153334:exon11:c.2181C>G:p.R727R,SCARF2:NM_182895:exon11:c.2166C>G:p.R722R	rs759609	.	ID=S2009_raw:GT=hom:DP=49:FQ=1:SQ=-1;ID=S2503_raw:GT=hom:DP=71:FQ=1:SQ=-1;ID=S2507_raw:GT=hom:DP=123:FQ=1:SQ=-1;ID=S2668_raw:GT=hom:DP=84:FQ=1:SQ=-1;ID=S2012_raw:GT=hom:DP=39:FQ=1:SQ=-1;ID=S2010_raw:GT=hom:DP=55:FQ=1:SQ=-1;ID=S2662_raw:GT=hom:DP=194:FQ=1:SQ=-1;ID=S2663_raw:GT=hom:DP=114:FQ=1:SQ=-1;ID=S2011_raw:GT=hom:DP=44:FQ=1:SQ=-1;ID=S2504_raw:GT=hom:DP=81:FQ=1:SQ=-1;ID=S2008_raw:GT=hom:DP=48:FQ=1:SQ=-1;ID=S2509_raw:GT=hom:DP=109:FQ=1:SQ=-1;ID=S2508_raw:GT=hom:DP=119:FQ=1:SQ=-1;ID=S2666_raw:GT=hom:DP=39:FQ=1:SQ=-1;ID=S2669_raw:GT=hom:DP=155:FQ=1:SQ=-1;ID=S2166_raw:GT=hom:DP=104:FQ=1:SQ=-1;ID=S1775_raw:GT=hom:DP=49:FQ=1:SQ=-1;ID=S2501_raw:GT=hom:DP=83:FQ=1:SQ=-1;ID=S2500_raw:GT=hom:DP=81:FQ=1:SQ=-1;ID=S2502_raw:GT=hom:DP=102:FQ=1:SQ=-1;ID=S2505_raw:GT=hom:DP=87:FQ=1:SQ=-1;ID=S2176_raw:GT=hom:DP=77:FQ=1:SQ=-1;ID=S2506_raw:GT=hom:DP=117:FQ=1:SQ=-1;ID=S2667_raw:GT=hom:DP=139:FQ=1:SQ=-1;
2	1,66E+08	1,66E+08	T	C	intronic	SCN2A	.	.	rs6432821	.	ID=S2009_raw:GT=hom:DP=67:FQ=1:SQ=-1;ID=S2503_raw:GT=hom:DP=51:FQ=1:SQ=-1;ID=S2507_raw:GT=hom:DP=50:FQ=1:SQ=-1;ID=S2668_raw:GT=hom:DP=105:FQ=1:SQ=-1;ID=S2012_raw:GT=hom:DP=86:FQ=1:SQ=-1;ID=S2010_raw:GT=hom:DP=87:FQ=1:SQ=-1;ID=S2662_raw:GT=hom:DP=5:FQ=1:SQ=-1;ID=S2663_raw:GT=hom:DP=69:FQ=1:SQ=-1;ID=S2011_raw:GT=hom:DP=113:FQ=1:SQ=-1;ID=S2504_raw:GT=hom:DP=42:FQ=1:SQ=-1;ID=S2008_raw:GT=hom:DP=75:FQ=1:SQ=-1;ID=S2509_raw:GT=hom:DP=49:FQ=1:SQ=-1;ID=S2508_raw:GT=hom:DP=72:FQ=1:SQ=-1;ID=S2666_raw:GT=hom:DP=81:FQ=1:SQ=-1;ID=S2669_raw:GT=hom:DP=61:FQ=1:SQ=-1;ID=S2166_raw:GT=hom:DP=18:FQ=1:SQ=-1;ID=S1775_raw:GT=hom:DP=9:FQ=1:SQ=-1;ID=S2501_raw:GT=hom:DP=58:FQ=1:SQ=-1;ID=S2500_raw:GT=hom:DP=42:FQ=1:SQ=-1;ID=S2502_raw:GT=hom:DP=46:FQ=1:SQ=-1;ID=S2505_raw:GT=hom:DP=54:FQ=1:SQ=-1;ID=S2176_raw:GT=hom:DP=21:FQ=1:SQ=-1;ID=S2506_raw:GT=hom:DP=44:FQ=1:SQ=-1;ID=S2667_raw:GT=hom:DP=60:FQ=1:SQ=-1;
20	18528343	18528343	G	A	intronic	SEC23B	.	.	rs4814761	.	ID=S2009_raw:GT=hom:DP=3:FQ=1:SQ=-1;ID=S2503_raw:GT=hom:DP=3:FQ=1:SQ=-1;ID=S2507_raw:GT=hom:DP=3:FQ=1:SQ=-1;ID=S2668_raw:GT=hom:DP=7:FQ=1:SQ=-1;ID=S2012_raw:GT=hom:DP=4:FQ=1:SQ=-1;ID=S2010_raw:GT=hom:DP=2:FQ=1:SQ=-1;ID=S2662_raw:GT=hom:DP=13:FQ=1:SQ=-1;ID=S2663_raw:GT=hom:DP=4:FQ=1:SQ=-1;ID=S2011_raw:GT=hom:DP=3:FQ=1:SQ=-1;ID=S2504_raw:GT=hom:DP=4:FQ=1:SQ=-1;ID=S2008_raw:GT=hom:DP=4:FQ=1:SQ=-1;ID=S2509_raw:GT=hom:DP=8:FQ=1:SQ=-1;ID=S2508_raw:GT=hom:DP=3:FQ=1:SQ=-1;ID=S2666_raw:GT=hom:DP=5:FQ=1:SQ=-1;ID=S2669_raw:GT=hom:DP=6:FQ=1:SQ=-1;ID=S2166_raw:GT=hom:DP=9:FQ=1:SQ=-1;ID=S1775_raw:GT=hom:DP=4:FQ=1:SQ=-1;ID=S2501_raw:GT=hom:DP=4:FQ=1:SQ=-1;ID=S2500_raw:GT=hom:DP=6:FQ=1:SQ=-1;ID=S2502_raw:GT=hom:DP=4:FQ=1:SQ=-1;ID=S2505_raw:GT=hom:DP=6:FQ=1:SQ=-1;ID=S2176_raw:GT=hom:DP=2:FQ=1:SQ=-1;ID=S2506_raw:GT=hom:DP=5:FQ=1:SQ=-1;ID=S2667_raw:GT=hom:DP=9:FQ=1:SQ=-1;
20	18531499	18531499	G	C	intronic	SEC23B	.	.	rs6112024	.	ID=S2009_raw:GT=hom:DP=7:FQ=1:SQ=-1;ID=S2503_raw:GT=hom:DP=10:FQ=1:SQ=-1;ID=S2507_raw:GT=hom:DP=10:FQ=1:SQ=-1;ID=S2668_raw:GT=hom:DP=24:FQ=1:SQ=-1;ID=S2012_raw:GT=hom:DP=15:FQ=1:SQ=-1;ID=S2010_raw:GT=hom:DP=5:FQ=1:SQ=-1;ID=S2662_raw:GT=hom:DP=4:FQ=1:SQ=-1;ID=S2663_raw:GT=hom:DP=11:FQ=1:SQ=-1;ID=S2011_raw:GT=hom:DP=18:FQ=1:SQ=-1;ID=S2504_raw:GT=hom:DP=11:FQ=1:SQ=-1;ID=S2008_raw:GT=hom:DP=12:FQ=1:SQ=-1;ID=S2509_raw:GT=hom:DP=9:FQ=1:SQ=-1;ID=S2508_raw:GT=hom:DP=9:FQ=1:SQ=-1;ID=S2666_raw:GT=hom:DP=22:FQ=1:SQ=-1;ID=S2669_raw:GT=hom:DP=4:FQ=1:SQ=-1;ID=S2166_raw:GT=hom:DP=4:FQ=1:SQ=-1;ID=S1775_raw:GT=hom:DP=7:FQ=1:SQ=-1;ID=S2501_raw:GT=hom:DP=9:FQ=1:SQ=-1;ID=S2500_raw:GT=hom:DP=18:FQ=1:SQ=-1;ID=S2502_raw:GT=hom:DP=15:FQ=1:SQ=-1;ID=S2505_raw:GT=hom:DP=20:FQ=1:SQ=-1;ID=S2176_raw:GT=hom:DP=4:FQ=1:SQ=-1;ID=S2506_raw:GT=hom:DP=7:FQ=1:SQ=-1;ID=S2667_raw:GT=hom:DP=14:FQ=1:SQ=-1;
6	1,08E+08	1,08E+08	T	C	intronic	SEC63	.	.	rs579120	.	ID=S2009_raw:GT=hom:DP=31:FQ=1:SQ=-1;ID=S2503_raw:GT=hom:DP=16:FQ=1:SQ=-1;ID=S2507_raw:GT=hom:DP=13:FQ=1:SQ=-1;ID=S2668_raw:GT=hom:DP=27:FQ=1:SQ=-1;ID=S2012_raw:GT=hom:DP=48:FQ=1:SQ=-1;ID=S2010_raw:GT=hom:DP=39:FQ=1:SQ=-1;ID=S2662_raw:GT=hom:DP=2:FQ=1:SQ=-1;ID=S2663_raw:GT=hom:DP=14:FQ=1:SQ=-1;ID=S2011_raw:GT=hom:DP=64:FQ=1:SQ=-1;ID=S2504_raw:GT=hom:DP=24:FQ=1:SQ=-1;ID=S2008_raw:GT=hom:DP=53:FQ=1:SQ=-1;ID=S2509_raw:GT=hom:DP=24:FQ=1:SQ=-1;ID=S2508_raw:GT=hom:DP=29:FQ=1:SQ=-1;ID=S2666_raw:GT=hom:DP=32:FQ=1:SQ=-1;ID=S2669_raw:GT=hom:DP=7:FQ=1:SQ=-1;ID=S2166_raw:GT=hom:DP=51:FQ=1:SQ=-1;ID=S1775_raw:GT=hom:DP=32:FQ=1:SQ=-1;ID=S2501_raw:GT=hom:DP=30:FQ=1:SQ=-1;ID=S2500_raw:GT=hom:DP=32:FQ=1:SQ=-1;ID=S2502_raw:GT=hom:DP=25:FQ=1:SQ=-1;ID=S2505_raw:GT=hom:DP=25:FQ=1:SQ=-1;ID=S2176_raw:GT=hom:DP=56:FQ=1:SQ=-1;ID=S2506_raw:GT=hom:DP=24:FQ=1:SQ=-1;ID=S2667_raw:GT=hom:DP=34:FQ=1:SQ=-1;
6	1,08E+08	1,08E+08	A	G	intronic	SEC63	.	.	rs570558	.	ID=S2009_raw:GT=hom:DP=1:FQ=1:SQ=-1;ID=S2503_raw:GT=hom:DP=58:FQ=1:SQ=-1;ID=S2507_raw:GT=hom:DP=50:FQ=1:SQ=-1;ID=S2668_raw:GT=hom:DP=79:FQ=1:SQ=-1;ID=S2012_raw:GT=hom:DP=4:FQ=1:SQ=-1;ID=S2010_raw:GT=hom:DP=12:FQ=1:SQ=-1;ID=S2662_raw:GT=hom:DP=2:FQ=1:SQ=-1;ID=S2663_raw:GT=hom:DP=72:FQ=1:SQ=-1;ID=S2011_raw:GT=hom:DP=4:FQ=1:SQ=-1;ID=S2504_raw:GT=hom:DP=68:FQ=1:SQ=-1;ID=S2008_raw:GT=hom:DP=2:FQ=1:SQ=-1;ID=S2509_raw:GT=hom:DP=65:FQ=1:SQ=-1;ID=S2508_raw:GT=hom:DP=69:FQ=1:SQ=-1;ID=S2666_raw:GT=hom:DP=92:FQ=1:SQ=-1;ID=S2669_raw:GT=hom:DP=14:FQ=1:SQ=-1;ID=S2166_raw:GT=hom:DP=8:FQ=1:SQ=-1;ID=S1775_raw:GT=hom:DP=18:FQ=1:SQ=-1;ID=S2501_raw:GT=hom:DP=64:FQ=1:SQ=-1;ID=S2500_raw:GT=hom:DP=88:FQ=1:SQ=-1;ID=S2502_raw:GT=hom:DP=72:FQ=1:SQ=-1;ID=S2505_raw:GT=hom:DP=58:FQ=1:SQ=-1;ID=S2176_raw:GT=hom:DP=8:FQ=1:SQ=-1;ID=S2506_raw:GT=hom:DP=57:FQ=1:SQ=-1;ID=S2667_raw:GT=hom:DP=37:FQ=1:SQ=-1;
6	1,08E+08	1,08E+08	T	C	intronic	SEC63	.	.	rs4945798	.	ID=S2009_raw:GT=hom:DP=1:FQ=1:SQ=-1;ID=S2503_raw:GT=hom:DP=6:FQ=1:SQ=-1;ID=S2507_raw:GT=hom:DP=4:FQ=1:SQ=-1;ID=S2668_raw:GT=hom:DP=13:FQ=1:SQ=-1;ID=S2012_raw:GT=hom:DP=4:FQ=1:SQ=-1;ID=S2010_raw:GT=hom:DP=4:FQ=1:SQ=-1;ID=S2662_raw:GT=hom:DP=10:FQ=1:SQ=-1;ID=S2663_raw:GT=hom:DP=11:FQ=1:SQ=-1;ID=S2011_raw:GT=hom:DP=2:FQ=1:SQ=-1;ID=S2504_raw:GT=hom:DP=10:FQ=1:SQ=-1;ID=S2008_raw:GT=hom:DP=2:FQ=1:SQ=-1;ID=S2509_raw:GT=hom:DP=7:FQ=1:SQ=-1;ID=S2508_raw:GT=hom:DP=9:FQ=1:SQ=-1;ID=S2666_raw:GT=hom:DP=5:FQ=1:SQ=-1;ID=S2669_raw:GT=hom:DP=16:FQ=1:SQ=-1;ID=S2166_raw:GT=hom:DP=1:FQ=1:SQ=-1;ID=S1775_raw:GT=hom:DP=2:FQ=1:SQ=-1;ID=S2501_raw:GT=hom:DP=8:FQ=1:SQ=-1;ID=S2500_raw:GT=hom:DP=5:FQ=1:SQ=-1;ID=S2502_raw:GT=hom:DP=4:FQ=1:SQ=-1;ID=S2505_raw:GT=hom:DP=3:FQ=1:SQ=-1;ID=S2176_raw:GT=hom:DP=4:FQ=1:SQ=-1;ID=S2506_raw:GT=hom:DP=4:FQ=1:SQ=-1;ID=S2667_raw:GT=hom:DP=11:FQ=1:SQ=-1;
6	1,08E+08	1,08E+08	C	A	intronic	SEC63	.	.	rs9486756	.	ID=S2009_raw:GT=hom:DP=20:FQ=1:SQ=-1;ID=S2503_raw:GT=hom:DP=31:FQ=1:SQ=-1;ID=S2507_raw:GT=hom:DP=22:FQ=1:SQ=-1;ID=S2668_raw:GT=hom:DP=20:FQ=1:SQ=-1;ID=S2012_raw:GT=hom:DP=16:FQ=1:SQ=-1;ID=S2010_raw:GT=hom:DP=20:FQ=1:SQ=-1;ID=S2662_raw:GT=hom:DP=32:FQ=1:SQ=-1;ID=S2663_raw:GT=hom:DP=18:FQ=1:SQ=-1;ID=S2011_raw:GT=hom:DP=30:FQ=1:SQ=-1;ID=S2504_raw:GT=hom:DP=29:FQ=1:SQ=-1;ID=S2008_raw:GT=hom:DP=19:FQ=1:SQ=-1;ID=S2509_raw:GT=hom:DP=28:FQ=1:SQ=-1;ID=S2508_raw:GT=hom:DP=37:FQ=1:SQ=-1;ID=S2666_raw:GT=hom:DP=22:FQ=1:SQ=-1;ID=S2669_raw:GT=hom:DP=25:FQ=1:SQ=-1;ID=S2166_raw:GT=hom:DP=33:FQ=1:SQ=-1;ID=S1775_raw:GT=hom:DP=46:FQ=1:SQ=-1;ID=S2501_raw:GT=hom:DP=40:FQ=1:SQ=-1;ID=S2500_raw:GT=hom:DP=34:FQ=1:SQ=-1;ID=S2502_raw:GT=hom:DP=21:FQ=1:SQ=-1;ID=S2505_raw:GT=hom:DP=30:FQ=1:SQ=-1;ID=S2176_raw:GT=hom:DP=63:FQ=1:SQ=-1;ID=S2506_raw:GT=hom:DP=36:FQ=1:SQ=-1;ID=S2667_raw:GT=hom:DP=33:FQ=1:SQ=-1;
6	1,08E+08	1,08E+08	G	A	UTR5	SEC63	.	NM_007214:c.-77C>T	rs2272884	Benign	ID=S2009_raw:GT=hom:DP=19:FQ=1:SQ=-1;ID=S2503_raw:GT=hom:DP=53:FQ=1:SQ=-1;ID=S2507_raw:GT=hom:DP=56:FQ=1:SQ=-1;ID=S2668_raw:GT=hom:DP=64:FQ=1:SQ=-1;ID=S2012_raw:GT=hom:DP=22:FQ=1:SQ=-1;ID=S2010_raw:GT=hom:DP=18:FQ=1:SQ=-1;ID=S2662_raw:GT=hom:DP=69:FQ=1:SQ=-1;ID=S2663_raw:GT=hom:DP=54:FQ=1:SQ=-1;ID=S2011_raw:GT=hom:DP=34:FQ=1:SQ=-1;ID=S2504_raw:GT=hom:DP=47:FQ=1:SQ=-1;ID=S2008_raw:GT=hom:DP=26:FQ=1:SQ=-1;ID=S2509_raw:GT=hom:DP=72:FQ=1:SQ=-1;ID=S2508_raw:GT=hom:DP=62:FQ=1:SQ=-1;ID=S2666_raw:GT=hom:DP=24:FQ=1:SQ=-1;ID=S2669_raw:GT=hom:DP=45:FQ=1:SQ=-1;ID=S2166_raw:GT=hom:DP=39:FQ=1:SQ=-1;ID=S1775_raw:GT=hom:DP=30:FQ=1:SQ=-1;ID=S2501_raw:GT=hom:DP=44:FQ=1:SQ=-1;ID=S2500_raw:GT=hom:DP=26:FQ=1:SQ=-1;ID=S2502_raw:GT=hom:DP=64:FQ=1:SQ=-1;ID=S2505_raw:GT=hom:DP=74:FQ=1:SQ=-1;ID=S2176_raw:GT=hom:DP=52:FQ=1:SQ=-1;ID=S2506_raw:GT=hom:DP=37:FQ=1:SQ=-1;ID=S2667_raw:GT=hom:DP=71:FQ=1:SQ=-1;
7	83023603	83023604	CA	-	splicing	SEMA3E	.	NM_012431:exon13:r.spl;NM_001178129:exon13:r.spl	.	.	ID=S2009_raw:GT=hom:DP=33:FQ=1:SQ=-1;ID=S2503_raw:GT=hom:DP=53:FQ=1:SQ=-1;ID=S2507_raw:GT=hom:DP=54:FQ=1:SQ=-1;ID=S2668_raw:GT=hom:DP=51:FQ=1:SQ=-1;ID=S2012_raw:GT=hom:DP=46:FQ=1:SQ=-1;ID=S2010_raw:GT=hom:DP=62:FQ=1:SQ=-1;ID=S2662_raw:GT=hom:DP=3:FQ=1:SQ=-1;ID=S2663_raw:GT=hom:DP=58:FQ=1:SQ=-1;ID=S2011_raw:GT=hom:DP=82:FQ=1:SQ=-1;ID=S2504_raw:GT=hom:DP=57:FQ=1:SQ=-1;ID=S2008_raw:GT=hom:DP=60:FQ=1:SQ=-1;ID=S2509_raw:GT=hom:DP=89:FQ=1:SQ=-1;ID=S2508_raw:GT=hom:DP=79:FQ=1:SQ=-1;ID=S2666_raw:GT=hom:DP=43:FQ=1:SQ=-1;ID=S2669_raw:GT=hom:DP=18:FQ=1:SQ=-1;ID=S2166_raw:GT=hom:DP=77:FQ=1:SQ=-1;ID=S1775_raw:GT=hom:DP=38:FQ=1:SQ=-1;ID=S2501_raw:GT=hom:DP=63:FQ=1:SQ=-1;ID=S2500_raw:GT=hom:DP=66:FQ=1:SQ=-1;ID=S2502_raw:GT=hom:DP=66:FQ=1:SQ=-1;ID=S2505_raw:GT=hom:DP=87:FQ=0.98:SQ=-1;ID=S2176_raw:GT=hom:DP=77:FQ=1:SQ=-1;ID=S2506_raw:GT=hom:DP=72:FQ=1:SQ=-1;ID=S2667_raw:GT=hom:DP=50:FQ=0.98:SQ=-1;
10	72619205	72619205	C	T	exonic	SGPL1	synonymous SNV	SGPL1:NM_003901:exon7:c.564C>T:p.I188I	rs827255	.	ID=S2009_raw:GT=hom:DP=73:FQ=1:SQ=-1;ID=S2503_raw:GT=hom:DP=117:FQ=1:SQ=-1;ID=S2507_raw:GT=hom:DP=126:FQ=1:SQ=-1;ID=S2668_raw:GT=hom:DP=162:FQ=1:SQ=-1;ID=S2012_raw:GT=hom:DP=88:FQ=1:SQ=-1;ID=S2010_raw:GT=hom:DP=95:FQ=1:SQ=-1;ID=S2662_raw:GT=hom:DP=30:FQ=1:SQ=-1;ID=S2663_raw:GT=hom:DP=130:FQ=1:SQ=-1;ID=S2011_raw:GT=hom:DP=131:FQ=1:SQ=-1;ID=S2504_raw:GT=hom:DP=108:FQ=1:SQ=-1;ID=S2008_raw:GT=hom:DP=111:FQ=1:SQ=-1;ID=S2509_raw:GT=hom:DP=154:FQ=1:SQ=-1;ID=S2508_raw:GT=hom:DP=108:FQ=1:SQ=-1;ID=S2666_raw:GT=hom:DP=114:FQ=1:SQ=-1;ID=S2669_raw:GT=hom:DP=99:FQ=1:SQ=-1;ID=S2166_raw:GT=hom:DP=230:FQ=1:SQ=-1;ID=S1775_raw:GT=hom:DP=123:FQ=1:SQ=-1;ID=S2501_raw:GT=hom:DP=108:FQ=1:SQ=-1;ID=S2500_raw:GT=hom:DP=108:FQ=1:SQ=-1;ID=S2502_raw:GT=hom:DP=142:FQ=1:SQ=-1;ID=S2505_raw:GT=hom:DP=150:FQ=1:SQ=-1;ID=S2176_raw:GT=hom:DP=306:FQ=1:SQ=-1;ID=S2506_raw:GT=hom:DP=134:FQ=1:SQ=-1;ID=S2667_raw:GT=hom:DP=82:FQ=1:SQ=-1;
7	1,56E+08	1,56E+08	A	G	intergenic	SHH;LOC389602	.	dist=144876;dist=5293	rs10256182	.	ID=S2009_raw:GT=hom:DP=22:FQ=1:SQ=-1;ID=S2503_raw:GT=hom:DP=104:FQ=0.99:SQ=-1;ID=S2507_raw:GT=hom:DP=56:FQ=1:SQ=-1;ID=S2668_raw:GT=hom:DP=138:FQ=1:SQ=-1;ID=S2012_raw:GT=hom:DP=23:FQ=1:SQ=-1;ID=S2010_raw:GT=hom:DP=27:FQ=1:SQ=-1;ID=S2662_raw:GT=hom:DP=161:FQ=1:SQ=-1;ID=S2663_raw:GT=hom:DP=111:FQ=1:SQ=-1;ID=S2011_raw:GT=hom:DP=51:FQ=1:SQ=-1;ID=S2504_raw:GT=hom:DP=116:FQ=0.99:SQ=-1;ID=S2008_raw:GT=hom:DP=39:FQ=1:SQ=-1;ID=S2509_raw:GT=hom:DP=100:FQ=1:SQ=-1;ID=S2508_raw:GT=hom:DP=147:FQ=1:SQ=-1;ID=S2666_raw:GT=hom:DP=101:FQ=0.99:SQ=-1;ID=S2669_raw:GT=hom:DP=143:FQ=1:SQ=-1;ID=S2166_raw:GT=hom:DP=74:FQ=1:SQ=-1;ID=S1775_raw:GT=hom:DP=57:FQ=1:SQ=-1;ID=S2501_raw:GT=hom:DP=90:FQ=1:SQ=-1;ID=S2500_raw:GT=hom:DP=111:FQ=1:SQ=-1;ID=S2502_raw:GT=hom:DP=79:FQ=1:SQ=-1;ID=S2505_raw:GT=hom:DP=116:FQ=1:SQ=-1;ID=S2176_raw:GT=hom:DP=89:FQ=1:SQ=-1;ID=S2506_raw:GT=hom:DP=87:FQ=1:SQ=-1;ID=S2667_raw:GT=hom:DP=123:FQ=1:SQ=-1;
17	3514028	3514028	G	C	exonic	SHPK	nonsynonymous SNV	SHPK:NM_013276:exon7:c.1263C>G:p.D421E	rs224496	.	ID=S2009_raw:GT=hom:DP=114:FQ=1:SQ=-1;ID=S2503_raw:GT=hom:DP=119:FQ=1:SQ=-1;ID=S2507_raw:GT=hom:DP=127:FQ=1:SQ=-1;ID=S2668_raw:GT=hom:DP=157:FQ=1:SQ=-1;ID=S2012_raw:GT=hom:DP=144:FQ=1:SQ=-1;ID=S2010_raw:GT=hom:DP=146:FQ=1:SQ=-1;ID=S2662_raw:GT=hom:DP=170:FQ=1:SQ=-1;ID=S2663_raw:GT=hom:DP=150:FQ=1:SQ=-1;ID=S2011_raw:GT=hom:DP=188:FQ=1:SQ=-1;ID=S2504_raw:GT=hom:DP=137:FQ=1:SQ=-1;ID=S2008_raw:GT=hom:DP=162:FQ=1:SQ=-1;ID=S2509_raw:GT=hom:DP=126:FQ=1:SQ=-1;ID=S2508_raw:GT=hom:DP=143:FQ=1:SQ=-1;ID=S2666_raw:GT=hom:DP=150:FQ=1:SQ=-1;ID=S2669_raw:GT=hom:DP=158:FQ=1:SQ=-1;ID=S2166_raw:GT=hom:DP=126:FQ=1:SQ=-1;ID=S1775_raw:GT=hom:DP=105:FQ=1:SQ=-1;ID=S2501_raw:GT=hom:DP=120:FQ=1:SQ=-1;ID=S2500_raw:GT=hom:DP=119:FQ=1:SQ=-1;ID=S2502_raw:GT=hom:DP=164:FQ=1:SQ=-1;ID=S2505_raw:GT=hom:DP=141:FQ=1:SQ=-1;ID=S2176_raw:GT=hom:DP=160:FQ=1:SQ=-1;ID=S2506_raw:GT=hom:DP=141:FQ=1:SQ=-1;ID=S2667_raw:GT=hom:DP=149:FQ=1:SQ=-1;
17	3533310	3533310	T	C	intronic	SHPK	.	.	rs161362	.	ID=S2009_raw:GT=hom:DP=17:FQ=1:SQ=-1;ID=S2503_raw:GT=hom:DP=63:FQ=1:SQ=-1;ID=S2507_raw:GT=hom:DP=40:FQ=1:SQ=-1;ID=S2668_raw:GT=hom:DP=73:FQ=1:SQ=-1;ID=S2012_raw:GT=hom:DP=27:FQ=1:SQ=-1;ID=S2010_raw:GT=hom:DP=25:FQ=1:SQ=-1;ID=S2662_raw:GT=hom:DP=63:FQ=1:SQ=-1;ID=S2663_raw:GT=hom:DP=50:FQ=1:SQ=-1;ID=S2011_raw:GT=hom:DP=35:FQ=1:SQ=-1;ID=S2504_raw:GT=hom:DP=48:FQ=1:SQ=-1;ID=S2008_raw:GT=hom:DP=24:FQ=1:SQ=-1;ID=S2509_raw:GT=hom:DP=59:FQ=1:SQ=-1;ID=S2508_raw:GT=hom:DP=52:FQ=1:SQ=-1;ID=S2666_raw:GT=hom:DP=73:FQ=1:SQ=-1;ID=S2669_raw:GT=hom:DP=44:FQ=1:SQ=-1;ID=S2166_raw:GT=hom:DP=27:FQ=1:SQ=-1;ID=S1775_raw:GT=hom:DP=16:FQ=1:SQ=-1;ID=S2501_raw:GT=hom:DP=51:FQ=1:SQ=-1;ID=S2500_raw:GT=hom:DP=44:FQ=1:SQ=-1;ID=S2502_raw:GT=hom:DP=51:FQ=1:SQ=-1;ID=S2505_raw:GT=hom:DP=64:FQ=1:SQ=-1;ID=S2176_raw:GT=hom:DP=30:FQ=1:SQ=-1;ID=S2506_raw:GT=hom:DP=38:FQ=1:SQ=-1;ID=S2667_raw:GT=hom:DP=56:FQ=1:SQ=-1;
5	483002	483002	A	G	intronic	SLC9A3	.	.	rs2721030	.	ID=S2009_raw:GT=hom:DP=7:FQ=1:SQ=-1;ID=S2503_raw:GT=hom:DP=4:FQ=1:SQ=-1;ID=S2507_raw:GT=hom:DP=4:FQ=1:SQ=-1;ID=S2668_raw:GT=hom:DP=6:FQ=1:SQ=-1;ID=S2012_raw:GT=hom:DP=7:FQ=1:SQ=-1;ID=S2010_raw:GT=hom:DP=7:FQ=1:SQ=-1;ID=S2662_raw:GT=hom:DP=8:FQ=1:SQ=-1;ID=S2663_raw:GT=hom:DP=6:FQ=1:SQ=-1;ID=S2011_raw:GT=hom:DP=4:FQ=1:SQ=-1;ID=S2504_raw:GT=hom:DP=6:FQ=1:SQ=-1;ID=S2008_raw:GT=hom:DP=8:FQ=1:SQ=-1;ID=S2509_raw:GT=hom:DP=7:FQ=1:SQ=-1;ID=S2508_raw:GT=hom:DP=7:FQ=1:SQ=-1;ID=S2666_raw:GT=hom:DP=2:FQ=1:SQ=-1;ID=S2669_raw:GT=hom:DP=4:FQ=1:SQ=-1;ID=S2166_raw:GT=hom:DP=13:FQ=1:SQ=-1;ID=S1775_raw:GT=hom:DP=10:FQ=1:SQ=-1;ID=S2501_raw:GT=hom:DP=8:FQ=1:SQ=-1;ID=S2500_raw:GT=hom:DP=3:FQ=1:SQ=-1;ID=S2502_raw:GT=hom:DP=7:FQ=1:SQ=-1;ID=S2505_raw:GT=hom:DP=9:FQ=1:SQ=-1;ID=S2176_raw:GT=hom:DP=7:FQ=1:SQ=-1;ID=S2506_raw:GT=hom:DP=8:FQ=1:SQ=-1;ID=S2667_raw:GT=hom:DP=9:FQ=1:SQ=-1;
15	48538993	48538993	-	CAA	intronic	SLC12A1	.	.	.	.	ID=S2009_raw:GT=hom:DP=31:FQ=1:SQ=-1;ID=S2503_raw:GT=hom:DP=19:FQ=1:SQ=-1;ID=S2507_raw:GT=hom:DP=13:FQ=1:SQ=-1;ID=S2668_raw:GT=hom:DP=17:FQ=1:SQ=-1;ID=S2012_raw:GT=hom:DP=37:FQ=1:SQ=-1;ID=S2010_raw:GT=hom:DP=54:FQ=0.98:SQ=-1;ID=S2662_raw:GT=hom:DP=8:FQ=1:SQ=-1;ID=S2663_raw:GT=hom:DP=16:FQ=1:SQ=-1;ID=S2011_raw:GT=hom:DP=58:FQ=1:SQ=-1;ID=S2504_raw:GT=hom:DP=24:FQ=1:SQ=-1;ID=S2008_raw:GT=hom:DP=45:FQ=1:SQ=-1;ID=S2509_raw:GT=hom:DP=11:FQ=1:SQ=-1;ID=S2508_raw:GT=hom:DP=20:FQ=1:SQ=-1;ID=S2666_raw:GT=hom:DP=23:FQ=1:SQ=-1;ID=S2669_raw:GT=hom:DP=21:FQ=1:SQ=-1;ID=S2166_raw:GT=hom:DP=15:FQ=1:SQ=-1;ID=S1775_raw:GT=hom:DP=18:FQ=1:SQ=-1;ID=S2501_raw:GT=hom:DP=25:FQ=1:SQ=-1;ID=S2500_raw:GT=hom:DP=12:FQ=1:SQ=-1;ID=S2502_raw:GT=hom:DP=14:FQ=1:SQ=-1;ID=S2505_raw:GT=hom:DP=12:FQ=1:SQ=-1;ID=S2176_raw:GT=hom:DP=23:FQ=1:SQ=-1;ID=S2506_raw:GT=hom:DP=22:FQ=1:SQ=-1;ID=S2667_raw:GT=hom:DP=16:FQ=1:SQ=-1;
15	48539043	48539043	G	A	intronic	SLC12A1	.	.	rs8028035	.	ID=S2009_raw:GT=hom:DP=103:FQ=1:SQ=-1;ID=S2503_raw:GT=hom:DP=74:FQ=1:SQ=-1;ID=S2507_raw:GT=hom:DP=77:FQ=1:SQ=-1;ID=S2668_raw:GT=hom:DP=125:FQ=1:SQ=-1;ID=S2012_raw:GT=hom:DP=139:FQ=1:SQ=-1;ID=S2010_raw:GT=hom:DP=140:FQ=1:SQ=-1;ID=S2662_raw:GT=hom:DP=25:FQ=1:SQ=-1;ID=S2663_raw:GT=hom:DP=96:FQ=1:SQ=-1;ID=S2011_raw:GT=hom:DP=178:FQ=1:SQ=-1;ID=S2504_raw:GT=hom:DP=64:FQ=1:SQ=-1;ID=S2008_raw:GT=hom:DP=153:FQ=1:SQ=-1;ID=S2509_raw:GT=hom:DP=76:FQ=1:SQ=-1;ID=S2508_raw:GT=hom:DP=105:FQ=1:SQ=-1;ID=S2666_raw:GT=hom:DP=208:FQ=1:SQ=-1;ID=S2669_raw:GT=hom:DP=91:FQ=1:SQ=-1;ID=S2166_raw:GT=hom:DP=91:FQ=1:SQ=-1;ID=S1775_raw:GT=hom:DP=71:FQ=1:SQ=-1;ID=S2501_raw:GT=hom:DP=83:FQ=1:SQ=-1;ID=S2500_raw:GT=hom:DP=81:FQ=1:SQ=-1;ID=S2502_raw:GT=hom:DP=73:FQ=1:SQ=-1;ID=S2505_raw:GT=hom:DP=52:FQ=1:SQ=-1;ID=S2176_raw:GT=hom:DP=69:FQ=1:SQ=-1;ID=S2506_raw:GT=hom:DP=70:FQ=1:SQ=-1;ID=S2667_raw:GT=hom:DP=95:FQ=1:SQ=-1;
15	48561834	48561834	A	G	intronic	SLC12A1	.	.	rs1484551	.	ID=S2009_raw:GT=hom:DP=31:FQ=1:SQ=-1;ID=S2503_raw:GT=hom:DP=62:FQ=1:SQ=-1;ID=S2507_raw:GT=hom:DP=45:FQ=1:SQ=-1;ID=S2668_raw:GT=hom:DP=122:FQ=1:SQ=-1;ID=S2012_raw:GT=hom:DP=29:FQ=1:SQ=-1;ID=S2010_raw:GT=hom:DP=34:FQ=1:SQ=-1;ID=S2662_raw:GT=hom:DP=3:FQ=1:SQ=-1;ID=S2663_raw:GT=hom:DP=86:FQ=1:SQ=-1;ID=S2011_raw:GT=hom:DP=38:FQ=1:SQ=-1;ID=S2504_raw:GT=hom:DP=89:FQ=1:SQ=-1;ID=S2008_raw:GT=hom:DP=37:FQ=1:SQ=-1;ID=S2509_raw:GT=hom:DP=76:FQ=1:SQ=-1;ID=S2508_raw:GT=hom:DP=99:FQ=1:SQ=-1;ID=S2666_raw:GT=hom:DP=108:FQ=1:SQ=-1;ID=S2669_raw:GT=hom:DP=56:FQ=1:SQ=-1;ID=S2166_raw:GT=hom:DP=151:FQ=0.99:SQ=-1;ID=S1775_raw:GT=hom:DP=89:FQ=1:SQ=-1;ID=S2501_raw:GT=hom:DP=82:FQ=1:SQ=-1;ID=S2500_raw:GT=hom:DP=71:FQ=1:SQ=-1;ID=S2502_raw:GT=hom:DP=91:FQ=1:SQ=-1;ID=S2505_raw:GT=hom:DP=86:FQ=1:SQ=-1;ID=S2176_raw:GT=hom:DP=184:FQ=0.99:SQ=-1;ID=S2506_raw:GT=hom:DP=58:FQ=1:SQ=-1;ID=S2667_raw:GT=hom:DP=89:FQ=1:SQ=-1;
15	48580713	48580713	T	C	exonic;splicing	SLC12A1	nonsynonymous SNV	SLC12A1:NM_000338:exon23:c.2873T>C:p.V958A,SLC12A1:NM_001184832:exon23:c.2873T>C:p.V958A	rs1552311	Benign/Likely_benign	ID=S2009_raw:GT=hom:DP=74:FQ=1:SQ=-1;ID=S2503_raw:GT=hom:DP=81:FQ=1:SQ=-1;ID=S2507_raw:GT=hom:DP=83:FQ=1:SQ=-1;ID=S2668_raw:GT=hom:DP=115:FQ=1:SQ=-1;ID=S2012_raw:GT=hom:DP=75:FQ=1:SQ=-1;ID=S2010_raw:GT=hom:DP=103:FQ=1:SQ=-1;ID=S2662_raw:GT=hom:DP=1:FQ=1:SQ=-1;ID=S2663_raw:GT=hom:DP=107:FQ=1:SQ=-1;ID=S2011_raw:GT=hom:DP=142:FQ=1:SQ=-1;ID=S2504_raw:GT=hom:DP=92:FQ=1:SQ=-1;ID=S2008_raw:GT=hom:DP=97:FQ=1:SQ=-1;ID=S2509_raw:GT=hom:DP=91:FQ=1:SQ=-1;ID=S2508_raw:GT=hom:DP=86:FQ=1:SQ=-1;ID=S2666_raw:GT=hom:DP=129:FQ=1:SQ=-1;ID=S2669_raw:GT=hom:DP=43:FQ=1:SQ=-1;ID=S2166_raw:GT=hom:DP=74:FQ=1:SQ=-1;ID=S1775_raw:GT=hom:DP=43:FQ=1:SQ=-1;ID=S2501_raw:GT=hom:DP=71:FQ=1:SQ=-1;ID=S2500_raw:GT=hom:DP=92:FQ=1:SQ=-1;ID=S2502_raw:GT=hom:DP=80:FQ=1:SQ=-1;ID=S2505_raw:GT=hom:DP=92:FQ=1:SQ=-1;ID=S2176_raw:GT=hom:DP=80:FQ=1:SQ=-1;ID=S2506_raw:GT=hom:DP=70:FQ=1:SQ=-1;ID=S2667_raw:GT=hom:DP=85:FQ=1:SQ=-1;
11	64367325	64367325	A	G	exonic	SLC22A12	synonymous SNV	SLC22A12:NM_001276327:exon5:c.924A>G:p.A308A,SLC22A12:NM_001276326:exon7:c.1146A>G:p.A382A,SLC22A12:NM_144585:exon7:c.1248A>G:p.A416A,SLC22A12:NM_153378:exon7:c.585A>G:p.A195A	rs1630320	Benign	ID=S2009_raw:GT=hom:DP=64:FQ=1:SQ=-1;ID=S2503_raw:GT=hom:DP=120:FQ=1:SQ=-1;ID=S2507_raw:GT=hom:DP=100:FQ=1:SQ=-1;ID=S2668_raw:GT=hom:DP=93:FQ=1:SQ=-1;ID=S2012_raw:GT=hom:DP=71:FQ=1:SQ=-1;ID=S2010_raw:GT=hom:DP=70:FQ=1:SQ=-1;ID=S2662_raw:GT=hom:DP=227:FQ=1:SQ=-1;ID=S2663_raw:GT=hom:DP=105:FQ=1:SQ=-1;ID=S2011_raw:GT=hom:DP=89:FQ=1:SQ=-1;ID=S2504_raw:GT=hom:DP=115:FQ=1:SQ=-1;ID=S2008_raw:GT=hom:DP=66:FQ=1:SQ=-1;ID=S2509_raw:GT=hom:DP=97:FQ=1:SQ=-1;ID=S2508_raw:GT=hom:DP=133:FQ=1:SQ=-1;ID=S2666_raw:GT=hom:DP=79:FQ=0.99:SQ=-1;ID=S2669_raw:GT=hom:DP=105:FQ=1:SQ=-1;ID=S2166_raw:GT=hom:DP=70:FQ=1:SQ=-1;ID=S1775_raw:GT=hom:DP=76:FQ=1:SQ=-1;ID=S2501_raw:GT=hom:DP=109:FQ=1:SQ=-1;ID=S2500_raw:GT=hom:DP=113:FQ=1:SQ=-1;ID=S2502_raw:GT=hom:DP=130:FQ=1:SQ=-1;ID=S2505_raw:GT=hom:DP=114:FQ=1:SQ=-1;ID=S2176_raw:GT=hom:DP=82:FQ=1:SQ=-1;ID=S2506_raw:GT=hom:DP=104:FQ=1:SQ=-1;ID=S2667_raw:GT=hom:DP=165:FQ=1:SQ=-1;
11	1,25E+08	1,25E+08	A	G	intergenic	SLC37A2;TMEM218	.	dist=1833;dist=2021	rs10893320	.	ID=S2009_raw:GT=hom:DP=6:FQ=1:SQ=-1;ID=S2503_raw:GT=hom:DP=5:FQ=1:SQ=-1;ID=S2507_raw:GT=hom:DP=5:FQ=1:SQ=-1;ID=S2668_raw:GT=hom:DP=8:FQ=1:SQ=-1;ID=S2012_raw:GT=hom:DP=3:FQ=1:SQ=-1;ID=S2010_raw:GT=hom:DP=5:FQ=1:SQ=-1;ID=S2662_raw:GT=hom:DP=4:FQ=1:SQ=-1;ID=S2663_raw:GT=hom:DP=11:FQ=1:SQ=-1;ID=S2011_raw:GT=hom:DP=10:FQ=1:SQ=-1;ID=S2504_raw:GT=hom:DP=4:FQ=1:SQ=-1;ID=S2008_raw:GT=hom:DP=7:FQ=1:SQ=-1;ID=S2509_raw:GT=hom:DP=6:FQ=1:SQ=-1;ID=S2508_raw:GT=hom:DP=4:FQ=1:SQ=-1;ID=S2666_raw:GT=hom:DP=2:FQ=1:SQ=-1;ID=S2669_raw:GT=hom:DP=3:FQ=1:SQ=-1;ID=S2166_raw:GT=hom:DP=9:FQ=1:SQ=-1;ID=S1775_raw:GT=hom:DP=4:FQ=1:SQ=-1;ID=S2501_raw:GT=hom:DP=6:FQ=1:SQ=-1;ID=S2500_raw:GT=hom:DP=4:FQ=1:SQ=-1;ID=S2502_raw:GT=hom:DP=2:FQ=1:SQ=-1;ID=S2505_raw:GT=hom:DP=5:FQ=1:SQ=-1;ID=S2176_raw:GT=hom:DP=6:FQ=1:SQ=-1;ID=S2506_raw:GT=hom:DP=7:FQ=1:SQ=-1;ID=S2667_raw:GT=hom:DP=8:FQ=1:SQ=-1;
11	1,19E+08	1,19E+08	C	-	exonic;splicing	SLC37A4	frameshift deletion	SLC37A4:NM_001164280:exon3:c.527delG:p.C176fs,SLC37A4:NM_001467:exon4:c.527delG:p.C176fs,SLC37A4:NM_001164277:exon5:c.527delG:p.C176fs,SLC37A4:NM_001164278:exon5:c.527delG:p.C176fs,SLC37A4:NM_001164279:exon5:c.308delG:p.C103fs	.	.	ID=S2009_raw:GT=hom:DP=55:FQ=1:SQ=-1;ID=S2503_raw:GT=hom:DP=173:FQ=1:SQ=-1;ID=S2507_raw:GT=hom:DP=145:FQ=1:SQ=-1;ID=S2668_raw:GT=hom:DP=191:FQ=1:SQ=-1;ID=S2012_raw:GT=hom:DP=41:FQ=1:SQ=-1;ID=S2010_raw:GT=hom:DP=61:FQ=1:SQ=-1;ID=S2662_raw:GT=hom:DP=276:FQ=1:SQ=-1;ID=S2663_raw:GT=hom:DP=130:FQ=1:SQ=-1;ID=S2011_raw:GT=hom:DP=96:FQ=1:SQ=-1;ID=S2504_raw:GT=hom:DP=179:FQ=1:SQ=-1;ID=S2008_raw:GT=hom:DP=66:FQ=1:SQ=-1;ID=S2509_raw:GT=hom:DP=192:FQ=1:SQ=-1;ID=S2508_raw:GT=hom:DP=179:FQ=1:SQ=-1;ID=S2666_raw:GT=hom:DP=130:FQ=1:SQ=-1;ID=S2669_raw:GT=hom:DP=130:FQ=1:SQ=-1;ID=S2166_raw:GT=hom:DP=170:FQ=1:SQ=-1;ID=S1775_raw:GT=hom:DP=160:FQ=1:SQ=-1;ID=S2501_raw:GT=hom:DP=171:FQ=1:SQ=-1;ID=S2500_raw:GT=hom:DP=131:FQ=1:SQ=-1;ID=S2502_raw:GT=hom:DP=188:FQ=1:SQ=-1;ID=S2505_raw:GT=hom:DP=153:FQ=1:SQ=-1;ID=S2176_raw:GT=hom:DP=198:FQ=1:SQ=-1;ID=S2506_raw:GT=hom:DP=189:FQ=1:SQ=-1;ID=S2667_raw:GT=hom:DP=191:FQ=1:SQ=-1;
12	56575742	56575742	T	G	intronic	SMARCC2	.	.	rs773644	.	ID=S2009_raw:GT=hom:DP=71:FQ=1:SQ=-1;ID=S2503_raw:GT=hom:DP=71:FQ=1:SQ=-1;ID=S2507_raw:GT=hom:DP=37:FQ=1:SQ=-1;ID=S2668_raw:GT=hom:DP=52:FQ=1:SQ=-1;ID=S2012_raw:GT=hom:DP=94:FQ=1:SQ=-1;ID=S2010_raw:GT=hom:DP=104:FQ=1:SQ=-1;ID=S2662_raw:GT=hom:DP=34:FQ=1:SQ=-1;ID=S2663_raw:GT=hom:DP=49:FQ=1:SQ=-1;ID=S2011_raw:GT=hom:DP=116:FQ=1:SQ=-1;ID=S2504_raw:GT=hom:DP=68:FQ=1:SQ=-1;ID=S2008_raw:GT=hom:DP=95:FQ=1:SQ=-1;ID=S2509_raw:GT=hom:DP=40:FQ=1:SQ=-1;ID=S2508_raw:GT=hom:DP=63:FQ=1:SQ=-1;ID=S2666_raw:GT=hom:DP=65:FQ=1:SQ=-1;ID=S2669_raw:GT=hom:DP=40:FQ=1:SQ=-1;ID=S2166_raw:GT=hom:DP=108:FQ=1:SQ=-1;ID=S1775_raw:GT=hom:DP=93:FQ=1:SQ=-1;ID=S2501_raw:GT=hom:DP=47:FQ=1:SQ=-1;ID=S2500_raw:GT=hom:DP=59:FQ=1:SQ=-1;ID=S2502_raw:GT=hom:DP=48:FQ=1:SQ=-1;ID=S2505_raw:GT=hom:DP=51:FQ=1:SQ=-1;ID=S2176_raw:GT=hom:DP=104:FQ=1:SQ=-1;ID=S2506_raw:GT=hom:DP=64:FQ=1:SQ=-1;ID=S2667_raw:GT=hom:DP=47:FQ=1:SQ=-1;
X	53449338	53449338	-	G	intronic	SMC1A	.	.	.	.	ID=S2009_raw:GT=hom:DP=6:FQ=1:SQ=-1;ID=S2503_raw:GT=hom:DP=11:FQ=1:SQ=-1;ID=S2507_raw:GT=hom:DP=8:FQ=1:SQ=-1;ID=S2668_raw:GT=hom:DP=11:FQ=1:SQ=-1;ID=S2012_raw:GT=hom:DP=9:FQ=1:SQ=-1;ID=S2010_raw:GT=hom:DP=16:FQ=1:SQ=-1;ID=S2662_raw:GT=hom:DP=22:FQ=1:SQ=-1;ID=S2663_raw:GT=hom:DP=15:FQ=1:SQ=-1;ID=S2011_raw:GT=hom:DP=17:FQ=1:SQ=-1;ID=S2504_raw:GT=hom:DP=5:FQ=1:SQ=-1;ID=S2008_raw:GT=hom:DP=20:FQ=1:SQ=-1;ID=S2509_raw:GT=hom:DP=9:FQ=1:SQ=-1;ID=S2508_raw:GT=hom:DP=6:FQ=1:SQ=-1;ID=S2666_raw:GT=hom:DP=3:FQ=1:SQ=-1;ID=S2669_raw:GT=hom:DP=7:FQ=1:SQ=-1;ID=S2166_raw:GT=hom:DP=12:FQ=1:SQ=-1;ID=S1775_raw:GT=hom:DP=5:FQ=1:SQ=-1;ID=S2501_raw:GT=hom:DP=9:FQ=1:SQ=-1;ID=S2500_raw:GT=hom:DP=5:FQ=1:SQ=-1;ID=S2502_raw:GT=hom:DP=22:FQ=1:SQ=-1;ID=S2505_raw:GT=hom:DP=8:FQ=1:SQ=-1;ID=S2176_raw:GT=hom:DP=10:FQ=1:SQ=-1;ID=S2506_raw:GT=hom:DP=14:FQ=1:SQ=-1;ID=S2667_raw:GT=hom:DP=11:FQ=1:SQ=-1;
10	1,12E+08	1,12E+08	A	G	exonic	SMC3	synonymous SNV	SMC3:NM_005445:exon25:c.3039A>G:p.S1013S	rs2419565	Benign	ID=S2009_raw:GT=hom:DP=74:FQ=1:SQ=-1;ID=S2503_raw:GT=hom:DP=86:FQ=1:SQ=-1;ID=S2507_raw:GT=hom:DP=88:FQ=1:SQ=-1;ID=S2668_raw:GT=hom:DP=132:FQ=1:SQ=-1;ID=S2012_raw:GT=hom:DP=63:FQ=1:SQ=-1;ID=S2010_raw:GT=hom:DP=101:FQ=1:SQ=-1;ID=S2662_raw:GT=hom:DP=3:FQ=1:SQ=-1;ID=S2663_raw:GT=hom:DP=60:FQ=1:SQ=-1;ID=S2011_raw:GT=hom:DP=113:FQ=1:SQ=-1;ID=S2504_raw:GT=hom:DP=76:FQ=1:SQ=-1;ID=S2008_raw:GT=hom:DP=74:FQ=1:SQ=-1;ID=S2509_raw:GT=hom:DP=68:FQ=1:SQ=-1;ID=S2508_raw:GT=hom:DP=96:FQ=1:SQ=-1;ID=S2666_raw:GT=hom:DP=131:FQ=1:SQ=-1;ID=S2669_raw:GT=hom:DP=60:FQ=1:SQ=-1;ID=S2166_raw:GT=hom:DP=65:FQ=1:SQ=-1;ID=S1775_raw:GT=hom:DP=60:FQ=1:SQ=-1;ID=S2501_raw:GT=hom:DP=92:FQ=1:SQ=-1;ID=S2500_raw:GT=hom:DP=78:FQ=1:SQ=-1;ID=S2502_raw:GT=hom:DP=82:FQ=1:SQ=-1;ID=S2505_raw:GT=hom:DP=97:FQ=1:SQ=-1;ID=S2176_raw:GT=hom:DP=83:FQ=1:SQ=-1;ID=S2506_raw:GT=hom:DP=72:FQ=1:SQ=-1;ID=S2667_raw:GT=hom:DP=71:FQ=1:SQ=-1;
16	11350059	11350059	T	C	upstream	SOCS1	.	dist=20	rs27831	.	ID=S2009_raw:GT=hom:DP=10:FQ=1:SQ=-1;ID=S2503_raw:GT=hom:DP=21:FQ=1:SQ=-1;ID=S2507_raw:GT=hom:DP=12:FQ=1:SQ=-1;ID=S2668_raw:GT=hom:DP=21:FQ=1:SQ=-1;ID=S2012_raw:GT=hom:DP=4:FQ=1:SQ=-1;ID=S2010_raw:GT=hom:DP=8:FQ=1:SQ=-1;ID=S2662_raw:GT=hom:DP=50:FQ=1:SQ=-1;ID=S2663_raw:GT=hom:DP=13:FQ=1:SQ=-1;ID=S2011_raw:GT=hom:DP=5:FQ=1:SQ=-1;ID=S2504_raw:GT=hom:DP=14:FQ=1:SQ=-1;ID=S2008_raw:GT=hom:DP=8:FQ=1:SQ=-1;ID=S2509_raw:GT=hom:DP=16:FQ=1:SQ=-1;ID=S2508_raw:GT=hom:DP=33:FQ=1:SQ=-1;ID=S2666_raw:GT=hom:DP=8:FQ=1:SQ=-1;ID=S2669_raw:GT=hom:DP=23:FQ=1:SQ=-1;ID=S2166_raw:GT=hom:DP=19:FQ=1:SQ=-1;ID=S1775_raw:GT=hom:DP=2:FQ=1:SQ=-1;ID=S2501_raw:GT=hom:DP=19:FQ=1:SQ=-1;ID=S2500_raw:GT=hom:DP=29:FQ=1:SQ=-1;ID=S2502_raw:GT=hom:DP=9:FQ=1:SQ=-1;ID=S2505_raw:GT=hom:DP=26:FQ=1:SQ=-1;ID=S2176_raw:GT=hom:DP=16:FQ=1:SQ=-1;ID=S2506_raw:GT=hom:DP=20:FQ=1:SQ=-1;ID=S2667_raw:GT=hom:DP=26:FQ=1:SQ=-1;
21	34925142	34925142	C	T	exonic	SON	nonsynonymous SNV	SON:NM_001291411:exon3:c.3605C>T:p.S1202L,SON:NM_032195:exon3:c.3605C>T:p.S1202L,SON:NM_138927:exon3:c.3605C>T:p.S1202L	rs13433428	.	ID=S2009_raw:GT=hom:DP=130:FQ=1:SQ=-1;ID=S2503_raw:GT=hom:DP=118:FQ=1:SQ=-1;ID=S2507_raw:GT=hom:DP=88:FQ=1:SQ=-1;ID=S2668_raw:GT=hom:DP=159:FQ=1:SQ=-1;ID=S2012_raw:GT=hom:DP=156:FQ=1:SQ=-1;ID=S2010_raw:GT=hom:DP=198:FQ=1:SQ=-1;ID=S2662_raw:GT=hom:DP=109:FQ=1:SQ=-1;ID=S2663_raw:GT=hom:DP=88:FQ=1:SQ=-1;ID=S2011_raw:GT=hom:DP=250:FQ=1:SQ=-1;ID=S2504_raw:GT=hom:DP=166:FQ=1:SQ=-1;ID=S2008_raw:GT=hom:DP=165:FQ=1:SQ=-1;ID=S2509_raw:GT=hom:DP=104:FQ=1:SQ=-1;ID=S2508_raw:GT=hom:DP=120:FQ=1:SQ=-1;ID=S2666_raw:GT=hom:DP=140:FQ=1:SQ=-1;ID=S2669_raw:GT=hom:DP=91:FQ=1:SQ=-1;ID=S2166_raw:GT=hom:DP=112:FQ=1:SQ=-1;ID=S1775_raw:GT=hom:DP=75:FQ=1:SQ=-1;ID=S2501_raw:GT=hom:DP=110:FQ=1:SQ=-1;ID=S2500_raw:GT=hom:DP=95:FQ=1:SQ=-1;ID=S2502_raw:GT=hom:DP=135:FQ=1:SQ=-1;ID=S2505_raw:GT=hom:DP=113:FQ=1:SQ=-1;ID=S2176_raw:GT=hom:DP=147:FQ=1:SQ=-1;ID=S2506_raw:GT=hom:DP=86:FQ=1:SQ=-1;ID=S2667_raw:GT=hom:DP=108:FQ=1:SQ=-1;
21	34948685	34948685	-	A	exonic	SON	frameshift insertion	SON:NM_001291412:exon11:c.1320dupA:p.G440fs,SON:NM_138927:exon12:c.7236dupA:p.G2412fs	.	.	ID=S2009_raw:GT=hom:DP=4:FQ=1:SQ=-1;ID=S2503_raw:GT=hom:DP=84:FQ=1:SQ=-1;ID=S2507_raw:GT=hom:DP=63:FQ=1:SQ=-1;ID=S2668_raw:GT=hom:DP=78:FQ=1:SQ=-1;ID=S2012_raw:GT=hom:DP=4:FQ=1:SQ=-1;ID=S2010_raw:GT=hom:DP=7:FQ=1:SQ=-1;ID=S2662_raw:GT=hom:DP=3:FQ=1:SQ=-1;ID=S2663_raw:GT=hom:DP=100:FQ=1:SQ=-1;ID=S2011_raw:GT=hom:DP=7:FQ=1:SQ=-1;ID=S2504_raw:GT=hom:DP=91:FQ=1:SQ=-1;ID=S2008_raw:GT=hom:DP=5:FQ=1:SQ=-1;ID=S2509_raw:GT=hom:DP=110:FQ=1:SQ=-1;ID=S2508_raw:GT=hom:DP=81:FQ=1:SQ=-1;ID=S2666_raw:GT=hom:DP=61:FQ=1:SQ=-1;ID=S2669_raw:GT=hom:DP=26:FQ=1:SQ=-1;ID=S2166_raw:GT=hom:DP=81:FQ=1:SQ=-1;ID=S1775_raw:GT=hom:DP=57:FQ=1:SQ=-1;ID=S2501_raw:GT=hom:DP=80:FQ=1:SQ=-1;ID=S2500_raw:GT=hom:DP=72:FQ=1:SQ=-1;ID=S2502_raw:GT=hom:DP=83:FQ=1:SQ=-1;ID=S2505_raw:GT=hom:DP=82:FQ=1:SQ=-1;ID=S2176_raw:GT=hom:DP=67:FQ=1:SQ=-1;ID=S2506_raw:GT=hom:DP=65:FQ=1:SQ=-1;ID=S2667_raw:GT=hom:DP=76:FQ=1:SQ=-1;
22	24717850	24717850	A	G	exonic	SPECC1L	nonsynonymous SNV	SPECC1L:NM_001145468:exon4:c.902A>G:p.D301G,SPECC1L:NM_001254732:exon4:c.902A>G:p.D301G,SPECC1L:NM_015330:exon5:c.902A>G:p.D301G	rs204710	.	ID=S2009_raw:GT=hom:DP=149:FQ=1:SQ=-1;ID=S2503_raw:GT=hom:DP=138:FQ=1:SQ=-1;ID=S2507_raw:GT=hom:DP=127:FQ=1:SQ=-1;ID=S2668_raw:GT=hom:DP=165:FQ=1:SQ=-1;ID=S2012_raw:GT=hom:DP=206:FQ=1:SQ=-1;ID=S2010_raw:GT=hom:DP=188:FQ=1:SQ=-1;ID=S2662_raw:GT=hom:DP=90:FQ=1:SQ=-1;ID=S2663_raw:GT=hom:DP=110:FQ=1:SQ=-1;ID=S2011_raw:GT=hom:DP=273:FQ=1:SQ=-1;ID=S2504_raw:GT=hom:DP=152:FQ=1:SQ=-1;ID=S2008_raw:GT=hom:DP=171:FQ=1:SQ=-1;ID=S2509_raw:GT=hom:DP=117:FQ=1:SQ=-1;ID=S2508_raw:GT=hom:DP=149:FQ=1:SQ=-1;ID=S2666_raw:GT=hom:DP=188:FQ=1:SQ=-1;ID=S2669_raw:GT=hom:DP=108:FQ=1:SQ=-1;ID=S2166_raw:GT=hom:DP=180:FQ=0.99:SQ=-1;ID=S1775_raw:GT=hom:DP=132:FQ=1:SQ=-1;ID=S2501_raw:GT=hom:DP=120:FQ=1:SQ=-1;ID=S2500_raw:GT=hom:DP=130:FQ=1:SQ=-1;ID=S2502_raw:GT=hom:DP=159:FQ=1:SQ=-1;ID=S2505_raw:GT=hom:DP=141:FQ=1:SQ=-1;ID=S2176_raw:GT=hom:DP=213:FQ=1:SQ=-1;ID=S2506_raw:GT=hom:DP=111:FQ=1:SQ=-1;ID=S2667_raw:GT=hom:DP=145:FQ=1:SQ=-1;
22	24761467	24761467	G	A	exonic	SPECC1L	nonsynonymous SNV	SPECC1L:NM_001254733:exon5:c.49G>A:p.V17M,SPECC1L:NM_001145468:exon12:c.2851G>A:p.V951M,SPECC1L:NM_001254732:exon12:c.2851G>A:p.V951M,SPECC1L:NM_015330:exon13:c.2851G>A:p.V951M	rs204718	.	ID=S2009_raw:GT=hom:DP=44:FQ=1:SQ=-1;ID=S2503_raw:GT=hom:DP=107:FQ=1:SQ=-1;ID=S2507_raw:GT=hom:DP=93:FQ=1:SQ=-1;ID=S2668_raw:GT=hom:DP=124:FQ=1:SQ=-1;ID=S2012_raw:GT=hom:DP=48:FQ=1:SQ=-1;ID=S2010_raw:GT=hom:DP=43:FQ=1:SQ=-1;ID=S2662_raw:GT=hom:DP=50:FQ=1:SQ=-1;ID=S2663_raw:GT=hom:DP=102:FQ=1:SQ=-1;ID=S2011_raw:GT=hom:DP=52:FQ=1:SQ=-1;ID=S2504_raw:GT=hom:DP=118:FQ=0.99:SQ=-1;ID=S2008_raw:GT=hom:DP=53:FQ=1:SQ=-1;ID=S2509_raw:GT=hom:DP=99:FQ=1:SQ=-1;ID=S2508_raw:GT=hom:DP=135:FQ=1:SQ=-1;ID=S2666_raw:GT=hom:DP=152:FQ=1:SQ=-1;ID=S2669_raw:GT=hom:DP=101:FQ=1:SQ=-1;ID=S2166_raw:GT=hom:DP=138:FQ=1:SQ=-1;ID=S1775_raw:GT=hom:DP=89:FQ=1:SQ=-1;ID=S2501_raw:GT=hom:DP=94:FQ=1:SQ=-1;ID=S2500_raw:GT=hom:DP=115:FQ=1:SQ=-1;ID=S2502_raw:GT=hom:DP=110:FQ=1:SQ=-1;ID=S2505_raw:GT=hom:DP=98:FQ=1:SQ=-1;ID=S2176_raw:GT=hom:DP=148:FQ=1:SQ=-1;ID=S2506_raw:GT=hom:DP=80:FQ=1:SQ=-1;ID=S2667_raw:GT=hom:DP=69:FQ=0.99:SQ=-1;
5	35838933	35838933	A	C	intergenic	SPEF2;IL7R	.	dist=24220;dist=18060	rs4330488	.	ID=S2009_raw:GT=hom:DP=2:FQ=1:SQ=-1;ID=S2503_raw:GT=hom:DP=11:FQ=1:SQ=-1;ID=S2507_raw:GT=hom:DP=2:FQ=1:SQ=-1;ID=S2668_raw:GT=hom:DP=16:FQ=1:SQ=-1;ID=S2012_raw:GT=hom:DP=7:FQ=1:SQ=-1;ID=S2010_raw:GT=hom:DP=12:FQ=1:SQ=-1;ID=S2662_raw:GT=hom:DP=3:FQ=1:SQ=-1;ID=S2663_raw:GT=hom:DP=11:FQ=1:SQ=-1;ID=S2011_raw:GT=hom:DP=6:FQ=1:SQ=-1;ID=S2504_raw:GT=hom:DP=19:FQ=1:SQ=-1;ID=S2008_raw:GT=hom:DP=6:FQ=1:SQ=-1;ID=S2509_raw:GT=hom:DP=4:FQ=1:SQ=-1;ID=S2508_raw:GT=hom:DP=12:FQ=1:SQ=-1;ID=S2666_raw:GT=hom:DP=6:FQ=1:SQ=-1;ID=S2669_raw:GT=hom:DP=6:FQ=1:SQ=-1;ID=S2166_raw:GT=hom:DP=9:FQ=1:SQ=-1;ID=S1775_raw:GT=hom:DP=5:FQ=1:SQ=-1;ID=S2501_raw:GT=hom:DP=5:FQ=1:SQ=-1;ID=S2500_raw:GT=hom:DP=6:FQ=1:SQ=-1;ID=S2502_raw:GT=hom:DP=7:FQ=1:SQ=-1;ID=S2505_raw:GT=hom:DP=3:FQ=1:SQ=-1;ID=S2176_raw:GT=hom:DP=12:FQ=1:SQ=-1;ID=S2506_raw:GT=hom:DP=8:FQ=1:SQ=-1;ID=S2667_raw:GT=hom:DP=2:FQ=1:SQ=-1;
15	38590722	38590722	T	G	intronic	SPRED1	.	.	rs8040553	.	ID=S2009_raw:GT=hom:DP=20:FQ=1:SQ=-1;ID=S2503_raw:GT=hom:DP=34:FQ=1:SQ=-1;ID=S2507_raw:GT=hom:DP=45:FQ=1:SQ=-1;ID=S2668_raw:GT=hom:DP=48:FQ=1:SQ=-1;ID=S2012_raw:GT=hom:DP=25:FQ=1:SQ=-1;ID=S2010_raw:GT=hom:DP=35:FQ=1:SQ=-1;ID=S2662_raw:GT=hom:DP=5:FQ=1:SQ=-1;ID=S2663_raw:GT=hom:DP=41:FQ=1:SQ=-1;ID=S2011_raw:GT=hom:DP=44:FQ=1:SQ=-1;ID=S2504_raw:GT=hom:DP=36:FQ=1:SQ=-1;ID=S2008_raw:GT=hom:DP=27:FQ=1:SQ=-1;ID=S2509_raw:GT=hom:DP=43:FQ=1:SQ=-1;ID=S2508_raw:GT=hom:DP=34:FQ=1:SQ=-1;ID=S2666_raw:GT=hom:DP=44:FQ=1:SQ=-1;ID=S2669_raw:GT=hom:DP=8:FQ=1:SQ=-1;ID=S2166_raw:GT=hom:DP=34:FQ=1:SQ=-1;ID=S1775_raw:GT=hom:DP=18:FQ=1:SQ=-1;ID=S2501_raw:GT=hom:DP=53:FQ=1:SQ=-1;ID=S2500_raw:GT=hom:DP=33:FQ=1:SQ=-1;ID=S2502_raw:GT=hom:DP=45:FQ=1:SQ=-1;ID=S2505_raw:GT=hom:DP=44:FQ=1:SQ=-1;ID=S2176_raw:GT=hom:DP=38:FQ=1:SQ=-1;ID=S2506_raw:GT=hom:DP=29:FQ=1:SQ=-1;ID=S2667_raw:GT=hom:DP=31:FQ=1:SQ=-1;
15	38632148	38632148	A	G	intronic	SPRED1	.	.	rs7181472	Benign	ID=S2009_raw:GT=hom:DP=10:FQ=1:SQ=-1;ID=S2503_raw:GT=hom:DP=26:FQ=1:SQ=-1;ID=S2507_raw:GT=hom:DP=27:FQ=1:SQ=-1;ID=S2668_raw:GT=hom:DP=16:FQ=1:SQ=-1;ID=S2012_raw:GT=hom:DP=36:FQ=1:SQ=-1;ID=S2010_raw:GT=hom:DP=25:FQ=1:SQ=-1;ID=S2662_raw:GT=hom:DP=1:FQ=1:SQ=-1;ID=S2663_raw:GT=hom:DP=23:FQ=1:SQ=-1;ID=S2011_raw:GT=hom:DP=37:FQ=1:SQ=-1;ID=S2504_raw:GT=hom:DP=36:FQ=1:SQ=-1;ID=S2008_raw:GT=hom:DP=31:FQ=1:SQ=-1;ID=S2509_raw:GT=hom:DP=24:FQ=1:SQ=-1;ID=S2508_raw:GT=hom:DP=11:FQ=1:SQ=-1;ID=S2666_raw:GT=hom:DP=17:FQ=1:SQ=-1;ID=S2669_raw:GT=hom:DP=27:FQ=1:SQ=-1;ID=S2166_raw:GT=hom:DP=50:FQ=1:SQ=-1;ID=S1775_raw:GT=hom:DP=20:FQ=1:SQ=-1;ID=S2501_raw:GT=hom:DP=23:FQ=1:SQ=-1;ID=S2500_raw:GT=hom:DP=25:FQ=1:SQ=-1;ID=S2502_raw:GT=hom:DP=14:FQ=1:SQ=-1;ID=S2505_raw:GT=hom:DP=37:FQ=1:SQ=-1;ID=S2176_raw:GT=hom:DP=46:FQ=1:SQ=-1;ID=S2506_raw:GT=hom:DP=17:FQ=1:SQ=-1;ID=S2667_raw:GT=hom:DP=22:FQ=1:SQ=-1;
20	36030939	36030939	G	C	exonic	SRC	synonymous SNV	SRC:NM_005417:exon12:c.1218G>C:p.A406A,SRC:NM_198291:exon12:c.1218G>C:p.A406A	rs1885257	.	ID=S2009_raw:GT=hom:DP=97:FQ=1:SQ=-1;ID=S2503_raw:GT=hom:DP=124:FQ=1:SQ=-1;ID=S2507_raw:GT=hom:DP=138:FQ=1:SQ=-1;ID=S2668_raw:GT=hom:DP=143:FQ=1:SQ=-1;ID=S2012_raw:GT=hom:DP=63:FQ=1:SQ=-1;ID=S2010_raw:GT=hom:DP=99:FQ=1:SQ=-1;ID=S2662_raw:GT=hom:DP=224:FQ=1:SQ=-1;ID=S2663_raw:GT=hom:DP=110:FQ=1:SQ=-1;ID=S2011_raw:GT=hom:DP=123:FQ=1:SQ=-1;ID=S2504_raw:GT=hom:DP=142:FQ=1:SQ=-1;ID=S2008_raw:GT=hom:DP=109:FQ=1:SQ=-1;ID=S2509_raw:GT=hom:DP=135:FQ=1:SQ=-1;ID=S2508_raw:GT=hom:DP=167:FQ=1:SQ=-1;ID=S2666_raw:GT=hom:DP=152:FQ=1:SQ=-1;ID=S2669_raw:GT=hom:DP=133:FQ=1:SQ=-1;ID=S2166_raw:GT=hom:DP=120:FQ=1:SQ=-1;ID=S1775_raw:GT=hom:DP=79:FQ=1:SQ=-1;ID=S2501_raw:GT=hom:DP=130:FQ=1:SQ=-1;ID=S2500_raw:GT=hom:DP=129:FQ=1:SQ=-1;ID=S2502_raw:GT=hom:DP=130:FQ=1:SQ=-1;ID=S2505_raw:GT=hom:DP=140:FQ=1:SQ=-1;ID=S2176_raw:GT=hom:DP=145:FQ=1:SQ=-1;ID=S2506_raw:GT=hom:DP=150:FQ=1:SQ=-1;ID=S2667_raw:GT=hom:DP=123:FQ=1:SQ=-1;
20	36031097	36031097	A	G	intronic	SRC	.	.	rs2145792	.	ID=S2009_raw:GT=hom:DP=95:FQ=1:SQ=-1;ID=S2503_raw:GT=hom:DP=151:FQ=1:SQ=-1;ID=S2507_raw:GT=hom:DP=146:FQ=1:SQ=-1;ID=S2668_raw:GT=hom:DP=202:FQ=1:SQ=-1;ID=S2012_raw:GT=hom:DP=85:FQ=1:SQ=-1;ID=S2010_raw:GT=hom:DP=89:FQ=1:SQ=-1;ID=S2662_raw:GT=hom:DP=260:FQ=1:SQ=-1;ID=S2663_raw:GT=hom:DP=145:FQ=1:SQ=-1;ID=S2011_raw:GT=hom:DP=132:FQ=1:SQ=-1;ID=S2504_raw:GT=hom:DP=183:FQ=1:SQ=-1;ID=S2008_raw:GT=hom:DP=78:FQ=1:SQ=-1;ID=S2509_raw:GT=hom:DP=155:FQ=1:SQ=-1;ID=S2508_raw:GT=hom:DP=191:FQ=1:SQ=-1;ID=S2666_raw:GT=hom:DP=153:FQ=1:SQ=-1;ID=S2669_raw:GT=hom:DP=135:FQ=1:SQ=-1;ID=S2166_raw:GT=hom:DP=138:FQ=1:SQ=-1;ID=S1775_raw:GT=hom:DP=76:FQ=1:SQ=-1;ID=S2501_raw:GT=hom:DP=151:FQ=1:SQ=-1;ID=S2500_raw:GT=hom:DP=161:FQ=1:SQ=-1;ID=S2502_raw:GT=hom:DP=198:FQ=1:SQ=-1;ID=S2505_raw:GT=hom:DP=165:FQ=1:SQ=-1;ID=S2176_raw:GT=hom:DP=150:FQ=1:SQ=-1;ID=S2506_raw:GT=hom:DP=152:FQ=1:SQ=-1;ID=S2667_raw:GT=hom:DP=176:FQ=1:SQ=-1;
16	30724070	30724070	G	T	exonic	SRCAP	synonymous SNV	SRCAP:NM_006662:exon14:c.2064G>T:p.R688R	rs4889500	.	ID=S2009_raw:GT=hom:DP=49:FQ=1:SQ=-1;ID=S2503_raw:GT=hom:DP=88:FQ=1:SQ=-1;ID=S2507_raw:GT=hom:DP=102:FQ=1:SQ=-1;ID=S2668_raw:GT=hom:DP=116:FQ=1:SQ=-1;ID=S2012_raw:GT=hom:DP=45:FQ=1:SQ=-1;ID=S2010_raw:GT=hom:DP=39:FQ=1:SQ=-1;ID=S2662_raw:GT=hom:DP=93:FQ=1:SQ=-1;ID=S2663_raw:GT=hom:DP=96:FQ=1:SQ=-1;ID=S2011_raw:GT=hom:DP=71:FQ=1:SQ=-1;ID=S2504_raw:GT=hom:DP=133:FQ=1:SQ=-1;ID=S2008_raw:GT=hom:DP=66:FQ=1:SQ=-1;ID=S2509_raw:GT=hom:DP=109:FQ=1:SQ=-1;ID=S2508_raw:GT=hom:DP=102:FQ=1:SQ=-1;ID=S2666_raw:GT=hom:DP=122:FQ=1:SQ=-1;ID=S2669_raw:GT=hom:DP=80:FQ=1:SQ=-1;ID=S2166_raw:GT=hom:DP=120:FQ=1:SQ=-1;ID=S1775_raw:GT=hom:DP=63:FQ=1:SQ=-1;ID=S2501_raw:GT=hom:DP=125:FQ=1:SQ=-1;ID=S2500_raw:GT=hom:DP=100:FQ=1:SQ=-1;ID=S2502_raw:GT=hom:DP=112:FQ=1:SQ=-1;ID=S2505_raw:GT=hom:DP=126:FQ=1:SQ=-1;ID=S2176_raw:GT=hom:DP=171:FQ=1:SQ=-1;ID=S2506_raw:GT=hom:DP=113:FQ=1:SQ=-1;ID=S2667_raw:GT=hom:DP=92:FQ=1:SQ=-1;
16	30724221	30724221	C	T	intronic	SRCAP	.	.	rs4889501	.	ID=S2009_raw:GT=hom:DP=4:FQ=1:SQ=-1;ID=S2503_raw:GT=hom:DP=17:FQ=1:SQ=-1;ID=S2507_raw:GT=hom:DP=19:FQ=1:SQ=-1;ID=S2668_raw:GT=hom:DP=20:FQ=1:SQ=-1;ID=S2012_raw:GT=hom:DP=6:FQ=1:SQ=-1;ID=S2010_raw:GT=hom:DP=6:FQ=1:SQ=-1;ID=S2662_raw:GT=hom:DP=16:FQ=1:SQ=-1;ID=S2663_raw:GT=hom:DP=12:FQ=1:SQ=-1;ID=S2011_raw:GT=hom:DP=12:FQ=1:SQ=-1;ID=S2504_raw:GT=hom:DP=30:FQ=0.97:SQ=-1;ID=S2008_raw:GT=hom:DP=4:FQ=1:SQ=-1;ID=S2509_raw:GT=hom:DP=23:FQ=1:SQ=-1;ID=S2508_raw:GT=hom:DP=16:FQ=1:SQ=-1;ID=S2666_raw:GT=hom:DP=18:FQ=1:SQ=-1;ID=S2669_raw:GT=hom:DP=17:FQ=1:SQ=-1;ID=S2166_raw:GT=hom:DP=30:FQ=1:SQ=-1;ID=S1775_raw:GT=hom:DP=9:FQ=1:SQ=-1;ID=S2501_raw:GT=hom:DP=21:FQ=1:SQ=-1;ID=S2500_raw:GT=hom:DP=13:FQ=1:SQ=-1;ID=S2502_raw:GT=hom:DP=23:FQ=1:SQ=-1;ID=S2505_raw:GT=hom:DP=22:FQ=1:SQ=-1;ID=S2176_raw:GT=hom:DP=32:FQ=1:SQ=-1;ID=S2506_raw:GT=hom:DP=27:FQ=1:SQ=-1;ID=S2667_raw:GT=hom:DP=30:FQ=1:SQ=-1;
16	30732406	30732406	C	G	intronic	SRCAP	.	.	rs1470129	.	ID=S2009_raw:GT=hom:DP=26:FQ=1:SQ=-1;ID=S2503_raw:GT=hom:DP=40:FQ=1:SQ=-1;ID=S2507_raw:GT=hom:DP=58:FQ=1:SQ=-1;ID=S2668_raw:GT=hom:DP=34:FQ=1:SQ=-1;ID=S2012_raw:GT=hom:DP=20:FQ=1:SQ=-1;ID=S2010_raw:GT=hom:DP=21:FQ=1:SQ=-1;ID=S2662_raw:GT=hom:DP=34:FQ=1:SQ=-1;ID=S2663_raw:GT=hom:DP=20:FQ=1:SQ=-1;ID=S2011_raw:GT=hom:DP=36:FQ=1:SQ=-1;ID=S2504_raw:GT=hom:DP=49:FQ=1:SQ=-1;ID=S2008_raw:GT=hom:DP=34:FQ=1:SQ=-1;ID=S2509_raw:GT=hom:DP=26:FQ=1:SQ=-1;ID=S2508_raw:GT=hom:DP=62:FQ=1:SQ=-1;ID=S2666_raw:GT=hom:DP=28:FQ=1:SQ=-1;ID=S2669_raw:GT=hom:DP=28:FQ=0.96:SQ=-1;ID=S2166_raw:GT=hom:DP=54:FQ=1:SQ=-1;ID=S1775_raw:GT=hom:DP=50:FQ=1:SQ=-1;ID=S2501_raw:GT=hom:DP=51:FQ=1:SQ=-1;ID=S2500_raw:GT=hom:DP=45:FQ=1:SQ=-1;ID=S2502_raw:GT=hom:DP=64:FQ=1:SQ=-1;ID=S2505_raw:GT=hom:DP=43:FQ=1:SQ=-1;ID=S2176_raw:GT=hom:DP=42:FQ=1:SQ=-1;ID=S2506_raw:GT=hom:DP=45:FQ=1:SQ=-1;ID=S2667_raw:GT=hom:DP=35:FQ=1:SQ=-1;
16	30734963	30734963	C	A	exonic	SRCAP	synonymous SNV	SRCAP:NM_006662:exon25:c.4218C>A:p.S1406S	rs4889502	Benign	ID=S2009_raw:GT=hom:DP=166:FQ=1:SQ=-1;ID=S2503_raw:GT=hom:DP=108:FQ=0.99:SQ=-1;ID=S2507_raw:GT=hom:DP=97:FQ=1:SQ=-1;ID=S2668_raw:GT=hom:DP=95:FQ=1:SQ=-1;ID=S2012_raw:GT=hom:DP=176:FQ=1:SQ=-1;ID=S2010_raw:GT=hom:DP=188:FQ=1:SQ=-1;ID=S2662_raw:GT=hom:DP=81:FQ=1:SQ=-1;ID=S2663_raw:GT=hom:DP=94:FQ=1:SQ=-1;ID=S2011_raw:GT=hom:DP=210:FQ=1:SQ=-1;ID=S2504_raw:GT=hom:DP=119:FQ=1:SQ=-1;ID=S2008_raw:GT=hom:DP=146:FQ=1:SQ=-1;ID=S2509_raw:GT=hom:DP=92:FQ=1:SQ=-1;ID=S2508_raw:GT=hom:DP=101:FQ=1:SQ=-1;ID=S2666_raw:GT=hom:DP=104:FQ=0.99:SQ=-1;ID=S2669_raw:GT=hom:DP=61:FQ=1:SQ=-1;ID=S2166_raw:GT=hom:DP=243:FQ=0.99:SQ=-1;ID=S1775_raw:GT=hom:DP=166:FQ=1:SQ=-1;ID=S2501_raw:GT=hom:DP=83:FQ=1:SQ=-1;ID=S2500_raw:GT=hom:DP=101:FQ=1:SQ=-1;ID=S2502_raw:GT=hom:DP=103:FQ=1:SQ=-1;ID=S2505_raw:GT=hom:DP=94:FQ=1:SQ=-1;ID=S2176_raw:GT=hom:DP=226:FQ=1:SQ=-1;ID=S2506_raw:GT=hom:DP=91:FQ=1:SQ=-1;ID=S2667_raw:GT=hom:DP=84:FQ=1:SQ=-1;
X	1,53E+08	1,53E+08	T	C	intronic	SSR4	.	.	rs5945165	.	ID=S2009_raw:GT=hom:DP=1:FQ=1:SQ=-1;ID=S2503_raw:GT=hom:DP=15:FQ=1:SQ=-1;ID=S2507_raw:GT=hom:DP=4:FQ=1:SQ=-1;ID=S2668_raw:GT=hom:DP=14:FQ=1:SQ=-1;ID=S2012_raw:GT=hom:DP=1:FQ=1:SQ=-1;ID=S2010_raw:GT=hom:DP=6:FQ=1:SQ=-1;ID=S2662_raw:GT=hom:DP=22:FQ=1:SQ=-1;ID=S2663_raw:GT=hom:DP=19:FQ=1:SQ=-1;ID=S2011_raw:GT=hom:DP=7:FQ=1:SQ=-1;ID=S2504_raw:GT=hom:DP=8:FQ=1:SQ=-1;ID=S2008_raw:GT=hom:DP=4:FQ=1:SQ=-1;ID=S2509_raw:GT=hom:DP=7:FQ=1:SQ=-1;ID=S2508_raw:GT=hom:DP=11:FQ=1:SQ=-1;ID=S2666_raw:GT=hom:DP=9:FQ=1:SQ=-1;ID=S2669_raw:GT=hom:DP=7:FQ=1:SQ=-1;ID=S2166_raw:GT=hom:DP=8:FQ=1:SQ=-1;ID=S1775_raw:GT=hom:DP=5:FQ=1:SQ=-1;ID=S2501_raw:GT=hom:DP=4:FQ=1:SQ=-1;ID=S2500_raw:GT=hom:DP=11:FQ=1:SQ=-1;ID=S2502_raw:GT=hom:DP=19:FQ=1:SQ=-1;ID=S2505_raw:GT=hom:DP=9:FQ=1:SQ=-1;ID=S2176_raw:GT=hom:DP=6:FQ=1:SQ=-1;ID=S2506_raw:GT=hom:DP=7:FQ=1:SQ=-1;ID=S2667_raw:GT=hom:DP=14:FQ=1:SQ=-1;
2	1,92E+08	1,92E+08	T	C	intronic	STAT1	.	.	rs12477076	.	ID=S2009_raw:GT=hom:DP=13:FQ=1:SQ=-1;ID=S2503_raw:GT=hom:DP=33:FQ=1:SQ=-1;ID=S2507_raw:GT=hom:DP=23:FQ=1:SQ=-1;ID=S2668_raw:GT=hom:DP=43:FQ=1:SQ=-1;ID=S2012_raw:GT=hom:DP=17:FQ=1:SQ=-1;ID=S2010_raw:GT=hom:DP=9:FQ=1:SQ=-1;ID=S2662_raw:GT=hom:DP=15:FQ=1:SQ=-1;ID=S2663_raw:GT=hom:DP=26:FQ=1:SQ=-1;ID=S2011_raw:GT=hom:DP=14:FQ=1:SQ=-1;ID=S2504_raw:GT=hom:DP=33:FQ=1:SQ=-1;ID=S2008_raw:GT=hom:DP=13:FQ=1:SQ=-1;ID=S2509_raw:GT=hom:DP=22:FQ=1:SQ=-1;ID=S2508_raw:GT=hom:DP=28:FQ=1:SQ=-1;ID=S2666_raw:GT=hom:DP=18:FQ=1:SQ=-1;ID=S2669_raw:GT=hom:DP=23:FQ=1:SQ=-1;ID=S2166_raw:GT=hom:DP=57:FQ=1:SQ=-1;ID=S1775_raw:GT=hom:DP=24:FQ=1:SQ=-1;ID=S2501_raw:GT=hom:DP=42:FQ=1:SQ=-1;ID=S2500_raw:GT=hom:DP=38:FQ=1:SQ=-1;ID=S2502_raw:GT=hom:DP=26:FQ=1:SQ=-1;ID=S2505_raw:GT=hom:DP=32:FQ=1:SQ=-1;ID=S2176_raw:GT=hom:DP=47:FQ=1:SQ=-1;ID=S2506_raw:GT=hom:DP=27:FQ=1:SQ=-1;ID=S2667_raw:GT=hom:DP=32:FQ=1:SQ=-1;
15	74489883	74489883	A	G	intronic	STRA6	.	.	rs351218	.	ID=S2009_raw:GT=hom:DP=4:FQ=1:SQ=-1;ID=S2503_raw:GT=hom:DP=18:FQ=1:SQ=-1;ID=S2507_raw:GT=hom:DP=19:FQ=1:SQ=-1;ID=S2668_raw:GT=hom:DP=17:FQ=1:SQ=-1;ID=S2012_raw:GT=hom:DP=2:FQ=1:SQ=-1;ID=S2010_raw:GT=hom:DP=5:FQ=1:SQ=-1;ID=S2662_raw:GT=hom:DP=29:FQ=1:SQ=-1;ID=S2663_raw:GT=hom:DP=27:FQ=1:SQ=-1;ID=S2011_raw:GT=hom:DP=11:FQ=1:SQ=-1;ID=S2504_raw:GT=hom:DP=23:FQ=1:SQ=-1;ID=S2008_raw:GT=hom:DP=12:FQ=1:SQ=-1;ID=S2509_raw:GT=hom:DP=20:FQ=1:SQ=-1;ID=S2508_raw:GT=hom:DP=24:FQ=1:SQ=-1;ID=S2666_raw:GT=hom:DP=24:FQ=1:SQ=-1;ID=S2669_raw:GT=hom:DP=11:FQ=1:SQ=-1;ID=S2166_raw:GT=hom:DP=23:FQ=1:SQ=-1;ID=S1775_raw:GT=hom:DP=10:FQ=1:SQ=-1;ID=S2501_raw:GT=hom:DP=22:FQ=1:SQ=-1;ID=S2500_raw:GT=hom:DP=10:FQ=1:SQ=-1;ID=S2502_raw:GT=hom:DP=11:FQ=1:SQ=-1;ID=S2505_raw:GT=hom:DP=18:FQ=1:SQ=-1;ID=S2176_raw:GT=hom:DP=18:FQ=1:SQ=-1;ID=S2506_raw:GT=hom:DP=15:FQ=1:SQ=-1;ID=S2667_raw:GT=hom:DP=20:FQ=1:SQ=-1;
17	61805401	61805401	T	G	intronic	STRADA	.	.	rs2137143	.	ID=S2009_raw:GT=hom:DP=2:FQ=1:SQ=-1;ID=S2503_raw:GT=hom:DP=5:FQ=1:SQ=-1;ID=S2507_raw:GT=hom:DP=5:FQ=1:SQ=-1;ID=S2668_raw:GT=hom:DP=11:FQ=1:SQ=-1;ID=S2012_raw:GT=hom:DP=3:FQ=1:SQ=-1;ID=S2010_raw:GT=hom:DP=2:FQ=1:SQ=-1;ID=S2662_raw:GT=hom:DP=1:FQ=1:SQ=-1;ID=S2663_raw:GT=hom:DP=9:FQ=1:SQ=-1;ID=S2011_raw:GT=hom:DP=1:FQ=1:SQ=-1;ID=S2504_raw:GT=hom:DP=10:FQ=1:SQ=-1;ID=S2008_raw:GT=hom:DP=4:FQ=1:SQ=-1;ID=S2509_raw:GT=hom:DP=4:FQ=1:SQ=-1;ID=S2508_raw:GT=hom:DP=4:FQ=1:SQ=-1;ID=S2666_raw:GT=hom:DP=5:FQ=1:SQ=-1;ID=S2669_raw:GT=hom:DP=2:FQ=1:SQ=-1;ID=S2166_raw:GT=hom:DP=7:FQ=1:SQ=-1;ID=S1775_raw:GT=hom:DP=4:FQ=1:SQ=-1;ID=S2501_raw:GT=hom:DP=5:FQ=1:SQ=-1;ID=S2500_raw:GT=hom:DP=2:FQ=1:SQ=-1;ID=S2502_raw:GT=hom:DP=9:FQ=1:SQ=-1;ID=S2505_raw:GT=hom:DP=8:FQ=1:SQ=-1;ID=S2176_raw:GT=hom:DP=12:FQ=1:SQ=-1;ID=S2506_raw:GT=hom:DP=1:FQ=1:SQ=-1;ID=S2667_raw:GT=hom:DP=8:FQ=1:SQ=-1;
7	73118398	73118398	T	C	intronic	STX1A	.	.	rs7807316	.	ID=S2009_raw:GT=hom:DP=11:FQ=1:SQ=-1;ID=S2503_raw:GT=hom:DP=13:FQ=1:SQ=-1;ID=S2507_raw:GT=hom:DP=26:FQ=1:SQ=-1;ID=S2668_raw:GT=hom:DP=5:FQ=1:SQ=-1;ID=S2012_raw:GT=hom:DP=15:FQ=1:SQ=-1;ID=S2010_raw:GT=hom:DP=16:FQ=1:SQ=-1;ID=S2662_raw:GT=hom:DP=36:FQ=1:SQ=-1;ID=S2663_raw:GT=hom:DP=21:FQ=1:SQ=-1;ID=S2011_raw:GT=hom:DP=23:FQ=1:SQ=-1;ID=S2504_raw:GT=hom:DP=16:FQ=1:SQ=-1;ID=S2008_raw:GT=hom:DP=18:FQ=1:SQ=-1;ID=S2509_raw:GT=hom:DP=4:FQ=1:SQ=-1;ID=S2508_raw:GT=hom:DP=15:FQ=1:SQ=-1;ID=S2666_raw:GT=hom:DP=1:FQ=1:SQ=-1;ID=S2669_raw:GT=hom:DP=16:FQ=1:SQ=-1;ID=S2166_raw:GT=hom:DP=14:FQ=1:SQ=-1;ID=S1775_raw:GT=hom:DP=8:FQ=1:SQ=-1;ID=S2501_raw:GT=hom:DP=12:FQ=1:SQ=-1;ID=S2500_raw:GT=hom:DP=12:FQ=1:SQ=-1;ID=S2502_raw:GT=hom:DP=15:FQ=1:SQ=-1;ID=S2505_raw:GT=hom:DP=24:FQ=1:SQ=-1;ID=S2176_raw:GT=hom:DP=14:FQ=1:SQ=-1;ID=S2506_raw:GT=hom:DP=21:FQ=1:SQ=-1;ID=S2667_raw:GT=hom:DP=17:FQ=1:SQ=-1;
10	1,04E+08	1,04E+08	G	C	intronic	SUFU	.	.	rs10748822	.	ID=S2009_raw:GT=hom:DP=9:FQ=1:SQ=-1;ID=S2503_raw:GT=hom:DP=37:FQ=1:SQ=-1;ID=S2507_raw:GT=hom:DP=37:FQ=1:SQ=-1;ID=S2668_raw:GT=hom:DP=49:FQ=1:SQ=-1;ID=S2012_raw:GT=hom:DP=21:FQ=1:SQ=-1;ID=S2010_raw:GT=hom:DP=34:FQ=1:SQ=-1;ID=S2662_raw:GT=hom:DP=45:FQ=1:SQ=-1;ID=S2663_raw:GT=hom:DP=44:FQ=1:SQ=-1;ID=S2011_raw:GT=hom:DP=24:FQ=1:SQ=-1;ID=S2504_raw:GT=hom:DP=56:FQ=1:SQ=-1;ID=S2008_raw:GT=hom:DP=18:FQ=1:SQ=-1;ID=S2509_raw:GT=hom:DP=60:FQ=0.98:SQ=-1;ID=S2508_raw:GT=hom:DP=57:FQ=1:SQ=-1;ID=S2666_raw:GT=hom:DP=59:FQ=1:SQ=-1;ID=S2669_raw:GT=hom:DP=36:FQ=1:SQ=-1;ID=S2166_raw:GT=hom:DP=3:FQ=1:SQ=-1;ID=S1775_raw:GT=hom:DP=2:FQ=1:SQ=-1;ID=S2501_raw:GT=hom:DP=43:FQ=1:SQ=-1;ID=S2500_raw:GT=hom:DP=47:FQ=1:SQ=-1;ID=S2502_raw:GT=hom:DP=56:FQ=1:SQ=-1;ID=S2505_raw:GT=hom:DP=48:FQ=1:SQ=-1;ID=S2176_raw:GT=hom:DP=4:FQ=1:SQ=-1;ID=S2506_raw:GT=hom:DP=34:FQ=1:SQ=-1;ID=S2667_raw:GT=hom:DP=53:FQ=1:SQ=-1;
10	1,04E+08	1,04E+08	A	G	intronic	SUFU	.	.	rs10786690	.	ID=S2009_raw:GT=hom:DP=27:FQ=1:SQ=-1;ID=S2503_raw:GT=hom:DP=90:FQ=1:SQ=-1;ID=S2507_raw:GT=hom:DP=77:FQ=1:SQ=-1;ID=S2668_raw:GT=hom:DP=124:FQ=1:SQ=-1;ID=S2012_raw:GT=hom:DP=36:FQ=1:SQ=-1;ID=S2010_raw:GT=hom:DP=54:FQ=1:SQ=-1;ID=S2662_raw:GT=hom:DP=95:FQ=1:SQ=-1;ID=S2663_raw:GT=hom:DP=75:FQ=1:SQ=-1;ID=S2011_raw:GT=hom:DP=62:FQ=1:SQ=-1;ID=S2504_raw:GT=hom:DP=100:FQ=1:SQ=-1;ID=S2008_raw:GT=hom:DP=45:FQ=1:SQ=-1;ID=S2509_raw:GT=hom:DP=89:FQ=1:SQ=-1;ID=S2508_raw:GT=hom:DP=84:FQ=1:SQ=-1;ID=S2666_raw:GT=hom:DP=101:FQ=1:SQ=-1;ID=S2669_raw:GT=hom:DP=96:FQ=1:SQ=-1;ID=S2166_raw:GT=hom:DP=72:FQ=1:SQ=-1;ID=S1775_raw:GT=hom:DP=41:FQ=1:SQ=-1;ID=S2501_raw:GT=hom:DP=112:FQ=1:SQ=-1;ID=S2500_raw:GT=hom:DP=99:FQ=1:SQ=-1;ID=S2502_raw:GT=hom:DP=92:FQ=1:SQ=-1;ID=S2505_raw:GT=hom:DP=79:FQ=1:SQ=-1;ID=S2176_raw:GT=hom:DP=57:FQ=1:SQ=-1;ID=S2506_raw:GT=hom:DP=115:FQ=1:SQ=-1;ID=S2667_raw:GT=hom:DP=71:FQ=1:SQ=-1;
10	1,04E+08	1,04E+08	T	G	UTR3	SUFU	.	NM_016169:c.*20T>G	rs4917980	.	ID=S2009_raw:GT=hom:DP=118:FQ=1:SQ=-1;ID=S2503_raw:GT=hom:DP=110:FQ=1:SQ=-1;ID=S2507_raw:GT=hom:DP=90:FQ=1:SQ=-1;ID=S2668_raw:GT=hom:DP=108:FQ=1:SQ=-1;ID=S2012_raw:GT=hom:DP=79:FQ=1:SQ=-1;ID=S2010_raw:GT=hom:DP=109:FQ=1:SQ=-1;ID=S2662_raw:GT=hom:DP=171:FQ=1:SQ=-1;ID=S2663_raw:GT=hom:DP=114:FQ=1:SQ=-1;ID=S2011_raw:GT=hom:DP=158:FQ=1:SQ=-1;ID=S2504_raw:GT=hom:DP=100:FQ=1:SQ=-1;ID=S2008_raw:GT=hom:DP=100:FQ=1:SQ=-1;ID=S2509_raw:GT=hom:DP=94:FQ=1:SQ=-1;ID=S2508_raw:GT=hom:DP=119:FQ=1:SQ=-1;ID=S2666_raw:GT=hom:DP=45:FQ=1:SQ=-1;ID=S2669_raw:GT=hom:DP=119:FQ=1:SQ=-1;ID=S2166_raw:GT=hom:DP=82:FQ=1:SQ=-1;ID=S1775_raw:GT=hom:DP=44:FQ=1:SQ=-1;ID=S2501_raw:GT=hom:DP=102:FQ=1:SQ=-1;ID=S2500_raw:GT=hom:DP=89:FQ=1:SQ=-1;ID=S2502_raw:GT=hom:DP=88:FQ=1:SQ=-1;ID=S2505_raw:GT=hom:DP=80:FQ=1:SQ=-1;ID=S2176_raw:GT=hom:DP=85:FQ=1:SQ=-1;ID=S2506_raw:GT=hom:DP=118:FQ=1:SQ=-1;ID=S2667_raw:GT=hom:DP=139:FQ=1:SQ=-1;
1	1,1E+08	1,1E+08	C	T	intronic	TAF13	.	.	rs673365	.	ID=S2009_raw:GT=hom:DP=1:FQ=1:SQ=-1;ID=S2503_raw:GT=hom:DP=27:FQ=1:SQ=-1;ID=S2507_raw:GT=hom:DP=20:FQ=1:SQ=-1;ID=S2668_raw:GT=hom:DP=52:FQ=1:SQ=-1;ID=S2012_raw:GT=hom:DP=3:FQ=1:SQ=-1;ID=S2010_raw:GT=hom:DP=2:FQ=1:SQ=-1;ID=S2662_raw:GT=hom:DP=13:FQ=1:SQ=-1;ID=S2663_raw:GT=hom:DP=12:FQ=1:SQ=-1;ID=S2011_raw:GT=hom:DP=6:FQ=1:SQ=-1;ID=S2504_raw:GT=hom:DP=29:FQ=1:SQ=-1;ID=S2008_raw:GT=hom:DP=4:FQ=1:SQ=-1;ID=S2509_raw:GT=hom:DP=24:FQ=1:SQ=-1;ID=S2508_raw:GT=hom:DP=25:FQ=1:SQ=-1;ID=S2666_raw:GT=hom:DP=37:FQ=1:SQ=-1;ID=S2669_raw:GT=hom:DP=25:FQ=1:SQ=-1;ID=S2166_raw:GT=hom:DP=8:FQ=1:SQ=-1;ID=S1775_raw:GT=hom:DP=5:FQ=1:SQ=-1;ID=S2501_raw:GT=hom:DP=18:FQ=1:SQ=-1;ID=S2500_raw:GT=hom:DP=37:FQ=1:SQ=-1;ID=S2502_raw:GT=hom:DP=41:FQ=1:SQ=-1;ID=S2505_raw:GT=hom:DP=22:FQ=1:SQ=-1;ID=S2176_raw:GT=hom:DP=4:FQ=1:SQ=-1;ID=S2506_raw:GT=hom:DP=28:FQ=0.96:SQ=-1;ID=S2667_raw:GT=hom:DP=32:FQ=1:SQ=-1;
3	1,77E+08	1,77E+08	G	A	intronic	TBL1XR1	.	.	rs1201300	.	ID=S2009_raw:GT=hom:DP=36:FQ=1:SQ=-1;ID=S2503_raw:GT=hom:DP=53:FQ=1:SQ=-1;ID=S2507_raw:GT=hom:DP=48:FQ=1:SQ=-1;ID=S2668_raw:GT=hom:DP=74:FQ=1:SQ=-1;ID=S2012_raw:GT=hom:DP=54:FQ=1:SQ=-1;ID=S2010_raw:GT=hom:DP=61:FQ=1:SQ=-1;ID=S2662_raw:GT=hom:DP=2:FQ=1:SQ=-1;ID=S2663_raw:GT=hom:DP=52:FQ=1:SQ=-1;ID=S2011_raw:GT=hom:DP=82:FQ=1:SQ=-1;ID=S2504_raw:GT=hom:DP=48:FQ=1:SQ=-1;ID=S2008_raw:GT=hom:DP=47:FQ=1:SQ=-1;ID=S2509_raw:GT=hom:DP=46:FQ=1:SQ=-1;ID=S2508_raw:GT=hom:DP=47:FQ=1:SQ=-1;ID=S2666_raw:GT=hom:DP=51:FQ=1:SQ=-1;ID=S2669_raw:GT=hom:DP=7:FQ=1:SQ=-1;ID=S2166_raw:GT=hom:DP=38:FQ=1:SQ=-1;ID=S1775_raw:GT=hom:DP=15:FQ=1:SQ=-1;ID=S2501_raw:GT=hom:DP=42:FQ=1:SQ=-1;ID=S2500_raw:GT=hom:DP=62:FQ=1:SQ=-1;ID=S2502_raw:GT=hom:DP=27:FQ=1:SQ=-1;ID=S2505_raw:GT=hom:DP=44:FQ=1:SQ=-1;ID=S2176_raw:GT=hom:DP=47:FQ=1:SQ=-1;ID=S2506_raw:GT=hom:DP=50:FQ=1:SQ=-1;ID=S2667_raw:GT=hom:DP=74:FQ=1:SQ=-1;
18	53302845	53302845	C	T	intronic	TCF4	.	.	rs610362	.	ID=S2009_raw:GT=hom:DP=35:FQ=1:SQ=-1;ID=S2503_raw:GT=hom:DP=31:FQ=1:SQ=-1;ID=S2507_raw:GT=hom:DP=30:FQ=1:SQ=-1;ID=S2668_raw:GT=hom:DP=63:FQ=1:SQ=-1;ID=S2012_raw:GT=hom:DP=44:FQ=1:SQ=-1;ID=S2010_raw:GT=hom:DP=37:FQ=1:SQ=-1;ID=S2662_raw:GT=hom:DP=11:FQ=1:SQ=-1;ID=S2663_raw:GT=hom:DP=35:FQ=1:SQ=-1;ID=S2011_raw:GT=hom:DP=59:FQ=1:SQ=-1;ID=S2504_raw:GT=hom:DP=48:FQ=1:SQ=-1;ID=S2008_raw:GT=hom:DP=40:FQ=1:SQ=-1;ID=S2509_raw:GT=hom:DP=18:FQ=1:SQ=-1;ID=S2508_raw:GT=hom:DP=35:FQ=1:SQ=-1;ID=S2666_raw:GT=hom:DP=52:FQ=1:SQ=-1;ID=S2669_raw:GT=hom:DP=36:FQ=1:SQ=-1;ID=S2166_raw:GT=hom:DP=50:FQ=1:SQ=-1;ID=S1775_raw:GT=hom:DP=39:FQ=1:SQ=-1;ID=S2501_raw:GT=hom:DP=32:FQ=1:SQ=-1;ID=S2500_raw:GT=hom:DP=34:FQ=1:SQ=-1;ID=S2502_raw:GT=hom:DP=39:FQ=1:SQ=-1;ID=S2505_raw:GT=hom:DP=43:FQ=1:SQ=-1;ID=S2176_raw:GT=hom:DP=50:FQ=1:SQ=-1;ID=S2506_raw:GT=hom:DP=38:FQ=1:SQ=-1;ID=S2667_raw:GT=hom:DP=30:FQ=1:SQ=-1;
18	53303101	53303101	C	G	exonic	TCF4	nonsynonymous SNV	TCF4:NM_001243226:exon1:c.28G>C:p.A10P	rs611326	.	ID=S2009_raw:GT=hom:DP=43:FQ=1:SQ=-1;ID=S2503_raw:GT=hom:DP=48:FQ=1:SQ=-1;ID=S2507_raw:GT=hom:DP=46:FQ=1:SQ=-1;ID=S2668_raw:GT=hom:DP=64:FQ=1:SQ=-1;ID=S2012_raw:GT=hom:DP=65:FQ=1:SQ=-1;ID=S2010_raw:GT=hom:DP=61:FQ=1:SQ=-1;ID=S2662_raw:GT=hom:DP=25:FQ=1:SQ=-1;ID=S2663_raw:GT=hom:DP=59:FQ=1:SQ=-1;ID=S2011_raw:GT=hom:DP=80:FQ=1:SQ=-1;ID=S2504_raw:GT=hom:DP=70:FQ=1:SQ=-1;ID=S2008_raw:GT=hom:DP=57:FQ=1:SQ=-1;ID=S2509_raw:GT=hom:DP=40:FQ=1:SQ=-1;ID=S2508_raw:GT=hom:DP=44:FQ=1:SQ=-1;ID=S2666_raw:GT=hom:DP=38:FQ=1:SQ=-1;ID=S2669_raw:GT=hom:DP=57:FQ=1:SQ=-1;ID=S2166_raw:GT=hom:DP=94:FQ=1:SQ=-1;ID=S1775_raw:GT=hom:DP=64:FQ=1:SQ=-1;ID=S2501_raw:GT=hom:DP=50:FQ=1:SQ=-1;ID=S2500_raw:GT=hom:DP=57:FQ=1:SQ=-1;ID=S2502_raw:GT=hom:DP=51:FQ=1:SQ=-1;ID=S2505_raw:GT=hom:DP=35:FQ=1:SQ=-1;ID=S2176_raw:GT=hom:DP=88:FQ=1:SQ=-1;ID=S2506_raw:GT=hom:DP=36:FQ=1:SQ=-1;ID=S2667_raw:GT=hom:DP=44:FQ=1:SQ=-1;
3	46621123	46621123	A	T	intronic	TDGF1	.	.	rs9819070	.	ID=S2009_raw:GT=hom:DP=5:FQ=1:SQ=-1;ID=S2503_raw:GT=hom:DP=10:FQ=1:SQ=-1;ID=S2507_raw:GT=hom:DP=9:FQ=1:SQ=-1;ID=S2668_raw:GT=hom:DP=7:FQ=1:SQ=-1;ID=S2012_raw:GT=hom:DP=2:FQ=1:SQ=-1;ID=S2010_raw:GT=hom:DP=3:FQ=1:SQ=-1;ID=S2662_raw:GT=hom:DP=3:FQ=1:SQ=-1;ID=S2663_raw:GT=hom:DP=5:FQ=1:SQ=-1;ID=S2011_raw:GT=hom:DP=3:FQ=1:SQ=-1;ID=S2504_raw:GT=hom:DP=8:FQ=1:SQ=-1;ID=S2008_raw:GT=hom:DP=3:FQ=1:SQ=-1;ID=S2509_raw:GT=hom:DP=11:FQ=1:SQ=-1;ID=S2508_raw:GT=hom:DP=10:FQ=1:SQ=-1;ID=S2666_raw:GT=hom:DP=15:FQ=1:SQ=-1;ID=S2669_raw:GT=hom:DP=4:FQ=1:SQ=-1;ID=S2166_raw:GT=hom:DP=21:FQ=1:SQ=-1;ID=S1775_raw:GT=hom:DP=11:FQ=1:SQ=-1;ID=S2501_raw:GT=hom:DP=9:FQ=1:SQ=-1;ID=S2500_raw:GT=hom:DP=11:FQ=1:SQ=-1;ID=S2502_raw:GT=hom:DP=9:FQ=1:SQ=-1;ID=S2505_raw:GT=hom:DP=8:FQ=1:SQ=-1;ID=S2176_raw:GT=hom:DP=14:FQ=1:SQ=-1;ID=S2506_raw:GT=hom:DP=9:FQ=1:SQ=-1;ID=S2667_raw:GT=hom:DP=8:FQ=1:SQ=-1;
X	48898183	48898183	C	G	intronic	TFE3	.	.	rs7056903	.	ID=S2009_raw:GT=hom:DP=12:FQ=1:SQ=-1;ID=S2503_raw:GT=hom:DP=16:FQ=1:SQ=-1;ID=S2507_raw:GT=hom:DP=13:FQ=1:SQ=-1;ID=S2668_raw:GT=hom:DP=20:FQ=1:SQ=-1;ID=S2012_raw:GT=hom:DP=14:FQ=1:SQ=-1;ID=S2010_raw:GT=hom:DP=13:FQ=1:SQ=-1;ID=S2662_raw:GT=hom:DP=26:FQ=1:SQ=-1;ID=S2663_raw:GT=hom:DP=15:FQ=1:SQ=-1;ID=S2011_raw:GT=hom:DP=7:FQ=1:SQ=-1;ID=S2504_raw:GT=hom:DP=16:FQ=1:SQ=-1;ID=S2008_raw:GT=hom:DP=27:FQ=1:SQ=-1;ID=S2509_raw:GT=hom:DP=8:FQ=1:SQ=-1;ID=S2508_raw:GT=hom:DP=12:FQ=1:SQ=-1;ID=S2666_raw:GT=hom:DP=19:FQ=1:SQ=-1;ID=S2669_raw:GT=hom:DP=6:FQ=1:SQ=-1;ID=S2166_raw:GT=hom:DP=18:FQ=1:SQ=-1;ID=S1775_raw:GT=hom:DP=14:FQ=1:SQ=-1;ID=S2501_raw:GT=hom:DP=15:FQ=1:SQ=-1;ID=S2500_raw:GT=hom:DP=14:FQ=1:SQ=-1;ID=S2502_raw:GT=hom:DP=31:FQ=1:SQ=-1;ID=S2505_raw:GT=hom:DP=13:FQ=1:SQ=-1;ID=S2176_raw:GT=hom:DP=14:FQ=1:SQ=-1;ID=S2506_raw:GT=hom:DP=8:FQ=1:SQ=-1;ID=S2667_raw:GT=hom:DP=25:FQ=1:SQ=-1;
14	24718402	24718423	CTCCGGGAGCCCTGGACTCCCC	-	UTR3	TGM1	.	NM_000359:c.*117_*96delGGGGAGTCCAGGGCTCCCGGAG	.	.	ID=S2009_raw:GT=hom:DP=7:FQ=1:SQ=-1;ID=S2503_raw:GT=hom:DP=21:FQ=1:SQ=-1;ID=S2507_raw:GT=hom:DP=11:FQ=1:SQ=-1;ID=S2668_raw:GT=hom:DP=16:FQ=1:SQ=-1;ID=S2012_raw:GT=hom:DP=7:FQ=1:SQ=-1;ID=S2010_raw:GT=hom:DP=22:FQ=1:SQ=-1;ID=S2662_raw:GT=hom:DP=11:FQ=1:SQ=-1;ID=S2663_raw:GT=hom:DP=10:FQ=1:SQ=-1;ID=S2011_raw:GT=hom:DP=10:FQ=1:SQ=-1;ID=S2504_raw:GT=hom:DP=11:FQ=1:SQ=-1;ID=S2008_raw:GT=hom:DP=10:FQ=1:SQ=-1;ID=S2509_raw:GT=hom:DP=16:FQ=1:SQ=-1;ID=S2508_raw:GT=hom:DP=18:FQ=1:SQ=-1;ID=S2666_raw:GT=hom:DP=7:FQ=1:SQ=-1;ID=S2669_raw:GT=hom:DP=8:FQ=1:SQ=-1;ID=S2166_raw:GT=hom:DP=23:FQ=1:SQ=-1;ID=S1775_raw:GT=hom:DP=22:FQ=1:SQ=-1;ID=S2501_raw:GT=hom:DP=16:FQ=1:SQ=-1;ID=S2500_raw:GT=hom:DP=14:FQ=1:SQ=-1;ID=S2502_raw:GT=hom:DP=17:FQ=1:SQ=-1;ID=S2505_raw:GT=hom:DP=18:FQ=1:SQ=-1;ID=S2176_raw:GT=hom:DP=32:FQ=1:SQ=-1;ID=S2506_raw:GT=hom:DP=13:FQ=1:SQ=-1;ID=S2667_raw:GT=hom:DP=11:FQ=1:SQ=-1;
3	53262867	53262867	G	A	intronic	TKT	.	.	rs13075304	.	ID=S2009_raw:GT=hom:DP=7:FQ=1:SQ=-1;ID=S2503_raw:GT=hom:DP=24:FQ=1:SQ=-1;ID=S2507_raw:GT=hom:DP=15:FQ=1:SQ=-1;ID=S2668_raw:GT=hom:DP=8:FQ=1:SQ=-1;ID=S2012_raw:GT=hom:DP=5:FQ=1:SQ=-1;ID=S2010_raw:GT=hom:DP=6:FQ=1:SQ=-1;ID=S2662_raw:GT=hom:DP=11:FQ=1:SQ=-1;ID=S2663_raw:GT=hom:DP=8:FQ=1:SQ=-1;ID=S2011_raw:GT=hom:DP=11:FQ=1:SQ=-1;ID=S2504_raw:GT=hom:DP=9:FQ=1:SQ=-1;ID=S2008_raw:GT=hom:DP=11:FQ=1:SQ=-1;ID=S2509_raw:GT=hom:DP=15:FQ=1:SQ=-1;ID=S2508_raw:GT=hom:DP=14:FQ=1:SQ=-1;ID=S2666_raw:GT=hom:DP=11:FQ=1:SQ=-1;ID=S2669_raw:GT=hom:DP=9:FQ=1:SQ=-1;ID=S2166_raw:GT=hom:DP=11:FQ=1:SQ=-1;ID=S1775_raw:GT=hom:DP=6:FQ=1:SQ=-1;ID=S2501_raw:GT=hom:DP=11:FQ=1:SQ=-1;ID=S2500_raw:GT=hom:DP=13:FQ=1:SQ=-1;ID=S2502_raw:GT=hom:DP=9:FQ=1:SQ=-1;ID=S2505_raw:GT=hom:DP=10:FQ=1:SQ=-1;ID=S2176_raw:GT=hom:DP=16:FQ=1:SQ=-1;ID=S2506_raw:GT=hom:DP=18:FQ=1:SQ=-1;ID=S2667_raw:GT=hom:DP=13:FQ=1:SQ=-1;
3	53267133	53267133	G	C	intronic	TKT	.	.	rs6445573	.	ID=S2009_raw:GT=hom:DP=28:FQ=1:SQ=-1;ID=S2503_raw:GT=hom:DP=74:FQ=1:SQ=-1;ID=S2507_raw:GT=hom:DP=77:FQ=1:SQ=-1;ID=S2668_raw:GT=hom:DP=95:FQ=1:SQ=-1;ID=S2012_raw:GT=hom:DP=23:FQ=1:SQ=-1;ID=S2010_raw:GT=hom:DP=36:FQ=1:SQ=-1;ID=S2662_raw:GT=hom:DP=122:FQ=1:SQ=-1;ID=S2663_raw:GT=hom:DP=89:FQ=1:SQ=-1;ID=S2011_raw:GT=hom:DP=35:FQ=1:SQ=-1;ID=S2504_raw:GT=hom:DP=91:FQ=1:SQ=-1;ID=S2008_raw:GT=hom:DP=34:FQ=1:SQ=-1;ID=S2509_raw:GT=hom:DP=72:FQ=1:SQ=-1;ID=S2508_raw:GT=hom:DP=78:FQ=1:SQ=-1;ID=S2666_raw:GT=hom:DP=73:FQ=1:SQ=-1;ID=S2669_raw:GT=hom:DP=83:FQ=1:SQ=-1;ID=S2166_raw:GT=hom:DP=87:FQ=1:SQ=-1;ID=S1775_raw:GT=hom:DP=30:FQ=1:SQ=-1;ID=S2501_raw:GT=hom:DP=73:FQ=1:SQ=-1;ID=S2500_raw:GT=hom:DP=60:FQ=1:SQ=-1;ID=S2502_raw:GT=hom:DP=102:FQ=0.99:SQ=-1;ID=S2505_raw:GT=hom:DP=87:FQ=1:SQ=-1;ID=S2176_raw:GT=hom:DP=73:FQ=1:SQ=-1;ID=S2506_raw:GT=hom:DP=71:FQ=1:SQ=-1;ID=S2667_raw:GT=hom:DP=68:FQ=1:SQ=-1;
21	45514146	45514146	G	-	intronic	TRAPPC10	.	.	.	.	ID=S2009_raw:GT=hom:DP=44:FQ=1:SQ=-1;ID=S2503_raw:GT=hom:DP=46:FQ=1:SQ=-1;ID=S2507_raw:GT=hom:DP=30:FQ=1:SQ=-1;ID=S2668_raw:GT=hom:DP=28:FQ=1:SQ=-1;ID=S2012_raw:GT=hom:DP=35:FQ=1:SQ=-1;ID=S2010_raw:GT=hom:DP=32:FQ=1:SQ=-1;ID=S2662_raw:GT=hom:DP=16:FQ=1:SQ=-1;ID=S2663_raw:GT=hom:DP=35:FQ=1:SQ=-1;ID=S2011_raw:GT=hom:DP=56:FQ=1:SQ=-1;ID=S2504_raw:GT=hom:DP=51:FQ=1:SQ=-1;ID=S2008_raw:GT=hom:DP=44:FQ=1:SQ=-1;ID=S2509_raw:GT=hom:DP=41:FQ=1:SQ=-1;ID=S2508_raw:GT=hom:DP=44:FQ=1:SQ=-1;ID=S2666_raw:GT=hom:DP=15:FQ=1:SQ=-1;ID=S2669_raw:GT=hom:DP=22:FQ=1:SQ=-1;ID=S2166_raw:GT=hom:DP=25:FQ=1:SQ=-1;ID=S1775_raw:GT=hom:DP=18:FQ=1:SQ=-1;ID=S2501_raw:GT=hom:DP=34:FQ=1:SQ=-1;ID=S2500_raw:GT=hom:DP=37:FQ=1:SQ=-1;ID=S2502_raw:GT=hom:DP=50:FQ=1:SQ=-1;ID=S2505_raw:GT=hom:DP=58:FQ=1:SQ=-1;ID=S2176_raw:GT=hom:DP=26:FQ=1:SQ=-1;ID=S2506_raw:GT=hom:DP=46:FQ=1:SQ=-1;ID=S2667_raw:GT=hom:DP=36:FQ=1:SQ=-1;
21	45514149	45514149	A	G	intronic	TRAPPC10	.	.	rs9941801	.	ID=S2009_raw:GT=hom:DP=46:FQ=0.98:SQ=-1;ID=S2503_raw:GT=hom:DP=40:FQ=1:SQ=-1;ID=S2507_raw:GT=hom:DP=24:FQ=1:SQ=-1;ID=S2668_raw:GT=hom:DP=26:FQ=1:SQ=-1;ID=S2012_raw:GT=hom:DP=36:FQ=1:SQ=-1;ID=S2010_raw:GT=hom:DP=30:FQ=0.97:SQ=-1;ID=S2662_raw:GT=hom:DP=14:FQ=1:SQ=-1;ID=S2663_raw:GT=hom:DP=35:FQ=1:SQ=-1;ID=S2011_raw:GT=hom:DP=52:FQ=1:SQ=-1;ID=S2504_raw:GT=hom:DP=44:FQ=0.98:SQ=-1;ID=S2008_raw:GT=hom:DP=43:FQ=1:SQ=-1;ID=S2509_raw:GT=hom:DP=38:FQ=1:SQ=-1;ID=S2508_raw:GT=hom:DP=43:FQ=1:SQ=-1;ID=S2666_raw:GT=hom:DP=15:FQ=1:SQ=-1;ID=S2669_raw:GT=hom:DP=17:FQ=1:SQ=-1;ID=S2166_raw:GT=hom:DP=24:FQ=1:SQ=-1;ID=S1775_raw:GT=hom:DP=20:FQ=1:SQ=-1;ID=S2501_raw:GT=hom:DP=30:FQ=0.97:SQ=-1;ID=S2500_raw:GT=hom:DP=29:FQ=1:SQ=-1;ID=S2502_raw:GT=hom:DP=47:FQ=1:SQ=-1;ID=S2505_raw:GT=hom:DP=54:FQ=1:SQ=-1;ID=S2176_raw:GT=hom:DP=25:FQ=1:SQ=-1;ID=S2506_raw:GT=hom:DP=40:FQ=1:SQ=-1;ID=S2667_raw:GT=hom:DP=29:FQ=1:SQ=-1;
21	45514168	45514168	C	T	intronic	TRAPPC10	.	.	rs4819371	.	ID=S2009_raw:GT=hom:DP=33:FQ=1:SQ=-1;ID=S2503_raw:GT=hom:DP=33:FQ=1:SQ=-1;ID=S2507_raw:GT=hom:DP=16:FQ=1:SQ=-1;ID=S2668_raw:GT=hom:DP=21:FQ=1:SQ=-1;ID=S2012_raw:GT=hom:DP=27:FQ=1:SQ=-1;ID=S2010_raw:GT=hom:DP=34:FQ=1:SQ=-1;ID=S2662_raw:GT=hom:DP=12:FQ=1:SQ=-1;ID=S2663_raw:GT=hom:DP=23:FQ=1:SQ=-1;ID=S2011_raw:GT=hom:DP=39:FQ=1:SQ=-1;ID=S2504_raw:GT=hom:DP=33:FQ=1:SQ=-1;ID=S2008_raw:GT=hom:DP=35:FQ=1:SQ=-1;ID=S2509_raw:GT=hom:DP=26:FQ=1:SQ=-1;ID=S2508_raw:GT=hom:DP=38:FQ=1:SQ=-1;ID=S2666_raw:GT=hom:DP=11:FQ=1:SQ=-1;ID=S2669_raw:GT=hom:DP=13:FQ=1:SQ=-1;ID=S2166_raw:GT=hom:DP=17:FQ=1:SQ=-1;ID=S1775_raw:GT=hom:DP=17:FQ=1:SQ=-1;ID=S2501_raw:GT=hom:DP=26:FQ=1:SQ=-1;ID=S2500_raw:GT=hom:DP=23:FQ=1:SQ=-1;ID=S2502_raw:GT=hom:DP=37:FQ=1:SQ=-1;ID=S2505_raw:GT=hom:DP=40:FQ=1:SQ=-1;ID=S2176_raw:GT=hom:DP=22:FQ=1:SQ=-1;ID=S2506_raw:GT=hom:DP=32:FQ=1:SQ=-1;ID=S2667_raw:GT=hom:DP=25:FQ=1:SQ=-1;
21	45514188	45514188	A	G	intronic	TRAPPC10	.	.	rs4818879	.	ID=S2009_raw:GT=hom:DP=19:FQ=1:SQ=-1;ID=S2503_raw:GT=hom:DP=19:FQ=1:SQ=-1;ID=S2507_raw:GT=hom:DP=9:FQ=1:SQ=-1;ID=S2668_raw:GT=hom:DP=12:FQ=1:SQ=-1;ID=S2012_raw:GT=hom:DP=25:FQ=1:SQ=-1;ID=S2010_raw:GT=hom:DP=22:FQ=1:SQ=-1;ID=S2662_raw:GT=hom:DP=4:FQ=1:SQ=-1;ID=S2663_raw:GT=hom:DP=18:FQ=1:SQ=-1;ID=S2011_raw:GT=hom:DP=27:FQ=1:SQ=-1;ID=S2504_raw:GT=hom:DP=24:FQ=1:SQ=-1;ID=S2008_raw:GT=hom:DP=25:FQ=1:SQ=-1;ID=S2509_raw:GT=hom:DP=17:FQ=1:SQ=-1;ID=S2508_raw:GT=hom:DP=21:FQ=1:SQ=-1;ID=S2666_raw:GT=hom:DP=4:FQ=1:SQ=-1;ID=S2669_raw:GT=hom:DP=8:FQ=1:SQ=-1;ID=S2166_raw:GT=hom:DP=12:FQ=1:SQ=-1;ID=S1775_raw:GT=hom:DP=13:FQ=1:SQ=-1;ID=S2501_raw:GT=hom:DP=17:FQ=1:SQ=-1;ID=S2500_raw:GT=hom:DP=13:FQ=1:SQ=-1;ID=S2502_raw:GT=hom:DP=26:FQ=1:SQ=-1;ID=S2505_raw:GT=hom:DP=21:FQ=1:SQ=-1;ID=S2176_raw:GT=hom:DP=14:FQ=1:SQ=-1;ID=S2506_raw:GT=hom:DP=20:FQ=1:SQ=-1;ID=S2667_raw:GT=hom:DP=17:FQ=1:SQ=-1;
10	1,04E+08	1,04E+08	T	C	intronic	TRIM8	.	.	rs4274160	.	ID=S2009_raw:GT=hom:DP=41:FQ=1:SQ=-1;ID=S2503_raw:GT=hom:DP=46:FQ=1:SQ=-1;ID=S2507_raw:GT=hom:DP=28:FQ=1:SQ=-1;ID=S2668_raw:GT=hom:DP=31:FQ=1:SQ=-1;ID=S2012_raw:GT=hom:DP=68:FQ=1:SQ=-1;ID=S2010_raw:GT=hom:DP=57:FQ=1:SQ=-1;ID=S2662_raw:GT=hom:DP=41:FQ=1:SQ=-1;ID=S2663_raw:GT=hom:DP=28:FQ=1:SQ=-1;ID=S2011_raw:GT=hom:DP=72:FQ=1:SQ=-1;ID=S2504_raw:GT=hom:DP=36:FQ=1:SQ=-1;ID=S2008_raw:GT=hom:DP=63:FQ=1:SQ=-1;ID=S2509_raw:GT=hom:DP=33:FQ=1:SQ=-1;ID=S2508_raw:GT=hom:DP=28:FQ=1:SQ=-1;ID=S2666_raw:GT=hom:DP=14:FQ=1:SQ=-1;ID=S2669_raw:GT=hom:DP=26:FQ=1:SQ=-1;ID=S2166_raw:GT=hom:DP=49:FQ=1:SQ=-1;ID=S1775_raw:GT=hom:DP=28:FQ=1:SQ=-1;ID=S2501_raw:GT=hom:DP=41:FQ=1:SQ=-1;ID=S2500_raw:GT=hom:DP=39:FQ=1:SQ=-1;ID=S2502_raw:GT=hom:DP=32:FQ=1:SQ=-1;ID=S2505_raw:GT=hom:DP=33:FQ=1:SQ=-1;ID=S2176_raw:GT=hom:DP=42:FQ=1:SQ=-1;ID=S2506_raw:GT=hom:DP=33:FQ=1:SQ=-1;ID=S2667_raw:GT=hom:DP=30:FQ=1:SQ=-1;
14	61445967	61445967	A	G	exonic	TRMT5	nonsynonymous SNV	TRMT5:NM_001350253:exon2:c.733T>C:p.S245P,TRMT5:NM_001350254:exon2:c.730T>C:p.S244P,TRMT5:NM_020810:exon2:c.649T>C:p.S217P	rs7142228	.	ID=S2009_raw:GT=hom:DP=25:FQ=1:SQ=-1;ID=S2503_raw:GT=hom:DP=94:FQ=1:SQ=-1;ID=S2507_raw:GT=hom:DP=94:FQ=1:SQ=-1;ID=S2668_raw:GT=hom:DP=152:FQ=1:SQ=-1;ID=S2012_raw:GT=hom:DP=46:FQ=1:SQ=-1;ID=S2010_raw:GT=hom:DP=49:FQ=1:SQ=-1;ID=S2662_raw:GT=hom:DP=2:FQ=1:SQ=-1;ID=S2663_raw:GT=hom:DP=96:FQ=1:SQ=-1;ID=S2011_raw:GT=hom:DP=62:FQ=1:SQ=-1;ID=S2504_raw:GT=hom:DP=88:FQ=1:SQ=-1;ID=S2008_raw:GT=hom:DP=56:FQ=1:SQ=-1;ID=S2509_raw:GT=hom:DP=93:FQ=1:SQ=-1;ID=S2508_raw:GT=hom:DP=99:FQ=1:SQ=-1;ID=S2666_raw:GT=hom:DP=96:FQ=1:SQ=-1;ID=S2669_raw:GT=hom:DP=68:FQ=1:SQ=-1;ID=S2166_raw:GT=hom:DP=155:FQ=1:SQ=-1;ID=S1775_raw:GT=hom:DP=110:FQ=1:SQ=-1;ID=S2501_raw:GT=hom:DP=101:FQ=1:SQ=-1;ID=S2500_raw:GT=hom:DP=84:FQ=1:SQ=-1;ID=S2502_raw:GT=hom:DP=93:FQ=1:SQ=-1;ID=S2505_raw:GT=hom:DP=110:FQ=1:SQ=-1;ID=S2176_raw:GT=hom:DP=149:FQ=1:SQ=-1;ID=S2506_raw:GT=hom:DP=70:FQ=1:SQ=-1;ID=S2667_raw:GT=hom:DP=88:FQ=1:SQ=-1;
3	3170792	3170792	C	T	exonic	TRNT1	nonsynonymous SNV	TRNT1:NM_001302946:exon2:c.68C>T:p.P23L,TRNT1:NM_001367321:exon2:c.68C>T:p.P23L,TRNT1:NM_001367322:exon2:c.68C>T:p.P23L,TRNT1:NM_001367323:exon2:c.68C>T:p.P23L,TRNT1:NM_182916:exon2:c.68C>T:p.P23L	rs334773	Benign	ID=S2009_raw:GT=hom:DP=40:FQ=1:SQ=-1;ID=S2503_raw:GT=hom:DP=80:FQ=1:SQ=-1;ID=S2507_raw:GT=hom:DP=86:FQ=1:SQ=-1;ID=S2668_raw:GT=hom:DP=103:FQ=1:SQ=-1;ID=S2012_raw:GT=hom:DP=48:FQ=1:SQ=-1;ID=S2010_raw:GT=hom:DP=43:FQ=1:SQ=-1;ID=S2662_raw:GT=hom:DP=31:FQ=1:SQ=-1;ID=S2663_raw:GT=hom:DP=87:FQ=1:SQ=-1;ID=S2011_raw:GT=hom:DP=64:FQ=1:SQ=-1;ID=S2504_raw:GT=hom:DP=97:FQ=1:SQ=-1;ID=S2008_raw:GT=hom:DP=68:FQ=1:SQ=-1;ID=S2509_raw:GT=hom:DP=84:FQ=1:SQ=-1;ID=S2508_raw:GT=hom:DP=107:FQ=1:SQ=-1;ID=S2666_raw:GT=hom:DP=91:FQ=1:SQ=-1;ID=S2669_raw:GT=hom:DP=87:FQ=1:SQ=-1;ID=S2166_raw:GT=hom:DP=96:FQ=1:SQ=-1;ID=S1775_raw:GT=hom:DP=43:FQ=1:SQ=-1;ID=S2501_raw:GT=hom:DP=88:FQ=1:SQ=-1;ID=S2500_raw:GT=hom:DP=70:FQ=1:SQ=-1;ID=S2502_raw:GT=hom:DP=108:FQ=1:SQ=-1;ID=S2505_raw:GT=hom:DP=75:FQ=1:SQ=-1;ID=S2176_raw:GT=hom:DP=97:FQ=1:SQ=-1;ID=S2506_raw:GT=hom:DP=92:FQ=1:SQ=-1;ID=S2667_raw:GT=hom:DP=84:FQ=1:SQ=-1;
3	3170910	3170910	A	G	intronic	TRNT1	.	.	rs334772	.	ID=S2009_raw:GT=hom:DP=59:FQ=1:SQ=-1;ID=S2503_raw:GT=hom:DP=28:FQ=1:SQ=-1;ID=S2507_raw:GT=hom:DP=32:FQ=1:SQ=-1;ID=S2668_raw:GT=hom:DP=35:FQ=1:SQ=-1;ID=S2012_raw:GT=hom:DP=52:FQ=1:SQ=-1;ID=S2010_raw:GT=hom:DP=76:FQ=1:SQ=-1;ID=S2662_raw:GT=hom:DP=10:FQ=1:SQ=-1;ID=S2663_raw:GT=hom:DP=41:FQ=1:SQ=-1;ID=S2011_raw:GT=hom:DP=100:FQ=1:SQ=-1;ID=S2504_raw:GT=hom:DP=29:FQ=1:SQ=-1;ID=S2008_raw:GT=hom:DP=87:FQ=1:SQ=-1;ID=S2509_raw:GT=hom:DP=33:FQ=1:SQ=-1;ID=S2508_raw:GT=hom:DP=34:FQ=0.97:SQ=-1;ID=S2666_raw:GT=hom:DP=33:FQ=0.94:SQ=-1;ID=S2669_raw:GT=hom:DP=27:FQ=1:SQ=-1;ID=S2166_raw:GT=hom:DP=26:FQ=1:SQ=-1;ID=S1775_raw:GT=hom:DP=16:FQ=1:SQ=-1;ID=S2501_raw:GT=hom:DP=21:FQ=1:SQ=-1;ID=S2500_raw:GT=hom:DP=22:FQ=1:SQ=-1;ID=S2502_raw:GT=hom:DP=24:FQ=1:SQ=-1;ID=S2505_raw:GT=hom:DP=21:FQ=1:SQ=-1;ID=S2176_raw:GT=hom:DP=25:FQ=1:SQ=-1;ID=S2506_raw:GT=hom:DP=40:FQ=1:SQ=-1;ID=S2667_raw:GT=hom:DP=31:FQ=1:SQ=-1;
3	3171460	3171460	C	T	intronic	TRNT1	.	.	rs334769	.	ID=S2009_raw:GT=hom:DP=12:FQ=1:SQ=-1;ID=S2503_raw:GT=hom:DP=33:FQ=0.97:SQ=-1;ID=S2507_raw:GT=hom:DP=37:FQ=1:SQ=-1;ID=S2668_raw:GT=hom:DP=28:FQ=1:SQ=-1;ID=S2012_raw:GT=hom:DP=20:FQ=1:SQ=-1;ID=S2010_raw:GT=hom:DP=15:FQ=1:SQ=-1;ID=S2662_raw:GT=hom:DP=16:FQ=1:SQ=-1;ID=S2663_raw:GT=hom:DP=26:FQ=1:SQ=-1;ID=S2011_raw:GT=hom:DP=25:FQ=1:SQ=-1;ID=S2504_raw:GT=hom:DP=30:FQ=1:SQ=-1;ID=S2008_raw:GT=hom:DP=21:FQ=1:SQ=-1;ID=S2509_raw:GT=hom:DP=21:FQ=1:SQ=-1;ID=S2508_raw:GT=hom:DP=33:FQ=1:SQ=-1;ID=S2666_raw:GT=hom:DP=45:FQ=1:SQ=-1;ID=S2669_raw:GT=hom:DP=33:FQ=1:SQ=-1;ID=S2166_raw:GT=hom:DP=68:FQ=1:SQ=-1;ID=S1775_raw:GT=hom:DP=34:FQ=1:SQ=-1;ID=S2501_raw:GT=hom:DP=19:FQ=1:SQ=-1;ID=S2500_raw:GT=hom:DP=31:FQ=1:SQ=-1;ID=S2502_raw:GT=hom:DP=30:FQ=1:SQ=-1;ID=S2505_raw:GT=hom:DP=26:FQ=1:SQ=-1;ID=S2176_raw:GT=hom:DP=66:FQ=1:SQ=-1;ID=S2506_raw:GT=hom:DP=21:FQ=1:SQ=-1;ID=S2667_raw:GT=hom:DP=23:FQ=1:SQ=-1;
3	3171715	3171715	-	TGACTCC	intronic	TRNT1	.	.	.	.	ID=S2009_raw:GT=hom:DP=25:FQ=1:SQ=-1;ID=S2503_raw:GT=hom:DP=33:FQ=1:SQ=-1;ID=S2507_raw:GT=hom:DP=32:FQ=1:SQ=-1;ID=S2668_raw:GT=hom:DP=44:FQ=1:SQ=-1;ID=S2012_raw:GT=hom:DP=30:FQ=1:SQ=-1;ID=S2010_raw:GT=hom:DP=34:FQ=1:SQ=-1;ID=S2662_raw:GT=hom:DP=6:FQ=1:SQ=-1;ID=S2663_raw:GT=hom:DP=26:FQ=1:SQ=-1;ID=S2011_raw:GT=hom:DP=38:FQ=1:SQ=-1;ID=S2504_raw:GT=hom:DP=30:FQ=1:SQ=-1;ID=S2008_raw:GT=hom:DP=43:FQ=1:SQ=-1;ID=S2509_raw:GT=hom:DP=24:FQ=1:SQ=-1;ID=S2508_raw:GT=hom:DP=38:FQ=1:SQ=-1;ID=S2666_raw:GT=hom:DP=30:FQ=1:SQ=-1;ID=S2669_raw:GT=hom:DP=22:FQ=1:SQ=-1;ID=S2166_raw:GT=hom:DP=50:FQ=1:SQ=-1;ID=S1775_raw:GT=hom:DP=26:FQ=1:SQ=-1;ID=S2501_raw:GT=hom:DP=21:FQ=1:SQ=-1;ID=S2500_raw:GT=hom:DP=34:FQ=1:SQ=-1;ID=S2502_raw:GT=hom:DP=45:FQ=1:SQ=-1;ID=S2505_raw:GT=hom:DP=26:FQ=1:SQ=-1;ID=S2176_raw:GT=hom:DP=65:FQ=1:SQ=-1;ID=S2506_raw:GT=hom:DP=23:FQ=1:SQ=-1;ID=S2667_raw:GT=hom:DP=26:FQ=1:SQ=-1;
3	3182485	3182485	A	G	intronic	TRNT1	.	.	rs184579	.	ID=S2009_raw:GT=hom:DP=1:FQ=1:SQ=-1;ID=S2503_raw:GT=hom:DP=4:FQ=1:SQ=-1;ID=S2507_raw:GT=hom:DP=8:FQ=1:SQ=-1;ID=S2668_raw:GT=hom:DP=8:FQ=1:SQ=-1;ID=S2012_raw:GT=hom:DP=2:FQ=1:SQ=-1;ID=S2010_raw:GT=hom:DP=4:FQ=1:SQ=-1;ID=S2662_raw:GT=hom:DP=6:FQ=1:SQ=-1;ID=S2663_raw:GT=hom:DP=13:FQ=1:SQ=-1;ID=S2011_raw:GT=hom:DP=3:FQ=1:SQ=-1;ID=S2504_raw:GT=hom:DP=11:FQ=1:SQ=-1;ID=S2008_raw:GT=hom:DP=3:FQ=1:SQ=-1;ID=S2509_raw:GT=hom:DP=7:FQ=1:SQ=-1;ID=S2508_raw:GT=hom:DP=5:FQ=1:SQ=-1;ID=S2666_raw:GT=hom:DP=1:FQ=1:SQ=-1;ID=S2669_raw:GT=hom:DP=8:FQ=1:SQ=-1;ID=S2166_raw:GT=hom:DP=9:FQ=1:SQ=-1;ID=S1775_raw:GT=hom:DP=7:FQ=1:SQ=-1;ID=S2501_raw:GT=hom:DP=4:FQ=1:SQ=-1;ID=S2500_raw:GT=hom:DP=7:FQ=1:SQ=-1;ID=S2502_raw:GT=hom:DP=7:FQ=1:SQ=-1;ID=S2505_raw:GT=hom:DP=5:FQ=1:SQ=-1;ID=S2176_raw:GT=hom:DP=11:FQ=1:SQ=-1;ID=S2506_raw:GT=hom:DP=5:FQ=1:SQ=-1;ID=S2667_raw:GT=hom:DP=3:FQ=1:SQ=-1;
3	3182500	3182500	G	C	intronic	TRNT1	.	.	rs334760	.	ID=S2009_raw:GT=hom:DP=1:FQ=1:SQ=-1;ID=S2503_raw:GT=hom:DP=3:FQ=1:SQ=-1;ID=S2507_raw:GT=hom:DP=5:FQ=1:SQ=-1;ID=S2668_raw:GT=hom:DP=8:FQ=1:SQ=-1;ID=S2012_raw:GT=hom:DP=3:FQ=1:SQ=-1;ID=S2010_raw:GT=hom:DP=4:FQ=1:SQ=-1;ID=S2662_raw:GT=hom:DP=6:FQ=1:SQ=-1;ID=S2663_raw:GT=hom:DP=9:FQ=1:SQ=-1;ID=S2011_raw:GT=hom:DP=1:FQ=1:SQ=-1;ID=S2504_raw:GT=hom:DP=10:FQ=1:SQ=-1;ID=S2008_raw:GT=hom:DP=1:FQ=1:SQ=-1;ID=S2509_raw:GT=hom:DP=6:FQ=1:SQ=-1;ID=S2508_raw:GT=hom:DP=5:FQ=1:SQ=-1;ID=S2666_raw:GT=hom:DP=1:FQ=1:SQ=-1;ID=S2669_raw:GT=hom:DP=7:FQ=1:SQ=-1;ID=S2166_raw:GT=hom:DP=7:FQ=1:SQ=-1;ID=S1775_raw:GT=hom:DP=8:FQ=1:SQ=-1;ID=S2501_raw:GT=hom:DP=4:FQ=1:SQ=-1;ID=S2500_raw:GT=hom:DP=6:FQ=1:SQ=-1;ID=S2502_raw:GT=hom:DP=6:FQ=1:SQ=-1;ID=S2505_raw:GT=hom:DP=6:FQ=1:SQ=-1;ID=S2176_raw:GT=hom:DP=9:FQ=1:SQ=-1;ID=S2506_raw:GT=hom:DP=4:FQ=1:SQ=-1;ID=S2667_raw:GT=hom:DP=2:FQ=1:SQ=-1;
14	89576523	89576523	T	A	intergenic	TTC8;FOXN3	.	dist=229101;dist=45993	rs2761400	.	ID=S2009_raw:GT=hom:DP=8:FQ=1:SQ=-1;ID=S2503_raw:GT=hom:DP=34:FQ=1:SQ=-1;ID=S2507_raw:GT=hom:DP=18:FQ=1:SQ=-1;ID=S2668_raw:GT=hom:DP=36:FQ=1:SQ=-1;ID=S2012_raw:GT=hom:DP=15:FQ=1:SQ=-1;ID=S2010_raw:GT=hom:DP=18:FQ=1:SQ=-1;ID=S2662_raw:GT=hom:DP=19:FQ=1:SQ=-1;ID=S2663_raw:GT=hom:DP=23:FQ=1:SQ=-1;ID=S2011_raw:GT=hom:DP=17:FQ=1:SQ=-1;ID=S2504_raw:GT=hom:DP=20:FQ=1:SQ=-1;ID=S2008_raw:GT=hom:DP=19:FQ=1:SQ=-1;ID=S2509_raw:GT=hom:DP=18:FQ=1:SQ=-1;ID=S2508_raw:GT=hom:DP=22:FQ=1:SQ=-1;ID=S2666_raw:GT=hom:DP=19:FQ=1:SQ=-1;ID=S2669_raw:GT=hom:DP=23:FQ=1:SQ=-1;ID=S2166_raw:GT=hom:DP=14:FQ=1:SQ=-1;ID=S1775_raw:GT=hom:DP=11:FQ=1:SQ=-1;ID=S2501_raw:GT=hom:DP=22:FQ=1:SQ=-1;ID=S2500_raw:GT=hom:DP=22:FQ=1:SQ=-1;ID=S2502_raw:GT=hom:DP=30:FQ=1:SQ=-1;ID=S2505_raw:GT=hom:DP=21:FQ=1:SQ=-1;ID=S2176_raw:GT=hom:DP=13:FQ=1:SQ=-1;ID=S2506_raw:GT=hom:DP=22:FQ=1:SQ=-1;ID=S2667_raw:GT=hom:DP=17:FQ=1:SQ=-1;
2	1,67E+08	1,67E+08	T	C	exonic	TTC21B	nonsynonymous SNV	TTC21B:NM_024753:exon8:c.826A>G:p.T276A	rs7592429	Benign	ID=S2009_raw:GT=hom:DP=59:FQ=1:SQ=-1;ID=S2503_raw:GT=hom:DP=106:FQ=1:SQ=-1;ID=S2507_raw:GT=hom:DP=124:FQ=1:SQ=-1;ID=S2668_raw:GT=hom:DP=170:FQ=1:SQ=-1;ID=S2012_raw:GT=hom:DP=80:FQ=1:SQ=-1;ID=S2010_raw:GT=hom:DP=78:FQ=1:SQ=-1;ID=S2662_raw:GT=hom:DP=10:FQ=1:SQ=-1;ID=S2663_raw:GT=hom:DP=127:FQ=1:SQ=-1;ID=S2011_raw:GT=hom:DP=95:FQ=1:SQ=-1;ID=S2504_raw:GT=hom:DP=132:FQ=1:SQ=-1;ID=S2008_raw:GT=hom:DP=82:FQ=1:SQ=-1;ID=S2509_raw:GT=hom:DP=127:FQ=1:SQ=-1;ID=S2508_raw:GT=hom:DP=119:FQ=1:SQ=-1;ID=S2666_raw:GT=hom:DP=133:FQ=1:SQ=-1;ID=S2669_raw:GT=hom:DP=77:FQ=1:SQ=-1;ID=S2166_raw:GT=hom:DP=140:FQ=1:SQ=-1;ID=S1775_raw:GT=hom:DP=104:FQ=1:SQ=-1;ID=S2501_raw:GT=hom:DP=110:FQ=1:SQ=-1;ID=S2500_raw:GT=hom:DP=111:FQ=1:SQ=-1;ID=S2502_raw:GT=hom:DP=135:FQ=1:SQ=-1;ID=S2505_raw:GT=hom:DP=112:FQ=1:SQ=-1;ID=S2176_raw:GT=hom:DP=136:FQ=1:SQ=-1;ID=S2506_raw:GT=hom:DP=107:FQ=1:SQ=-1;ID=S2667_raw:GT=hom:DP=121:FQ=1:SQ=-1;
2	1,67E+08	1,67E+08	A	G	upstream	TTC21B	.	dist=87	rs1347041	.	ID=S2009_raw:GT=hom:DP=17:FQ=1:SQ=-1;ID=S2503_raw:GT=hom:DP=69:FQ=1:SQ=-1;ID=S2507_raw:GT=hom:DP=66:FQ=1:SQ=-1;ID=S2668_raw:GT=hom:DP=49:FQ=1:SQ=-1;ID=S2012_raw:GT=hom:DP=19:FQ=1:SQ=-1;ID=S2010_raw:GT=hom:DP=23:FQ=1:SQ=-1;ID=S2662_raw:GT=hom:DP=113:FQ=1:SQ=-1;ID=S2663_raw:GT=hom:DP=72:FQ=1:SQ=-1;ID=S2011_raw:GT=hom:DP=35:FQ=1:SQ=-1;ID=S2504_raw:GT=hom:DP=63:FQ=1:SQ=-1;ID=S2008_raw:GT=hom:DP=21:FQ=1:SQ=-1;ID=S2509_raw:GT=hom:DP=64:FQ=1:SQ=-1;ID=S2508_raw:GT=hom:DP=80:FQ=1:SQ=-1;ID=S2666_raw:GT=hom:DP=26:FQ=1:SQ=-1;ID=S2669_raw:GT=hom:DP=77:FQ=1:SQ=-1;ID=S2166_raw:GT=hom:DP=60:FQ=1:SQ=-1;ID=S1775_raw:GT=hom:DP=36:FQ=1:SQ=-1;ID=S2501_raw:GT=hom:DP=52:FQ=1:SQ=-1;ID=S2500_raw:GT=hom:DP=39:FQ=1:SQ=-1;ID=S2502_raw:GT=hom:DP=45:FQ=1:SQ=-1;ID=S2505_raw:GT=hom:DP=51:FQ=1:SQ=-1;ID=S2176_raw:GT=hom:DP=33:FQ=1:SQ=-1;ID=S2506_raw:GT=hom:DP=51:FQ=1:SQ=-1;ID=S2667_raw:GT=hom:DP=75:FQ=1:SQ=-1;
2	1,67E+08	1,67E+08	T	-	upstream	TTC21B	.	dist=148	rs5836068	.	ID=S2009_raw:GT=hom:DP=6:FQ=1:SQ=-1;ID=S2503_raw:GT=hom:DP=20:FQ=1:SQ=-1;ID=S2507_raw:GT=hom:DP=17:FQ=1:SQ=-1;ID=S2668_raw:GT=hom:DP=10:FQ=1:SQ=-1;ID=S2012_raw:GT=hom:DP=4:FQ=1:SQ=-1;ID=S2010_raw:GT=hom:DP=8:FQ=1:SQ=-1;ID=S2662_raw:GT=hom:DP=23:FQ=1:SQ=-1;ID=S2663_raw:GT=hom:DP=24:FQ=1:SQ=-1;ID=S2011_raw:GT=hom:DP=15:FQ=1:SQ=-1;ID=S2504_raw:GT=hom:DP=19:FQ=1:SQ=-1;ID=S2008_raw:GT=hom:DP=4:FQ=1:SQ=-1;ID=S2509_raw:GT=hom:DP=17:FQ=1:SQ=-1;ID=S2508_raw:GT=hom:DP=26:FQ=1:SQ=-1;ID=S2666_raw:GT=hom:DP=2:FQ=1:SQ=-1;ID=S2669_raw:GT=hom:DP=11:FQ=1:SQ=-1;ID=S2166_raw:GT=hom:DP=24:FQ=1:SQ=-1;ID=S1775_raw:GT=hom:DP=15:FQ=1:SQ=-1;ID=S2501_raw:GT=hom:DP=16:FQ=1:SQ=-1;ID=S2500_raw:GT=hom:DP=11:FQ=1:SQ=-1;ID=S2502_raw:GT=hom:DP=14:FQ=1:SQ=-1;ID=S2505_raw:GT=hom:DP=20:FQ=1:SQ=-1;ID=S2176_raw:GT=hom:DP=16:FQ=1:SQ=-1;ID=S2506_raw:GT=hom:DP=17:FQ=1:SQ=-1;ID=S2667_raw:GT=hom:DP=20:FQ=1:SQ=-1;
18	77733976	77733976	A	G	intronic	TXNL4A	.	.	rs4799114	.	ID=S2009_raw:GT=hom:DP=2:FQ=1:SQ=-1;ID=S2503_raw:GT=hom:DP=12:FQ=1:SQ=-1;ID=S2507_raw:GT=hom:DP=5:FQ=1:SQ=-1;ID=S2668_raw:GT=hom:DP=5:FQ=1:SQ=-1;ID=S2012_raw:GT=hom:DP=2:FQ=1:SQ=-1;ID=S2010_raw:GT=hom:DP=3:FQ=1:SQ=-1;ID=S2662_raw:GT=hom:DP=2:FQ=1:SQ=-1;ID=S2663_raw:GT=hom:DP=7:FQ=1:SQ=-1;ID=S2011_raw:GT=hom:DP=6:FQ=1:SQ=-1;ID=S2504_raw:GT=hom:DP=10:FQ=1:SQ=-1;ID=S2008_raw:GT=hom:DP=1:FQ=1:SQ=-1;ID=S2509_raw:GT=hom:DP=8:FQ=1:SQ=-1;ID=S2508_raw:GT=hom:DP=4:FQ=1:SQ=-1;ID=S2666_raw:GT=hom:DP=3:FQ=1:SQ=-1;ID=S2669_raw:GT=hom:DP=4:FQ=1:SQ=-1;ID=S2166_raw:GT=hom:DP=8:FQ=1:SQ=-1;ID=S1775_raw:GT=hom:DP=6:FQ=1:SQ=-1;ID=S2501_raw:GT=hom:DP=8:FQ=1:SQ=-1;ID=S2500_raw:GT=hom:DP=8:FQ=1:SQ=-1;ID=S2502_raw:GT=hom:DP=9:FQ=1:SQ=-1;ID=S2505_raw:GT=hom:DP=14:FQ=1:SQ=-1;ID=S2176_raw:GT=hom:DP=8:FQ=1:SQ=-1;ID=S2506_raw:GT=hom:DP=6:FQ=1:SQ=-1;ID=S2667_raw:GT=hom:DP=9:FQ=1:SQ=-1;
19	34942872	34942872	A	T	intronic	UBA2	.	.	rs2161472	.	ID=S2009_raw:GT=hom:DP=7:FQ=1:SQ=-1;ID=S2503_raw:GT=hom:DP=21:FQ=1:SQ=-1;ID=S2507_raw:GT=hom:DP=22:FQ=1:SQ=-1;ID=S2668_raw:GT=hom:DP=20:FQ=1:SQ=-1;ID=S2012_raw:GT=hom:DP=6:FQ=1:SQ=-1;ID=S2010_raw:GT=hom:DP=6:FQ=1:SQ=-1;ID=S2662_raw:GT=hom:DP=3:FQ=1:SQ=-1;ID=S2663_raw:GT=hom:DP=21:FQ=1:SQ=-1;ID=S2011_raw:GT=hom:DP=9:FQ=1:SQ=-1;ID=S2504_raw:GT=hom:DP=11:FQ=1:SQ=-1;ID=S2008_raw:GT=hom:DP=3:FQ=1:SQ=-1;ID=S2509_raw:GT=hom:DP=23:FQ=1:SQ=-1;ID=S2508_raw:GT=hom:DP=30:FQ=1:SQ=-1;ID=S2666_raw:GT=hom:DP=3:FQ=1:SQ=-1;ID=S2669_raw:GT=hom:DP=18:FQ=1:SQ=-1;ID=S2166_raw:GT=hom:DP=23:FQ=1:SQ=-1;ID=S1775_raw:GT=hom:DP=12:FQ=1:SQ=-1;ID=S2501_raw:GT=hom:DP=27:FQ=1:SQ=-1;ID=S2500_raw:GT=hom:DP=27:FQ=1:SQ=-1;ID=S2502_raw:GT=hom:DP=16:FQ=1:SQ=-1;ID=S2505_raw:GT=hom:DP=15:FQ=1:SQ=-1;ID=S2176_raw:GT=hom:DP=25:FQ=1:SQ=-1;ID=S2506_raw:GT=hom:DP=47:FQ=1:SQ=-1;ID=S2667_raw:GT=hom:DP=34:FQ=1:SQ=-1;
13	99853443	99853443	T	G	UTR5	UBAC2	.	NM_177967:c.-223T>G	rs7338947	.	ID=S2009_raw:GT=hom:DP=58:FQ=1:SQ=-1;ID=S2503_raw:GT=hom:DP=53:FQ=1:SQ=-1;ID=S2507_raw:GT=hom:DP=65:FQ=1:SQ=-1;ID=S2668_raw:GT=hom:DP=44:FQ=1:SQ=-1;ID=S2012_raw:GT=hom:DP=59:FQ=1:SQ=-1;ID=S2010_raw:GT=hom:DP=48:FQ=1:SQ=-1;ID=S2662_raw:GT=hom:DP=101:FQ=1:SQ=-1;ID=S2663_raw:GT=hom:DP=55:FQ=1:SQ=-1;ID=S2011_raw:GT=hom:DP=91:FQ=1:SQ=-1;ID=S2504_raw:GT=hom:DP=53:FQ=1:SQ=-1;ID=S2008_raw:GT=hom:DP=66:FQ=1:SQ=-1;ID=S2509_raw:GT=hom:DP=46:FQ=1:SQ=-1;ID=S2508_raw:GT=hom:DP=47:FQ=1:SQ=-1;ID=S2666_raw:GT=hom:DP=17:FQ=1:SQ=-1;ID=S2669_raw:GT=hom:DP=75:FQ=1:SQ=-1;ID=S2166_raw:GT=hom:DP=39:FQ=1:SQ=-1;ID=S1775_raw:GT=hom:DP=28:FQ=1:SQ=-1;ID=S2501_raw:GT=hom:DP=40:FQ=1:SQ=-1;ID=S2500_raw:GT=hom:DP=53:FQ=1:SQ=-1;ID=S2502_raw:GT=hom:DP=50:FQ=1:SQ=-1;ID=S2505_raw:GT=hom:DP=57:FQ=1:SQ=-1;ID=S2176_raw:GT=hom:DP=32:FQ=1:SQ=-1;ID=S2506_raw:GT=hom:DP=33:FQ=1:SQ=-1;ID=S2667_raw:GT=hom:DP=59:FQ=1:SQ=-1;
3	1,13E+08	1,13E+08	A	G	exonic	USF3	nonsynonymous SNV	USF3:NM_001009899:exon7:c.6599T>C:p.V2200A	rs930818	.	ID=S2009_raw:GT=hom:DP=129:FQ=1:SQ=-1;ID=S2503_raw:GT=hom:DP=85:FQ=1:SQ=-1;ID=S2507_raw:GT=hom:DP=112:FQ=1:SQ=-1;ID=S2668_raw:GT=hom:DP=126:FQ=1:SQ=-1;ID=S2012_raw:GT=hom:DP=138:FQ=1:SQ=-1;ID=S2010_raw:GT=hom:DP=125:FQ=1:SQ=-1;ID=S2662_raw:GT=hom:DP=16:FQ=1:SQ=-1;ID=S2663_raw:GT=hom:DP=84:FQ=1:SQ=-1;ID=S2011_raw:GT=hom:DP=183:FQ=1:SQ=-1;ID=S2504_raw:GT=hom:DP=88:FQ=1:SQ=-1;ID=S2008_raw:GT=hom:DP=122:FQ=1:SQ=-1;ID=S2509_raw:GT=hom:DP=74:FQ=1:SQ=-1;ID=S2508_raw:GT=hom:DP=88:FQ=1:SQ=-1;ID=S2666_raw:GT=hom:DP=119:FQ=1:SQ=-1;ID=S2669_raw:GT=hom:DP=70:FQ=1:SQ=-1;ID=S2166_raw:GT=hom:DP=236:FQ=1:SQ=-1;ID=S1775_raw:GT=hom:DP=223:FQ=1:SQ=-1;ID=S2501_raw:GT=hom:DP=93:FQ=1:SQ=-1;ID=S2500_raw:GT=hom:DP=113:FQ=1:SQ=-1;ID=S2502_raw:GT=hom:DP=107:FQ=1:SQ=-1;ID=S2505_raw:GT=hom:DP=101:FQ=1:SQ=-1;ID=S2176_raw:GT=hom:DP=279:FQ=1:SQ=-1;ID=S2506_raw:GT=hom:DP=77:FQ=1:SQ=-1;ID=S2667_raw:GT=hom:DP=98:FQ=1:SQ=-1;
15	50773755	50773755	G	A	exonic	USP8	synonymous SNV	USP8:NM_001283049:exon9:c.1065G>A:p.Q355Q,USP8:NM_001128610:exon11:c.1296G>A:p.Q432Q,USP8:NM_005154:exon11:c.1296G>A:p.Q432Q	rs3131561	.	ID=S2009_raw:GT=hom:DP=68:FQ=1:SQ=-1;ID=S2503_raw:GT=hom:DP=81:FQ=1:SQ=-1;ID=S2507_raw:GT=hom:DP=81:FQ=1:SQ=-1;ID=S2668_raw:GT=hom:DP=83:FQ=1:SQ=-1;ID=S2012_raw:GT=hom:DP=97:FQ=1:SQ=-1;ID=S2010_raw:GT=hom:DP=91:FQ=1:SQ=-1;ID=S2662_raw:GT=hom:DP=6:FQ=1:SQ=-1;ID=S2663_raw:GT=hom:DP=46:FQ=1:SQ=-1;ID=S2011_raw:GT=hom:DP=126:FQ=1:SQ=-1;ID=S2504_raw:GT=hom:DP=94:FQ=1:SQ=-1;ID=S2008_raw:GT=hom:DP=90:FQ=1:SQ=-1;ID=S2509_raw:GT=hom:DP=74:FQ=1:SQ=-1;ID=S2508_raw:GT=hom:DP=84:FQ=1:SQ=-1;ID=S2666_raw:GT=hom:DP=83:FQ=1:SQ=-1;ID=S2669_raw:GT=hom:DP=70:FQ=1:SQ=-1;ID=S2166_raw:GT=hom:DP=76:FQ=1:SQ=-1;ID=S1775_raw:GT=hom:DP=54:FQ=1:SQ=-1;ID=S2501_raw:GT=hom:DP=68:FQ=1:SQ=-1;ID=S2500_raw:GT=hom:DP=62:FQ=1:SQ=-1;ID=S2502_raw:GT=hom:DP=82:FQ=1:SQ=-1;ID=S2505_raw:GT=hom:DP=65:FQ=1:SQ=-1;ID=S2176_raw:GT=hom:DP=82:FQ=1:SQ=-1;ID=S2506_raw:GT=hom:DP=90:FQ=1:SQ=-1;ID=S2667_raw:GT=hom:DP=92:FQ=1:SQ=-1;
X	1,55E+08	1,55E+08	C	-	intergenic	VAMP7;IL9R	.	dist=41724;dist=12089	.	.	ID=S2009_raw:GT=hom:DP=9:FQ=1:SQ=-1;ID=S2503_raw:GT=hom:DP=25:FQ=1:SQ=-1;ID=S2507_raw:GT=hom:DP=29:FQ=1:SQ=-1;ID=S2668_raw:GT=hom:DP=17:FQ=1:SQ=-1;ID=S2012_raw:GT=hom:DP=5:FQ=1:SQ=-1;ID=S2010_raw:GT=hom:DP=12:FQ=1:SQ=-1;ID=S2662_raw:GT=hom:DP=7:FQ=1:SQ=-1;ID=S2663_raw:GT=hom:DP=24:FQ=1:SQ=-1;ID=S2011_raw:GT=hom:DP=9:FQ=1:SQ=-1;ID=S2504_raw:GT=hom:DP=28:FQ=1:SQ=-1;ID=S2008_raw:GT=hom:DP=11:FQ=1:SQ=-1;ID=S2509_raw:GT=hom:DP=39:FQ=1:SQ=-1;ID=S2508_raw:GT=hom:DP=29:FQ=1:SQ=-1;ID=S2666_raw:GT=hom:DP=11:FQ=1:SQ=-1;ID=S2669_raw:GT=hom:DP=22:FQ=1:SQ=-1;ID=S2166_raw:GT=hom:DP=74:FQ=1:SQ=-1;ID=S1775_raw:GT=hom:DP=33:FQ=1:SQ=-1;ID=S2501_raw:GT=hom:DP=26:FQ=1:SQ=-1;ID=S2500_raw:GT=hom:DP=36:FQ=1:SQ=-1;ID=S2502_raw:GT=hom:DP=30:FQ=1:SQ=-1;ID=S2505_raw:GT=hom:DP=38:FQ=1:SQ=-1;ID=S2176_raw:GT=hom:DP=89:FQ=1:SQ=-1;ID=S2506_raw:GT=hom:DP=33:FQ=1:SQ=-1;ID=S2667_raw:GT=hom:DP=26:FQ=1:SQ=-1;
14	77919587	77919587	T	C	intronic	VIPAS39	.	.	rs2364158	.	ID=S2009_raw:GT=hom:DP=165:FQ=1:SQ=-1;ID=S2503_raw:GT=hom:DP=135:FQ=1:SQ=-1;ID=S2507_raw:GT=hom:DP=127:FQ=1:SQ=-1;ID=S2668_raw:GT=hom:DP=139:FQ=1:SQ=-1;ID=S2012_raw:GT=hom:DP=198:FQ=1:SQ=-1;ID=S2010_raw:GT=hom:DP=212:FQ=1:SQ=-1;ID=S2662_raw:GT=hom:DP=14:FQ=1:SQ=-1;ID=S2663_raw:GT=hom:DP=115:FQ=1:SQ=-1;ID=S2011_raw:GT=hom:DP=265:FQ=1:SQ=-1;ID=S2504_raw:GT=hom:DP=117:FQ=1:SQ=-1;ID=S2008_raw:GT=hom:DP=218:FQ=1:SQ=-1;ID=S2509_raw:GT=hom:DP=102:FQ=1:SQ=-1;ID=S2508_raw:GT=hom:DP=91:FQ=1:SQ=-1;ID=S2666_raw:GT=hom:DP=99:FQ=1:SQ=-1;ID=S2669_raw:GT=hom:DP=73:FQ=1:SQ=-1;ID=S2166_raw:GT=hom:DP=184:FQ=1:SQ=-1;ID=S1775_raw:GT=hom:DP=150:FQ=1:SQ=-1;ID=S2501_raw:GT=hom:DP=105:FQ=1:SQ=-1;ID=S2500_raw:GT=hom:DP=78:FQ=1:SQ=-1;ID=S2502_raw:GT=hom:DP=124:FQ=1:SQ=-1;ID=S2505_raw:GT=hom:DP=122:FQ=1:SQ=-1;ID=S2176_raw:GT=hom:DP=188:FQ=1:SQ=-1;ID=S2506_raw:GT=hom:DP=124:FQ=1:SQ=-1;ID=S2667_raw:GT=hom:DP=84:FQ=1:SQ=-1;
10	28822373	28822373	A	C	intronic	WAC	.	.	rs12767429	.	ID=S2009_raw:GT=hom:DP=17:FQ=1:SQ=-1;ID=S2503_raw:GT=hom:DP=37:FQ=1:SQ=-1;ID=S2507_raw:GT=hom:DP=46:FQ=1:SQ=-1;ID=S2668_raw:GT=hom:DP=36:FQ=1:SQ=-1;ID=S2012_raw:GT=hom:DP=15:FQ=1:SQ=-1;ID=S2010_raw:GT=hom:DP=24:FQ=1:SQ=-1;ID=S2662_raw:GT=hom:DP=52:FQ=1:SQ=-1;ID=S2663_raw:GT=hom:DP=53:FQ=1:SQ=-1;ID=S2011_raw:GT=hom:DP=20:FQ=1:SQ=-1;ID=S2504_raw:GT=hom:DP=22:FQ=1:SQ=-1;ID=S2008_raw:GT=hom:DP=23:FQ=1:SQ=-1;ID=S2509_raw:GT=hom:DP=42:FQ=1:SQ=-1;ID=S2508_raw:GT=hom:DP=53:FQ=1:SQ=-1;ID=S2666_raw:GT=hom:DP=13:FQ=1:SQ=-1;ID=S2669_raw:GT=hom:DP=32:FQ=1:SQ=-1;ID=S2166_raw:GT=hom:DP=62:FQ=1:SQ=-1;ID=S1775_raw:GT=hom:DP=3:FQ=1:SQ=-1;ID=S2501_raw:GT=hom:DP=32:FQ=1:SQ=-1;ID=S2500_raw:GT=hom:DP=39:FQ=1:SQ=-1;ID=S2502_raw:GT=hom:DP=32:FQ=1:SQ=-1;ID=S2505_raw:GT=hom:DP=19:FQ=1:SQ=-1;ID=S2176_raw:GT=hom:DP=34:FQ=1:SQ=-1;ID=S2506_raw:GT=hom:DP=41:FQ=1:SQ=-1;ID=S2667_raw:GT=hom:DP=44:FQ=1:SQ=-1;
X	48544650	48544650	A	G	intronic	WAS	.	.	rs235423	.	ID=S2009_raw:GT=hom:DP=6:FQ=1:SQ=-1;ID=S2503_raw:GT=hom:DP=8:FQ=1:SQ=-1;ID=S2507_raw:GT=hom:DP=12:FQ=1:SQ=-1;ID=S2668_raw:GT=hom:DP=33:FQ=1:SQ=-1;ID=S2012_raw:GT=hom:DP=7:FQ=1:SQ=-1;ID=S2010_raw:GT=hom:DP=10:FQ=1:SQ=-1;ID=S2662_raw:GT=hom:DP=25:FQ=1:SQ=-1;ID=S2663_raw:GT=hom:DP=34:FQ=1:SQ=-1;ID=S2011_raw:GT=hom:DP=7:FQ=1:SQ=-1;ID=S2504_raw:GT=hom:DP=15:FQ=1:SQ=-1;ID=S2008_raw:GT=hom:DP=13:FQ=1:SQ=-1;ID=S2509_raw:GT=hom:DP=7:FQ=1:SQ=-1;ID=S2508_raw:GT=hom:DP=12:FQ=1:SQ=-1;ID=S2666_raw:GT=hom:DP=24:FQ=1:SQ=-1;ID=S2669_raw:GT=hom:DP=12:FQ=1:SQ=-1;ID=S2166_raw:GT=hom:DP=11:FQ=1:SQ=-1;ID=S1775_raw:GT=hom:DP=3:FQ=1:SQ=-1;ID=S2501_raw:GT=hom:DP=26:FQ=1:SQ=-1;ID=S2500_raw:GT=hom:DP=14:FQ=1:SQ=-1;ID=S2502_raw:GT=hom:DP=31:FQ=1:SQ=-1;ID=S2505_raw:GT=hom:DP=17:FQ=1:SQ=-1;ID=S2176_raw:GT=hom:DP=1:FQ=1:SQ=-1;ID=S2506_raw:GT=hom:DP=12:FQ=1:SQ=-1;ID=S2667_raw:GT=hom:DP=32:FQ=1:SQ=-1;
8	1,26E+08	1,26E+08	-	C	intronic	WASHC5	.	.	rs11370883	Benign	ID=S2009_raw:GT=hom:DP=39:FQ=1:SQ=-1;ID=S2503_raw:GT=hom:DP=81:FQ=1:SQ=-1;ID=S2507_raw:GT=hom:DP=77:FQ=1:SQ=-1;ID=S2668_raw:GT=hom:DP=128:FQ=1:SQ=-1;ID=S2012_raw:GT=hom:DP=41:FQ=1:SQ=-1;ID=S2010_raw:GT=hom:DP=34:FQ=1:SQ=-1;ID=S2662_raw:GT=hom:DP=27:FQ=1:SQ=-1;ID=S2663_raw:GT=hom:DP=115:FQ=1:SQ=-1;ID=S2011_raw:GT=hom:DP=39:FQ=1:SQ=-1;ID=S2504_raw:GT=hom:DP=83:FQ=1:SQ=-1;ID=S2008_raw:GT=hom:DP=36:FQ=1:SQ=-1;ID=S2509_raw:GT=hom:DP=80:FQ=1:SQ=-1;ID=S2508_raw:GT=hom:DP=95:FQ=1:SQ=-1;ID=S2666_raw:GT=hom:DP=104:FQ=1:SQ=-1;ID=S2669_raw:GT=hom:DP=96:FQ=1:SQ=-1;ID=S2166_raw:GT=hom:DP=117:FQ=1:SQ=-1;ID=S1775_raw:GT=hom:DP=63:FQ=1:SQ=-1;ID=S2501_raw:GT=hom:DP=92:FQ=1:SQ=-1;ID=S2500_raw:GT=hom:DP=91:FQ=1:SQ=-1;ID=S2502_raw:GT=hom:DP=75:FQ=1:SQ=-1;ID=S2505_raw:GT=hom:DP=88:FQ=1:SQ=-1;ID=S2176_raw:GT=hom:DP=136:FQ=1:SQ=-1;ID=S2506_raw:GT=hom:DP=103:FQ=1:SQ=-1;ID=S2667_raw:GT=hom:DP=93:FQ=1:SQ=-1;
10	1,23E+08	1,23E+08	A	C	intronic	WDR11	.	.	rs1652726	.	ID=S2009_raw:GT=hom:DP=19:FQ=1:SQ=-1;ID=S2503_raw:GT=hom:DP=15:FQ=0.93:SQ=-1;ID=S2507_raw:GT=hom:DP=13:FQ=1:SQ=-1;ID=S2668_raw:GT=hom:DP=14:FQ=1:SQ=-1;ID=S2012_raw:GT=hom:DP=16:FQ=1:SQ=-1;ID=S2010_raw:GT=hom:DP=19:FQ=1:SQ=-1;ID=S2662_raw:GT=hom:DP=5:FQ=1:SQ=-1;ID=S2663_raw:GT=hom:DP=9:FQ=1:SQ=-1;ID=S2011_raw:GT=hom:DP=25:FQ=1:SQ=-1;ID=S2504_raw:GT=hom:DP=10:FQ=1:SQ=-1;ID=S2008_raw:GT=hom:DP=20:FQ=1:SQ=-1;ID=S2509_raw:GT=hom:DP=13:FQ=1:SQ=-1;ID=S2508_raw:GT=hom:DP=22:FQ=1:SQ=-1;ID=S2666_raw:GT=hom:DP=11:FQ=1:SQ=-1;ID=S2669_raw:GT=hom:DP=10:FQ=1:SQ=-1;ID=S2166_raw:GT=hom:DP=37:FQ=1:SQ=-1;ID=S1775_raw:GT=hom:DP=13:FQ=1:SQ=-1;ID=S2501_raw:GT=hom:DP=21:FQ=1:SQ=-1;ID=S2500_raw:GT=hom:DP=20:FQ=1:SQ=-1;ID=S2502_raw:GT=hom:DP=17:FQ=1:SQ=-1;ID=S2505_raw:GT=hom:DP=17:FQ=1:SQ=-1;ID=S2176_raw:GT=hom:DP=43:FQ=1:SQ=-1;ID=S2506_raw:GT=hom:DP=9:FQ=1:SQ=-1;ID=S2667_raw:GT=hom:DP=7:FQ=1:SQ=-1;
4	39205365	39205365	C	T	intronic	WDR19	.	.	rs1451817	.	ID=S2009_raw:GT=hom:DP=93:FQ=0.99:SQ=-1;ID=S2503_raw:GT=hom:DP=70:FQ=1:SQ=-1;ID=S2507_raw:GT=hom:DP=66:FQ=1:SQ=-1;ID=S2668_raw:GT=hom:DP=102:FQ=1:SQ=-1;ID=S2012_raw:GT=hom:DP=118:FQ=1:SQ=-1;ID=S2010_raw:GT=hom:DP=109:FQ=1:SQ=-1;ID=S2662_raw:GT=hom:DP=4:FQ=1:SQ=-1;ID=S2663_raw:GT=hom:DP=65:FQ=1:SQ=-1;ID=S2011_raw:GT=hom:DP=159:FQ=1:SQ=-1;ID=S2504_raw:GT=hom:DP=66:FQ=1:SQ=-1;ID=S2008_raw:GT=hom:DP=128:FQ=1:SQ=-1;ID=S2509_raw:GT=hom:DP=60:FQ=1:SQ=-1;ID=S2508_raw:GT=hom:DP=83:FQ=1:SQ=-1;ID=S2666_raw:GT=hom:DP=100:FQ=1:SQ=-1;ID=S2669_raw:GT=hom:DP=40:FQ=1:SQ=-1;ID=S2166_raw:GT=hom:DP=243:FQ=1:SQ=-1;ID=S1775_raw:GT=hom:DP=249:FQ=1:SQ=-1;ID=S2501_raw:GT=hom:DP=47:FQ=1:SQ=-1;ID=S2500_raw:GT=hom:DP=86:FQ=1:SQ=-1;ID=S2502_raw:GT=hom:DP=81:FQ=1:SQ=-1;ID=S2505_raw:GT=hom:DP=63:FQ=1:SQ=-1;ID=S2176_raw:GT=hom:DP=266:FQ=1:SQ=-1;ID=S2506_raw:GT=hom:DP=61:FQ=1:SQ=-1;ID=S2667_raw:GT=hom:DP=68:FQ=1:SQ=-1;
19	36562292	36562292	G	A	intronic	WDR62	.	.	rs4806260	.	ID=S2009_raw:GT=hom:DP=13:FQ=1:SQ=-1;ID=S2503_raw:GT=hom:DP=6:FQ=1:SQ=-1;ID=S2507_raw:GT=hom:DP=12:FQ=1:SQ=-1;ID=S2668_raw:GT=hom:DP=14:FQ=1:SQ=-1;ID=S2012_raw:GT=hom:DP=20:FQ=1:SQ=-1;ID=S2010_raw:GT=hom:DP=15:FQ=1:SQ=-1;ID=S2662_raw:GT=hom:DP=7:FQ=1:SQ=-1;ID=S2663_raw:GT=hom:DP=6:FQ=1:SQ=-1;ID=S2011_raw:GT=hom:DP=24:FQ=1:SQ=-1;ID=S2504_raw:GT=hom:DP=12:FQ=1:SQ=-1;ID=S2008_raw:GT=hom:DP=15:FQ=1:SQ=-1;ID=S2509_raw:GT=hom:DP=8:FQ=1:SQ=-1;ID=S2508_raw:GT=hom:DP=9:FQ=1:SQ=-1;ID=S2666_raw:GT=hom:DP=17:FQ=1:SQ=-1;ID=S2669_raw:GT=hom:DP=7:FQ=1:SQ=-1;ID=S2166_raw:GT=hom:DP=15:FQ=1:SQ=-1;ID=S1775_raw:GT=hom:DP=18:FQ=1:SQ=-1;ID=S2501_raw:GT=hom:DP=9:FQ=1:SQ=-1;ID=S2500_raw:GT=hom:DP=10:FQ=1:SQ=-1;ID=S2502_raw:GT=hom:DP=17:FQ=1:SQ=-1;ID=S2505_raw:GT=hom:DP=11:FQ=1:SQ=-1;ID=S2176_raw:GT=hom:DP=19:FQ=1:SQ=-1;ID=S2506_raw:GT=hom:DP=19:FQ=1:SQ=-1;ID=S2667_raw:GT=hom:DP=13:FQ=1:SQ=-1;
8	30958360	30958360	T	-	splicing	WRN	.	NM_000553:exon18:c.1982-5T>-	.	.	ID=S2009_raw:GT=hom:DP=31:FQ=1:SQ=-1;ID=S2503_raw:GT=hom:DP=40:FQ=1:SQ=-1;ID=S2507_raw:GT=hom:DP=27:FQ=1:SQ=-1;ID=S2668_raw:GT=hom:DP=36:FQ=1:SQ=-1;ID=S2012_raw:GT=hom:DP=27:FQ=1:SQ=-1;ID=S2010_raw:GT=hom:DP=40:FQ=1:SQ=-1;ID=S2662_raw:GT=hom:DP=7:FQ=1:SQ=-1;ID=S2663_raw:GT=hom:DP=22:FQ=1:SQ=-1;ID=S2011_raw:GT=hom:DP=59:FQ=1:SQ=-1;ID=S2504_raw:GT=hom:DP=30:FQ=1:SQ=-1;ID=S2008_raw:GT=hom:DP=41:FQ=1:SQ=-1;ID=S2509_raw:GT=hom:DP=31:FQ=1:SQ=-1;ID=S2508_raw:GT=hom:DP=30:FQ=1:SQ=-1;ID=S2666_raw:GT=hom:DP=16:FQ=1:SQ=-1;ID=S2669_raw:GT=hom:DP=21:FQ=1:SQ=-1;ID=S2166_raw:GT=hom:DP=66:FQ=1:SQ=-1;ID=S1775_raw:GT=hom:DP=36:FQ=1:SQ=-1;ID=S2501_raw:GT=hom:DP=23:FQ=1:SQ=-1;ID=S2500_raw:GT=hom:DP=34:FQ=1:SQ=-1;ID=S2502_raw:GT=hom:DP=40:FQ=1:SQ=-1;ID=S2505_raw:GT=hom:DP=40:FQ=1:SQ=-1;ID=S2176_raw:GT=hom:DP=75:FQ=0.99:SQ=-1;ID=S2506_raw:GT=hom:DP=19:FQ=1:SQ=-1;ID=S2667_raw:GT=hom:DP=31:FQ=1:SQ=-1;
2	31567743	31567743	T	C	intronic	XDH	.	.	rs566224	.	ID=S2009_raw:GT=hom:DP=37:FQ=1:SQ=-1;ID=S2503_raw:GT=hom:DP=84:FQ=1:SQ=-1;ID=S2507_raw:GT=hom:DP=65:FQ=1:SQ=-1;ID=S2668_raw:GT=hom:DP=141:FQ=1:SQ=-1;ID=S2012_raw:GT=hom:DP=58:FQ=1:SQ=-1;ID=S2010_raw:GT=hom:DP=54:FQ=1:SQ=-1;ID=S2662_raw:GT=hom:DP=58:FQ=1:SQ=-1;ID=S2663_raw:GT=hom:DP=77:FQ=1:SQ=-1;ID=S2011_raw:GT=hom:DP=67:FQ=1:SQ=-1;ID=S2504_raw:GT=hom:DP=62:FQ=1:SQ=-1;ID=S2008_raw:GT=hom:DP=37:FQ=1:SQ=-1;ID=S2509_raw:GT=hom:DP=80:FQ=1:SQ=-1;ID=S2508_raw:GT=hom:DP=107:FQ=1:SQ=-1;ID=S2666_raw:GT=hom:DP=164:FQ=1:SQ=-1;ID=S2669_raw:GT=hom:DP=84:FQ=1:SQ=-1;ID=S2166_raw:GT=hom:DP=51:FQ=1:SQ=-1;ID=S1775_raw:GT=hom:DP=32:FQ=1:SQ=-1;ID=S2501_raw:GT=hom:DP=70:FQ=1:SQ=-1;ID=S2500_raw:GT=hom:DP=82:FQ=1:SQ=-1;ID=S2502_raw:GT=hom:DP=96:FQ=1:SQ=-1;ID=S2505_raw:GT=hom:DP=68:FQ=1:SQ=-1;ID=S2176_raw:GT=hom:DP=44:FQ=1:SQ=-1;ID=S2506_raw:GT=hom:DP=69:FQ=1:SQ=-1;ID=S2667_raw:GT=hom:DP=111:FQ=1:SQ=-1;
2	31609448	31609448	A	C	intronic	XDH	.	.	rs477626	.	ID=S2009_raw:GT=hom:DP=75:FQ=1:SQ=-1;ID=S2503_raw:GT=hom:DP=120:FQ=1:SQ=-1;ID=S2507_raw:GT=hom:DP=82:FQ=1:SQ=-1;ID=S2668_raw:GT=hom:DP=135:FQ=1:SQ=-1;ID=S2012_raw:GT=hom:DP=69:FQ=1:SQ=-1;ID=S2010_raw:GT=hom:DP=58:FQ=1:SQ=-1;ID=S2662_raw:GT=hom:DP=22:FQ=1:SQ=-1;ID=S2663_raw:GT=hom:DP=102:FQ=1:SQ=-1;ID=S2011_raw:GT=hom:DP=95:FQ=1:SQ=-1;ID=S2504_raw:GT=hom:DP=102:FQ=1:SQ=-1;ID=S2008_raw:GT=hom:DP=67:FQ=1:SQ=-1;ID=S2509_raw:GT=hom:DP=74:FQ=1:SQ=-1;ID=S2508_raw:GT=hom:DP=114:FQ=1:SQ=-1;ID=S2666_raw:GT=hom:DP=100:FQ=1:SQ=-1;ID=S2669_raw:GT=hom:DP=59:FQ=1:SQ=-1;ID=S2166_raw:GT=hom:DP=118:FQ=1:SQ=-1;ID=S1775_raw:GT=hom:DP=40:FQ=1:SQ=-1;ID=S2501_raw:GT=hom:DP=131:FQ=1:SQ=-1;ID=S2500_raw:GT=hom:DP=95:FQ=0.99:SQ=-1;ID=S2502_raw:GT=hom:DP=89:FQ=1:SQ=-1;ID=S2505_raw:GT=hom:DP=102:FQ=1:SQ=-1;ID=S2176_raw:GT=hom:DP=117:FQ=1:SQ=-1;ID=S2506_raw:GT=hom:DP=72:FQ=1:SQ=-1;ID=S2667_raw:GT=hom:DP=99:FQ=1:SQ=-1;
17	48432042	48432042	-	A	intronic	XYLT2	.	.	rs11430429	.	ID=S2009_raw:GT=hom:DP=11:FQ=1:SQ=-1;ID=S2503_raw:GT=hom:DP=75:FQ=1:SQ=-1;ID=S2507_raw:GT=hom:DP=62:FQ=1:SQ=-1;ID=S2668_raw:GT=hom:DP=89:FQ=1:SQ=-1;ID=S2012_raw:GT=hom:DP=3:FQ=1:SQ=-1;ID=S2010_raw:GT=hom:DP=6:FQ=1:SQ=-1;ID=S2662_raw:GT=hom:DP=118:FQ=1:SQ=-1;ID=S2663_raw:GT=hom:DP=69:FQ=1:SQ=-1;ID=S2011_raw:GT=hom:DP=3:FQ=1:SQ=-1;ID=S2504_raw:GT=hom:DP=85:FQ=1:SQ=-1;ID=S2008_raw:GT=hom:DP=9:FQ=1:SQ=-1;ID=S2509_raw:GT=hom:DP=81:FQ=1:SQ=-1;ID=S2508_raw:GT=hom:DP=85:FQ=1:SQ=-1;ID=S2666_raw:GT=hom:DP=66:FQ=1:SQ=-1;ID=S2669_raw:GT=hom:DP=49:FQ=1:SQ=-1;ID=S2166_raw:GT=hom:DP=63:FQ=1:SQ=-1;ID=S1775_raw:GT=hom:DP=34:FQ=1:SQ=-1;ID=S2501_raw:GT=hom:DP=69:FQ=1:SQ=-1;ID=S2500_raw:GT=hom:DP=68:FQ=1:SQ=-1;ID=S2502_raw:GT=hom:DP=91:FQ=1:SQ=-1;ID=S2505_raw:GT=hom:DP=80:FQ=1:SQ=-1;ID=S2176_raw:GT=hom:DP=55:FQ=1:SQ=-1;ID=S2506_raw:GT=hom:DP=84:FQ=1:SQ=-1;ID=S2667_raw:GT=hom:DP=81:FQ=1:SQ=-1;
17	48440528	48440528	-	CCA	intergenic	XYLT2;MRPL27	.	dist=1982;dist=4700	.	.	ID=S2009_raw:GT=hom:DP=125:FQ=1:SQ=-1;ID=S2503_raw:GT=hom:DP=66:FQ=1:SQ=-1;ID=S2507_raw:GT=hom:DP=71:FQ=1:SQ=-1;ID=S2668_raw:GT=hom:DP=74:FQ=1:SQ=-1;ID=S2012_raw:GT=hom:DP=147:FQ=1:SQ=-1;ID=S2010_raw:GT=hom:DP=164:FQ=1:SQ=-1;ID=S2662_raw:GT=hom:DP=69:FQ=1:SQ=-1;ID=S2663_raw:GT=hom:DP=58:FQ=1:SQ=-1;ID=S2011_raw:GT=hom:DP=200:FQ=1:SQ=-1;ID=S2504_raw:GT=hom:DP=58:FQ=1:SQ=-1;ID=S2008_raw:GT=hom:DP=146:FQ=1:SQ=-1;ID=S2509_raw:GT=hom:DP=70:FQ=1:SQ=-1;ID=S2508_raw:GT=hom:DP=61:FQ=1:SQ=-1;ID=S2666_raw:GT=hom:DP=86:FQ=1:SQ=-1;ID=S2669_raw:GT=hom:DP=52:FQ=1:SQ=-1;ID=S2166_raw:GT=hom:DP=95:FQ=1:SQ=-1;ID=S1775_raw:GT=hom:DP=111:FQ=1:SQ=-1;ID=S2501_raw:GT=hom:DP=56:FQ=1:SQ=-1;ID=S2500_raw:GT=hom:DP=48:FQ=1:SQ=-1;ID=S2502_raw:GT=hom:DP=49:FQ=1:SQ=-1;ID=S2505_raw:GT=hom:DP=42:FQ=1:SQ=-1;ID=S2176_raw:GT=hom:DP=118:FQ=1:SQ=-1;ID=S2506_raw:GT=hom:DP=73:FQ=1:SQ=-1;ID=S2667_raw:GT=hom:DP=49:FQ=1:SQ=-1;
13	20608354	20608354	-	TTTCTTGAGGTG	intronic	ZMYM2	.	.	rs11272380	.	ID=S2009_raw:GT=hom:DP=26:FQ=1:SQ=-1;ID=S2503_raw:GT=hom:DP=26:FQ=1:SQ=-1;ID=S2507_raw:GT=hom:DP=30:FQ=1:SQ=-1;ID=S2668_raw:GT=hom:DP=28:FQ=1:SQ=-1;ID=S2012_raw:GT=hom:DP=54:FQ=1:SQ=-1;ID=S2010_raw:GT=hom:DP=46:FQ=1:SQ=-1;ID=S2662_raw:GT=hom:DP=1:FQ=1:SQ=-1;ID=S2663_raw:GT=hom:DP=18:FQ=1:SQ=-1;ID=S2011_raw:GT=hom:DP=71:FQ=1:SQ=-1;ID=S2504_raw:GT=hom:DP=26:FQ=1:SQ=-1;ID=S2008_raw:GT=hom:DP=53:FQ=1:SQ=-1;ID=S2509_raw:GT=hom:DP=17:FQ=1:SQ=-1;ID=S2508_raw:GT=hom:DP=28:FQ=1:SQ=-1;ID=S2666_raw:GT=hom:DP=18:FQ=1:SQ=-1;ID=S2669_raw:GT=hom:DP=10:FQ=1:SQ=-1;ID=S2166_raw:GT=hom:DP=34:FQ=1:SQ=-1;ID=S1775_raw:GT=hom:DP=29:FQ=1:SQ=-1;ID=S2501_raw:GT=hom:DP=21:FQ=1:SQ=-1;ID=S2500_raw:GT=hom:DP=27:FQ=1:SQ=-1;ID=S2502_raw:GT=hom:DP=21:FQ=1:SQ=-1;ID=S2505_raw:GT=hom:DP=31:FQ=1:SQ=-1;ID=S2176_raw:GT=hom:DP=45:FQ=1:SQ=-1;ID=S2506_raw:GT=hom:DP=18:FQ=1:SQ=-1;ID=S2667_raw:GT=hom:DP=14:FQ=1:SQ=-1;
